# Significance of Visible Non-Invasive Risk Attributes for the Initial Prediction of Heart Disease Using Different Machine Learning Techniques

**DOI:** 10.1155/2022/9580896

**Published:** 2022-02-21

**Authors:** Syed Immamul Ansarullah, Syed Mohsin Saif, Pradeep Kumar, Mudasir Manzoor Kirmani

**Affiliations:** ^1^Lecturer at the Department of Computer Science, Cluster University, Jammu, India; ^2^Assistant Professor at the Department of Information Technology, IUST Awantipora, Kashmir, India; ^3^Associate Professor at the Department of Computer Science and Information Technology, MANUU, Hyderabad, India; ^4^Assistant Professor at the Department of Fisheries, SKAUST, Kashmir, India

## Abstract

**Introduction:**

Heart disease is emerging as the single most critical cause of death worldwide and is one of the costliest chronic conditions.

**Purpose:**

Stimulated by the increasing heart disease mortality rate incidents, an effective, low-cost, and reliable heart disease risk evaluation model is developed using significant non-invasive risk attributes. The significant non-invasive risk attributes like (age, systolic BP, diastolic BP, BMI, hereditary factor, smoking, alcohol, and physical inactivity) are identified by the help of medical domain experts, and their reliability in heart disease prediction is investigated through different feature selection techniques. *Methodology*. The enhancements of applying specific investigated techniques like random forest, Naïve Bayes, decision tree, support vector machine, and K nearest neighbor to the risk factors are tested. The heart disease risk assessment model is developed using the Jupyter Notebook web application, and its performance is tested not only through medical domain measures but also through the model performance measures. *Findings*. To evaluate heart disease risk evaluation model, we calculated measures of discrimination like error rate, AUROC, sensitivity, specificity, accuracy, precision, and so on. Experimental results show that the random forest heart disease risk evaluation model outperforms other existing risk models with admirable predictive accuracy and minimum misclassification rate. *Originality.* The heart disease risk evaluation model is developed based on novel non-invasive heart disease dataset, which consists of 5776 records. This dataset is collected from different heterogeneous data sources of Kashmir (India) through quantitative data collection methods. *Research Implications.* The risk model is applicable where people lack the facilities of integrated primary medical care technologies for untimely heart disease risk prediction. *Future Work.* To investigate deep learning and study the significance of other controlled attributes on different age and sex groups in the risk estimation of heart disease.

## 1. Introduction

Heart disease is the most influential socioeconomic and public health problem, which has potentially affected both genders with a significant number of causalities and other disabilities [1, 2]. Regardless of being among the most widespread chronic condition leading to a large percentage of disability and mortality across the globe, heart disease is recognized to be among the most avoidable and controllable diseases [3]. Initial identification of cardiac disorder victims can benefit from recuperating patients' health and diminishing the death ratio [4]. If we are to reduce the alarming circumstances emerging out from heart disease, it is implicit to recognize its causal factors that have pushed the world to an unfavorable situation [5]. It is widely accepted that risk factors like age, harmful intake of alcohol, unhealthy diet, smoking, and stagnation are the significant risk attributes of heart disease and continuing exposure to these risk attributes results in raised hypertension, diabetes, dyslipidemia, obesity, and stroke [6–12]. The initial prediction of heart disease decreases advancement to critical conditions and complexities [13, 14]. Hence, keeping in view its consequences, we developed a heart disease risk assessment model using machine learning techniques, which would help physicians in initial prediction with high predictive power.

## 2. Literature Review

In recent times, researchers made decisive contributions to predict heart disease using different machine learning techniques. Palaniappan and Awang [15] applied Naive Bayes, neural network, and decision tree on patient attributes and evaluated model's performance using lift chart and classification matrix. However, the model is only used by stake holders. Anooj [16] applied a weighted fuzzy rule to develop risk model and then evaluated its performance through neural network based system on UCI heart disease dataset. Taneja [17] collected transthoracic echocardiography dataset and applied J48 classifier, Naive Bayes, and multilayer perceptron to get the significant risk attributes. These researchers analyzed the model results, and it is observed that the model has optimal accuracy and high specificity rate. Sujata and Nair [18] applied decision tree, Naïve Bayes, and K nearest neighbor techniques for initial prediction of heart disease. Purushottam et al. [19] applied support vector machine, C4.5, neural network, PART, multilayer perceptron, and radial basis function to find out the relationship between several patients and to find out the cause of heart disease. Kim and Kang [20] collected Korean heart disease dataset of 4146 records and applied neural network using feature correlation analysis to identify significant risk attributes and identify existence of correlations between feature relations. The proposed model outperformed Framingham risk score. Haq et al. [21] developed hybrid heart disease model on Cleveland dataset and applied classification algorithms and selection algorithms to select important attributes. They used 10-fold cross-validation for system validation and checked the performance of the classifiers using seven different evaluation metrics. Shah et al. [22] collected Z-Alizadeh Sani from Iranian patients and applied ten machine learning algorithms to predict heart disease. They introduced N2Genetic optimizer that provided optimal accuracy and F-1 score while predicting heart disease risk. Budholiya et al. [23] proposed an approach to identify important heart disease risk attributes using mean Fisher based feature selection algorithm and accuracy based feature selection algorithm. Researchers used principal component analysis to refine the selected feature subset, and the resulting feature subset is used for the classification purpose through RBF-based support vector machine (SVM). Martins et al. [24] applied Bayesian optimization XG boost classifier and one-hot encoding technique to predict heart disease. The performance of the model is evaluated on Cleveland heart disease dataset, and the results are compared with different existing models. Barik et al. [25] applied decision tree, optimized decision tree, random forest, and other algorithms to predict heart disease at its initial stages. These risk models were developed using RapidMiner and WEKA tool and were analyzed based on accuracy, precision, sensitivity, and specificity. Though different methods and algorithms were used to predict heart disease with optimal accuracy in state-of-the-art research; however, some performed with less efficiency. Our research focus on identifying the significant non-invasive heart disease risk attributes by using different feature selection techniques and classification algorithms. The results obtained are a measure to indicate how these techniques can efficiently be used in medical field.

## 3. Research Design for Heart Disease Risk Evaluation Model

**Figure 1:**
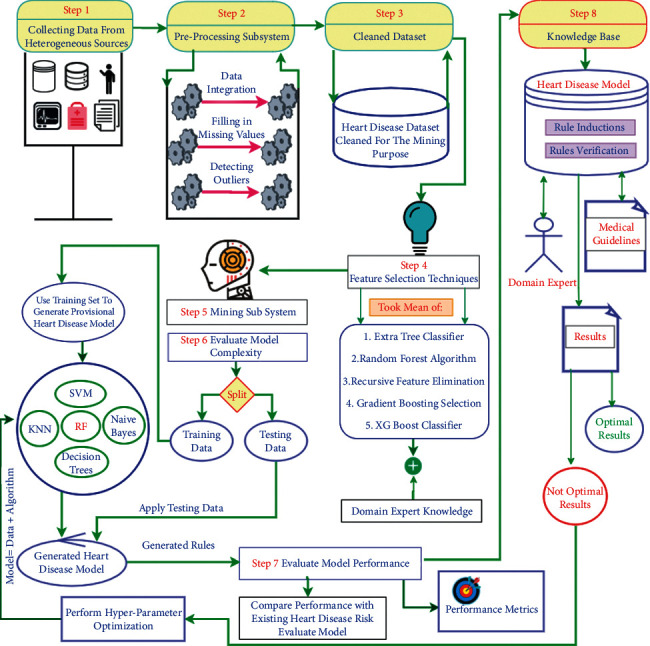
Research design for initial heart disease risk prediction.

To build an efficient heart disease risk evaluation model, we formulate the research design which is described in Figure 1.

The proposed research design consists of four main phases.

### 3.1. Data Phase

The data phase contains the whole process from data collection to feature engineering. This phase includes the qualitative data collection, the preprocessing subsystem, the cleaned data set storage, and the feature selection step. In this phase, the basic statistical description is performed to learn about each attribute value of the heart disease dataset. The heart disease dataset consists of a combination of nominal and numeric risk attributes. The missing numeric values are removed through the simple mean imputation method, and categorical missing values are filled by mode imputation technique. We performed class balance test on the heart disease dataset because highly imbalanced data makes the machine learning algorithms biased. After analysis, it is found that the heart disease dataset is balanced and has a skewness of (−0.03065287) and kurtosis of (−2.000136). These values of skewness and kurtosis represent that the heart disease dataset values are normally distributed.

The dataset contains 5776 records, of which 2745 (47.5%) have heart disease, and 3031 (52.5%) are healthy. Heart disease affects both men and women approximately in the same proportion with substantial death rates and disabilities, and predicting it accurately constitutes several basic causes like social, commercial, and cultural transition. The long-term disclosure to these risk attributes affects the hardest and ends up in death.

#### 3.1.1. Finding Correlation among Different Heart Disease Risk Attributes

**Figure 2:**
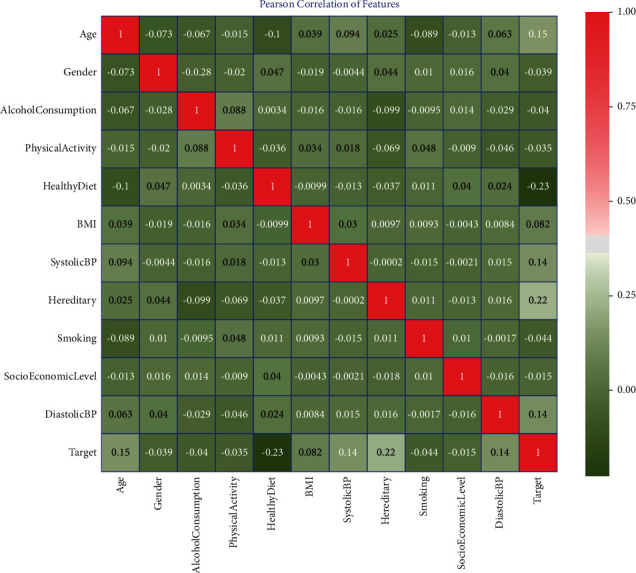
Correlation among different heart disease risk attributes.

In this research, Pearson's correlation is applied to check the mutual relationship among the heart disease attributes. The result of the applied Pearson's correlation coefficients among the heart disease risk attributes is shown in Figure 2 in the form of heatmap representation. The heatmap grid represents the correlation between the heart disease attributes with their corresponding coefficients. After heatmap analysis, we found that independent attributes are loosely correlated with one another, which are a good sign to improve the performance of the model. However, if the attributes in a dataset are tightly correlated (called multicollinearity), then a change in one variable can lead to change to another variable that can deteriorate the performance of an algorithm. Correlation among the attributes does not mean causation; hence, the strong relationship among attributes should be evaluated significantly. Mostly, a relationship among attributes may look causal through strong correlation because of some overlooked factors.

#### 3.1.2. Feature Selection Techniques for Heart Disease Risk Assessment

**Figure 3:**
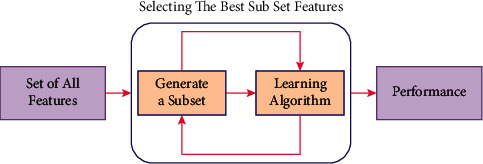
Working of feature selection techniques to select the best subset of features.

In this study, filter, wrapper, and embedded feature selection methods are applied to get an appropriate subset of feature for initial heart disease risk evaluation. The five different feature elimination techniques (extra tree classifier, gradient boosting classifier, random forest, recursive feature elimination, and XG boost classifier) are used as shown in Figure 3.

Each risk attribute is weighted by these feature selection techniques as per their role in disease prediction. The applied feature selection techniques provide weight in between the scale of 0 to 1 to each risk attribute. The risk attribute with the mean value near to 1 are important and attributes with values near to 0 are less significant in predicting heart disease.

Table 1 show the different non-invasive heart disease risk attributes with their respective weights assigned by different feature selection techniques and the overall mean of all the techniques. These heart disease risk attributes were identified by professional cardiologists and many other general physicians who are working in the cardiology department at various hospitals across, India.

After analyzing the results, it is derived that the attributes (systolic BP, diastolic BP, age, BMI, hereditary, healthy diet, and physical activity) are the most significant for the early prediction of the heart disease. The highly weighted significant subset of risk features is used to develop the heart disease risk model.

### 3.2. Data Mining Phase

The heart disease dataset is mined through random forest, decision tree, support vector machine, K nearest neighbor, and Naive Bayes techniques with 10-fold cross-validation. Various medical and model domain performance metrics like sensitivity, specificity, accuracy, precision, AUROC score, misclassification rates, computational complexity, and comprehensibility are calculated to obtain the optimal and accurate results. The following subsections explain the experimental results obtained by different heart disease risk evaluation models.

### 3.3. Model Evaluation and Validation Phase

#### 3.3.1. Experimental Results of Decision Tree Model

**Figure 4:**
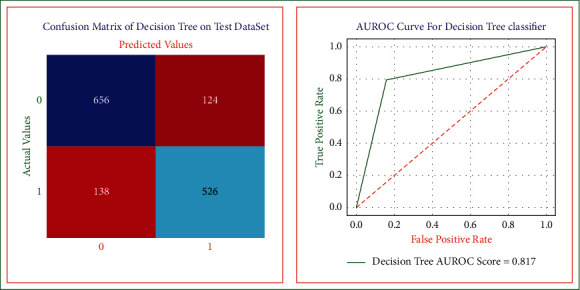
Decision tree confusion matrix and AUROC curve on the test dataset.

The rationale to apply a decision tree is to develop a heart disease risk evaluation model that can predict a class (diseased or healthy) by learning simple decision rules deduced from training data [25]. The cross-validation on the training dataset is used to get the unbiased results [26]. The performance results like sensitivity, specificity, accuracy, precision, error rates, and AUROC score are derived using decision tree model (Figure 4.

The sensitivity [26] of decision tree heart disease model is equal to 0.82. The closer the value for this measure is to 1, the better the rules are at identifying those patients who have heart disease. The specificity [27] of the model is equal to 0.80. The nearer the value for this measure is to 1, the best the rules are at identifying those patients without the disease. The overall accuracy of the decision tree model is equal to 0.81, which represents the decision tree heart disease model's overall performance (in diagnosing both the diseased and nondiseased heart disease cases). If accuracy of the model is high, then the model is more accurate in predicting the healthy and diseased cases. The precision is equal to 0.84. The closer the value for this measurement is to 1, the greater the chance that those with a positive outcome will have a disease. If a high precision rate of the decision tree model is obtained, then it means that the model will obtain a low false-positive rate. The error rate of this decision tree model is equivalent to 0.18. The lower the percentage of misclassification rate of the model is, the more accurate the model is in identifying the diseased and healthy cases. The AUROC score is equal to 0.81. The area under a correlation curve plotting true positive against false positive is higher for models best able to correctly identify positive and negative cases.

#### 3.3.2. Experimental Results of K-Nearest Neighbor Model

**Figure 5:**
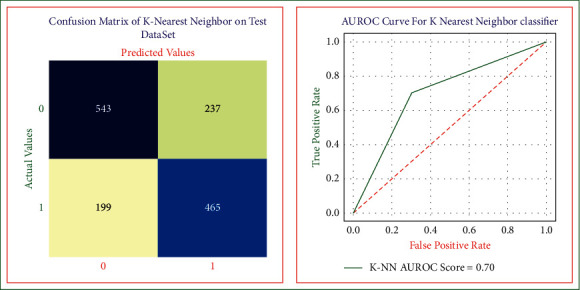
K nearest neighbor confusion matrix and AUROC curve on the test dataset.

The purpose of using the K nearest neighbor algorithm is to develop a risk evaluation model that can predict heart disease at its earliest. We used the 10-fold cross-validation on training data to get optimal and unbiased results [28, 29].  Figure 5 shows the K-NN heart disease model, and by using this, we obtained the sensitivity, specificity, accuracy, precision, error rate, and AUROC score equal to 0.73, 0.66, 0.69, 0.69, 0.30, and 0.70, respectively.

#### 3.3.3. Experimental Results of Support Vector Machine Model

**Figure 6:**
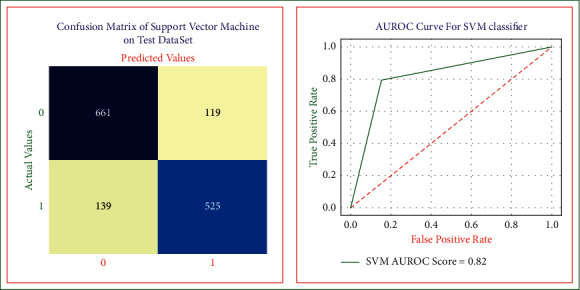
SVM risk model confusion matrix and AUROC on test dataset.

In this research, we used support vector machine model to predict heart disease in its early stages [30, 31]. The heart disease SVM model is shown in Figure 6 and using this model, we obtained the sensitivity, specificity, accuracy, precision, error rates, and AUROC score equal to 0.82, 0.81, 0.82, 0.84, 0.17, and 0.82, respectively.

#### 3.3.4. Experimental Results of Random Forest Model

**Figure 7:**
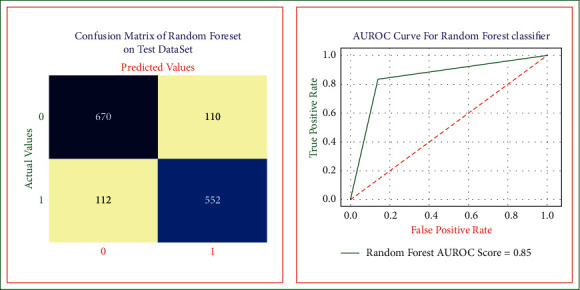
Random forest model confusion matrix and AUROC on test dataset.

The predictive results of the heart disease random forest model are shown in Figure 7 [32]. The random forest model recognizes different patient cases with a sensitivity of 0.85, specificity of 0.83, accuracy of 0.84, precision of 0.85, error rate of 0.15, and AUROC score of 0.85.

#### 3.3.5. Experimental Results of Naive Bayes Model

**Figure 8:**
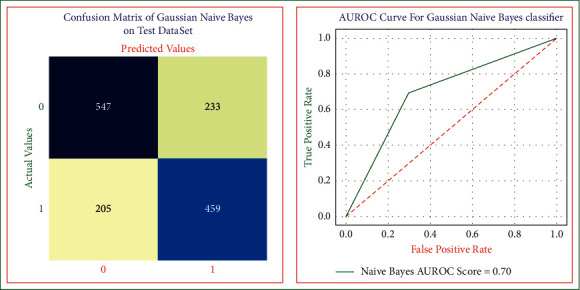
Naive Bayes model confusion matrix and AUROC on test dataset.

Naive Bayes risk evaluation model is shown in Figure 8 which is used to predict the heart disease at its initial stages. We applied 10-fold cross-validation on heart disease dataset to achieve the maximum accuracy and unbiased results. The performance results of the Gaussian Naive Bayes risk model are sensitivity equal to 0.72, specificity equal to 0.66, the overall accuracy equal to 0.69, precision equal to 0.70, error rate equal to 0.30, and AUROC score equal to 0.70.

### 3.4. Knowledge-Based Phase

**Figure 9:**
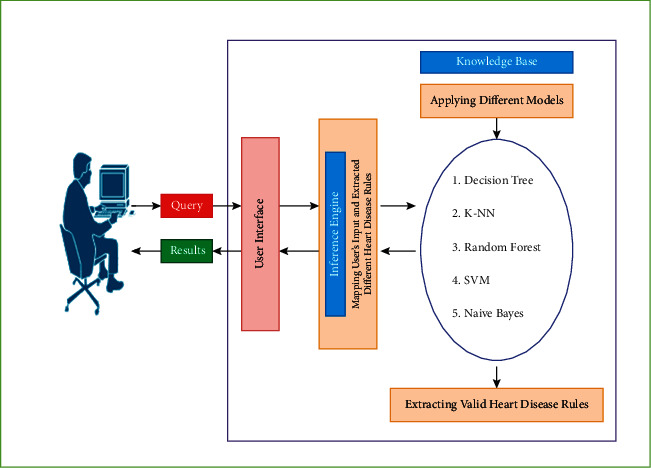
Heart disease expert system evaluation tool components.

The knowledge-based phase includes the steps to store and retrieve knowledge about heart disease. The generated heart disease risk rules would be stored in the knowledge base and cross-checked as per medical guidelines and domain expertise knowledge. The developed heart disease risk evaluation model is innovative because it identifies the degree of risk of heart disease patients using only the non-invasive risk attributes, thus supporting its application as a public screening test. For simplicity, we have called this model heart disease risk evaluation model (HDREM).  Figure 9 shows three main components of HDREM and their working: the knowledge base, inference engine, and the interface.

The knowledge base component applies the proposed models on non-invasive heart disease data attributes to extract the expert system rules. The inference engine uses the extracted rules, and the users' input component draws conclusions from the knowledge base and presents them to the user via the user interface. The user interface allows for “communication” screens where the user enters input data, and the expert system returns the degree of heart disease risk as calculated by the inference engine.

The results demonstrate that the combination of age, systolic BP, diastolic BP, BMI, healthy diet, hereditary, and physical activity provides the best results. The rules are extracted to create a chart as community screening tests to support healthcare experts predict the degree of risk of heart disease patients. An optimal set of predictive risk rules are generated using the above-derived attribute combinations, which help in the initial prediction of heart disease victims. The generated heart disease risk evaluation rules are pruned, evaluated, and validated by different medical domain experts; however, their use is restricted as the extracted rules are inductive because they are based on the specific ethnic heart disease dataset.

## 4. Consolidated Results of the Risk Model

We simulate the accomplished experimental results of the developed heart disease risk models with the prevailing research; the results obtained are the best based on the study conducted than the published results in the literature. However, there are some exceptions to every proposed heart disease risk assessment model, which are described as follows.

- In decision tree risk model, the derived decision tree rules are complex and large, which increases the time complexity of the risk evaluation model and makes the system slow.
- The K nearest neighbor model is not optimal for risk prediction because the misclassification rate is high. The computational complexity and the comprehensibility of the developed heart disease risk model are also high.
- Naive Bayes model is not the best for predicting heart disease because the misclassification rates are higher than the existing proposed models in the literature. Apart from medical domain performance measures, the computational complexity and the comprehensibility of the developed Naive Bayes model are high. The higher values of misclassification rate and model complexity factors restrain its applications because medical prediction models must satisfy greater prediction accuracy and a single misdiagnose can lead to severe consequences.

We also describe the performance and comparison of the proposed risk prediction models through different measures as described in Table 2. Experimental results demonstrate that the random forest model performs most excellent compared with other risk models. The performance of the developed heart disease risk evaluation model is tested with the prevailing risk tools, which demonstrate that the results are exceptionally encouraging with outstanding predictive accuracy. The results show that the random forest model outperforms other risk evaluation models with an optimal accuracy of 85%, specificity of 83%, sensitivity of 85%, precision of 85%, AUROC score of 85%, and with less misclassification rate of only 13%. The accuracy obtained by the random forest is highest for predicting heart disease and is not achieved by previous studies.

**Figure 10:**
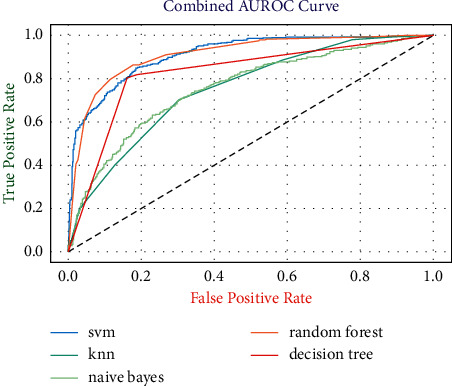
Combined AUROCs of the developed risk evaluation models.

Figure 10 shows the combined AUROC curves of different developed heart disease risk evaluation models. The random forest risk evaluation model has the highest AUROC score of 0.85, which means the model is highly skillful in predicting the diseased and healthy patients.

## 5. Results of the Non-Invasive Heart Disease Risk Attributes

Table 3 demonstrates the performance of various combinations of non-invasive risk attributes in early heart disease predictions. The combinations of systolic BP, diastolic BP, heredity, and age show the best accuracy of 77.3% obtained by the decision tree model. We also measure the sensitivity and specificity of all the attribute combinations. Here, sensitivity is most effective in diagnosing sick cases to provide proper care. By adding BMI (height and weight) attribute with the combination of (age, systolic BP, diastolic BP, and heredity), risk features accuracy is increased up to 78.9% by the random forest model. However, further combinations of the risk attributes with different permutations and combinations decrease the accuracy.

The results demonstrate that the combination of age, systolic BP, diastolic BP, BMI, healthy diet, hereditary, and physical activity provides the best results. The rules are extracted to create a chart as community screening tests to support healthcare experts diagnose the degree of risk of heart disease patients.

**Figure 11:**
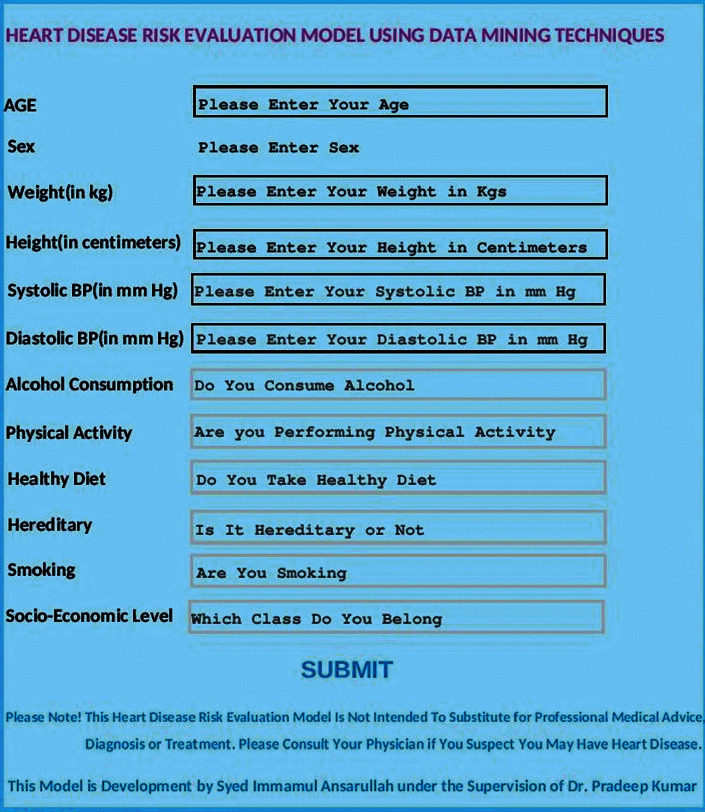
Heart disease risk evaluation model interface.

The developed model is implemented using the Python Jupyter Notebook web application.  Figure 11 shows the start screen of the model, where the user enters his/her data, and based on data, the degree of heart disease risk is calculated and displayed.

The simplicity of the user interface allows health care practitioners to identify patients at high risk of heart disease using very low-cost non-invasive attributes. The model is implemented on mobile as well as desktop applications.

## 6. Conclusion

We developed a non-invasive risk evaluation model that helps in the initial prediction of heart disease. The important and significant risk attributes are selected through careful analysis by cardiologists and different feature selection techniques. After weight assignment to every risk attribute through this process, the overall mean of all attribute weights is considered for the development of heart disease risk model. The higher numeric weight to an attribute is significant and plays a crucial role in predicting heart disease patients at its initial stage. Finally, data mining techniques use weighted risk attributes in predicting and diagnosing heart disease patients. The heart disease dataset is mined using the random forest, K nearest neighbor, support vector machine, decision tree, and Naive Bayes classifiers to discover if an individual possessing certain modifiable risk features will have the heart disease or not. The specificity, sensitivity, precision, accuracy, misclassification rate, and AUROC scores are calculated for each method using out-of-sample testing to check how accurately the risk evaluation model performs. Experimental results show that the random forest model outperforms other models with the highest sensitivity, specificity, precision, accuracy, AUROC score, and minimum misclassification rate. We simulate the accomplished outcomes against the prevailing research; the results obtained are, to the best of our perception, greater than published values in the literature. This risk model is applicable where people lack the facilities of the integrated primary medical care technologies for untimely heart disease risk prediction.

## 7. Future Work

In future, we can enhance the model using the following.

- The proposed research could be enhanced by investigating the performance of other robust machine learning techniques like deep learning
- The risk model could be enhanced by adding other non-invasive attributes
- The risk model could give optimal results by identifying the significance of controlled non-invasive attributes, such as weight and smoking on different age and sex groups in the risk estimation of heart disease

## Figures and Tables

**Table 1:** Feature selection techniques providing weight to each risk attribute.

Attributes	Feature selection techniques with their results and mean values					
	ETC	GBC	RF	RFE	XGB	MEAN
Age	0.92	0.92	0.87	0.25	0.92	0.78
Sex	0.0	0.0	0.11	0.83	0.0	0.19
Alcohol consumption	0.09	0.09	0.09	0.75	0.09	0.22
Physical activity	0.25	0.25	0.08	0.67	0.25	0.30
Healthy diet	0.71	0.71	0.52	1.0	0.71	0.73
BMI	0.74	0.74	0.79	0.0	0.74	0.60
Hereditary	0.38	0.38	0.4	0.92	0.38	0.49
Smoking	0.17	0.17	0.09	0.5	0.17	0.22
Systolic BP	1.0	1.0	1.0	0.08	1.0	0.82
Diastolic BP	0.88	0.88	0.78	0.33	0.88	0.75
Socio-economic level	0.17	0.17	0.11	0.42	0.17	0.21

**Table 2:** Performance measures of developed heart disease models.

	Performance measures					
Models	Sensitivity (%)	Specificity (%)	Accuracy (%)	Precision (%)	Error rate (%)	AUROC (%)
Decision tree	82	80	81	84	18	81
K nearest neighbor	73	66	70	69	30	70
Support vector machine	82	81	82	84	17	82
Random forest	85	83	84	85	15	85
Naive Bayes	72	66	69	70	30	70

**Table 3:** Integrating different non-invasive heart disease risk factors.

Techniques	Risk attributes	Sensitivity (%)	Specificity (%)	Accuracy (%)
Decision tree	Systolic BP, diastolic BP, age, heredity	78	80	77.3
	Systolic BP, diastolic BP, age, BMI	72	70	70.9
	Age, healthy diet, BMI	68	61	63.3
	Systolic BP, diastolic BP, age, physical activity	53	60	58.6
	Healthy diet, BMI, physical activity, age	58	41	50.9
	Healthy diet, physical activity, age, systolic BP, diastolic BP	45	43	42.5
	Physical activity, age, healthy diet, BMI, systolic BP, diastolic BP	38	30	38.2
	Age, physical activity, smoking, systolic BP, diastolic BP, healthy diet, alcohol consumption, BMI	30	28	42.7
K nearest neighbor	Age, healthy diet, alcohol consumption, smoking	42	45	38.2
	Age, BMI, healthy diet	70	60	67.9
	Age, BMI, alcohol consumption, smoking, sex	52	50	48.9
	BMI, systolic BP, diastolic BP, age, physical activity	38	35	42.7
	BMI, systolic BP, diastolic BP, age	68	74	72.5
	Age, systolic BP, BMI, diastolic BP, heredity	68	70	72.8
Random forest	Systolic BP, diastolic BP, age, healthy diet, smoking	51	48	45.4
	BMI, age, systolic BP, diastolic BP, heredity	72	78	78.9
	Alcohol consumption, physical activity, age, systolic BP, diastolic BP, BMI, smoking, healthy diet	35	45	58.7
	Age, sex, physical activity, BMI,	32	34	40.8
	Age, sex, physical activity, BMI, systolic BP, diastolic BP	39	45	42.6
Support vector machine	Systolic BP, diastolic BP, age	72	62	76.1
	Systolic BP, diastolic BP, age, BMI, heredity	70	78	75.2
	Healthy diet, age, BMI	41	53	50.9
	Systolic BP, diastolic BP, age, BMI, physical activity	50	44	51.6
	BMI, physical activity, alcohol consumption, age	49	50	52.4
	Age, alcohol consumption, BMI, healthy diet	41	59	52.2
Naive Bayes	Systolic BP, diastolic BP, age	74	78	75.1
	Age, alcohol consumption, healthy diet, sex, BMI	40	44	48.8
	Systolic BP, diastolic BP, age, BMI, heredity	68	75	77.2
	Systolic BP, diastolic BP, alcohol consumption, heredity, age, BMI, smoking, healthy diet, sex, physical activity,	46	51	50.6

## Data Availability

The heart disease risk data used to support the findings of this study are included within the supplementary information file.

## Supplementary Materials

Supplementary Materials The heart disease risk data used to support the findings of this study are included within the supplementary information file. Data of the heart disease risk are in the supplementary section.
