# Supplementary material for: Significance of Visible Non-Invasive Risk Attributes for the Initial Prediction of Heart Disease Using Different Machine Learning Techniques
Source: Comput Intell Neurosci. 2022 Feb 21;2022:9580896. doi: 10.1155/2022/9580896 (PMC8885242; doi:10.1155/2022/9580896)
Supplement: Supplementary Materials — The heart disease risk data used to support the findings of this study are included within the supplementary information file. Data of the heart disease risk are in the supplementary section. [file 9580896.f1.pdf]

Age	Gender	Height	Weight	Systolic BP	Diastolic BP	Hereditary
57	Female	140	52	92	68	Yes
63	Female	152	55	120	75	No
70	Male	155	56	108	78	Yes
60	Female	145	60	102	84	Yes
57	Male	146	60	101	80	Yes
50	Female	165	61	101	81	No
57	Male	148	80	142	90	Yes
57	Female	151	55	135	71	Yes
70	Male	145	50	98	66	Yes
48	Female	149	53	121	80	No
39	Female	145	54	131	78	Yes
60	Female	148	59	101	95	No
42	Female	152	76	135	88	Yes
39	Male	157	71	148	94	No
63	Female	156	68	145	72	Yes
52	Male	150	79	109	94	Yes
55	Female	152	85	152	67	Yes
53	Male	179	88	108	95	No
57	Female	152	87	128	87	Yes
58	Female	152	84	121	80	Yes
63	Female	152	82	111	84	No
62	Female	145	65	105	71	Yes
70	Female	156	68	122	95	Yes
44	Male	176	76	149	78	No
54	Female	180	87	110	75	Yes
63	Female	160	90	135	77	Yes
64	Female	150	79	150	69	No
60	Female	152	78	145	81	Yes
63	Female	145	79	147	85	Yes
53	Male	165	100	145	95	Yes
62	Male	176	99	145	71	Yes
45	Male	160	87	152	88	No
54	Female	165	90	121	81	Yes
42	Male	153	83	111	75	Yes
46	Male	153	76	109	78	No
42	Male	155	80	102	68	No
45	Female	156	76	101	81	Yes
55	Female	157	68	109	90	Yes
52	Male	156	64	108	80	Yes
67	Male	157	67	121	69	No
58	Male	158	63	118	88	No
57	Male	159	59	123	68	Yes
64	Male	161	61	132	73	No
61	Male	165	76	134	85	No
50	Female	166	72	151	88	No

43	Female	176	89	120	97	Yes
63	Female	171	66	134	78	No
41	Female	172	88	111	85	No
48	Male	143	68	108	66	Yes
66	Female	147	76	135	87	Yes
45	Female	149	59	110	75	Yes
76	Female	156	78	112	78	Yes
53	Male	155	77	110	88	No
48	Male	154	67	95	80	Yes
52	Female	152	76	142	91	No
57	Female	151	68	98	89	No
41	Male	153	69	132	78	Yes
58	Female	158	64	121	82	No
37	Male	179	70	102	81	No
58	Male	180	70	107	88	Yes
64	Male	180	89	101	75	Yes
66	Female	175	70	101	95	Yes
42	Male	176	79	120	74	Yes
55	Female	167	70	125	92	Yes
63	Female	178	79	110	83	No
58	Female	179	81	134	88	Yes
45	Male	174	70	148	95	Yes
35	Male	167	70	141	92	Yes
67	Male	168	70	129	80	Yes
58	Female	156	70	130	78	Yes
41	Female	159	73	145	87	No
44	Male	180	83	134	81	Yes
41	Male	174	84	134	95	Yes
62	Female	173	82	128	71	Yes
53	Female	156	76	132	72	No
62	Female	154	79	120	80	Yes
62	Male	154	78	120	78	Yes
66	Male	157	80	101	95	Yes
51	Male	159	86	142	88	No
70	Male	140	67	120	94	Yes
46	Male	152	66	140	88	Yes
51	Male	155	63	120	88	Yes
39	Male	145	58	104	82	Yes
57	Male	146	56	142	71	No
51	Male	165	67	121	92	Yes
66	Male	148	58	99	84	Yes
47	Male	153	61	118	81	Yes
57	Male	154	63	120	88	Yes
52	Female	165	67	148	94	No
53	Female	180	78	119	75	Yes
60	Female	150	79	101	73	Yes
71	Female	145	92	139	81	Yes

68	Female	141	54	118	88	Yes
64	Female	165	55	105	78	Yes
53	Male	155	59	121	81	Yes
34	Male	156	61	149	85	Yes
59	Male	155	61	114	71	Yes
54	Male	176	89	114	92	Yes
59	Male	178	89	101	75	Yes
60	Male	179	90	145	89	Yes
52	Female	145	60	135	92	No
63	Female	146	66	110	66	No
59	Female	165	64	104	77	Yes
74	Male	155	67	119	90	Yes
65	Female	152	61	152	89	Yes
52	Male	150	62	117	84	No
54	Male	151	85	146	88	No
49	Male	145	62	138	82	Yes
40	Male	149	82	101	66	Yes
53	Female	145	75	117	89	Yes
52	Male	148	72	123	78	No
62	Male	152	70	135	82	No
69	Male	157	67	101	92	Yes
49	Male	156	66	107	79	Yes
74	Female	145	79	123	80	Yes
61	Male	160	89	148	86	No
59	Male	165	90	118	65	No
60	Male	180	65	132	71	Yes
67	Female	162	94	109	90	Yes
56	Male	178	65	142	95	No
49	Female	145	49	142	65	Yes
65	Female	156	65	104	80	Yes
46	Male	176	65	105	85	Yes
44	Female	150	88	98	85	No
69	Male	179	65	121	75	No
64	Female	155	91	145	76	Yes
41	Male	165	65	149	88	Yes
51	Female	163	88	118	77	No
64	Male	165	65	152	68	Yes
59	Female	160	81	101	69	Yes
62	Female	178	65	132	71	Yes
54	Male	165	85	152	83	No
64	Male	153	80	100	88	No
62	Female	153	80	120	95	Yes
62	Female	155	65	151	92	Yes
42	Male	156	80	121	80	No
56	Male	157	80	99	78	Yes
54	Male	156	54	95	89	Yes
57	Male	157	80	111	81	Yes

63	Female	158	56	108	95	No
44	Female	159	67	121	71	Yes
54	Male	161	80	102	74	Yes
57	Male	165	68	152	80	Yes
54	Male	166	80	121	78	Yes
51	Male	165	73	142	95	Yes
39	Male	150	77	131	88	No
66	Female	150	79	148	80	Yes
59	Male	143	61	142	72	Yes
57	Male	147	85	129	94	Yes
48	Female	149	54	108	87	Yes
65	Female	156	50	135	95	Yes
64	Male	155	85	120	87	Yes
64	Male	154	85	140	80	Yes
55	Female	152	85	150	84	No
51	Female	151	51	112	71	No
60	Female	153	85	142	89	Yes
41	Female	158	55	148	78	No
58	Female	150	92	114	75	Yes
68	Female	160	88	131	77	Yes
55	Female	165	82	142	90	No
59	Male	155	81	151	88	No
56	Male	176	81	132	85	No
41	Male	167	55	138	95	No
48	Male	178	55	101	71	Yes
61	Male	179	78	110	88	Yes
54	Male	174	77	108	81	Yes
67	Female	167	55	104	75	Yes
56	Male	168	55	101	78	Yes
66	Male	156	55	145	68	No
50	Female	159	80	110	81	No
64	Female	165	80	132	90	Yes
64	Female	165	81	132	80	No
68	Male	173	55	123	69	Yes
37	Female	156	85	129	88	Yes
63	Female	154	85	118	68	Yes
64	Female	154	55	142	73	No
44	Male	157	55	131	85	No
54	Female	159	85	95	88	Yes
52	Male	140	55	131	97	Yes
42	Female	152	85	142	78	Yes
51	Female	155	85	138	85	Yes
60	Female	145	85	105	66	Yes
43	Female	146	58	102	87	No
43	Female	165	85	128	75	No
59	Female	148	71	143	78	Yes
43	Female	153	72	122	88	Yes

41	Male	154	83	104	80	Yes
62	Female	165	90	120	91	Yes
59	Female	170	87	108	89	Yes
66	Female	170	88	144	78	No
63	Female	179	83	105	82	No
54	Male	139	90	124	81	Yes
61	Female	165	90	112	88	Yes
48	Male	167	90	140	75	Yes
58	Male	156	90	152	95	Yes
61	Male	155	90	112	74	Yes
63	Female	160	90	114	92	No
51	Male	178	67	118	83	No
55	Female	179	87	132	88	Yes
58	Male	145	120	108	95	Yes
57	Male	146	67	132	92	Yes
64	Female	165	120	145	80	Yes
77	Male	155	81	149	78	Yes
44	Male	152	65	99	89	No
44	Male	150	120	90	81	No
53	Male	151	120	111	88	Yes
69	Male	145	60	134	95	Yes
62	Female	149	120	104	92	Yes
58	Male	145	54	149	80	Yes
76	Female	148	51	121	78	Yes
54	Male	152	55	107	89	No
57	Male	157	51	120	81	No
66	Female	156	56	118	95	Yes
68	Male	170	78	142	71	Yes
67	Male	180	71	121	98	Yes
41	Male	179	76	140	80	Yes
43	Male	180	77	132	78	Yes
58	Male	174	65	120	95	No
51	Male	180	67	135	88	No
54	Male	145	71	123	94	Yes
66	Female	156	72	101	72	Yes
51	Female	176	77	141	90	Yes
70	Male	170	79	147	87	Yes
67	Male	179	75	132	95	Yes
54	Female	170	90	147	87	No
50	Male	155	56	149	80	No
47	Male	159	74	118	84	Yes
55	Female	165	90	147	71	Yes
43	Male	176	61	120	95	Yes
55	Male	178	65	120	78	Yes
65	Female	165	90	114	75	Yes
67	Male	153	49	149	77	No
35	Male	153	49	138	92	No

76	Female	155	90	121	88	Yes
58	Male	156	51	131	85	Yes
52	Female	157	90	138	82	Yes
60	Female	156	90	120	90	Yes
66	Male	157	56	121	90	Yes
77	Female	158	71	110	84	No
56	Male	159	77	126	88	No
56	Male	161	76	121	82	No
38	Male	167	78	97	77	No
65	Female	178	77	123	90	Yes
71	Female	179	71	100	88	No
57	Female	174	77	127	89	Yes
47	Male	167	70	109	92	No
42	Female	168	73	152	82	No
59	Male	156	69	118	87	No
54	Female	159	66	142	81	Yes
50	Female	180	86	99	82	Yes
68	Female	174	81	98	95	Yes
56	Male	173	83	102	98	Yes
55	Female	156	69	101	97	No
46	Male	154	78	100	95	Yes
57	Female	154	71	115	98	Yes
48	Male	157	71	114	91	No
45	Male	159	67	120	80	Yes
34	Male	140	68	123	78	No
56	Male	152	66	132	87	No
53	Male	155	67	111	90	No
52	Male	145	60	120	95	Yes
41	Male	146	66	151	92	No
63	Male	165	67	142	97	Yes
59	Female	148	64	123	90	Yes
44	Male	153	72	132	97	Yes
54	Female	154	73	128	93	No
65	Female	165	59	99	91	Yes
45	Female	180	81	135	95	Yes
64	Female	175	82	124	80	Yes
61	Female	160	85	120	85	Yes
66	Male	139	66	125	88	Yes
44	Female	165	80	132	86	Yes
60	Female	167	52	125	87	Yes
38	Male	156	55	128	89	No
44	Female	155	56	99	71	Yes
43	Male	176	60	98	99	Yes
48	Male	178	60	99	80	Yes
35	Female	179	61	99	78	No
49	Male	145	57	102	95	Yes
54	Male	146	57	108	88	Yes

66	Male	165	58	132	94	Yes
55	Female	155	70	142	72	No
55	Male	152	75	145	94	Yes
48	Male	150	79	152	87	Yes
52	Female	151	83	132	95	Yes
47	Female	145	52	120	87	No
52	Female	149	65	122	80	Yes
58	Male	145	67	118	84	Yes
45	Male	148	60	105	71	Yes
74	Female	152	67	120	95	No
48	Male	157	80	123	78	Yes
41	Female	156	81	126	75	Yes
54	Female	178	83	127	77	Yes
51	Female	181	60	128	99	No
53	Male	160	61	120	88	Yes
38	Male	180	65	123	85	Yes
60	Male	177	59	124	95	Yes
52	Male	149	57	125	90	No
41	Female	145	59	124	90	Yes
49	Female	156	55	120	84	Yes
43	Female	176	50	90	88	Yes
56	Female	180	53	93	82	No
58	Female	160	54	92	66	Yes
67	Female	174	59	94	89	Yes
44	Female	157	76	99	78	Yes
54	Female	165	71	100	82	No
35	Female	165	68	124	92	Yes
42	Female	176	79	125	79	Yes
57	Female	140	50	95	80	Not Known
63	Female	152	55	121	86	Not Known
70	Female	169	82	110	65	Not Known
60	Female	145	65	92	80	Not Known
57	Male	162	85	111	88	Yes
52	Female	165	61	108	90	No
57	Female	148	72	102	92	Yes
58	Male	172	80	101	98	Not Known
62	Male	172	90	101	95	Not Known
41	Male	165	70	142	90	No
45	Female	172	100	121	85	No
46	Male	178	65	148	88	Yes
74	Female	162	85	145	89	Not Known
54	Male	144	70	101	75	No
62	Female	165	88	108	77	Not Known
63	Female	172	88	132	90	Not Known
60	Male	180	100	112	88	Yes
61	Male	155	50	120	89	Not Known
60	Female	172	93	102	92	Not Known

51	Female	178	81	121	82	No
49	Male	179	85	120	87	Not Known
61	Female	145	74	119	81	Yes
43	Female	146	62	123	82	No
57	Male	165	75	98	95	Not Known
66	Female	177	90	142	88	Not Known
39	Female	152	57	130	97	Yes
42	Male	150	46	132	95	No
57	Male	175	95	96	98	Yes
70	Female	145	50	120	91	No
48	Female	149	53	115	80	Yes
39	Female	145	72	135	78	Not Known
60	Female	159	65	98	87	No
42	Female	175	85	121	90	Yes
39	Male	157	55	131	95	No
63	Male	177	75	101	92	No
52	Female	178	96	135	97	No
55	Female	178	105	148	82	Not Known
53	Female	162	92	145	90	No
57	Female	170	92	109	93	No
58	Female	168	102	152	78	No
63	Male	156	101	108	87	Not Known
62	Female	145	71	128	95	Yes
70	Female	156	75	121	87	Yes
44	Male	176	76	111	80	Yes
54	Female	140	68	105	84	No
63	Female	172	103	122	71	Yes
64	Male	174	93	149	95	Not Known
60	Male	178	109	110	78	Yes
63	Male	180	104	135	75	Not Known
53	Male	181	100	151	77	Yes
62	Male	176	95	145	99	Not Known
45	Male	178	80	147	88	Yes
54	Female	165	90	145	85	Not Known
42	Female	153	48	145	83	Yes
46	Female	153	76	152	71	Yes
42	Male	155	80	121	88	Not Known
45	Female	156	58	111	81	Not Known
55	Female	157	60	109	75	Yes
52	Female	156	50	102	78	Not Known
67	Male	178	50	101	68	Yes
58	Female	175	77	109	81	Not Known
57	Female	180	102	108	90	Not Known
64	Male	177	75	121	80	No
61	Male	165	55	118	69	No
50	Male	175	70	123	88	No
43	Male	176	95	132	68	Yes

63	Female	171	68	134	73	No
41	Male	172	89	151	85	Yes
48	Female	143	68	120	88	Yes
66	Female	147	76	134	97	Yes
51	Female	149	73	111	78	Yes
76	Female	156	73	108	85	Yes
53	Male	156	55	135	66	Not Known
48	Male	154	77	110	87	Not Known
52	Male	152	50	112	75	Yes
41	Female	153	80	110	78	Not Known
58	Male	179	64	95	88	Yes
37	Female	176	75	142	80	Yes
58	Female	156	77	98	87	No
64	Female	174	104	132	84	Yes
66	Female	164	83	121	90	No
42	Female	176	82	102	82	No
55	Female	178	75	107	74	No
45	Male	174	75	101	80	Yes
35	Female	167	76	101	88	No
67	Female	168	72	120	85	No
41	Female	170	76	125	90	Yes
44	Female	180	105	110	81	No
41	Female	159	52	134	78	Yes
62	Female	173	90	148	84	Yes
53	Female	156	65	110	88	No
62	Male	154	65	95	75	Not Known
62	Male	158	60	142	91	No
66	Male	178	103	98	85	Not Known
38	Male	159	75	132	74	No
70	Male	140	67	121	75	Not Known
46	Male	158	55	102	88	No
51	Male	155	58	107	91	Not Known
39	Male	145	68	101	80	Yes
51	Male	165	86	101	78	Yes
66	Female	170	73	120	81	Not Known
47	Male	168	60	125	80	Not Known
28	Female	165	67	110	69	No
53	Female	174	70	134	91	Yes
60	Female	174	70	148	86	Yes
71	Female	172	106	141	90	Not Known
68	Female	142	40	129	88	Not Known
64	Female	165	55	130	81	No
31	Male	167	75	145	65	Not Known
34	Male	165	80	134	68	Yes
59	Female	179	85	134	88	Yes
54	Male	176	98	128	88	No
59	Male	171	88	132	82	No

60	Male	179	74	120	71	Not Known
52	Female	148	51	120	92	Yes
59	Female	165	71	101	84	Not Known
74	Male	171	60	142	81	No
65	Male	152	71	120	88	Yes
52	Female	150	74	140	90	No
54	Female	151	74	120	75	No
49	Male	145	55	104	73	Yes
40	Male	149	55	142	81	No
31	Female	145	42	121	88	No
52	Male	148	72	99	78	Not Known
62	Male	180	70	118	81	Not Known
69	Female	179	80	120	85	Not Known
49	Male	156	78	148	71	Not Known
74	Female	169	99	119	92	Yes
61	Male	179	107	101	75	Not Known
59	Female	159	99	139	89	No
67	Female	170	100	118	92	Not Known
56	Male	157	98	105	66	No
49	Female	145	50	121	77	No
65	Female	156	70	149	90	Not Known
46	Male	176	76	114	90	Yes
44	Male	181	76	114	84	Yes
69	Male	181	100	101	88	Yes
64	Male	154	93	145	82	Not Known
41	Male	154	68	135	66	No
51	Female	154	107	110	89	No
64	Female	165	69	104	78	Not Known
62	Female	178	77	119	82	No
54	Male	178	86	152	92	No
64	Male	148	76	117	79	Yes
62	Female	153	73	150	80	Not Known
42	Female	156	77	138	86	Yes
56	Female	157	76	101	72	Yes
54	Male	180	65	117	71	No
44	Female	159	75	123	90	No
51	Male	176	96	135	95	Not Known
39	Male	171	68	101	65	No
66	Female	172	90	107	80	Not Known
48	Female	149	54	123	85	No
65	Female	156	52	148	85	Not Known
64	Female	145	75	118	75	Yes
55	Female	152	60	132	76	Not Known
51	Female	151	51	109	88	No
41	Female	158	67	142	77	No
68	Female	181	102	142	68	Yes
55	Female	181	100	104	69	No

56	Female	168	70	105	71	Yes
41	Female	166	101	98	78	No
48	Male	178	67	121	84	No
61	Female	179	86	145	78	Not Known
67	Female	154	62	149	88	Yes
56	Female	178	85	118	95	Not Known
66	Male	168	53	152	78	No
50	Female	180	79	101	78	Yes
68	Male	173	69	132	78	Not Known
37	Female	156	78	152	66	Not Known
44	Female	157	50	100	88	No
52	Female	140	45	120	66	No
42	Male	152	71	151	80	No
51	Male	155	73	121	80	Yes
43	Female	146	75	99	98	No
43	Male	165	75	95	80	Not Known
43	Female	153	49	111	90	No
41	Male	178	62	108	88	Yes
62	Female	177	69	121	67	Yes
61	Female	165	85	102	98	Yes
48	Female	167	88	152	80	Yes
61	Male	155	72	121	71	Not Known
51	Female	178	78	142	68	Not Known
55	Female	179	80	131	95	Yes
77	Female	155	54	148	88	No
44	Female	152	54	147	78	No
44	Male	150	65	129	64	No
31	Male	151	50	108	66	Yes
69	Male	169	65	135	84	Not Known
76	Female	169	74	120	81	Not Known
68	Female	178	102	140	71	Yes
67	Female	177	88	150	81	Not Known
41	Female	154	76	112	92	No
43	Female	173	72	142	80	No
51	Male	162	67	143	88	Yes
51	Female	176	77	114	92	No
70	Male	175	105	131	76	Not Known
67	Female	162	98	142	86	No
50	Male	162	56	151	69	No
47	Female	162	74	132	93	Yes
55	Male	165	70	138	90	Yes
43	Male	176	61	101	74	Yes
65	Female	165	83	110	84	Not Known
67	Female	153	62	108	82	Not Known
35	Female	153	50	104	90	No
76	Male	181	75	101	82	Not Known
52	Male	165	65	145	95	Yes

77	Female	158	85	110	98	Yes
56	Female	159	62	132	90	No
45	Female	166	66	132	98	No
32	Male	176	85	123	80	No
35	Female	147	45	129	95	Yes
45	Female	154	56	118	99	No
71	Female	153	74	142	80	Yes
47	Female	158	78	131	80	No
35	Female	180	110	95	83	No
38	Male	171	72	131	78	Yes
65	Female	178	77	142	86	Not Known
71	Male	179	71	138	88	No
47	Male	167	70	105	88	Yes
42	Female	168	80	102	88	No
50	Female	170	80	128	70	Not Known
68	Female	149	96	146	74	No
46	Male	162	78	122	92	Not Known
48	Female	157	56	104	87	Yes
45	Male	159	67	120	87	No
34	Male	140	45	108	66	No
25	Female	155	53	99	66	No
52	Female	145	48	105	80	No
41	Female	146	43	124	80	Yes
44	Female	153	51	112	74	Yes
65	Male	165	88	140	82	Not Known
45	Female	177	81	152	78	Not Known
44	Female	165	71	112	72	Not Known
38	Male	178	105	114	90	No
44	Male	155	77	118	79	Yes
43	Female	176	98	132	80	Yes
48	Female	178	99	108	80	Not Known
35	Female	175	98	132	98	Yes
49	Female	145	74	132	81	No
48	Female	150	55	120	66	No
47	Male	145	47	135	95	No
45	Female	148	62	123	77	Yes
74	Female	149	55	101	87	No
41	Male	156	60	141	82	Yes
51	Male	181	63	142	92	No
38	Female	156	65	132	88	Yes
41	Female	145	78	147	66	No
49	Female	156	55	128	89	No
43	Female	176	62	118	78	No
44	Male	176	108	147	88	Not Known
35	Female	165	68	120	95	No
37	Female	159	66	120	80	Yes
39	Female	166	70	114	98	Yes

49	Male	143	67	149	91	Yes
43	Male	149	55	138	68	No
38	Female	158	51	121	90	No
43	Male	152	50	131	70	Yes
45	Female	179	65	138	75	No
40	Male	156	76	120	85	Yes
71	Male	159	62	121	69	No
68	Male	145	42	110	75	Yes
65	Female	148	75	126	81	Not Known
47	Female	154	51	121	80	yes
68	Female	162	76	114	77	Not Known
35	Female	154	62	129	92	No
40	Female	140	62	130	82	Yes
39	Female	165	60	101	78	No
69	Female	162	99	105	82	Yes
50	Female	165	85	138	86	No
47	Female	151	92	152	81	No
45	Female	173	85	145	87	No
39	Female	174	68	100	72	Yes
71	Female	145	68	122	79	Yes
69	Female	158	60	102	83	Not Known
73	Male	179	105	101	88	Not Known
38	Male	180	75	145	66	Yes
69	Male	156	59	109	86	No
73	Female	169	70	120	85	No
46	Female	165	62	125	79	No
70	Female	150	55	142	72	No
39	Male	145	52	97	77	No
65	Female	152	71	128	78	No
72	Male	181	102	120	80	No
34	Male	170	60	97	87	Yes
65	Male	165	77	123	89	Yes
73	Female	172	76	100	90	Not Known
68	Male	171	109	127	76	Yes
68	Female	157	54	109	79	No
40	Male	140	66	152	85	Not Known
35	Male	178	85	118	74	Yes
46	Female	146	65	142	90	No
46	Male	179	99	152	88	Yes
40	Female	147	62	145	88	No
39	Female	151	52	128	99	No
46	Female	165	65	120	87	No
46	Female	145	73	118	78	Yes
77	Female	165	65	142	86	Not Known
25	Male	177	75	92	65	No
54	Male	174	59	99	88	Yes
59	Male	168	82	98	94	No

47	Male	169	85	95	88	Yes
77	Male	165	67	99	88	Yes
45	Female	176	76	94	82	Yes
58	Male	178	68	102	71	No
46	Male	165	69	108	92	Yes
67	Male	153	64	110	84	Yes
69	Female	153	92	121	81	Yes
49	Female	155	90	99	88	No
58	Female	156	89	98	94	Yes
66	Female	157	88	92	75	Yes
52	Female	156	79	91	73	Yes
57	Male	157	94	120	81	Yes
60	Male	158	79	123	88	No
41	Male	159	81	101	78	Yes
46	Female	161	82	104	81	Yes
58	Female	165	76	115	85	Yes
43	Female	166	78	112	71	No
71	Female	176	75	102	92	Yes
69	Female	171	73	105	75	Yes
56	Female	172	83	108	89	Yes
47	Female	143	84	107	92	No
41	Male	147	82	106	66	Yes
51	Male	149	76	109	77	Yes
47	Female	156	79	111	90	Yes
47	Male	155	78	100	90	Yes
54	Female	154	86	105	84	Yes
67	Female	152	89	120	88	No
56	Male	151	67	132	82	No
57	Female	153	66	125	66	Yes
43	Male	158	63	124	89	Yes
59	Male	179	58	128	78	Yes
55	Female	181	56	129	82	No
55	Male	181	67	124	92	Yes
52	Male	174	58	126	79	Yes
53	Male	176	61	121	80	Yes
58	Male	167	63	122	86	Yes
29	Male	178	67	132	85	Yes
71	Female	179	78	130	71	No
59	Female	174	79	131	90	No
41	Male	167	92	123	95	Yes
41	Male	168	54	125	65	Yes
46	Male	156	55	128	80	Yes
68	Male	159	59	124	85	No
71	Female	180	61	129	85	Yes
62	Female	174	61	126	75	Yes
46	Female	173	89	110	76	Yes
70	Male	156	89	100	88	Yes

41	Male	154	90	102	77	Yes
40	Female	154	60	103	68	No
62	Female	157	66	105	69	No
35	Male	159	64	108	81	Yes
44	Male	140	67	107	83	Yes
60	Male	152	61	106	88	Yes
58	Male	155	62	104	95	No
59	Male	145	90	109	92	Yes
54	Male	146	62	103	80	Yes
44	Male	165	92	102	78	Yes
45	Male	148	75	108	89	Yes
47	Male	153	72	120	81	Yes
41	Female	154	70	111	95	No
44	Female	165	67	102	71	No
57	Male	181	66	121	99	Yes
66	Male	179	79	122	80	Yes
51	Female	179	89	135	78	Yes
54	Male	143	90	145	95	No
56	Female	165	93	152	88	Yes
57	Male	167	94	135	94	Yes
54	Male	156	87	137	72	Yes
40	Male	155	49	138	94	Yes
67	Male	176	58	129	87	Yes
43	Male	178	84	90	95	No
41	Male	179	88	99	87	No
58	Female	145	94	98	80	Yes
58	Male	146	91	97	84	Yes
55	Female	165	68	90	71	Yes
34	Male	155	88	95	95	No
51	Female	152	69	96	78	Yes
57	Female	150	81	99	75	Yes
67	Male	151	83	97	77	Yes
47	Female	145	85	93	99	Yes
58	Female	149	76	92	88	Yes
52	Female	145	71	100	85	No
50	Female	148	72	110	95	No
44	Male	152	77	102	71	Yes
54	Male	157	66	104	88	Yes
44	Male	156	54	106	81	Yes
62	Male	178	58	120	75	No
57	Male	181	56	121	78	Yes
54	Female	179	67	135	68	Yes
65	Female	181	65	142	81	Yes
45	Female	168	68	152	90	Yes
56	Male	173	69	150	80	Yes
46	Male	145	73	142	69	No
54	Female	156	77	124	88	No

63	Female	176	79	132	68	Yes
64	Female	181	61	134	73	Yes
76	Female	179	55	129	85	Yes
40	Female	174	54	127	88	No
59	Male	174	50	125	97	Yes
63	Female	172	51	102	78	Yes
55	Female	165	55	105	85	Yes
71	Female	176	52	123	66	No
52	Male	178	51	149	87	No
56	Male	165	65	138	75	Yes
54	Female	153	67	121	78	Yes
59	Female	153	92	131	88	Yes
46	Female	155	88	138	80	No
55	Female	156	82	120	91	Yes
56	Male	157	81	121	89	Yes
61	Female	156	81	110	78	Yes
40	Male	157	84	126	82	No
59	Male	158	67	121	81	No
60	Male	159	78	114	88	Yes
58	Male	161	77	129	75	Yes
59	Female	165	75	130	95	Yes
44	Female	166	75	101	74	No
53	Female	176	67	105	92	Yes
57	Male	171	65	138	83	Yes
58	Female	172	80	152	86	Yes
54	Male	143	81	145	95	No
62	Male	147	82	100	92	No
57	Male	149	69	122	80	Yes
67	Male	156	65	102	78	Yes
57	Male	155	62	101	89	Yes
52	Female	154	61	145	81	No
38	Female	152	60	109	88	Yes
52	Male	151	65	120	95	Yes
44	Male	153	61	125	92	Yes
50	Male	158	62	142	80	No
62	Male	179	59	97	78	No
58	Female	181	58	128	89	Yes
61	Male	181	81	120	81	Yes
63	Male	175	71	97	95	Yes
65	Female	176	72	123	71	No
64	Male	167	93	100	99	Yes
44	Female	178	96	127	80	Yes
57	Female	179	87	109	78	Yes
52	Female	174	88	152	87	No
53	Female	167	83	118	88	No
67	Male	168	45	142	94	Yes
58	Male	156	56	99	72	Yes

41	Male	159	67	98	94	No
50	Male	180	71	102	87	Yes
41	Male	174	71	101	95	No
29	Female	173	75	100	87	No
61	Male	156	67	115	80	No
45	Female	154	87	114	84	No
59	Male	154	69	120	71	No
63	Female	157	67	123	95	Yes
58	Female	159	59	132	78	Yes
65	Female	140	81	111	75	Yes
66	Female	152	65	120	77	Yes
45	Female	155	68	151	99	No
64	Female	145	61	142	82	Yes
62	Female	146	60	123	85	Yes
35	Male	165	59	132	95	No
53	Male	148	54	128	90	Yes
59	Female	153	51	99	90	No
35	Male	154	55	135	84	No
64	Male	165	51	124	88	Yes
63	Male	181	56	120	82	Yes
49	Male	179	68	125	66	Yes
62	Male	179	78	132	89	Yes
45	Male	145	76	125	78	Yes
35	Female	165	77	128	82	Yes
53	Female	167	65	99	92	Yes
42	Female	156	67	98	79	No
62	Female	155	71	99	80	Yes
55	Male	176	72	99	86	Yes
61	Male	178	77	102	80	No
44	Male	179	79	108	80	Yes
59	Male	145	75	132	88	Yes
49	Male	146	56	142	90	Yes
68	Male	165	56	145	92	No
65	Female	155	74	152	80	Yes
59	Male	152	67	132	95	Yes
54	Female	150	61	120	90	No
57	Male	151	65	122	85	Yes
61	Male	145	76	118	88	Yes
53	Female	149	49	105	89	Yes
29	Male	145	49	120	75	No
56	Female	148	55	123	77	Yes
52	Male	152	51	126	90	Yes
70	Male	157	58	127	88	No
47	Male	156	58	128	89	Yes
53	Male	178	56	120	92	Yes
66	Male	177	71	123	82	Yes
46	Male	179	77	124	87	No

56	Male	181	76	125	81	Yes
57	Male	169	69	124	82	Yes
62	Male	176	66	120	95	No
63	Female	145	72	90	98	Yes
62	Female	156	73	93	97	Yes
42	Male	176	70	92	95	Yes
52	Male	181	72	94	98	No
45	Male	179	56	99	91	Yes
44	Male	174	56	100	80	Yes
52	Male	177	51	124	78	No
58	Male	170	65	125	87	Yes
43	Female	165	65	95	90	Yes
53	Male	176	62	121	95	Yes
52	Male	178	69	110	92	No
67	Male	165	70	92	97	Yes
57	Male	153	71	111	90	Yes
54	Female	153	81	108	97	No
49	Male	155	83	102	93	Yes
45	Male	156	83	101	91	Yes
62	Female	157	84	101	95	Yes
60	Male	156	73	142	80	No
41	Male	157	78	121	85	Yes
70	Male	158	77	148	88	Yes
60	Male	159	71	145	86	No
42	Male	161	77	101	87	Yes
54	Male	165	75	108	89	Yes
53	Female	166	73	132	71	Yes
58	Male	176	69	112	99	No
29	Male	171	66	120	80	Yes
62	Male	172	86	102	78	Yes
66	Female	143	81	121	95	No
54	Female	147	83	120	88	Yes
63	Female	149	69	119	94	Yes
57	Male	156	76	123	72	Yes
62	Female	155	71	98	94	No
53	Female	154	71	142	87	Yes
61	Female	152	67	130	95	Yes
62	Male	151	68	132	87	No
69	Female	153	66	96	80	Yes
55	Female	158	67	120	84	Yes
60	Female	179	60	115	71	Yes
67	Female	177	66	135	95	No
49	Female	181	67	98	78	Yes
58	Female	168	64	121	75	Yes
54	Female	176	72	131	77	No
58	Male	167	73	101	99	Yes
58	Female	178	59	135	88	Yes

37	Female	148	67	111	98	Yes
64	Male	153	60	109	95	Yes
63	Female	154	67	102	90	Yes
53	Female	165	80	101	85	No
66	Female	177	81	109	88	Yes
69	Male	179	83	108	89	No
66	Female	179	60	121	75	Yes
41	Male	148	61	118	77	Yes
59	Female	165	65	123	90	Yes
59	Female	167	59	132	88	Yes
56	Female	156	57	134	89	No
55	Male	155	59	151	92	Yes
52	Female	176	55	120	82	Yes
45	Female	178	50	134	87	No
67	Female	179	53	111	81	Yes
45	Male	145	54	108	82	Yes
51	Male	146	59	135	95	No
64	Female	165	76	110	98	Yes
35	Male	155	71	112	97	No
53	Male	152	68	110	95	Yes
41	Male	150	79	95	98	Yes
61	Male	151	85	142	91	Yes
54	Female	145	88	98	80	Yes
66	Male	149	87	132	78	No
62	Male	145	84	121	87	Yes
61	Male	148	82	102	90	Yes
54	Male	152	65	107	95	No
58	Male	157	68	101	92	Yes
54	Male	156	76	101	97	Yes
63	Male	178	87	120	90	No
55	Female	177	90	125	97	Yes
48	Male	179	79	110	93	No
51	Male	181	78	134	91	Yes
44	Male	175	79	148	87	Yes
43	Female	169	80	110	95	Yes
67	Male	145	65	95	87	Yes
58	Male	156	87	142	80	No
62	Male	176	90	98	84	Yes
57	Male	181	83	132	71	Yes
62	Female	169	76	121	95	No
55	Female	174	80	102	78	Yes
43	Male	178	76	107	75	Yes
34	Female	179	68	101	77	No
61	Male	165	64	101	99	Yes
51	Female	176	67	120	88	No
42	Male	178	63	125	85	Yes
57	Female	165	59	110	95	Yes

43	Female	153	61	134	71	Yes
62	Female	153	76	148	81	Yes
59	Female	155	72	141	81	No
62	Male	156	89	129	75	Yes
52	Male	157	90	130	78	Yes
58	Male	156	88	145	68	No
40	Male	157	68	134	81	Yes
63	Female	158	76	134	90	Yes
60	Female	159	59	128	80	No
61	Male	161	69	132	69	Yes
45	Male	165	68	120	88	No
62	Male	178	82	114	85	No
57	Female	179	76	114	90	Yes
43	Female	174	79	101	81	Yes
59	Male	167	78	145	78	Yes
57	Male	180	61	135	99	Yes
53	Female	156	70	110	88	No
45	Male	159	67	104	75	No
41	Female	180	66	119	91	Yes
41	Male	174	63	152	85	No
57	Male	173	58	117	74	Yes
37	Female	156	56	145	75	No
41	Male	154	67	138	88	Yes
59	Female	154	58	101	91	Yes
50	Female	157	61	117	80	Yes
65	Female	159	63	123	78	Yes
47	Female	140	67	135	81	Yes
46	Male	172	85	101	80	Yes
73	Male	176	70	107	69	Yes
69	Female	171	87	123	91	Yes
25	Female	174	70	91	78	No
73	Female	164	100	118	90	No
75	Female	148	55	132	88	Yes
35	Female	159	78	109	81	No
72	Female	178	75	142	65	Yes
40	Female	154	50	142	68	Yes
40	Male	155	56	104	88	No
81	Female	146	68	105	88	Yes
76	Female	162	78	98	82	No
40	Female	174	60	121	71	Yes
40	Male	172	61	145	92	No
50	Female	148	42	148	78	No
75	Female	176	80	118	81	Yes
38	Female	152	45	152	88	Yes
35	Female	154	60	101	94	No
50	Female	158	78	132	75	Yes
50	Male	180	80	152	73	No

25	Female	159	68	92	81	Not Known
35	Male	165	88	120	88	No
35	Female	165	66	154	78	Yes
26	Male	145	49	121	81	Yes
72	Male	179	70	99	85	Not Known
72	Female	152	77	95	71	No
26	Female	154	75	111	92	No
69	Male	153	77	108	75	Yes
75	Male	159	58	121	89	No
37	Male	148	45	102	92	No
69	Female	160	85	152	78	No
35	Male	155	54	121	77	Yes
26	Female	159	68	142	90	No
50	Female	166	67	131	90	No
37	Female	156	82	148	77	Not Known
50	Female	157	51	149	88	No
50	Male	146	41	129	82	Not Known
24	Female	143	55	96	61	No
69	Female	162	50	135	89	Yes
35	Male	157	53	120	78	No
50	Female	141	63	140	82	Yes
29	Male	156	59	150	92	No
50	Male	161	80	112	79	Yes
26	Male	176	75	142	80	Yes
50	Female	142	60	135	86	No
75	Female	159	105	114	71	Not Known
50	Male	167	62	131	71	Yes
39	Female	178	68	142	90	Yes
23	Female	168	70	92	66	No
78	Female	156	75	132	65	Yes
74	Female	170	78	138	80	Yes
39	Female	155	75	101	85	No
34	Male	172	65	110	85	No
39	Male	177	60	108	75	Not Known
23	Female	143	50	91	65	Not Known
35	Female	165	68	101	88	Yes
37	Female	180	66	145	77	No
78	Female	148	54	110	68	Not Known
78	Male	178	96	132	69	Not Known
26	Male	172	72	132	77	No
72	Female	149	58	123	78	Yes
67	Male	177	79	129	84	No
37	Female	145	48	118	78	Yes
26	Male	153	60	142	88	Yes
34	Male	174	89	131	95	Yes
34	Male	178	72	95	78	No
79	Male	153	57	131	78	No

27	Male	172	103	142	78	Yes
38	Female	159	47	138	89	Yes
27	Male	178	105	105	88	No
27	Male	154	49	102	66	No
82	Female	152	55	128	80	No
23	Male	176	85	93	72	Yes
38	Male	171	100	122	98	No
31	Male	174	72	104	80	No
34	Female	152	48	120	90	No
37	Female	176	62	108	88	No
37	Male	176	95	144	67	No
80	Female	155	52	105	98	Not Known
38	Female	168	71	124	80	Not Known
37	Male	156	62	112	71	Yes
80	Male	172	80	140	68	Not Known
26	Female	162	83	152	95	Yes
26	Female	160	55	112	95	No
28	Male	164	87	114	78	Yes
34	Female	155	59	118	70	Not Known
28	Female	162	84	132	66	No
34	Female	168	56	108	84	No
80	Female	159	81	132	81	Yes
34	Female	165	87	132	71	Yes
22	Female	143	52	93	64	No
80	Female	145	75	135	92	No
80	Male	167	62	123	80	Not Known
23	Female	175	88	97	64	Yes
24	Female	153	74	91	77	Not Known
24	Male	178	88	99	76	No
34	Female	153	56	132	86	Yes
34	Female	155	53	147	69	No
81	Female	140	45	150	88	Yes
34	Female	154	58	118	90	No
24	Female	146	62	90	90	No
23	Female	152	75	97	99	Yes
34	Female	156	62	120	82	No
82	Female	174	60	114	90	Not Known
82	Male	172	65	149	82	Not Known
24	Male	168	60	92	80	Yes
24	Male	178	80	93	98	Not Known
81	Female	140	65	131	90	Not Known
81	Female	150	55	138	98	Not Known
81	Female	150	59	120	80	No
81	Female	158	76	121	95	Yes
76	Female	178	76	110	99	Yes
24	Female	172	97	90	99	No
82	Male	171	70	121	80	Yes

28	Male	166	70	114	83	Not Known
29	Male	173	90	129	78	No
29	Male	175	67	130	86	Yes
30	Male	172	87	101	88	Yes
30	Male	166	90	105	88	No
30	Male	168	68	154	88	No
29	Female	152	51	152	70	Yes
25	Male	174	86	94	74	Yes
25	Female	166	70	91	95	No
25	Male	158	62	92	82	Yes
25	Female	172	71	93	84	No
25	Male	178	98	91	95	Yes
25	Female	168	71	91	100	No
25	Male	176	90	92	80	No
25	Female	167	65	100	80	No
25	Male	165	64	93	83	No
25	Male	149	72	95	90	Yes
25	Female	158	70	97	78	Not Known
25	Female	157	61	94	87	No
25	Female	159	78	92	92	Yes
25	Female	155	62	97	82	No
25	Female	158	75	123	80	No
27	Female	170	68	100	80	No
27	Female	176	88	127	98	Not Known
27	Female	153	52	109	81	No
25	Female	157	71	152	66	Yes
28	Female	165	69	118	95	Yes
29	Female	154	72	142	77	Not Known
29	Female	152	75	152	87	No
29	Female	158	60	145	82	No
29	Female	152	58	128	92	Yes
30	Female	153	52	120	88	No
30	Female	154	58	118	66	Not Known
30	Female	158	55	142	89	Yes
25	Female	148	53	92	78	Yes
32	Male	170	75	118	88	Not Known
32	Male	176	101	106	95	No
32	Male	181	100	140	80	Yes
33	Male	175	103	125	98	No
33	Female	165	72	114	91	Yes
33	Male	180	85	118	68	No
33	Male	152	55	110	90	No
36	Male	152	65	120	70	No
36	Female	158	75	128	75	Yes
36	Male	180	105	128	85	Not Known
33	Male	180	102	99	69	Yes
36	Female	174	98	130	75	No

79	Female	179	90	142	81	Not Known
79	Female	165	98	145	78	Not Known
75	Female	180	64	109	77	Not Known
23	Female	138	65	90	98	No
23	Male	179	100	94	67	Not Known
23	Female	148	62	95	71	No
34	Female	171	94	122	82	Yes
36	Female	181	103	152	79	No
43	Female	175	90	120	94	No
43	Male	156	74	118	92	Yes
44	Male	170	73	152	72	Yes
45	Female	178	98	134	79	No
47	Female	178	101	137	83	No
47	Female	181	91	141	88	No
49	Male	157	94	122	66	No
49	Female	180	101	132	86	No
50	Male	178	94	121	85	No
50	Female	172	60	142	79	No
50	Female	170	94	138	72	No
52	Female	176	97	125	77	Yes
52	Female	175	102	128	78	No
52	Male	173	93	121	80	Yes
52	Female	174	91	104	87	No
54	Female	181	93	132	89	No
55	Female	177	102	132	90	No
55	Female	166	84	128	76	Not Known
55	Female	160	67	108	79	Yes
55	Male	164	88	102	85	No
55	Female	160	71	148	75	No
56	Male	170	94	121	90	No
56	Male	155	81	137	88	No
56	Female	172	103	142	90	No
57	Female	170	65	99	99	No
57	Female	169	86	145	71	Yes
57	Male	175	58	129	78	No
57	Female	177	80	142	90	Yes
57	Female	167	90	129	65	No
58	Female	178	95	132	85	No
58	Female	178	91	123	81	No
58	Female	165	77	145	73	Yes
59	Female	172	91	152	74	Yes
59	Male	158	49	108	88	No
59	Female	158	66	90	80	Yes
59	Female	180	105	99	66	No
59	Male	168	88	114	69	No
59	Male	161	70	117	92	No
60	Male	164	67	120	77	No

60	Male	165	69	120	95	Yes
60	Male	160	101	113	67	No
60	Female	167	72	118	88	Yes
60	Male	178	104	120	68	No
61	Male	145	85	120	71	No
61	Male	165	79	109	73	No
61	Male	167	87	118	91	Yes
61	Female	178	73	112	75	Yes
61	Male	167	98	116	79	No
62	Male	174	83	120	80	No
62	Female	152	59	110	88	No
62	Male	156	73	101	90	No
62	Male	178	89	123	92	No
62	Male	156	48	129	98	Yes
62	Male	161	81	130	95	No
62	Male	178	79	131	90	No
62	Male	162	70	142	85	No
62	Female	169	119	145	88	No
62	Female	179	92	152	71	Yes
63	Female	181	98	110	75	Yes
63	Male	154	92	101	77	Yes
63	Male	160	86	121	90	No
63	Male	161	89	125	88	No
63	Female	172	76	128	89	Yes
63	Female	179	86	129	92	No
63	Male	165	126	129	82	No
63	Male	162	65	132	87	Yes
63	Female	160	52	145	81	No
63	Female	155	61	132	82	Yes
63	Female	180	86	133	95	No
63	Female	170	79	138	69	Yes
63	Female	180	100	136	97	No
63	Female	180	92	139	95	Yes
63	Female	170	60	152	73	No
64	Female	161	97	102	91	Yes
64	Male	163	73	101	80	No
64	Female	164	60	121	78	No
64	Male	157	64	120	87	Yes
64	Female	175	78	130	90	Not Known
64	Male	157	97	131	95	No
64	Female	170	89	133	92	Not Known
64	Male	163	81	137	97	No
64	Female	177	102	129	90	Not Known
64	Female	160	73	132	97	Yes
64	Female	179	116	144	90	Not Known
64	Male	176	84	142	91	No
64	Female	172	107	139	90	Yes

65	Male	153	83	101	80	Yes
65	Male	178	80	110	85	No
65	Male	157	99	112	88	Yes
65	Male	167	77	124	86	No
65	Female	172	98	120	87	No
65	Female	179	100	118	89	No
65	Male	161	78	135	92	Yes
65	Male	164	79	138	95	No
65	Female	174	88	140	97	No
65	Female	181	78	141	88	Yes
66	Female	175	64	99	68	No
66	Female	178	85	95	77	No
66	Male	175	99	105	95	Not Known
66	Female	180	94	130	76	Not Known
66	Female	179	86	132	79	No
66	Male	170	79	134	73	No
66	Female	181	78	128	71	Yes
66	Male	167	80	129	75	Not Known
66	Female	171	86	130	82	No
66	Male	167	68	145	80	Not Known
67	Male	155	66	118	85	No
67	Female	170	80	117	75	No
67	Male	158	59	116	74	Yes
67	Male	161	78	109	73	No
67	Female	177	121	132	71	No
67	Male	151	74	135	90	Yes
67	Female	172	75	139	90	Yes
67	Female	177	97	129	92	No
68	Female	160	97	101	68	No
68	Male	157	77	102	75	No
68	Female	165	65	110	71	No
68	Female	176	97	108	72	Yes
68	Female	174	78	105	79	Not Known
68	Female	167	82	120	77	Yes
68	Male	167	58	121	85	No
68	Male	160	75	102	88	No
68	Male	157	62	101	95	Not Known
68	Male	158	87	101	92	Not Known
68	Male	162	74	142	91	No
68	Female	177	78	121	97	No
68	Female	165	77	148	71	Not Known
68	Female	179	95	145	75	Yes
68	Female	180	105	101	97	Not Known
59	Male	176	66	148	71	Yes
54	Male	178	86	145	99	Yes
65	Female	179	81	109	80	Yes
43	Female	145	83	152	78	Yes

41	Female	146	69	108	95	Yes
42	Female	165	80	128	88	No
59	Male	155	90	121	94	Yes
64	Male	152	89	111	72	Yes
58	Male	150	88	105	94	No
70	Male	151	63	122	87	Yes
51	Female	145	94	149	78	Yes
59	Male	149	79	110	87	Yes
37	Female	145	81	135	80	Yes
54	Male	148	82	134	84	Yes
57	Female	152	76	145	71	Yes
64	Female	157	78	147	81	Yes
47	Male	156	65	145	78	Yes
44	Male	178	73	145	75	No
48	Male	177	83	152	77	Yes
60	Female	178	84	121	91	Yes
51	Male	178	82	111	88	No
42	Male	177	76	109	85	Yes
65	Male	177	79	102	95	No
35	Male	145	78	101	71	Yes
41	Female	156	69	109	88	Yes
41	Male	176	69	108	81	Yes
68	Female	178	67	121	75	Yes
62	Male	169	66	118	78	Yes
58	Male	174	63	123	68	Yes
53	Male	165	58	132	81	No
44	Male	168	56	134	90	Yes
58	Female	165	67	151	80	Yes
62	Male	176	58	120	69	No
70	Male	178	61	134	88	Yes
65	Female	165	63	111	68	No
34	Female	153	67	108	73	Yes
43	Male	153	78	135	85	Yes
49	Male	155	79	110	88	Yes
59	Female	156	92	112	97	Yes
60	Male	157	54	110	78	Yes
58	Female	156	55	95	85	Yes
43	Female	157	59	142	66	No
55	Female	158	61	98	87	Yes
46	Male	159	61	132	75	Yes
58	Female	161	89	121	78	No
34	Male	165	89	102	88	Yes
44	Male	166	90	107	80	No
59	Female	176	60	101	91	Yes
67	Male	171	66	101	89	Yes
54	Female	172	64	120	78	Yes
41	Female	143	67	125	82	Yes

59	Male	147	61	110	81	Yes
54	Male	149	62	134	88	Yes
59	Male	156	70	148	75	No
59	Male	155	62	141	95	Yes
57	Male	154	71	129	74	Yes
60	Male	152	75	130	92	No
60	Female	151	72	145	83	Yes
48	Male	153	70	134	88	No
53	Male	158	67	134	95	Yes
59	Male	169	66	128	92	Yes
48	Male	177	79	132	80	Yes
60	Male	178	89	120	78	Yes
70	Female	177	90	120	89	Yes
70	Female	176	93	101	81	Yes
52	Female	167	94	142	84	No
62	Female	178	87	120	71	Yes
44	Male	179	49	140	99	Yes
58	Female	174	58	120	80	No
63	Female	167	84	104	78	Yes
42	Female	168	88	142	87	Yes
54	Female	156	94	121	88	Yes
34	Female	159	91	99	94	Yes
52	Female	180	68	118	88	Yes
44	Female	174	88	120	88	Yes
62	Female	173	69	148	82	Yes
47	Male	156	81	119	71	Yes
57	Female	154	83	101	92	No
41	Female	154	85	139	84	Yes
56	Female	157	76	118	81	Yes
41	Female	159	71	105	88	No
50	Female	140	72	121	94	Yes
38	Female	152	77	149	75	No
49	Male	152	82	142	79	No
43	Male	150	81	142	80	Yes
46	Female	151	81	104	86	Yes
41	Male	145	84	105	86	Yes
57	Male	149	67	98	71	Yes
66	Male	145	78	121	90	Yes
55	Male	148	76	145	95	Yes
43	Male	152	75	134	65	No
54	Male	157	75	118	80	Yes
53	Female	156	67	152	85	Yes
57	Female	178	65	101	85	No
35	Female	166	80	132	75	Yes
57	Female	178	81	152	76	No
42	Male	178	82	100	88	Yes
35	Male	173	69	120	77	Yes

56	Female	177	65	151	68	Yes
39	Male	145	62	121	69	Yes
35	Male	156	61	99	81	Yes
45	Male	176	60	95	83	Yes
57	Male	178	65	111	88	No
43	Female	169	61	108	95	Yes
47	Female	174	62	121	92	Yes
52	Female	178	59	102	80	No
58	Male	172	58	152	78	Yes
51	Male	165	81	121	89	No
39	Male	176	71	142	81	Yes
29	Male	178	72	131	95	Yes
67	Female	165	78	148	71	Yes
68	Male	153	79	130	85	Yes
44	Male	153	87	129	80	Yes
57	Male	155	88	108	78	Yes
63	Male	156	83	135	95	No
60	Male	157	45	120	88	Yes
56	Female	156	56	140	84	Yes
62	Female	157	67	150	72	No
60	Female	158	71	112	94	Yes
45	Female	159	71	142	87	No
52	Male	161	75	138	95	Yes
59	Male	165	67	114	87	Yes
44	Male	166	87	131	80	Yes
58	Male	176	69	142	84	Yes
60	Male	171	67	151	71	Yes
57	Female	172	59	132	95	Yes
56	Male	143	81	138	78	No
70	Male	147	65	101	75	Yes
57	Male	149	68	110	77	Yes
59	Male	156	61	108	99	No
59	Male	155	60	104	88	Yes
51	Male	154	59	101	85	No
52	Male	152	54	145	95	Yes
51	Female	151	51	110	71	Yes
57	Female	153	55	132	88	Yes
46	Male	158	51	132	81	Yes
38	Male	169	56	123	75	Yes
43	Female	166	78	129	78	Yes
70	Female	178	66	118	68	No
44	Female	177	76	142	81	Yes
68	Female	176	77	131	90	Yes
54	Male	167	65	95	80	No
61	Male	178	67	131	69	Yes
60	Male	179	71	142	88	No
41	Male	174	72	138	68	Yes

41	Male	167	77	105	73	Yes
62	Female	168	79	102	85	Yes
70	Female	156	75	128	88	Yes
41	Female	159	56	130	97	Yes
49	Female	180	56	122	78	Yes
62	Female	174	74	104	85	No
44	Male	173	67	120	66	Yes
44	Male	156	61	108	87	Yes
51	Female	154	65	144	75	No
59	Male	154	76	105	78	Yes
61	Male	157	49	124	88	No
58	Male	159	49	112	80	Yes
41	Female	140	55	140	81	Yes
68	Male	152	51	152	89	Yes
60	Male	155	58	112	78	Yes
44	Male	145	58	114	82	Yes
57	Male	146	56	118	81	Yes
49	Female	165	71	132	88	No
64	Male	148	77	108	75	Yes
61	Male	153	76	132	95	Yes
52	Female	154	69	145	74	No
60	Female	165	66	149	75	Yes
66	Male	166	72	99	83	No
47	Female	178	73	90	88	Yes
61	Female	179	68	111	95	Yes
54	Female	144	69	134	92	Yes
41	Male	165	56	104	80	Yes
64	Male	167	56	149	78	Yes
43	Female	156	51	121	89	Yes
42	Female	155	65	107	81	No
51	Male	176	65	120	88	Yes
54	Male	178	62	118	95	Yes
47	Male	179	69	142	92	No
48	Male	145	70	121	80	Yes
62	Male	146	71	140	78	No
42	Male	165	81	132	89	Yes
58	Male	155	83	120	81	Yes
56	Male	152	83	135	95	Yes
58	Male	150	84	123	71	Yes
53	Male	151	84	101	88	Yes
68	Male	145	78	141	80	Yes
42	Male	149	77	133	78	No
59	Male	145	71	132	95	Yes
41	Male	148	77	147	88	Yes
41	Male	152	70	137	94	No
35	Male	157	73	118	72	Yes
56	Male	156	69	147	94	No

47	Female	178	66	120	87	Yes
40	Male	166	86	120	95	Yes
54	Female	178	81	114	87	Yes
59	Male	178	83	149	80	Yes
60	Female	176	69	138	84	Yes
48	Male	177	70	121	71	Yes
54	Female	145	71	131	95	Yes
58	Female	156	71	138	78	Yes
58	Female	176	67	120	75	No
42	Male	179	68	121	77	Yes
68	Male	169	66	110	72	Yes
65	Male	174	67	126	88	Yes
54	Female	171	60	121	85	No
43	Female	169	66	114	95	Yes
62	Female	165	67	129	90	Yes
56	Male	176	64	130	90	Yes
43	Female	178	72	101	84	No
64	Male	165	73	105	88	Yes
62	Male	153	59	136	82	Yes
50	Male	153	81	152	66	Yes
50	Male	155	82	145	89	No
57	Female	156	85	100	78	Yes
42	Female	157	66	122	82	Yes
67	Female	156	72	102	92	Yes
43	Male	157	79	101	79	No
66	Male	158	74	145	80	Yes
44	Male	159	76	109	86	Yes
58	Male	161	87	120	81	Yes
67	Male	165	90	125	80	No
57	Female	166	83	142	88	Yes
56	Male	176	76	97	90	Yes
58	Male	171	80	128	92	Yes
39	Male	172	76	120	98	No
40	Male	143	68	97	95	Yes
68	Male	147	64	123	90	Yes
64	Male	149	67	100	85	Yes
59	Male	156	63	127	88	Yes
58	Female	155	59	109	89	Yes
29	Male	154	61	152	75	Yes
71	Female	152	76	118	77	No
60	Male	151	72	142	90	Yes
50	Male	153	89	99	88	Yes
53	Female	158	90	98	89	No
59	Male	169	88	102	92	No
65	Female	166	68	101	82	No
56	Male	179	76	100	87	Yes
42	Male	177	59	115	81	Yes

62	Male	176	79	114	82	No
50	Male	167	80	120	95	No
45	Female	178	67	123	89	Yes
58	Male	179	76	132	97	Yes
76	Female	174	68	111	95	No
59	Male	167	69	120	80	Yes
62	Female	168	64	151	91	No
53	Male	156	92	142	80	Yes
42	Female	159	90	123	78	Yes
60	Male	180	89	132	87	Yes
64	Female	174	88	128	90	Yes
61	Male	173	79	99	95	No
54	Male	156	94	135	92	Yes
65	Male	154	79	124	97	Yes
55	Male	154	81	120	90	Yes
67	Female	157	82	125	97	Yes
58	Male	159	76	132	93	Yes
46	Female	140	78	125	91	Yes
40	Female	152	75	128	95	Yes
57	Female	155	73	99	80	Yes
46	Male	145	83	98	85	No
62	Female	146	84	139	88	Yes
40	Female	165	82	99	86	Yes
44	Male	148	76	102	87	Yes
42	Female	153	79	108	89	Yes
50	Female	154	78	132	71	Yes
60	Female	165	82	142	78	Yes
69	Male	179	86	110	78	Yes
38	Male	178	62	92	82	Yes
66	Male	177	69	111	92	No
35	Female	145	75	108	79	No
42	Female	156	72	102	80	No
60	Male	176	70	101	86	Yes
59	Male	179	67	101	74	Yes
60	Female	169	66	142	80	No
74	Male	174	79	121	88	No
54	Male	177	89	148	90	Yes
55	Female	166	90	145	80	No
52	Male	165	93	101	98	Yes
42	Male	176	94	108	95	Yes
62	Female	178	87	132	90	No
56	Male	165	49	112	85	Yes
44	Male	153	58	120	88	Yes
59	Male	153	84	102	89	No
59	Male	155	88	121	75	No
47	Male	156	94	120	77	No
56	Male	157	91	119	90	Yes

67	Female	156	68	123	88	Yes
58	Male	157	88	98	89	Yes
44	Female	158	69	142	82	Yes
58	Male	159	81	130	82	Yes
56	Male	161	83	132	87	No
62	Female	165	85	96	81	Yes
67	Male	166	76	120	82	Yes
58	Female	176	71	115	95	Yes
59	Male	171	72	135	98	No
54	Female	172	77	98	97	Yes
54	Female	143	66	121	95	No
58	Female	147	54	131	85	Yes
52	Female	149	58	101	91	No
60	Female	156	56	135	80	No
57	Female	155	67	148	78	No
49	Female	154	65	145	87	Yes
42	Female	152	68	109	90	No
52	Male	151	69	152	95	No
41	Male	153	73	108	92	No
67	Female	158	77	128	97	Yes
48	Male	177	79	121	90	No
47	Female	166	61	111	97	Yes
58	Female	179	55	105	93	Yes
61	Male	177	54	122	91	Yes
60	Female	176	50	149	77	Yes
46	Male	167	51	110	95	No
46	Male	178	55	135	87	No
66	Female	179	52	145	81	Yes
68	Male	174	51	145	84	Yes
41	Female	167	65	147	71	No
59	Male	168	67	145	95	Yes
58	Male	156	92	145	78	No
57	Male	159	88	152	75	Yes
55	Male	180	82	121	77	Yes
57	Male	174	81	111	99	Yes
51	Male	173	81	109	88	No
40	Male	156	84	102	85	No
42	Female	154	67	101	95	No
49	Female	154	78	109	71	No
49	Female	157	77	108	88	No
56	Male	159	75	121	81	Yes
58	Female	140	75	118	75	No
69	Male	152	67	123	78	Yes
59	Male	155	65	132	68	Yes
48	Male	145	80	134	81	No
46	Female	146	81	151	90	Yes
41	Male	165	82	120	80	Yes

56	Male	148	69	134	69	Yes
60	Female	153	65	111	88	Yes
65	Female	154	62	108	68	No
49	Male	165	61	135	73	Yes
56	Male	166	60	110	85	Yes
52	Male	177	65	112	88	No
59	Male	179	61	110	97	Yes
61	Female	147	62	95	78	No
52	Male	165	59	142	85	Yes
48	Male	167	58	98	66	No
60	Female	156	81	132	87	Yes
53	Female	155	71	121	75	No
58	Female	176	72	102	78	Yes
52	Female	178	70	107	88	No
59	Female	179	72	101	80	No
41	Female	145	87	101	87	No
58	Female	146	88	120	84	No
64	Female	165	83	125	90	Yes
54	Female	155	45	110	82	Yes
55	Female	152	56	134	74	Yes
68	Female	150	67	148	80	No
59	Female	151	71	110	88	No
67	Female	145	71	95	85	No
54	Male	149	75	142	90	Yes
42	Male	145	67	98	81	Yes
70	Female	148	87	132	78	No
34	Female	152	69	121	90	No
52	Male	157	67	102	88	No
45	Female	156	59	107	75	No
53	Female	178	81	101	91	No
52	Female	166	65	101	85	Yes
44	Female	177	68	120	74	Yes
43	Male	179	61	125	75	No
58	Male	179	60	110	88	Yes
39	Male	177	59	134	91	No
57	Male	145	54	148	80	No
58	Male	156	51	141	78	No
37	Female	176	55	129	81	No
57	Male	179	51	130	80	No
69	Male	177	56	145	69	No
50	Female	174	77	134	91	Yes
53	Male	155	76	134	88	Yes
57	Male	154	76	128	90	Yes
46	Male	165	77	132	88	No
37	Male	176	65	120	81	No
54	Male	178	67	120	65	Yes
60	Male	165	71	101	68	Yes

48	Male	153	72	142	88	No
61	Female	153	77	120	88	Yes
46	Male	155	79	140	82	No
61	Male	156	75	120	71	No
68	Male	157	56	104	92	Yes
47	Male	156	56	142	84	Yes
67	Male	157	74	121	81	Yes
53	Female	158	67	99	88	Yes
70	Male	159	61	118	94	Yes
43	Male	161	65	120	75	No
48	Male	165	76	148	73	No
42	Male	166	49	119	81	No
42	Male	176	49	101	88	No
60	Male	171	55	139	78	Yes
47	Male	172	51	118	81	No
65	Female	143	58	105	85	No
62	Female	147	58	121	71	No
62	Female	149	56	149	92	Yes
62	Female	156	71	114	75	No
74	Female	155	77	114	89	No
63	Male	154	76	101	92	Yes
56	Female	152	69	145	66	No
41	Male	151	66	135	77	Yes
58	Female	153	72	110	90	No
35	Female	158	73	104	90	No
51	Female	177	79	119	84	Yes
64	Female	166	80	152	88	Yes
52	Female	179	56	117	82	Yes
48	Male	177	56	140	66	No
60	Female	176	51	138	89	No
71	Female	177	65	101	78	No
56	Female	152	65	117	82	No
54	Female	179	62	123	92	Yes
52	Male	174	69	135	79	Yes
62	Female	167	70	101	80	No
38	Male	168	71	107	86	No
57	Male	156	81	123	75	Yes
58	Male	153	83	148	71	No
43	Male	180	83	118	90	No
59	Male	174	84	132	95	No
63	Male	173	82	109	65	No
58	Male	156	78	142	80	Yes
57	Male	154	77	142	85	Yes
55	Male	154	71	104	85	Yes
35	Male	157	77	105	75	Yes
42	Male	159	83	98	76	Yes
56	Male	160	73	121	88	No

63	Male	162	69	145	77	No
61	Male	165	66	149	68	Yes
51	Male	168	86	118	69	No
44	Male	167	81	152	74	Yes
51	Male	169	83	101	78	Yes
39	Male	140	69	132	84	No
62	Male	152	85	152	78	Yes
61	Male	155	71	100	88	Yes
58	Male	145	71	120	95	Yes
50	Female	155	60	112	76	Yes
66	Female	152	61	142	66	Yes
67	Male	150	57	147	84	Yes
48	Female	151	57	114	81	No
58	Male	145	58	131	71	Yes
54	Male	149	70	142	84	No
44	Male	145	75	151	92	No
65	Female	148	79	132	80	Yes
66	Female	152	83	138	88	No
67	Female	157	52	101	92	No
46	Female	156	65	110	76	Yes
49	Male	178	67	108	86	No
55	Female	166	60	104	69	No
63	Female	177	67	101	93	Yes
40	Female	179	80	145	90	Yes
59	Male	180	81	110	74	No
43	Male	177	83	132	84	No
67	Male	145	60	132	82	No
69	Female	156	61	123	90	No
55	Male	176	65	129	82	Yes
43	Male	179	59	118	95	No
44	Female	177	57	142	89	Yes
52	Male	174	59	131	90	Yes
57	Female	169	55	95	98	No
57	Female	171	50	131	80	No
58	Male	165	53	142	95	No
70	Male	176	54	138	99	No
35	Female	178	59	105	80	Yes
66	Male	165	76	102	80	Yes
58	Male	153	71	128	83	No
56	Female	153	68	142	78	Yes
58	Female	155	79	122	86	Yes
64	Female	156	85	104	88	Yes
67	Female	157	88	120	88	No
64	Male	156	87	108	88	No
56	Male	157	84	144	70	No
59	Male	158	82	105	74	No
65	Male	159	65	124	92	No

56	Male	161	68	112	87	Yes
56	Female	165	76	140	87	Yes
63	Male	166	87	152	66	No
56	Male	176	90	112	66	Yes
58	Female	171	79	114	80	Yes
63	Female	172	78	118	80	No
60	Male	143	79	132	89	No
59	Male	147	77	108	82	Yes
49	Male	149	85	132	78	No
64	Female	156	87	132	80	Yes
61	Female	155	90	120	90	No
47	Male	154	83	125	82	Yes
44	Female	152	76	110	80	Yes
41	Male	151	80	134	80	Yes
64	Female	153	76	148	82	Yes
39	Male	158	68	141	81	No
67	Male	177	64	129	66	No
63	Male	165	78	101	94	Yes
51	Male	168	75	145	92	Yes
64	Male	177	73	135	72	No
57	Male	179	83	110	79	No
52	Male	143	84	104	83	Yes
45	Male	165	82	119	88	Yes
59	Male	167	76	152	66	Yes
46	Female	156	79	117	86	Yes
46	Female	155	78	146	85	No
41	Female	176	82	138	79	No
51	Female	178	83	101	72	No
43	Female	179	67	117	77	Yes
49	Female	145	66	123	78	No
57	Female	146	63	135	80	No
56	Female	165	58	101	87	Yes
64	Female	155	56	107	89	No
42	Male	152	67	123	90	No
41	Male	150	58	148	76	Yes
44	Male	151	61	118	79	No
56	Male	145	63	132	85	No
61	Male	149	67	109	74	No
50	Male	145	78	142	90	Yes
58	Male	148	79	142	88	No
42	Male	152	92	104	95	No
59	Male	157	54	105	99	Yes
43	Male	156	55	98	87	Yes
52	Male	178	59	121	78	No
63	Male	168	61	145	92	Yes
35	Male	177	61	149	65	No
64	Male	179	89	118	88	Yes

50	Male	180	89	152	94	Yes
57	Female	177	90	101	88	Yes
60	Male	145	60	132	88	No
52	Male	156	66	152	82	Yes
41	Male	176	64	100	71	No
46	Male	179	67	120	92	Yes
63	Male	177	61	151	84	Yes
45	Male	174	62	121	81	Yes
44	Female	170	86	99	88	Yes
62	Male	172	62	95	94	Yes
58	Male	165	88	111	75	No
62	Female	176	75	108	73	Yes
68	Female	178	72	121	81	Yes
41	Male	165	70	102	88	Yes
57	Female	153	67	152	78	Yes
64	Female	153	66	121	81	No
57	Female	155	79	142	85	Yes
49	Female	156	89	131	71	No
52	Female	157	90	148	84	Yes
54	Female	156	93	148	75	Yes
41	Female	157	94	129	89	Yes
62	Female	158	87	108	92	Yes
59	Male	159	49	135	66	Yes
58	Male	161	58	120	77	No
58	Female	165	84	140	80	Yes
50	Female	166	88	150	74	Yes
62	Female	176	94	112	84	Yes
45	Male	171	91	142	88	Yes
74	Female	172	68	130	82	No
55	Male	143	88	114	66	Yes
48	Male	147	69	131	89	No
53	Female	149	81	142	78	Yes
60	Female	156	83	151	82	Yes
40	Male	155	85	132	92	Yes
63	Male	154	76	138	79	Yes
52	Male	152	71	101	80	Yes
44	Male	151	72	110	86	No
61	Male	153	77	108	66	Yes
48	Female	158	66	104	71	Yes
66	Male	177	54	101	90	Yes
62	Female	168	58	145	90	Yes
63	Female	179	56	110	65	No
64	Male	177	67	132	80	Yes
64	Male	176	65	132	85	No
41	Male	177	68	123	85	Yes
46	Female	178	69	129	75	Yes
57	Male	179	73	118	76	Yes

48	Male	174	77	142	88	Yes
55	Male	167	79	131	77	Yes
44	Male	168	61	95	68	No
55	Male	156	55	131	69	Yes
48	Male	159	54	142	68	Yes
61	Female	180	50	138	83	Yes
66	Male	174	51	105	88	Yes
41	Male	178	60	139	77	Yes
52	Male	179	65	132	99	No
54	Male	145	61	147	88	Yes
43	Male	146	62	134	85	No
41	Male	165	59	118	95	Yes
63	Male	155	58	147	71	Yes
60	Female	152	81	120	88	Yes
41	Male	150	71	120	81	Yes
59	Male	151	72	114	75	Yes
41	Female	145	78	149	71	No
57	Female	149	79	138	68	Yes
56	Female	145	87	121	81	Yes
57	Male	148	88	131	90	Yes
60	Male	152	83	138	80	Yes
61	Female	157	45	120	69	No
42	Female	156	56	121	88	Yes
54	Male	178	67	110	68	No
49	Male	168	71	126	73	Yes
54	Male	177	71	121	85	Yes
69	Female	177	75	114	88	Yes
54	Male	181	67	129	97	Yes
64	Female	177	87	130	78	Yes
70	Female	145	69	101	85	No
47	Male	156	67	105	66	Yes
57	Female	176	59	136	87	Yes
64	Male	177	81	152	75	Yes
50	Female	177	65	145	78	Yes
40	Female	174	68	100	88	No
57	Female	173	61	122	80	Yes
37	Female	174	60	102	91	No
54	Female	165	59	101	89	Yes
49	Female	176	54	145	78	Yes
53	Female	178	51	109	82	Yes
34	Female	165	55	120	81	Yes
51	Female	153	51	125	88	Yes
55	Male	153	56	142	75	No
41	Male	155	72	97	95	Yes
52	Male	156	72	128	74	Yes
67	Male	157	76	120	92	Yes
65	Male	156	77	97	83	Yes

59	Female	157	65	123	88	No
52	Male	158	67	100	95	Yes
57	Male	159	71	127	92	No
53	Male	161	72	109	80	Yes
62	Male	165	77	152	78	Yes
49	Male	166	79	118	89	Yes
58	Male	176	75	142	81	Yes
63	Female	171	56	152	88	Yes
46	Female	172	56	145	95	No
60	Male	143	74	128	92	Yes
64	Male	147	67	120	80	Yes
54	Male	149	61	118	78	Yes
46	Male	156	65	142	89	Yes
51	Male	155	76	92	81	No
57	Male	154	49	99	95	Yes
58	Female	152	49	98	71	No
57	Male	151	55	95	99	Yes
61	Male	153	51	138	80	Yes
35	Male	158	58	137	78	Yes
50	Male	177	58	130	95	Yes
50	Male	168	56	131	88	Yes
69	Female	177	71	136	94	No
35	Female	177	77	99	72	Yes
53	Male	176	76	99	94	Yes
71	Female	177	69	98	87	Yes
49	Male	178	66	92	95	Yes
39	Male	179	72	91	87	No
48	Male	174	73	120	80	Yes
51	Male	167	70	123	84	No
29	Female	168	60	101	71	Yes
39	Female	156	56	104	95	Yes
52	Female	159	56	115	78	Yes
61	Female	180	51	112	75	Yes
67	Female	174	65	102	77	Yes
67	Female	173	65	105	99	No
49	Female	156	62	108	88	Yes
45	Male	154	69	107	85	Yes
41	Male	154	70	106	95	Yes
59	Male	157	71	109	90	Yes
57	Male	159	81	111	90	No
67	Male	140	83	100	84	Yes
58	Male	152	83	105	88	No
34	Male	155	84	120	82	Yes
57	Female	145	84	132	66	Yes
41	Female	146	78	125	89	Yes
41	Male	165	77	124	78	Yes
56	Female	148	71	128	82	Yes

56	Male	153	77	129	92	No
65	Female	154	70	124	79	Yes
69	Female	165	73	126	80	Yes
53	Female	168	69	121	86	Yes
60	Male	169	66	122	64	Yes
64	Male	179	86	132	80	No
61	Male	149	81	130	88	Yes
58	Male	165	83	131	90	No
60	Male	167	69	123	92	Yes
59	Male	156	65	125	98	Yes
52	Male	155	71	128	95	Yes
42	Male	176	71	124	90	Yes
56	Female	178	67	129	85	Yes
41	Female	179	68	126	88	No
60	Male	145	66	110	89	Yes
67	Male	146	67	100	75	Yes
65	Female	165	60	102	77	Yes
61	Female	155	66	103	90	Yes
55	Male	152	67	105	88	No
62	Female	150	64	108	89	Yes
64	Male	151	72	107	92	No
59	Male	145	73	106	82	Yes
64	Male	149	59	104	87	Yes
58	Male	145	81	109	81	Yes
57	Female	148	82	103	82	Yes
52	Female	152	85	102	95	Yes
45	Male	157	66	108	98	No
44	Female	156	58	120	97	Yes
58	Female	178	51	111	95	Yes
59	Female	168	55	102	98	Yes
50	Male	169	52	121	91	Yes
44	Male	177	51	122	80	No
66	Male	179	65	135	78	Yes
66	Male	177	67	145	87	No
59	Male	145	92	152	90	Yes
45	Female	156	88	135	95	Yes
64	Female	176	82	137	92	Yes
44	Female	177	81	138	97	Yes
55	Male	177	81	129	90	Yes
50	Female	174	84	90	97	No
58	Female	175	67	99	93	Yes
39	Female	176	78	98	91	Yes
45	Male	165	77	97	95	Yes
52	Male	176	75	90	80	Yes
41	Male	178	75	95	85	No
66	Male	165	67	96	88	Yes
69	Male	153	65	99	86	No

40	Male	153	80	97	87	Yes
49	Female	155	81	93	89	Yes
62	Female	156	82	92	71	Yes
60	Male	157	69	100	99	Yes
61	Female	156	65	110	80	Yes
56	Female	157	62	102	78	No
57	Male	158	61	104	95	Yes
68	Male	159	60	106	88	Yes
55	Male	161	65	120	94	Yes
44	Male	165	61	121	72	Yes
29	Male	166	62	135	94	No
63	Male	176	59	142	87	Yes
40	Male	171	58	152	95	Yes
64	Female	172	81	150	87	Yes
58	Female	143	71	142	80	Yes
55	Male	147	72	124	84	Yes
59	Male	149	59	132	71	Yes
59	Male	156	56	134	95	Yes
50	Male	155	87	129	78	No
67	Male	154	88	127	75	Yes
64	Male	152	83	125	77	Yes
61	Male	151	45	102	99	Yes
60	Male	153	56	105	88	Yes
69	Male	158	67	123	85	No
57	Female	177	71	149	76	Yes
64	Male	168	71	138	90	No
54	Female	177	75	121	90	Yes
57	Male	177	67	131	84	Yes
58	Female	176	87	138	88	Yes
55	Male	177	69	120	82	Yes
45	Female	178	67	121	66	Yes
52	Male	179	59	110	89	No
51	Female	174	81	126	78	Yes
67	Female	167	65	121	82	Yes
64	Male	165	56	97	92	Yes
34	Male	168	56	123	82	Yes
46	Female	169	74	100	87	Yes
77	Female	179	67	127	81	No
53	Female	148	61	109	82	Yes
55	Female	165	65	152	80	Yes
58	Female	167	76	118	98	Yes
57	Female	156	49	142	90	Yes
59	Female	155	49	99	95	No
42	Female	176	55	98	98	Yes
47	Female	178	51	102	91	No
56	Male	179	58	101	80	Yes
66	Female	145	58	100	78	Yes

51	Female	146	56	115	87	Yes
57	Male	165	71	114	90	Yes
64	Male	155	77	120	95	Yes
65	Female	152	76	123	92	No
54	Female	150	69	132	97	Yes
53	Female	151	66	111	90	Yes
66	Female	145	72	120	97	Yes
47	Female	149	73	154	88	Yes
52	Male	145	110	142	91	Yes
64	Male	148	82	123	87	Yes
57	Female	152	56	132	95	No
49	Male	157	56	128	87	Yes
61	Female	156	51	99	80	Yes
57	Male	178	65	135	84	Yes
63	Male	168	65	124	71	Yes
40	Male	169	62	120	95	Yes
37	Male	177	69	125	78	No
48	Male	174	70	132	75	Yes
60	Male	177	71	125	77	Yes
66	Male	145	81	128	70	Yes
56	Male	156	83	99	88	Yes
44	Male	176	83	98	85	No
38	Male	177	84	150	95	Yes
51	Male	177	70	99	71	No
64	Male	174	78	102	88	Yes
45	Male	178	77	108	81	Yes
57	Male	177	71	132	75	Yes
58	Male	165	77	142	78	Yes
71	Male	176	68	145	68	Yes
66	Male	178	73	152	81	Yes
61	Male	165	69	108	90	Yes
65	Male	153	66	110	80	No
39	Male	153	86	90	69	Yes
51	Male	155	81	110	88	No
41	Male	156	83	110	68	No
64	Male	157	69	106	73	Yes
45	Male	156	80	110	85	No
58	Male	157	52	114	88	Yes
35	Female	158	55	109	97	No
57	Male	159	56	115	78	Yes
49	Male	161	60	95	85	Yes
51	Male	165	60	105	66	No
60	Male	166	61	119	87	Yes
39	Male	176	57	98	75	No
56	Male	171	57	104	78	Yes
44	Male	172	58	117	88	Yes
57	Female	143	70	120	80	Yes

41	Female	147	75	115	87	Yes
54	Male	149	79	121	84	Yes
44	Male	156	83	120	90	Yes
50	Female	155	52	123	82	No
53	Male	154	65	123	74	Yes
61	Female	152	67	123	80	No
59	Male	151	60	122	88	Yes
58	Male	153	67	124	85	Yes
42	Female	158	80	120	90	No
64	Male	166	81	120	81	Yes
60	Male	168	83	120	78	No
70	Male	177	60	124	99	Yes
41	Male	177	61	123	88	Yes
61	Female	176	65	124	75	Yes
51	Male	177	59	124	91	Yes
58	Male	178	57	125	85	No
63	Male	179	59	125	74	Yes
52	Male	174	55	120	75	Yes
63	Female	167	50	125	88	Yes
42	Male	168	53	126	91	Yes
47	Male	156	54	126	80	Yes
74	Female	159	59	121	78	Yes
60	Female	180	76	127	81	No
56	Female	174	71	120	80	Yes
56	Female	173	68	127	69	No
70	Male	156	79	127	91	Yes
60	Female	154	85	127	88	Yes
52	Male	154	88	128	90	No
41	Male	157	87	128	88	Yes
59	Male	159	84	128	81	No
60	Female	140	82	128	65	Yes
54	Male	152	65	129	68	Yes
58	Male	155	68	129	88	Yes
66	Female	145	76	130	88	Yes
49	Female	146	87	142	82	No
34	Female	165	90	130	71	Yes
41	Female	148	79	130	92	Yes
60	Female	153	78	130	84	Yes
63	Male	154	79	130	81	Yes
47	Male	165	78	133	88	Yes
71	Female	168	74	130	94	Yes
44	Male	169	87	132	75	No
55	Female	179	90	131	73	Yes
59	Male	140	83	132	81	No
64	Male	165	76	131	88	Yes
52	Male	167	80	131	78	Yes
64	Male	156	76	132	81	No

50	Male	155	68	132	85	Yes
56	Male	176	64	132	71	No
41	Male	178	67	133	92	Yes
66	Female	179	63	133	75	Yes
54	Female	145	59	133	89	Yes
56	Male	146	61	133	92	Yes
71	Female	165	76	133	66	No
59	Male	155	72	134	77	Yes
52	Female	152	89	134	90	Yes
54	Female	150	90	134	90	Yes
51	Female	151	88	135	84	Yes
50	Female	145	68	135	88	Yes
49	Male	149	76	135	82	Yes
74	Male	145	59	142	66	No
70	Male	148	75	152	89	Yes
70	Female	152	76	100	78	No
67	Male	157	67	101	82	Yes
57	Male	156	76	105	92	Yes
55	Female	178	68	105	79	No
65	Male	168	69	105	80	Yes
58	Male	169	64	101	86	No
44	Male	180	92	108	77	Yes
62	Male	172	90	104	71	Yes
46	Male	177	89	108	90	Yes
60	Female	145	88	110	95	Yes
55	Male	156	79	90	65	No
65	Female	176	94	110	80	Yes
60	Female	180	79	110	85	Yes
51	Female	166	81	106	85	Yes
51	Female	174	82	110	75	Yes
65	Male	179	76	114	76	Yes
60	Female	156	78	109	88	Yes
44	Male	165	75	115	77	No
45	Female	176	73	95	68	Yes
56	Female	178	83	105	69	No
64	Female	165	84	119	72	Yes
55	Female	153	82	98	78	Yes
39	Female	153	76	104	84	No
52	Female	155	79	117	78	Yes
42	Male	156	78	120	88	No
63	Female	157	77	115	95	Yes
35	Female	156	70	121	78	Yes
35	Male	157	67	120	78	Yes
52	Female	158	66	123	78	Yes
67	Male	159	63	123	89	No
49	Female	161	58	123	88	Yes
45	Female	165	56	122	66	Yes

61	Female	166	67	124	80	Yes
70	Male	176	58	120	80	Yes
42	Female	171	61	120	98	Yes
52	Female	172	63	120	80	Yes
78	Female	143	67	124	90	No
63	Female	147	78	123	88	Yes
39	Female	149	79	124	67	No
42	Female	156	92	124	98	Yes
60	Female	155	54	125	80	Yes
47	Female	154	55	125	71	No
62	Male	152	59	120	68	Yes
54	Female	151	61	125	95	No
61	Female	153	61	126	95	Yes
53	Male	158	89	126	78	Yes
58	Male	166	89	121	74	Yes
58	Male	169	90	127	66	Yes
50	Male	180	60	120	84	No
53	Male	177	66	127	81	Yes
41	Male	176	64	127	71	Yes
45	Male	177	67	127	84	Yes
64	Male	165	61	128	92	Yes
63	Male	179	62	128	80	Yes
67	Female	174	72	128	88	Yes
74	Male	167	62	128	92	No
66	Male	168	68	129	76	Yes
59	Male	156	75	129	86	No
45	Male	168	72	130	69	Yes
51	Male	180	70	142	93	Yes
50	Male	174	67	130	90	No
65	Male	173	66	130	74	Yes
53	Female	156	79	130	84	No
60	Male	154	89	130	82	Yes
54	Male	154	90	133	90	Yes
43	Male	157	93	130	82	Yes
59	Male	159	94	132	95	Yes
77	Female	169	87	131	98	No
45	Female	176	49	132	90	Yes
42	Male	168	58	131	98	Yes
59	Male	176	84	131	80	Yes
70	Female	178	88	132	95	Yes
50	Male	165	94	132	99	Yes
45	Female	153	91	132	80	Yes
62	Male	153	68	133	80	No
62	Female	155	88	133	83	No
46	Male	156	69	133	78	No
62	Female	157	81	133	86	Yes
56	Male	156	83	133	88	Yes

56	Male	157	85	134	88	No
58	Male	158	76	134	88	Yes
77	Female	177	65	142	87	Yes
39	Female	178	67	142	82	Yes
62	Female	179	92	142	90	Yes
62	Male	174	88	144	88	Yes
56	Female	167	82	144	66	Yes
63	Female	168	81	144	89	Yes
54	Female	156	81	145	78	Yes
59	Male	159	84	145	88	Yes
60	Female	180	67	140	90	Yes
59	Male	174	78	152	80	No
69	Male	173	77	147	98	Yes
41	Male	156	75	147	91	No
45	Female	154	75	140	68	Yes
55	Male	154	67	152	90	Yes
51	Female	157	65	152	77	No
64	Female	159	80	145	75	Yes
59	Male	140	81	152	85	No
35	Female	152	82	150	69	Yes
62	Female	155	69	90	75	Yes
43	Male	145	65	99	81	Yes
59	Male	146	62	95	80	Yes
57	Male	165	61	91	77	No
52	Male	148	60	96	92	Yes
50	Male	153	65	97	82	Yes
57	Male	154	61	93	78	Yes
43	Male	165	62	102	82	Yes
44	Male	169	59	104	86	Yes
62	Male	169	58	105	94	Yes
60	Female	179	81	120	92	No
52	Male	144	71	110	72	Yes
52	Male	165	72	111	79	No
52	Male	167	69	110	83	Yes
41	Male	156	56	103	88	Yes
34	Male	155	87	106	66	No
42	Female	176	88	101	86	Yes
51	Female	178	83	105	85	No
42	Female	179	45	109	79	Yes
43	Female	145	56	111	72	Yes
37	Female	146	67	121	77	Yes
63	Female	165	71	122	78	Yes
44	Female	155	71	108	80	No
47	Male	152	75	110	87	Yes
63	Male	150	67	112	89	Yes
38	Male	151	87	148	90	Yes
52	Male	145	69	132	76	Yes

65	Female	149	67	119	79	Yes
52	Male	145	59	130	85	Yes
50	Female	148	81	128	74	No
54	Male	152	65	127	90	Yes
49	Male	157	68	124	88	No
39	Female	156	61	125	95	Yes
58	Female	178	60	120	99	Yes
61	Female	169	59	121	87	No
62	Female	169	54	105	78	Yes
62	Female	180	51	109	92	No
55	Female	176	55	112	65	Yes
41	Female	177	51	128	85	Yes
54	Female	145	56	109	81	Yes
43	Female	156	58	132	95	Yes
42	Male	176	59	130	92	No
47	Female	180	76	138	88	Yes
35	Male	166	77	137	80	Yes
44	Female	174	65	139	66	Yes
44	Female	151	70	129	82	Yes
46	Male	153	56	132	87	No
70	Male	158	56	131	81	Yes
52	Male	166	51	130	82	Yes
70	Male	169	65	138	95	Yes
38	Female	180	65	135	98	Yes
47	Male	177	62	139	97	Yes
62	Female	176	69	139	90	Yes
52	Male	177	70	120	98	Yes
59	Male	178	71	121	91	Yes
59	Male	179	81	125	80	Yes
59	Female	174	83	128	78	Yes
59	Male	167	83	129	87	Yes
47	Male	168	84	131	90	No
41	Male	156	84	127	95	Yes
56	Male	159	78	121	92	No
54	Male	180	77	120	97	Yes
44	Male	174	71	129	90	No
63	Female	173	77	131	97	No
58	Female	156	70	135	93	Yes
67	Male	154	73	134	91	No
43	Male	154	69	145	95	Yes
71	Female	157	66	142	80	Yes
54	Male	159	86	111	85	Yes
44	Male	140	81	110	88	Yes
66	Male	152	83	103	86	No
60	Female	155	69	102	87	Yes
59	Male	145	74	120	89	Yes
64	Male	146	71	125	92	Yes

47	Male	165	71	127	95	Yes
45	Male	148	67	129	97	Yes
61	Male	153	68	129	99	Yes
66	Male	154	66	130	68	No
58	Male	165	67	131	77	Yes
67	Male	169	60	134	95	No
59	Male	169	66	137	76	Yes
58	Male	179	67	138	97	Yes
52	Male	143	64	139	96	Yes
55	Female	165	72	142	97	Yes
62	Female	167	73	143	92	No
62	Female	156	59	143	90	Yes
57	Female	155	81	143	84	Yes
58	Male	176	82	144	85	Yes
56	Female	178	85	146	80	Yes
51	Female	179	66	150	68	No
54	Male	145	75	106	78	Yes
57	Male	146	52	105	90	Yes
35	Male	165	55	108	94	Yes
47	Male	155	56	120	92	Yes
58	Female	152	60	120	93	Yes
56	Male	150	60	121	91	Yes
56	Female	151	61	129	96	No
57	Female	145	57	129	95	Yes
67	Female	149	57	129	94	No
62	Female	145	58	129	98	Yes
42	Male	148	70	130	99	Yes
38	Male	152	75	135	85	No
54	Female	157	79	138	88	Yes
41	Male	156	83	105	94	No
57	Male	178	52	120	95	Yes
46	Female	169	65	127	80	Yes
54	Male	169	67	125	80	Yes
60	Female	180	60	130	80	Yes
52	Female	174	67	131	92	No
58	Female	177	80	129	94	Yes
45	Male	145	81	139	95	Yes
63	Male	156	83	128	92	Yes
57	Female	176	60	145	72	Yes
58	Male	180	61	101	88	Yes
49	Male	166	65	108	84	Yes
69	Male	174	59	115	78	No
58	Male	172	57	120	90	Yes
54	Male	173	59	128	74	No
63	Male	165	55	127	77	Yes
71	Female	176	50	129	75	Yes
67	Male	178	53	130	85	No

59	Male	165	54	138	87	Yes
34	Male	153	59	134	91	No
37	Female	153	76	135	85	Yes
63	Male	155	71	134	94	Yes
57	Male	156	68	135	95	Yes
47	Male	157	79	142	92	Yes
52	Male	156	85	144	92	No
60	Male	157	88	145	88	Yes
52	Male	158	87	143	90	Yes
66	Female	159	84	147	84	Yes
52	Male	161	82	105	90	Yes
64	Male	165	65	101	94	Yes
44	Male	166	68	109	88	Yes
54	Female	176	76	103	75	No
58	Male	171	87	121	68	Yes
54	Female	172	90	123	69	No
70	Male	143	79	128	78	Yes
54	Male	147	78	127	77	Yes
49	Male	149	79	125	71	No
57	Male	156	76	129	72	Yes
49	Female	155	78	132	74	No
42	Female	154	87	142	76	Yes
52	Male	152	90	145	75	Yes
49	Female	151	83	102	95	Yes
58	Male	153	76	110	95	Yes
43	Female	158	80	108	96	No
65	Female	166	76	128	97	Yes
46	Female	169	68	129	68	Yes
62	Male	180	64	135	69	Yes
44	Male	177	67	120	90	Yes
55	Female	176	63	135	92	Yes
54	Male	177	59	123	95	Yes
51	Male	178	61	101	94	No
52	Female	179	76	141	83	Yes
47	Male	174	72	146	91	Yes
61	Male	167	89	132	99	Yes
62	Female	168	90	147	68	Not Known
52	Male	156	88	148	86	No
65	Female	159	68	118	78	Yes
49	Male	180	76	147	91	No
58	Male	174	59	120	93	Yes
44	Male	173	60	120	91	Yes
41	Female	156	65	114	99	Yes
64	Female	154	67	149	81	Yes
46	Female	154	76	138	90	No
54	Female	157	68	121	96	Yes
46	Female	159	69	131	92	Yes

65	Female	140	64	138	80	Yes
58	Female	152	92	120	88	Yes
57	Female	155	90	121	87	Yes
62	Female	145	89	110	85	Yes
70	Female	146	88	126	80	No
60	Female	165	65	121	80	Yes
56	Female	148	68	114	78	No
46	Female	153	61	129	80	Yes
68	Male	154	60	130	78	Yes
57	Male	165	59	101	69	No
59	Female	169	54	105	75	Yes
62	Male	169	51	142	95	No
48	Female	169	55	152	66	Yes
74	Female	142	51	145	74	Yes
59	Male	165	56	100	89	Yes
67	Male	167	69	122	78	Yes
62	Male	156	68	102	68	No
46	Male	155	76	101	99	Yes
44	Male	176	77	145	88	Yes
52	Male	178	65	109	77	Yes
63	Male	179	67	120	80	Yes
57	Female	145	71	125	80	Yes
77	Female	146	72	142	80	Yes
56	Female	165	77	97	92	No
68	Male	155	79	128	94	Yes
61	Male	152	75	120	95	No
51	Male	150	56	97	92	Yes
50	Male	151	56	123	91	Yes
71	Male	145	74	100	88	No
54	Male	149	67	127	84	Yes
61	Male	145	61	109	78	No
52	Male	148	65	152	90	Yes
58	Female	152	76	118	74	Yes
59	Male	157	49	142	77	Yes
56	Male	156	49	152	75	Yes
51	Male	178	55	145	85	No
56	Female	169	51	128	87	Yes
57	Male	169	58	120	69	Yes
45	Male	180	58	118	78	Yes
63	Female	177	56	142	77	Yes
56	Male	177	71	92	71	Yes
44	Male	145	77	99	72	Yes
46	Male	156	76	98	74	No
43	Male	176	69	95	76	Yes
39	Female	180	66	140	75	No
51	Male	166	72	147	95	Yes
67	Male	174	73	99	95	Yes

63	Male	175	70	92	96	Yes
51	Male	174	72	91	97	Yes
52	Female	165	56	95	68	No
66	Female	176	56	99	69	No
49	Male	178	51	98	90	No
47	Male	165	65	92	92	Yes
50	Male	153	65	91	95	No
57	Male	153	62	120	94	Yes
59	Male	155	69	123	93	Yes
51	Male	156	70	101	91	Yes
61	Female	157	71	104	99	No
66	Male	156	81	115	68	Yes
61	Female	157	83	112	86	No
60	Female	158	83	102	78	Yes
49	Male	159	84	105	91	Yes
46	Female	161	75	108	93	Yes
35	Female	165	78	107	91	Yes
58	Female	179	64	124	78	No
52	Female	174	72	129	82	Yes
58	Female	167	73	126	86	No
41	Male	168	59	110	94	Yes
47	Male	156	81	100	92	Yes
61	Female	165	82	102	72	Yes
50	Male	180	85	103	79	Yes
68	Male	174	66	105	83	Yes
53	Female	173	78	108	88	Yes
55	Male	156	52	107	66	Yes
48	Male	154	55	106	86	No
48	Male	154	56	104	85	Yes
37	Male	157	60	109	79	Yes
41	Male	159	60	103	72	Yes
44	Female	176	61	102	77	No
56	Male	168	57	108	78	Yes
59	Male	169	57	120	80	No
54	Male	160	58	111	87	Yes
51	Male	167	70	102	89	Yes
55	Female	169	75	121	99	Yes
35	Female	140	79	122	98	Yes
89	Male	152	83	135	95	Yes
52	Male	155	52	145	96	Yes
58	Male	145	65	152	92	Yes
54	Female	146	67	135	80	No
48	Male	165	60	137	88	Yes
57	Male	148	67	138	87	Yes
56	Male	153	80	129	85	Yes
41	Female	154	81	90	80	No
51	Male	165	83	99	80	Yes

44	Male	169	60	98	78	No
58	Female	168	61	97	80	Yes
63	Female	169	65	90	78	Yes
63	Male	148	59	95	69	Yes
70	Female	165	57	96	75	Yes
54	Female	167	59	99	95	Yes
45	Male	156	55	97	66	Yes
48	Male	177	87	139	97	Yes
53	Male	145	90	128	100	Yes
58	Male	156	83	145	92	Yes
56	Male	176	76	101	100	Yes
68	Male	179	80	108	95	Yes
65	Male	166	76	129	98	Yes
67	Male	174	68	129	100	No
43	Male	154	64	129	88	Yes
35	Male	152	67	129	98	Yes
44	Male	165	63	130	95	Yes
51	Female	176	59	135	92	No
46	Female	178	61	138	92	Yes
69	Male	165	76	105	80	No
59	Male	153	72	120	88	Yes
66	Male	153	89	127	89	Yes
54	Male	155	90	125	90	Yes
52	Male	156	88	130	92	Yes
49	Male	157	68	131	97	Yes
62	Male	156	76	129	96	Yes
41	Female	157	59	139	87	Yes
46	Male	158	85	128	92	No
50	Female	159	86	145	78	Yes
58	Male	161	67	101	84	Yes
49	Female	165	76	108	85	Yes
42	Female	166	68	115	80	No
62	Female	176	69	120	68	Yes
62	Female	171	64	128	78	No
58	Male	172	92	127	90	Yes
70	Female	143	90	129	94	Yes
62	Female	147	89	130	92	Yes
58	Female	149	88	138	91	Yes
48	Male	156	79	134	91	Yes
71	Female	155	94	135	96	Yes
52	Male	154	79	134	95	Yes
58	Female	152	81	135	94	No
66	Male	151	82	142	98	Yes
55	Female	153	76	144	84	Yes
39	Female	158	78	145	84	Yes
41	Male	166	75	143	88	Yes
66	Female	169	73	147	75	Yes

58	Female	179	83	105	95	No
58	Female	177	84	101	80	Yes
62	Female	176	82	109	80	Yes
57	Female	179	76	103	80	Yes
46	Female	178	79	121	92	Yes
51	Male	179	78	123	94	Yes
42	Female	174	89	128	95	Yes
63	Female	167	87	127	92	Yes
43	Female	168	67	125	91	No
40	Female	156	66	129	88	Yes
64	Female	159	63	132	84	Yes
57	Female	180	58	142	78	Yes
59	Male	174	56	145	90	No
52	Female	173	67	102	74	Yes
42	Female	156	58	110	77	No
51	Female	154	61	108	75	Yes
55	Male	154	63	128	85	Yes
56	Male	157	67	129	87	Yes
56	Male	159	78	135	91	Yes
67	Male	140	79	138	85	Yes
51	Female	152	92	140	91	Yes
49	Male	155	54	142	95	Yes
38	Female	145	55	148	92	Yes
66	Female	146	59	120	92	No
65	Female	165	61	110	88	Yes
43	Male	148	61	120	90	No
47	Female	153	89	123	94	Yes
39	Female	154	89	125	90	Yes
57	Female	165	90	127	94	Yes
59	Male	169	60	128	88	No
54	Male	168	66	128	75	Yes
58	Male	169	64	129	68	Yes
60	Female	148	67	129	69	No
49	Male	165	61	130	78	No
34	Female	167	62	133	77	No
71	Female	156	68	135	71	No
56	Male	155	62	137	72	Yes
55	Male	176	69	138	74	Yes
71	Female	178	75	140	76	Not Known
50	Male	179	72	142	75	No
41	Male	145	70	145	95	Yes
62	Female	146	67	148	95	No
58	Female	165	66	152	79	Yes
50	Female	155	79	129	97	No
76	Female	152	89	98	68	Yes
44	Male	150	90	99	69	No
46	Female	151	93	127	90	No

56	Male	145	94	128	92	Yes
58	Male	149	87	130	95	Yes
66	Male	145	49	131	94	Yes
71	Female	148	58	137	93	Yes
54	Male	152	84	139	91	Yes
54	Male	157	88	153	99	Yes
54	Male	156	94	90	68	Yes
59	Male	178	91	99	86	Yes
44	Male	169	68	95	78	No
64	Male	168	88	120	91	Yes
55	Male	179	69	123	93	Yes
56	Male	175	81	127	91	No
57	Male	177	83	128	99	No
67	Male	145	85	129	98	Yes
44	Male	156	76	131	95	No
63	Male	176	71	132	96	Yes
45	Male	179	72	132	92	No
37	Male	166	77	133	80	No
45	Female	174	66	137	88	Yes
60	Male	150	54	142	87	Yes
58	Female	152	58	152	81	Yes
64	Male	165	56	100	80	Yes
68	Male	176	67	101	80	No
41	Male	178	65	105	78	No
62	Female	165	68	105	80	Yes
62	Male	153	69	105	78	Yes
54	Male	153	73	101	69	Yes
70	Male	155	77	108	75	Yes
55	Female	156	79	104	95	Yes
58	Female	157	61	108	66	No
62	Male	156	55	110	74	Yes
57	Female	157	54	90	89	No
39	Female	158	50	110	78	No
64	Male	159	51	110	68	Yes
50	Male	161	55	106	99	No
60	Female	165	52	110	88	Yes
76	Female	166	51	114	77	Yes
60	Female	176	65	109	80	Yes
45	Female	171	67	115	94	Yes
43	Female	168	65	124	88	No
59	Male	156	61	125	98	No
59	Female	159	62	125	102	Yes
58	Male	180	59	120	98	No
66	Female	174	58	125	89	No
48	Female	173	81	126	98	No
56	Male	156	71	126	98	Yes
44	Male	154	72	121	85	No

45	Female	154	70	127	103	No
59	Male	157	72	120	95	Yes
55	Female	159	87	127	101	No
57	Male	140	88	127	92	No
54	Male	152	83	127	94	Yes
46	Female	155	45	128	99	Yes
59	Male	145	56	128	98	Yes
54	Male	146	67	128	91	No
46	Female	165	71	128	100	No
69	Male	148	71	129	95	Yes
43	Male	153	75	129	97	No
51	Female	154	67	130	91	No
57	Male	165	87	142	95	No
63	Female	173	69	130	96	Yes
60	Male	168	67	130	97	Yes
45	Male	169	59	130	100	No
51	Male	142	81	130	96	Yes
35	Male	165	65	133	95	Yes
54	Female	167	68	130	95	No
42	Female	156	61	132	97	Yes
68	Male	155	60	131	98	No
60	Male	176	59	132	95	Yes
59	Male	178	54	131	99	No
42	Female	179	51	131	105	Yes
59	Female	145	55	132	102	Yes
53	Female	146	51	132	95	Yes
51	Female	165	56	132	105	No
44	Male	155	74	133	105	No
56	Female	152	75	133	106	Yes
48	Male	150	76	133	95	No
57	Male	151	77	133	95	Yes
60	Female	145	65	133	105	No
48	Male	149	67	134	99	No
43	Male	145	71	134	99	No
54	Male	148	72	134	102	Yes
44	Male	152	77	135	104	Yes
48	Male	157	79	135	92	Yes
71	Male	156	75	135	96	No
63	Male	178	56	136	105	Yes
35	Male	173	56	136	92	Yes
37	Male	168	74	137	108	No
57	Male	179	67	137	106	No
46	Male	178	61	137	105	Yes
59	Male	177	65	137	105	No
64	Male	145	76	137	97	Yes
52	Male	156	49	137	89	No
41	Female	176	49	137	94	No

49	Female	179	55	138	108	No
66	Male	166	51	138	109	Yes
62	Female	174	58	138	91	Yes
43	Male	151	58	138	96	Yes
35	Male	155	56	139	90	No
41	Male	165	71	140	91	No
45	Male	176	77	140	95	Yes
40	Female	178	76	144	86	No
56	Male	165	69	140	98	Yes
71	Female	153	66	142	83	Yes
51	Female	153	72	142	81	Yes
67	Female	155	73	142	87	Yes
41	Female	156	77	144	84	Yes
41	Male	157	78	144	91	Yes
53	Male	156	56	144	90	Yes
48	Male	157	56	145	94	Yes
61	Male	158	51	145	95	Yes
39	Male	159	65	140	92	Yes
58	Male	161	65	152	93	Yes
67	Female	165	62	147	77	Not Known
59	Female	166	69	147	72	Yes
55	Male	176	70	140	98	No
70	Male	171	71	152	97	No
50	Male	172	81	124	93	No
63	Female	143	83	145	88	No
58	Male	147	83	125	94	No
77	Male	149	84	135	95	Yes
53	Male	156	84	90	92	Yes
44	Male	155	78	99	96	No
65	Male	154	77	95	96	No
58	Female	152	71	91	96	Yes
39	Female	151	77	96	80	Yes
66	Female	153	70	97	80	Yes
44	Male	158	73	93	92	Yes
40	Female	166	69	102	91	Yes
64	Female	173	66	104	90	Yes
64	Female	179	86	105	93	Yes
65	Female	177	81	120	94	No
51	Female	176	83	110	95	No
62	Male	179	69	111	96	Yes
70	Male	178	79	110	92	Yes
59	Female	179	71	103	92	Yes
61	Female	174	71	106	80	Yes
55	Male	167	67	101	88	Yes
67	Male	168	68	105	89	Yes
58	Male	156	66	109	90	No
42	Female	159	67	111	92	No

51	Female	180	60	121	97	Yes
56	Male	174	66	122	96	Yes
35	Male	173	67	108	97	Yes
67	Male	156	64	110	92	Yes
66	Male	154	72	112	96	Yes
51	Male	154	73	148	84	Yes
45	Male	157	59	132	85	No
57	Male	159	81	119	80	No
57	Male	140	82	130	68	Yes
65	Female	152	85	128	78	Yes
54	Female	155	66	127	90	Yes
71	Female	145	80	124	94	Yes
54	Male	146	51	125	92	Yes
57	Male	165	55	120	93	Yes
50	Female	148	52	121	91	No
57	Female	153	51	105	96	No
51	Female	154	65	109	95	Yes
67	Female	165	67	112	94	Yes
57	Male	173	92	128	98	Yes
66	Male	168	88	109	78	Yes
51	Female	169	82	132	85	Yes
67	Male	149	81	130	88	Yes
60	Female	165	81	138	94	No
58	Male	167	84	137	95	No
43	Female	156	67	139	80	Yes
46	Male	155	78	138	80	Yes
63	Male	176	77	145	80	Yes
59	Male	178	75	152	92	Yes
41	Male	179	75	106	94	Yes
54	Male	145	67	111	95	Yes
60	Male	146	65	125	92	No
57	Male	165	80	128	91	No
44	Male	155	81	129	88	Yes
40	Male	152	82	132	84	Yes
69	Female	153	71	121	75	Not Known
69	Female	176	80	125	95	Yes
69	Male	156	61	128	95	Yes
69	Male	156	61	129	96	Not Known
69	Male	167	84	131	97	No
69	Male	150	96	127	68	Not Known
69	Male	170	69	121	69	Yes
69	Female	171	73	120	90	Yes
69	Male	165	83	129	92	Not Known
69	Female	170	83	131	95	Not Known
70	Male	149	90	135	94	Not Known
70	Male	165	57	134	93	Not Known
70	Male	165	56	145	91	Not Known

70	Male	162	87	142	87	No
70	Female	154	70	111	68	No
70	Female	153	73	110	86	Yes
70	Female	173	106	103	78	No
70	Female	173	106	102	91	Yes
70	Female	178	81	120	93	No
70	Female	178	80	125	91	Not Known
70	Male	171	101	127	79	Not Known
70	Female	165	62	129	84	Not Known
70	Female	171	83	129	95	No
71	Female	174	75	130	96	Yes
71	Female	180	86	131	92	Not Known
71	Female	177	92	134	80	No
71	Female	165	65	137	88	Not Known
71	Female	174	91	138	87	Yes
71	Female	168	94	139	85	Not Known
71	Female	175	60	142	80	Not Known
71	Male	155	89	143	82	Yes
71	Female	178	81	143	90	No
71	Female	168	104	143	83	No
72	Male	154	59	144	85	Not Known
72	Male	163	78	146	94	Not Known
72	Male	165	80	150	95	Not Known
72	Male	160	87	106	92	Not Known
72	Male	163	62	105	96	No
72	Male	165	75	108	96	Yes
72	Female	181	94	120	96	Not Known
72	Female	168	67	120	80	Not Known
72	Female	181	78	121	80	Yes
72	Female	170	70	129	78	No
72	Female	171	72	129	95	Not Known
72	Female	173	96	129	92	Not Known
73	Female	172	75	129	91	Not Known
73	Male	143	67	130	90	Not Known
73	Male	165	71	135	93	Not Known
73	Male	165	67	138	94	Yes
73	Male	155	69	105	95	Not Known
73	Female	170	75	120	96	Not Known
73	Female	177	80	127	92	Not Known
73	Female	170	79	125	92	Not Known
73	Male	164	97	130	80	No
73	Male	160	106	131	88	Not Known
73	Female	175	80	129	89	Not Known
74	Female	162	67	139	90	Not Known
74	Male	147	64	128	92	Not Known
74	Male	155	69	145	97	Not Known
74	Male	165	70	101	96	Not Known

74	Female	163	65	108	97	Yes
74	Male	163	80	115	92	Not Known
74	Male	151	69	120	96	Not Known
74	Male	157	86	128	84	Not Known
74	Female	173	81	127	85	No
74	Female	166	90	129	80	Not Known
74	Male	148	103	130	68	Not Known
74	Female	167	78	138	78	No
74	Male	167	101	134	90	Not Known
74	Female	179	90	135	94	Not Known
74	Female	178	105	134	92	Yes
75	Female	180	69	135	93	No
75	Male	147	57	142	91	Not Known
75	Female	176	80	144	72	Not Known
75	Female	178	77	145	82	Not Known
75	Male	167	66	143	94	No
75	Male	156	60	147	86	Not Known
75	Female	181	96	105	81	Yes
75	Male	162	57	101	85	Not Known
75	Male	165	91	109	88	No
75	Male	171	97	103	94	Not Known
75	Female	167	73	121	95	Yes
75	Female	173	76	123	80	Yes
75	Male	158	84	128	80	Not Known
75	Female	175	93	127	80	Not Known
75	Female	173	97	125	92	Not Known
75	Female	178	66	129	94	Not Known
77	Male	160	84	129	69	Yes
77	Female	177	102	130	78	Yes
77	Male	158	82	133	88	No
77	Female	172	84	135	80	No
77	Female	176	109	137	83	Not Known
77	Female	177	95	138	85	Not Known
77	Female	176	93	140	86	Not Known
78	Female	177	79	142	84	No
78	Female	180	77	145	90	Not Known
78	Female	178	76	148	82	Not Known
78	Female	152	59	152	97	Yes
78	Female	173	101	145	86	Not Known
79	Female	160	64	133	92	Not Known
79	Female	175	98	137	91	Not Known
79	Male	163	84	142	88	No
79	Female	181	85	152	84	NO
79	Female	178	81	100	78	No
79	Male	164	85	101	90	No
80	Female	180	80	105	74	No
80	Female	163	72	105	77	No

80	Female	158	57	105	75	Not Known
80	Male	162	80	101	85	Yes
80	Male	168	55	108	87	Not Known
80	Male	161	66	104	91	Not Known
80	Male	163	63	108	85	No
80	Male	172	67	110	94	No
80	Female	177	78	90	95	No
80	Female	175	102	110	92	No
81	Female	164	60	110	92	No
81	Female	180	94	106	88	Not Known
81	Female	178	78	110	90	Not Known
81	Male	157	52	114	94	No
81	Male	162	67	109	90	Yes
81	Female	168	89	115	94	Not Known
81	Female	152	72	95	88	Not Known
81	Female	180	74	105	75	Yes
81	Female	175	84	119	68	Yes
81	Female	179	77	98	69	Not Known
81	Female	173	97	104	78	Not Known
81	Female	174	79	117	77	No
81	Female	177	102	120	71	No
81	Male	163	75	115	72	Not Known
81	Female	164	68	121	74	Not Known
81	Female	170	79	120	76	Yes
81	Female	173	83	123	75	Yes
81	Female	177	76	123	95	Not Known
81	Female	172	78	123	95	No
81	Female	174	91	122	96	Not Known
81	Female	163	83	124	97	No
82	Female	168	95	120	68	No
82	Female	175	85	120	69	Not Known
82	Female	169	80	120	90	Not Known
82	Male	160	79	124	92	Not Known
82	Male	155	63	123	95	Yes
82	Male	149	64	124	94	Not Known
82	Female	180	77	124	93	No
82	Male	168	64	125	91	Not Known
82	Male	163	61	125	99	No
83	Male	155	66	120	68	No
83	Female	169	78	125	86	Not Known
83	Female	170	88	126	78	Yes
83	Male	158	64	126	91	No
83	Male	162	68	121	93	Not Known
83	Male	142	62	127	91	Not Known
83	Male	158	72	120	99	Yes
83	Female	170	73	127	98	Not Known
83	Male	160	62	127	95	Not Known

83	Male	163	81	127	96	No
83	Female	173	84	128	92	Not Known
83	Female	151	59	128	80	Yes
83	Female	168	80	128	88	Not Known
83	Female	175	84	128	87	Yes
83	Female	172	81	129	85	Not Known
91	Female	175	78	129	80	Yes
85	Female	172	89	130	80	Not Known
91	Female	173	87	142	78	Not Known
89	Female	175	95	130	80	Yes
87	Female	165	87	130	78	No
84	Female	180	77	130	69	Not Known
87	Male	154	46	130	75	Not Known
91	Female	152	65	133	95	Not Known
84	Female	166	86	130	66	Yes
87	Female	171	74	132	74	Not Known
92	Female	169	72	131	89	Not Known
97	Female	160	60	132	78	Yes
84	Female	173	71	131	68	No
91	Male	161	83	131	99	No
85	Female	175	68	132	88	Yes
87	Male	160	55	132	77	Yes
91	Male	162	73	132	80	Not Known
87	Female	172	77	133	94	Not Known
92	Female	172	89	133	90	Yes
86	Male	161	59	133	78	No
87	Male	158	58	133	80	No
92	Female	178	87	133	90	Yes
84	Male	167	79	134	67	Yes
87	Female	171	72	134	80	Not Known
87	Male	166	69	134	92	Not Known
85	Male	163	67	135	95	No
87	Male	157	74	135	99	Yes
85	Male	156	55	135	92	Yes
89	Male	165	75	136	85	Not Known
87	Male	160	70	136	87	Not Known
88	Male	162	61	137	88	Not Known
88	Male	162	61	137	85	Yes
85	Female	173	67	137	95	Yes
94	Female	170	88	137	95	Yes
84	Male	155	68	137	92	Not Known
86	Male	163	68	137	94	Yes
86	Male	163	67	137	91	Not Known
85	Female	165	68	138	98	Not Known
90	Male	158	84	138	104	Yes
89	Male	160	64	138	88	Not Known
95	Male	168	59	138	98	Yes

95	Female	173	69	139	86	Yes
93	Male	165	68	140	98	Not Known
88	Female	164	89	140	89	Not Known
91	Male	150	54	144	98	Not Known
94	Female	165	77	140	84	Not Known
91	Female	160	76	142	85	Not Known
90	Male	165	65	142	103	Not Known
92	Female	178	62	142	85	Not Known
96	Female	160	73	144	77	Yes
86	Female	158	67	144	101	Not Known
84	Female	172	75	144	71	Not Known
92	Female	173	76	145	79	Not Known
91	Female	170	65	145	77	Yes
90	Male	169	78	140	103	Not Known
95	Female	169	77	152	90	Not Known
88	Male	160	65	147	95	Not Known
86	Female	168	83	147	79	Not Known
87	Female	163	70	140	90	Not Known
98	Male	162	78	152	109	Not Known
85	Female	169	77	140	96	Not Known
88	Male	148	66	145	97	Yes
92	Female	170	76	141	85	Not Known
84	Male	164	79	147	96	Yes
85	Male	162	85	95	95	Not Known
84	Female	180	88	98	95	Not Known
85	Male	162	72	108	97	No
86	Male	160	70	110	98	Not Known
84	Male	160	59	112	95	No
94	Male	155	85	148	99	Not Known
90	Male	145	51	132	105	Yes
97	Male	155	51	119	102	Not Known
85	Female	175	110	132	95	Not Known
91	Female	178	83	132	105	Not Known
90	Male	151	100	133	105	No
90	Male	170	61	133	106	Not Known
88	Male	165	62	133	95	Yes
89	Male	165	75	133	95	Not Known
92	Female	172	76	133	105	No
86	Male	155	62	134	105	Not Known
89	Male	159	54	134	99	Yes
89	Male	160	61	134	102	Not Known
92	Female	167	77	135	104	Yes
86	Female	171	57	135	103	Yes
88	Female	179	95	135	96	Not Known
89	Female	158	64	136	91	Not Known
88	Female	158	67	136	92	No
90	Female	162	54	137	92	Not Known

89	Female	168	73	137	93	No
92	Female	170	73	137	97	Yes
94	Female	157	68	137	99	No
93	Female	173	82	137	97	Not Known
87	Female	166	74	140	84	Not Known
87	Female	161	84	152	68	Yes
87	Female	173	77	134	106	Not Known
90	Female	164	58	145	78	Yes
84	Female	161	76	138	88	Yes
88	Male	168	101	131	105	Not Known
22	Male	152	56	91	67	No
22	Female	172	65	97	62	No
52	Female	153	88	108	98	Yes
65	Female	158	94	110	76	Yes
65	Female	175	91	112	80	Yes
61	Female	173	68	148	88	Yes
64	Male	179	88	132	87	Yes
51	Female	177	69	119	86	No
67	Female	176	81	130	83	Yes
60	Male	179	83	128	81	Yes
66	Male	178	85	127	89	Yes
56	Female	179	76	124	88	No
52	Male	174	71	125	87	Yes
49	Female	167	72	120	85	Yes
60	Male	168	77	121	90	No
61	Female	156	66	105	71	Yes
54	Female	159	54	109	70	No
53	Female	180	58	112	95	Yes
42	Male	174	56	128	90	Yes
67	Female	173	67	109	92	Yes
58	Male	156	65	132	88	No
57	Female	154	68	130	87	Yes
54	Female	154	69	138	78	Yes
67	Male	157	73	137	77	Yes
46	Male	159	77	139	75	Yes
64	Male	140	79	138	69	Yes
60	Female	152	61	145	84	Yes
58	Male	155	55	152	81	Yes
62	Female	145	54	106	85	Yes
57	Male	146	50	111	87	No
61	Male	165	51	125	98	Yes
65	Male	148	55	128	90	Yes
56	Male	153	52	129	89	Yes
50	Male	154	51	132	78	No
56	Male	165	65	135	90	Yes
71	Female	173	67	138	86	Yes
38	Male	168	92	142	96	No

40	Male	169	88	141	100	Not Known
64	Male	140	82	121	81	No
57	Female	165	81	128	86	Yes
47	Female	167	81	127	83	Yes
44	Male	156	84	129	92	Yes
44	Female	155	67	132	91	No
59	Male	176	78	142	90	Yes
51	Female	178	77	144	79	Yes
54	Female	179	75	145	87	Yes
51	Female	145	75	139	85	Yes
42	Male	146	67	137	91	Yes
51	Female	165	65	131	90	Yes
55	Male	155	80	151	85	Yes
60	Female	152	81	121	76	Yes
67	Female	150	82	127	74	No
45	Female	151	69	125	75	Yes
53	Male	145	65	129	78	Yes
62	Female	149	62	132	85	Yes
45	Male	145	61	131	68	No
43	Female	148	60	130	69	Yes
64	Male	152	65	138	74	Yes
66	Female	157	61	135	72	No
58	Female	156	62	139	73	Yes
58	Male	178	59	139	77	No
41	Female	173	58	120	74	Yes
68	Male	168	81	121	88	Yes
52	Male	179	71	125	78	Yes
61	Male	180	72	128	75	No
43	Female	177	68	129	86	Yes
50	Male	145	65	131	84	Yes
71	Female	156	87	127	85	Yes
59	Male	176	88	121	85	Yes
44	Male	172	71	143	94	Yes
52	Male	143	75	143	95	Yes
49	Male	147	76	143	92	Yes
59	Male	149	77	144	96	No
64	Male	156	65	146	96	Yes
60	Female	155	67	150	76	Yes
53	Male	154	71	106	80	Yes
48	Male	152	72	105	80	No
47	Male	151	77	120	92	Yes
60	Female	153	79	121	91	Yes
51	Male	158	75	129	90	No
58	Male	175	56	129	93	Yes
62	Male	173	56	129	94	No
51	Male	179	74	129	95	Yes
58	Male	177	67	130	96	Yes

64	Female	176	61	135	92	Yes
67	Male	179	65	138	92	No
53	Female	178	76	105	80	Yes
57	Female	179	49	120	88	Yes
67	Female	174	49	127	89	Yes
41	Male	167	55	125	90	Yes
53	Female	168	51	130	92	Yes
61	Female	156	58	131	97	Yes
63	Male	159	58	129	96	Yes
59	Female	180	56	139	78	Yes
55	Male	174	71	128	92	No
42	Female	173	77	145	81	Yes
58	Male	156	76	101	84	Yes
58	Female	154	69	108	85	Yes
58	Female	154	66	115	80	No
58	Female	157	72	120	68	Yes
55	Female	159	73	128	78	Yes
63	Female	140	76	127	90	No
53	Female	152	79	129	94	Yes
59	Female	155	56	130	92	Yes
54	Female	145	56	138	83	No
56	Female	146	51	134	91	Yes
67	Female	165	65	135	96	Yes
48	Female	148	65	134	95	No
62	Male	153	62	135	94	Yes
41	Male	154	69	142	98	Yes
51	Female	165	70	144	72	No
57	Male	173	71	145	85	No
35	Male	162	81	143	88	No
62	Female	169	83	147	78	Yes
46	Female	144	83	105	95	No
66	Male	165	84	101	80	Yes
62	Female	167	84	109	80	Yes
35	Male	156	78	103	80	No
58	Male	155	77	121	92	Yes
60	Female	176	71	123	94	No
54	Female	178	77	128	95	No
52	Female	179	70	127	92	No
46	Male	145	66	127	94	No
45	Male	156	72	128	88	No
65	Female	176	51	128	75	Yes
76	Female	181	55	129	68	Yes
62	Male	175	52	129	69	Yes
51	Female	174	51	130	78	No
64	Male	160	65	133	77	Yes
59	Male	164	67	135	71	No
56	Female	165	92	137	72	Yes

64	Male	176	88	138	74	Yes
61	Male	178	82	140	76	No
55	Male	165	81	142	75	No
63	Female	153	81	145	83	Yes
58	Female	153	84	148	75	No
38	Male	155	67	152	96	Yes
53	Male	156	78	133	97	No
50	Male	157	77	98	68	No
54	Male	156	75	99	69	Yes
51	Male	157	75	127	90	Yes
43	Female	158	67	128	92	Yes
49	Female	159	65	130	95	No
67	Male	161	80	131	94	Yes
65	Female	165	81	137	93	Yes
55	Male	166	82	139	91	No
70	Female	176	69	144	75	Yes
54	Male	171	65	90	68	Yes
51	Male	172	62	99	86	Yes
69	Male	143	61	95	78	No
57	Male	147	60	120	91	Yes
61	Female	149	65	123	93	No
60	Female	156	61	127	91	Yes
60	Male	155	62	128	99	Yes
43	Male	154	59	129	98	Yes
67	Male	152	58	131	95	No
64	Male	151	81	132	96	Yes
35	Male	153	71	132	92	No
59	Male	158	72	133	80	Yes
43	Male	175	71	137	88	No
54	Male	173	70	142	87	Yes
41	Male	181	87	152	85	Yes
51	Female	177	88	100	80	Yes
62	Male	176	83	137	92	Yes
64	Male	179	45	138	97	Yes
63	Female	178	56	139	79	Yes
43	Female	179	67	142	88	No
58	Female	174	71	143	88	Yes
44	Female	167	71	143	78	No
60	Female	168	75	143	89	Yes
58	Male	156	67	144	96	Yes
54	Male	159	87	146	96	Yes
54	Male	180	69	150	96	Yes
55	Male	174	67	106	80	No
61	Male	173	59	105	80	Yes
59	Male	156	81	108	78	No
57	Male	154	65	120	95	Yes
48	Male	154	68	120	92	No

39	Female	157	61	121	91	Yes
62	Male	159	60	129	90	No
49	Male	140	59	129	93	No
57	Male	152	54	129	94	No
60	Female	155	51	129	95	No
52	Male	145	55	130	96	Yes
56	Male	146	51	135	92	Yes
55	Male	165	56	138	92	Yes
41	Male	148	70	105	80	No
71	Female	153	70	120	88	No
60	Female	154	76	127	89	Yes
44	Male	165	77	125	90	Yes
54	Male	173	65	130	92	Yes
58	Male	162	67	131	97	No
64	Male	169	71	129	96	Yes
49	Female	147	72	139	71	Yes
43	Female	165	77	128	92	No
51	Male	167	79	145	96	Yes
45	Female	156	75	101	84	Yes
56	Male	155	56	108	85	Yes
66	Female	176	56	115	80	Yes
59	Male	178	74	120	68	Yes
45	Male	179	67	128	78	Yes
56	Male	145	61	127	90	Yes
46	Female	146	65	129	94	Yes
57	Female	143	89	105	86	Yes
51	Female	147	88	120	84	No
41	Female	149	70	132	90	No
63	Female	156	94	145	99	Yes
57	Male	155	79	152	97	No
59	Female	154	81	122	92	Yes
44	Female	152	82	105	94	No
57	Female	140	52	92	68	Yes
63	Female	152	55	120	75	No
70	Male	155	56	108	78	Yes
60	Female	145	60	102	84	Yes
57	Male	146	60	101	80	Yes
50	Female	165	61	101	81	No
57	Male	148	80	142	90	Yes
58	Male	153	57	121	91	Yes
62	Male	154	58	148	91	No
41	Male	165	70	145	89	No
45	Female	180	75	101	78	Yes
46	Male	179	79	108	82	Yes
66	Female	142	83	132	81	No
54	Male	140	52	112	88	Yes
62	Female	165	65	120	75	Yes

63	Female	167	67	102	95	Yes
60	Male	156	60	121	74	Yes
61	Female	155	67	120	88	No
60	Female	150	80	119	83	Yes
51	Female	178	81	123	88	Yes
49	Female	179	83	98	95	Yes
61	Female	145	60	142	90	Yes
43	Female	146	80	130	80	No
57	Female	140	65	132	78	Yes
66	Female	140	59	96	89	Yes
39	Female	152	57	120	81	No
36	Female	150	59	115	95	Yes
57	Female	151	55	135	71	Yes
70	Male	145	50	98	66	Yes
48	Female	149	53	121	80	No
39	Female	145	54	131	78	Yes
60	Female	148	59	101	95	No
42	Female	152	76	135	88	Yes
39	Male	157	71	148	94	No
63	Female	156	68	145	72	Yes
52	Male	150	79	109	94	Yes
55	Female	152	85	152	67	Yes
53	Male	179	88	108	95	No
57	Female	152	87	128	87	Yes
58	Female	152	84	121	80	Yes
63	Female	152	82	111	84	No
62	Female	145	65	105	71	Yes
70	Female	156	68	122	95	Yes
44	Male	176	76	149	78	No
54	Female	180	87	110	75	Yes
63	Female	160	90	135	77	Yes
64	Female	150	79	150	69	No
60	Female	152	78	145	81	Yes
63	Female	145	79	147	85	Yes
53	Male	165	100	145	95	Yes
62	Male	176	99	145	71	Yes
45	Male	160	87	152	88	No
54	Female	165	90	121	81	Yes
42	Male	153	83	111	75	Yes
46	Male	153	76	109	78	No
42	Male	155	80	102	68	No
45	Female	156	76	101	81	Yes
55	Female	157	68	109	90	Yes
52	Male	156	64	108	80	Yes
67	Male	157	67	121	69	No
58	Male	158	63	118	88	No
57	Male	159	59	123	68	Yes

64	Male	161	61	132	73	No
61	Male	165	76	134	85	No
50	Female	166	72	151	88	No
43	Female	176	89	120	97	Yes
63	Female	171	66	134	78	No
41	Female	172	88	111	85	No
48	Male	143	68	108	66	Yes
66	Female	147	76	135	87	Yes
45	Female	149	59	110	75	Yes
76	Female	156	78	112	78	Yes
53	Male	155	77	110	88	No
48	Male	154	67	95	80	Yes
52	Female	152	76	142	91	No
57	Female	151	68	98	89	No
41	Male	153	69	132	78	Yes
58	Female	158	64	121	82	No
37	Male	179	70	102	81	No
58	Male	180	70	107	88	Yes
64	Male	180	89	101	75	Yes
66	Female	175	70	101	95	Yes
42	Male	176	79	120	74	Yes
55	Female	167	70	125	92	Yes
63	Female	178	79	110	83	No
58	Female	179	81	134	88	Yes
45	Male	174	70	148	95	Yes
35	Male	167	70	141	92	Yes
67	Male	168	70	129	80	Yes
58	Female	156	70	130	78	Yes
41	Female	159	73	145	87	No
44	Male	180	83	134	81	Yes
41	Male	174	84	134	95	Yes
62	Female	173	82	128	71	Yes
53	Female	156	76	132	72	No
62	Female	154	79	120	80	Yes
62	Male	154	78	120	78	Yes
66	Male	157	80	101	95	Yes
51	Male	159	86	142	88	No
70	Male	140	67	120	94	Yes
46	Male	152	66	140	88	Yes
51	Male	155	63	120	88	Yes
39	Male	145	58	104	82	Yes
57	Male	146	56	142	71	No
51	Male	165	67	121	92	Yes
66	Male	148	58	99	84	Yes
47	Male	153	61	118	81	Yes
57	Male	154	63	120	88	Yes
52	Female	165	67	148	94	No

53	Female	180	78	119	75	Yes
60	Female	150	79	101	73	Yes
71	Female	145	92	139	81	Yes
68	Female	141	54	118	88	Yes
64	Female	165	55	105	78	Yes
53	Male	155	59	121	81	Yes
34	Male	156	61	149	85	Yes
59	Male	155	61	114	71	Yes
54	Male	176	89	114	92	Yes
59	Male	178	89	101	75	Yes
60	Male	179	90	145	89	Yes
52	Female	145	60	135	92	No
63	Female	146	66	110	66	No
59	Female	165	64	104	77	Yes
74	Male	155	67	119	90	Yes
65	Female	152	61	152	89	Yes
52	Male	150	62	117	84	No
54	Male	151	85	146	88	No
49	Male	145	62	138	82	Yes
40	Male	149	82	101	66	Yes
53	Female	145	75	117	89	Yes
52	Male	148	72	123	78	No
62	Male	152	70	135	82	No
69	Male	157	67	101	92	Yes
49	Male	156	66	107	79	Yes
74	Female	145	79	123	80	Yes
61	Male	160	89	148	86	No
59	Male	165	90	118	65	No
60	Male	180	65	132	71	Yes
67	Female	162	94	109	90	Yes
56	Male	178	65	142	95	No
49	Female	145	49	142	65	Yes
65	Female	156	65	104	80	Yes
46	Male	176	65	105	85	Yes
44	Female	150	88	98	85	No
69	Male	179	65	121	75	No
64	Female	155	91	145	76	Yes
41	Male	165	65	149	88	Yes
51	Female	163	88	118	77	No
64	Male	165	65	152	68	Yes
59	Female	160	81	101	69	Yes
62	Female	178	65	132	71	Yes
54	Male	165	85	152	83	No
64	Male	153	80	100	88	No
62	Female	153	80	120	95	Yes
62	Female	155	65	151	92	Yes
42	Male	156	80	121	80	No

56	Male	157	80	99	78	Yes
54	Male	156	54	95	89	Yes
57	Male	157	80	111	81	Yes
63	Female	158	56	108	95	No
44	Female	159	67	121	71	Yes
54	Male	161	80	102	74	Yes
57	Male	165	68	152	80	Yes
54	Male	166	80	121	78	Yes
51	Male	165	73	142	95	Yes
39	Male	150	77	131	88	No
66	Female	150	79	148	80	Yes
59	Male	143	61	142	72	Yes
57	Male	147	85	129	94	Yes
48	Female	149	54	108	87	Yes
65	Female	156	50	135	95	Yes
64	Male	155	85	120	87	Yes
64	Male	154	85	140	80	Yes
55	Female	152	85	150	84	No
51	Female	151	51	112	71	No
60	Female	153	85	142	89	Yes
41	Female	158	55	148	78	No
58	Female	150	92	114	75	Yes
68	Female	160	88	131	77	Yes
55	Female	165	82	142	90	No
59	Male	155	81	151	88	No
56	Male	176	81	132	85	No
41	Male	167	55	138	95	No
48	Male	178	55	101	71	Yes
61	Male	179	78	110	88	Yes
54	Male	174	77	108	81	Yes
67	Female	167	55	104	75	Yes
56	Male	168	55	101	78	Yes
66	Male	156	55	145	68	No
50	Female	159	80	110	81	No
64	Female	165	80	132	90	Yes
64	Female	165	81	132	80	No
68	Male	173	55	123	69	Yes
37	Female	156	85	129	88	Yes
63	Female	154	85	118	68	Yes
64	Female	154	55	142	73	No
44	Male	157	55	131	85	No
54	Female	159	85	95	88	Yes
52	Male	140	55	131	97	Yes
42	Female	152	85	142	78	Yes
51	Female	155	85	138	85	Yes
60	Female	145	85	105	66	Yes
43	Female	146	58	102	87	No

43	Female	165	85	128	75	No
59	Female	148	71	143	78	Yes
43	Female	153	72	122	88	Yes
41	Male	154	83	104	80	Yes
62	Female	165	90	120	91	Yes
59	Female	170	87	108	89	Yes
57	Female	172	75	105	92	No
57	Female	143	76	140	79	Yes
35	Male	147	56	152	80	No
58	Male	149	89	145	86	Yes
57	Male	156	89	100	78	Yes
48	Male	155	65	122	80	No
45	Female	154	65	102	88	No
56	Male	152	62	101	90	No
62	Female	151	69	145	92	No
71	Female	153	89	109	71	Yes
47	Male	158	71	120	95	No
57	Male	175	81	125	90	Yes
59	Male	180	83	142	85	Yes
58	Male	170	83	97	88	Yes
35	Male	174	84	128	89	No
54	Male	176	84	120	75	No
38	Male	167	78	97	77	No
65	Female	178	77	123	90	Yes
71	Female	179	71	100	88	No
57	Female	174	77	127	89	Yes
47	Male	167	70	109	92	No
42	Female	168	73	152	82	No
59	Male	156	69	118	87	No
54	Female	159	66	142	81	Yes
50	Female	180	86	99	82	Yes
68	Female	174	81	98	95	Yes
56	Male	173	83	102	98	Yes
55	Female	156	69	101	97	No
46	Male	154	78	100	95	Yes
57	Female	154	71	115	98	Yes
48	Male	157	71	114	91	No
45	Male	159	67	120	80	Yes
34	Male	140	68	123	78	No
54	Male	146	57	108	88	Yes
66	Male	165	58	132	94	Yes
55	Female	155	70	142	72	No
55	Male	152	75	145	94	Yes
48	Male	150	79	152	87	Yes
52	Female	151	83	132	95	Yes
47	Female	145	52	120	87	No
52	Female	149	65	122	80	Yes

58	Male	145	67	118	84	Yes
45	Male	148	60	105	71	Yes
74	Female	152	67	120	95	No
48	Male	157	80	123	78	Yes
41	Female	156	81	126	75	Yes
54	Female	178	83	127	77	Yes
51	Female	181	60	128	99	No
53	Male	160	61	120	88	Yes
38	Male	180	65	123	85	Yes
60	Male	177	59	124	95	Yes
52	Male	149	57	125	90	No
41	Female	145	59	124	90	Yes
49	Female	156	55	120	84	Yes
43	Female	176	50	90	88	Yes
56	Female	180	53	93	82	No
58	Female	160	54	92	66	Yes
67	Female	174	59	94	89	Yes
44	Female	157	76	99	78	Yes
54	Female	165	71	100	82	No
35	Female	165	68	124	92	Yes
42	Female	176	79	125	79	Yes
57	Female	140	50	95	80	Not Known
63	Female	152	55	121	86	Not Known
70	Female	169	82	110	65	Not Known
60	Female	145	65	92	80	Not Known
57	Male	162	85	111	88	Yes
52	Female	165	61	108	90	No
57	Female	148	72	102	92	Yes
58	Male	172	80	101	98	Not Known
62	Male	172	90	101	95	Not Known
41	Male	165	70	142	90	No
45	Female	172	100	121	85	No
46	Male	178	65	148	88	Yes
74	Female	162	85	145	89	Not Known
54	Male	144	70	101	75	No
62	Female	165	88	108	77	Not Known
63	Female	172	88	132	90	Not Known
60	Male	180	100	112	88	Yes
61	Male	155	50	120	89	Not Known
60	Female	172	93	102	92	Not Known
51	Female	178	81	121	82	No
49	Male	179	85	120	87	Not Known
61	Female	145	74	119	81	Yes
43	Female	146	62	123	82	No
57	Male	165	75	98	95	Not Known
66	Female	177	90	142	88	Not Known
39	Female	152	57	130	97	Yes

42	Male	150	46	132	95	No
57	Male	175	95	96	98	Yes
70	Female	145	50	120	91	No
48	Female	149	53	115	80	Yes
39	Female	145	72	135	78	Not Known
60	Female	159	65	98	87	No
42	Female	175	85	121	90	Yes
39	Male	157	55	131	95	No
63	Male	177	75	101	92	No
52	Female	178	96	135	97	No
55	Female	178	105	148	82	Not Known
53	Female	162	92	145	90	No
57	Female	170	92	109	93	No
58	Female	168	102	152	78	No
63	Male	156	101	108	87	Not Known
62	Female	145	71	128	95	Yes
70	Female	156	75	121	87	Yes
44	Male	176	76	111	80	Yes
54	Female	140	68	105	84	No
63	Female	172	103	122	71	Yes
64	Male	174	93	149	95	Not Known
60	Male	178	109	110	78	Yes
63	Male	180	104	135	75	Not Known
53	Male	181	100	151	77	Yes
62	Male	176	95	145	99	Not Known
45	Male	178	80	147	88	Yes
54	Female	165	90	145	85	Not Known
42	Female	153	48	145	83	Yes
46	Female	153	76	152	71	Yes
42	Male	155	80	121	88	Not Known
45	Female	156	58	111	81	Not Known
55	Female	157	60	109	75	Yes
52	Female	156	50	102	78	Not Known
67	Male	178	50	101	68	Yes
58	Female	175	77	109	81	Not Known
57	Female	180	102	108	90	Not Known
64	Male	177	75	121	80	No
61	Male	165	55	118	69	No
50	Male	175	70	123	88	No
43	Male	176	95	132	68	Yes
63	Female	171	68	134	73	No
41	Male	172	89	151	85	Yes
48	Female	143	68	120	88	Yes
66	Female	147	76	134	97	Yes
51	Female	149	73	111	78	Yes
76	Female	156	73	108	85	Yes
53	Male	156	55	135	66	Not Known

48	Male	154	77	110	87	Not Known
52	Male	152	50	112	75	Yes
41	Female	153	80	110	78	Not Known
58	Male	179	64	95	88	Yes
37	Female	176	75	142	80	Yes
58	Female	156	77	98	87	No
64	Female	174	104	132	84	Yes
66	Female	164	83	121	90	No
42	Female	176	82	102	82	No
55	Female	178	75	107	74	No
45	Male	174	75	101	80	Yes
35	Female	167	76	101	88	No
67	Female	168	72	120	85	No
41	Female	170	76	125	90	Yes
44	Female	180	105	110	81	No
41	Female	159	52	134	78	Yes
62	Female	173	90	148	84	Yes
53	Female	156	65	110	88	No
62	Male	154	65	95	75	Not Known
62	Male	158	60	142	91	No
66	Male	178	103	98	85	Not Known
38	Male	159	75	132	74	No
70	Male	140	67	121	75	Not Known
46	Male	158	55	102	88	No
51	Male	155	58	107	91	Not Known
39	Male	145	68	101	80	Yes
51	Male	165	86	101	78	Yes
66	Female	170	73	120	81	Not Known
47	Male	168	60	125	80	Not Known
28	Female	165	67	110	69	No
53	Female	174	70	134	91	Yes
60	Female	174	70	148	86	Yes
71	Female	172	106	141	90	Not Known
68	Female	142	40	129	88	Not Known
64	Female	165	55	130	81	No
31	Male	167	75	145	65	Not Known
34	Male	165	80	134	68	Yes
59	Female	179	85	134	88	Yes
54	Male	176	98	128	88	No
59	Male	171	88	132	82	No
60	Male	179	74	120	71	Not Known
52	Female	148	51	120	92	Yes
59	Female	165	71	101	84	Not Known
74	Male	171	60	142	81	No
65	Male	152	71	120	88	Yes
52	Female	150	74	140	90	No
54	Female	151	74	120	75	No

49	Male	145	55	104	73	Yes
40	Male	149	55	142	81	No
31	Female	145	42	121	88	No
52	Male	148	72	99	78	Not Known
62	Male	180	70	118	81	Not Known
69	Female	179	80	120	85	Not Known
49	Male	156	78	148	71	Not Known
74	Female	169	99	119	92	Yes
61	Male	179	107	101	75	Not Known
59	Female	159	99	139	89	No
67	Female	170	100	118	92	Not Known
56	Male	157	98	105	66	No
49	Female	145	50	121	77	No
65	Female	156	70	149	90	Not Known
46	Male	176	76	114	90	Yes
44	Male	181	76	114	84	Yes
69	Male	181	100	101	88	Yes
64	Male	154	93	145	82	Not Known
41	Male	154	68	135	66	No
51	Female	154	107	110	89	No
64	Female	165	69	104	78	Not Known
62	Female	178	77	119	82	No
54	Male	178	86	152	92	No
64	Male	148	76	117	79	Yes
62	Female	153	73	150	80	Not Known
42	Female	156	77	138	86	Yes
56	Female	157	76	101	72	Yes
54	Male	180	65	117	71	No
44	Female	159	75	123	90	No
51	Male	176	96	135	95	Not Known
39	Male	171	68	101	65	No
66	Female	172	90	107	80	Not Known
48	Female	149	54	123	85	No
65	Female	156	52	148	85	Not Known
64	Female	145	75	118	75	Yes
55	Female	152	60	132	76	Not Known
51	Female	151	51	109	88	No
41	Female	158	67	142	77	No
68	Female	181	102	142	68	Yes
55	Female	181	100	104	69	No
56	Female	168	70	105	71	Yes
41	Female	166	101	98	78	No
48	Male	178	67	121	84	No
61	Female	179	86	145	78	Not Known
67	Female	154	62	149	88	Yes
56	Female	178	85	118	95	Not Known
66	Male	168	53	152	78	No

50	Female	180	79	101	78	Yes
68	Male	173	69	132	78	Not Known
37	Female	156	78	152	66	Not Known
44	Female	157	50	100	88	No
52	Female	140	45	120	66	No
42	Male	152	71	151	80	No
51	Male	155	73	121	80	Yes
43	Female	146	75	99	98	No
43	Male	165	75	95	80	Not Known
43	Female	153	49	111	90	No
41	Male	178	62	108	88	Yes
62	Female	177	69	121	67	Yes
61	Female	165	85	102	98	Yes
48	Female	167	88	152	80	Yes
61	Male	155	72	121	71	Not Known
51	Female	178	78	142	68	Not Known
55	Female	179	80	131	95	Yes
77	Female	155	54	148	88	No
44	Female	152	54	147	78	No
44	Male	150	65	129	64	No
31	Male	151	50	108	66	Yes
69	Male	169	65	135	84	Not Known
76	Female	169	74	120	81	Not Known
68	Female	178	102	140	71	Yes
67	Female	177	88	150	81	Not Known
41	Female	154	76	112	92	No
43	Female	173	72	142	80	No
51	Male	162	67	143	88	Yes
51	Female	176	77	114	92	No
70	Male	175	105	131	76	Not Known
67	Female	162	98	142	86	No
50	Male	162	56	151	69	No
47	Female	162	74	132	93	Yes
55	Male	165	70	138	90	Yes
43	Male	176	61	101	74	Yes
65	Female	165	83	110	84	Not Known
67	Female	153	62	108	82	Not Known
35	Female	153	50	104	90	No
76	Male	181	75	101	82	Not Known
52	Male	165	65	145	95	Yes
77	Female	158	85	110	98	Yes
56	Female	159	62	132	90	No
45	Female	166	66	132	98	No
32	Male	176	85	123	80	No
35	Female	147	45	129	95	Yes
45	Female	154	56	118	99	No
71	Female	153	74	142	80	Yes

47	Female	158	78	131	80	No
35	Female	180	110	95	83	No
38	Male	171	72	131	78	Yes
65	Female	178	77	142	86	Not Known
71	Male	179	71	138	88	No
47	Male	167	70	105	88	Yes
42	Female	168	80	102	88	No
50	Female	170	80	128	70	Not Known
68	Female	149	96	146	74	No
46	Male	162	78	122	92	Not Known
48	Female	157	56	104	87	Yes
45	Male	159	67	120	87	No
34	Male	140	45	108	66	No
25	Female	155	53	99	66	No
52	Female	145	48	105	80	No
41	Female	146	43	124	80	Yes
44	Female	153	51	112	74	Yes
65	Male	165	88	140	82	Not Known
45	Female	177	81	152	78	Not Known
44	Female	165	71	112	72	Not Known
38	Male	178	105	114	90	No
44	Male	155	77	118	79	Yes
43	Female	176	98	132	80	Yes
48	Female	178	99	108	80	Not Known
35	Female	175	98	132	98	Yes
49	Female	145	74	132	81	No
48	Female	150	55	120	66	No
47	Male	145	47	135	95	No
45	Female	148	62	123	77	Yes
74	Female	149	55	101	87	No
41	Male	156	60	141	82	Yes
51	Male	181	63	142	92	No
38	Female	156	65	132	88	Yes
41	Female	145	78	147	66	No
49	Female	156	55	128	89	No
43	Female	176	62	118	78	No
44	Male	176	108	147	88	Not Known
35	Female	165	68	120	95	No
37	Female	159	66	120	80	Yes
39	Female	166	70	114	98	Yes
49	Male	143	67	149	91	Yes
43	Male	149	55	138	68	No
38	Female	158	51	121	90	No
43	Male	152	50	131	70	Yes
45	Female	179	65	138	75	No
40	Male	156	76	120	85	Yes
71	Male	159	62	121	69	No

68	Male	145	42	110	75	Yes
65	Female	148	75	126	81	Not Known
47	Female	154	51	121	80	yes
68	Female	162	76	114	77	Not Known
35	Female	154	62	129	92	No
40	Female	140	62	130	82	Yes
39	Female	165	60	101	78	No
69	Female	162	99	105	82	Yes
50	Female	165	85	138	86	No
47	Female	151	92	152	81	No
45	Female	173	85	145	87	No
39	Female	174	68	100	72	Yes
71	Female	145	68	122	79	Yes
69	Female	158	60	102	83	Not Known
73	Male	179	105	101	88	Not Known
38	Male	180	75	145	66	Yes
69	Male	156	59	109	86	No
73	Female	169	70	120	85	No
46	Female	165	62	125	79	No
70	Female	150	55	142	72	No
39	Male	145	52	97	77	No
65	Female	152	71	128	78	No
72	Male	181	102	120	80	No
34	Male	170	60	97	87	Yes
65	Male	165	77	123	89	Yes
73	Female	172	76	100	90	Not Known
68	Male	171	109	127	76	Yes
68	Female	157	54	109	79	No
40	Male	140	66	152	85	Not Known
35	Male	178	85	118	74	Yes
46	Female	146	65	142	90	No
46	Male	179	99	152	88	Yes
40	Female	147	62	145	88	No
39	Female	151	52	128	99	No
46	Female	165	65	120	87	No
46	Female	145	73	118	78	Yes
77	Female	165	65	142	86	Not Known
25	Male	177	75	92	65	No
54	Male	174	59	99	88	Yes
59	Male	168	82	98	94	No
47	Male	169	85	95	88	Yes
77	Male	165	67	99	88	Yes
45	Female	176	76	94	82	Yes
58	Male	178	68	102	71	No
46	Male	165	69	108	92	Yes
67	Male	153	64	110	84	Yes
69	Female	153	92	121	81	Yes

49	Female	155	90	99	88	No
58	Female	156	89	98	94	Yes
66	Female	157	88	92	75	Yes
52	Female	156	79	91	73	Yes
57	Male	157	94	120	81	Yes
60	Male	158	79	123	88	No
41	Male	159	81	101	78	Yes
46	Female	161	82	104	81	Yes
58	Female	165	76	115	85	Yes
43	Female	166	78	112	71	No
71	Female	176	75	102	92	Yes
69	Female	171	73	105	75	Yes
59	Female	174	79	131	90	No
41	Male	167	92	123	95	Yes
41	Male	168	54	125	65	Yes
46	Male	156	55	128	80	Yes
68	Male	159	59	124	85	No
71	Female	180	61	129	85	Yes
62	Female	174	61	126	75	Yes
46	Female	173	89	110	76	Yes
70	Male	156	89	100	88	Yes
41	Male	154	90	102	77	Yes
40	Female	154	60	103	68	No
62	Female	157	66	105	69	No
35	Male	159	64	108	81	Yes
44	Male	140	67	107	83	Yes
60	Male	152	61	106	88	Yes
58	Male	155	62	104	95	No
59	Male	145	90	109	92	Yes
54	Male	146	62	103	80	Yes
44	Male	165	92	102	78	Yes
45	Male	148	75	108	89	Yes
47	Male	153	72	120	81	Yes
41	Female	154	70	111	95	No
44	Female	165	67	102	71	No
57	Male	181	66	121	99	Yes
66	Male	179	79	122	80	Yes
51	Female	179	89	135	78	Yes
54	Male	143	90	145	95	No
56	Female	165	93	152	88	Yes
57	Male	167	94	135	94	Yes
54	Male	156	87	137	72	Yes
40	Male	155	49	138	94	Yes
67	Male	176	58	129	87	Yes
43	Male	178	84	90	95	No
41	Male	179	88	99	87	No
58	Female	145	94	98	80	Yes

58	Male	146	91	97	84	Yes
55	Female	165	68	90	71	Yes
34	Male	155	88	95	95	No
51	Female	152	69	96	78	Yes
57	Female	150	81	99	75	Yes
67	Male	151	83	97	77	Yes
47	Female	145	85	93	99	Yes
58	Female	149	76	92	88	Yes
52	Female	145	71	100	85	No
50	Female	148	72	110	95	No
44	Male	152	77	102	71	Yes
54	Male	157	66	104	88	Yes
44	Male	156	54	106	81	Yes
62	Male	178	58	120	75	No
57	Male	181	56	121	78	Yes
54	Female	179	67	135	68	Yes
65	Female	181	65	142	81	Yes
45	Female	168	68	152	90	Yes
56	Male	173	69	150	80	Yes
46	Male	145	73	142	69	No
54	Female	156	77	124	88	No
63	Female	176	79	132	68	Yes
64	Female	181	61	134	73	Yes
76	Female	179	55	129	85	Yes
40	Female	174	54	127	88	No
59	Male	174	50	125	97	Yes
63	Female	172	51	102	78	Yes
55	Female	165	55	105	85	Yes
71	Female	176	52	123	66	No
52	Male	178	51	149	87	No
56	Male	165	65	138	75	Yes
54	Female	153	67	121	78	Yes
59	Female	153	92	131	88	Yes
46	Female	155	88	138	80	No
55	Female	156	82	120	91	Yes
56	Male	157	81	121	89	Yes
61	Female	156	81	110	78	Yes
40	Male	157	84	126	82	No
59	Male	158	67	121	81	No
60	Male	159	78	114	88	Yes
58	Male	161	77	129	75	Yes
59	Female	165	75	130	95	Yes
44	Female	166	75	101	74	No
53	Female	176	67	105	92	Yes
57	Male	171	65	138	83	Yes
58	Female	172	80	152	86	Yes
54	Male	143	81	145	95	No

62	Male	147	82	100	92	No
57	Male	149	69	122	80	Yes
67	Male	156	65	102	78	Yes
57	Male	155	62	101	89	Yes
52	Female	154	61	145	81	No
38	Female	152	60	109	88	Yes
52	Male	151	65	120	95	Yes
44	Male	153	61	125	92	Yes
50	Male	158	62	142	80	No
62	Male	179	59	97	78	No
58	Female	181	58	128	89	Yes
61	Male	181	81	120	81	Yes
63	Male	175	71	97	95	Yes
65	Female	176	72	123	71	No
64	Male	167	93	100	99	Yes
44	Female	178	96	127	80	Yes
57	Female	179	87	109	78	Yes
52	Female	174	88	152	87	No
53	Female	167	83	118	88	No
67	Male	168	45	142	94	Yes
58	Male	156	56	99	72	Yes
41	Male	159	67	98	94	No
50	Male	180	71	102	87	Yes
41	Male	174	71	101	95	No
29	Female	173	75	100	87	No
61	Male	156	67	115	80	No
45	Female	154	87	114	84	No
59	Male	154	69	120	71	No
63	Female	157	67	123	95	Yes
58	Female	159	59	132	78	Yes
65	Female	140	81	111	75	Yes
66	Female	152	65	120	77	Yes
45	Female	155	68	151	99	No
64	Female	145	61	142	82	Yes
62	Female	146	60	123	85	Yes
35	Male	165	59	132	95	No
53	Male	148	54	128	90	Yes
59	Female	153	51	99	90	No
35	Male	154	55	135	84	No
64	Male	165	51	124	88	Yes
63	Male	181	56	120	82	Yes
49	Male	179	68	125	66	Yes
62	Male	179	78	132	89	Yes
45	Male	145	76	125	78	Yes
35	Female	165	77	128	82	Yes
53	Female	167	65	99	92	Yes
42	Female	156	67	98	79	No

62	Female	155	71	99	80	Yes
55	Male	176	72	99	86	Yes
61	Male	178	77	102	80	No
44	Male	179	79	108	80	Yes
59	Male	145	75	132	88	Yes
49	Male	146	56	142	90	Yes
68	Male	165	56	145	92	No
65	Female	155	74	152	80	Yes
59	Male	152	67	132	95	Yes
54	Female	150	61	120	90	No
57	Male	151	65	122	85	Yes
61	Male	145	76	118	88	Yes
53	Female	149	49	105	89	Yes
29	Male	145	49	120	75	No
56	Female	148	55	123	77	Yes
52	Male	152	51	126	90	Yes
70	Male	157	58	127	88	No
47	Male	156	58	128	89	Yes
53	Male	178	56	120	92	Yes
66	Male	177	71	123	82	Yes
46	Male	179	77	124	87	No
56	Male	181	76	125	81	Yes
57	Male	169	69	124	82	Yes
62	Male	176	66	120	95	No
63	Female	145	72	90	98	Yes
62	Female	156	73	93	97	Yes
42	Male	176	70	92	95	Yes
52	Male	181	72	94	98	No
45	Male	179	56	99	91	Yes
44	Male	174	56	100	80	Yes
52	Male	177	51	124	78	No
58	Male	170	65	125	87	Yes
43	Female	165	65	95	90	Yes
53	Male	176	62	121	95	Yes
52	Male	178	69	110	92	No
67	Male	165	70	92	97	Yes
57	Male	153	71	111	90	Yes
54	Female	153	81	108	97	No
49	Male	155	83	102	93	Yes
45	Male	156	83	101	91	Yes
62	Female	157	84	101	95	Yes
60	Male	156	73	142	80	No
41	Male	157	78	121	85	Yes
70	Male	158	77	148	88	Yes
60	Male	159	71	145	86	No
42	Male	161	77	101	87	Yes
54	Male	165	75	108	89	Yes

53	Female	166	73	132	71	Yes
58	Male	176	69	112	99	No
29	Male	171	66	120	80	Yes
62	Male	172	86	102	78	Yes
66	Female	143	81	121	95	No
54	Female	147	83	120	88	Yes
63	Female	149	69	119	94	Yes
57	Male	156	76	123	72	Yes
62	Female	155	71	98	94	No
53	Female	154	71	142	87	Yes
61	Female	152	67	130	95	Yes
62	Male	151	68	132	87	No
69	Female	153	66	96	80	Yes
55	Female	158	67	120	84	Yes
60	Female	179	60	115	71	Yes
67	Female	177	66	135	95	No
49	Female	181	67	98	78	Yes
58	Female	168	64	121	75	Yes
54	Female	176	72	131	77	No
58	Male	167	73	101	99	Yes
58	Female	178	59	135	88	Yes
65	Female	179	81	148	84	Yes
67	Male	174	82	145	95	No
63	Female	167	85	109	90	Yes
48	Male	168	66	152	90	Yes
58	Female	156	79	108	84	No
45	Male	159	52	128	88	Yes
59	Female	180	55	121	82	Yes
54	Male	174	56	111	66	Yes
64	Female	173	60	105	89	No
53	Male	156	60	122	78	Yes
57	Female	154	61	149	82	Yes
56	Female	154	57	110	92	No
42	Male	157	57	135	79	Yes
71	Female	159	58	101	80	Yes
48	Male	140	70	145	86	Yes
49	Female	152	75	147	78	No
52	Female	155	79	145	80	Yes
62	Female	145	83	145	88	Yes
65	Female	146	52	152	75	Yes
56	Female	165	65	121	92	Yes
37	Female	148	67	111	98	Yes
64	Male	153	60	109	95	Yes
63	Female	154	67	102	90	Yes
53	Female	165	80	101	85	No
66	Female	177	81	109	88	Yes
69	Male	179	83	108	89	No

66	Female	179	60	121	75	Yes
41	Male	148	61	118	77	Yes
59	Female	165	65	123	90	Yes
59	Female	167	59	132	88	Yes
56	Female	156	57	134	89	No
55	Male	155	59	151	92	Yes
52	Female	176	55	120	82	Yes
45	Female	178	50	134	87	No
67	Female	179	53	111	81	Yes
45	Male	145	54	108	82	Yes
51	Male	146	59	135	95	No
64	Female	165	76	110	98	Yes
35	Male	155	71	112	97	No
53	Male	152	68	110	95	Yes
41	Male	150	79	95	98	Yes
61	Male	151	85	142	91	Yes
54	Female	145	88	98	80	Yes
66	Male	149	87	132	78	No
62	Male	145	84	121	87	Yes
61	Male	148	82	102	90	Yes
54	Male	152	65	107	95	No
58	Male	157	68	101	92	Yes
54	Male	156	76	101	97	Yes
63	Male	178	87	120	90	No
55	Female	177	90	125	97	Yes
48	Male	179	79	110	93	No
51	Male	181	78	134	91	Yes
44	Male	175	79	148	87	Yes
43	Female	169	80	110	95	Yes
67	Male	145	65	95	87	Yes
58	Male	156	87	142	80	No
62	Male	176	90	98	84	Yes
57	Male	181	83	132	71	Yes
62	Female	169	76	121	95	No
55	Female	174	80	102	78	Yes
43	Male	178	76	107	75	Yes
34	Female	179	68	101	77	No
61	Male	165	64	101	99	Yes
51	Female	176	67	120	88	No
42	Male	178	63	125	85	Yes
57	Female	165	59	110	95	Yes
43	Female	153	61	134	71	Yes
62	Female	153	76	148	81	Yes
59	Female	155	72	141	81	No
62	Male	156	89	129	75	Yes
52	Male	157	90	130	78	Yes
58	Male	156	88	145	68	No

40	Male	157	68	134	81	Yes
63	Female	158	76	134	90	Yes
60	Female	159	59	128	80	No
61	Male	161	69	132	69	Yes
45	Male	165	68	120	88	No
50	Female	166	67	120	68	Yes
41	Male	176	76	101	73	Yes
59	Male	171	68	142	85	Yes
41	Female	172	69	120	88	Yes
51	Female	143	64	140	88	No
58	Female	147	92	120	78	Yes
59	Male	149	90	104	85	Yes
59	Female	156	89	142	66	No
57	Female	155	88	121	87	Yes
61	Female	154	79	99	75	Yes
56	Male	152	94	118	78	No
56	Male	151	79	120	88	Yes
41	Male	153	81	148	80	No
60	Male	158	82	119	87	Yes
51	Male	169	76	101	84	Yes
61	Male	177	78	139	90	Yes
57	Male	178	75	118	82	Yes
60	Female	156	73	105	74	Yes
59	Male	176	83	121	80	No
55	Male	167	84	149	88	Yes
62	Male	178	82	114	85	No
57	Female	179	76	114	90	Yes
43	Female	174	79	101	81	Yes
59	Male	167	78	145	78	Yes
57	Male	180	61	135	99	Yes
53	Female	156	70	110	88	No
45	Male	159	67	104	75	No
41	Female	180	66	119	91	Yes
41	Male	174	63	152	85	No
57	Male	173	58	117	74	Yes
37	Female	156	56	145	75	No
41	Male	154	67	138	88	Yes
59	Female	154	58	101	91	Yes
50	Female	157	61	117	80	Yes
65	Female	159	63	123	78	Yes
47	Female	140	67	135	81	Yes
46	Male	172	85	101	80	Yes
69	Female	171	87	123	91	Yes
73	Female	164	100	118	90	No
75	Female	148	55	132	88	Yes
72	Female	178	75	142	65	Yes
81	Female	146	68	105	88	Yes

50	Female	148	42	148	78	No
75	Female	176	80	118	81	Yes
35	Female	154	60	101	94	No
50	Female	158	78	132	75	Yes
25	Female	159	68	92	81	Not Known
35	Female	165	66	154	78	Yes
26	Female	154	75	111	92	No
69	Male	153	77	108	75	Yes
75	Male	159	58	121	89	No
37	Male	148	45	102	92	No
69	Female	160	85	152	78	No
50	Female	166	67	131	90	No
37	Female	156	82	148	77	Not Known
50	Female	141	63	140	82	Yes
50	Male	161	80	112	79	Yes
26	Male	176	75	142	80	Yes
75	Female	159	105	114	71	Not Known
39	Female	178	68	142	90	Yes
78	Female	156	75	132	65	Yes
34	Male	172	65	110	85	No
35	Female	165	68	101	88	Yes
78	Male	178	96	132	69	Not Known
72	Female	149	58	123	78	Yes
67	Male	177	79	129	84	No
26	Male	153	60	142	88	Yes
34	Male	174	89	131	95	Yes
34	Male	178	72	95	78	No
27	Male	172	103	142	78	Yes
38	Female	159	47	138	89	Yes
27	Male	178	105	105	88	No
38	Male	171	100	122	98	No
37	Female	176	62	108	88	No
37	Male	176	95	144	67	No
80	Male	172	80	140	68	Not Known
80	Female	159	81	132	81	Yes
80	Female	145	75	135	92	No
23	Female	175	88	97	64	Yes
34	Female	155	53	147	69	No
81	Female	140	45	150	88	Yes
23	Female	152	75	97	99	Yes
34	Female	156	62	120	82	No
82	Male	172	65	149	82	Not Known
24	Male	178	80	93	98	Not Known
81	Female	140	65	131	90	Not Known
81	Female	150	59	120	80	No
81	Female	158	76	121	95	Yes
76	Female	178	76	110	99	Yes

24	Female	172	97	90	99	No
82	Male	171	70	121	80	Yes
30	Male	172	87	101	88	Yes
25	Female	166	70	91	95	No
25	Male	178	98	91	95	Yes
25	Female	168	71	91	100	No
25	Male	149	72	95	90	Yes
25	Female	158	70	97	78	Not Known
25	Female	159	78	92	92	Yes
25	Female	158	75	123	80	No
28	Female	165	69	118	95	Yes
29	Female	154	72	142	77	Not Known
30	Female	154	58	118	66	Not Known
32	Male	181	100	140	80	Yes
33	Male	175	103	125	98	No
36	Male	152	65	120	70	No
79	Female	179	90	142	81	Not Known
79	Female	165	98	145	78	Not Known
23	Female	138	65	90	98	No
36	Female	181	103	152	79	No
55	Female	160	71	148	75	No
56	Female	172	103	142	90	No
57	Female	169	86	145	71	Yes
57	Female	177	80	142	90	Yes
58	Female	165	77	145	73	Yes
59	Female	172	91	152	74	Yes
59	Female	158	66	90	80	Yes
60	Male	165	69	120	95	Yes
60	Female	167	72	118	88	Yes
61	Male	167	87	118	91	Yes
61	Female	178	73	112	75	Yes
62	Male	156	48	129	98	Yes
62	Female	179	92	152	71	Yes
63	Female	181	98	110	75	Yes
63	Male	154	92	101	77	Yes
63	Male	161	89	125	88	No
63	Female	179	86	129	92	No
63	Male	162	65	132	87	Yes
63	Female	170	79	138	69	Yes
63	Female	170	60	152	73	No
64	Male	163	73	101	80	No
64	Female	170	89	133	92	Not Known
64	Female	179	116	144	90	Not Known
64	Female	172	107	139	90	Yes
65	Male	161	78	135	92	Yes
65	Female	181	78	141	88	Yes
66	Female	178	85	95	77	No

67	Male	151	74	135	90	Yes
67	Female	172	75	139	90	Yes
67	Female	177	97	129	92	No
68	Female	174	78	105	79	Not Known
68	Female	167	82	120	77	Yes
68	Male	162	74	142	91	No
68	Female	165	77	148	71	Not Known
68	Female	179	95	145	75	Yes
69	Female	168	82	132	90	Not Known
51	Female	159	51	120	91	No
59	Female	152	65	121	78	Yes
58	Female	146	70	123	88	No
58	Female	165	71	98	75	Yes
65	Male	155	69	135	95	Yes
59	Male	176	66	148	71	Yes
54	Male	178	86	145	99	Yes
65	Female	179	81	109	80	Yes
41	Female	146	69	108	95	Yes
64	Male	152	89	111	72	Yes
70	Male	151	63	122	87	Yes
51	Female	145	94	149	78	Yes
59	Male	149	79	110	87	Yes
64	Female	157	78	147	81	Yes
60	Female	178	84	121	91	Yes
51	Male	178	82	111	88	No
42	Male	177	76	109	85	Yes
41	Male	176	69	108	81	Yes
68	Female	178	67	121	75	Yes
62	Male	169	66	118	78	Yes
53	Male	165	58	132	81	No
44	Male	168	56	134	90	Yes
58	Female	165	67	151	80	Yes
70	Male	178	61	134	88	Yes
43	Male	153	78	135	85	Yes
59	Female	156	92	112	97	Yes
58	Female	156	55	95	85	Yes
46	Male	159	61	132	75	Yes
34	Male	165	89	102	88	Yes
59	Female	176	60	101	91	Yes
67	Male	171	66	101	89	Yes
54	Female	172	64	120	78	Yes
59	Male	147	61	110	81	Yes
59	Male	156	70	148	75	No
59	Male	155	62	141	95	Yes
60	Male	152	75	130	92	No
48	Male	153	70	134	88	No
53	Male	158	67	134	95	Yes

60	Male	178	89	120	78	Yes
70	Female	177	90	120	89	Yes
70	Female	176	93	101	81	Yes
52	Female	167	94	142	84	No
62	Female	178	87	120	71	Yes
44	Male	179	49	140	99	Yes
58	Female	174	58	120	80	No
63	Female	167	84	104	78	Yes
42	Female	168	88	142	87	Yes
54	Female	156	94	121	88	Yes
52	Female	180	68	118	88	Yes
47	Male	156	81	119	71	Yes
57	Female	154	83	101	92	No
51	Male	145	54	114	81	Yes
64	Male	146	58	101	88	Yes
65	Female	165	56	145	78	Yes
52	Female	148	67	135	81	Yes
54	Female	177	73	152	75	Yes
29	Female	179	79	139	85	Yes
54	Female	176	55	101	88	Yes
57	Female	146	63	135	80	No
64	Female	155	56	107	89	No
44	Male	151	61	118	79	No
56	Male	145	63	132	85	No
61	Male	149	67	109	74	No
50	Male	145	78	142	90	Yes
43	Male	156	55	98	87	Yes
63	Male	168	61	145	92	Yes
64	Male	179	89	118	88	Yes
50	Male	180	89	152	94	Yes
60	Male	145	60	132	88	No
52	Male	156	66	152	82	Yes
63	Male	177	61	151	84	Yes
44	Female	170	86	99	88	Yes
62	Male	172	62	95	94	Yes
62	Female	168	58	145	90	Yes
48	Male	159	54	142	68	Yes
61	Female	180	50	138	83	Yes
66	Male	174	51	105	88	Yes
46	Female	154	51	133	80	No
51	Male	154	65	122	78	Yes
52	Male	157	67	104	89	Yes
58	Female	159	92	120	81	Yes
41	Male	140	88	108	95	Yes
43	Female	152	82	144	71	Yes
67	Female	155	81	105	99	No
58	Male	145	81	124	80	Yes

62	Female	146	84	112	78	Yes
44	Male	165	67	140	95	Yes
60	Male	148	78	152	88	Yes
60	Male	153	77	112	94	No
57	Female	154	75	114	72	Yes
53	Male	165	75	118	94	No
56	Female	168	67	132	87	Yes
62	Male	177	65	108	95	Yes
61	Male	179	80	132	87	Yes
62	Female	142	81	132	80	Yes
34	Female	165	82	120	84	Yes
48	Female	167	69	135	71	No
65	Female	156	65	123	95	Yes
52	Female	155	62	101	78	Yes
56	Female	176	61	141	75	Yes
41	Male	178	60	139	77	Yes
52	Male	179	65	132	99	No
54	Male	145	61	147	88	Yes
43	Male	146	62	134	85	No
41	Male	165	59	118	95	Yes
63	Male	155	58	147	71	Yes
60	Female	152	81	120	88	Yes
41	Male	150	71	120	81	Yes
59	Male	151	72	114	75	Yes
41	Female	145	78	149	71	No
57	Female	149	79	138	68	Yes
56	Female	145	87	121	81	Yes
57	Male	148	88	131	90	Yes
60	Male	152	83	138	80	Yes
61	Female	157	45	120	69	No
42	Female	156	56	121	88	Yes
54	Male	178	67	110	68	No
49	Male	168	71	126	73	Yes
54	Male	177	71	121	85	Yes
69	Female	177	75	114	88	Yes
54	Male	181	67	129	97	Yes
64	Female	177	87	130	78	Yes
70	Female	145	69	101	85	No
47	Male	156	67	105	66	Yes
57	Female	176	59	136	87	Yes
64	Male	177	81	152	75	Yes
50	Female	177	65	145	78	Yes
40	Female	174	68	100	88	No
57	Female	173	61	122	80	Yes
37	Female	174	60	102	91	No
54	Female	165	59	101	89	Yes
49	Female	176	54	145	78	Yes

53	Female	178	51	109	82	Yes
34	Female	165	55	120	81	Yes
51	Female	153	51	125	88	Yes
55	Male	153	56	142	75	No
41	Male	155	72	97	95	Yes
52	Male	156	72	128	74	Yes
67	Male	157	76	120	92	Yes
65	Male	156	77	97	83	Yes
59	Female	157	65	123	88	No
52	Male	158	67	100	95	Yes
57	Male	159	71	127	92	No
53	Male	161	72	109	80	Yes
62	Male	165	77	152	78	Yes
49	Male	166	79	118	89	Yes
58	Male	176	75	142	81	Yes
63	Female	171	56	152	88	Yes
46	Female	172	56	145	95	No
60	Male	143	74	128	92	Yes
64	Male	147	67	120	80	Yes
54	Male	149	61	118	78	Yes
46	Male	156	65	142	89	Yes
51	Male	155	76	92	81	No
57	Male	154	49	99	95	Yes
58	Female	152	49	98	71	No
57	Male	151	55	95	99	Yes
61	Male	153	51	138	80	Yes
35	Male	158	58	137	78	Yes
50	Male	177	58	130	95	Yes
50	Male	168	56	131	88	Yes
69	Female	177	71	136	94	No
35	Female	177	77	99	72	Yes
53	Male	176	76	99	94	Yes
71	Female	177	69	98	87	Yes
49	Male	178	66	92	95	Yes
39	Male	179	72	91	87	No
48	Male	174	73	120	80	Yes
51	Male	167	70	123	84	No
29	Female	168	60	101	71	Yes
39	Female	156	56	104	95	Yes
52	Female	159	56	115	78	Yes
61	Female	180	51	112	75	Yes
67	Female	174	65	102	77	Yes
67	Female	173	65	105	99	No
49	Female	156	62	108	88	Yes
45	Male	154	69	107	85	Yes
41	Male	154	70	106	95	Yes
59	Male	157	71	109	90	Yes

57	Male	159	81	111	90	No
67	Male	140	83	100	84	Yes
58	Male	152	83	105	88	No
34	Male	155	84	120	82	Yes
57	Female	145	84	132	66	Yes
41	Female	146	78	125	89	Yes
41	Male	165	77	124	78	Yes
56	Female	148	71	128	82	Yes
56	Male	153	77	129	92	No
65	Female	154	70	124	79	Yes
69	Female	165	73	126	80	Yes
53	Female	168	69	121	86	Yes
60	Male	169	66	122	64	Yes
64	Male	179	86	132	80	No
61	Male	149	81	130	88	Yes
58	Male	165	83	131	90	No
60	Male	167	69	123	92	Yes
59	Male	156	65	125	98	Yes
52	Male	155	71	128	95	Yes
42	Male	176	71	124	90	Yes
56	Female	178	67	129	85	Yes
41	Female	179	68	126	88	No
60	Male	145	66	110	89	Yes
67	Male	146	67	100	75	Yes
65	Female	165	60	102	77	Yes
61	Female	155	66	103	90	Yes
55	Male	152	67	105	88	No
62	Female	150	64	108	89	Yes
64	Male	151	72	107	92	No
59	Male	145	73	106	82	Yes
64	Male	149	59	104	87	Yes
58	Male	145	81	109	81	Yes
57	Female	148	82	103	82	Yes
52	Female	152	85	102	95	Yes
45	Male	157	66	108	98	No
44	Female	156	58	120	97	Yes
58	Female	178	51	111	95	Yes
59	Female	168	55	102	98	Yes
50	Male	169	52	121	91	Yes
44	Male	177	51	122	80	No
66	Male	179	65	135	78	Yes
66	Male	177	67	145	87	No
59	Male	145	92	152	90	Yes
45	Female	156	88	135	95	Yes
64	Female	176	82	137	92	Yes
44	Female	177	81	138	97	Yes
55	Male	177	81	129	90	Yes

50	Female	174	84	90	97	No
58	Female	175	67	99	93	Yes
39	Female	176	78	98	91	Yes
45	Male	165	77	97	95	Yes
52	Male	176	75	90	80	Yes
41	Male	178	75	95	85	No
66	Male	165	67	96	88	Yes
69	Male	153	65	99	86	No
40	Male	153	80	97	87	Yes
49	Female	155	81	93	89	Yes
62	Female	156	82	92	71	Yes
60	Male	157	69	100	99	Yes
61	Female	156	65	110	80	Yes
56	Female	157	62	102	78	No
57	Male	158	61	104	95	Yes
68	Male	159	60	106	88	Yes
55	Male	161	65	120	94	Yes
44	Male	165	61	121	72	Yes
29	Male	166	62	135	94	No
63	Male	176	59	142	87	Yes
40	Male	171	58	152	95	Yes
64	Female	172	81	150	87	Yes
58	Female	143	71	142	80	Yes
55	Male	147	72	124	84	Yes
59	Male	149	59	132	71	Yes
59	Male	156	56	134	95	Yes
50	Male	155	87	129	78	No
67	Male	154	88	127	75	Yes
64	Male	152	83	125	77	Yes
61	Male	151	45	102	99	Yes
60	Male	153	56	105	88	Yes
69	Male	158	67	123	85	No
57	Female	177	71	149	76	Yes
64	Male	168	71	138	90	No
54	Female	177	75	121	90	Yes
57	Male	177	67	131	84	Yes
58	Female	176	87	138	88	Yes
55	Male	177	69	120	82	Yes
45	Female	178	67	121	66	Yes
52	Male	179	59	110	89	No
51	Female	174	81	126	78	Yes
67	Female	167	65	121	82	Yes
51	Female	168	68	114	92	Yes
50	Female	156	61	129	79	Yes
63	Female	159	60	130	80	No
57	Male	180	59	101	86	Yes
44	Male	174	54	105	69	No

54	Female	173	51	98	80	Yes
41	Male	156	55	152	88	Yes
40	Male	154	51	145	90	Yes
61	Female	154	56	100	92	Yes
57	Female	157	46	122	98	Yes
51	Male	159	70	102	95	No
55	Female	140	76	101	90	Yes
47	Male	152	77	145	85	Yes
53	Female	155	65	109	88	Yes
63	Female	145	67	120	89	Yes
59	Female	146	71	125	75	No
56	Male	165	72	142	77	Yes
55	Female	148	77	97	90	No
51	Female	153	79	128	88	Yes
57	Male	154	75	120	89	Yes
64	Male	165	56	97	92	Yes
34	Male	168	56	123	82	Yes
46	Female	169	74	100	87	Yes
77	Female	179	67	127	81	No
53	Female	148	61	109	82	Yes
55	Female	165	65	152	80	Yes
58	Female	167	76	118	98	Yes
57	Female	156	49	142	90	Yes
59	Female	155	49	99	95	No
42	Female	176	55	98	98	Yes
47	Female	178	51	102	91	No
56	Male	179	58	101	80	Yes
66	Female	145	58	100	78	Yes
51	Female	146	56	115	87	Yes
57	Male	165	71	114	90	Yes
64	Male	155	77	120	95	Yes
65	Female	152	76	123	92	No
54	Female	150	69	132	97	Yes
53	Female	151	66	111	90	Yes
66	Female	145	72	120	97	Yes
47	Female	149	73	154	88	Yes
52	Male	145	110	142	91	Yes
64	Male	148	82	123	87	Yes
57	Female	152	56	132	95	No
49	Male	157	56	128	87	Yes
61	Female	156	51	99	80	Yes
57	Male	178	65	135	84	Yes
63	Male	168	65	124	71	Yes
40	Male	169	62	120	95	Yes
37	Male	177	69	125	78	No
48	Male	174	70	132	75	Yes
60	Male	177	71	125	77	Yes

66	Male	145	81	128	70	Yes
56	Male	156	83	99	88	Yes
44	Male	176	83	98	85	No
38	Male	177	84	150	95	Yes
51	Male	177	70	99	71	No
64	Male	174	78	102	88	Yes
45	Male	178	77	108	81	Yes
57	Male	177	71	132	75	Yes
58	Male	165	77	142	78	Yes
71	Male	176	68	145	68	Yes
66	Male	178	73	152	81	Yes
61	Male	165	69	108	90	Yes
65	Male	153	66	110	80	No
39	Male	153	86	90	69	Yes
51	Male	155	81	110	88	No
41	Male	156	83	110	68	No
64	Male	157	69	106	73	Yes
45	Male	156	80	110	85	No
58	Male	157	52	114	88	Yes
35	Female	158	55	109	97	No
57	Male	159	56	115	78	Yes
49	Male	161	60	95	85	Yes
51	Male	165	60	105	66	No
60	Male	166	61	119	87	Yes
39	Male	176	57	98	75	No
56	Male	171	57	104	78	Yes
44	Male	172	58	117	88	Yes
57	Female	143	70	120	80	Yes
41	Female	147	75	115	87	Yes
54	Male	149	79	121	84	Yes
44	Male	156	83	120	90	Yes
50	Female	155	52	123	82	No
53	Male	154	65	123	74	Yes
61	Female	152	67	123	80	No
59	Male	151	60	122	88	Yes
58	Male	153	67	124	85	Yes
42	Female	158	80	120	90	No
64	Male	166	81	120	81	Yes
60	Male	168	83	120	78	No
70	Male	177	60	124	99	Yes
41	Male	177	61	123	88	Yes
61	Female	176	65	124	75	Yes
51	Male	177	59	124	91	Yes
58	Male	178	57	125	85	No
63	Male	179	59	125	74	Yes
52	Male	174	55	120	75	Yes
63	Female	167	50	125	88	Yes

42	Male	168	53	126	91	Yes
47	Male	156	54	126	80	Yes
74	Female	159	59	121	78	Yes
60	Female	180	76	127	81	No
56	Female	174	71	120	80	Yes
56	Female	173	68	127	69	No
70	Male	156	79	127	91	Yes
60	Female	154	85	127	88	Yes
52	Male	154	88	128	90	No
41	Male	157	87	128	88	Yes
59	Male	159	84	128	81	No
60	Female	140	82	128	65	Yes
54	Male	152	65	129	68	Yes
58	Male	155	68	129	88	Yes
66	Female	145	76	130	88	Yes
49	Female	146	87	142	82	No
34	Female	165	90	130	71	Yes
41	Female	148	79	130	92	Yes
60	Female	153	78	130	84	Yes
63	Male	154	79	130	81	Yes
47	Male	165	78	133	88	Yes
71	Female	168	74	130	94	Yes
44	Male	169	87	132	75	No
55	Female	179	90	131	73	Yes
59	Male	140	83	132	81	No
64	Male	165	76	131	88	Yes
52	Male	167	80	131	78	Yes
64	Male	156	76	132	81	No
50	Male	155	68	132	85	Yes
56	Male	176	64	132	71	No
41	Male	178	67	133	92	Yes
66	Female	179	63	133	75	Yes
54	Female	145	59	133	89	Yes
56	Male	146	61	133	92	Yes
71	Female	165	76	133	66	No
59	Male	155	72	134	77	Yes
52	Female	152	89	134	90	Yes
54	Female	150	90	134	90	Yes
51	Female	151	88	135	84	Yes
50	Female	145	68	135	88	Yes
49	Male	149	76	135	82	Yes
74	Male	145	59	142	66	No
70	Male	148	75	152	89	Yes
70	Female	152	76	100	78	No
67	Male	157	67	101	82	Yes
57	Male	156	76	105	92	Yes
55	Female	178	68	105	79	No

65	Male	168	69	105	80	Yes
58	Male	169	64	101	86	No
44	Male	180	92	108	77	Yes
62	Male	172	90	104	71	Yes
46	Male	177	89	108	90	Yes
60	Female	145	88	110	95	Yes
55	Male	156	79	90	65	No
65	Female	176	94	110	80	Yes
60	Female	180	79	110	85	Yes
51	Female	166	81	106	85	Yes
51	Female	174	82	110	75	Yes
65	Male	179	76	114	76	Yes
60	Female	156	78	109	88	Yes
44	Male	165	75	115	77	No
45	Female	176	73	95	68	Yes
56	Female	178	83	105	69	No
64	Female	165	84	119	72	Yes
55	Female	153	82	98	78	Yes
39	Female	153	76	104	84	No
52	Female	155	79	117	78	Yes
42	Male	156	78	120	88	No
63	Female	157	77	115	95	Yes
35	Female	156	70	121	78	Yes
35	Male	157	67	120	78	Yes
52	Female	158	66	123	78	Yes
67	Male	159	63	123	89	No
49	Female	161	58	123	88	Yes
45	Female	165	56	122	66	Yes
61	Female	166	67	124	80	Yes
70	Male	176	58	120	80	Yes
42	Female	171	61	120	98	Yes
52	Female	172	63	120	80	Yes
78	Female	143	67	124	90	No
63	Female	147	78	123	88	Yes
39	Female	149	79	124	67	No
42	Female	156	92	124	98	Yes
60	Female	155	54	125	80	Yes
47	Female	154	55	125	71	No
62	Male	152	59	120	68	Yes
54	Female	151	61	125	95	No
61	Female	153	61	126	95	Yes
53	Male	158	89	126	78	Yes
58	Male	166	89	121	74	Yes
58	Male	169	90	127	66	Yes
50	Male	180	60	120	84	No
53	Male	177	66	127	81	Yes
41	Male	176	64	127	71	Yes

45	Male	177	67	127	84	Yes
64	Male	165	61	128	92	Yes
63	Male	179	62	128	80	Yes
67	Female	174	72	128	88	Yes
74	Male	167	62	128	92	No
66	Male	168	68	129	76	Yes
59	Male	156	75	129	86	No
45	Male	168	72	130	69	Yes
51	Male	180	70	142	93	Yes
50	Male	174	67	130	90	No
65	Male	173	66	130	74	Yes
53	Female	156	79	130	84	No
60	Male	154	89	130	82	Yes
54	Male	154	90	133	90	Yes
43	Male	157	93	130	82	Yes
59	Male	159	94	132	95	Yes
77	Female	169	87	131	98	No
45	Female	176	49	132	90	Yes
42	Male	168	58	131	98	Yes
59	Male	176	84	131	80	Yes
70	Female	178	88	132	95	Yes
50	Male	165	94	132	99	Yes
45	Female	153	91	132	80	Yes
62	Male	153	68	133	80	No
62	Female	155	88	133	83	No
46	Male	156	69	133	78	No
62	Female	157	81	133	86	Yes
56	Male	156	83	133	88	Yes
56	Male	157	85	134	88	No
58	Male	158	76	134	88	Yes
55	Female	159	71	134	70	No
63	Male	161	72	135	74	Yes
62	Male	165	77	135	92	Yes
60	Male	166	66	135	87	Yes
51	Male	176	54	136	87	Yes
57	Male	171	58	136	66	No
39	Female	172	56	137	66	Yes
59	Male	143	67	137	80	Yes
42	Female	147	65	137	80	Yes
39	Female	149	68	137	92	Yes
46	Female	156	69	137	82	Yes
67	Female	155	73	137	78	Yes
60	Male	154	77	137	70	No
67	Male	152	79	138	90	Yes
67	Male	151	61	138	77	No
77	Female	153	55	138	80	Yes
58	Female	158	54	138	80	Yes

71	Female	166	50	139	91	Yes
48	Male	169	51	140	81	Yes
52	Female	180	55	140	66	No
35	Male	177	52	144	95	Yes
70	Female	176	51	140	77	Yes
77	Female	177	65	142	87	Yes
39	Female	178	67	142	82	Yes
62	Female	179	92	142	90	Yes
62	Male	174	88	144	88	Yes
56	Female	167	82	144	66	Yes
63	Female	168	81	144	89	Yes
54	Female	156	81	145	78	Yes
59	Male	159	84	145	88	Yes
60	Female	180	67	140	90	Yes
59	Male	174	78	152	80	No
69	Male	173	77	147	98	Yes
41	Male	156	75	147	91	No
45	Female	154	75	140	68	Yes
55	Male	154	67	152	90	Yes
51	Female	157	65	152	77	No
64	Female	159	80	145	75	Yes
59	Male	140	81	152	85	No
35	Female	152	82	150	69	Yes
62	Female	155	69	90	75	Yes
43	Male	145	65	99	81	Yes
59	Male	146	62	95	80	Yes
57	Male	165	61	91	77	No
52	Male	148	60	96	92	Yes
50	Male	153	65	97	82	Yes
57	Male	154	61	93	78	Yes
43	Male	165	62	102	82	Yes
44	Male	169	59	104	86	Yes
62	Male	169	58	105	94	Yes
60	Female	179	81	120	92	No
52	Male	144	71	110	72	Yes
52	Male	165	72	111	79	No
52	Male	167	69	110	83	Yes
41	Male	156	56	103	88	Yes
34	Male	155	87	106	66	No
42	Female	176	88	101	86	Yes
51	Female	178	83	105	85	No
42	Female	179	45	109	79	Yes
43	Female	145	56	111	72	Yes
37	Female	146	67	121	77	Yes
63	Female	165	71	122	78	Yes
44	Female	155	71	108	80	No
47	Male	152	75	110	87	Yes

63	Male	150	67	112	89	Yes
38	Male	151	87	148	90	Yes
52	Male	145	69	132	76	Yes
65	Female	149	67	119	79	Yes
52	Male	145	59	130	85	Yes
50	Female	148	81	128	74	No
54	Male	152	65	127	90	Yes
49	Male	157	68	124	88	No
39	Female	156	61	125	95	Yes
58	Female	178	60	120	99	Yes
61	Female	169	59	121	87	No
62	Female	169	54	105	78	Yes
62	Female	180	51	109	92	No
55	Female	176	55	112	65	Yes
41	Female	177	51	128	85	Yes
54	Female	145	56	109	81	Yes
43	Female	156	58	132	95	Yes
42	Male	176	59	130	92	No
47	Female	180	76	138	88	Yes
35	Male	166	77	137	80	Yes
44	Female	174	65	139	66	Yes
48	Male	175	67	138	69	Yes
47	Male	174	71	145	92	Yes
49	Female	165	72	152	75	Yes
53	Female	176	77	106	95	No
70	Male	178	79	111	67	Yes
46	Female	179	75	123	66	No
62	Female	145	65	148	68	Yes
53	Male	146	67	118	78	Yes
59	Male	165	92	132	82	No
71	Female	155	88	109	92	Yes
49	Male	152	82	142	79	No
43	Male	150	81	142	80	Yes
46	Female	151	81	104	86	Yes
41	Male	145	84	105	86	Yes
57	Male	149	67	98	71	Yes
66	Male	145	78	121	90	Yes
55	Male	148	76	145	95	Yes
43	Male	152	75	134	65	No
54	Male	157	75	118	80	Yes
53	Female	156	67	152	85	Yes
57	Female	178	65	101	85	No
35	Female	166	80	132	75	Yes
57	Female	178	81	152	76	No
42	Male	178	82	100	88	Yes
35	Male	173	69	120	77	Yes
56	Female	177	65	151	68	Yes

39	Male	145	62	121	69	Yes
35	Male	156	61	99	81	Yes
45	Male	176	60	95	83	Yes
57	Male	178	65	111	88	No
43	Female	169	61	108	95	Yes
47	Female	174	62	121	92	Yes
52	Female	178	59	102	80	No
58	Male	172	58	152	78	Yes
51	Male	165	81	121	89	No
39	Male	176	71	142	81	Yes
29	Male	178	72	131	95	Yes
67	Female	165	78	148	71	Yes
68	Male	153	79	130	85	Yes
44	Male	153	87	129	80	Yes
57	Male	155	88	108	78	Yes
63	Male	156	83	135	95	No
60	Male	157	45	120	88	Yes
56	Female	156	56	140	84	Yes
62	Female	157	67	150	72	No
60	Female	158	71	112	94	Yes
45	Female	159	71	142	87	No
52	Male	161	75	138	95	Yes
59	Male	165	67	114	87	Yes
44	Male	166	87	131	80	Yes
58	Male	176	69	142	84	Yes
60	Male	171	67	151	71	Yes
57	Female	172	59	132	95	Yes
56	Male	143	81	138	78	No
70	Male	147	65	101	75	Yes
57	Male	149	68	110	77	Yes
59	Male	156	61	108	99	No
59	Male	155	60	104	88	Yes
51	Male	154	59	101	85	No
52	Male	152	54	145	95	Yes
51	Female	151	51	110	71	Yes
57	Female	153	55	132	88	Yes
46	Male	158	51	132	81	Yes
38	Male	169	56	123	75	Yes
43	Female	166	78	129	78	Yes
70	Female	178	66	118	68	No
44	Female	177	76	142	81	Yes
68	Female	176	77	131	90	Yes
54	Male	167	65	95	80	No
61	Male	178	67	131	69	Yes
60	Male	179	71	142	88	No
41	Male	174	72	138	68	Yes
41	Male	167	77	105	73	Yes

62	Female	168	79	102	85	Yes
70	Female	156	75	128	88	Yes
41	Female	159	56	130	97	Yes
49	Female	180	56	122	78	Yes
62	Female	174	74	104	85	No
44	Male	173	67	120	66	Yes
44	Male	156	61	108	87	Yes
51	Female	154	65	144	75	No
59	Male	154	76	105	78	Yes
61	Male	157	49	124	88	No
58	Male	159	49	112	80	Yes
41	Female	140	55	140	81	Yes
68	Male	152	51	152	89	Yes
60	Male	155	58	112	78	Yes
44	Male	145	58	114	82	Yes
57	Male	146	56	118	81	Yes
49	Female	165	71	132	88	No
64	Male	148	77	108	75	Yes
61	Male	153	76	132	95	Yes
52	Female	154	69	145	74	No
60	Female	165	66	149	75	Yes
66	Male	166	72	99	83	No
47	Female	178	73	90	88	Yes
61	Female	179	68	111	95	Yes
54	Female	144	69	134	92	Yes
41	Male	165	56	104	80	Yes
64	Male	167	56	149	78	Yes
43	Female	156	51	121	89	Yes
42	Female	155	65	107	81	No
51	Male	176	65	120	88	Yes
54	Male	178	62	118	95	Yes
47	Male	179	69	142	92	No
48	Male	145	70	121	80	Yes
62	Male	146	71	140	78	No
42	Male	165	81	132	89	Yes
58	Male	155	83	120	81	Yes
56	Male	152	83	135	95	Yes
58	Male	150	84	123	71	Yes
53	Male	151	84	101	88	Yes
68	Male	145	78	141	80	Yes
42	Male	149	77	133	78	No
59	Male	145	71	132	95	Yes
41	Male	148	77	147	88	Yes
41	Male	152	70	137	94	No
35	Male	157	73	118	72	Yes
56	Male	156	69	147	94	No
47	Female	178	66	120	87	Yes

40	Male	166	86	120	95	Yes
54	Female	178	81	114	87	Yes
59	Male	178	83	149	80	Yes
60	Female	176	69	138	84	Yes
48	Male	177	70	121	71	Yes
54	Female	145	71	131	95	Yes
58	Female	156	71	138	78	Yes
58	Female	176	67	120	75	No
42	Male	179	68	121	77	Yes
68	Male	169	66	110	72	Yes
65	Male	174	67	126	88	Yes
54	Female	171	60	121	85	No
43	Female	169	66	114	95	Yes
62	Female	165	67	129	90	Yes
56	Male	176	64	130	90	Yes
43	Female	178	72	101	84	No
64	Male	165	73	105	88	Yes
62	Male	153	59	136	82	Yes
50	Male	153	81	152	66	Yes
50	Male	155	82	145	89	No
57	Female	156	85	100	78	Yes
42	Female	157	66	122	82	Yes
67	Female	156	72	102	92	Yes
43	Male	157	79	101	79	No
66	Male	158	74	145	80	Yes
44	Male	159	76	109	86	Yes
58	Male	161	87	120	81	Yes
67	Male	165	90	125	80	No
57	Female	166	83	142	88	Yes
56	Male	176	76	97	90	Yes
58	Male	171	80	128	92	Yes
39	Male	172	76	120	98	No
40	Male	143	68	97	95	Yes
68	Male	147	64	123	90	Yes
64	Male	149	67	100	85	Yes
59	Male	156	63	127	88	Yes
58	Female	155	59	109	89	Yes
29	Male	154	61	152	75	Yes
71	Female	152	76	118	77	No
60	Male	151	72	142	90	Yes
50	Male	153	89	99	88	Yes
53	Female	158	90	98	89	No
59	Male	169	88	102	92	No
65	Female	166	68	101	82	No
56	Male	179	76	100	87	Yes
42	Male	177	59	115	81	Yes
62	Male	176	79	114	82	No

50	Male	167	80	120	95	No
45	Female	178	67	123	89	Yes
58	Male	179	76	132	97	Yes
76	Female	174	68	111	95	No
59	Male	167	69	120	80	Yes
62	Female	168	64	151	91	No
53	Male	156	92	142	80	Yes
42	Female	159	90	123	78	Yes
60	Male	180	89	132	87	Yes
64	Female	174	88	128	90	Yes
61	Male	173	79	99	95	No
54	Male	156	94	135	92	Yes
65	Male	154	79	124	97	Yes
55	Male	154	81	120	90	Yes
67	Female	157	82	125	97	Yes
58	Male	159	76	132	93	Yes
46	Female	140	78	125	91	Yes
40	Female	152	75	128	95	Yes
57	Female	155	73	99	80	Yes
46	Male	145	83	98	85	No
62	Female	146	84	139	88	Yes
40	Female	165	82	99	86	Yes
44	Male	148	76	102	87	Yes
42	Female	153	79	108	89	Yes
50	Female	154	78	132	71	Yes
60	Female	165	82	142	78	Yes
71	Female	166	84	145	80	No
56	Male	178	67	152	78	Yes
52	Female	179	66	132	95	Yes
58	Female	142	63	120	88	No
52	Female	165	58	122	94	Yes
64	Female	167	56	118	72	No
60	Female	156	67	105	94	Yes
59	Female	155	58	120	87	Yes
77	Female	176	61	123	95	No
46	Male	178	63	126	87	Yes
58	Male	179	67	127	80	No
60	Female	145	78	128	84	Yes
50	Female	146	79	120	71	No
53	Male	165	92	123	95	No
58	Male	155	54	124	78	Yes
58	Male	152	55	125	75	Yes
57	Male	150	59	124	77	Yes
50	Male	151	61	120	82	No
46	Male	145	61	90	88	No
54	Male	149	89	93	85	Yes
66	Male	145	89	92	95	No

57	Male	148	90	94	90	Yes
48	Male	152	60	99	90	No
60	Male	157	66	100	84	Yes
55	Male	156	64	124	88	Yes
59	Male	178	67	125	82	No
52	Male	166	61	95	66	No
51	Male	177	62	121	89	No
69	Male	179	86	110	78	Yes
38	Male	178	62	92	82	Yes
66	Male	177	69	111	92	No
35	Female	145	75	108	79	No
42	Female	156	72	102	80	No
60	Male	176	70	101	86	Yes
59	Male	179	67	101	74	Yes
60	Female	169	66	142	80	No
74	Male	174	79	121	88	No
54	Male	177	89	148	90	Yes
55	Female	166	90	145	80	No
52	Male	165	93	101	98	Yes
42	Male	176	94	108	95	Yes
62	Female	178	87	132	90	No
56	Male	165	49	112	85	Yes
44	Male	153	58	120	88	Yes
59	Male	153	84	102	89	No
59	Male	155	88	121	75	No
47	Male	156	94	120	77	No
56	Male	157	91	119	90	Yes
67	Female	156	68	123	88	Yes
58	Male	157	88	98	89	Yes
44	Female	158	69	142	82	Yes
58	Male	159	81	130	82	Yes
56	Male	161	83	132	87	No
62	Female	165	85	96	81	Yes
67	Male	166	76	120	82	Yes
58	Female	176	71	115	95	Yes
59	Male	171	72	135	98	No
54	Female	172	77	98	97	Yes
54	Female	143	66	121	95	No
58	Female	147	54	131	85	Yes
52	Female	149	58	101	91	No
60	Female	156	56	135	80	No
57	Female	155	67	148	78	No
49	Female	154	65	145	87	Yes
42	Female	152	68	109	90	No
52	Male	151	69	152	95	No
41	Male	153	73	108	92	No
67	Female	158	77	128	97	Yes

48	Male	177	79	121	90	No
47	Female	166	61	111	97	Yes
58	Female	179	55	105	93	Yes
61	Male	177	54	122	91	Yes
60	Female	176	50	149	77	Yes
46	Male	167	51	110	95	No
46	Male	178	55	135	87	No
66	Female	179	52	145	81	Yes
68	Male	174	51	145	84	Yes
41	Female	167	65	147	71	No
59	Male	168	67	145	95	Yes
58	Male	156	92	145	78	No
57	Male	159	88	152	75	Yes
55	Male	180	82	121	77	Yes
57	Male	174	81	111	99	Yes
51	Male	173	81	109	88	No
40	Male	156	84	102	85	No
42	Female	154	67	101	95	No
49	Female	154	78	109	71	No
49	Female	157	77	108	88	No
56	Male	159	75	121	81	Yes
58	Female	140	75	118	75	No
69	Male	152	67	123	78	Yes
59	Male	155	65	132	68	Yes
48	Male	145	80	134	81	No
46	Female	146	81	151	90	Yes
41	Male	165	82	120	80	Yes
56	Male	148	69	134	69	Yes
60	Female	153	65	111	88	Yes
65	Female	154	62	108	68	No
49	Male	165	61	135	73	Yes
56	Male	166	60	110	85	Yes
52	Male	177	65	112	88	No
59	Male	179	61	110	97	Yes
61	Female	147	62	95	78	No
52	Male	165	59	142	85	Yes
48	Male	167	58	98	66	No
60	Female	156	81	132	87	Yes
53	Female	155	71	121	75	No
58	Female	176	72	102	78	Yes
52	Female	178	70	107	88	No
59	Female	179	72	101	80	No
41	Female	145	87	101	87	No
58	Female	146	88	120	84	No
64	Female	165	83	125	90	Yes
54	Female	155	45	110	82	Yes
55	Female	152	56	134	74	Yes

68	Female	150	67	148	80	No
59	Female	151	71	110	88	No
67	Female	145	71	95	85	No
54	Male	149	75	142	90	Yes
42	Male	145	67	98	81	Yes
70	Female	148	87	132	78	No
34	Female	152	69	121	90	No
52	Male	157	67	102	88	No
45	Female	156	59	107	75	No
53	Female	178	81	101	91	No
52	Female	166	65	101	85	Yes
44	Female	177	68	120	74	Yes
43	Male	179	61	125	75	No
58	Male	179	60	110	88	Yes
39	Male	177	59	134	91	No
57	Male	145	54	148	80	No
58	Male	156	51	141	78	No
37	Female	176	55	129	81	No
57	Male	179	51	130	80	No
69	Male	177	56	145	69	No
50	Female	174	77	134	91	Yes
53	Male	155	76	134	88	Yes
57	Male	154	76	128	90	Yes
46	Male	165	77	132	88	No
37	Male	176	65	120	81	No
54	Male	178	67	120	65	Yes
60	Male	165	71	101	68	Yes
48	Male	153	72	142	88	No
61	Female	153	77	120	88	Yes
46	Male	155	79	140	82	No
61	Male	156	75	120	71	No
68	Male	157	56	104	92	Yes
47	Male	156	56	142	84	Yes
67	Male	157	74	121	81	Yes
53	Female	158	67	99	88	Yes
70	Male	159	61	118	94	Yes
43	Male	161	65	120	75	No
48	Male	165	76	148	73	No
42	Male	166	49	119	81	No
42	Male	176	49	101	88	No
60	Male	171	55	139	78	Yes
47	Male	172	51	118	81	No
65	Female	143	58	105	85	No
62	Female	147	58	121	71	No
62	Female	149	56	149	92	Yes
62	Female	156	71	114	75	No
74	Female	155	77	114	89	No

63	Male	154	76	101	92	Yes
56	Female	152	69	145	66	No
41	Male	151	66	135	77	Yes
58	Female	153	72	110	90	No
35	Female	158	73	104	90	No
51	Female	177	79	119	84	Yes
64	Female	166	80	152	88	Yes
52	Female	179	56	117	82	Yes
48	Male	177	56	140	66	No
60	Female	176	51	138	89	No
71	Female	177	65	101	78	No
56	Female	152	65	117	82	No
54	Female	179	62	123	92	Yes
52	Male	174	69	135	79	Yes
62	Female	167	70	101	80	No
38	Male	168	71	107	86	No
57	Male	156	81	123	75	Yes
58	Male	153	83	148	71	No
43	Male	180	83	118	90	No
59	Male	174	84	132	95	No
63	Male	173	82	109	65	No
58	Male	156	78	142	80	Yes
57	Male	154	77	142	85	Yes
55	Male	154	71	104	85	Yes
35	Male	157	77	105	75	Yes
42	Male	159	83	98	76	Yes
56	Male	160	73	121	88	No
63	Male	162	69	145	77	No
61	Male	165	66	149	68	Yes
51	Male	168	86	118	69	No
44	Male	167	81	152	74	Yes
51	Male	169	83	101	78	Yes
39	Male	140	69	132	84	No
62	Male	152	85	152	78	Yes
61	Male	155	71	100	88	Yes
58	Male	145	71	120	95	Yes
44	Male	146	67	151	78	No
66	Female	165	68	121	78	No
52	Male	148	66	99	78	No
52	Female	153	67	95	89	Yes
56	Male	154	60	111	88	No
64	Female	165	66	108	66	Yes
70	Female	166	67	121	80	Yes
52	Male	177	64	102	80	Yes
55	Male	179	72	152	98	No
56	Male	145	73	121	80	No
46	Female	165	59	142	90	Yes

42	Female	167	81	131	88	No
37	Female	156	82	148	67	No
50	Female	155	85	143	88	Yes
58	Male	176	66	129	80	No
57	Male	178	86	108	71	No
52	Male	179	52	135	68	Yes
58	Female	145	55	120	95	No
60	Female	146	56	140	85	Yes
65	Female	165	60	150	78	No
50	Female	155	60	112	76	Yes
66	Female	152	61	142	66	Yes
67	Male	150	57	147	84	Yes
48	Female	151	57	114	81	No
58	Male	145	58	131	71	Yes
54	Male	149	70	142	84	No
44	Male	145	75	151	92	No
65	Female	148	79	132	80	Yes
66	Female	152	83	138	88	No
67	Female	157	52	101	92	No
46	Female	156	65	110	76	Yes
49	Male	178	67	108	86	No
55	Female	166	60	104	69	No
63	Female	177	67	101	93	Yes
40	Female	179	80	145	90	Yes
59	Male	180	81	110	74	No
43	Male	177	83	132	84	No
67	Male	145	60	132	82	No
69	Female	156	61	123	90	No
55	Male	176	65	129	82	Yes
43	Male	179	59	118	95	No
44	Female	177	57	142	89	Yes
52	Male	174	59	131	90	Yes
57	Female	169	55	95	98	No
57	Female	171	50	131	80	No
58	Male	165	53	142	95	No
70	Male	176	54	138	99	No
35	Female	178	59	105	80	Yes
66	Male	165	76	102	80	Yes
58	Male	153	71	128	83	No
64	Female	153	76	148	82	Yes
39	Male	158	68	141	81	No
67	Male	177	64	129	66	No
55	Male	168	67	130	95	No
41	Female	179	63	145	77	No
46	Female	177	59	134	87	Yes
57	Female	176	61	134	82	No
57	Female	177	76	128	92	Yes

55	Female	178	72	132	88	No
60	Female	179	89	120	66	Yes
49	Female	174	90	120	89	No
51	Male	167	88	101	78	No
57	Male	168	68	142	88	Yes
61	Male	156	76	120	95	Yes
55	Female	159	59	140	80	No
66	Male	180	80	120	98	Yes
56	Male	174	81	104	91	Yes
48	Male	173	67	142	68	Yes
56	Male	156	76	121	90	Yes
63	Male	154	68	99	70	Yes
55	Female	154	69	118	75	No
51	Female	157	64	120	85	No
59	Female	159	92	148	69	No
52	Female	140	90	119	75	No
64	Male	152	89	101	81	No
57	Male	155	88	139	80	Yes
58	Female	145	79	118	77	No
42	Male	146	94	105	92	No
67	Male	165	79	121	82	Yes
58	Male	148	81	149	78	Yes
69	Male	153	82	114	82	No
66	Male	154	76	114	86	Yes
63	Male	165	78	101	94	Yes
51	Male	168	75	145	92	Yes
64	Male	177	73	135	72	No
57	Male	179	83	110	79	No
52	Male	143	84	104	83	Yes
45	Male	165	82	119	88	Yes
59	Male	167	76	152	66	Yes
46	Female	156	79	117	86	Yes
46	Female	155	78	146	85	No
41	Female	176	82	138	79	No
57	Male	146	60	101	80	Yes
50	Female	165	61	101	81	No
57	Male	148	80	142	90	Yes
58	Male	153	57	121	91	Yes
62	Male	154	58	148	91	No
41	Male	165	70	145	89	No
46	Male	179	79	108	82	Yes
61	Female	155	67	120	88	No
51	Female	178	81	123	88	Yes
43	Female	146	80	130	80	No
39	Female	152	57	120	81	No
36	Female	150	59	115	95	Yes
70	Male	145	50	98	66	Yes

48	Female	149	53	121	80	No
39	Female	145	54	131	78	Yes
60	Female	148	59	101	95	No
42	Female	152	76	135	88	Yes
39	Male	157	71	148	94	No
63	Female	156	68	145	72	Yes
52	Male	150	79	109	94	Yes
55	Female	152	85	152	67	Yes
53	Male	179	88	108	95	No
44	Male	176	76	149	78	No
54	Female	180	87	110	75	Yes
42	Male	153	83	111	75	Yes
42	Male	155	80	102	68	No
45	Female	156	76	101	81	Yes
55	Female	157	68	109	90	Yes
52	Male	156	64	108	80	Yes
67	Male	157	67	121	69	No
58	Male	158	63	118	88	No
57	Male	159	59	123	68	Yes
64	Male	161	61	132	73	No
61	Male	165	76	134	85	No
50	Female	166	72	151	88	No
43	Female	176	89	120	97	Yes
63	Female	171	66	134	78	No
41	Female	172	88	111	85	No
48	Male	143	68	108	66	Yes
66	Female	147	76	135	87	Yes
45	Female	149	59	110	75	Yes
76	Female	156	78	112	78	Yes
52	Female	152	76	142	91	No
57	Female	151	68	98	89	No
58	Female	158	64	121	82	No
37	Male	179	70	102	81	No
58	Male	180	70	107	88	Yes
66	Female	175	70	101	95	Yes
55	Female	167	70	125	92	Yes
45	Male	174	70	148	95	Yes
67	Male	168	70	129	80	Yes
58	Female	156	70	130	78	Yes
44	Male	180	83	134	81	Yes
41	Male	174	84	134	95	Yes
53	Female	156	76	132	72	No
51	Male	159	86	142	88	No
46	Male	152	66	140	88	Yes
51	Male	155	63	120	88	Yes
47	Male	153	61	118	81	Yes
52	Female	165	67	148	94	No

53	Female	180	78	119	75	Yes
68	Female	141	54	118	88	Yes
64	Female	165	55	105	78	Yes
59	Male	155	61	114	71	Yes
63	Female	146	66	110	66	No
74	Male	155	67	119	90	Yes
54	Male	151	85	146	88	No
40	Male	149	82	101	66	Yes
53	Female	145	75	117	89	Yes
69	Male	157	67	101	92	Yes
74	Female	145	79	123	80	Yes
61	Male	160	89	148	86	No
59	Male	165	90	118	65	No
60	Male	180	65	132	71	Yes
67	Female	162	94	109	90	Yes
56	Male	178	65	142	95	No
49	Female	145	49	142	65	Yes
65	Female	156	65	104	80	Yes
46	Male	176	65	105	85	Yes
44	Female	150	88	98	85	No
69	Male	179	65	121	75	No
64	Female	155	91	145	76	Yes
41	Male	165	65	149	88	Yes
51	Female	163	88	118	77	No
64	Male	165	65	152	68	Yes
59	Female	160	81	101	69	Yes
62	Female	178	65	132	71	Yes
54	Male	165	85	152	83	No
64	Male	153	80	100	88	No
62	Female	155	65	151	92	Yes
54	Male	156	54	95	89	Yes
63	Female	158	56	108	95	No
44	Female	159	67	121	71	Yes
57	Male	165	68	152	80	Yes
48	Female	149	54	108	87	Yes
65	Female	156	50	135	95	Yes
51	Female	151	51	112	71	No
41	Female	158	55	148	78	No
41	Male	167	55	138	95	No
48	Male	178	55	101	71	Yes
67	Female	167	55	104	75	Yes
56	Male	168	55	101	78	Yes
66	Male	156	55	145	68	No
64	Female	165	81	132	80	No
68	Male	173	55	123	69	Yes
64	Female	154	55	142	73	No
44	Male	157	55	131	85	No

52	Male	140	55	131	97	Yes
43	Female	146	58	102	87	No
43	Female	165	85	128	75	No
43	Female	153	72	122	88	Yes
41	Male	154	83	104	80	Yes
63	Female	179	83	105	82	No
54	Male	139	90	124	81	Yes
77	Male	155	81	149	78	Yes
44	Male	152	65	99	89	No
54	Male	152	55	107	89	No
57	Male	157	51	120	81	No
66	Female	156	56	118	95	Yes
67	Male	180	71	121	98	Yes
41	Male	179	76	140	80	Yes
43	Male	180	77	132	78	Yes
51	Male	180	67	135	88	No
50	Male	155	56	149	80	No
47	Male	159	74	118	84	Yes
43	Male	176	61	120	95	Yes
55	Male	178	65	120	78	Yes
67	Male	153	49	149	77	No
35	Male	153	49	138	92	No
66	Male	157	56	121	90	Yes
77	Female	158	71	110	84	No
56	Male	159	77	126	88	No
56	Male	161	76	121	82	No
54	Male	165	69	114	66	Yes
45	Male	166	66	129	89	No
59	Male	171	73	101	82	No
35	Male	147	56	152	80	No
48	Male	155	65	122	80	No
45	Female	154	65	102	88	No
56	Male	152	62	101	90	No
62	Female	151	69	145	92	No
47	Male	158	71	120	95	No
35	Male	174	84	128	89	No
38	Male	167	78	97	77	No
65	Female	178	77	123	90	Yes
71	Female	179	71	100	88	No
57	Female	174	77	127	89	Yes
47	Male	167	70	109	92	No
42	Female	168	73	152	82	No
54	Female	159	66	142	81	Yes
50	Female	180	86	99	82	Yes
55	Female	156	69	101	97	No
46	Male	154	78	100	95	Yes
48	Male	157	71	114	91	No

45	Male	159	67	120	80	Yes
34	Male	140	68	123	78	No
56	Male	152	66	132	87	No
53	Male	155	67	111	90	No
52	Male	145	60	120	95	Yes
41	Male	146	66	151	92	No
44	Male	153	72	132	97	Yes
45	Female	180	81	135	95	Yes
66	Male	139	66	125	88	Yes
44	Female	165	80	132	86	Yes
38	Male	156	55	128	89	No
54	Male	146	57	108	88	Yes
66	Male	165	58	132	94	Yes
55	Female	155	70	142	72	No
48	Male	150	79	152	87	Yes
52	Female	151	83	132	95	Yes
47	Female	145	52	120	87	No
52	Female	149	65	122	80	Yes
74	Female	152	67	120	95	No
41	Female	156	81	126	75	Yes
54	Female	178	83	127	77	Yes
51	Female	181	60	128	99	No
38	Male	180	65	123	85	Yes
52	Male	149	57	125	90	No
41	Female	145	59	124	90	Yes
49	Female	156	55	120	84	Yes
43	Female	176	50	90	88	Yes
56	Female	180	53	93	82	No
35	Female	165	68	124	92	Yes
42	Female	176	79	125	79	Yes
57	Female	140	50	95	80	Not Known
63	Female	152	55	121	86	Not Known
60	Female	145	65	92	80	Not Known
52	Female	165	61	108	90	No
58	Male	172	80	101	98	Not Known
62	Male	172	90	101	95	Not Known
45	Female	172	100	121	85	No
54	Male	144	70	101	75	No
62	Female	165	88	108	77	Not Known
61	Male	155	50	120	89	Not Known
51	Female	178	81	121	82	No
49	Male	179	85	120	87	Not Known
43	Female	146	62	123	82	No
57	Male	165	75	98	95	Not Known
39	Female	152	57	130	97	Yes
42	Male	150	46	132	95	No
57	Male	175	95	96	98	Yes

70	Female	145	50	120	91	No
48	Female	149	53	115	80	Yes
60	Female	159	65	98	87	No
39	Male	157	55	131	95	No
63	Male	177	75	101	92	No
57	Female	170	92	109	93	No
44	Male	176	76	111	80	Yes
54	Female	140	68	105	84	No
63	Female	172	103	122	71	Yes
63	Male	180	104	135	75	Not Known
42	Male	155	80	121	88	Not Known
45	Female	156	58	111	81	Not Known
55	Female	157	60	109	75	Yes
52	Female	156	50	102	78	Not Known
58	Female	175	77	109	81	Not Known
64	Male	177	75	121	80	No
61	Male	165	55	118	69	No
50	Male	175	70	123	88	No
43	Male	176	95	132	68	Yes
63	Female	171	68	134	73	No
51	Female	149	73	111	78	Yes
53	Male	156	55	135	66	Not Known
48	Male	154	77	110	87	Not Known
52	Male	152	50	112	75	Yes
58	Male	179	64	95	88	Yes
58	Female	156	77	98	87	No
66	Female	164	83	121	90	No
42	Female	176	82	102	82	No
55	Female	178	75	107	74	No
45	Male	174	75	101	80	Yes
35	Female	167	76	101	88	No
67	Female	168	72	120	85	No
41	Female	170	76	125	90	Yes
44	Female	180	105	110	81	No
41	Female	159	52	134	78	Yes
53	Female	156	65	110	88	No
62	Male	154	65	95	75	Not Known
38	Male	159	75	132	74	No
46	Male	158	55	102	88	No
39	Male	145	68	101	80	Yes
51	Male	165	86	101	78	Yes
66	Female	170	73	120	81	Not Known
47	Male	168	60	125	80	Not Known
28	Female	165	67	110	69	No
68	Female	142	40	129	88	Not Known
64	Female	165	55	130	81	No
31	Male	167	75	145	65	Not Known

59	Female	179	85	134	88	Yes
59	Male	171	88	132	82	No
60	Male	179	74	120	71	Not Known
59	Female	165	71	101	84	Not Known
74	Male	171	60	142	81	No
54	Female	151	74	120	75	No
49	Male	145	55	104	73	Yes
40	Male	149	55	142	81	No
52	Male	148	72	99	78	Not Known
62	Male	180	70	118	81	Not Known
69	Female	179	80	120	85	Not Known
49	Male	156	78	148	71	Not Known
56	Male	157	98	105	66	No
49	Female	145	50	121	77	No
65	Female	156	70	149	90	Not Known
46	Male	176	76	114	90	Yes
41	Male	154	68	135	66	No
51	Female	154	107	110	89	No
64	Female	165	69	104	78	Not Known
62	Female	178	77	119	82	No
62	Female	153	73	150	80	Not Known
42	Female	156	77	138	86	Yes
56	Female	157	76	101	72	Yes
54	Male	180	65	117	71	No
39	Male	171	68	101	65	No
48	Female	149	54	123	85	No
65	Female	156	52	148	85	Not Known
57	Female	140	50	95	68	Not Known
63	Female	152	55	121	88	Not Known
70	Male	169	82	110	90	Not Known
60	Female	145	65	92	81	Not Known
57	Male	162	85	111	68	Yes
52	Female	165	61	108	75	No
57	Female	148	72	102	78	Yes
58	Male	172	80	101	84	Not Known
62	Male	172	90	101	80	Not Known
41	Male	165	70	142	81	No
45	Female	172	100	121	90	No
46	Male	178	65	148	91	Yes
74	Female	162	85	145	91	Not Known
54	Male	144	70	101	89	No
62	Female	165	88	108	78	Not Known
63	Female	172	88	132	82	Not Known
60	Male	180	100	112	81	Yes
61	Male	155	50	120	88	Not Known
60	Female	172	93	102	75	Not Known
51	Female	178	81	121	95	No

49	Male	179	85	120	74	Not Known
61	Female	145	74	119	92	Yes
43	Female	146	62	123	83	No
57	Male	165	75	98	88	Not Known
66	Male	177	90	142	95	Not Known
39	Female	152	57	130	92	Yes
42	Male	150	46	132	80	No
57	Male	175	95	96	78	Yes
70	Female	145	50	120	89	No
48	Female	149	53	115	81	Yes
39	Female	145	72	135	95	Not Known
60	Female	159	65	98	71	No
42	Male	175	85	121	99	Yes
39	Male	157	55	131	80	No
63	Male	177	75	101	78	No
52	Male	178	96	135	95	No
55	Male	183	105	148	88	Not Known
53	Female	162	92	145	94	No
57	Female	170	92	109	72	No
58	Female	168	102	152	94	No
63	Male	182	101	108	87	Not Known
62	Female	145	71	128	95	Yes
70	Female	156	75	121	87	Yes
44	Male	176	76	111	80	Yes
54	Female	140	68	105	84	No
63	Female	172	103	122	71	Yes
64	Male	174	93	149	95	Not Known
60	Male	178	109	110	78	Yes
63	Male	180	104	135	75	Not Known
53	Male	181	100	155	77	Yes
62	Male	176	95	145	99	Not Known
45	Male	178	80	147	88	Yes
54	Female	165	90	145	85	Not Known
42	Female	153	48	145	95	Yes
46	Female	153	76	152	71	Yes
42	Male	155	80	121	88	Not Known
45	Female	156	58	111	81	Not Known
55	Female	157	60	109	75	Yes
52	Female	156	50	102	78	Not Known
67	Male	178	50	101	68	Yes
58	Female	175	77	109	81	Not Known
57	Female	180	102	108	90	Not Known
64	Male	177	75	121	80	No
61	Male	165	55	118	69	No
50	Male	175	70	123	88	No
43	Male	176	95	132	68	Yes
63	Female	171	68	134	73	No

41	Male	172	89	151	85	Yes
48	Female	143	68	120	88	Yes
66	Female	147	76	134	97	Yes
51	Female	149	73	111	78	Yes
76	Female	156	73	108	85	Yes
53	Male	156	55	135	66	Not Known
48	Male	154	77	110	87	Not Known
52	Male	152	50	112	75	Yes
41	Female	153	80	110	78	Not Known
58	Male	179	64	95	88	Yes
37	Female	176	75	142	80	Yes
58	Female	156	77	98	87	No
64	Female	174	104	132	84	Yes
66	Female	164	83	121	90	No
42	Female	176	82	102	82	No
55	Female	178	75	107	74	No
45	Male	174	75	101	80	Yes
35	Male	167	76	101	88	No
67	Male	168	72	120	85	No
41	Male	170	76	125	90	Yes
44	Male	180	105	110	81	No
41	Female	159	52	134	78	Yes
62	Female	173	90	148	99	Yes
53	Female	156	65	141	88	No
62	Male	154	65	129	75	Not Known
62	Male	158	60	130	91	No
66	Male	178	103	145	85	Not Known
38	Male	159	75	134	74	No
70	Male	140	67	134	75	Not Known
46	Male	158	55	128	88	No
51	Male	155	58	132	91	Not Known
39	Male	145	68	120	80	Yes
51	Male	165	86	120	78	Yes
66	Female	170	73	101	81	Not Known
47	Male	168	60	142	80	Not Known
28	Female	165	67	120	69	No
53	Female	174	70	140	91	Yes
60	Female	174	70	120	88	Yes
71	Female	172	106	104	90	Not Known
68	Female	134	40	142	88	Not Known
64	Female	165	55	121	81	No
31	Male	167	75	99	65	Not Known
34	Male	165	80	118	68	Yes
59	Female	179	85	120	88	Yes
54	Male	176	98	148	88	No
59	Male	171	88	119	82	No
60	Male	179	74	101	71	Not Known

52	Female	148	51	139	92	Yes
59	Female	165	71	118	84	Not Known
74	Male	171	60	105	81	No
65	Male	152	71	121	88	Yes
52	Female	150	74	149	94	No
54	Female	151	74	114	75	No
49	Male	145	55	114	73	Yes
40	Male	149	55	101	81	No
31	Female	145	42	145	88	No
52	Male	148	72	135	78	Not Known
62	Male	180	70	110	81	Not Known
69	Female	179	80	104	85	Not Known
49	Male	156	78	119	71	Not Known
74	Female	169	99	152	92	Yes
61	Male	183	107	117	75	Not Known
59	Female	159	99	155	89	No
67	Female	170	100	138	92	Not Known
56	Male	182	98	101	66	No
49	Female	145	50	117	77	No
65	Female	156	70	123	90	Not Known
46	Male	176	76	135	90	Yes
44	Male	181	76	101	84	Yes
69	Male	181	100	107	88	Yes
64	Male	154	93	123	82	Not Known
41	Male	154	68	148	66	No
51	Female	154	107	118	89	No
64	Female	165	69	132	78	Not Known
62	Female	178	77	109	82	No
54	Male	178	86	142	92	No
64	Male	148	76	142	79	Yes
62	Female	153	73	104	80	Not Known
42	Female	156	77	105	86	Yes
56	Female	157	76	98	62	Yes
54	Male	180	65	121	71	No
44	Female	159	75	145	90	No
51	Male	176	96	155	95	Not Known
39	Male	171	68	118	65	No
66	Female	172	90	152	80	Not Known
48	Female	149	54	101	85	No
65	Female	156	52	132	85	Not Known
64	Female	145	75	152	75	Yes
55	Male	152	60	100	76	Not Known
51	Female	151	51	120	88	No
41	Female	158	67	151	77	No
68	Male	181	102	121	68	Yes
55	Female	181	100	99	69	No
56	Female	168	70	95	61	Yes

41	Female	182	101	111	78	No
48	Male	178	67	108	84	No
61	Female	179	86	121	78	Not Known
67	Female	154	62	102	88	Yes
56	Male	178	85	152	95	Not Known
66	Male	168	53	121	78	No
50	Male	180	79	142	78	Yes
68	Male	173	69	131	78	Not Known
37	Male	156	78	148	89	Not Known
44	Male	157	50	157	88	No
52	Female	140	45	129	66	No
42	Male	152	71	108	80	No
51	Male	155	73	135	80	Yes
43	Female	146	75	120	98	No
43	Male	165	75	140	80	Not Known
43	Female	153	49	150	90	No
41	Male	178	62	112	88	Yes
62	Female	177	69	142	67	Yes
61	Female	165	85	158	98	Yes
48	Male	167	88	114	80	Yes
61	Male	155	72	131	71	Not Known
51	Female	178	78	142	68	Not Known
55	Female	179	80	151	95	Yes
77	Female	155	54	132	95	No
44	Female	152	54	138	78	No
44	Male	150	65	101	64	No
31	Male	151	50	110	66	Yes
69	Male	169	65	108	84	Not Known
76	Male	169	74	104	81	Not Known
68	Female	178	102	101	71	Yes
67	Female	177	88	145	84	Not Known
41	Female	154	76	110	92	No
43	Female	173	72	132	80	No
51	Male	182	67	132	88	Yes
51	Female	176	77	123	92	No
70	Male	175	105	129	76	Not Known
67	Female	162	98	118	86	No
50	Male	162	56	142	69	No
47	Female	162	74	131	93	Yes
55	Male	165	70	95	90	Yes
43	Male	176	61	131	74	Yes
65	Male	165	83	142	84	Not Known
67	Female	153	62	138	82	Not Known
35	Female	153	50	105	90	No
76	Male	181	75	102	82	Not Known
52	Male	165	65	128	95	Yes
77	Female	158	85	155	98	Yes

56	Female	159	62	122	90	No
45	Female	166	66	104	98	No
32	Male	176	85	120	80	No
35	Female	147	45	108	95	Yes
45	Female	154	56	144	99	No
71	Female	153	74	105	80	Yes
47	Male	158	78	124	80	No
35	Male	182	110	112	83	No
38	Male	171	72	140	78	Yes
65	Female	178	77	152	86	Not Known
71	Male	179	71	112	88	No
47	Male	167	70	114	88	Yes
42	Female	168	80	118	88	No
50	Female	170	80	132	70	Not Known
68	Female	149	96	108	74	No
46	Male	162	78	132	92	Not Known
48	Female	157	56	145	87	Yes
45	Male	159	67	149	87	No
34	Male	140	45	99	66	No
25	Male	155	53	90	66	No
52	Male	145	48	111	80	No
41	Female	146	43	134	80	Yes
44	Male	153	51	104	61	Yes
65	Male	165	88	149	82	Not Known
45	Female	177	81	121	78	Not Known
44	Female	165	71	107	62	Not Known
38	Male	182	105	120	90	No
44	Male	155	77	118	63	Yes
43	Female	176	98	142	80	Yes
48	Female	178	99	121	80	Not Known
35	Female	175	98	140	98	Yes
49	Male	145	74	132	81	No
48	Male	150	55	120	66	No
47	Male	145	47	135	95	No
45	Female	148	62	123	77	Yes
74	Female	149	55	101	87	No
41	Male	156	60	141	82	Yes
51	Male	181	63	158	92	No
38	Female	156	65	132	88	Yes
41	Female	145	78	147	66	No
49	Female	156	55	157	89	No
43	Female	176	62	118	78	No
44	Male	176	108	147	88	Not Known
35	Female	165	68	120	95	No

Healthy Diet	Physical Activity	Alcohol Consumption	Smoking	SocioEconomicLevel
Following	No Exercise	Non Alcoholic	Regular	Poor
Not Following	Regular Exercise	Non Alcoholic	Occasionally	High Class
Following	No Exercise	Occasionally	Regular	Poor
Following	Occasionally	Occasionally	Non Smoker	Poor
Occasionally	Occasionally	Non Alcoholic	Regular	Middle Class
Not Following	Regular Exercise	Non Alcoholic	Regular	High Class
Occasionally	No Exercise	Non Alcoholic	Occasionally	High Class
Occasionally	No Exercise	Non Alcoholic	Regular	Poor
Not Following	Regular Exercise	Non Alcoholic	Occasionally	Poor
Occasionally	Regular Exercise	Non Alcoholic	Regular	Poor
Occasionally	No Exercise	Non Alcoholic	Non Smoker	Middle Class
Occasionally	Regular Exercise	Non Alcoholic	Occasionally	High Class
Occasionally	No Exercise	Non Alcoholic	Regular	High Class
Occasionally	No Exercise	Occasionally	Regular	High Class
Occasionally	No Exercise	Non Alcoholic	Regular	Poor
Occasionally	Regular Exercise	Occasionally	Occasionally	Poor
Occasionally	Regular Exercise	Occasionally	Regular	Poor
Occasionally	No Exercise	Occasionally	Regular	High Class
Following	Regular Exercise	Occasionally	Regular	Poor
Following	No Exercise	Non Alcoholic	Non Smoker	Poor
Not Following	Regular Exercise	Non Alcoholic	Occasionally	High Class
Following	No Exercise	Non Alcoholic	Regular	Middle Class
Occasionally	Regular Exercise	Non Alcoholic	Regular	Middle Class
Occasionally	Regular Exercise	Occasionally	Occasionally	High Class
Occasionally	Regular Exercise	Non Alcoholic	Regular	High Class
Occasionally	No Exercise	Non Alcoholic	Occasionally	Poor
Occasionally	No Exercise	Non Alcoholic	Regular	Poor
Occasionally	Occasionally	Non Alcoholic	Regular	Poor
Occasionally	No Exercise	Non Alcoholic	Occasionally	Middle Class
Occasionally	No Exercise	Non Alcoholic	Regular	High Class
Following	No Exercise	Occasionally	Regular	High Class
Occasionally	Occasionally	Non Alcoholic	Regular	Middle Class
Occasionally	No Exercise	Non Alcoholic	Occasionally	Middle Class
Occasionally	Regular Exercise	Non Alcoholic	Regular	Middle Class
Occasionally	No Exercise	Non Alcoholic	Regular	High Class
Not Following	No Exercise	Non Alcoholic	Regular	Poor
Occasionally	Regular Exercise	Non Alcoholic	Regular	Middle Class
Not Following	No Exercise	Non Alcoholic	Occasionally	Poor
Not Following	No Exercise	Occasionally	Non Smoker	Poor
Not Following	No Exercise	Non Alcoholic	Regular	Poor
Not Following	Occasionally	Non Alcoholic	Occasionally	Poor
Following	No Exercise	Non Alcoholic	Regular	Poor
Occasionally	Regular Exercise	Non Alcoholic	Occasionally	High Class
Occasionally	Regular Exercise	Non Alcoholic	Regular	Middle Class
Occasionally	No Exercise	Non Alcoholic	Regular	High Class

Not Following	No Exercise	Non Alcoholic	Occasionally	Middle Class
Occasionally	Regular Exercise	Non Alcoholic	Regular	High Class
Following	No Exercise	Occasionally	Regular	Poor
Not Following	Regular Exercise	Occasionally	Regular	Middle Class
Not Following	No Exercise	Non Alcoholic	Occasionally	Poor
Not Following	Regular Exercise	Non Alcoholic	Regular	Middle Class
Not Following	Occasionally	Non Alcoholic	Regular	Poor
Not Following	Regular Exercise	Occasionally	Regular	Poor
Not Following	No Exercise	Occasionally	Regular	Poor
Occasionally	Regular Exercise	Occasionally	Non Smoker	High Class
Occasionally	No Exercise	Occasionally	Regular	High Class
Not Following	Regular Exercise	Occasionally	Regular	High Class
Occasionally	Regular Exercise	Occasionally	Occasionally	High Class
Not Following	Regular Exercise	Non Alcoholic	Regular	High Class
Occasionally	Occasionally	Non Alcoholic	Occasionally	Middle Class
Not Following	Regular Exercise	Non Alcoholic	Regular	Poor
Occasionally	Regular Exercise	Non Alcoholic	Regular	High Class
Not Following	No Exercise	Non Alcoholic	Occasionally	High Class
Occasionally	No Exercise	Non Alcoholic	Regular	Middle Class
Not Following	No Exercise	Non Alcoholic	Regular	High Class
Following	Regular Exercise	Non Alcoholic	Regular	High Class
Occasionally	Regular Exercise	Non Alcoholic	Occasionally	Poor
Not Following	Regular Exercise	Non Alcoholic	Regular	Poor
Occasionally	Regular Exercise	Non Alcoholic	Regular	Middle Class
Occasionally	Occasionally	Non Alcoholic	Regular	Middle Class
Not Following	Regular Exercise	Non Alcoholic	Regular	Middle Class
Occasionally	Regular Exercise	Non Alcoholic	Non Smoker	Middle Class
Occasionally	No Exercise	Non Alcoholic	Regular	Middle Class
Not Following	No Exercise	Non Alcoholic	Regular	High Class
Following	No Exercise	Occasionally	Occasionally	High Class
Following	Occasionally	Non Alcoholic	Regular	Middle Class
Occasionally	No Exercise	Non Alcoholic	Occasionally	Middle Class
Occasionally	No Exercise	Non Alcoholic	Regular	High Class
Occasionally	Regular Exercise	Non Alcoholic	Regular	Poor
Occasionally	No Exercise	Non Alcoholic	Occasionally	Poor
Occasionally	No Exercise	Non Alcoholic	Regular	High Class
Following	No Exercise	Non Alcoholic	Regular	High Class
Occasionally	No Exercise	Non Alcoholic	Regular	Poor
Occasionally	Occasionally	Non Alcoholic	Occasionally	Middle Class
Occasionally	No Exercise	Non Alcoholic	Regular	Poor
Following	No Exercise	Non Alcoholic	Regular	Poor
Following	Occasionally	Non Alcoholic	Regular	Middle Class
Not Following	No Exercise	Non Alcoholic	Non Smoker	Poor
Following	No Exercise	Occasionally	Occasionally	Middle Class
Following	No Exercise	Non Alcoholic	Regular	Middle Class
Not Following	Regular Exercise	Non Alcoholic	Regular	Poor
Not Following	Regular Exercise	Non Alcoholic	Occasionally	Middle Class

Occasionally	Occasionally	Non Alcoholic	Regular	Poor
Occasionally	Regular Exercise	Non Alcoholic	Occasionally	Poor
Not Following	Regular Exercise	Non Alcoholic	Regular	High Class
Not Following	No Exercise	Non Alcoholic	Regular	Poor
Occasionally	Occasionally	Non Alcoholic	Occasionally	High Class
Not Following	Regular Exercise	Occasionally	Regular	High Class
Not Following	No Exercise	Non Alcoholic	Regular	High Class
Not Following	Regular Exercise	Non Alcoholic	Regular	Middle Class
Not Following	No Exercise	Non Alcoholic	Occasionally	Middle Class
Occasionally	Regular Exercise	Non Alcoholic	Regular	Middle Class
Not Following	No Exercise	Non Alcoholic	Regular	Middle Class
Occasionally	Regular Exercise	Non Alcoholic	Regular	Middle Class
Not Following	No Exercise	Non Alcoholic	Regular	Poor
Not Following	No Exercise	Non Alcoholic	Occasionally	Poor
Occasionally	No Exercise	Non Alcoholic	Regular	High Class
Following	Regular Exercise	Non Alcoholic	Regular	Poor
Following	Regular Exercise	Non Alcoholic	Occasionally	Middle Class
Following	Regular Exercise	Non Alcoholic	Non Smoker	Middle Class
Not Following	Occasionally	Occasionally	Occasionally	Poor
Occasionally	No Exercise	Non Alcoholic	Regular	Poor
Occasionally	No Exercise	Non Alcoholic	Regular	High Class
Following	Occasionally	Non Alcoholic	Occasionally	Poor
Following	No Exercise	Non Alcoholic	Regular	Poor
Following	No Exercise	Non Alcoholic	Regular	Poor
Following	Occasionally	Non Alcoholic	Regular	Middle Class
Occasionally	No Exercise	Non Alcoholic	Occasionally	High Class
Occasionally	Occasionally	Non Alcoholic	Regular	Poor
Following	No Exercise	Non Alcoholic	Regular	High Class
Not Following	Regular Exercise	Alcoholic	Regular	Middle Class
Following	Regular Exercise	Non Alcoholic	Non Smoker	Poor
Following	Regular Exercise	Non Alcoholic	Occasionally	High Class
Not Following	Regular Exercise	Occasionally	Regular	Poor
Following	No Exercise	Non Alcoholic	Regular	High Class
Not Following	Regular Exercise	Non Alcoholic	Occasionally	Poor
Following	No Exercise	Occasionally	Regular	High Class
Occasionally	Regular Exercise	Non Alcoholic	Occasionally	Poor
Following	No Exercise	Non Alcoholic	Regular	Middle Class
Occasionally	Regular Exercise	Non Alcoholic	Regular	Middle Class
Following	No Exercise	Non Alcoholic	Occasionally	Middle Class
Following	Regular Exercise	Non Alcoholic	Regular	Middle Class
Following	No Exercise	Non Alcoholic	Regular	Middle Class
Following	Regular Exercise	Non Alcoholic	Regular	Poor
Following	No Exercise	Non Alcoholic	Occasionally	High Class
Occasionally	No Exercise	Non Alcoholic	Regular	Middle Class
Occasionally	Regular Exercise	Non Alcoholic	Regular	High Class
Following	No Exercise	Non Alcoholic	Regular	Poor
Occasionally	Regular Exercise	Non Alcoholic	Regular	High Class

Following	Regular Exercise	Non Alcoholic	Occasionally	High Class
Following	Regular Exercise	Occasionally	Regular	High Class
Occasionally	No Exercise	Occasionally	Regular	Poor
Following	Regular Exercise	Occasionally	Occasionally	High Class
Occasionally	Regular Exercise	Alcoholic	Regular	Poor
Following	No Exercise	Occasionally	Occasionally	Poor
Occasionally	Regular Exercise	Occasionally	Regular	High Class
Following	No Exercise	Non Alcoholic	Regular	Poor
Following	Regular Exercise	Alcoholic	Occasionally	Poor
Following	Occasionally	Alcoholic	Regular	Middle Class
Following	Regular Exercise	Alcoholic	Regular	High Class
Occasionally	No Exercise	Non Alcoholic	Regular	Middle Class
Following	Regular Exercise	Non Alcoholic	Occasionally	Poor
Following	No Exercise	Non Alcoholic	Regular	Poor
Following	No Exercise	Non Alcoholic	Non Smoker	High Class
Occasionally	No Exercise	Non Alcoholic	Regular	High Class
Following	Regular Exercise	Non Alcoholic	Regular	High Class
Occasionally	No Exercise	Non Alcoholic	Occasionally	High Class
Following	No Exercise	Non Alcoholic	Regular	High Class
Following	No Exercise	Non Alcoholic	Regular	High Class
Occasionally	Occasionally	Occasionally	Occasionally	High Class
Occasionally	No Exercise	Non Alcoholic	Regular	Poor
Occasionally	No Exercise	Non Alcoholic	Occasionally	Poor
Occasionally	Regular Exercise	Alcoholic	Regular	Poor
Occasionally	Regular Exercise	Non Alcoholic	Regular	High Class
Occasionally	Regular Exercise	Alcoholic	Occasionally	High Class
Occasionally	No Exercise	Non Alcoholic	Regular	Poor
Occasionally	Regular Exercise	Non Alcoholic	Regular	Poor
Occasionally	No Exercise	Non Alcoholic	Regular	High Class
Occasionally	Regular Exercise	Non Alcoholic	Non Smoker	Poor
Occasionally	Regular Exercise	Non Alcoholic	Regular	High Class
Following	Regular Exercise	Non Alcoholic	Regular	Poor
Following	Regular Exercise	Non Alcoholic	Non Smoker	Poor
Occasionally	Regular Exercise	Non Alcoholic	Regular	High Class
Occasionally	No Exercise	Occasionally	Occasionally	Middle Class
Occasionally	Regular Exercise	Non Alcoholic	Regular	Poor
Occasionally	No Exercise	Non Alcoholic	Regular	High Class
Occasionally	Regular Exercise	Alcoholic	Occasionally	High Class
Following	Regular Exercise	Non Alcoholic	Regular	Poor
Occasionally	No Exercise	Alcoholic	Occasionally	High Class
Following	Regular Exercise	Occasionally	Regular	Poor
Following	No Exercise	Occasionally	Non Smoker	Poor
Following	Regular Exercise	Non Alcoholic	Occasionally	Poor
Occasionally	Regular Exercise	Alcoholic	Regular	Poor
Following	Regular Exercise	Non Alcoholic	Regular	Middle Class
Following	No Exercise	Non Alcoholic	Regular	Middle Class
Occasionally	No Exercise	Alcoholic	Occasionally	Poor

Occasionally	No Exercise	Alcoholic	Regular	High Class
Following	Regular Exercise	Non Alcoholic	Regular	High Class
Following	Regular Exercise	Non Alcoholic	Regular	Middle Class
Following	Regular Exercise	Non Alcoholic	Regular	High Class
Occasionally	No Exercise	Non Alcoholic	Occasionally	Poor
Following	No Exercise	Non Alcoholic	Regular	Poor
Following	Regular Exercise	Non Alcoholic	Regular	Poor
Following	No Exercise	Alcoholic	Occasionally	Poor
Occasionally	Regular Exercise	Occasionally	Regular	Poor
Not Following	Regular Exercise	Non Alcoholic	Non Smoker	High Class
Occasionally	Regular Exercise	Non Alcoholic	Regular	Poor
Occasionally	No Exercise	Alcoholic	Regular	Poor
Occasionally	No Exercise	Non Alcoholic	Occasionally	Middle Class
Occasionally	Occasionally	Non Alcoholic	Regular	Middle Class
Not Following	No Exercise	Alcoholic	Regular	High Class
Occasionally	Regular Exercise	Non Alcoholic	Regular	Poor
Not Following	Regular Exercise	Non Alcoholic	Occasionally	Middle Class
Not Following	Regular Exercise	Occasionally	Regular	High Class
Occasionally	No Exercise	Non Alcoholic	Regular	Poor
Occasionally	Occasionally	Occasionally	Regular	High Class
Not Following	Regular Exercise	Occasionally	Regular	Middle Class
Occasionally	Regular Exercise	Non Alcoholic	Occasionally	Middle Class
Occasionally	No Exercise	Non Alcoholic	Regular	Middle Class
Occasionally	Regular Exercise	Non Alcoholic	Regular	High Class
Not Following	No Exercise	Non Alcoholic	Occasionally	Poor
Not Following	Regular Exercise	Non Alcoholic	Regular	High Class
Not Following	Regular Exercise	Non Alcoholic	Occasionally	High Class
Occasionally	No Exercise	Non Alcoholic	Regular	Middle Class
Occasionally	Regular Exercise	Non Alcoholic	Regular	High Class
Occasionally	No Exercise	Non Alcoholic	Occasionally	High Class
Occasionally	Regular Exercise	Non Alcoholic	Regular	High Class
Occasionally	No Exercise	Non Alcoholic	Regular	Middle Class
Not Following	No Exercise	Non Alcoholic	Regular	High Class
Occasionally	Occasionally	Non Alcoholic	Occasionally	Poor
Not Following	No Exercise	Non Alcoholic	Regular	Poor
Not Following	Regular Exercise	Non Alcoholic	Non Smoker	Poor
Occasionally	Regular Exercise	Non Alcoholic	Regular	High Class
Occasionally	Regular Exercise	Non Alcoholic	Regular	Poor
Following	Regular Exercise	Non Alcoholic	Occasionally	Poor
Occasionally	No Exercise	Non Alcoholic	Regular	High Class
Not Following	No Exercise	Alcoholic	Non Smoker	High Class
Following	Regular Exercise	Alcoholic	Occasionally	Middle Class
Following	No Exercise	Alcoholic	Regular	High Class
Not Following	Regular Exercise	Occasionally	Occasionally	High Class
Following	No Exercise	Non Alcoholic	Regular	Poor
Not Following	Regular Exercise	Non Alcoholic	Regular	High Class
Not Following	Regular Exercise	Occasionally	Occasionally	Middle Class

Following	Regular Exercise	Non Alcoholic	Regular	High Class
Not Following	No Exercise	Non Alcoholic	Regular	High Class
Following	Regular Exercise	Occasionally	Regular	Poor
Following	No Exercise	Non Alcoholic	Occasionally	High Class
Not Following	Regular Exercise	Non Alcoholic	Regular	High Class
Not Following	Regular Exercise	Non Alcoholic	Regular	High Class
Not Following	Regular Exercise	Non Alcoholic	Regular	High Class
Not Following	Regular Exercise	Non Alcoholic	Regular	High Class
Following	No Exercise	Non Alcoholic	Occasionally	Middle Class
Following	Regular Exercise	Non Alcoholic	Regular	Middle Class
Not Following	No Exercise	Non Alcoholic	Occasionally	High Class
Not Following	Regular Exercise	Non Alcoholic	Regular	High Class
Not Following	Regular Exercise	Non Alcoholic	Regular	High Class
Not Following	Regular Exercise	Alcoholic	Occasionally	High Class
Not Following	Regular Exercise	Non Alcoholic	Regular	Poor
Not Following	Regular Exercise	Occasionally	Regular	High Class
Occasionally	No Exercise	Occasionally	Regular	Middle Class
Occasionally	Regular Exercise	Non Alcoholic	Occasionally	Poor
Occasionally	Regular Exercise	Non Alcoholic	Non Smoker	Poor
Not Following	Regular Exercise	Non Alcoholic	Regular	Poor
Not Following	No Exercise	Non Alcoholic	Regular	Poor
Not Following	Regular Exercise	Non Alcoholic	Regular	Middle Class
Not Following	Regular Exercise	Non Alcoholic	Occasionally	Poor
Occasionally	Regular Exercise	Non Alcoholic	Regular	High Class
Occasionally	Occasionally	Non Alcoholic	Regular	High Class
Occasionally	Regular Exercise	Occasionally	Non Smoker	Middle Class
Occasionally	Regular Exercise	Non Alcoholic	Regular	Middle Class
Occasionally	Occasionally	Non Alcoholic	Occasionally	Middle Class
Occasionally	Regular Exercise	Non Alcoholic	Regular	Middle Class
Not Following	Regular Exercise	Non Alcoholic	Regular	Middle Class
Not Following	No Exercise	Non Alcoholic	Occasionally	High Class
Occasionally	No Exercise	Alcoholic	Regular	Middle Class
Not Following	Regular Exercise	Non Alcoholic	Regular	Poor
Not Following	Regular Exercise	Non Alcoholic	Regular	High Class
Occasionally	Regular Exercise	Non Alcoholic	Occasionally	Poor
Not Following	No Exercise	Non Alcoholic	Regular	Middle Class
Not Following	No Exercise	Non Alcoholic	Regular	High Class
Occasionally	Regular Exercise	Non Alcoholic	Regular	Middle Class
Occasionally	Regular Exercise	Occasionally	Regular	Poor
Occasionally	No Exercise	Non Alcoholic	Occasionally	Poor
Occasionally	Regular Exercise	Non Alcoholic	Regular	Middle Class
Occasionally	No Exercise	Non Alcoholic	Regular	Middle Class
Occasionally	Regular Exercise	Occasionally	Occasionally	Middle Class
Not Following	No Exercise	Alcoholic	Regular	High Class
Not Following	Regular Exercise	Alcoholic	Occasionally	High Class
Not Following	No Exercise	Non Alcoholic	Regular	High Class
Occasionally	Regular Exercise	Non Alcoholic	Regular	Middle Class

Occasionally	No Exercise	Alcoholic	Occasionally	Middle Class
Occasionally	Regular Exercise	Occasionally	Regular	Middle Class
Not Following	No Exercise	Occasionally	Regular	Middle Class
Occasionally	Regular Exercise	Non Alcoholic	Non Smoker	Poor
Occasionally	No Exercise	Occasionally	Occasionally	Poor
Occasionally	Occasionally	Occasionally	Regular	Poor
Occasionally	Regular Exercise	Non Alcoholic	Regular	High Class
Not Following	Regular Exercise	Non Alcoholic	Regular	High Class
Not Following	Occasionally	Non Alcoholic	Regular	High Class
Not Following	Regular Exercise	Non Alcoholic	Occasionally	High Class
Not Following	Regular Exercise	Non Alcoholic	Regular	Middle Class
Not Following	Regular Exercise	Occasionally	Regular	High Class
Not Following	Regular Exercise	Non Alcoholic	Occasionally	Middle Class
Not Following	No Exercise	Non Alcoholic	Regular	High Class
Not Following	Regular Exercise	Non Alcoholic	Occasionally	Middle Class
Not Following	Occasionally	Non Alcoholic	Regular	High Class
Not Following	Regular Exercise	Non Alcoholic	Regular	Poor
Not Following	No Exercise	Non Alcoholic	Occasionally	Poor
Not Following	Regular Exercise	Non Alcoholic	Regular	Middle Class
Not Following	Regular Exercise	Non Alcoholic	Regular	Middle Class
Not Following	Occasionally	Occasionally	Regular	Middle Class
Not Following	Regular Exercise	Non Alcoholic	Non Smoker	Middle Class
Not Following	Regular Exercise	Alcoholic	Regular	High Class
Not Following	Regular Exercise	Non Alcoholic	Regular	High Class
Not Following	No Exercise	Non Alcoholic	Regular	High Class
Not Following	Regular Exercise	Non Alcoholic	Regular	High Class
Not Following	Occasionally	Non Alcoholic	Occasionally	High Class
Not Following	Regular Exercise	Non Alcoholic	Regular	Middle Class
Occasionally	Occasionally	Alcoholic	Occasionally	Poor
Not Following	Occasionally	Non Alcoholic	Occasionally	Middle Class
Not Following	No Exercise	Occasionally	Regular	Middle Class
Following	Regular Exercise	Occasionally	Occasionally	High Class
Following	Regular Exercise	Occasionally	Occasionally	Poor
Following	Regular Exercise	Non Alcoholic	Regular	Middle Class
Not Following	Occasionally	Occasionally	Occasionally	High Class
Occasionally	Regular Exercise	Occasionally	Regular	Middle Class
Not Following	Occasionally	Non Alcoholic	Regular	High Class
Not Following	No Exercise	Non Alcoholic	Non Smoker	Middle Class
Following	Regular Exercise	Non Alcoholic	Non Smoker	High Class
Following	Regular Exercise	Non Alcoholic	Occasionally	Middle Class
Following	Regular Exercise	Non Alcoholic	Non Smoker	High Class
Following	Regular Exercise	Non Alcoholic	Occasionally	Poor
Following	Regular Exercise	Occasionally	Occasionally	Poor
Occasionally	No Exercise	Non Alcoholic	Regular	Poor
Occasionally	No Exercise	Non Alcoholic	Regular	Poor
Occasionally	No Exercise	Non Alcoholic	Regular	Middle Class
Following	Occasionally	Alcoholic	Regular	Middle Class

Following	No Exercise	Non Alcoholic	Regular	High Class
Occasionally	Regular Exercise	Occasionally	Regular	High Class
Occasionally	Occasionally	Alcoholic	Regular	High Class
Occasionally	Occasionally	Occasionally	Regular	Middle Class
Occasionally	Regular Exercise	Non Alcoholic	Occasionally	Middle Class
Following	Occasionally	Alcoholic	Regular	Middle Class
Occasionally	Regular Exercise	Occasionally	Regular	High Class
Occasionally	Regular Exercise	Non Alcoholic	Occasionally	High Class
Occasionally	Regular Exercise	Alcoholic	Regular	Poor
Occasionally	Regular Exercise	Non Alcoholic	Occasionally	Poor
Occasionally	Occasionally	Occasionally	Occasionally	Poor
Occasionally	No Exercise	Non Alcoholic	Non Smoker	High Class
Occasionally	Regular Exercise	Non Alcoholic	Occasionally	High Class
Following	Regular Exercise	Occasionally	Regular	High Class
Following	No Exercise	Non Alcoholic	Regular	Middle Class
Occasionally	No Exercise	Alcoholic	Regular	Middle Class
Following	Regular Exercise	Non Alcoholic	Occasionally	Middle Class
Following	Regular Exercise	Non Alcoholic	Non Smoker	Middle Class
Following	Regular Exercise	Non Alcoholic	Non Smoker	Poor
Occasionally	No Exercise	Alcoholic	Regular	Poor
Following	Regular Exercise	Non Alcoholic	Regular	High Class
Occasionally	No Exercise	Non Alcoholic	Occasionally	High Class
Occasionally	No Exercise	Non Alcoholic	Regular	High Class
Occasionally	Regular Exercise	Occasionally	Regular	Middle Class
Occasionally	Regular Exercise	Non Alcoholic	Occasionally	Middle Class
Following	Regular Exercise	Non Alcoholic	Non Smoker	Middle Class
Occasionally	No Exercise	Non Alcoholic	Non Smoker	Middle Class
Occasionally	No Exercise	Non Alcoholic	Non Smoker	Middle Class
Occasionally	Occasionally	Alcoholic	Regular	Poor
Following	Occasionally	Non Alcoholic	Non Smoker	Poor
Occasionally	No Exercise	Alcoholic	Regular	Poor
Following	Regular Exercise	Alcoholic	Regular	Poor
Occasionally	Occasionally	Occasionally	Occasionally	Poor
Occasionally	No Exercise	Alcoholic	Regular	Middle Class
Not Following	Regular Exercise	Alcoholic	Occasionally	Middle Class
Not Following	Regular Exercise	Non Alcoholic	Non Smoker	Middle Class
Occasionally	No Exercise	Occasionally	Non Smoker	Middle Class
Occasionally	No Exercise	Alcoholic	Regular	Middle Class
Occasionally	Regular Exercise	Occasionally	Regular	Middle Class
Not Following	No Exercise	Occasionally	Non Smoker	High Class
Not Following	No Exercise	Non Alcoholic	Non Smoker	High Class
Not Following	No Exercise	Alcoholic	Occasionally	High Class
Occasionally	Occasionally	Occasionally	Occasionally	Poor
Following	Regular Exercise	Non Alcoholic	Occasionally	High Class
Occasionally	Regular Exercise	Alcoholic	Regular	Poor
Occasionally	Regular Exercise	Non Alcoholic	Non Smoker	Poor
Occasionally	Regular Exercise	Non Alcoholic	Occasionally	Middle Class

Occasionally	Regular Exercise	Occasionally	Regular	High Class
Not Following	Occasionally	Non Alcoholic	Occasionally	High Class
Not Following	Regular Exercise	Non Alcoholic	Regular	Middle Class
Following	Regular Exercise	Non Alcoholic	Non Smoker	High Class
Not Following	Regular Exercise	Alcoholic	Regular	High Class
Following	Regular Exercise	Non Alcoholic	Non Smoker	High Class
Not Following	Regular Exercise	Non Alcoholic	Non Smoker	Middle Class
Occasionally	Occasionally	Occasionally	Occasionally	High Class
Occasionally	Regular Exercise	Alcoholic	Regular	Poor
Not Following	Regular Exercise	Occasionally	Non Smoker	High Class
Occasionally	Regular Exercise	Alcoholic	Regular	Poor
Following	Regular Exercise	Non Alcoholic	Regular	Poor
Following	Regular Exercise	Non Alcoholic	Occasionally	High Class
Following	Regular Exercise	Alcoholic	Regular	Poor
Following	Regular Exercise	Non Alcoholic	Regular	Poor
Not Following	Regular Exercise	Non Alcoholic	Occasionally	High Class
Following	Regular Exercise	Occasionally	Regular	Middle Class
Not Following	Regular Exercise	Occasionally	Regular	High Class
Not Following	Regular Exercise	Non Alcoholic	Regular	Middle Class
Following	Regular Exercise	Non Alcoholic	Regular	Middle Class
Occasionally	Occasionally	Alcoholic	Occasionally	High Class
Occasionally	Regular Exercise	Non Alcoholic	Non Smoker	High Class
Following	Regular Exercise	Occasionally	Regular	Poor
Following	Regular Exercise	Non Alcoholic	Non Smoker	Poor
Following	Occasionally	Occasionally	Occasionally	Poor
Occasionally	Regular Exercise	Occasionally	Regular	Middle Class
Occasionally	Occasionally	Alcoholic	Occasionally	High Class
Occasionally	No Exercise	Non Alcoholic	Non Smoker	High Class
Following	Regular Exercise	Non Alcoholic	Regular	High Class
Not Following	No Exercise	Alcoholic	Occasionally	Poor
Not Following	Occasionally	Occasionally	Regular	Poor
Occasionally	Occasionally	Non Alcoholic	Non Smoker	Poor
Not Following	Regular Exercise	Alcoholic	Non Smoker	High Class
Not Following	Regular Exercise	Occasionally	Non Smoker	Poor
Occasionally	Occasionally	Occasionally	Occasionally	Poor
Following	Regular Exercise	Alcoholic	Regular	High Class
Following	Regular Exercise	Non Alcoholic	Occasionally	Middle Class
Occasionally	No Exercise	Alcoholic	Non Smoker	Middle Class
Following	No Exercise	Alcoholic	Regular	High Class
Not Following	Occasionally	Occasionally	Occasionally	High Class
Occasionally	Occasionally	Non Alcoholic	Regular	Poor
Occasionally	Occasionally	Non Alcoholic	Occasionally	Poor
Following	Regular Exercise	Alcoholic	Regular	Poor
Following	No Exercise	Occasionally	Non Smoker	Middle Class
Following	Regular Exercise	Alcoholic	Occasionally	High Class
Not Following	Occasionally	Occasionally	Regular	High Class
Occasionally	No Exercise	Non Alcoholic	Regular	Middle Class

Occasionally	Occasionally	Occasionally	Regular	Middle Class
Following	Regular Exercise	Alcoholic	Regular	Middle Class
Following	No Exercise	Alcoholic	Non Smoker	High Class
Following	Occasionally	Occasionally	Occasionally	Poor
Following	Occasionally	Occasionally	Non Smoker	Middle Class
Occasionally	Occasionally	Occasionally	Occasionally	Poor
Following	Occasionally	Alcoholic	Regular	Poor
Following	Regular Exercise	Non Alcoholic	Occasionally	Poor
Occasionally	Regular Exercise	Alcoholic	Occasionally	Poor
Occasionally	Regular Exercise	Non Alcoholic	Non Smoker	Poor
Occasionally	Regular Exercise	Alcoholic	Occasionally	High Class
Following	Regular Exercise	Occasionally	Regular	Middle Class
Following	Regular Exercise	Non Alcoholic	Non Smoker	High Class
Following	Occasionally	Alcoholic	Occasionally	Middle Class
Occasionally	Regular Exercise	Occasionally	Non Smoker	High Class
Not Following	No Exercise	Occasionally	Regular	Poor
Following	Occasionally	Occasionally	Occasionally	Middle Class
Not Following	Regular Exercise	Occasionally	Non Smoker	Poor
Not Following	Regular Exercise	Occasionally	Occasionally	Middle Class
Following	Regular Exercise	Non Alcoholic	Non Smoker	Poor
Following	Regular Exercise	Non Alcoholic	Occasionally	Poor
Following	Regular Exercise	Occasionally	Occasionally	Poor
Occasionally	Occasionally	Occasionally	Non Smoker	High Class
Following	Regular Exercise	Alcoholic	Regular	High Class
Occasionally	Regular Exercise	Occasionally	Occasionally	High Class
Not Following	Occasionally	Alcoholic	Regular	High Class
Following	Occasionally	Occasionally	Occasionally	High Class
Following	Occasionally	Non Alcoholic	Regular	Middle Class
Following	Regular Exercise	Alcoholic	Occasionally	Poor
Following	Regular Exercise	Non Alcoholic	Non Smoker	High Class
Not Following	Regular Exercise	Occasionally	Regular	High Class
Following	Occasionally	Occasionally	Regular	Middle Class
Following	Occasionally	Non Alcoholic	Occasionally	High Class
Following	Regular Exercise	Alcoholic	Regular	High Class
Occasionally	Occasionally	Occasionally	Occasionally	Poor
Not Following	Regular Exercise	Alcoholic	Regular	Poor
Occasionally	No Exercise	Occasionally	Regular	Middle Class
Following	Occasionally	Alcoholic	Regular	Middle Class
Not Following	Regular Exercise	Alcoholic	Regular	Middle Class
Occasionally	No Exercise	Non Alcoholic	Regular	Middle Class
Occasionally	Regular Exercise	Non Alcoholic	Regular	Middle Class
Occasionally	Regular Exercise	Non Alcoholic	Regular	High Class
Following	No Exercise	Alcoholic	Occasionally	High Class
Not Following	Occasionally	Alcoholic	Regular	Middle Class
Following	Regular Exercise	Non Alcoholic	Occasionally	Middle Class
Following	Regular Exercise	Non Alcoholic	Non Smoker	High Class
Following	Regular Exercise	Non Alcoholic	Non Smoker	Poor

Occasionally	Regular Exercise	Occasionally	Regular	Poor
Following	Occasionally	Occasionally	Regular	High Class
Following	Regular Exercise	Non Alcoholic	Non Smoker	High Class
Occasionally	Occasionally	Occasionally	Occasionally	Poor
Following	Regular Exercise	Non Alcoholic	Non Smoker	Middle Class
Not Following	No Exercise	Alcoholic	Non Smoker	Poor
Following	Regular Exercise	Alcoholic	Regular	Poor
Not Following	Regular Exercise	Occasionally	Regular	Middle Class
Not Following	Regular Exercise	Occasionally	Regular	Poor
Not Following	Occasionally	Occasionally	Occasionally	Middle Class
Following	Regular Exercise	Alcoholic	Regular	Middle Class
Following	No Exercise	Occasionally	Occasionally	Poor
Following	Regular Exercise	Occasionally	Regular	Middle Class
Following	Regular Exercise	Occasionally	Non Smoker	Poor
Following	Occasionally	Non Alcoholic	Non Smoker	Poor
Following	Regular Exercise	Occasionally	Occasionally	High Class
Following	Regular Exercise	Non Alcoholic	Regular	Poor
Occasionally	Occasionally	Alcoholic	Regular	High Class
Not Following	No Exercise	Non Alcoholic	Regular	High Class
Occasionally	Occasionally	Non Alcoholic	Non Smoker	High Class
Not Following	Regular Exercise	Occasionally	Occasionally	Middle Class
Following	Regular Exercise	Occasionally	Regular	Middle Class
Occasionally	Occasionally	Occasionally	Regular	Middle Class
Not Following	No Exercise	Non Alcoholic	Non Smoker	Middle Class
Occasionally	Regular Exercise	Non Alcoholic	Occasionally	Middle Class
Following	Regular Exercise	Non Alcoholic	Non Smoker	Poor
Occasionally	Occasionally	Occasionally	Occasionally	Poor
Occasionally	Occasionally	Occasionally	Occasionally	High Class
Following	No Exercise	Occasionally	Regular	Poor
Not Following	No Exercise	Alcoholic	Regular	Middle Class
Not Following	Regular Exercise	Occasionally	Regular	Middle Class
Following	Regular Exercise	Non Alcoholic	Regular	Poor
Not Following	No Exercise	Occasionally	Occasionally	Poor
Following	Regular Exercise	Non Alcoholic	Non Smoker	High Class
Following	Regular Exercise	Alcoholic	Non Smoker	Poor
Not Following	Occasionally	Alcoholic	Regular	Poor
Not Following	Regular Exercise	Occasionally	Non Smoker	Poor
Following	Regular Exercise	Occasionally	Non Smoker	Middle Class
Following	Regular Exercise	Non Alcoholic	Regular	High Class
Following	Occasionally	Occasionally	Non Smoker	Poor
Not Following	No Exercise	Alcoholic	Occasionally	High Class
Following	Occasionally	Alcoholic	Regular	Middle Class
Not Following	Regular Exercise	Alcoholic	Regular	Poor
Following	Regular Exercise	Non Alcoholic	Regular	High Class
Following	No Exercise	Non Alcoholic	Occasionally	Poor
Occasionally	No Exercise	Non Alcoholic	Non Smoker	High Class
Occasionally	Regular Exercise	Alcoholic	Non Smoker	Poor

Not Following	Occasionally	Occasionally	Regular	High Class
Not Following	Occasionally	Non Alcoholic	Regular	Poor
Occasionally	Regular Exercise	Non Alcoholic	Regular	Middle Class
Not Following	No Exercise	Alcoholic	Regular	Middle Class
Occasionally	Regular Exercise	Occasionally	Occasionally	Middle Class
Occasionally	Regular Exercise	Non Alcoholic	Non Smoker	Middle Class
Following	Regular Exercise	Non Alcoholic	Non Smoker	Middle Class
Occasionally	No Exercise	Occasionally	Non Smoker	Poor
Occasionally	Regular Exercise	Alcoholic	Regular	High Class
Following	Regular Exercise	Non Alcoholic	Occasionally	Middle Class
Following	Occasionally	Occasionally	Occasionally	High Class
Following	Regular Exercise	Non Alcoholic	Occasionally	Poor
Following	No Exercise	Alcoholic	Occasionally	High Class
Occasionally	No Exercise	Alcoholic	Occasionally	High Class
Occasionally	No Exercise	Alcoholic	Regular	High Class
Not Following	Occasionally	Non Alcoholic	Occasionally	Poor
Occasionally	Occasionally	Alcoholic	Regular	High Class
Not Following	No Exercise	Alcoholic	Regular	Poor
Following	Occasionally	Non Alcoholic	Regular	Poor
Not Following	Occasionally	Alcoholic	Regular	High Class
Not Following	Occasionally	Non Alcoholic	Regular	Poor
Not Following	No Exercise	Occasionally	Regular	Poor
Following	Occasionally	Non Alcoholic	Regular	Middle Class
Occasionally	No Exercise	Alcoholic	Non Smoker	High Class
Occasionally	Occasionally	Alcoholic	Regular	Middle Class
Occasionally	Occasionally	Occasionally	Occasionally	Poor
Occasionally	No Exercise	Occasionally	Regular	Poor
Not Following	Occasionally	Non Alcoholic	Regular	High Class
Following	Regular Exercise	Alcoholic	Occasionally	High Class
Following	Regular Exercise	Occasionally	Occasionally	High Class
Following	No Exercise	Alcoholic	Occasionally	High Class
Following	Occasionally	Non Alcoholic	Occasionally	High Class
Occasionally	Occasionally	Occasionally	Occasionally	High Class
Following	Occasionally	Occasionally	Regular	High Class
Not Following	Occasionally	Alcoholic	Regular	Poor
Following	Regular Exercise	Occasionally	Occasionally	Poor
Occasionally	No Exercise	Non Alcoholic	Non Smoker	Poor
Occasionally	Regular Exercise	Non Alcoholic	Regular	High Class
Occasionally	No Exercise	Alcoholic	Regular	High Class
Occasionally	Occasionally	Occasionally	Occasionally	Poor
Following	Occasionally	Occasionally	Regular	Poor
Not Following	Occasionally	Non Alcoholic	Regular	High Class
Following	Occasionally	Occasionally	Regular	Poor
Not Following	Occasionally	Occasionally	Occasionally	High Class
Not Following	Occasionally	Alcoholic	Occasionally	Poor
Occasionally	Occasionally	Non Alcoholic	Non Smoker	Poor
Occasionally	Regular Exercise	Occasionally	Non Smoker	High Class

Not Following	Occasionally	Occasionally	Occasionally	Middle Class
Following	Regular Exercise	Non Alcoholic	Non Smoker	Poor
Not Following	Regular Exercise	Alcoholic	Regular	High Class
Not Following	Regular Exercise	Occasionally	Occasionally	High Class
Occasionally	Occasionally	Occasionally	Non Smoker	Poor
Following	Occasionally	Non Alcoholic	Non Smoker	High Class
Not Following	Occasionally	Non Alcoholic	Regular	Poor
Following	Occasionally	Non Alcoholic	Non Smoker	Poor
Following	Occasionally	Non Alcoholic	Regular	Poor
Following	Occasionally	Alcoholic	Regular	Poor
Following	Regular Exercise	Alcoholic	Regular	Middle Class
Following	Regular Exercise	Non Alcoholic	Non Smoker	Middle Class
Following	Regular Exercise	Non Alcoholic	Occasionally	Poor
Following	Occasionally	Alcoholic	Regular	High Class
Following	Regular Exercise	Non Alcoholic	Non Smoker	High Class
Occasionally	Occasionally	Alcoholic	Occasionally	Middle Class
Occasionally	Occasionally	Non Alcoholic	Non Smoker	High Class
Following	Regular Exercise	Occasionally	Regular	Poor
Occasionally	Regular Exercise	Occasionally	Occasionally	Poor
Following	Occasionally	Occasionally	Non Smoker	Poor
Occasionally	Occasionally	Occasionally	Non Smoker	Poor
Following	Regular Exercise	Non Alcoholic	Regular	Poor
Following	No Exercise	Occasionally	Occasionally	High Class
Not Following	Occasionally	Non Alcoholic	Occasionally	Poor
Not Following	Regular Exercise	Alcoholic	Regular	Poor
Following	Regular Exercise	Occasionally	Regular	Middle Class
Not Following	No Exercise	Alcoholic	Regular	Middle Class
Following	Regular Exercise	Non Alcoholic	Non Smoker	High Class
Following	Regular Exercise	Alcoholic	Occasionally	Poor
Following	Regular Exercise	Non Alcoholic	Regular	Middle Class
Following	Regular Exercise	Non Alcoholic	Non Smoker	High Class
Not Following	No Exercise	Non Alcoholic	Occasionally	Poor
Following	Regular Exercise	Non Alcoholic	Occasionally	High Class
Following	No Exercise	Non Alcoholic	Regular	Middle Class
Following	No Exercise	Alcoholic	Regular	Middle Class
Occasionally	Occasionally	Occasionally	Occasionally	Middle Class
Occasionally	Occasionally	Non Alcoholic	Occasionally	High Class
Occasionally	Regular Exercise	Non Alcoholic	Occasionally	Poor
Not Following	No Exercise	Occasionally	Occasionally	High Class
Occasionally	Regular Exercise	Non Alcoholic	Non Smoker	High Class
Following	Regular Exercise	Non Alcoholic	Non Smoker	Middle Class
Following	Occasionally	Occasionally	Regular	High Class
Not Following	No Exercise	Non Alcoholic	Occasionally	High Class
Occasionally	No Exercise	Alcoholic	Non Smoker	High Class
Following	Regular Exercise	Non Alcoholic	Occasionally	Middle Class
Following	No Exercise	Occasionally	Regular	High Class
Following	Regular Exercise	Non Alcoholic	Occasionally	Poor

Following	No Exercise	Occasionally	Regular	Poor
Following	Regular Exercise	Non Alcoholic	Regular	Poor
Following	No Exercise	Non Alcoholic	Regular	High Class
Following	Regular Exercise	Non Alcoholic	Regular	Poor
Occasionally	Regular Exercise	Non Alcoholic	Occasionally	Poor
Occasionally	No Exercise	Non Alcoholic	Regular	High Class
Following	Regular Exercise	Non Alcoholic	Regular	High Class
Following	No Exercise	Non Alcoholic	Occasionally	Middle Class
Occasionally	Regular Exercise	Non Alcoholic	Regular	High Class
Occasionally	No Exercise	Non Alcoholic	Occasionally	High Class
Occasionally	Regular Exercise	Non Alcoholic	Regular	Poor
Occasionally	Regular Exercise	Non Alcoholic	Regular	High Class
Occasionally	No Exercise	Non Alcoholic	Occasionally	Middle Class
Occasionally	Regular Exercise	Non Alcoholic	Regular	High Class
Following	No Exercise	Non Alcoholic	Regular	High Class
Following	Regular Exercise	Non Alcoholic	Regular	Poor
Following	No Exercise	Non Alcoholic	Occasionally	High Class
Following	Regular Exercise	Non Alcoholic	Regular	High Class
Following	Regular Exercise	Non Alcoholic	Regular	High Class
Following	Regular Exercise	Non Alcoholic	Regular	High Class
Following	Regular Exercise	Non Alcoholic	Regular	High Class
Following	No Exercise	Non Alcoholic	Occasionally	Middle Class
Following	Regular Exercise	Non Alcoholic	Regular	Middle Class
Following	Regular Exercise	Non Alcoholic	Regular	Middle Class
Following	No Exercise	Occasionally	Occasionally	Middle Class
Following	Regular Exercise	Occasionally	Non Smoker	Middle Class
Following	No Exercise	Non Alcoholic	Occasionally	Middle Class
Following	Regular Exercise	Non Alcoholic	Regular	Poor
Following	Regular Exercise	Non Alcoholic	Regular	Poor
Following	No Exercise	Non Alcoholic	Occasionally	Poor
Following	Regular Exercise	Non Alcoholic	Regular	Poor
Following	Regular Exercise	Non Alcoholic	Regular	High Class
Following	No Exercise	Non Alcoholic	Regular	High Class
Following	Regular Exercise	Occasionally	Occasionally	High Class
Following	No Exercise	Non Alcoholic	Non Smoker	Poor
Following	Regular Exercise	Non Alcoholic	Regular	High Class
Following	Regular Exercise	Non Alcoholic	Regular	Poor
Following	No Exercise	Occasionally	Regular	Middle Class
Following	Regular Exercise	Non Alcoholic	Occasionally	Middle Class
Following	No Exercise	Non Alcoholic	Regular	Middle Class
Following	Regular Exercise	Non Alcoholic	Regular	Middle Class
Following	Regular Exercise	Non Alcoholic	Occasionally	Middle Class
Following	Regular Exercise	Occasionally	Regular	Middle Class
Following	No Exercise	Non Alcoholic	Occasionally	High Class
Following	Regular Exercise	Non Alcoholic	Regular	High Class
Following	Regular Exercise	Non Alcoholic	Regular	High Class
Following	No Exercise	Non Alcoholic	Occasionally	High Class

Following	Regular Exercise	Non Alcoholic	Regular	Poor
Following	Regular Exercise	Non Alcoholic	Regular	High Class
Following	No Exercise	Non Alcoholic	Regular	Middle Class
Following	Regular Exercise	Non Alcoholic	Occasionally	Poor
Following	No Exercise	Non Alcoholic	Regular	Poor
Following	Regular Exercise	Non Alcoholic	Regular	Poor
Following	Regular Exercise	Non Alcoholic	Regular	Poor
Following	Regular Exercise	Occasionally	Regular	Middle Class
Following	No Exercise	Non Alcoholic	Occasionally	Poor
Occasionally	Regular Exercise	Non Alcoholic	Regular	High Class
Occasionally	No Exercise	Non Alcoholic	Regular	High Class
Occasionally	Regular Exercise	Occasionally	Occasionally	Middle Class
Following	No Exercise	Non Alcoholic	Non Smoker	Middle Class
Following	Regular Exercise	Non Alcoholic	Occasionally	Middle Class
Following	Occasionally	Non Alcoholic	Regular	Middle Class
Occasionally	Regular Exercise	Non Alcoholic	Regular	Middle Class
Following	No Exercise	Occasionally	Occasionally	High Class
Occasionally	Regular Exercise	Non Alcoholic	Regular	Middle Class
Following	Regular Exercise	Non Alcoholic	Regular	Poor
Occasionally	No Exercise	Non Alcoholic	Regular	High Class
Following	Occasionally	Non Alcoholic	Occasionally	Poor
Following	Regular Exercise	Non Alcoholic	Non Smoker	Middle Class
Following	No Exercise	Alcoholic	Regular	High Class
Following	Regular Exercise	Occasionally	Regular	Middle Class
Following	No Exercise	Non Alcoholic	Regular	Poor
Following	Regular Exercise	Non Alcoholic	Occasionally	Poor
Following	No Exercise	Non Alcoholic	Regular	Middle Class
Following	Regular Exercise	Occasionally	Regular	Middle Class
Following	No Exercise	Non Alcoholic	Occasionally	Middle Class
Following	Regular Exercise	Non Alcoholic	Regular	High Class
Following	No Exercise	Non Alcoholic	Occasionally	High Class
Following	Occasionally	Non Alcoholic	Regular	High Class
Following	Regular Exercise	Non Alcoholic	Regular	Middle Class
Following	No Exercise	Non Alcoholic	Occasionally	Middle Class
Following	No Exercise	Non Alcoholic	Regular	Middle Class
Following	Regular Exercise	Occasionally	Regular	Middle Class
Following	Regular Exercise	Non Alcoholic	Regular	Poor
Following	No Exercise	Non Alcoholic	Non Smoker	Poor
Following	No Exercise	Non Alcoholic	Regular	Poor
Following	No Exercise	Non Alcoholic	Regular	High Class
Following	No Exercise	Non Alcoholic	Regular	High Class
Following	Regular Exercise	Non Alcoholic	Regular	High Class
Following	No Exercise	Non Alcoholic	Occasionally	High Class
Following	Regular Exercise	Non Alcoholic	Regular	Middle Class
Following	Occasionally	Occasionally	Regular	High Class
Following	No Exercise	Non Alcoholic	Occasionally	Middle Class
Following	No Exercise	Non Alcoholic	Non Smoker	High Class

Following	No Exercise	Non Alcoholic	Occasionally	Middle Class
Occasionally	Regular Exercise	Non Alcoholic	Regular	High Class
Following	No Exercise	Non Alcoholic	Regular	Poor
Following	Regular Exercise	Non Alcoholic	Occasionally	Poor
Following	No Exercise	Non Alcoholic	Regular	Middle Class
Following	Regular Exercise	Non Alcoholic	Regular	Middle Class
Following	No Exercise	Alcoholic	Regular	Middle Class
Following	Regular Exercise	Non Alcoholic	Occasionally	Middle Class
Following	No Exercise	Non Alcoholic	Regular	High Class
Following	Occasionally	Non Alcoholic	Regular	High Class
Occasionally	Regular Exercise	Non Alcoholic	Regular	Poor
Occasionally	Regular Exercise	Non Alcoholic	Regular	Poor
Following	Regular Exercise	Non Alcoholic	Occasionally	Middle Class
Occasionally	Regular Exercise	Non Alcoholic	Regular	Middle Class
Following	Regular Exercise	Non Alcoholic	Regular	Middle Class
Following	No Exercise	Non Alcoholic	Non Smoker	Middle Class
Following	Regular Exercise	Non Alcoholic	Regular	Middle Class
Following	Regular Exercise	Occasionally	Occasionally	High Class
Following	Regular Exercise	Non Alcoholic	Regular	Middle Class
Occasionally	Regular Exercise	Non Alcoholic	Regular	High Class
Occasionally	Regular Exercise	Non Alcoholic	Occasionally	High Class
Occasionally	Regular Exercise	Non Alcoholic	Regular	High Class
Occasionally	Regular Exercise	Non Alcoholic	Regular	High Class
Occasionally	Regular Exercise	Non Alcoholic	Regular	High Class
Following	No Exercise	Alcoholic	Non Smoker	High Class
Following	No Exercise	Non Alcoholic	Regular	Poor
Following	No Exercise	Alcoholic	Regular	Poor
Following	Regular Exercise	Non Alcoholic	Regular	Poor
Following	No Exercise	Non Alcoholic	Regular	Middle Class
Following	Regular Exercise	Non Alcoholic	Occasionally	High Class
Following	Regular Exercise	Non Alcoholic	Regular	Poor
Following	Regular Exercise	Non Alcoholic	Regular	Poor
Following	No Exercise	Non Alcoholic	Occasionally	Poor
Following	No Exercise	Non Alcoholic	Regular	Poor
Following	Regular Exercise	Alcoholic	Occasionally	Poor
Following	No Exercise	Non Alcoholic	Regular	Poor
Following	No Exercise	Non Alcoholic	Regular	Poor
Following	Regular Exercise	Non Alcoholic	Occasionally	Poor
Following	Regular Exercise	Non Alcoholic	Regular	Middle Class
Following	No Exercise	Non Alcoholic	Regular	High Class
Occasionally	Regular Exercise	Non Alcoholic	Non Smoker	Poor
Following	No Exercise	Non Alcoholic	Occasionally	Middle Class
Following	No Exercise	Non Alcoholic	Regular	Poor
Following	Regular Exercise	Non Alcoholic	Regular	Poor
Following	No Exercise	Non Alcoholic	Regular	Poor
Following	Regular Exercise	Non Alcoholic	Regular	High Class
Following	Regular Exercise	Occasionally	Occasionally	High Class

Following	No Exercise	Occasionally	Regular	High Class
Following	No Exercise	Non Alcoholic	Regular	Poor
Following	No Exercise	Non Alcoholic	Occasionally	Poor
Not Following	Regular Exercise	Non Alcoholic	Regular	Middle Class
Following	Regular Exercise	Non Alcoholic	Occasionally	Middle Class
Not Following	Regular Exercise	Non Alcoholic	Regular	High Class
Following	No Exercise	Non Alcoholic	Regular	Poor
Following	Regular Exercise	Non Alcoholic	Occasionally	High Class
Following	No Exercise	Occasionally	Regular	Poor
Not Following	Regular Exercise	Non Alcoholic	Regular	Middle Class
Not Following	No Exercise	Non Alcoholic	Regular	Middle Class
Not Following	Regular Exercise	Non Alcoholic	Occasionally	High Class
Following	Regular Exercise	Non Alcoholic	Regular	Poor
Not Following	Regular Exercise	Non Alcoholic	Regular	Poor
Not Following	Regular Exercise	Occasionally	Regular	Poor
Following	Regular Exercise	Non Alcoholic	Regular	High Class
Not Following	Regular Exercise	Non Alcoholic	Occasionally	High Class
Not Following	No Exercise	Non Alcoholic	Regular	High Class
Not Following	Regular Exercise	Non Alcoholic	Non Smoker	High Class
Occasionally	Regular Exercise	Non Alcoholic	Occasionally	High Class
Not Following	Regular Exercise	Occasionally	Regular	Poor
Following	Regular Exercise	Non Alcoholic	Non Smoker	Middle Class
Occasionally	Occasionally	Non Alcoholic	Regular	High Class
Occasionally	Regular Exercise	Non Alcoholic	Regular	High Class
Occasionally	Regular Exercise	Non Alcoholic	Occasionally	High Class
Occasionally	Occasionally	Occasionally	Regular	High Class
Following	No Exercise	Non Alcoholic	Regular	High Class
Following	No Exercise	Non Alcoholic	Regular	High Class
Occasionally	Occasionally	Non Alcoholic	Occasionally	High Class
Occasionally	Regular Exercise	Non Alcoholic	Regular	High Class
Not Following	No Exercise	Non Alcoholic	Regular	Middle Class
Not Following	Regular Exercise	Non Alcoholic	Regular	Middle Class
Following	Regular Exercise	Non Alcoholic	Regular	Middle Class
Following	Regular Exercise	Non Alcoholic	Occasionally	Poor
Not Following	No Exercise	Non Alcoholic	Regular	Poor
Following	Regular Exercise	Occasionally	Regular	High Class
Following	No Exercise	Non Alcoholic	Occasionally	Middle Class
Following	Regular Exercise	Occasionally	Regular	High Class
Not Following	No Exercise	Non Alcoholic	Occasionally	Poor
Not Following	Regular Exercise	Non Alcoholic	Regular	Middle Class
Not Following	Regular Exercise	Non Alcoholic	Regular	High Class
Not Following	No Exercise	Non Alcoholic	Occasionally	Poor
Following	Occasionally	Non Alcoholic	Regular	Middle Class
Not Following	Regular Exercise	Non Alcoholic	Regular	High Class
Following	No Exercise	Occasionally	Regular	Middle Class
Not Following	Regular Exercise	Non Alcoholic	Occasionally	Poor
Following	No Exercise	Non Alcoholic	Non Smoker	High Class

Following	Regular Exercise	Non Alcoholic	Regular	Middle Class
Following	No Exercise	Occasionally	Regular	Poor
Following	Regular Exercise	Non Alcoholic	Regular	High Class
Following	No Exercise	Non Alcoholic	Occasionally	Middle Class
Following	Regular Exercise	Non Alcoholic	Regular	High Class
Not Following	No Exercise	Non Alcoholic	Regular	Middle Class
Following	No Exercise	Non Alcoholic	Occasionally	Poor
Following	Regular Exercise	Non Alcoholic	Regular	Poor
Not Following	Occasionally	Non Alcoholic	Occasionally	Poor
Not Following	No Exercise	Occasionally	Regular	Poor
Occasionally	Regular Exercise	Non Alcoholic	Regular	High Class
Occasionally	Regular Exercise	Non Alcoholic	Occasionally	High Class
Occasionally	No Exercise	Non Alcoholic	Regular	Poor
Not Following	No Exercise	Non Alcoholic	Regular	High Class
Occasionally	No Exercise	Non Alcoholic	Regular	High Class
Occasionally	No Exercise	Non Alcoholic	Occasionally	Middle Class
Occasionally	Regular Exercise	Non Alcoholic	Regular	High Class
Not Following	No Exercise	Non Alcoholic	Regular	Middle Class
Not Following	Regular Exercise	Occasionally	Regular	High Class
Not Following	Occasionally	Non Alcoholic	Regular	Poor
Following	No Exercise	Non Alcoholic	Occasionally	Poor
Following	No Exercise	Non Alcoholic	Regular	Poor
Following	No Exercise	Occasionally	Regular	Poor
Following	Regular Exercise	Non Alcoholic	Occasionally	Poor
Not Following	No Exercise	Non Alcoholic	Non Smoker	High Class
Not Following	Regular Exercise	Non Alcoholic	Occasionally	High Class
Following	No Exercise	Non Alcoholic	Regular	Poor
Following	Regular Exercise	Non Alcoholic	Regular	Poor
Not Following	No Exercise	Non Alcoholic	Occasionally	High Class
Occasionally	Regular Exercise	Non Alcoholic	Regular	Poor
Not Following	No Exercise	Non Alcoholic	Regular	Middle Class
Not Following	Occasionally	Occasionally	Regular	Middle Class
Not Following	Regular Exercise	Non Alcoholic	Occasionally	Middle Class
Occasionally	Occasionally	Non Alcoholic	Regular	Poor
Occasionally	Regular Exercise	Non Alcoholic	Regular	Poor
Not Following	Regular Exercise	Non Alcoholic	Regular	High Class
Not Following	Occasionally	Non Alcoholic	Regular	Middle Class
Not Following	No Exercise	Non Alcoholic	Occasionally	Poor
Not Following	Regular Exercise	Non Alcoholic	Regular	Poor
Not Following	Regular Exercise	Non Alcoholic	Regular	Middle Class
Occasionally	Regular Exercise	Occasionally	Occasionally	Middle Class
Not Following	No Exercise	Non Alcoholic	Regular	High Class
Occasionally	Regular Exercise	Non Alcoholic	Occasionally	High Class
Following	Regular Exercise	Non Alcoholic	Regular	High Class
Following	Regular Exercise	Non Alcoholic	Regular	Poor
Not Following	Regular Exercise	Non Alcoholic	Occasionally	Poor
Not Following	No Exercise	Occasionally	Non Smoker	High Class

Not Following	Regular Exercise	Occasionally	Regular	Poor
Following	Regular Exercise	Non Alcoholic	Regular	Poor
Following	Regular Exercise	Non Alcoholic	Regular	Poor
Not Following	No Exercise	Alcoholic	Regular	Poor
Following	Occasionally	Non Alcoholic	Occasionally	Poor
Not Following	Regular Exercise	Non Alcoholic	Regular	High Class
Following	No Exercise	Non Alcoholic	Regular	Poor
Not Following	Regular Exercise	Occasionally	Occasionally	High Class
Following	Occasionally	Non Alcoholic	Regular	Poor
Following	Regular Exercise	Non Alcoholic	Occasionally	Poor
Not Following	No Exercise	Non Alcoholic	Regular	High Class
Following	Regular Exercise	Non Alcoholic	Regular	Middle Class
Not Following	No Exercise	Non Alcoholic	Occasionally	Middle Class
Not Following	No Exercise	Alcoholic	Regular	Middle Class
Following	Regular Exercise	Non Alcoholic	Regular	Poor
Not Following	Regular Exercise	Non Alcoholic	Regular	High Class
Following	Regular Exercise	Non Alcoholic	Occasionally	Middle Class
Not Following	Regular Exercise	Non Alcoholic	Regular	Poor
Not Following	Regular Exercise	Occasionally	Regular	Poor
Following	No Exercise	Non Alcoholic	Regular	High Class
Following	Regular Exercise	Non Alcoholic	Regular	Poor
Following	Regular Exercise	Non Alcoholic	Occasionally	Poor
Not Following	Regular Exercise	Non Alcoholic	Regular	Middle Class
Following	No Exercise	Occasionally	Regular	Poor
Following	Regular Exercise	Non Alcoholic	Occasionally	Poor
Not Following	Regular Exercise	Non Alcoholic	Regular	High Class
Not Following	Regular Exercise	Occasionally	Occasionally	High Class
Following	Regular Exercise	Non Alcoholic	Regular	Poor
Following	No Exercise	Non Alcoholic	Regular	Poor
Not Following	No Exercise	Alcoholic	Occasionally	Poor
Not Following	No Exercise	Non Alcoholic	Regular	Poor
Following	Regular Exercise	Non Alcoholic	Regular	Poor
Not Following	No Exercise	Non Alcoholic	Non Smoker	High Class
Not Following	No Exercise	Occasionally	Occasionally	High Class
Not Following	No Exercise	Non Alcoholic	Regular	High Class
Not Following	Regular Exercise	Non Alcoholic	Regular	High Class
Not Following	No Exercise	Non Alcoholic	Regular	High Class
Following	Regular Exercise	Non Alcoholic	Regular	High Class
Not Following	No Exercise	Occasionally	Occasionally	Middle Class
Following	Regular Exercise	Non Alcoholic	Regular	Middle Class
Not Following	Regular Exercise	Non Alcoholic	Regular	Middle Class
Not Following	Regular Exercise	Non Alcoholic	Occasionally	Middle Class
Not Following	Regular Exercise	Non Alcoholic	Regular	High Class
Following	No Exercise	Non Alcoholic	Occasionally	High Class
Not Following	Regular Exercise	Non Alcoholic	Regular	High Class
Following	No Exercise	Alcoholic	Regular	High Class
Not Following	No Exercise	Non Alcoholic	Occasionally	High Class

Following	Regular Exercise	Non Alcoholic	Regular	Middle Class
Following	Occasionally	Occasionally	Regular	Poor
Following	Occasionally	Non Alcoholic	Regular	Middle Class
Following	No Exercise	Non Alcoholic	Occasionally	Middle Class
Not Following	Occasionally	Non Alcoholic	Regular	High Class
Following	No Exercise	Non Alcoholic	Regular	Poor
Following	No Exercise	Non Alcoholic	Regular	Middle Class
Following	No Exercise	Non Alcoholic	Non Smoker	High Class
Following	Regular Exercise	Non Alcoholic	Occasionally	Middle Class
Occasionally	Regular Exercise	Non Alcoholic	Regular	High Class
Occasionally	No Exercise	Occasionally	Non Smoker	Middle Class
Occasionally	Occasionally	Occasionally	Regular	High Class
Not Following	No Exercise	Occasionally	Regular	High Class
Not Following	Occasionally	Non Alcoholic	Occasionally	High Class
Not Following	No Exercise	Non Alcoholic	Regular	Middle Class
Occasionally	No Exercise	Non Alcoholic	Regular	Middle Class
Occasionally	Regular Exercise	Non Alcoholic	Regular	Middle Class
Occasionally	No Exercise	Occasionally	Occasionally	Middle Class
Not Following	No Exercise	Non Alcoholic	Regular	Poor
Not Following	Regular Exercise	Non Alcoholic	Regular	Poor
Following	No Exercise	Non Alcoholic	Regular	High Class
Following	No Exercise	Non Alcoholic	Regular	High Class
Not Following	No Exercise	Non Alcoholic	Occasionally	High Class
Following	No Exercise	Non Alcoholic	Regular	Middle Class
Not Following	No Exercise	Occasionally	Regular	Middle Class
Not Following	Regular Exercise	Non Alcoholic	Occasionally	Middle Class
Not Following	Regular Exercise	Non Alcoholic	Regular	Middle Class
Following	Regular Exercise	Occasionally	Occasionally	Middle Class
Occasionally	Occasionally	Occasionally	Occasionally	Poor
Occasionally	Occasionally	Non Alcoholic	Occasionally	Poor
Following	Regular Exercise	Non Alcoholic	Occasionally	Poor
Occasionally	No Exercise	Occasionally	Non Smoker	Poor
Following	Regular Exercise	Non Alcoholic	Occasionally	Poor
Following	No Exercise	Occasionally	Occasionally	Middle Class
Occasionally	Regular Exercise	Occasionally	Occasionally	Middle Class
Occasionally	Regular Exercise	Occasionally	Occasionally	Middle Class
Following	No Exercise	Alcoholic	Non Smoker	Middle Class
Occasionally	No Exercise	Occasionally	Regular	Middle Class
Occasionally	Occasionally	Occasionally	Regular	Middle Class
Following	Regular Exercise	Alcoholic	Occasionally	High Class
Occasionally	Occasionally	Alcoholic	Occasionally	High Class
Following	No Exercise	Non Alcoholic	Regular	High Class
Following	Regular Exercise	Non Alcoholic	Regular	Poor
Following	No Exercise	Alcoholic	Occasionally	High Class
Occasionally	No Exercise	Non Alcoholic	Regular	Poor
Not Following	Regular Exercise	Occasionally	Occasionally	Poor
Following	Regular Exercise	Alcoholic	Regular	Middle Class

Not Following	No Exercise	Occasionally	Regular	High Class
Following	Regular Exercise	Non Alcoholic	Non Smoker	High Class
Following	Regular Exercise	Alcoholic	Regular	Middle Class
Following	Regular Exercise	Non Alcoholic	Occasionally	High Class
Not Following	Regular Exercise	Alcoholic	Occasionally	High Class
Not Following	No Exercise	Alcoholic	Regular	High Class
Not Following	No Exercise	Occasionally	Occasionally	Middle Class
Following	No Exercise	Occasionally	Non Smoker	High Class
Not Following	Occasionally	Alcoholic	Regular	Poor
Not Following	No Exercise	Alcoholic	Non Smoker	High Class
Occasionally	Regular Exercise	Non Alcoholic	Non Smoker	Poor
Occasionally	Regular Exercise	Non Alcoholic	Non Smoker	Poor
Not Following	Occasionally	Non Alcoholic	Regular	High Class
Following	Regular Exercise	Non Alcoholic	Non Smoker	Poor
Occasionally	Regular Exercise	Occasionally	Regular	Poor
Following	No Exercise	Occasionally	Regular	High Class
Occasionally	Regular Exercise	Occasionally	Occasionally	Middle Class
Not Following	Regular Exercise	Non Alcoholic	Regular	High Class
Following	Regular Exercise	Occasionally	Occasionally	Middle Class
Following	Occasionally	Occasionally	Occasionally	Middle Class
Following	No Exercise	Alcoholic	Occasionally	High Class
Following	No Exercise	Non Alcoholic	Regular	High Class
Not Following	Regular Exercise	Alcoholic	Regular	Poor
Following	Occasionally	Non Alcoholic	Regular	Poor
Occasionally	Occasionally	Non Alcoholic	Regular	Poor
Occasionally	No Exercise	Occasionally	Occasionally	Middle Class
Occasionally	Regular Exercise	Alcoholic	Regular	High Class
Not Following	No Exercise	Occasionally	Occasionally	High Class
Occasionally	Regular Exercise	Non Alcoholic	Non Smoker	High Class
Not Following	No Exercise	Non Alcoholic	Non Smoker	Poor
Not Following	No Exercise	Alcoholic	Occasionally	Poor
Occasionally	Occasionally	Occasionally	Occasionally	Poor
Not Following	No Exercise	Alcoholic	Regular	High Class
Following	Regular Exercise	Alcoholic	Regular	Poor
Not Following	Regular Exercise	Occasionally	Regular	Poor
Occasionally	Occasionally	Occasionally	Occasionally	High Class
Not Following	Occasionally	Occasionally	Regular	Middle Class
Occasionally	No Exercise	Non Alcoholic	Non Smoker	Middle Class
Not Following	No Exercise	Non Alcoholic	Non Smoker	High Class
Not Following	Regular Exercise	Alcoholic	Regular	High Class
Following	No Exercise	Non Alcoholic	Non Smoker	Poor
Not Following	No Exercise	Non Alcoholic	Regular	Poor
Occasionally	No Exercise	Occasionally	Regular	Poor
Not Following	Occasionally	Occasionally	Occasionally	Middle Class
Following	Regular Exercise	Non Alcoholic	Non Smoker	High Class
Not Following	Occasionally	Alcoholic	Regular	High Class
Not Following	No Exercise	Non Alcoholic	Occasionally	Middle Class

Not Following	No Exercise	Occasionally	Regular	Middle Class
Not Following	Regular Exercise	Occasionally	Regular	Middle Class
Following	No Exercise	Non Alcoholic	Occasionally	High Class
Not Following	Occasionally	Occasionally	Occasionally	Poor
Occasionally	No Exercise	Non Alcoholic	Non Smoker	Middle Class
Occasionally	Regular Exercise	Occasionally	Occasionally	High Class
Following	No Exercise	Alcoholic	Regular	Poor
Not Following	No Exercise	Non Alcoholic	Regular	Poor
Occasionally	Regular Exercise	Non Alcoholic	Non Smoker	Poor
Following	No Exercise	Alcoholic	Occasionally	Poor
Following	Regular Exercise	Non Alcoholic	Occasionally	High Class
Following	No Exercise	Non Alcoholic	Non Smoker	Middle Class
Occasionally	No Exercise	Non Alcoholic	Regular	High Class
Following	Regular Exercise	Non Alcoholic	Occasionally	Middle Class
Occasionally	Occasionally	Non Alcoholic	Occasionally	High Class
Following	Occasionally	Non Alcoholic	Occasionally	Poor
Following	Regular Exercise	Non Alcoholic	Non Smoker	Middle Class
Occasionally	Occasionally	Occasionally	Occasionally	Poor
Following	Occasionally	Occasionally	Occasionally	Middle Class
Following	No Exercise	Alcoholic	Non Smoker	Poor
Following	No Exercise	Non Alcoholic	Regular	Poor
Occasionally	No Exercise	Occasionally	Non Smoker	Poor
Not Following	No Exercise	Non Alcoholic	Regular	High Class
Following	Occasionally	Alcoholic	Regular	High Class
Not Following	Occasionally	Occasionally	Occasionally	High Class
Following	No Exercise	Non Alcoholic	Non Smoker	High Class
Not Following	No Exercise	Alcoholic	Regular	High Class
Following	Regular Exercise	Occasionally	Occasionally	Middle Class
Following	No Exercise	Occasionally	Regular	Poor
Not Following	Occasionally	Non Alcoholic	Non Smoker	High Class
Occasionally	No Exercise	Non Alcoholic	Regular	High Class
Not Following	No Exercise	Non Alcoholic	Non Smoker	Middle Class
Occasionally	Occasionally	Occasionally	Occasionally	High Class
Occasionally	Occasionally	Alcoholic	Non Smoker	High Class
Not Following	No Exercise	Occasionally	Occasionally	Poor
Occasionally	No Exercise	Non Alcoholic	Occasionally	Poor
Not Following	Occasionally	Non Alcoholic	Non Smoker	Middle Class
Occasionally	Occasionally	Non Alcoholic	Non Smoker	Middle Class
Not Following	Regular Exercise	Alcoholic	Regular	Middle Class
Following	Regular Exercise	Non Alcoholic	Regular	Middle Class
Following	No Exercise	Non Alcoholic	Non Smoker	Middle Class
Following	Occasionally	Non Alcoholic	Non Smoker	High Class
Occasionally	Occasionally	Non Alcoholic	Non Smoker	High Class
Not Following	No Exercise	Non Alcoholic	Non Smoker	Middle Class
Following	No Exercise	Alcoholic	Non Smoker	Middle Class
Not Following	No Exercise	Non Alcoholic	Non Smoker	High Class
Following	No Exercise	Non Alcoholic	Non Smoker	Poor

Following	No Exercise	Alcoholic	Regular	Poor
Not Following	No Exercise	Non Alcoholic	Non Smoker	High Class
Following	Occasionally	Occasionally	Occasionally	High Class
Not Following	No Exercise	Alcoholic	Regular	Poor
Not Following	No Exercise	Non Alcoholic	Non Smoker	Middle Class
Following	Occasionally	Occasionally	Occasionally	Poor
Following	Regular Exercise	Non Alcoholic	Non Smoker	Poor
Following	No Exercise	Alcoholic	Regular	Middle Class
Following	No Exercise	Non Alcoholic	Occasionally	Poor
Not Following	Occasionally	Occasionally	Regular	Middle Class
Not Following	No Exercise	Non Alcoholic	Non Smoker	Middle Class
Not Following	No Exercise	Occasionally	Occasionally	Poor
Not Following	No Exercise	Non Alcoholic	Regular	Middle Class
Not Following	No Exercise	Alcoholic	Regular	Poor
Not Following	Regular Exercise	Occasionally	Non Smoker	Poor
Not Following	Occasionally	Occasionally	Non Smoker	High Class
Following	Regular Exercise	Alcoholic	Regular	Poor
Not Following	Regular Exercise	Non Alcoholic	Occasionally	High Class
Following	Regular Exercise	Occasionally	Regular	High Class
Following	Occasionally	Non Alcoholic	Non Smoker	High Class
Following	Regular Exercise	Alcoholic	Regular	Middle Class
Occasionally	Occasionally	Occasionally	Occasionally	Middle Class
Following	Regular Exercise	Non Alcoholic	Non Smoker	Middle Class
Following	No Exercise	Occasionally	Regular	Middle Class
Occasionally	No Exercise	Non Alcoholic	Occasionally	Middle Class
Following	Regular Exercise	Occasionally	Occasionally	Poor
Following	No Exercise	Alcoholic	Regular	Poor
Occasionally	No Exercise	Occasionally	Regular	High Class
Following	Occasionally	Non Alcoholic	Occasionally	Poor
Following	Occasionally	Alcoholic	Occasionally	Middle Class
Following	Regular Exercise	Non Alcoholic	Non Smoker	Middle Class
Occasionally	No Exercise	Non Alcoholic	Occasionally	Poor
Not Following	No Exercise	Non Alcoholic	Regular	Poor
Following	Regular Exercise	Occasionally	Regular	High Class
Following	Regular Exercise	Non Alcoholic	Occasionally	Poor
Occasionally	No Exercise	Non Alcoholic	Non Smoker	Poor
Following	Regular Exercise	Occasionally	Regular	Poor
Not Following	Regular Exercise	Occasionally	Occasionally	Middle Class
Following	Regular Exercise	Non Alcoholic	Non Smoker	High Class
Occasionally	Occasionally	Occasionally	Occasionally	Poor
Following	Regular Exercise	Alcoholic	Occasionally	High Class
Following	No Exercise	Non Alcoholic	Regular	Middle Class
Not Following	Regular Exercise	Alcoholic	Non Smoker	Poor
Occasionally	Regular Exercise	Non Alcoholic	Regular	High Class
Following	No Exercise	Non Alcoholic	Non Smoker	Poor
Following	Regular Exercise	Occasionally	Non Smoker	High Class
Following	Regular Exercise	Alcoholic	Occasionally	Poor

Following	Regular Exercise	Alcoholic	Regular	High Class
Following	Occasionally	Non Alcoholic	Non Smoker	Poor
Following	No Exercise	Alcoholic	Occasionally	Middle Class
Following	Occasionally	Occasionally	Occasionally	Middle Class
Occasionally	Regular Exercise	Non Alcoholic	Regular	Poor
Occasionally	No Exercise	Non Alcoholic	Occasionally	Middle Class
Following	Regular Exercise	Non Alcoholic	Non Smoker	Middle Class
Following	Regular Exercise	Non Alcoholic	Non Smoker	Poor
Following	Occasionally	Alcoholic	Regular	High Class
Occasionally	No Exercise	Alcoholic	Non Smoker	Middle Class
Occasionally	No Exercise	Non Alcoholic	Regular	High Class
Following	No Exercise	Non Alcoholic	Non Smoker	Poor
Following	No Exercise	Occasionally	Non Smoker	High Class
Occasionally	Regular Exercise	Non Alcoholic	Occasionally	High Class
Following	Regular Exercise	Alcoholic	Regular	High Class
Occasionally	No Exercise	Alcoholic	Regular	Poor
Occasionally	No Exercise	Non Alcoholic	Non Smoker	High Class
Occasionally	Regular Exercise	Occasionally	Non Smoker	Poor
Following	Occasionally	Alcoholic	Non Smoker	Poor
Occasionally	No Exercise	Occasionally	Occasionally	High Class
Not Following	Regular Exercise	Non Alcoholic	Non Smoker	Poor
Occasionally	Occasionally	Alcoholic	Non Smoker	Poor
Following	Regular Exercise	Alcoholic	Non Smoker	Middle Class
Following	Regular Exercise	Alcoholic	Regular	High Class
Following	Regular Exercise	Alcoholic	Non Smoker	Middle Class
Occasionally	Regular Exercise	Occasionally	Regular	Poor
Occasionally	Occasionally	Occasionally	Regular	Poor
Occasionally	No Exercise	Alcoholic	Regular	High Class
Occasionally	Regular Exercise	Non Alcoholic	Regular	High Class
Occasionally	Regular Exercise	Non Alcoholic	Occasionally	High Class
Occasionally	Regular Exercise	Occasionally	Non Smoker	High Class
Occasionally	Regular Exercise	Alcoholic	Regular	High Class
Occasionally	Occasionally	Alcoholic	Occasionally	High Class
Occasionally	No Exercise	Occasionally	Occasionally	High Class
Following	Regular Exercise	Non Alcoholic	Non Smoker	Poor
Occasionally	Regular Exercise	Alcoholic	Non Smoker	Poor
Following	Regular Exercise	Occasionally	Non Smoker	Poor
Occasionally	Occasionally	Non Alcoholic	Regular	High Class
Occasionally	Occasionally	Non Alcoholic	Occasionally	High Class
Occasionally	Occasionally	Non Alcoholic	Non Smoker	Poor
Occasionally	No Exercise	Non Alcoholic	Regular	Poor
Not Following	Regular Exercise	Alcoholic	Non Smoker	High Class
Occasionally	Regular Exercise	Alcoholic	Occasionally	Poor
Following	Regular Exercise	Alcoholic	Occasionally	High Class
Following	Regular Exercise	Alcoholic	Occasionally	Poor
Following	Regular Exercise	Alcoholic	Occasionally	Poor
Not Following	Regular Exercise	Non Alcoholic	Occasionally	High Class

Occasionally	Occasionally	Non Alcoholic	Non Smoker	Middle Class
Not Following	No Exercise	Non Alcoholic	Regular	Poor
Occasionally	Regular Exercise	Non Alcoholic	Regular	High Class
Not Following	Regular Exercise	Non Alcoholic	Occasionally	High Class
Following	Occasionally	Alcoholic	Non Smoker	Poor
Not Following	Regular Exercise	Non Alcoholic	Non Smoker	High Class
Occasionally	Occasionally	Alcoholic	Occasionally	Poor
Occasionally	Regular Exercise	Occasionally	Occasionally	Poor
Occasionally	Regular Exercise	Occasionally	Regular	Poor
Following	Regular Exercise	Alcoholic	Occasionally	Poor
Following	Occasionally	Alcoholic	Regular	Middle Class
Following	No Exercise	Non Alcoholic	Regular	Middle Class
Not Following	Occasionally	Occasionally	Occasionally	Poor
Occasionally	Regular Exercise	Alcoholic	Non Smoker	High Class
Following	Regular Exercise	Non Alcoholic	Occasionally	High Class
Not Following	No Exercise	Alcoholic	Regular	Middle Class
Occasionally	Regular Exercise	Occasionally	Regular	High Class
Not Following	No Exercise	Occasionally	Occasionally	Poor
Occasionally	Regular Exercise	Alcoholic	Regular	Poor
Occasionally	Regular Exercise	Occasionally	Non Smoker	Poor
Occasionally	No Exercise	Alcoholic	Occasionally	Poor
Not Following	Occasionally	Non Alcoholic	Occasionally	Poor
Occasionally	No Exercise	Alcoholic	Occasionally	High Class
Not Following	Regular Exercise	Non Alcoholic	Non Smoker	Poor
Occasionally	Regular Exercise	Occasionally	Non Smoker	Poor
Following	Occasionally	Occasionally	Non Smoker	Middle Class
Not Following	No Exercise	Non Alcoholic	Regular	Middle Class
Following	No Exercise	Non Alcoholic	Regular	High Class
Occasionally	Occasionally	Occasionally	Regular	Poor
Occasionally	Regular Exercise	Non Alcoholic	Occasionally	Middle Class
Occasionally	Regular Exercise	Non Alcoholic	Regular	High Class
Not Following	Occasionally	Alcoholic	Non Smoker	Poor
Following	Regular Exercise	Alcoholic	Occasionally	High Class
Occasionally	Occasionally	Alcoholic	Regular	Middle Class
Not Following	Occasionally	Non Alcoholic	Non Smoker	Middle Class
Occasionally	Occasionally	Alcoholic	Non Smoker	Middle Class
Not Following	Occasionally	Alcoholic	Non Smoker	High Class
Not Following	No Exercise	Non Alcoholic	Regular	Poor
Following	No Exercise	Alcoholic	Regular	High Class
Following	No Exercise	Occasionally	Occasionally	High Class
Occasionally	No Exercise	Occasionally	Regular	Middle Class
Following	No Exercise	Non Alcoholic	Occasionally	High Class
Occasionally	Regular Exercise	Non Alcoholic	Occasionally	High Class
Occasionally	Regular Exercise	Alcoholic	Occasionally	High Class
Following	No Exercise	Alcoholic	Regular	Middle Class
Following	Occasionally	Non Alcoholic	Regular	High Class
Following	Occasionally	Non Alcoholic	Regular	Poor

Following	No Exercise	Non Alcoholic	Non Smoker	Poor
Occasionally	Regular Exercise	Non Alcoholic	Non Smoker	Poor
Occasionally	Regular Exercise	Occasionally	Non Smoker	High Class
Following	No Exercise	Occasionally	Non Smoker	Poor
Not Following	No Exercise	Alcoholic	Non Smoker	Poor
Occasionally	Regular Exercise	Alcoholic	Non Smoker	High Class
Not Following	Occasionally	Alcoholic	Non Smoker	High Class
Following	Occasionally	Occasionally	Non Smoker	Middle Class
Occasionally	Regular Exercise	Occasionally	Non Smoker	High Class
Following	Regular Exercise	Alcoholic	Non Smoker	High Class
Following	Occasionally	Non Alcoholic	Non Smoker	Poor
Not Following	Regular Exercise	Non Alcoholic	Regular	High Class
Occasionally	No Exercise	Occasionally	Non Smoker	Middle Class
Occasionally	No Exercise	Occasionally	Non Smoker	High Class
Following	Regular Exercise	Alcoholic	Non Smoker	High Class
Not Following	No Exercise	Alcoholic	Occasionally	Poor
Not Following	Regular Exercise	Alcoholic	Non Smoker	High Class
Not Following	Occasionally	Occasionally	Non Smoker	High Class
Following	Occasionally	Alcoholic	Non Smoker	High Class
Following	Occasionally	Non Alcoholic	Non Smoker	High Class
Following	No Exercise	Non Alcoholic	Regular	High Class
Following	Occasionally	Non Alcoholic	Non Smoker	Middle Class
Following	Regular Exercise	Occasionally	Non Smoker	Middle Class
Not Following	Regular Exercise	Occasionally	Non Smoker	Middle Class
Not Following	Occasionally	Occasionally	Non Smoker	Middle Class
Not Following	Occasionally	Alcoholic	Non Smoker	Middle Class
Occasionally	No Exercise	Alcoholic	Regular	Middle Class
Not Following	No Exercise	Alcoholic	Regular	Poor
Following	Regular Exercise	Alcoholic	Occasionally	Poor
Occasionally	Regular Exercise	Alcoholic	Occasionally	Poor
Occasionally	Occasionally	Non Alcoholic	Occasionally	Poor
Not Following	No Exercise	Non Alcoholic	Regular	High Class
Following	No Exercise	Non Alcoholic	Regular	High Class
Following	Regular Exercise	Occasionally	Regular	High Class
Not Following	Regular Exercise	Occasionally	Occasionally	Poor
Occasionally	No Exercise	Occasionally	Occasionally	High Class
Occasionally	No Exercise	Alcoholic	Non Smoker	Poor
Following	Occasionally	Occasionally	Non Smoker	Middle Class
Not Following	Occasionally	Occasionally	Non Smoker	Middle Class
Not Following	Regular Exercise	Non Alcoholic	Non Smoker	Middle Class
Occasionally	Occasionally	Non Alcoholic	Occasionally	Middle Class
Following	No Exercise	Occasionally	Occasionally	Middle Class
Following	Regular Exercise	Occasionally	Occasionally	Middle Class
Following	Regular Exercise	Occasionally	Regular	Poor
Not Following	No Exercise	Non Alcoholic	Occasionally	High Class
Following	No Exercise	Non Alcoholic	Regular	Poor
Occasionally	Regular Exercise	Non Alcoholic	Occasionally	Middle Class

Following	Regular Exercise	Non Alcoholic	Regular	High Class
Occasionally	Occasionally	Non Alcoholic	Regular	Middle Class
Not Following	No Exercise	Occasionally	Occasionally	Poor
Following	No Exercise	Non Alcoholic	Regular	Poor
Not Following	No Exercise	Non Alcoholic	Regular	Middle Class
Following	No Exercise	Occasionally	Regular	Middle Class
Not Following	No Exercise	Non Alcoholic	Occasionally	Middle Class
Following	Regular Exercise	Non Alcoholic	Regular	High Class
Not Following	No Exercise	Non Alcoholic	Regular	High Class
Following	Regular Exercise	Occasionally	Regular	High Class
Not Following	Occasionally	Non Alcoholic	Regular	Middle Class
Not Following	Occasionally	Non Alcoholic	Occasionally	Middle Class
Not Following	Regular Exercise	Non Alcoholic	Regular	Middle Class
Not Following	Regular Exercise	Non Alcoholic	Regular	Middle Class
Not Following	No Exercise	Occasionally	Occasionally	Poor
Following	Regular Exercise	Non Alcoholic	Regular	Poor
Following	No Exercise	Non Alcoholic	Occasionally	Poor
Following	Regular Exercise	Non Alcoholic	Regular	High Class
Occasionally	No Exercise	Occasionally	Regular	High Class
Occasionally	Regular Exercise	Non Alcoholic	Occasionally	High Class
Occasionally	Occasionally	Alcoholic	Regular	High Class
Occasionally	Regular Exercise	Alcoholic	Regular	Middle Class
Occasionally	Occasionally	Non Alcoholic	Regular	High Class
Occasionally	Regular Exercise	Non Alcoholic	Occasionally	Middle Class
Occasionally	Regular Exercise	Occasionally	Regular	High Class
Occasionally	Regular Exercise	Non Alcoholic	Regular	Middle Class
Not Following	No Exercise	Occasionally	Regular	High Class
Occasionally	No Exercise	Non Alcoholic	Regular	Poor
Not Following	Occasionally	Non Alcoholic	Occasionally	Poor
Not Following	No Exercise	Non Alcoholic	Regular	Middle Class
Not Following	Regular Exercise	Non Alcoholic	Regular	Middle Class
Following	Occasionally	Non Alcoholic	Occasionally	Middle Class
Occasionally	No Exercise	Non Alcoholic	Regular	Middle Class
Not Following	Regular Exercise	Non Alcoholic	Occasionally	High Class
Occasionally	No Exercise	Non Alcoholic	Regular	High Class
Not Following	No Exercise	Non Alcoholic	Regular	Poor
Occasionally	No Exercise	Occasionally	Occasionally	Poor
Not Following	No Exercise	Non Alcoholic	Regular	Middle Class
Not Following	Regular Exercise	Non Alcoholic	Regular	Middle Class
Following	Regular Exercise	Alcoholic	Regular	Middle Class
Not Following	No Exercise	Non Alcoholic	Occasionally	Middle Class
Following	Regular Exercise	Alcoholic	Regular	Middle Class
Following	Occasionally	Alcoholic	Regular	High Class
Following	Regular Exercise	Non Alcoholic	Non Smoker	Middle Class
Following	No Exercise	Non Alcoholic	Regular	High Class
Following	Regular Exercise	Occasionally	Occasionally	High Class
Not Following	No Exercise	Non Alcoholic	Regular	High Class

Following	Occasionally	Non Alcoholic	Regular	High Class
Following	Occasionally	Occasionally	Occasionally	High Class
Following	No Exercise	Non Alcoholic	Regular	High Class
Following	No Exercise	Non Alcoholic	Occasionally	Poor
Not Following	No Exercise	Non Alcoholic	Regular	Poor
Following	Occasionally	Non Alcoholic	Regular	Poor
Occasionally	Regular Exercise	Non Alcoholic	Occasionally	Middle Class
Following	No Exercise	Non Alcoholic	Regular	High Class
Following	No Exercise	Non Alcoholic	Regular	Poor
Occasionally	Occasionally	Non Alcoholic	Regular	Poor
Not Following	No Exercise	Occasionally	Occasionally	Poor
Following	No Exercise	Non Alcoholic	Regular	Poor
Following	No Exercise	Non Alcoholic	Regular	Poor
Following	Regular Exercise	Non Alcoholic	Regular	Poor
Occasionally	Regular Exercise	Alcoholic	Regular	Poor
Occasionally	Regular Exercise	Occasionally	Occasionally	Poor
Not Following	No Exercise	Non Alcoholic	Regular	Middle Class
Occasionally	No Exercise	Non Alcoholic	Non Smoker	High Class
Not Following	Regular Exercise	Non Alcoholic	Occasionally	Poor
Not Following	Regular Exercise	Alcoholic	Regular	Middle Class
Following	Regular Exercise	Non Alcoholic	Occasionally	Poor
Not Following	Regular Exercise	Non Alcoholic	Non Smoker	Poor
Following	No Exercise	Non Alcoholic	Regular	Poor
Not Following	Occasionally	Non Alcoholic	Occasionally	High Class
Not Following	Regular Exercise	Non Alcoholic	Regular	High Class
Not Following	Regular Exercise	Occasionally	Regular	High Class
Following	Regular Exercise	Non Alcoholic	Regular	Poor
Not Following	No Exercise	Non Alcoholic	Occasionally	Poor
Not Following	Regular Exercise	Non Alcoholic	Regular	Middle Class
Not Following	Regular Exercise	Non Alcoholic	Regular	Middle Class
Not Following	Occasionally	Occasionally	Regular	High Class
Not Following	No Exercise	Non Alcoholic	Regular	Poor
Not Following	Regular Exercise	Occasionally	Regular	Middle Class
Not Following	Regular Exercise	Occasionally	Regular	Middle Class
Not Following	Regular Exercise	Non Alcoholic	Occasionally	Middle Class
Not Following	Occasionally	Non Alcoholic	Regular	Poor
Occasionally	Regular Exercise	Non Alcoholic	Regular	Poor
Occasionally	Regular Exercise	Non Alcoholic	Regular	High Class
Occasionally	Occasionally	Occasionally	Occasionally	Middle Class
Occasionally	Regular Exercise	Non Alcoholic	Regular	High Class
Following	Regular Exercise	Non Alcoholic	Regular	Poor
Not Following	Regular Exercise	Non Alcoholic	Regular	Middle Class
Not Following	Regular Exercise	Non Alcoholic	Regular	High Class
Not Following	Regular Exercise	Non Alcoholic	Occasionally	Poor
Not Following	Regular Exercise	Occasionally	Non Smoker	Middle Class
Not Following	Occasionally	Non Alcoholic	Regular	High Class
Not Following	Regular Exercise	Non Alcoholic	Occasionally	Middle Class

Following	Regular Exercise	Occasionally	Regular	Poor
Not Following	Occasionally	Non Alcoholic	Occasionally	High Class
Not Following	Regular Exercise	Non Alcoholic	Regular	Middle Class
Not Following	Regular Exercise	Non Alcoholic	Regular	Poor
Not Following	Regular Exercise	Non Alcoholic	Occasionally	High Class
Following	No Exercise	Non Alcoholic	Regular	Middle Class
Following	No Exercise	Non Alcoholic	Regular	High Class
Not Following	No Exercise	Occasionally	Regular	Middle Class
Not Following	No Exercise	Non Alcoholic	Occasionally	Poor
Following	No Exercise	Non Alcoholic	Regular	Poor
Not Following	Regular Exercise	Non Alcoholic	Regular	Poor
Following	Regular Exercise	Non Alcoholic	Regular	Poor
Following	Occasionally	Occasionally	Regular	High Class
Occasionally	Regular Exercise	Non Alcoholic	Occasionally	High Class
Not Following	No Exercise	Non Alcoholic	Regular	Poor
Not Following	Regular Exercise	Non Alcoholic	Regular	High Class
Occasionally	No Exercise	Non Alcoholic	Occasionally	High Class
Occasionally	Regular Exercise	Non Alcoholic	Regular	Middle Class
Occasionally	No Exercise	Occasionally	Occasionally	High Class
Occasionally	Regular Exercise	Non Alcoholic	Regular	Middle Class
Occasionally	Regular Exercise	Non Alcoholic	Regular	High Class
Not Following	No Exercise	Non Alcoholic	Occasionally	Poor
Following	Occasionally	Non Alcoholic	Regular	Poor
Not Following	No Exercise	Non Alcoholic	Regular	Poor
Occasionally	No Exercise	Occasionally	Regular	Poor
Following	Regular Exercise	Non Alcoholic	Occasionally	Poor
Following	Regular Exercise	Non Alcoholic	Non Smoker	High Class
Not Following	Regular Exercise	Non Alcoholic	Regular	High Class
Not Following	Regular Exercise	Non Alcoholic	Regular	Poor
Following	Regular Exercise	Non Alcoholic	Regular	Poor
Not Following	Regular Exercise	Non Alcoholic	Occasionally	High Class
Not Following	Occasionally	Non Alcoholic	Regular	Poor
Following	No Exercise	Alcoholic	Regular	Middle Class
Occasionally	Regular Exercise	Non Alcoholic	Occasionally	Middle Class
Not Following	Regular Exercise	Non Alcoholic	Regular	Middle Class
Following	Regular Exercise	Non Alcoholic	Occasionally	Poor
Following	No Exercise	Non Alcoholic	Regular	Poor
Not Following	Regular Exercise	Non Alcoholic	Regular	High Class
Not Following	No Exercise	Non Alcoholic	Occasionally	Middle Class
Not Following	Regular Exercise	Non Alcoholic	Regular	Poor
Not Following	Occasionally	Non Alcoholic	Regular	Poor
Occasionally	Regular Exercise	Non Alcoholic	Regular	Middle Class
Following	No Exercise	Non Alcoholic	Occasionally	Middle Class
Occasionally	Regular Exercise	Non Alcoholic	Regular	High Class
Following	Occasionally	Non Alcoholic	Regular	High Class
Occasionally	Regular Exercise	Alcoholic	Regular	High Class
Following	No Exercise	Non Alcoholic	Regular	Poor

Occasionally	Regular Exercise	Non Alcoholic	Non Smoker	Poor
Occasionally	No Exercise	Non Alcoholic	Regular	High Class
Following	Occasionally	Non Alcoholic	Regular	Middle Class
Following	No Exercise	Non Alcoholic	Occasionally	High Class
Occasionally	Regular Exercise	Alcoholic	Regular	Middle Class
Occasionally	Occasionally	Non Alcoholic	Occasionally	High Class
Occasionally	Regular Exercise	Non Alcoholic	Regular	High Class
Not Following	Regular Exercise	Non Alcoholic	Regular	High Class
Not Following	Regular Exercise	Non Alcoholic	Occasionally	High Class
Not Following	No Exercise	Non Alcoholic	Regular	High Class
Not Following	Occasionally	Non Alcoholic	Regular	Poor
Not Following	No Exercise	Occasionally	Regular	High Class
Following	Occasionally	Non Alcoholic	Occasionally	High Class
Following	No Exercise	Non Alcoholic	Regular	High Class
Following	No Exercise	Non Alcoholic	Regular	High Class
Not Following	No Exercise	Occasionally	Regular	High Class
Following	Occasionally	Non Alcoholic	Regular	Middle Class
Not Following	Regular Exercise	Non Alcoholic	Occasionally	High Class
Following	Occasionally	Non Alcoholic	Regular	Middle Class
Not Following	Regular Exercise	Non Alcoholic	Regular	Poor
Not Following	No Exercise	Occasionally	Occasionally	High Class
Following	No Exercise	Non Alcoholic	Regular	Middle Class
Following	Regular Exercise	Non Alcoholic	Occasionally	Poor
Not Following	No Exercise	Occasionally	Regular	Poor
Following	Regular Exercise	Non Alcoholic	Regular	Poor
Following	Regular Exercise	Occasionally	Occasionally	Poor
Following	Regular Exercise	Occasionally	Regular	Poor
Following	Regular Exercise	Non Alcoholic	Regular	High Class
Not Following	Regular Exercise	Non Alcoholic	Regular	Poor
Not Following	Occasionally	Non Alcoholic	Occasionally	High Class
Not Following	No Exercise	Non Alcoholic	Regular	Poor
Following	No Exercise	Non Alcoholic	Regular	Poor
Not Following	No Exercise	Non Alcoholic	Regular	High Class
Following	Regular Exercise	Non Alcoholic	Regular	Middle Class
Following	No Exercise	Non Alcoholic	Occasionally	Middle Class
Not Following	No Exercise	Non Alcoholic	Regular	Middle Class
Not Following	No Exercise	Non Alcoholic	Regular	Poor
Following	Occasionally	Non Alcoholic	Occasionally	High Class
Not Following	Regular Exercise	Non Alcoholic	Regular	Middle Class
Following	Regular Exercise	Non Alcoholic	Occasionally	Poor
Following	Regular Exercise	Non Alcoholic	Regular	Poor
Not Following	Regular Exercise	Non Alcoholic	Regular	High Class
Not Following	Occasionally	Non Alcoholic	Occasionally	Poor
Not Following	Regular Exercise	Non Alcoholic	Regular	Poor
Not Following	Regular Exercise	Occasionally	Regular	Middle Class
Not Following	No Exercise	Occasionally	Regular	Poor
Not Following	Occasionally	Non Alcoholic	Occasionally	Poor

Following	No Exercise	Non Alcoholic	Regular	High Class
Not Following	Occasionally	Non Alcoholic	Regular	High Class
Not Following	No Exercise	Non Alcoholic	Regular	Poor
Following	No Exercise	Non Alcoholic	Regular	Poor
Not Following	Regular Exercise	Non Alcoholic	Occasionally	Poor
Not Following	Regular Exercise	Non Alcoholic	Regular	Poor
Following	No Exercise	Non Alcoholic	Regular	Poor
Not Following	Regular Exercise	Non Alcoholic	Non Smoker	High Class
Not Following	Regular Exercise	Occasionally	Regular	High Class
Not Following	Regular Exercise	Non Alcoholic	Occasionally	High Class
Not Following	Regular Exercise	Non Alcoholic	Regular	Middle Class
Following	Regular Exercise	Non Alcoholic	Regular	High Class
Following	Regular Exercise	Non Alcoholic	Occasionally	Poor
Following	No Exercise	Non Alcoholic	Regular	Poor
Following	No Exercise	Non Alcoholic	Regular	Poor
Following	Regular Exercise	Non Alcoholic	Regular	Poor
Not Following	No Exercise	Occasionally	Occasionally	High Class
Following	Regular Exercise	Non Alcoholic	Regular	High Class
Not Following	No Exercise	Non Alcoholic	Regular	High Class
Following	No Exercise	Non Alcoholic	Regular	High Class
Following	No Exercise	Non Alcoholic	Regular	Middle Class
Occasionally	Occasionally	Non Alcoholic	Occasionally	Middle Class
Not Following	Regular Exercise	Non Alcoholic	Regular	Middle Class
Not Following	Regular Exercise	Non Alcoholic	Regular	Middle Class
Not Following	No Exercise	Non Alcoholic	Occasionally	Middle Class
Occasionally	Regular Exercise	Non Alcoholic	Regular	High Class
Occasionally	No Exercise	Occasionally	Occasionally	High Class
Not Following	Regular Exercise	Non Alcoholic	Regular	High Class
Occasionally	No Exercise	Non Alcoholic	Non Smoker	Poor
Not Following	Regular Exercise	Non Alcoholic	Occasionally	Poor
Occasionally	No Exercise	Non Alcoholic	Regular	Middle Class
Occasionally	Regular Exercise	Non Alcoholic	Regular	Poor
Not Following	Regular Exercise	Non Alcoholic	Regular	Middle Class
Not Following	Regular Exercise	Occasionally	Occasionally	Poor
Occasionally	No Exercise	Non Alcoholic	Regular	Poor
Following	Regular Exercise	Non Alcoholic	Regular	Middle Class
Following	No Exercise	Non Alcoholic	Regular	Poor
Following	Regular Exercise	Non Alcoholic	Regular	High Class
Not Following	Regular Exercise	Non Alcoholic	Occasionally	High Class
Not Following	Regular Exercise	Non Alcoholic	Regular	High Class
Not Following	Regular Exercise	Non Alcoholic	Regular	Poor
Occasionally	Regular Exercise	Alcoholic	Occasionally	Middle Class
Occasionally	No Exercise	Non Alcoholic	Regular	High Class
Following	No Exercise	Non Alcoholic	Occasionally	Poor
Following	No Exercise	Non Alcoholic	Regular	Poor
Following	No Exercise	Non Alcoholic	Regular	Poor
Occasionally	Regular Exercise	Non Alcoholic	Occasionally	Poor

Occasionally	No Exercise	Non Alcoholic	Regular	Poor
Following	Regular Exercise	Non Alcoholic	Regular	Poor
Occasionally	No Exercise	Non Alcoholic	Regular	Middle Class
Occasionally	Regular Exercise	Non Alcoholic	Occasionally	Middle Class
Occasionally	No Exercise	Non Alcoholic	Regular	Middle Class
Occasionally	Regular Exercise	Non Alcoholic	Regular	High Class
Occasionally	No Exercise	Non Alcoholic	Regular	High Class
Occasionally	No Exercise	Non Alcoholic	Regular	High Class
Occasionally	Regular Exercise	Alcoholic	Occasionally	High Class
Occasionally	No Exercise	Non Alcoholic	Regular	High Class
Occasionally	Regular Exercise	Non Alcoholic	Regular	High Class
Occasionally	No Exercise	Non Alcoholic	Occasionally	Middle Class
Occasionally	Regular Exercise	Non Alcoholic	Regular	Poor
Following	Regular Exercise	Non Alcoholic	Occasionally	Poor
Not Following	No Exercise	Non Alcoholic	Regular	Poor
Not Following	Regular Exercise	Non Alcoholic	Regular	Poor
Not Following	No Exercise	Non Alcoholic	Occasionally	Middle Class
Following	Regular Exercise	Non Alcoholic	Regular	Middle Class
Not Following	No Exercise	Non Alcoholic	Regular	Middle Class
Following	Regular Exercise	Non Alcoholic	Regular	Middle Class
Not Following	No Exercise	Non Alcoholic	Occasionally	Middle Class
Following	Regular Exercise	Non Alcoholic	Regular	Middle Class
Following	Regular Exercise	Non Alcoholic	Regular	High Class
Not Following	No Exercise	Non Alcoholic	Regular	High Class
Not Following	Regular Exercise	Non Alcoholic	Regular	High Class
Following	Regular Exercise	Non Alcoholic	Occasionally	High Class
Following	No Exercise	Non Alcoholic	Regular	High Class
Following	Occasionally	Non Alcoholic	Regular	Poor
Not Following	Regular Exercise	Non Alcoholic	Regular	Poor
Not Following	No Exercise	Occasionally	Regular	Poor
Not Following	Regular Exercise	Occasionally	Regular	High Class
Not Following	No Exercise	Occasionally	Occasionally	High Class
Following	Regular Exercise	Non Alcoholic	Non Smoker	High Class
Following	Occasionally	Non Alcoholic	Regular	High Class
Following	Regular Exercise	Occasionally	Occasionally	High Class
Not Following	No Exercise	Non Alcoholic	Regular	Poor
Following	Regular Exercise	Non Alcoholic	Occasionally	Poor
Not Following	No Exercise	Occasionally	Regular	High Class
Following	Occasionally	Non Alcoholic	Regular	High Class
Not Following	Regular Exercise	Non Alcoholic	Occasionally	High Class
Not Following	No Exercise	Non Alcoholic	Regular	High Class
Following	No Exercise	Non Alcoholic	Regular	Middle Class
Not Following	Regular Exercise	Non Alcoholic	Regular	Middle Class
Following	Regular Exercise	Non Alcoholic	Occasionally	Middle Class
Not Following	Regular Exercise	Non Alcoholic	Regular	Middle Class
Not Following	Regular Exercise	Non Alcoholic	Regular	High Class
Following	Regular Exercise	Occasionally	Regular	Middle Class

Not Following	Occasionally	Non Alcoholic	Regular	Middle Class
Following	Occasionally	Non Alcoholic	Occasionally	High Class
Not Following	Occasionally	Non Alcoholic	Regular	Middle Class
Occasionally	Regular Exercise	Non Alcoholic	Regular	Middle Class
Occasionally	Occasionally	Non Alcoholic	Occasionally	Poor
Following	Occasionally	Non Alcoholic	Regular	Poor
Occasionally	Regular Exercise	Occasionally	Non Smoker	High Class
Following	No Exercise	Non Alcoholic	Regular	High Class
Occasionally	No Exercise	Non Alcoholic	Regular	High Class
Following	Regular Exercise	Non Alcoholic	Occasionally	High Class
Occasionally	Regular Exercise	Non Alcoholic	Regular	Poor
Following	Regular Exercise	Alcoholic	Regular	Poor
Not Following	Regular Exercise	Non Alcoholic	Regular	Poor
Not Following	Regular Exercise	Occasionally	Occasionally	High Class
Not Following	No Exercise	Non Alcoholic	Regular	High Class
Not Following	Regular Exercise	Non Alcoholic	Regular	High Class
Not Following	No Exercise	Non Alcoholic	Regular	High Class
Not Following	Regular Exercise	Non Alcoholic	Regular	High Class
Following	Regular Exercise	Non Alcoholic	Occasionally	Poor
Not Following	No Exercise	Non Alcoholic	Regular	High Class
Following	No Exercise	Non Alcoholic	Regular	Middle Class
Not Following	No Exercise	Non Alcoholic	Occasionally	Middle Class
Not Following	Regular Exercise	Non Alcoholic	Regular	Poor
Not Following	No Exercise	Occasionally	Occasionally	Middle Class
Following	Regular Exercise	Non Alcoholic	Regular	Poor
Following	No Exercise	Non Alcoholic	Regular	Poor
Occasionally	Regular Exercise	Non Alcoholic	Occasionally	High Class
Following	No Exercise	Non Alcoholic	Regular	Poor
Occasionally	Regular Exercise	Non Alcoholic	Regular	High Class
Not Following	No Exercise	Occasionally	Regular	Middle Class
Following	Regular Exercise	Non Alcoholic	Occasionally	Poor
Not Following	Regular Exercise	Non Alcoholic	Regular	Poor
Following	Regular Exercise	Non Alcoholic	Regular	High Class
Following	Regular Exercise	Non Alcoholic	Regular	High Class
Occasionally	Regular Exercise	Non Alcoholic	Regular	Middle Class
Occasionally	Regular Exercise	Alcoholic	Occasionally	High Class
Occasionally	No Exercise	Non Alcoholic	Regular	High Class
Occasionally	Regular Exercise	Non Alcoholic	Regular	High Class
Occasionally	No Exercise	Non Alcoholic	Occasionally	Poor
Occasionally	Regular Exercise	Non Alcoholic	Regular	Middle Class
Occasionally	No Exercise	Non Alcoholic	Occasionally	Poor
Occasionally	Regular Exercise	Occasionally	Regular	Middle Class
Occasionally	Regular Exercise	Non Alcoholic	Regular	Poor
Occasionally	Regular Exercise	Non Alcoholic	Occasionally	Poor
Occasionally	Regular Exercise	Non Alcoholic	Regular	Poor
Occasionally	No Exercise	Non Alcoholic	Regular	Middle Class
Not Following	No Exercise	Non Alcoholic	Regular	Middle Class

Following	No Exercise	Alcoholic	Occasionally	Middle Class
Not Following	Regular Exercise	Non Alcoholic	Regular	Middle Class
Not Following	No Exercise	Non Alcoholic	Regular	High Class
Following	Regular Exercise	Non Alcoholic	Regular	High Class
Occasionally	Regular Exercise	Non Alcoholic	Regular	High Class
Not Following	Regular Exercise	Non Alcoholic	Occasionally	High Class
Not Following	No Exercise	Non Alcoholic	Regular	High Class
Occasionally	No Exercise	Non Alcoholic	Regular	High Class
Occasionally	Regular Exercise	Occasionally	Occasionally	High Class
Not Following	No Exercise	Alcoholic	Non Smoker	Middle Class
Occasionally	No Exercise	Non Alcoholic	Occasionally	Middle Class
Following	Regular Exercise	Non Alcoholic	Regular	Middle Class
Following	Regular Exercise	Non Alcoholic	Regular	High Class
Not Following	No Exercise	Non Alcoholic	Occasionally	High Class
Following	No Exercise	Non Alcoholic	Regular	High Class
Following	Regular Exercise	Non Alcoholic	Regular	High Class
Following	No Exercise	Non Alcoholic	Regular	High Class
Following	Regular Exercise	Occasionally	Occasionally	High Class
Following	No Exercise	Non Alcoholic	Regular	High Class
Following	Regular Exercise	Non Alcoholic	Regular	Middle Class
Following	Regular Exercise	Non Alcoholic	Regular	Middle Class
Following	No Exercise	Occasionally	Regular	Middle Class
Following	No Exercise	Non Alcoholic	Occasionally	High Class
Following	No Exercise	Non Alcoholic	Regular	High Class
Following	Regular Exercise	Non Alcoholic	Regular	Middle Class
Not Following	No Exercise	Non Alcoholic	Occasionally	High Class
Not Following	Regular Exercise	Non Alcoholic	Regular	Middle Class
Following	No Exercise	Non Alcoholic	Occasionally	High Class
Not Following	Regular Exercise	Non Alcoholic	Regular	Poor
Following	Occasionally	Occasionally	Regular	Poor
Following	Regular Exercise	Non Alcoholic	Occasionally	Poor
Following	No Exercise	Non Alcoholic	Regular	Poor
Not Following	No Exercise	Non Alcoholic	Regular	Poor
Following	Regular Exercise	Non Alcoholic	Regular	Poor
Not Following	Regular Exercise	Non Alcoholic	Occasionally	Middle Class
Following	Regular Exercise	Non Alcoholic	Regular	Middle Class
Following	Regular Exercise	Occasionally	Regular	Middle Class
Not Following	Occasionally	Non Alcoholic	Regular	Middle Class
Not Following	No Exercise	Occasionally	Non Smoker	High Class
Not Following	Regular Exercise	Non Alcoholic	Occasionally	Poor
Following	Regular Exercise	Non Alcoholic	Regular	Poor
Following	Regular Exercise	Non Alcoholic	Regular	High Class
Following	No Exercise	Non Alcoholic	Occasionally	Middle Class
Not Following	Regular Exercise	Non Alcoholic	Regular	Middle Class
Not Following	Regular Exercise	Non Alcoholic	Occasionally	Middle Class
Following	Regular Exercise	Non Alcoholic	Regular	High Class
Following	Occasionally	Non Alcoholic	Regular	High Class

Not Following	No Exercise	Alcoholic	Occasionally	High Class
Not Following	No Exercise	Non Alcoholic	Regular	Poor
Not Following	No Exercise	Non Alcoholic	Regular	Poor
Not Following	Regular Exercise	Non Alcoholic	Regular	Poor
Occasionally	No Exercise	Non Alcoholic	Occasionally	High Class
Occasionally	Regular Exercise	Non Alcoholic	Regular	Poor
Not Following	No Exercise	Non Alcoholic	Regular	Poor
Not Following	Regular Exercise	Occasionally	Regular	Middle Class
Not Following	No Exercise	Non Alcoholic	Regular	High Class
Occasionally	Regular Exercise	Non Alcoholic	Occasionally	High Class
Not Following	Occasionally	Non Alcoholic	Regular	Poor
Not Following	Regular Exercise	Alcoholic	Regular	High Class
Following	No Exercise	Non Alcoholic	Non Smoker	High Class
Following	Regular Exercise	Non Alcoholic	Regular	High Class
Not Following	Regular Exercise	Non Alcoholic	Occasionally	Middle Class
Not Following	No Exercise	Non Alcoholic	Regular	High Class
Following	No Exercise	Non Alcoholic	Regular	Poor
Not Following	Regular Exercise	Non Alcoholic	Occasionally	High Class
Not Following	No Exercise	Non Alcoholic	Regular	Poor
Not Following	Regular Exercise	Non Alcoholic	Regular	Poor
Following	No Exercise	Non Alcoholic	Regular	High Class
Following	Regular Exercise	Non Alcoholic	Occasionally	Poor
Following	Regular Exercise	Non Alcoholic	Regular	Poor
Not Following	Regular Exercise	Occasionally	Regular	High Class
Not Following	No Exercise	Non Alcoholic	Regular	Middle Class
Occasionally	Regular Exercise	Non Alcoholic	Regular	High Class
Following	No Exercise	Non Alcoholic	Occasionally	Middle Class
Occasionally	No Exercise	Non Alcoholic	Regular	Middle Class
Occasionally	Regular Exercise	Non Alcoholic	Regular	High Class
Following	No Exercise	Non Alcoholic	Occasionally	High Class
Not Following	No Exercise	Non Alcoholic	Regular	Poor
Not Following	Regular Exercise	Non Alcoholic	Occasionally	Poor
Following	Regular Exercise	Non Alcoholic	Regular	Poor
Not Following	No Exercise	Non Alcoholic	Regular	Middle Class
Not Following	Regular Exercise	Non Alcoholic	Occasionally	High Class
Not Following	Regular Exercise	Non Alcoholic	Regular	High Class
Not Following	No Exercise	Non Alcoholic	Regular	High Class
Not Following	Regular Exercise	Non Alcoholic	Regular	Poor
Not Following	No Exercise	Non Alcoholic	Occasionally	Poor
Occasionally	Regular Exercise	Non Alcoholic	Regular	Poor
Occasionally	Regular Exercise	Non Alcoholic	Regular	High Class
Occasionally	No Exercise	Non Alcoholic	Regular	Poor
Occasionally	Regular Exercise	Alcoholic	Regular	Poor
Not Following	Regular Exercise	Non Alcoholic	Occasionally	High Class
Not Following	Regular Exercise	Non Alcoholic	Regular	Middle Class
Not Following	No Exercise	Occasionally	Regular	Middle Class
Not Following	Regular Exercise	Occasionally	Occasionally	High Class

Not Following	No Exercise	Non Alcoholic	Regular	High Class
Occasionally	Regular Exercise	Non Alcoholic	Occasionally	Poor
Not Following	No Exercise	Non Alcoholic	Regular	Poor
Following	Regular Exercise	Non Alcoholic	Regular	Poor
Following	No Exercise	Non Alcoholic	Occasionally	Middle Class
Following	Regular Exercise	Non Alcoholic	Regular	High Class
Following	Regular Exercise	Non Alcoholic	Regular	High Class
Not Following	Regular Exercise	Non Alcoholic	Regular	Middle Class
Occasionally	Regular Exercise	Non Alcoholic	Occasionally	Middle Class
Following	Occasionally	Non Alcoholic	Regular	Poor
Following	Regular Exercise	Occasionally	Occasionally	High Class
Not Following	No Exercise	Non Alcoholic	Regular	High Class
Not Following	No Exercise	Occasionally	Regular	High Class
Not Following	Regular Exercise	Non Alcoholic	Occasionally	High Class
Not Following	Regular Exercise	Non Alcoholic	Regular	High Class
Not Following	No Exercise	Non Alcoholic	Occasionally	Middle Class
Following	No Exercise	Non Alcoholic	Regular	Poor
Following	Occasionally	Occasionally	Regular	High Class
Following	No Exercise	Non Alcoholic	Occasionally	High Class
Following	Regular Exercise	Non Alcoholic	Regular	Middle Class
Following	No Exercise	Non Alcoholic	Regular	High Class
Not Following	Regular Exercise	Non Alcoholic	Regular	High Class
Following	Regular Exercise	Non Alcoholic	Occasionally	Poor
Occasionally	No Exercise	Non Alcoholic	Regular	Poor
Occasionally	Occasionally	Non Alcoholic	Non Smoker	Middle Class
Occasionally	No Exercise	Non Alcoholic	Regular	Middle Class
Occasionally	Regular Exercise	Non Alcoholic	Regular	Middle Class
Not Following	No Exercise	Non Alcoholic	Occasionally	Middle Class
Following	Regular Exercise	Occasionally	Regular	Middle Class
Following	No Exercise	Non Alcoholic	Regular	High Class
Following	Regular Exercise	Non Alcoholic	Occasionally	High Class
Following	Occasionally	Non Alcoholic	Regular	Middle Class
Not Following	Regular Exercise	Non Alcoholic	Occasionally	Middle Class
Not Following	Occasionally	Non Alcoholic	Regular	High Class
Not Following	No Exercise	Non Alcoholic	Regular	Poor
Following	Regular Exercise	Non Alcoholic	Occasionally	Poor
Following	Regular Exercise	Non Alcoholic	Regular	High Class
Following	Regular Exercise	Non Alcoholic	Regular	High Class
Not Following	Regular Exercise	Occasionally	Regular	Poor
Following	Occasionally	Non Alcoholic	Occasionally	Middle Class
Following	No Exercise	Non Alcoholic	Regular	Poor
Following	Regular Exercise	Non Alcoholic	Regular	Poor
Following	Regular Exercise	Non Alcoholic	Regular	Middle Class
Not Following	Regular Exercise	Non Alcoholic	Regular	Poor
Occasionally	No Exercise	Non Alcoholic	Occasionally	Middle Class
Not Following	Regular Exercise	Occasionally	Non Smoker	Middle Class
Not Following	Regular Exercise	Non Alcoholic	Regular	Poor

Occasionally	Occasionally	Non Alcoholic	Occasionally	Middle Class
Occasionally	Regular Exercise	Non Alcoholic	Regular	Poor
Following	No Exercise	Non Alcoholic	Occasionally	Poor
Following	No Exercise	Non Alcoholic	Regular	High Class
Not Following	No Exercise	Non Alcoholic	Regular	Poor
Occasionally	Regular Exercise	Non Alcoholic	Occasionally	High Class
Occasionally	No Exercise	Occasionally	Regular	High Class
Following	Occasionally	Non Alcoholic	Regular	High Class
Occasionally	Occasionally	Non Alcoholic	Regular	Middle Class
Following	Regular Exercise	Non Alcoholic	Occasionally	Middle Class
Occasionally	No Exercise	Occasionally	Regular	Middle Class
Following	Regular Exercise	Non Alcoholic	Regular	Middle Class
Occasionally	No Exercise	Non Alcoholic	Regular	Middle Class
Not Following	Regular Exercise	Non Alcoholic	Regular	Poor
Following	Regular Exercise	Non Alcoholic	Occasionally	Poor
Not Following	Regular Exercise	Non Alcoholic	Regular	High Class
Following	Regular Exercise	Non Alcoholic	Regular	Poor
Following	Regular Exercise	Occasionally	Regular	Poor
Not Following	Regular Exercise	Non Alcoholic	Regular	High Class
Not Following	Regular Exercise	Non Alcoholic	Non Smoker	High Class
Following	Regular Exercise	Non Alcoholic	Regular	High Class
Not Following	No Exercise	Non Alcoholic	Regular	Poor
Not Following	Regular Exercise	Non Alcoholic	Occasionally	High Class
Not Following	No Exercise	Non Alcoholic	Non Smoker	Poor
Not Following	Regular Exercise	Non Alcoholic	Occasionally	Poor
Occasionally	No Exercise	Non Alcoholic	Regular	High Class
Occasionally	Regular Exercise	Non Alcoholic	Regular	Poor
Not Following	No Exercise	Non Alcoholic	Occasionally	Poor
Not Following	Regular Exercise	Non Alcoholic	Regular	Middle Class
Occasionally	No Exercise	Occasionally	Regular	High Class
Occasionally	No Exercise	Occasionally	Regular	Middle Class
Occasionally	Regular Exercise	Non Alcoholic	Occasionally	Poor
Occasionally	Regular Exercise	Non Alcoholic	Regular	Poor
Occasionally	No Exercise	Non Alcoholic	Regular	High Class
Occasionally	Regular Exercise	Non Alcoholic	Regular	High Class
Occasionally	No Exercise	Non Alcoholic	Regular	High Class
Occasionally	No Exercise	Non Alcoholic	Occasionally	High Class
Occasionally	Regular Exercise	Non Alcoholic	Regular	High Class
Occasionally	Regular Exercise	Non Alcoholic	Regular	High Class
Occasionally	No Exercise	Non Alcoholic	Occasionally	High Class
Occasionally	No Exercise	Non Alcoholic	Regular	Poor
Occasionally	Regular Exercise	Non Alcoholic	Occasionally	Poor
Occasionally	No Exercise	Non Alcoholic	Regular	Poor
Occasionally	Regular Exercise	Alcoholic	Regular	High Class
Occasionally	Occasionally	Non Alcoholic	Occasionally	High Class
Not Following	Regular Exercise	Non Alcoholic	Regular	Poor
Following	No Exercise	Non Alcoholic	Regular	Poor

Following	Regular Exercise	Non Alcoholic	Regular	High Class
Occasionally	No Exercise	Non Alcoholic	Occasionally	Poor
Following	Regular Exercise	Non Alcoholic	Regular	High Class
Following	No Exercise	Non Alcoholic	Regular	Poor
Not Following	Regular Exercise	Non Alcoholic	Regular	Poor
Not Following	Regular Exercise	Non Alcoholic	Regular	High Class
Following	Occasionally	Non Alcoholic	Occasionally	Middle Class
Not Following	Regular Exercise	Non Alcoholic	Regular	Poor
Not Following	Regular Exercise	Alcoholic	Regular	High Class
Following	Regular Exercise	Non Alcoholic	Occasionally	High Class
Following	No Exercise	Non Alcoholic	Regular	Poor
Following	Occasionally	Non Alcoholic	Occasionally	High Class
Following	No Exercise	Non Alcoholic	Regular	Poor
Not Following	Regular Exercise	Non Alcoholic	Regular	Poor
Following	Regular Exercise	Non Alcoholic	Occasionally	Poor
Following	Regular Exercise	Non Alcoholic	Regular	Poor
Following	Regular Exercise	Non Alcoholic	Regular	Middle Class
Occasionally	Occasionally	Non Alcoholic	Regular	Middle Class
Following	Regular Exercise	Non Alcoholic	Occasionally	Poor
Following	Regular Exercise	Non Alcoholic	Regular	High Class
Occasionally	No Exercise	Occasionally	Regular	High Class
Following	Regular Exercise	Non Alcoholic	Regular	Middle Class
Following	No Exercise	Non Alcoholic	Non Smoker	High Class
Occasionally	Regular Exercise	Alcoholic	Occasionally	Poor
Following	No Exercise	Non Alcoholic	Regular	Poor
Following	Regular Exercise	Non Alcoholic	Regular	Poor
Occasionally	Regular Exercise	Non Alcoholic	Occasionally	Poor
Not Following	No Exercise	Non Alcoholic	Regular	Poor
Following	Regular Exercise	Non Alcoholic	Occasionally	High Class
Not Following	No Exercise	Non Alcoholic	Regular	Poor
Not Following	Regular Exercise	Non Alcoholic	Regular	Poor
Following	No Exercise	Non Alcoholic	Occasionally	Middle Class
Occasionally	No Exercise	Non Alcoholic	Regular	Middle Class
Not Following	No Exercise	Non Alcoholic	Regular	High Class
Occasionally	Regular Exercise	Non Alcoholic	Regular	Poor
Not Following	No Exercise	Non Alcoholic	Occasionally	Middle Class
Not Following	Regular Exercise	Non Alcoholic	Regular	High Class
Not Following	No Exercise	Non Alcoholic	Regular	Poor
Occasionally	Regular Exercise	Occasionally	Regular	High Class
Occasionally	Regular Exercise	Occasionally	Regular	Middle Class
Following	Regular Exercise	Non Alcoholic	Occasionally	Middle Class
Following	Regular Exercise	Non Alcoholic	Regular	Middle Class
Not Following	Regular Exercise	Non Alcoholic	Regular	High Class
Not Following	No Exercise	Non Alcoholic	Occasionally	Poor
Not Following	Regular Exercise	Non Alcoholic	Regular	High Class
Occasionally	Regular Exercise	Non Alcoholic	Occasionally	High Class
Not Following	No Exercise	Non Alcoholic	Regular	Middle Class

Occasionally	Regular Exercise	Non Alcoholic	Regular	High Class
Not Following	No Exercise	Non Alcoholic	Occasionally	High Class
Following	Regular Exercise	Non Alcoholic	Regular	High Class
Not Following	No Exercise	Non Alcoholic	Regular	Middle Class
Following	Regular Exercise	Non Alcoholic	Regular	High Class
Following	No Exercise	Non Alcoholic	Occasionally	Poor
Following	Regular Exercise	Non Alcoholic	Regular	Poor
Not Following	Regular Exercise	Non Alcoholic	Occasionally	Middle Class
Occasionally	No Exercise	Alcoholic	Regular	Poor
Following	Regular Exercise	Non Alcoholic	Regular	Poor
Not Following	No Exercise	Non Alcoholic	Occasionally	Poor
Not Following	Occasionally	Non Alcoholic	Regular	Poor
Not Following	No Exercise	Non Alcoholic	Regular	High Class
Not Following	No Exercise	Non Alcoholic	Regular	High Class
Not Following	Regular Exercise	Non Alcoholic	Occasionally	High Class
Following	Regular Exercise	Non Alcoholic	Regular	Poor
Following	Occasionally	Alcoholic	Regular	High Class
Following	No Exercise	Non Alcoholic	Regular	Poor
Following	No Exercise	Non Alcoholic	Regular	Middle Class
Not Following	Regular Exercise	Non Alcoholic	Occasionally	Middle Class
Following	No Exercise	Occasionally	Regular	Middle Class
Following	Regular Exercise	Non Alcoholic	Regular	Middle Class
Not Following	No Exercise	Non Alcoholic	Occasionally	Middle Class
Following	Regular Exercise	Non Alcoholic	Regular	Middle Class
Not Following	Regular Exercise	Non Alcoholic	Occasionally	High Class
Occasionally	Regular Exercise	Non Alcoholic	Regular	High Class
Occasionally	Regular Exercise	Non Alcoholic	Regular	High Class
Occasionally	Regular Exercise	Non Alcoholic	Occasionally	High Class
Occasionally	No Exercise	Non Alcoholic	Regular	Poor
Occasionally	No Exercise	Non Alcoholic	Regular	High Class
Occasionally	No Exercise	Non Alcoholic	Regular	Middle Class
Occasionally	Regular Exercise	Occasionally	Occasionally	Poor
Occasionally	No Exercise	Non Alcoholic	Regular	Poor
Occasionally	Regular Exercise	Non Alcoholic	Regular	Poor
Occasionally	No Exercise	Non Alcoholic	Regular	Poor
Occasionally	Regular Exercise	Non Alcoholic	Regular	Middle Class
Occasionally	No Exercise	Non Alcoholic	Occasionally	Poor
Occasionally	No Exercise	Non Alcoholic	Regular	High Class
Following	Regular Exercise	Non Alcoholic	Regular	High Class
Following	Regular Exercise	Non Alcoholic	Occasionally	Middle Class
Occasionally	Regular Exercise	Non Alcoholic	Regular	Middle Class
Following	No Exercise	Non Alcoholic	Occasionally	Middle Class
Occasionally	Regular Exercise	Non Alcoholic	Regular	Middle Class
Occasionally	No Exercise	Occasionally	Regular	Middle Class
Occasionally	Regular Exercise	Non Alcoholic	Occasionally	High Class
Not Following	Regular Exercise	Non Alcoholic	Regular	Middle Class
Not Following	No Exercise	Non Alcoholic	Regular	Poor

Following	Regular Exercise	Non Alcoholic	Regular	High Class
Not Following	Regular Exercise	Non Alcoholic	Occasionally	Poor
Following	No Exercise	Non Alcoholic	Regular	Middle Class
Following	Regular Exercise	Non Alcoholic	Non Smoker	High Class
Not Following	No Exercise	Non Alcoholic	Regular	Middle Class
Following	Regular Exercise	Non Alcoholic	Regular	Poor
Not Following	Regular Exercise	Non Alcoholic	Occasionally	Poor
Not Following	Regular Exercise	Non Alcoholic	Regular	Middle Class
Not Following	Regular Exercise	Alcoholic	Regular	Middle Class
Following	No Exercise	Non Alcoholic	Occasionally	Middle Class
Not Following	Regular Exercise	Non Alcoholic	Regular	High Class
Following	No Exercise	Non Alcoholic	Occasionally	High Class
Following	Regular Exercise	Non Alcoholic	Regular	High Class
Not Following	Regular Exercise	Non Alcoholic	Regular	Middle Class
Following	No Exercise	Non Alcoholic	Occasionally	Middle Class
Following	Regular Exercise	Non Alcoholic	Regular	Middle Class
Following	No Exercise	Non Alcoholic	Regular	Middle Class
Not Following	Regular Exercise	Non Alcoholic	Regular	Poor
Occasionally	Regular Exercise	Non Alcoholic	Occasionally	Poor
Occasionally	Occasionally	Non Alcoholic	Regular	Poor
Following	No Exercise	Non Alcoholic	Regular	High Class
Following	Occasionally	Non Alcoholic	Regular	High Class
Occasionally	No Exercise	Alcoholic	Regular	High Class
Occasionally	Regular Exercise	Non Alcoholic	Occasionally	High Class
Following	Regular Exercise	Non Alcoholic	Regular	Middle Class
Not Following	Regular Exercise	Non Alcoholic	Regular	High Class
Not Following	No Exercise	Non Alcoholic	Occasionally	Middle Class
Not Following	No Exercise	Non Alcoholic	Regular	High Class
Not Following	No Exercise	Non Alcoholic	Occasionally	Middle Class
Following	No Exercise	Non Alcoholic	Regular	High Class
Not Following	Regular Exercise	Non Alcoholic	Regular	Poor
Following	Regular Exercise	Non Alcoholic	Occasionally	Poor
Following	Regular Exercise	Non Alcoholic	Non Smoker	Middle Class
Following	No Exercise	Non Alcoholic	Regular	Middle Class
Following	No Exercise	Non Alcoholic	Regular	Middle Class
Occasionally	Regular Exercise	Occasionally	Occasionally	Middle Class
Following	Regular Exercise	Occasionally	Regular	High Class
Following	Occasionally	Alcoholic	Regular	High Class
Occasionally	Regular Exercise	Non Alcoholic	Regular	High Class
Occasionally	No Exercise	Non Alcoholic	Regular	High Class
Following	Regular Exercise	Non Alcoholic	Occasionally	High Class
Not Following	Regular Exercise	Non Alcoholic	Regular	Middle Class
Not Following	No Exercise	Non Alcoholic	Regular	Middle Class
Following	Regular Exercise	Non Alcoholic	Occasionally	Poor
Not Following	No Exercise	Non Alcoholic	Regular	Middle Class
Not Following	Regular Exercise	Non Alcoholic	Occasionally	Poor
Not Following	No Exercise	Occasionally	Regular	High Class

Not Following	Regular Exercise	Non Alcoholic	Regular	High Class
Following	Regular Exercise	Non Alcoholic	Occasionally	Poor
Not Following	No Exercise	Non Alcoholic	Regular	High Class
Following	Regular Exercise	Non Alcoholic	Regular	High Class
Not Following	Regular Exercise	Non Alcoholic	Regular	High Class
Following	Regular Exercise	Non Alcoholic	Occasionally	High Class
Following	Regular Exercise	Non Alcoholic	Non Smoker	High Class
Not Following	No Exercise	Non Alcoholic	Regular	Poor
Following	No Exercise	Non Alcoholic	Regular	Middle Class
Following	Regular Exercise	Non Alcoholic	Regular	Middle Class
Not Following	No Exercise	Non Alcoholic	Occasionally	Poor
Following	Regular Exercise	Non Alcoholic	Regular	Poor
Not Following	Regular Exercise	Non Alcoholic	Regular	Poor
Following	Regular Exercise	Alcoholic	Occasionally	Middle Class
Not Following	Regular Exercise	Non Alcoholic	Regular	Poor
Not Following	Regular Exercise	Non Alcoholic	Occasionally	Poor
Not Following	No Exercise	Non Alcoholic	Regular	Poor
Following	Regular Exercise	Non Alcoholic	Regular	Poor
Not Following	No Exercise	Non Alcoholic	Occasionally	Poor
Not Following	Regular Exercise	Non Alcoholic	Regular	High Class
Following	Regular Exercise	Non Alcoholic	Regular	Middle Class
Following	Regular Exercise	Non Alcoholic	Regular	Middle Class
Not Following	Regular Exercise	Non Alcoholic	Occasionally	Middle Class
Following	Regular Exercise	Non Alcoholic	Regular	High Class
Following	Regular Exercise	Non Alcoholic	Regular	High Class
Following	No Exercise	Alcoholic	Regular	High Class
Not Following	Regular Exercise	Non Alcoholic	Regular	Middle Class
Following	Regular Exercise	Non Alcoholic	Occasionally	Middle Class
Not Following	Regular Exercise	Non Alcoholic	Regular	Middle Class
Following	No Exercise	Non Alcoholic	Regular	Middle Class
Following	No Exercise	Non Alcoholic	Occasionally	Middle Class
Not Following	Regular Exercise	Non Alcoholic	Regular	Middle Class
Not Following	Regular Exercise	Non Alcoholic	Occasionally	Poor
Following	Regular Exercise	Non Alcoholic	Regular	Poor
Not Following	No Exercise	Non Alcoholic	Regular	High Class
Following	No Exercise	Non Alcoholic	Occasionally	High Class
Not Following	Regular Exercise	Non Alcoholic	Regular	High Class
Not Following	Regular Exercise	Alcoholic	Regular	Poor
Following	Regular Exercise	Non Alcoholic	Regular	Poor
Not Following	Regular Exercise	Non Alcoholic	Occasionally	Poor
Not Following	Regular Exercise	Non Alcoholic	Regular	Poor
Occasionally	Regular Exercise	Alcoholic	Regular	Poor
Not Following	Regular Exercise	Non Alcoholic	Regular	High Class
Not Following	No Exercise	Non Alcoholic	Regular	Middle Class
Not Following	Regular Exercise	Non Alcoholic	Occasionally	Middle Class
Occasionally	No Exercise	Non Alcoholic	Regular	Middle Class
Occasionally	Regular Exercise	Non Alcoholic	Regular	Middle Class

Occasionally	Regular Exercise	Non Alcoholic	Occasionally	Middle Class
Not Following	Regular Exercise	Non Alcoholic	Regular	Middle Class
Not Following	Regular Exercise	Non Alcoholic	Occasionally	Middle Class
Occasionally	Regular Exercise	Non Alcoholic	Regular	Middle Class
Not Following	No Exercise	Non Alcoholic	Regular	Middle Class
Not Following	Regular Exercise	Non Alcoholic	Occasionally	Middle Class
Occasionally	Regular Exercise	Occasionally	Regular	Middle Class
Occasionally	Regular Exercise	Non Alcoholic	Regular	Middle Class
Not Following	No Exercise	Non Alcoholic	Regular	Middle Class
Not Following	Regular Exercise	Non Alcoholic	Non Smoker	Poor
Following	Occasionally	Alcoholic	Regular	Poor
Following	Regular Exercise	Non Alcoholic	Regular	Poor
Not Following	Regular Exercise	Non Alcoholic	Regular	Poor
Not Following	Occasionally	Non Alcoholic	Regular	Poor
Not Following	Regular Exercise	Non Alcoholic	Occasionally	Poor
Not Following	Regular Exercise	Non Alcoholic	Regular	Poor
Not Following	Regular Exercise	Non Alcoholic	Regular	Poor
Following	Regular Exercise	Non Alcoholic	Occasionally	High Class
Not Following	No Exercise	Occasionally	Regular	High Class
Following	Regular Exercise	Non Alcoholic	Occasionally	High Class
Not Following	Regular Exercise	Non Alcoholic	Regular	High Class
Following	No Exercise	Non Alcoholic	Regular	High Class
Following	Occasionally	Non Alcoholic	Occasionally	High Class
Following	Occasionally	Non Alcoholic	Regular	Middle Class
Not Following	No Exercise	Non Alcoholic	Regular	Middle Class
Following	Occasionally	Non Alcoholic	Regular	Middle Class
Following	Regular Exercise	Occasionally	Occasionally	High Class
Following	No Exercise	Non Alcoholic	Regular	High Class
Occasionally	Regular Exercise	Non Alcoholic	Regular	High Class
Following	No Exercise	Non Alcoholic	Regular	Poor
Occasionally	Regular Exercise	Alcoholic	Regular	Poor
Following	Regular Exercise	Non Alcoholic	Occasionally	Middle Class
Occasionally	Regular Exercise	Non Alcoholic	Regular	High Class
Occasionally	No Exercise	Non Alcoholic	Regular	Middle Class
Not Following	Regular Exercise	Non Alcoholic	Regular	Middle Class
Not Following	Regular Exercise	Occasionally	Regular	Middle Class
Following	Regular Exercise	Occasionally	Occasionally	Middle Class
Not Following	Regular Exercise	Non Alcoholic	Regular	High Class
Not Following	Regular Exercise	Non Alcoholic	Regular	High Class
Not Following	No Exercise	Non Alcoholic	Non Smoker	High Class
Occasionally	Regular Exercise	Non Alcoholic	Occasionally	Poor
Following	Regular Exercise	Non Alcoholic	Regular	Poor
Following	Regular Exercise	Non Alcoholic	Regular	Middle Class
Following	Regular Exercise	Occasionally	Regular	Poor
Following	No Exercise	Occasionally	Regular	Middle Class
Not Following	Regular Exercise	Non Alcoholic	Occasionally	Poor
Following	No Exercise	Non Alcoholic	Regular	Poor

Not Following	Regular Exercise	Non Alcoholic	Regular	Middle Class
Not Following	No Exercise	Non Alcoholic	Occasionally	Poor
Not Following	Regular Exercise	Non Alcoholic	Regular	High Class
Following	Regular Exercise	Non Alcoholic	Occasionally	High Class
Not Following	Regular Exercise	Non Alcoholic	Regular	High Class
Following	Regular Exercise	Non Alcoholic	Regular	Poor
Not Following	Regular Exercise	Non Alcoholic	Occasionally	Middle Class
Following	Regular Exercise	Non Alcoholic	Regular	High Class
Not Following	No Exercise	Non Alcoholic	Regular	Poor
Not Following	Regular Exercise	Non Alcoholic	Regular	Poor
Following	Regular Exercise	Non Alcoholic	Occasionally	Poor
Not Following	Regular Exercise	Non Alcoholic	Regular	Poor
Following	No Exercise	Non Alcoholic	Regular	Poor
Following	No Exercise	Non Alcoholic	Regular	Poor
Not Following	Regular Exercise	Non Alcoholic	Regular	Middle Class
Not Following	Regular Exercise	Occasionally	Occasionally	Middle Class
Not Following	No Exercise	Non Alcoholic	Regular	Middle Class
Not Following	Regular Exercise	Occasionally	Non Smoker	High Class
Not Following	No Exercise	Non Alcoholic	Occasionally	High Class
Not Following	Regular Exercise	Non Alcoholic	Regular	High Class
Not Following	No Exercise	Non Alcoholic	Occasionally	High Class
Following	Regular Exercise	Non Alcoholic	Regular	High Class
Following	Regular Exercise	Non Alcoholic	Regular	High Class
Not Following	Regular Exercise	Non Alcoholic	Occasionally	Middle Class
Not Following	No Exercise	Non Alcoholic	Regular	Poor
Following	No Exercise	Non Alcoholic	Regular	Poor
Following	No Exercise	Non Alcoholic	Regular	Poor
Following	Regular Exercise	Alcoholic	Occasionally	Poor
Following	Regular Exercise	Non Alcoholic	Regular	Middle Class
Following	No Exercise	Non Alcoholic	Regular	Middle Class
Not Following	Regular Exercise	Non Alcoholic	Regular	Middle Class
Not Following	Regular Exercise	Non Alcoholic	Regular	Middle Class
Following	Regular Exercise	Non Alcoholic	Occasionally	Middle Class
Not Following	No Exercise	Non Alcoholic	Regular	Middle Class
Not Following	No Exercise	Alcoholic	Regular	High Class
Not Following	Regular Exercise	Non Alcoholic	Occasionally	High Class
Not Following	Regular Exercise	Non Alcoholic	Regular	High Class
Following	Regular Exercise	Non Alcoholic	Occasionally	High Class
Not Following	No Exercise	Non Alcoholic	Regular	High Class
Following	Occasionally	Non Alcoholic	Regular	High Class
Not Following	No Exercise	Non Alcoholic	Occasionally	High Class
Not Following	No Exercise	Non Alcoholic	Regular	High Class
Following	No Exercise	Non Alcoholic	Non Smoker	Poor
Occasionally	Regular Exercise	Non Alcoholic	Regular	Poor
Occasionally	Occasionally	Non Alcoholic	Occasionally	Poor
Not Following	Regular Exercise	Non Alcoholic	Regular	Middle Class
Not Following	Regular Exercise	Occasionally	Non Smoker	Poor

Not Following	No Exercise	Non Alcoholic	Regular	Poor
Occasionally	Regular Exercise	Non Alcoholic	Regular	High Class
Not Following	Regular Exercise	Non Alcoholic	Occasionally	Poor
Not Following	No Exercise	Non Alcoholic	Regular	Poor
Occasionally	Regular Exercise	Non Alcoholic	Regular	Middle Class
Following	No Exercise	Non Alcoholic	Occasionally	Poor
Following	Regular Exercise	Non Alcoholic	Regular	Poor
Occasionally	No Exercise	Non Alcoholic	Occasionally	High Class
Not Following	Regular Exercise	Non Alcoholic	Regular	Poor
Occasionally	Occasionally	Non Alcoholic	Regular	Poor
Occasionally	Regular Exercise	Non Alcoholic	Occasionally	Middle Class
Occasionally	Regular Exercise	Non Alcoholic	Regular	Poor
Occasionally	Regular Exercise	Non Alcoholic	Regular	Poor
Following	Regular Exercise	Non Alcoholic	Regular	Middle Class
Occasionally	Regular Exercise	Non Alcoholic	Occasionally	Poor
Occasionally	No Exercise	Non Alcoholic	Regular	Poor
Following	Occasionally	Non Alcoholic	Regular	Middle Class
Occasionally	No Exercise	Alcoholic	Regular	Middle Class
Not Following	No Exercise	Non Alcoholic	Regular	Middle Class
Not Following	Regular Exercise	Non Alcoholic	Occasionally	Middle Class
Not Following	Regular Exercise	Non Alcoholic	Non Smoker	Poor
Following	No Exercise	Non Alcoholic	Regular	Poor
Following	Regular Exercise	Alcoholic	Occasionally	Poor
Following	No Exercise	Non Alcoholic	Regular	High Class
Following	No Exercise	Non Alcoholic	Occasionally	High Class
Not Following	No Exercise	Non Alcoholic	Regular	High Class
Following	Occasionally	Non Alcoholic	Regular	High Class
Occasionally	Regular Exercise	Non Alcoholic	Occasionally	High Class
Occasionally	Regular Exercise	Non Alcoholic	Regular	Poor
Occasionally	No Exercise	Non Alcoholic	Regular	Poor
Following	Regular Exercise	Alcoholic	Regular	High Class
Not Following	No Exercise	Non Alcoholic	Occasionally	High Class
Not Following	Regular Exercise	Non Alcoholic	Regular	High Class
Following	No Exercise	Non Alcoholic	Regular	High Class
Following	Regular Exercise	Non Alcoholic	Regular	Middle Class
Following	No Exercise	Non Alcoholic	Regular	Middle Class
Not Following	Occasionally	Non Alcoholic	Occasionally	Middle Class
Following	No Exercise	Non Alcoholic	Regular	Middle Class
Following	No Exercise	Non Alcoholic	Regular	High Class
Not Following	Occasionally	Non Alcoholic	Occasionally	Middle Class
Not Following	No Exercise	Non Alcoholic	Regular	Middle Class
Not Following	Occasionally	Occasionally	Occasionally	High Class
Following	Regular Exercise	Non Alcoholic	Regular	Middle Class
Following	No Exercise	Non Alcoholic	Regular	Middle Class
Following	No Exercise	Non Alcoholic	Occasionally	Poor
Not Following	Occasionally	Non Alcoholic	Non Smoker	Poor
Following	No Exercise	Non Alcoholic	Regular	High Class

Not Following	No Exercise	Occasionally	Regular	High Class
Not Following	Occasionally	Non Alcoholic	Occasionally	High Class
Not Following	Regular Exercise	Non Alcoholic	Regular	High Class
Following	Regular Exercise	Non Alcoholic	Regular	Poor
Not Following	Regular Exercise	Non Alcoholic	Regular	Poor
Following	No Exercise	Non Alcoholic	Regular	Poor
Following	No Exercise	Non Alcoholic	Occasionally	High Class
Following	Regular Exercise	Non Alcoholic	Regular	High Class
Not Following	Regular Exercise	Non Alcoholic	Regular	High Class
Following	Regular Exercise	Non Alcoholic	Occasionally	High Class
Not Following	Regular Exercise	Non Alcoholic	Regular	High Class
Not Following	Occasionally	Non Alcoholic	Occasionally	Poor
Following	Regular Exercise	Alcoholic	Regular	High Class
Following	Regular Exercise	Non Alcoholic	Regular	Middle Class
Following	Regular Exercise	Non Alcoholic	Occasionally	Middle Class
Following	Regular Exercise	Non Alcoholic	Regular	Poor
Following	No Exercise	Non Alcoholic	Regular	Middle Class
Following	Regular Exercise	Non Alcoholic	Regular	Poor
Not Following	Regular Exercise	Non Alcoholic	Occasionally	Poor
Following	Occasionally	Non Alcoholic	Regular	High Class
Following	No Exercise	Non Alcoholic	Regular	Poor
Following	Regular Exercise	Non Alcoholic	Regular	High Class
Following	Regular Exercise	Non Alcoholic	Regular	Middle Class
Following	No Exercise	Non Alcoholic	Non Smoker	Poor
Following	Regular Exercise	Non Alcoholic	Regular	Poor
Following	No Exercise	Non Alcoholic	Regular	High Class
Not Following	Regular Exercise	Non Alcoholic	Occasionally	High Class
Not Following	No Exercise	Non Alcoholic	Non Smoker	Middle Class
Not Following	No Exercise	Occasionally	Occasionally	High Class
Not Following	Regular Exercise	Non Alcoholic	Regular	High Class
Following	Regular Exercise	Non Alcoholic	Regular	High Class
Following	No Exercise	Non Alcoholic	Occasionally	Poor
Occasionally	Occasionally	Non Alcoholic	Regular	Middle Class
Following	No Exercise	Non Alcoholic	Regular	Poor
Following	Regular Exercise	Non Alcoholic	Regular	Middle Class
Not Following	No Exercise	Non Alcoholic	Occasionally	Poor
Following	Regular Exercise	Non Alcoholic	Regular	Poor
Following	Occasionally	Non Alcoholic	Regular	Poor
Following	Regular Exercise	Occasionally	Regular	Middle Class
Not Following	Regular Exercise	Non Alcoholic	Regular	Middle Class
Not Following	No Exercise	Non Alcoholic	Occasionally	Middle Class
Following	No Exercise	Non Alcoholic	Regular	Middle Class
Not Following	Regular Exercise	Non Alcoholic	Regular	High Class
Following	No Exercise	Non Alcoholic	Occasionally	High Class
Not Following	Regular Exercise	Non Alcoholic	Regular	High Class
Following	Regular Exercise	Non Alcoholic	Occasionally	High Class
Not Following	Regular Exercise	Alcoholic	Regular	High Class

Following	Regular Exercise	Non Alcoholic	Regular	High Class
Not Following	Regular Exercise	Non Alcoholic	Non Smoker	High Class
Following	Regular Exercise	Non Alcoholic	Regular	Middle Class
Occasionally	Regular Exercise	Non Alcoholic	Regular	Middle Class
Not Following	Regular Exercise	Non Alcoholic	Regular	Middle Class
Occasionally	Regular Exercise	Non Alcoholic	Occasionally	High Class
Not Following	Regular Exercise	Non Alcoholic	Regular	High Class
Not Following	Regular Exercise	Non Alcoholic	Regular	High Class
Occasionally	Regular Exercise	Non Alcoholic	Regular	High Class
Not Following	Regular Exercise	Non Alcoholic	Regular	High Class
Occasionally	Regular Exercise	Non Alcoholic	Occasionally	High Class
Not Following	Regular Exercise	Non Alcoholic	Regular	High Class
Not Following	Regular Exercise	Alcoholic	Regular	Middle Class
Occasionally	Regular Exercise	Non Alcoholic	Occasionally	Middle Class
Not Following	Regular Exercise	Non Alcoholic	Regular	Middle Class
Occasionally	Regular Exercise	Non Alcoholic	Occasionally	High Class
Not Following	Regular Exercise	Non Alcoholic	Regular	High Class
Occasionally	No Exercise	Non Alcoholic	Regular	Middle Class
Occasionally	Occasionally	Non Alcoholic	Occasionally	High Class
Not Following	No Exercise	Alcoholic	Regular	Middle Class
Not Following	No Exercise	Non Alcoholic	Regular	High Class
Occasionally	Regular Exercise	Non Alcoholic	Regular	Poor
Occasionally	Regular Exercise	Non Alcoholic	Occasionally	Poor
Not Following	Regular Exercise	Non Alcoholic	Regular	Poor
Not Following	Regular Exercise	Alcoholic	Regular	Poor
Not Following	Regular Exercise	Non Alcoholic	Regular	Poor
Not Following	No Exercise	Non Alcoholic	Non Smoker	Poor
Not Following	Regular Exercise	Non Alcoholic	Occasionally	Middle Class
Not Following	No Exercise	Non Alcoholic	Regular	Middle Class
Following	Regular Exercise	Non Alcoholic	Regular	Middle Class
Following	Occasionally	Non Alcoholic	Occasionally	Middle Class
Following	Regular Exercise	Non Alcoholic	Regular	High Class
Not Following	Regular Exercise	Non Alcoholic	Occasionally	Poor
Not Following	No Exercise	Alcoholic	Regular	Poor
Following	No Exercise	Non Alcoholic	Regular	High Class
Following	No Exercise	Non Alcoholic	Occasionally	Middle Class
Not Following	No Exercise	Alcoholic	Regular	Middle Class
Occasionally	Regular Exercise	Alcoholic	Regular	Middle Class
Following	Regular Exercise	Non Alcoholic	Regular	High Class
Occasionally	Regular Exercise	Non Alcoholic	Occasionally	High Class
Following	No Exercise	Non Alcoholic	Regular	High Class
Following	Regular Exercise	Non Alcoholic	Regular	Poor
Following	Regular Exercise	Non Alcoholic	Regular	Poor
Occasionally	Regular Exercise	Non Alcoholic	Regular	High Class
Following	No Exercise	Non Alcoholic	Occasionally	Middle Class
Not Following	Regular Exercise	Non Alcoholic	Regular	High Class
Following	Regular Exercise	Non Alcoholic	Regular	Poor

Not Following	Regular Exercise	Non Alcoholic	Occasionally	High Class
Not Following	No Exercise	Non Alcoholic	Regular	High Class
Following	Regular Exercise	Non Alcoholic	Regular	High Class
Following	No Exercise	Non Alcoholic	Regular	High Class
Not Following	No Exercise	Non Alcoholic	Occasionally	High Class
Not Following	No Exercise	Non Alcoholic	Regular	High Class
Not Following	No Exercise	Non Alcoholic	Regular	High Class
Following	Regular Exercise	Non Alcoholic	Non Smoker	Poor
Not Following	Regular Exercise	Non Alcoholic	Regular	Poor
Following	Regular Exercise	Non Alcoholic	Occasionally	Poor
Following	No Exercise	Non Alcoholic	Regular	Poor
Not Following	No Exercise	Non Alcoholic	Regular	High Class
Following	Regular Exercise	Non Alcoholic	Occasionally	Poor
Not Following	No Exercise	Alcoholic	Regular	High Class
Not Following	Regular Exercise	Non Alcoholic	Occasionally	High Class
Following	Regular Exercise	Non Alcoholic	Regular	Poor
Following	Occasionally	Non Alcoholic	Regular	Poor
Following	Regular Exercise	Non Alcoholic	Occasionally	Poor
Following	Occasionally	Non Alcoholic	Regular	Middle Class
Occasionally	No Exercise	Non Alcoholic	Regular	Poor
Following	No Exercise	Non Alcoholic	Regular	High Class
Following	No Exercise	Non Alcoholic	Occasionally	High Class
Occasionally	No Exercise	Occasionally	Regular	High Class
Following	Regular Exercise	Non Alcoholic	Regular	Middle Class
Occasionally	No Exercise	Non Alcoholic	Regular	Middle Class
Following	No Exercise	Non Alcoholic	Regular	Poor
Following	No Exercise	Non Alcoholic	Occasionally	Poor
Not Following	Regular Exercise	Occasionally	Regular	Middle Class
Not Following	Regular Exercise	Occasionally	Regular	Middle Class
Following	Regular Exercise	Non Alcoholic	Occasionally	Poor
Not Following	Regular Exercise	Non Alcoholic	Regular	High Class
Not Following	Regular Exercise	Non Alcoholic	Occasionally	High Class
Following	Regular Exercise	Non Alcoholic	Non Smoker	Poor
Not Following	Regular Exercise	Non Alcoholic	Regular	High Class
Not Following	Regular Exercise	Non Alcoholic	Occasionally	High Class
Not Following	Occasionally	Non Alcoholic	Regular	High Class
Not Following	No Exercise	Non Alcoholic	Regular	Middle Class
Following	Occasionally	Alcoholic	Regular	Poor
Occasionally	No Exercise	Non Alcoholic	Occasionally	Middle Class
Occasionally	No Exercise	Non Alcoholic	Regular	High Class
Occasionally	Occasionally	Non Alcoholic	Regular	High Class
Not Following	Regular Exercise	Non Alcoholic	Regular	Middle Class
Not Following	Regular Exercise	Non Alcoholic	Regular	High Class
Not Following	No Exercise	Non Alcoholic	Occasionally	High Class
Occasionally	Regular Exercise	Non Alcoholic	Regular	High Class
Not Following	Regular Exercise	Non Alcoholic	Regular	Middle Class
Not Following	Regular Exercise	Non Alcoholic	Occasionally	High Class

Occasionally	Regular Exercise	Non Alcoholic	Regular	High Class
Not Following	Regular Exercise	Non Alcoholic	Occasionally	Middle Class
Occasionally	Regular Exercise	Non Alcoholic	Regular	High Class
Not Following	No Exercise	Non Alcoholic	Regular	High Class
Following	No Exercise	Occasionally	Occasionally	Poor
Following	Regular Exercise	Non Alcoholic	Regular	Middle Class
Following	No Exercise	Non Alcoholic	Regular	Middle Class
Following	Regular Exercise	Non Alcoholic	Non Smoker	Poor
Not Following	No Exercise	Non Alcoholic	Occasionally	Poor
Following	No Exercise	Non Alcoholic	Regular	Middle Class
Following	No Exercise	Non Alcoholic	Regular	Poor
Not Following	No Exercise	Non Alcoholic	Regular	Middle Class
Not Following	Regular Exercise	Non Alcoholic	Regular	High Class
Not Following	Regular Exercise	Non Alcoholic	Occasionally	High Class
Following	No Exercise	Non Alcoholic	Regular	High Class
Not Following	Regular Exercise	Non Alcoholic	Regular	High Class
Occasionally	No Exercise	Non Alcoholic	Occasionally	High Class
Not Following	Occasionally	Alcoholic	Regular	High Class
Not Following	No Exercise	Non Alcoholic	Regular	Middle Class
Following	Regular Exercise	Non Alcoholic	Occasionally	Middle Class
Not Following	No Exercise	Non Alcoholic	Regular	Poor
Following	Regular Exercise	Non Alcoholic	Regular	Middle Class
Following	Regular Exercise	Non Alcoholic	Occasionally	Poor
Not Following	No Exercise	Non Alcoholic	Regular	Middle Class
Not Following	Regular Exercise	Non Alcoholic	Occasionally	Poor
Not Following	No Exercise	Non Alcoholic	Regular	Middle Class
Not Following	Regular Exercise	Non Alcoholic	Regular	Poor
Not Following	No Exercise	Non Alcoholic	Occasionally	High Class
Following	Regular Exercise	Non Alcoholic	Regular	Poor
Not Following	No Exercise	Non Alcoholic	Non Smoker	High Class
Following	Regular Exercise	Non Alcoholic	Regular	Poor
Following	Occasionally	Non Alcoholic	Occasionally	High Class
Not Following	No Exercise	Alcoholic	Regular	Poor
Not Following	Regular Exercise	Non Alcoholic	Regular	High Class
Following	Regular Exercise	Non Alcoholic	Regular	Poor
Not Following	No Exercise	Non Alcoholic	Regular	Middle Class
Not Following	Regular Exercise	Non Alcoholic	Occasionally	High Class
Not Following	No Exercise	Alcoholic	Regular	Middle Class
Following	Regular Exercise	Non Alcoholic	Regular	High Class
Following	Regular Exercise	Non Alcoholic	Occasionally	Middle Class
Not Following	No Exercise	Non Alcoholic	Regular	High Class
Following	No Exercise	Non Alcoholic	Occasionally	Middle Class
Following	Regular Exercise	Non Alcoholic	Regular	High Class
Following	Occasionally	Non Alcoholic	Regular	Poor
Following	Regular Exercise	Non Alcoholic	Occasionally	High Class
Not Following	Occasionally	Non Alcoholic	Regular	Poor
Following	Regular Exercise	Alcoholic	Regular	High Class

Not Following	Regular Exercise	Non Alcoholic	Regular	Poor
Not Following	No Exercise	Non Alcoholic	Occasionally	Middle Class
Not Following	Regular Exercise	Non Alcoholic	Regular	Poor
Following	No Exercise	Non Alcoholic	Regular	Poor
Following	Regular Exercise	Non Alcoholic	Regular	Poor
Following	No Exercise	Non Alcoholic	Regular	Poor
Not Following	Regular Exercise	Non Alcoholic	Occasionally	High Class
Not Following	No Exercise	Non Alcoholic	Non Smoker	Poor
Not Following	Regular Exercise	Non Alcoholic	Regular	High Class
Following	Regular Exercise	Non Alcoholic	Occasionally	Poor
Following	Occasionally	Non Alcoholic	Regular	Middle Class
Following	Regular Exercise	Non Alcoholic	Occasionally	Poor
Following	Regular Exercise	Non Alcoholic	Regular	Poor
Not Following	No Exercise	Non Alcoholic	Regular	High Class
Following	Regular Exercise	Non Alcoholic	Non Smoker	Poor
Following	No Exercise	Non Alcoholic	Regular	Poor
Following	Regular Exercise	Non Alcoholic	Regular	Poor
Following	No Exercise	Non Alcoholic	Regular	Poor
Occasionally	Regular Exercise	Alcoholic	Occasionally	High Class
Occasionally	No Exercise	Non Alcoholic	Regular	Middle Class
Occasionally	Regular Exercise	Non Alcoholic	Regular	High Class
Following	Occasionally	Non Alcoholic	Regular	Poor
Following	Occasionally	Non Alcoholic	Regular	Poor
Following	No Exercise	Non Alcoholic	Occasionally	Poor
Following	Regular Exercise	Non Alcoholic	Regular	Poor
Following	No Exercise	Non Alcoholic	Regular	Poor
Occasionally	Regular Exercise	Non Alcoholic	Occasionally	High Class
Not Following	No Exercise	Non Alcoholic	Regular	High Class
Occasionally	Regular Exercise	Non Alcoholic	Occasionally	High Class
Not Following	Regular Exercise	Non Alcoholic	Regular	High Class
Not Following	No Exercise	Non Alcoholic	Regular	High Class
Occasionally	No Exercise	Occasionally	Occasionally	Poor
Not Following	Regular Exercise	Non Alcoholic	Non Smoker	Poor
Occasionally	No Exercise	Non Alcoholic	Regular	Poor
Occasionally	Occasionally	Non Alcoholic	Regular	Poor
Occasionally	No Exercise	Non Alcoholic	Occasionally	Poor
Not Following	Regular Exercise	Non Alcoholic	Regular	Middle Class
Not Following	No Exercise	Non Alcoholic	Regular	High Class
Occasionally	Regular Exercise	Non Alcoholic	Regular	High Class
Not Following	No Exercise	Non Alcoholic	Regular	High Class
Not Following	Regular Exercise	Non Alcoholic	Occasionally	High Class
Not Following	No Exercise	Non Alcoholic	Regular	High Class
Not Following	No Exercise	Occasionally	Regular	Middle Class
Following	Regular Exercise	Non Alcoholic	Occasionally	Middle Class
Following	No Exercise	Non Alcoholic	Regular	Middle Class
Following	Occasionally	Non Alcoholic	Occasionally	Poor
Not Following	Regular Exercise	Non Alcoholic	Regular	Poor

Not Following	Regular Exercise	Non Alcoholic	Regular	Poor
Not Following	No Exercise	Non Alcoholic	Occasionally	Middle Class
Occasionally	No Exercise	Non Alcoholic	Regular	Poor
Not Following	No Exercise	Non Alcoholic	Regular	Middle Class
Occasionally	No Exercise	Non Alcoholic	Regular	Middle Class
Occasionally	Regular Exercise	Non Alcoholic	Occasionally	Poor
Occasionally	Occasionally	Non Alcoholic	Regular	Poor
Not Following	Regular Exercise	Non Alcoholic	Regular	Poor
Following	Regular Exercise	Non Alcoholic	Regular	Middle Class
Not Following	No Exercise	Non Alcoholic	Regular	Middle Class
Not Following	No Exercise	Alcoholic	Non Smoker	Middle Class
Following	No Exercise	Non Alcoholic	Regular	Poor
Not Following	Regular Exercise	Non Alcoholic	Regular	High Class
Not Following	No Exercise	Non Alcoholic	Occasionally	High Class
Not Following	Regular Exercise	Non Alcoholic	Regular	High Class
Following	No Exercise	Non Alcoholic	Occasionally	Poor
Following	Regular Exercise	Non Alcoholic	Regular	Middle Class
Following	No Exercise	Non Alcoholic	Regular	Poor
Following	Regular Exercise	Non Alcoholic	Occasionally	Middle Class
Following	No Exercise	Non Alcoholic	Regular	Poor
Occasionally	No Exercise	Non Alcoholic	Regular	High Class
Occasionally	Regular Exercise	Non Alcoholic	Regular	High Class
Following	Regular Exercise	Non Alcoholic	Regular	Poor
Occasionally	Regular Exercise	Non Alcoholic	Regular	High Class
Following	Regular Exercise	Non Alcoholic	Regular	Middle Class
Occasionally	Regular Exercise	Occasionally	Regular	Poor
Following	No Exercise	Non Alcoholic	Regular	High Class
Not Following	Regular Exercise	Occasionally	Occasionally	High Class
Following	Regular Exercise	Non Alcoholic	Regular	High Class
Not Following	Regular Exercise	Non Alcoholic	Regular	High Class
Not Following	No Exercise	Non Alcoholic	Occasionally	High Class
Not Following	Regular Exercise	Non Alcoholic	Regular	High Class
Following	Regular Exercise	Alcoholic	Occasionally	High Class
Not Following	Regular Exercise	Non Alcoholic	Regular	Middle Class
Not Following	Regular Exercise	Non Alcoholic	Regular	High Class
Following	No Exercise	Non Alcoholic	Non Smoker	High Class
Following	No Exercise	Non Alcoholic	Regular	Poor
Not Following	No Exercise	Non Alcoholic	Regular	High Class
Following	Regular Exercise	Non Alcoholic	Regular	High Class
Following	No Exercise	Non Alcoholic	Occasionally	Middle Class
Not Following	Regular Exercise	Non Alcoholic	Regular	High Class
Not Following	Occasionally	Non Alcoholic	Regular	Middle Class
Occasionally	Regular Exercise	Occasionally	Regular	High Class
Following	No Exercise	Non Alcoholic	Regular	High Class
Following	No Exercise	Non Alcoholic	Regular	Poor
Not Following	Regular Exercise	Non Alcoholic	Regular	High Class
Not Following	No Exercise	Non Alcoholic	Regular	High Class

Following	No Exercise	Non Alcoholic	Regular	Poor
Not Following	Regular Exercise	Non Alcoholic	Occasionally	High Class
Not Following	Regular Exercise	Non Alcoholic	Regular	High Class
Following	No Exercise	Non Alcoholic	Regular	Poor
Not Following	No Exercise	Non Alcoholic	Occasionally	Middle Class
Following	No Exercise	Non Alcoholic	Regular	Poor
Not Following	No Exercise	Non Alcoholic	Occasionally	High Class
Following	Regular Exercise	Non Alcoholic	Regular	Middle Class
Occasionally	No Exercise	Non Alcoholic	Regular	High Class
Following	Regular Exercise	Non Alcoholic	Occasionally	Poor
Occasionally	Regular Exercise	Non Alcoholic	Regular	Poor
Following	No Exercise	Non Alcoholic	Regular	Poor
Not Following	No Exercise	Non Alcoholic	Regular	Poor
Following	No Exercise	Non Alcoholic	Occasionally	Poor
Following	Regular Exercise	Non Alcoholic	Regular	High Class
Following	No Exercise	Non Alcoholic	Regular	High Class
Not Following	Regular Exercise	Non Alcoholic	Regular	High Class
Not Following	No Exercise	Non Alcoholic	Regular	High Class
Not Following	Regular Exercise	Non Alcoholic	Regular	High Class
Not Following	No Exercise	Non Alcoholic	Regular	Poor
Following	Regular Exercise	Non Alcoholic	Regular	Poor
Not Following	No Exercise	Non Alcoholic	Regular	Poor
Following	No Exercise	Non Alcoholic	Occasionally	Poor
Not Following	Regular Exercise	Non Alcoholic	Regular	Poor
Not Following	Regular Exercise	Non Alcoholic	Regular	Middle Class
Following	Regular Exercise	Non Alcoholic	Occasionally	High Class
Not Following	Regular Exercise	Non Alcoholic	Regular	High Class
Not Following	Regular Exercise	Occasionally	Occasionally	High Class
Following	No Exercise	Non Alcoholic	Regular	High Class
Following	Regular Exercise	Non Alcoholic	Regular	High Class
Following	Regular Exercise	Non Alcoholic	Occasionally	Middle Class
Following	Regular Exercise	Non Alcoholic	Regular	Middle Class
Following	No Exercise	Non Alcoholic	Regular	Middle Class
Following	Regular Exercise	Non Alcoholic	Regular	Poor
Not Following	Regular Exercise	Non Alcoholic	Occasionally	Poor
Not Following	Regular Exercise	Non Alcoholic	Regular	Poor
Not Following	Regular Exercise	Non Alcoholic	Regular	Middle Class
Following	No Exercise	Non Alcoholic	Regular	Poor
Not Following	No Exercise	Non Alcoholic	Regular	Middle Class
Not Following	No Exercise	Non Alcoholic	Regular	Middle Class
Following	Regular Exercise	Non Alcoholic	Regular	Poor
Not Following	No Exercise	Occasionally	Regular	Poor
Not Following	Regular Exercise	Non Alcoholic	Regular	Poor
Following	No Exercise	Occasionally	Occasionally	Middle Class
Not Following	Regular Exercise	Occasionally	Regular	Middle Class
Not Following	No Exercise	Non Alcoholic	Non Smoker	Middle Class
Following	Regular Exercise	Non Alcoholic	Occasionally	Poor

Following	No Exercise	Non Alcoholic	Regular	High Class
Following	Regular Exercise	Non Alcoholic	Occasionally	High Class
Not Following	No Exercise	Non Alcoholic	Regular	High Class
Not Following	Regular Exercise	Non Alcoholic	Regular	Poor
Not Following	Regular Exercise	Non Alcoholic	Occasionally	Middle Class
Following	Occasionally	Alcoholic	Regular	Poor
Not Following	No Exercise	Non Alcoholic	Regular	Middle Class
Following	Regular Exercise	Non Alcoholic	Regular	Poor
Following	No Exercise	Non Alcoholic	Occasionally	High Class
Following	Regular Exercise	Non Alcoholic	Regular	High Class
Not Following	No Exercise	Non Alcoholic	Regular	Poor
Not Following	Regular Exercise	Non Alcoholic	Regular	High Class
Not Following	No Exercise	Non Alcoholic	Regular	Middle Class
Following	Regular Exercise	Non Alcoholic	Regular	Poor
Not Following	No Exercise	Non Alcoholic	Regular	High Class
Following	Regular Exercise	Alcoholic	Regular	High Class
Following	No Exercise	Non Alcoholic	Regular	High Class
Following	Regular Exercise	Non Alcoholic	Occasionally	Poor
Following	Regular Exercise	Non Alcoholic	Regular	Middle Class
Occasionally	Regular Exercise	Non Alcoholic	Regular	High Class
Not Following	No Exercise	Alcoholic	Occasionally	High Class
Not Following	No Exercise	Alcoholic	Regular	High Class
Occasionally	Regular Exercise	Non Alcoholic	Occasionally	High Class
Not Following	Regular Exercise	Non Alcoholic	Regular	Middle Class
Occasionally	Regular Exercise	Non Alcoholic	Regular	Middle Class
Occasionally	Regular Exercise	Non Alcoholic	Occasionally	Middle Class
Occasionally	Regular Exercise	Non Alcoholic	Regular	Middle Class
Occasionally	No Exercise	Non Alcoholic	Regular	Middle Class
Occasionally	Regular Exercise	Non Alcoholic	Regular	Middle Class
Occasionally	Regular Exercise	Non Alcoholic	Occasionally	Poor
Not Following	Regular Exercise	Alcoholic	Regular	Poor
Occasionally	Regular Exercise	Non Alcoholic	Regular	Poor
Occasionally	Regular Exercise	Non Alcoholic	Regular	Poor
Occasionally	Regular Exercise	Non Alcoholic	Regular	Poor
Following	Regular Exercise	Non Alcoholic	Regular	Poor
Following	Regular Exercise	Non Alcoholic	Regular	High Class
Not Following	No Exercise	Non Alcoholic	Regular	Poor
Not Following	No Exercise	Non Alcoholic	Regular	Poor
Following	No Exercise	Occasionally	Occasionally	Poor
Following	Regular Exercise	Non Alcoholic	Regular	Poor
Following	No Exercise	Non Alcoholic	Regular	Poor
Following	Regular Exercise	Non Alcoholic	Occasionally	Poor
Following	No Exercise	Non Alcoholic	Regular	Poor
Following	Regular Exercise	Non Alcoholic	Occasionally	Poor
Following	Occasionally	Non Alcoholic	Regular	Poor
Not Following	No Exercise	Occasionally	Regular	High Class
Following	Regular Exercise	Non Alcoholic	Occasionally	High Class

Following	No Exercise	Non Alcoholic	Regular	High Class
Not Following	No Exercise	Non Alcoholic	Regular	High Class
Not Following	Regular Exercise	Non Alcoholic	Regular	Middle Class
Following	Regular Exercise	Non Alcoholic	Occasionally	High Class
Not Following	No Exercise	Non Alcoholic	Regular	Middle Class
Not Following	No Exercise	Non Alcoholic	Regular	Middle Class
Following	Occasionally	Occasionally	Regular	High Class
Not Following	Regular Exercise	Alcoholic	Non Smoker	Middle Class
Following	Regular Exercise	Non Alcoholic	Regular	High Class
Following	Regular Exercise	Non Alcoholic	Regular	Middle Class
Not Following	No Exercise	Non Alcoholic	Occasionally	High Class
Not Following	Regular Exercise	Non Alcoholic	Regular	High Class
Following	Regular Exercise	Non Alcoholic	Regular	Middle Class
Following	Regular Exercise	Non Alcoholic	Occasionally	High Class
Not Following	Regular Exercise	Non Alcoholic	Regular	Middle Class
Following	No Exercise	Non Alcoholic	Occasionally	High Class
Following	No Exercise	Occasionally	Regular	Middle Class
Not Following	No Exercise	Non Alcoholic	Regular	High Class
Following	Regular Exercise	Non Alcoholic	Occasionally	Middle Class
Following	No Exercise	Non Alcoholic	Regular	High Class
Following	No Exercise	Non Alcoholic	Regular	High Class
Not Following	No Exercise	Non Alcoholic	Regular	Poor
Not Following	Regular Exercise	Non Alcoholic	Occasionally	Middle Class
Not Following	No Exercise	Occasionally	Regular	Middle Class
Not Following	Regular Exercise	Non Alcoholic	Regular	Poor
Not Following	No Exercise	Non Alcoholic	Regular	High Class
Not Following	Regular Exercise	Non Alcoholic	Regular	High Class
Following	Regular Exercise	Non Alcoholic	Regular	Poor
Not Following	Regular Exercise	Non Alcoholic	Regular	High Class
Following	Regular Exercise	Non Alcoholic	Regular	Poor
Following	No Exercise	Non Alcoholic	Regular	Poor
Not Following	Regular Exercise	Non Alcoholic	Occasionally	High Class
Not Following	No Exercise	Non Alcoholic	Regular	High Class
Following	No Exercise	Non Alcoholic	Regular	Poor
Not Following	Regular Exercise	Occasionally	Occasionally	High Class
Following	No Exercise	Non Alcoholic	Regular	Poor
Not Following	Regular Exercise	Non Alcoholic	Occasionally	High Class
Occasionally	No Exercise	Non Alcoholic	Regular	High Class
Not Following	No Exercise	Non Alcoholic	Regular	High Class
Not Following	No Exercise	Non Alcoholic	Occasionally	High Class
Occasionally	No Exercise	Non Alcoholic	Regular	Middle Class
Not Following	No Exercise	Non Alcoholic	Regular	Middle Class
Occasionally	Regular Exercise	Non Alcoholic	Regular	Middle Class
Not Following	Regular Exercise	Non Alcoholic	Occasionally	Middle Class
Not Following	No Exercise	Non Alcoholic	Regular	Middle Class
Not Following	Regular Exercise	Alcoholic	Regular	Middle Class
Not Following	Regular Exercise	Non Alcoholic	Regular	Middle Class

Occasionally	No Exercise	Non Alcoholic	Regular	Poor
Occasionally	Regular Exercise	Non Alcoholic	Regular	Middle Class
Occasionally	No Exercise	Non Alcoholic	Regular	Poor
Occasionally	No Exercise	Non Alcoholic	Regular	High Class
Occasionally	No Exercise	Alcoholic	Regular	Poor
Occasionally	Regular Exercise	Non Alcoholic	Occasionally	High Class
Occasionally	Regular Exercise	Non Alcoholic	Regular	Poor
Not Following	No Exercise	Non Alcoholic	Regular	High Class
Not Following	Regular Exercise	Alcoholic	Occasionally	High Class
Not Following	No Exercise	Non Alcoholic	Regular	Poor
Occasionally	Regular Exercise	Non Alcoholic	Occasionally	Poor
Not Following	No Exercise	Non Alcoholic	Regular	High Class
Occasionally	Regular Exercise	Non Alcoholic	Regular	Poor
Not Following	Regular Exercise	Non Alcoholic	Occasionally	High Class
Not Following	Regular Exercise	Non Alcoholic	Regular	High Class
Not Following	Regular Exercise	Non Alcoholic	Regular	Poor
Not Following	No Exercise	Non Alcoholic	Regular	Poor
Not Following	Occasionally	Non Alcoholic	Occasionally	Poor
Occasionally	No Exercise	Non Alcoholic	Regular	Poor
Following	No Exercise	Non Alcoholic	Regular	Poor
Not Following	No Exercise	Non Alcoholic	Regular	Poor
Occasionally	No Exercise	Alcoholic	Regular	Middle Class
Not Following	No Exercise	Non Alcoholic	Regular	Middle Class
Occasionally	Regular Exercise	Non Alcoholic	Regular	Middle Class
Not Following	No Exercise	Non Alcoholic	Regular	Middle Class
Not Following	Regular Exercise	Non Alcoholic	Regular	Poor
Following	Regular Exercise	Non Alcoholic	Occasionally	Middle Class
Following	No Exercise	Non Alcoholic	Regular	Poor
Following	Regular Exercise	Non Alcoholic	Regular	Poor
Not Following	No Exercise	Non Alcoholic	Occasionally	Poor
Following	Regular Exercise	Non Alcoholic	Regular	Middle Class
Following	No Exercise	Non Alcoholic	Occasionally	Middle Class
Not Following	Regular Exercise	Non Alcoholic	Regular	Poor
Not Following	Regular Exercise	Non Alcoholic	Regular	Poor
Not Following	No Exercise	Non Alcoholic	Occasionally	Middle Class
Not Following	No Exercise	Non Alcoholic	Regular	Poor
Following	Regular Exercise	Occasionally	Regular	Poor
Following	No Exercise	Non Alcoholic	Regular	High Class
Not Following	Regular Exercise	Non Alcoholic	Occasionally	Poor
Not Following	No Exercise	Non Alcoholic	Regular	High Class
Not Following	Regular Exercise	Non Alcoholic	Regular	High Class
Not Following	No Exercise	Non Alcoholic	Regular	High Class
Following	Regular Exercise	Non Alcoholic	Regular	Middle Class
Not Following	No Exercise	Non Alcoholic	Regular	Poor
Following	Regular Exercise	Non Alcoholic	Regular	High Class
Not Following	No Exercise	Non Alcoholic	Regular	High Class
Not Following	No Exercise	Non Alcoholic	Regular	High Class

Following	Regular Exercise	Occasionally	Occasionally	Poor
Following	Regular Exercise	Non Alcoholic	Regular	Middle Class
Not Following	Regular Exercise	Non Alcoholic	Regular	Poor
Following	Regular Exercise	Non Alcoholic	Occasionally	Middle Class
Not Following	Regular Exercise	Non Alcoholic	Regular	Poor
Following	Regular Exercise	Non Alcoholic	Occasionally	Middle Class
Following	No Exercise	Non Alcoholic	Regular	Poor
Following	No Exercise	Non Alcoholic	Regular	Middle Class
Not Following	Regular Exercise	Non Alcoholic	Occasionally	Poor
Following	Regular Exercise	Non Alcoholic	Regular	Middle Class
Following	Regular Exercise	Non Alcoholic	Regular	Poor
Not Following	Regular Exercise	Non Alcoholic	Regular	High Class
Not Following	Regular Exercise	Occasionally	Occasionally	High Class
Following	Regular Exercise	Non Alcoholic	Non Smoker	High Class
Not Following	Regular Exercise	Non Alcoholic	Regular	Middle Class
Following	No Exercise	Non Alcoholic	Regular	High Class
Not Following	No Exercise	Non Alcoholic	Regular	Middle Class
Not Following	Occasionally	Occasionally	Regular	High Class
Following	No Exercise	Non Alcoholic	Regular	Poor
Following	Occasionally	Non Alcoholic	Regular	High Class
Following	No Exercise	Non Alcoholic	Regular	High Class
Following	Regular Exercise	Non Alcoholic	Occasionally	Poor
Not Following	No Exercise	Non Alcoholic	Regular	High Class
Not Following	No Exercise	Non Alcoholic	Regular	High Class
Following	Occasionally	Non Alcoholic	Occasionally	High Class
Not Following	No Exercise	Non Alcoholic	Regular	High Class
Following	No Exercise	Occasionally	Occasionally	Poor
Following	Occasionally	Non Alcoholic	Regular	Poor
Following	No Exercise	Non Alcoholic	Regular	Poor
Occasionally	No Exercise	Non Alcoholic	Occasionally	Poor
Following	Occasionally	Non Alcoholic	Regular	High Class
Not Following	No Exercise	Non Alcoholic	Regular	High Class
Not Following	No Exercise	Occasionally	Regular	Poor
Following	Regular Exercise	Non Alcoholic	Occasionally	High Class
Not Following	Regular Exercise	Non Alcoholic	Regular	Poor
Following	Regular Exercise	Non Alcoholic	Regular	High Class
Following	Regular Exercise	Non Alcoholic	Regular	Poor
Following	Regular Exercise	Non Alcoholic	Regular	Poor
Following	No Exercise	Non Alcoholic	Regular	Poor
Not Following	No Exercise	Non Alcoholic	Regular	Middle Class
Not Following	No Exercise	Non Alcoholic	Regular	High Class
Following	No Exercise	Occasionally	Regular	Poor
Not Following	Regular Exercise	Non Alcoholic	Occasionally	High Class
Not Following	Regular Exercise	Non Alcoholic	Regular	High Class
Not Following	Regular Exercise	Non Alcoholic	Regular	High Class
Following	Regular Exercise	Alcoholic	Occasionally	Poor
Not Following	No Exercise	Non Alcoholic	Regular	High Class

Not Following	No Exercise	Non Alcoholic	Occasionally	High Class
Following	Regular Exercise	Non Alcoholic	Regular	Poor
Not Following	Regular Exercise	Non Alcoholic	Regular	High Class
Not Following	No Exercise	Non Alcoholic	Occasionally	High Class
Occasionally	Regular Exercise	Alcoholic	Regular	Poor
Occasionally	No Exercise	Non Alcoholic	Regular	Poor
Occasionally	Regular Exercise	Non Alcoholic	Regular	Poor
Not Following	No Exercise	Non Alcoholic	Occasionally	Poor
Not Following	Regular Exercise	Non Alcoholic	Regular	Poor
Occasionally	No Exercise	Non Alcoholic	Regular	Poor
Not Following	Regular Exercise	Occasionally	Regular	Poor
Not Following	No Exercise	Non Alcoholic	Regular	Poor
Not Following	Regular Exercise	Non Alcoholic	Regular	Poor
Occasionally	Regular Exercise	Non Alcoholic	Regular	High Class
Occasionally	Regular Exercise	Non Alcoholic	Regular	High Class
Not Following	Regular Exercise	Non Alcoholic	Regular	Poor
Occasionally	No Exercise	Non Alcoholic	Occasionally	High Class
Occasionally	No Exercise	Occasionally	Regular	High Class
Not Following	Regular Exercise	Non Alcoholic	Regular	Poor
Occasionally	No Exercise	Non Alcoholic	Occasionally	High Class
Not Following	Regular Exercise	Non Alcoholic	Regular	Poor
Occasionally	Regular Exercise	Non Alcoholic	Occasionally	High Class
Not Following	Regular Exercise	Non Alcoholic	Regular	Poor
Occasionally	Regular Exercise	Non Alcoholic	Regular	Poor
Occasionally	Regular Exercise	Non Alcoholic	Occasionally	Poor
Following	No Exercise	Non Alcoholic	Regular	Poor
Not Following	Regular Exercise	Non Alcoholic	Regular	Middle Class
Not Following	No Exercise	Occasionally	Regular	Middle Class
Following	No Exercise	Non Alcoholic	Occasionally	Middle Class
Not Following	No Exercise	Non Alcoholic	Regular	Poor
Following	Regular Exercise	Non Alcoholic	Regular	Middle Class
Not Following	Regular Exercise	Non Alcoholic	Regular	Poor
Not Following	No Exercise	Non Alcoholic	Regular	Poor
Not Following	Regular Exercise	Non Alcoholic	Regular	High Class
Following	No Exercise	Non Alcoholic	Regular	Poor
Following	Regular Exercise	Non Alcoholic	Regular	High Class
Not Following	No Exercise	Non Alcoholic	Regular	Poor
Not Following	Regular Exercise	Non Alcoholic	Occasionally	High Class
Following	Regular Exercise	Non Alcoholic	Regular	Poor
Not Following	Regular Exercise	Non Alcoholic	Regular	High Class
Not Following	No Exercise	Non Alcoholic	Occasionally	Poor
Following	Regular Exercise	Non Alcoholic	Regular	High Class
Not Following	No Exercise	Occasionally	Occasionally	High Class
Not Following	Regular Exercise	Non Alcoholic	Regular	Middle Class
Not Following	Regular Exercise	Non Alcoholic	Regular	High Class
Following	Regular Exercise	Non Alcoholic	Occasionally	Middle Class
Not Following	Regular Exercise	Non Alcoholic	Regular	High Class

Not Following	Regular Exercise	Non Alcoholic	Regular	Middle Class
Following	No Exercise	Non Alcoholic	Regular	Poor
Following	Regular Exercise	Non Alcoholic	Occasionally	High Class
Not Following	No Exercise	Non Alcoholic	Regular	Middle Class
Not Following	Regular Exercise	Non Alcoholic	Regular	High Class
Occasionally	Regular Exercise	Non Alcoholic	Regular	Middle Class
Not Following	No Exercise	Non Alcoholic	Regular	Poor
Following	Regular Exercise	Non Alcoholic	Regular	High Class
Not Following	No Exercise	Occasionally	Regular	Middle Class
Following	Regular Exercise	Non Alcoholic	Regular	High Class
Not Following	No Exercise	Non Alcoholic	Regular	High Class
Following	Regular Exercise	Non Alcoholic	Occasionally	High Class
Not Following	No Exercise	Non Alcoholic	Regular	High Class
Following	Regular Exercise	Non Alcoholic	Regular	Poor
Following	No Exercise	Non Alcoholic	Occasionally	Poor
Not Following	Regular Exercise	Non Alcoholic	Regular	High Class
Following	Regular Exercise	Non Alcoholic	Occasionally	Poor
Not Following	Regular Exercise	Non Alcoholic	Regular	High Class
Following	No Exercise	Non Alcoholic	Regular	Poor
Not Following	No Exercise	Non Alcoholic	Occasionally	High Class
Not Following	Regular Exercise	Non Alcoholic	Regular	High Class
Following	Regular Exercise	Alcoholic	Regular	Poor
Following	Regular Exercise	Non Alcoholic	Regular	High Class
Following	Regular Exercise	Non Alcoholic	Non Smoker	High Class
Following	Regular Exercise	Non Alcoholic	Regular	High Class
Not Following	No Exercise	Non Alcoholic	Regular	Middle Class
Not Following	Regular Exercise	Non Alcoholic	Regular	Middle Class
Following	No Exercise	Non Alcoholic	Regular	Poor
Not Following	Regular Exercise	Alcoholic	Regular	Middle Class
Not Following	Regular Exercise	Non Alcoholic	Regular	Poor
Following	Regular Exercise	Non Alcoholic	Regular	Poor
Following	Regular Exercise	Non Alcoholic	Regular	Poor
Following	No Exercise	Non Alcoholic	Occasionally	Middle Class
Occasionally	No Exercise	Non Alcoholic	Regular	Middle Class
Following	Regular Exercise	Non Alcoholic	Regular	Poor
Following	Regular Exercise	Alcoholic	Occasionally	Poor
Occasionally	No Exercise	Non Alcoholic	Regular	Middle Class
Following	Regular Exercise	Non Alcoholic	Occasionally	Poor
Following	No Exercise	Non Alcoholic	Regular	Poor
Occasionally	Regular Exercise	Non Alcoholic	Regular	Middle Class
Not Following	Regular Exercise	Non Alcoholic	Occasionally	Middle Class
Following	No Exercise	Non Alcoholic	Regular	Poor
Following	Regular Exercise	Non Alcoholic	Regular	Poor
Not Following	No Exercise	Alcoholic	Regular	High Class
Not Following	Regular Exercise	Non Alcoholic	Occasionally	High Class
Not Following	Regular Exercise	Non Alcoholic	Regular	High Class
Following	No Exercise	Non Alcoholic	Regular	Poor

Following	Occasionally	Non Alcoholic	Regular	Poor
Following	No Exercise	Non Alcoholic	Regular	Poor
Not Following	Regular Exercise	Non Alcoholic	Regular	High Class
Following	Regular Exercise	Non Alcoholic	Regular	Middle Class
Occasionally	Regular Exercise	Non Alcoholic	Regular	Poor
Not Following	Regular Exercise	Non Alcoholic	Regular	Middle Class
Not Following	No Exercise	Non Alcoholic	Occasionally	Middle Class
Following	No Exercise	Non Alcoholic	Regular	Poor
Following	Regular Exercise	Non Alcoholic	Regular	Poor
Following	No Exercise	Alcoholic	Occasionally	Poor
Following	Regular Exercise	Non Alcoholic	Regular	Poor
Following	No Exercise	Non Alcoholic	Occasionally	Poor
Not Following	Regular Exercise	Non Alcoholic	Regular	Middle Class
Following	Regular Exercise	Non Alcoholic	Regular	Middle Class
Not Following	Regular Exercise	Non Alcoholic	Occasionally	Poor
Following	No Exercise	Non Alcoholic	Regular	Poor
Not Following	Regular Exercise	Non Alcoholic	Regular	High Class
Not Following	No Exercise	Alcoholic	Regular	High Class
Following	Regular Exercise	Non Alcoholic	Occasionally	High Class
Following	Regular Exercise	Non Alcoholic	Regular	Middle Class
Not Following	Regular Exercise	Non Alcoholic	Regular	High Class
Following	Regular Exercise	Non Alcoholic	Regular	Poor
Not Following	Regular Exercise	Non Alcoholic	Regular	High Class
Occasionally	Regular Exercise	Non Alcoholic	Regular	Poor
Not Following	Regular Exercise	Non Alcoholic	Regular	High Class
Not Following	Regular Exercise	Non Alcoholic	Regular	High Class
Not Following	Regular Exercise	Non Alcoholic	Regular	Middle Class
Occasionally	Regular Exercise	Non Alcoholic	Occasionally	High Class
Not Following	No Exercise	Non Alcoholic	Regular	Poor
Occasionally	No Exercise	Non Alcoholic	Regular	High Class
Occasionally	Regular Exercise	Non Alcoholic	Occasionally	Poor
Following	Regular Exercise	Non Alcoholic	Regular	High Class
Not Following	Regular Exercise	Alcoholic	Occasionally	High Class
Not Following	No Exercise	Alcoholic	Regular	High Class
Occasionally	Regular Exercise	Non Alcoholic	Non Smoker	Poor
Following	Regular Exercise	Non Alcoholic	Non Smoker	Poor
Occasionally	Regular Exercise	Non Alcoholic	Non Smoker	Middle Class
Following	Occasionally	Alcoholic	Regular	Middle Class
Occasionally	Regular Exercise	Alcoholic	Regular	Middle Class
Following	Occasionally	Alcoholic	Regular	Poor
Occasionally	Occasionally	Alcoholic	Regular	Poor
Occasionally	Regular Exercise	Occasionally	Non Smoker	Poor
Not Following	Regular Exercise	Occasionally	Regular	Poor
Not Following	Occasionally	Occasionally	Regular	High Class
Following	No Exercise	Occasionally	Occasionally	High Class
Not Following	Regular Exercise	Occasionally	Occasionally	Poor
Following	Regular Exercise	Occasionally	Occasionally	Poor

Following	Occasionally	Non Alcoholic	Non Smoker	Poor
Occasionally	Occasionally	Non Alcoholic	Non Smoker	Poor
Occasionally	Occasionally	Non Alcoholic	Regular	High Class
Following	Regular Exercise	Alcoholic	Regular	High Class
Occasionally	Regular Exercise	Alcoholic	Regular	Poor
Occasionally	No Exercise	Alcoholic	Non Smoker	Middle Class
Following	No Exercise	Alcoholic	Non Smoker	Poor
Following	No Exercise	Non Alcoholic	Occasionally	Poor
Occasionally	No Exercise	Non Alcoholic	Occasionally	Middle Class
Not Following	No Exercise	Non Alcoholic	Regular	Middle Class
Occasionally	No Exercise	Non Alcoholic	Non Smoker	Poor
Occasionally	Regular Exercise	Non Alcoholic	Regular	Poor
Following	Regular Exercise	Non Alcoholic	Regular	Poor
Following	Occasionally	Alcoholic	Non Smoker	Middle Class
Not Following	Occasionally	Alcoholic	Non Smoker	High Class
Occasionally	Regular Exercise	Occasionally	Occasionally	Poor
Occasionally	No Exercise	Alcoholic	Occasionally	High Class
Not Following	No Exercise	Non Alcoholic	Non Smoker	Middle Class
Occasionally	Regular Exercise	Non Alcoholic	Non Smoker	Poor
Occasionally	Regular Exercise	Non Alcoholic	Regular	High Class
Occasionally	Regular Exercise	Non Alcoholic	Regular	High Class
Occasionally	Regular Exercise	Non Alcoholic	Regular	High Class
Not Following	No Exercise	Non Alcoholic	Regular	Poor
Occasionally	No Exercise	Occasionally	Regular	Poor
Following	Occasionally	Occasionally	Non Smoker	Poor
Following	Occasionally	Occasionally	Non Smoker	High Class
Not Following	No Exercise	Alcoholic	Non Smoker	Poor
Not Following	Occasionally	Alcoholic	Non Smoker	Poor
Occasionally	Regular Exercise	Alcoholic	Occasionally	Poor
Not Following	Regular Exercise	Occasionally	Occasionally	High Class
Occasionally	Occasionally	Occasionally	Occasionally	Poor
Not Following	Regular Exercise	Occasionally	Occasionally	High Class
Occasionally	Regular Exercise	Alcoholic	Non Smoker	Poor
Occasionally	Regular Exercise	Alcoholic	Non Smoker	High Class
Following	Occasionally	Non Alcoholic	Non Smoker	High Class
Following	No Exercise	Non Alcoholic	Regular	Middle Class
Not Following	Regular Exercise	Occasionally	Regular	High Class
Occasionally	Occasionally	Non Alcoholic	Occasionally	Middle Class
Occasionally	No Exercise	Occasionally	Occasionally	High Class
Not Following	No Exercise	Alcoholic	Regular	Middle Class
Following	Occasionally	Alcoholic	Regular	Poor
Following	Regular Exercise	Alcoholic	Occasionally	High Class
Following	Regular Exercise	Occasionally	Occasionally	Middle Class
Following	Regular Exercise	Alcoholic	Non Smoker	High Class
Not Following	Regular Exercise	Alcoholic	Non Smoker	Middle Class
Following	Regular Exercise	Alcoholic	Non Smoker	Poor
Not Following	No Exercise	Non Alcoholic	Non Smoker	High Class

Following	No Exercise	Non Alcoholic	Regular	Middle Class
Not Following	No Exercise	Non Alcoholic	Non Smoker	High Class
Not Following	Occasionally	Occasionally	Occasionally	High Class
Not Following	No Exercise	Non Alcoholic	Occasionally	High Class
Occasionally	Regular Exercise	Alcoholic	Occasionally	High Class
Occasionally	Occasionally	Non Alcoholic	Occasionally	Poor
Not Following	No Exercise	Occasionally	Occasionally	Poor
Following	Regular Exercise	Alcoholic	Occasionally	High Class
Following	Regular Exercise	Non Alcoholic	Non Smoker	Poor
Occasionally	No Exercise	Occasionally	Non Smoker	High Class
Occasionally	Occasionally	Alcoholic	Non Smoker	Poor
Occasionally	No Exercise	Occasionally	Regular	High Class
Following	No Exercise	Occasionally	Regular	High Class
Occasionally	Regular Exercise	Occasionally	Regular	Poor
Not Following	Regular Exercise	Non Alcoholic	Regular	High Class
Following	Occasionally	Non Alcoholic	Regular	High Class
Occasionally	Occasionally	Non Alcoholic	Non Smoker	High Class
Not Following	No Exercise	Non Alcoholic	Non Smoker	Middle Class
Occasionally	Occasionally	Non Alcoholic	Non Smoker	Middle Class
Occasionally	Regular Exercise	Non Alcoholic	Non Smoker	Poor
Occasionally	No Exercise	Alcoholic	Non Smoker	Middle Class
Not Following	Occasionally	Alcoholic	Non Smoker	Poor
Not Following	Regular Exercise	Alcoholic	Non Smoker	Poor
Following	No Exercise	Non Alcoholic	Occasionally	Poor
Following	Regular Exercise	Alcoholic	Occasionally	Middle Class
Not Following	Occasionally	Non Alcoholic	Occasionally	Middle Class
Occasionally	No Exercise	Occasionally	Occasionally	Poor
Not Following	Regular Exercise	Occasionally	Non Smoker	Poor
Following	Regular Exercise	Non Alcoholic	Non Smoker	Poor
Occasionally	Occasionally	Non Alcoholic	Non Smoker	Middle Class
Occasionally	Occasionally	Non Alcoholic	Non Smoker	Middle Class
Occasionally	Occasionally	Occasionally	Regular	Poor
Not Following	Regular Exercise	Alcoholic	Regular	Poor
Not Following	Regular Exercise	Alcoholic	Regular	High Class
Following	Regular Exercise	Alcoholic	Regular	High Class
Following	Regular Exercise	Alcoholic	Regular	High Class
Not Following	Regular Exercise	Alcoholic	Regular	Middle Class
Occasionally	No Exercise	Non Alcoholic	Regular	High Class
Following	No Exercise	Non Alcoholic	Non Smoker	Poor
Not Following	Occasionally	Alcoholic	Non Smoker	Poor
Not Following	Occasionally	Non Alcoholic	Regular	Middle Class
Following	Occasionally	Non Alcoholic	Regular	Middle Class
Following	No Exercise	Non Alcoholic	Non Smoker	Poor
Following	No Exercise	Non Alcoholic	Regular	Poor
Following	No Exercise	Occasionally	Non Smoker	High Class
Occasionally	No Exercise	Occasionally	Non Smoker	High Class
Occasionally	Regular Exercise	Occasionally	Non Smoker	High Class

Following	Regular Exercise	Non Alcoholic	Occasionally	High Class
Following	Occasionally	Non Alcoholic	Occasionally	Middle Class
Not Following	No Exercise	Non Alcoholic	Occasionally	High Class
Not Following	No Exercise	Alcoholic	Non Smoker	Middle Class
Not Following	No Exercise	Alcoholic	Non Smoker	High Class
Not Following	No Exercise	Alcoholic	Non Smoker	Middle Class
Not Following	No Exercise	Alcoholic	Non Smoker	High Class
Occasionally	No Exercise	Alcoholic	Non Smoker	Middle Class
Not Following	No Exercise	Alcoholic	Occasionally	Poor
Not Following	Regular Exercise	Alcoholic	Occasionally	Middle Class
Following	Regular Exercise	Alcoholic	Occasionally	Poor
Following	No Exercise	Alcoholic	Non Smoker	Poor
Following	No Exercise	Occasionally	Non Smoker	Poor
Occasionally	Occasionally	Occasionally	Non Smoker	High Class
Occasionally	Occasionally	Occasionally	Regular	Poor
Not Following	No Exercise	Occasionally	Regular	High Class
Not Following	Regular Exercise	Occasionally	Regular	Poor
Not Following	No Exercise	Occasionally	Non Smoker	High Class
Occasionally	Occasionally	Non Alcoholic	Non Smoker	High Class
Occasionally	No Exercise	Non Alcoholic	Non Smoker	Middle Class
Following	Regular Exercise	Non Alcoholic	Non Smoker	High Class
Following	Occasionally	Non Alcoholic	Regular	Middle Class
Following	No Exercise	Non Alcoholic	Regular	High Class
Not Following	Occasionally	Non Alcoholic	Regular	Middle Class
Occasionally	Regular Exercise	Non Alcoholic	Occasionally	Poor
Following	No Exercise	Non Alcoholic	Non Smoker	High Class
Following	Regular Exercise	Non Alcoholic	Non Smoker	Middle Class
Following	Regular Exercise	Non Alcoholic	Non Smoker	High Class
Following	No Exercise	Non Alcoholic	Non Smoker	Middle Class
Following	No Exercise	Occasionally	Regular	Poor
Occasionally	No Exercise	Occasionally	Regular	High Class
Occasionally	No Exercise	Occasionally	Regular	Middle Class
Following	Regular Exercise	Occasionally	Occasionally	High Class
Not Following	Regular Exercise	Occasionally	Occasionally	High Class
Not Following	Occasionally	Occasionally	Occasionally	High Class
Following	Occasionally	Alcoholic	Regular	High Class
Following	Regular Exercise	Alcoholic	Regular	Poor
Occasionally	No Exercise	Alcoholic	Non Smoker	Poor
Occasionally	Regular Exercise	Non Alcoholic	Regular	High Class
Not Following	Regular Exercise	Non Alcoholic	Regular	Poor
Not Following	Occasionally	Non Alcoholic	Non Smoker	High Class
Occasionally	Occasionally	Occasionally	Non Smoker	Poor
Occasionally	Regular Exercise	Occasionally	Non Smoker	High Class
Occasionally	Occasionally	Non Alcoholic	Non Smoker	High Class
Occasionally	Occasionally	Occasionally	Occasionally	Poor
Occasionally	Regular Exercise	Occasionally	Occasionally	High Class
Occasionally	No Exercise	Non Alcoholic	Regular	High Class

Occasionally	No Exercise	Alcoholic	Regular	High Class
Occasionally	No Exercise	Alcoholic	Regular	Middle Class
Occasionally	No Exercise	Non Alcoholic	Regular	Middle Class
Occasionally	No Exercise	Occasionally	Regular	Poor
Occasionally	Occasionally	Occasionally	Non Smoker	Middle Class
Occasionally	Occasionally	Alcoholic	Non Smoker	Poor
Occasionally	Regular Exercise	Non Alcoholic	Non Smoker	Poor
Occasionally	Regular Exercise	Alcoholic	Regular	Poor
Occasionally	Regular Exercise	Non Alcoholic	Non Smoker	Middle Class
Occasionally	Regular Exercise	Non Alcoholic	Occasionally	Middle Class
Not Following	Regular Exercise	Non Alcoholic	Non Smoker	Poor
Occasionally	Regular Exercise	Non Alcoholic	Non Smoker	Poor
Following	Regular Exercise	Non Alcoholic	Non Smoker	Middle Class
Not Following	Regular Exercise	Non Alcoholic	Non Smoker	Poor
Following	Regular Exercise	Non Alcoholic	Non Smoker	Poor
Following	Regular Exercise	Non Alcoholic	Non Smoker	Middle Class
Following	Regular Exercise	Non Alcoholic	Occasionally	Middle Class
Following	No Exercise	Alcoholic	Non Smoker	Poor
Following	No Exercise	Non Alcoholic	Non Smoker	Poor
Following	No Exercise	Non Alcoholic	Regular	High Class
Following	Regular Exercise	Alcoholic	Regular	High Class
Following	No Exercise	Occasionally	Regular	High Class
Following	No Exercise	Alcoholic	Regular	Poor
Occasionally	No Exercise	Occasionally	Regular	Poor
Not Following	Regular Exercise	Non Alcoholic	Occasionally	Poor
Following	Regular Exercise	Non Alcoholic	Regular	High Class
Not Following	No Exercise	Occasionally	Occasionally	Middle Class
Occasionally	Regular Exercise	Non Alcoholic	Non Smoker	Poor
Following	No Exercise	Non Alcoholic	Non Smoker	Middle Class
Occasionally	No Exercise	Non Alcoholic	Occasionally	Middle Class
Occasionally	Occasionally	Non Alcoholic	Occasionally	Poor
Following	Regular Exercise	Non Alcoholic	Regular	Poor
Occasionally	Occasionally	Non Alcoholic	Regular	Poor
Not Following	No Exercise	Non Alcoholic	Occasionally	Poor
Following	Regular Exercise	Occasionally	Occasionally	Poor
Not Following	Occasionally	Non Alcoholic	Regular	Middle Class
Following	Regular Exercise	Alcoholic	Regular	Middle Class
Not Following	Regular Exercise	Alcoholic	Regular	Poor
Not Following	No Exercise	Non Alcoholic	Non Smoker	Poor
Following	Regular Exercise	Occasionally	Non Smoker	High Class
Occasionally	No Exercise	Occasionally	Regular	High Class
Not Following	Regular Exercise	Non Alcoholic	Regular	High Class
Occasionally	No Exercise	Non Alcoholic	Occasionally	Middle Class
Not Following	No Exercise	Non Alcoholic	Non Smoker	High Class
Following	No Exercise	Non Alcoholic	Regular	Poor
Following	Regular Exercise	Non Alcoholic	Non Smoker	High Class
Following	No Exercise	Non Alcoholic	Non Smoker	Poor

Following	No Exercise	Non Alcoholic	Non Smoker	High Class
Following	Regular Exercise	Non Alcoholic	Non Smoker	High Class
Following	Regular Exercise	Non Alcoholic	Regular	Middle Class
Occasionally	No Exercise	Alcoholic	Regular	High Class
Following	No Exercise	Non Alcoholic	Non Smoker	Poor
Occasionally	No Exercise	Non Alcoholic	Non Smoker	High Class
Not Following	No Exercise	Non Alcoholic	Occasionally	Poor
Occasionally	Occasionally	Non Alcoholic	Non Smoker	High Class
Occasionally	No Exercise	Non Alcoholic	Non Smoker	High Class
Occasionally	No Exercise	Non Alcoholic	Non Smoker	High Class
Not Following	Regular Exercise	Occasionally	Regular	Poor
Occasionally	Occasionally	Non Alcoholic	Non Smoker	High Class
Occasionally	No Exercise	Non Alcoholic	Non Smoker	High Class
Not Following	No Exercise	Non Alcoholic	Occasionally	High Class
Occasionally	No Exercise	Non Alcoholic	Non Smoker	Middle Class
Occasionally	No Exercise	Non Alcoholic	Non Smoker	Poor
Not Following	No Exercise	Occasionally	Non Smoker	Poor
Not Following	No Exercise	Alcoholic	Regular	High Class
Occasionally	Regular Exercise	Non Alcoholic	Non Smoker	Poor
Occasionally	No Exercise	Occasionally	Non Smoker	Middle Class
Occasionally	No Exercise	Non Alcoholic	Non Smoker	Middle Class
Occasionally	Occasionally	Non Alcoholic	Occasionally	Poor
Not Following	Regular Exercise	Non Alcoholic	Occasionally	Poor
Occasionally	No Exercise	Occasionally	Non Smoker	High Class
Occasionally	Regular Exercise	Non Alcoholic	Occasionally	High Class
Occasionally	No Exercise	Occasionally	Non Smoker	High Class
Occasionally	No Exercise	Occasionally	Non Smoker	High Class
Following	No Exercise	Non Alcoholic	Regular	Middle Class
Following	No Exercise	Non Alcoholic	Non Smoker	High Class
Occasionally	No Exercise	Occasionally	Non Smoker	Middle Class
Not Following	Regular Exercise	Non Alcoholic	Non Smoker	High Class
Occasionally	Occasionally	Occasionally	Non Smoker	Middle Class
Following	No Exercise	Occasionally	Non Smoker	High Class
Occasionally	Occasionally	Occasionally	Non Smoker	Middle Class
Occasionally	Occasionally	Non Alcoholic	Non Smoker	Poor
Occasionally	No Exercise	Non Alcoholic	Non Smoker	Middle Class
Occasionally	No Exercise	Non Alcoholic	Non Smoker	Poor
Following	Occasionally	Non Alcoholic	Occasionally	Poor
Not Following	No Exercise	Alcoholic	Occasionally	Poor
Following	No Exercise	Non Alcoholic	Non Smoker	High Class
Not Following	No Exercise	Occasionally	Non Smoker	Poor
Following	Occasionally	Occasionally	Non Smoker	High Class
Not Following	No Exercise	Alcoholic	Occasionally	Poor
Occasionally	No Exercise	Occasionally	Non Smoker	High Class
Not Following	No Exercise	Non Alcoholic	Regular	High Class
Occasionally	No Exercise	Occasionally	Non Smoker	Middle Class
Occasionally	Occasionally	Non Alcoholic	Non Smoker	High Class

Occasionally	No Exercise	Non Alcoholic	Non Smoker	Middle Class
Following	No Exercise	Occasionally	Non Smoker	High Class
Occasionally	No Exercise	Non Alcoholic	Non Smoker	Middle Class
Following	Occasionally	Non Alcoholic	Non Smoker	Poor
Following	No Exercise	Non Alcoholic	Regular	Middle Class
Following	No Exercise	Non Alcoholic	Regular	Middle Class
Following	No Exercise	Non Alcoholic	Regular	Poor
Not Following	Regular Exercise	Non Alcoholic	Non Smoker	Middle Class
Not Following	No Exercise	Non Alcoholic	Non Smoker	Poor
Not Following	Occasionally	Non Alcoholic	Occasionally	Poor
Not Following	Regular Exercise	Occasionally	Occasionally	Poor
Not Following	No Exercise	Non Alcoholic	Non Smoker	Middle Class
Following	Regular Exercise	Occasionally	Occasionally	Middle Class
Not Following	No Exercise	Non Alcoholic	Regular	Poor
Following	Regular Exercise	Non Alcoholic	Occasionally	Poor
Following	No Exercise	Non Alcoholic	Regular	Middle Class
Following	Regular Exercise	Non Alcoholic	Regular	Poor
Not Following	Regular Exercise	Non Alcoholic	Occasionally	Poor
Following	Regular Exercise	Non Alcoholic	Regular	Middle Class
Following	No Exercise	Non Alcoholic	Regular	Middle Class
Not Following	Regular Exercise	Non Alcoholic	Regular	Poor
Not Following	No Exercise	Non Alcoholic	Occasionally	Poor
Following	Regular Exercise	Non Alcoholic	Regular	High Class
Not Following	Regular Exercise	Non Alcoholic	Regular	High Class
Following	Regular Exercise	Non Alcoholic	Regular	High Class
Following	Regular Exercise	Non Alcoholic	Regular	Poor
Not Following	Regular Exercise	Occasionally	Regular	Poor
Following	No Exercise	Non Alcoholic	Regular	Poor
Not Following	Regular Exercise	Non Alcoholic	Regular	High Class
Following	No Exercise	Non Alcoholic	Regular	Middle Class
Not Following	Regular Exercise	Non Alcoholic	Occasionally	Poor
Not Following	Regular Exercise	Non Alcoholic	Regular	Middle Class
Not Following	No Exercise	Non Alcoholic	Regular	Middle Class
Not Following	Regular Exercise	Non Alcoholic	Occasionally	Poor
Not Following	No Exercise	Non Alcoholic	Regular	Poor
Following	Regular Exercise	Non Alcoholic	Occasionally	Poor
Following	No Exercise	Non Alcoholic	Regular	Poor
Occasionally	Regular Exercise	Non Alcoholic	Regular	Poor
Not Following	No Exercise	Non Alcoholic	Occasionally	Middle Class
Occasionally	Regular Exercise	Non Alcoholic	Regular	Middle Class
Occasionally	Regular Exercise	Non Alcoholic	Regular	Poor
Not Following	No Exercise	Non Alcoholic	Regular	Poor
Occasionally	Regular Exercise	Non Alcoholic	Occasionally	High Class
Occasionally	No Exercise	Occasionally	Regular	High Class
Following	Regular Exercise	Non Alcoholic	Regular	High Class
Not Following	Regular Exercise	Non Alcoholic	Regular	Middle Class
Not Following	No Exercise	Non Alcoholic	Regular	High Class

Following	Regular Exercise	Non Alcoholic	Regular	Poor
Occasionally	No Exercise	Non Alcoholic	Regular	High Class
Occasionally	Regular Exercise	Non Alcoholic	Regular	Poor
Following	No Exercise	Non Alcoholic	Regular	High Class
Occasionally	Regular Exercise	Non Alcoholic	Occasionally	High Class
Not Following	No Exercise	Alcoholic	Regular	Middle Class
Not Following	Regular Exercise	Non Alcoholic	Regular	High Class
Following	Regular Exercise	Non Alcoholic	Occasionally	Poor
Following	Occasionally	Non Alcoholic	Regular	High Class
Following	Regular Exercise	Non Alcoholic	Occasionally	Poor
Not Following	Regular Exercise	Non Alcoholic	Non Smoker	High Class
Not Following	No Exercise	Non Alcoholic	Regular	High Class
Occasionally	Regular Exercise	Non Alcoholic	Occasionally	High Class
Not Following	No Exercise	Non Alcoholic	Regular	Poor
Not Following	Regular Exercise	Non Alcoholic	Regular	High Class
Not Following	Regular Exercise	Alcoholic	Regular	High Class
Occasionally	Occasionally	Non Alcoholic	Occasionally	High Class
Occasionally	No Exercise	Non Alcoholic	Regular	Middle Class
Occasionally	Regular Exercise	Non Alcoholic	Regular	Poor
Not Following	No Exercise	Non Alcoholic	Regular	Poor
Following	Regular Exercise	Non Alcoholic	Regular	High Class
Following	No Exercise	Non Alcoholic	Regular	Poor
Following	Regular Exercise	Non Alcoholic	Regular	Middle Class
Following	Regular Exercise	Non Alcoholic	Regular	Middle Class
Following	No Exercise	Non Alcoholic	Regular	Poor
Occasionally	Regular Exercise	Non Alcoholic	Occasionally	Poor
Following	No Exercise	Alcoholic	Regular	High Class
Not Following	Regular Exercise	Non Alcoholic	Regular	High Class
Following	No Exercise	Non Alcoholic	Occasionally	High Class
Not Following	Regular Exercise	Non Alcoholic	Non Smoker	High Class
Not Following	Occasionally	Non Alcoholic	Occasionally	Middle Class
Not Following	Regular Exercise	Non Alcoholic	Regular	High Class
Not Following	Regular Exercise	Non Alcoholic	Regular	Middle Class
Following	No Exercise	Non Alcoholic	Regular	Middle Class
Not Following	Regular Exercise	Occasionally	Regular	Middle Class
Following	No Exercise	Non Alcoholic	Occasionally	Middle Class
Following	Regular Exercise	Non Alcoholic	Regular	Middle Class
Not Following	No Exercise	Non Alcoholic	Non Smoker	Middle Class
Following	Regular Exercise	Non Alcoholic	Regular	Middle Class
Following	No Exercise	Non Alcoholic	Regular	Middle Class
Not Following	Regular Exercise	Non Alcoholic	Regular	Middle Class
Not Following	Occasionally	Non Alcoholic	Regular	Middle Class
Not Following	Occasionally	Non Alcoholic	Regular	Middle Class
Following	Regular Exercise	Non Alcoholic	Regular	Poor
Following	No Exercise	Non Alcoholic	Occasionally	Poor
Following	No Exercise	Non Alcoholic	Regular	Poor
Following	Regular Exercise	Non Alcoholic	Regular	Poor

Not Following	Regular Exercise	Non Alcoholic	Occasionally	High Class
Following	No Exercise	Non Alcoholic	Regular	High Class
Following	No Exercise	Occasionally	Occasionally	High Class
Following	No Exercise	Non Alcoholic	Regular	High Class
Not Following	No Exercise	Non Alcoholic	Regular	High Class
Not Following	Regular Exercise	Non Alcoholic	Occasionally	High Class
Following	No Exercise	Non Alcoholic	Regular	High Class
Following	Regular Exercise	Non Alcoholic	Regular	High Class
Not Following	Regular Exercise	Non Alcoholic	Regular	High Class
Following	No Exercise	Non Alcoholic	Occasionally	High Class
Following	Occasionally	Non Alcoholic	Regular	High Class
Not Following	No Exercise	Non Alcoholic	Regular	High Class
Following	Regular Exercise	Non Alcoholic	Regular	High Class
Not Following	No Exercise	Non Alcoholic	Regular	High Class
Following	Regular Exercise	Non Alcoholic	Regular	Poor
Following	Regular Exercise	Non Alcoholic	Regular	Poor
Not Following	Regular Exercise	Non Alcoholic	Regular	Poor
Following	Regular Exercise	Non Alcoholic	Regular	Poor
Following	Regular Exercise	Non Alcoholic	Occasionally	Poor
Not Following	Regular Exercise	Non Alcoholic	Regular	Middle Class
Occasionally	Occasionally	Non Alcoholic	Regular	Poor
Occasionally	Regular Exercise	Non Alcoholic	Occasionally	Poor
Occasionally	Regular Exercise	Non Alcoholic	Regular	Poor
Not Following	Regular Exercise	Non Alcoholic	Occasionally	Middle Class
Occasionally	Regular Exercise	Non Alcoholic	Regular	Middle Class
Not Following	Regular Exercise	Non Alcoholic	Regular	Middle Class
Occasionally	Regular Exercise	Non Alcoholic	Occasionally	High Class
Not Following	Regular Exercise	Non Alcoholic	Regular	Poor
Following	Regular Exercise	Non Alcoholic	Regular	Poor
Not Following	Regular Exercise	Non Alcoholic	Regular	Poor
Not Following	Regular Exercise	Non Alcoholic	Occasionally	Middle Class
Following	Regular Exercise	Non Alcoholic	Regular	Poor
Not Following	Regular Exercise	Non Alcoholic	Regular	Middle Class
Occasionally	Regular Exercise	Non Alcoholic	Regular	Middle Class
Not Following	Regular Exercise	Non Alcoholic	Non Smoker	Middle Class
Following	No Exercise	Non Alcoholic	Regular	Poor
Following	No Exercise	Non Alcoholic	Regular	Poor
Not Following	No Exercise	Non Alcoholic	Regular	Middle Class
Following	No Exercise	Non Alcoholic	Regular	Poor
Not Following	Regular Exercise	Non Alcoholic	Regular	Poor
Following	Regular Exercise	Non Alcoholic	Occasionally	Poor
Not Following	Regular Exercise	Non Alcoholic	Regular	Middle Class
Not Following	No Exercise	Non Alcoholic	Regular	Middle Class
Not Following	Regular Exercise	Non Alcoholic	Occasionally	Middle Class
Following	No Exercise	Non Alcoholic	Regular	Poor
Not Following	Regular Exercise	Non Alcoholic	Non Smoker	Poor
Not Following	No Exercise	Alcoholic	Regular	Poor

Not Following	Regular Exercise	Non Alcoholic	Regular	Poor
Following	No Exercise	Non Alcoholic	Occasionally	High Class
Not Following	Regular Exercise	Non Alcoholic	Regular	High Class
Following	Regular Exercise	Non Alcoholic	Regular	Poor
Not Following	Regular Exercise	Non Alcoholic	Regular	Poor
Not Following	Regular Exercise	Non Alcoholic	Occasionally	Poor
Following	Regular Exercise	Occasionally	Regular	Poor
Not Following	Regular Exercise	Non Alcoholic	Regular	High Class
Following	Regular Exercise	Non Alcoholic	Regular	High Class
Following	Regular Exercise	Non Alcoholic	Regular	Poor
Occasionally	Regular Exercise	Non Alcoholic	Regular	Middle Class
Following	Regular Exercise	Non Alcoholic	Regular	Poor
Following	Regular Exercise	Non Alcoholic	Regular	Poor
Following	Regular Exercise	Non Alcoholic	Regular	Middle Class
Occasionally	Regular Exercise	Non Alcoholic	Occasionally	Middle Class
Not Following	Regular Exercise	Non Alcoholic	Regular	Poor
Following	Regular Exercise	Non Alcoholic	Regular	Poor
Occasionally	No Exercise	Non Alcoholic	Non Smoker	Poor
Not Following	Occasionally	Non Alcoholic	Regular	Middle Class
Following	Occasionally	Non Alcoholic	Occasionally	High Class
Occasionally	Regular Exercise	Non Alcoholic	Regular	Poor
Following	No Exercise	Non Alcoholic	Regular	High Class
Following	Regular Exercise	Non Alcoholic	Occasionally	Middle Class
Not Following	No Exercise	Non Alcoholic	Regular	Poor
Not Following	Regular Exercise	Alcoholic	Regular	High Class
Not Following	No Exercise	Non Alcoholic	Regular	High Class
Not Following	Regular Exercise	Non Alcoholic	Occasionally	High Class
Following	Occasionally	Non Alcoholic	Regular	Poor
Following	Regular Exercise	Non Alcoholic	Regular	Poor
Following	Occasionally	Non Alcoholic	Regular	Poor
Not Following	Regular Exercise	Non Alcoholic	Regular	High Class
Not Following	Regular Exercise	Non Alcoholic	Regular	Poor
Not Following	No Exercise	Non Alcoholic	Regular	Poor
Following	No Exercise	Non Alcoholic	Regular	Poor
Not Following	Regular Exercise	Alcoholic	Regular	Poor
Following	No Exercise	Non Alcoholic	Occasionally	High Class
Not Following	Regular Exercise	Non Alcoholic	Regular	High Class
Following	Occasionally	Non Alcoholic	Regular	Poor
Following	Regular Exercise	Non Alcoholic	Occasionally	Poor
Not Following	Occasionally	Non Alcoholic	Regular	Poor
Following	Regular Exercise	Non Alcoholic	Occasionally	Poor
Not Following	No Exercise	Non Alcoholic	Regular	High Class
Not Following	Regular Exercise	Non Alcoholic	Regular	High Class
Following	Occasionally	Non Alcoholic	Occasionally	Poor
Not Following	No Exercise	Non Alcoholic	Regular	Middle Class
Not Following	Regular Exercise	Non Alcoholic	Regular	Poor
Not Following	No Exercise	Non Alcoholic	Regular	Poor

Following	No Exercise	Alcoholic	Occasionally	Middle Class
Not Following	Regular Exercise	Non Alcoholic	Non Smoker	Middle Class
Following	Regular Exercise	Non Alcoholic	Regular	Poor
Not Following	No Exercise	Non Alcoholic	Regular	Poor
Not Following	Regular Exercise	Non Alcoholic	Regular	Poor
Occasionally	Regular Exercise	Non Alcoholic	Regular	Middle Class
Occasionally	Occasionally	Non Alcoholic	Regular	High Class
Not Following	Regular Exercise	Non Alcoholic	Regular	Poor
Occasionally	No Exercise	Non Alcoholic	Regular	High Class
Not Following	Regular Exercise	Non Alcoholic	Occasionally	Middle Class
Following	Regular Exercise	Non Alcoholic	Regular	Poor
Not Following	Occasionally	Alcoholic	Regular	Poor
Following	No Exercise	Non Alcoholic	Occasionally	Poor
Following	No Exercise	Non Alcoholic	Regular	Poor
Following	Regular Exercise	Non Alcoholic	Occasionally	Poor
Not Following	No Exercise	Non Alcoholic	Regular	Middle Class
Not Following	Regular Exercise	Non Alcoholic	Regular	Middle Class
Not Following	Regular Exercise	Non Alcoholic	Occasionally	Middle Class
Following	Regular Exercise	Non Alcoholic	Regular	Middle Class
Not Following	No Exercise	Non Alcoholic	Regular	Middle Class
Following	Regular Exercise	Non Alcoholic	Regular	Middle Class
Following	Regular Exercise	Occasionally	Occasionally	Middle Class
Not Following	Regular Exercise	Non Alcoholic	Regular	Middle Class
Following	Regular Exercise	Non Alcoholic	Regular	Poor
Following	Regular Exercise	Non Alcoholic	Regular	Poor
Following	Regular Exercise	Non Alcoholic	Regular	Poor
Occasionally	Occasionally	Non Alcoholic	Regular	Middle Class
Not Following	No Exercise	Non Alcoholic	Occasionally	Middle Class
Occasionally	Regular Exercise	Non Alcoholic	Regular	Middle Class
Occasionally	Regular Exercise	Non Alcoholic	Regular	Middle Class
Following	Regular Exercise	Non Alcoholic	Occasionally	Middle Class
Not Following	Regular Exercise	Occasionally	Non Smoker	Poor
Following	No Exercise	Non Alcoholic	Regular	Poor
Not Following	Regular Exercise	Non Alcoholic	Occasionally	High Class
Following	No Exercise	Occasionally	Regular	Poor
Following	Occasionally	Occasionally	Non Smoker	Poor
Occasionally	Occasionally	Non Alcoholic	Regular	Middle Class
Not Following	Regular Exercise	Non Alcoholic	Regular	High Class
Occasionally	No Exercise	Non Alcoholic	Occasionally	High Class
Occasionally	Regular Exercise	Non Alcoholic	Regular	Middle Class
Occasionally	No Exercise	Non Alcoholic	Regular	High Class
Occasionally	Regular Exercise	Non Alcoholic	Non Smoker	High Class
Occasionally	Regular Exercise	Non Alcoholic	Regular	High Class
Not Following	Regular Exercise	Alcoholic	Occasionally	Middle Class
Not Following	Regular Exercise	Non Alcoholic	Regular	High Class
Following	No Exercise	Non Alcoholic	Regular	Poor
Not Following	Regular Exercise	Non Alcoholic	Occasionally	High Class

Following	No Exercise	Non Alcoholic	Regular	Poor
Occasionally	No Exercise	Non Alcoholic	Non Smoker	Poor
Occasionally	Regular Exercise	Non Alcoholic	Regular	High Class
Occasionally	No Exercise	Non Alcoholic	Non Smoker	Poor
Occasionally	Regular Exercise	Non Alcoholic	Regular	Poor
Occasionally	No Exercise	Alcoholic	Regular	High Class
Occasionally	Occasionally	Non Alcoholic	Regular	Middle Class
Following	Occasionally	Occasionally	Regular	High Class
Occasionally	No Exercise	Occasionally	Occasionally	Middle Class
Occasionally	No Exercise	Non Alcoholic	Regular	Middle Class
Not Following	Regular Exercise	Non Alcoholic	Regular	High Class
Not Following	Regular Exercise	Occasionally	Occasionally	High Class
Occasionally	No Exercise	Non Alcoholic	Regular	Poor
Not Following	Regular Exercise	Non Alcoholic	Occasionally	Poor
Occasionally	Regular Exercise	Non Alcoholic	Regular	Poor
Occasionally	No Exercise	Non Alcoholic	Non Smoker	Middle Class
Occasionally	Regular Exercise	Non Alcoholic	Occasionally	High Class
Occasionally	No Exercise	Non Alcoholic	Regular	High Class
Occasionally	No Exercise	Occasionally	Regular	High Class
Occasionally	No Exercise	Non Alcoholic	Regular	Poor
Occasionally	Regular Exercise	Occasionally	Occasionally	Poor
Occasionally	Regular Exercise	Occasionally	Regular	Poor
Occasionally	No Exercise	Occasionally	Regular	High Class
Following	Regular Exercise	Occasionally	Regular	Poor
Following	No Exercise	Non Alcoholic	Non Smoker	Poor
Not Following	Regular Exercise	Non Alcoholic	Occasionally	High Class
Following	No Exercise	Non Alcoholic	Regular	Middle Class
Occasionally	Regular Exercise	Non Alcoholic	Regular	Middle Class
Occasionally	Regular Exercise	Occasionally	Occasionally	High Class
Occasionally	Regular Exercise	Non Alcoholic	Regular	High Class
Occasionally	No Exercise	Non Alcoholic	Occasionally	Poor
Occasionally	No Exercise	Non Alcoholic	Regular	Poor
Occasionally	Occasionally	Non Alcoholic	Regular	Poor
Occasionally	No Exercise	Non Alcoholic	Occasionally	Middle Class
Occasionally	No Exercise	Non Alcoholic	Regular	High Class
Following	No Exercise	Occasionally	Regular	High Class
Occasionally	Occasionally	Non Alcoholic	Regular	Middle Class
Occasionally	No Exercise	Non Alcoholic	Occasionally	Middle Class
Occasionally	Regular Exercise	Non Alcoholic	Regular	Middle Class
Occasionally	No Exercise	Non Alcoholic	Regular	High Class
Not Following	No Exercise	Non Alcoholic	Regular	Poor
Occasionally	Regular Exercise	Non Alcoholic	Regular	Middle Class
Not Following	No Exercise	Non Alcoholic	Occasionally	Poor
Not Following	No Exercise	Occasionally	Non Smoker	Poor
Not Following	No Exercise	Non Alcoholic	Regular	Poor
Not Following	Occasionally	Non Alcoholic	Occasionally	Poor
Following	No Exercise	Non Alcoholic	Regular	Poor

Occasionally	Regular Exercise	Non Alcoholic	Occasionally	High Class
Occasionally	Regular Exercise	Non Alcoholic	Regular	Middle Class
Occasionally	No Exercise	Non Alcoholic	Regular	High Class
Not Following	No Exercise	Non Alcoholic	Occasionally	Middle Class
Occasionally	Regular Exercise	Non Alcoholic	Regular	High Class
Following	No Exercise	Occasionally	Regular	Poor
Not Following	Regular Exercise	Occasionally	Regular	Middle Class
Not Following	No Exercise	Non Alcoholic	Occasionally	Poor
Not Following	Regular Exercise	Non Alcoholic	Regular	Middle Class
Not Following	Occasionally	Non Alcoholic	Regular	Poor
Not Following	Regular Exercise	Occasionally	Regular	Poor
Not Following	No Exercise	Occasionally	Regular	Poor
Occasionally	Regular Exercise	Occasionally	Non Smoker	High Class
Occasionally	No Exercise	Occasionally	Regular	High Class
Not Following	Regular Exercise	Occasionally	Regular	High Class
Occasionally	Regular Exercise	Occasionally	Occasionally	High Class
Not Following	Regular Exercise	Non Alcoholic	Regular	High Class
Occasionally	Occasionally	Non Alcoholic	Occasionally	Middle Class
Not Following	Regular Exercise	Non Alcoholic	Regular	Poor
Occasionally	Regular Exercise	Non Alcoholic	Regular	High Class
Not Following	No Exercise	Non Alcoholic	Occasionally	High Class
Occasionally	No Exercise	Non Alcoholic	Regular	Middle Class
Not Following	No Exercise	Non Alcoholic	Regular	High Class
Following	Regular Exercise	Non Alcoholic	Regular	High Class
Occasionally	Regular Exercise	Non Alcoholic	Occasionally	Poor
Not Following	Regular Exercise	Non Alcoholic	Regular	Poor
Occasionally	Regular Exercise	Non Alcoholic	Regular	Middle Class
Occasionally	Occasionally	Non Alcoholic	Regular	Middle Class
Not Following	Regular Exercise	Non Alcoholic	Regular	Middle Class
Occasionally	Regular Exercise	Non Alcoholic	Non Smoker	Middle Class
Occasionally	No Exercise	Non Alcoholic	Regular	Middle Class
Not Following	No Exercise	Non Alcoholic	Regular	High Class
Following	No Exercise	Occasionally	Occasionally	High Class
Following	Occasionally	Non Alcoholic	Regular	Middle Class
Occasionally	No Exercise	Non Alcoholic	Occasionally	Middle Class
Occasionally	No Exercise	Non Alcoholic	Regular	High Class
Occasionally	Regular Exercise	Non Alcoholic	Regular	Poor
Occasionally	No Exercise	Non Alcoholic	Occasionally	Poor
Occasionally	No Exercise	Non Alcoholic	Regular	High Class
Following	No Exercise	Non Alcoholic	Regular	High Class
Occasionally	No Exercise	Non Alcoholic	Regular	Poor
Occasionally	Occasionally	Non Alcoholic	Occasionally	Middle Class
Occasionally	No Exercise	Non Alcoholic	Regular	Poor
Following	No Exercise	Non Alcoholic	Regular	Poor
Following	Occasionally	Non Alcoholic	Regular	Middle Class
Not Following	No Exercise	Non Alcoholic	Non Smoker	Poor
Following	No Exercise	Occasionally	Occasionally	Middle Class

Following	No Exercise	Non Alcoholic	Regular	Middle Class
Not Following	Regular Exercise	Non Alcoholic	Regular	Poor
Not Following	Regular Exercise	Non Alcoholic	Occasionally	Middle Class
Occasionally	Occasionally	Non Alcoholic	Regular	Poor
Occasionally	Regular Exercise	Non Alcoholic	Occasionally	Poor
Not Following	Regular Exercise	Non Alcoholic	Regular	High Class
Not Following	No Exercise	Non Alcoholic	Regular	Poor
Occasionally	Occasionally	Non Alcoholic	Occasionally	High Class
Not Following	Regular Exercise	Occasionally	Regular	High Class
Not Following	No Exercise	Non Alcoholic	Regular	High Class
Not Following	Regular Exercise	Non Alcoholic	Regular	Middle Class
Not Following	No Exercise	Non Alcoholic	Occasionally	Middle Class
Occasionally	Regular Exercise	Non Alcoholic	Regular	Middle Class
Not Following	No Exercise	Non Alcoholic	Regular	Middle Class
Occasionally	Regular Exercise	Non Alcoholic	Regular	Middle Class
Not Following	No Exercise	Non Alcoholic	Regular	Poor
Not Following	No Exercise	Non Alcoholic	Occasionally	Poor
Occasionally	No Exercise	Non Alcoholic	Regular	High Class
Following	Regular Exercise	Non Alcoholic	Regular	Poor
Following	Regular Exercise	Non Alcoholic	Occasionally	Middle Class
Following	Regular Exercise	Non Alcoholic	Non Smoker	Middle Class
Not Following	Occasionally	Occasionally	Occasionally	Poor
Occasionally	No Exercise	Non Alcoholic	Regular	Poor
Occasionally	No Exercise	Non Alcoholic	Regular	High Class
Following	Occasionally	Non Alcoholic	Occasionally	Poor
Following	No Exercise	Non Alcoholic	Regular	Poor
Following	No Exercise	Non Alcoholic	Regular	Poor
Following	Occasionally	Non Alcoholic	Regular	Middle Class
Occasionally	No Exercise	Non Alcoholic	Occasionally	High Class
Occasionally	Occasionally	Non Alcoholic	Regular	Poor
Following	No Exercise	Non Alcoholic	Regular	High Class
Not Following	Regular Exercise	Alcoholic	Regular	Middle Class
Following	Regular Exercise	Non Alcoholic	Non Smoker	Poor
Following	Regular Exercise	Non Alcoholic	Occasionally	High Class
Not Following	Regular Exercise	Occasionally	Regular	Poor
Following	No Exercise	Non Alcoholic	Regular	High Class
Not Following	Regular Exercise	Non Alcoholic	Occasionally	Poor
Following	No Exercise	Occasionally	Regular	High Class
Occasionally	Regular Exercise	Non Alcoholic	Occasionally	Poor
Following	No Exercise	Non Alcoholic	Regular	Middle Class
Occasionally	Regular Exercise	Non Alcoholic	Regular	Middle Class
Following	No Exercise	Non Alcoholic	Occasionally	Middle Class
Following	Regular Exercise	Non Alcoholic	Regular	Middle Class
Following	No Exercise	Non Alcoholic	Regular	Middle Class
Following	Regular Exercise	Non Alcoholic	Regular	Poor
Following	No Exercise	Non Alcoholic	Occasionally	High Class
Occasionally	No Exercise	Non Alcoholic	Regular	Middle Class

Occasionally	Regular Exercise	Non Alcoholic	Regular	High Class
Following	No Exercise	Non Alcoholic	Regular	Poor
Occasionally	Regular Exercise	Non Alcoholic	Regular	High Class
Following	Regular Exercise	Non Alcoholic	Occasionally	High Class
Following	Regular Exercise	Occasionally	Regular	High Class
Occasionally	No Exercise	Occasionally	Regular	Poor
Following	Regular Exercise	Occasionally	Occasionally	High Class
Occasionally	Regular Exercise	Alcoholic	Regular	Poor
Following	No Exercise	Occasionally	Occasionally	Poor
Occasionally	Regular Exercise	Occasionally	Regular	High Class
Following	No Exercise	Non Alcoholic	Regular	Poor
Following	Regular Exercise	Alcoholic	Occasionally	Poor
Following	Occasionally	Alcoholic	Regular	Middle Class
Following	Regular Exercise	Alcoholic	Regular	High Class
Occasionally	No Exercise	Non Alcoholic	Regular	Middle Class
Following	Regular Exercise	Non Alcoholic	Occasionally	Poor
Following	No Exercise	Non Alcoholic	Regular	Poor
Following	No Exercise	Non Alcoholic	Non Smoker	High Class
Occasionally	No Exercise	Non Alcoholic	Regular	High Class
Following	Regular Exercise	Non Alcoholic	Regular	High Class
Occasionally	No Exercise	Non Alcoholic	Occasionally	High Class
Following	No Exercise	Non Alcoholic	Regular	High Class
Following	No Exercise	Non Alcoholic	Regular	High Class
Occasionally	Occasionally	Occasionally	Occasionally	High Class
Occasionally	No Exercise	Non Alcoholic	Regular	Poor
Occasionally	No Exercise	Non Alcoholic	Occasionally	Poor
Occasionally	Regular Exercise	Alcoholic	Regular	Poor
Occasionally	Regular Exercise	Non Alcoholic	Regular	High Class
Occasionally	Regular Exercise	Alcoholic	Occasionally	High Class
Occasionally	No Exercise	Non Alcoholic	Regular	Poor
Occasionally	Regular Exercise	Non Alcoholic	Regular	Poor
Occasionally	No Exercise	Non Alcoholic	Regular	High Class
Occasionally	Regular Exercise	Non Alcoholic	Non Smoker	Poor
Occasionally	Regular Exercise	Non Alcoholic	Regular	High Class
Following	Regular Exercise	Non Alcoholic	Regular	Poor
Following	Regular Exercise	Non Alcoholic	Non Smoker	Poor
Occasionally	Regular Exercise	Non Alcoholic	Regular	High Class
Occasionally	No Exercise	Occasionally	Occasionally	Middle Class
Occasionally	Regular Exercise	Non Alcoholic	Regular	Poor
Occasionally	No Exercise	Non Alcoholic	Regular	High Class
Occasionally	Regular Exercise	Alcoholic	Occasionally	High Class
Following	Regular Exercise	Non Alcoholic	Regular	Poor
Occasionally	No Exercise	Alcoholic	Occasionally	High Class
Following	Regular Exercise	Occasionally	Regular	Poor
Following	No Exercise	Occasionally	Non Smoker	Poor
Following	Regular Exercise	Non Alcoholic	Occasionally	Poor
Occasionally	Regular Exercise	Alcoholic	Regular	Poor

Following	Regular Exercise	Non Alcoholic	Regular	Middle Class
Following	No Exercise	Non Alcoholic	Regular	Middle Class
Occasionally	No Exercise	Alcoholic	Occasionally	Poor
Occasionally	No Exercise	Alcoholic	Regular	High Class
Following	Regular Exercise	Non Alcoholic	Regular	High Class
Following	Regular Exercise	Non Alcoholic	Regular	Middle Class
Not Following	Regular Exercise	Non Alcoholic	Regular	Middle Class
Following	Regular Exercise	Non Alcoholic	Occasionally	Middle Class
Not Following	No Exercise	Occasionally	Regular	Poor
Following	No Exercise	Alcoholic	Regular	Poor
Following	Regular Exercise	Non Alcoholic	Occasionally	Poor
Following	Regular Exercise	Occasionally	Regular	Poor
Following	Regular Exercise	Alcoholic	Regular	High Class
Not Following	No Exercise	Non Alcoholic	Regular	High Class
Not Following	Occasionally	Occasionally	Non Smoker	High Class
Not Following	No Exercise	Alcoholic	Regular	Poor
Not Following	Regular Exercise	Non Alcoholic	Regular	High Class
Not Following	Regular Exercise	Occasionally	Regular	Poor
Not Following	Regular Exercise	Alcoholic	Regular	Middle Class
Not Following	Regular Exercise	Non Alcoholic	Occasionally	Middle Class
Not Following	Regular Exercise	Occasionally	Regular	Middle Class
Not Following	Regular Exercise	Alcoholic	Regular	Middle Class
Following	No Exercise	Non Alcoholic	Occasionally	Middle Class
Following	Regular Exercise	Non Alcoholic	Regular	Middle Class
Not Following	No Exercise	Non Alcoholic	Occasionally	High Class
Not Following	Regular Exercise	Non Alcoholic	Regular	High Class
Not Following	Regular Exercise	Non Alcoholic	Regular	High Class
Not Following	Regular Exercise	Alcoholic	Occasionally	High Class
Not Following	Regular Exercise	Non Alcoholic	Regular	Poor
Not Following	Regular Exercise	Occasionally	Regular	High Class
Occasionally	No Exercise	Occasionally	Regular	Middle Class
Occasionally	Regular Exercise	Non Alcoholic	Occasionally	Poor
Occasionally	Regular Exercise	Non Alcoholic	Non Smoker	Poor
Not Following	Regular Exercise	Non Alcoholic	Regular	Poor
Not Following	No Exercise	Non Alcoholic	Regular	Poor
Not Following	Regular Exercise	Non Alcoholic	Regular	Middle Class
Not Following	Regular Exercise	Non Alcoholic	Occasionally	Poor
Occasionally	Regular Exercise	Non Alcoholic	Regular	High Class
Occasionally	Occasionally	Non Alcoholic	Regular	High Class
Occasionally	Regular Exercise	Non Alcoholic	Regular	Middle Class
Occasionally	No Exercise	Alcoholic	Occasionally	Middle Class
Occasionally	Regular Exercise	Occasionally	Regular	Middle Class
Not Following	No Exercise	Occasionally	Regular	Middle Class
Occasionally	Regular Exercise	Non Alcoholic	Non Smoker	Poor
Occasionally	No Exercise	Occasionally	Occasionally	Poor
Occasionally	Occasionally	Occasionally	Regular	Poor
Occasionally	Regular Exercise	Non Alcoholic	Regular	High Class

Not Following	Regular Exercise	Non Alcoholic	Regular	High Class
Not Following	Occasionally	Non Alcoholic	Regular	High Class
Not Following	Regular Exercise	Non Alcoholic	Occasionally	High Class
Not Following	Regular Exercise	Non Alcoholic	Regular	Middle Class
Not Following	Regular Exercise	Occasionally	Regular	High Class
Not Following	Regular Exercise	Non Alcoholic	Occasionally	Middle Class
Not Following	No Exercise	Non Alcoholic	Regular	High Class
Not Following	Regular Exercise	Non Alcoholic	Occasionally	Middle Class
Not Following	Occasionally	Non Alcoholic	Regular	High Class
Not Following	Regular Exercise	Non Alcoholic	Regular	Poor
Not Following	No Exercise	Non Alcoholic	Occasionally	Poor
Not Following	Regular Exercise	Non Alcoholic	Regular	Middle Class
Not Following	Regular Exercise	Non Alcoholic	Regular	Middle Class
Not Following	Occasionally	Occasionally	Regular	Middle Class
Not Following	Regular Exercise	Non Alcoholic	Non Smoker	Middle Class
Not Following	Regular Exercise	Alcoholic	Regular	High Class
Not Following	Regular Exercise	Non Alcoholic	Regular	High Class
Not Following	No Exercise	Non Alcoholic	Regular	High Class
Not Following	Regular Exercise	Non Alcoholic	Regular	High Class
Not Following	Occasionally	Non Alcoholic	Occasionally	High Class
Not Following	Regular Exercise	Non Alcoholic	Regular	Middle Class
Occasionally	Occasionally	Alcoholic	Occasionally	Poor
Not Following	Occasionally	Non Alcoholic	Occasionally	Middle Class
Not Following	No Exercise	Occasionally	Regular	Middle Class
Following	Regular Exercise	Occasionally	Occasionally	High Class
Following	Regular Exercise	Occasionally	Occasionally	Poor
Following	Regular Exercise	Non Alcoholic	Regular	Middle Class
Not Following	Occasionally	Occasionally	Occasionally	High Class
Occasionally	Regular Exercise	Occasionally	Regular	Middle Class
Not Following	Occasionally	Non Alcoholic	Regular	High Class
Not Following	No Exercise	Non Alcoholic	Non Smoker	Middle Class
Following	Regular Exercise	Non Alcoholic	Non Smoker	High Class
Following	Regular Exercise	Non Alcoholic	Occasionally	Middle Class
Following	Regular Exercise	Non Alcoholic	Non Smoker	High Class
Following	Regular Exercise	Non Alcoholic	Occasionally	Poor
Following	Regular Exercise	Occasionally	Occasionally	Poor
Occasionally	No Exercise	Non Alcoholic	Regular	Poor
Occasionally	No Exercise	Non Alcoholic	Regular	Poor
Occasionally	No Exercise	Non Alcoholic	Regular	Middle Class
Following	Occasionally	Alcoholic	Regular	Middle Class
Following	No Exercise	Non Alcoholic	Regular	High Class
Occasionally	Regular Exercise	Occasionally	Regular	High Class
Occasionally	Occasionally	Alcoholic	Regular	High Class
Occasionally	Occasionally	Occasionally	Regular	Middle Class
Occasionally	Regular Exercise	Non Alcoholic	Occasionally	Middle Class
Following	Occasionally	Alcoholic	Regular	Middle Class
Occasionally	Regular Exercise	Occasionally	Regular	High Class

Occasionally	Regular Exercise	Non Alcoholic	Occasionally	High Class
Occasionally	Regular Exercise	Alcoholic	Regular	Poor
Occasionally	Regular Exercise	Non Alcoholic	Occasionally	Poor
Occasionally	Occasionally	Occasionally	Occasionally	Poor
Occasionally	No Exercise	Non Alcoholic	Non Smoker	High Class
Occasionally	Regular Exercise	Non Alcoholic	Occasionally	High Class
Following	Regular Exercise	Occasionally	Regular	High Class
Following	No Exercise	Non Alcoholic	Regular	Middle Class
Occasionally	No Exercise	Alcoholic	Regular	Middle Class
Following	Regular Exercise	Non Alcoholic	Occasionally	Middle Class
Following	Regular Exercise	Non Alcoholic	Non Smoker	Middle Class
Following	Regular Exercise	Non Alcoholic	Non Smoker	Poor
Occasionally	No Exercise	Alcoholic	Regular	Poor
Following	Regular Exercise	Non Alcoholic	Regular	High Class
Occasionally	No Exercise	Non Alcoholic	Occasionally	High Class
Occasionally	No Exercise	Non Alcoholic	Regular	High Class
Occasionally	Regular Exercise	Occasionally	Regular	Middle Class
Occasionally	Regular Exercise	Non Alcoholic	Occasionally	Middle Class
Following	Regular Exercise	Non Alcoholic	Non Smoker	Middle Class
Occasionally	No Exercise	Non Alcoholic	Non Smoker	Middle Class
Occasionally	No Exercise	Non Alcoholic	Non Smoker	Middle Class
Occasionally	Occasionally	Alcoholic	Regular	Poor
Following	Occasionally	Non Alcoholic	Non Smoker	Poor
Occasionally	No Exercise	Alcoholic	Regular	Poor
Following	Regular Exercise	Alcoholic	Regular	Poor
Occasionally	Occasionally	Occasionally	Occasionally	Poor
Occasionally	No Exercise	Alcoholic	Regular	Middle Class
Not Following	Regular Exercise	Alcoholic	Occasionally	Middle Class
Not Following	Regular Exercise	Non Alcoholic	Non Smoker	Middle Class
Occasionally	No Exercise	Occasionally	Non Smoker	Middle Class
Occasionally	No Exercise	Alcoholic	Regular	Middle Class
Occasionally	Regular Exercise	Occasionally	Regular	Middle Class
Not Following	No Exercise	Occasionally	Non Smoker	High Class
Not Following	No Exercise	Non Alcoholic	Non Smoker	High Class
Not Following	No Exercise	Alcoholic	Occasionally	High Class
Occasionally	Occasionally	Occasionally	Occasionally	Poor
Following	Regular Exercise	Non Alcoholic	Occasionally	High Class
Occasionally	Regular Exercise	Alcoholic	Regular	Poor
Occasionally	Regular Exercise	Non Alcoholic	Non Smoker	Poor
Occasionally	Regular Exercise	Non Alcoholic	Occasionally	Middle Class
Occasionally	Regular Exercise	Occasionally	Regular	High Class
Not Following	Occasionally	Non Alcoholic	Occasionally	High Class
Not Following	Regular Exercise	Non Alcoholic	Regular	Middle Class
Following	Regular Exercise	Non Alcoholic	Non Smoker	High Class
Not Following	Regular Exercise	Alcoholic	Regular	High Class
Following	Regular Exercise	Non Alcoholic	Non Smoker	High Class
Not Following	Regular Exercise	Non Alcoholic	Non Smoker	Middle Class

Occasionally	Occasionally	Occasionally	Occasionally	High Class
Occasionally	Regular Exercise	Alcoholic	Regular	Poor
Not Following	Regular Exercise	Occasionally	Non Smoker	High Class
Occasionally	Regular Exercise	Alcoholic	Regular	Poor
Following	Regular Exercise	Non Alcoholic	Regular	Poor
Following	Regular Exercise	Non Alcoholic	Occasionally	High Class
Following	Regular Exercise	Alcoholic	Regular	Poor
Following	Regular Exercise	Non Alcoholic	Regular	Poor
Not Following	Regular Exercise	Non Alcoholic	Occasionally	High Class
Following	Regular Exercise	Occasionally	Regular	Middle Class
Not Following	Regular Exercise	Occasionally	Regular	High Class
Not Following	Regular Exercise	Non Alcoholic	Regular	Middle Class
Following	Regular Exercise	Non Alcoholic	Regular	Middle Class
Occasionally	Occasionally	Alcoholic	Occasionally	High Class
Occasionally	Regular Exercise	Non Alcoholic	Non Smoker	High Class
Following	Regular Exercise	Occasionally	Regular	Poor
Following	Regular Exercise	Non Alcoholic	Non Smoker	Poor
Following	Occasionally	Occasionally	Occasionally	Poor
Occasionally	Regular Exercise	Occasionally	Regular	Middle Class
Occasionally	Occasionally	Alcoholic	Occasionally	High Class
Occasionally	No Exercise	Non Alcoholic	Non Smoker	High Class
Following	Regular Exercise	Non Alcoholic	Regular	High Class
Not Following	No Exercise	Alcoholic	Occasionally	Poor
Not Following	Occasionally	Occasionally	Regular	Poor
Occasionally	Occasionally	Non Alcoholic	Non Smoker	Poor
Not Following	Regular Exercise	Alcoholic	Non Smoker	High Class
Not Following	Regular Exercise	Occasionally	Non Smoker	Poor
Occasionally	Occasionally	Occasionally	Occasionally	Poor
Following	Regular Exercise	Alcoholic	Regular	High Class
Following	Regular Exercise	Non Alcoholic	Occasionally	Middle Class
Occasionally	No Exercise	Alcoholic	Non Smoker	Middle Class
Following	No Exercise	Alcoholic	Regular	High Class
Not Following	Occasionally	Occasionally	Occasionally	High Class
Occasionally	Occasionally	Non Alcoholic	Regular	Poor
Occasionally	Occasionally	Non Alcoholic	Occasionally	Poor
Following	Regular Exercise	Alcoholic	Regular	Poor
Following	No Exercise	Occasionally	Non Smoker	Middle Class
Following	Regular Exercise	Alcoholic	Occasionally	High Class
Not Following	Occasionally	Occasionally	Regular	High Class
Occasionally	No Exercise	Non Alcoholic	Regular	Middle Class
Occasionally	Occasionally	Occasionally	Regular	Middle Class
Following	Regular Exercise	Alcoholic	Regular	Middle Class
Following	No Exercise	Alcoholic	Non Smoker	High Class
Following	Occasionally	Occasionally	Occasionally	Poor
Following	Occasionally	Occasionally	Non Smoker	Middle Class
Occasionally	Occasionally	Occasionally	Occasionally	Poor
Following	Occasionally	Alcoholic	Regular	Poor

Following	Regular Exercise	Non Alcoholic	Occasionally	Poor
Occasionally	Regular Exercise	Alcoholic	Occasionally	Poor
Occasionally	Regular Exercise	Non Alcoholic	Non Smoker	Poor
Occasionally	Regular Exercise	Alcoholic	Occasionally	High Class
Following	Regular Exercise	Occasionally	Regular	Middle Class
Following	Regular Exercise	Non Alcoholic	Non Smoker	High Class
Following	Occasionally	Alcoholic	Occasionally	Middle Class
Occasionally	Regular Exercise	Occasionally	Non Smoker	High Class
Not Following	No Exercise	Occasionally	Regular	Poor
Following	Occasionally	Occasionally	Occasionally	Middle Class
Not Following	Regular Exercise	Occasionally	Non Smoker	Poor
Not Following	Regular Exercise	Occasionally	Occasionally	Middle Class
Following	Regular Exercise	Non Alcoholic	Non Smoker	Poor
Following	Regular Exercise	Non Alcoholic	Occasionally	Poor
Following	Regular Exercise	Occasionally	Occasionally	Poor
Occasionally	Occasionally	Occasionally	Non Smoker	High Class
Following	Regular Exercise	Alcoholic	Regular	High Class
Occasionally	Regular Exercise	Occasionally	Occasionally	High Class
Not Following	Occasionally	Alcoholic	Regular	High Class
Following	Occasionally	Occasionally	Occasionally	High Class
Following	Occasionally	Non Alcoholic	Regular	Middle Class
Following	Regular Exercise	Alcoholic	Occasionally	Poor
Following	Regular Exercise	Non Alcoholic	Non Smoker	High Class
Not Following	Regular Exercise	Occasionally	Regular	High Class
Following	Occasionally	Occasionally	Regular	Middle Class
Following	Occasionally	Non Alcoholic	Occasionally	High Class
Following	Regular Exercise	Alcoholic	Regular	High Class
Occasionally	Occasionally	Occasionally	Occasionally	Poor
Not Following	Regular Exercise	Alcoholic	Regular	Poor
Occasionally	No Exercise	Occasionally	Regular	Middle Class
Following	Occasionally	Alcoholic	Regular	Middle Class
Not Following	Regular Exercise	Alcoholic	Regular	Middle Class
Occasionally	No Exercise	Non Alcoholic	Regular	Middle Class
Occasionally	Regular Exercise	Non Alcoholic	Regular	Middle Class
Occasionally	Regular Exercise	Non Alcoholic	Regular	High Class
Following	No Exercise	Alcoholic	Occasionally	High Class
Not Following	Occasionally	Alcoholic	Regular	Middle Class
Following	Regular Exercise	Non Alcoholic	Occasionally	Middle Class
Following	Regular Exercise	Non Alcoholic	Non Smoker	High Class
Following	Regular Exercise	Non Alcoholic	Non Smoker	Poor
Occasionally	Regular Exercise	Occasionally	Regular	Poor
Following	Occasionally	Occasionally	Regular	High Class
Following	Regular Exercise	Non Alcoholic	Non Smoker	High Class
Occasionally	Occasionally	Occasionally	Occasionally	Poor
Following	Regular Exercise	Non Alcoholic	Non Smoker	Middle Class
Not Following	No Exercise	Alcoholic	Non Smoker	Poor
Following	Regular Exercise	Alcoholic	Regular	Poor

Not Following	Regular Exercise	Occasionally	Regular	Middle Class
Not Following	Regular Exercise	Occasionally	Regular	Poor
Not Following	Occasionally	Occasionally	Occasionally	Middle Class
Following	Regular Exercise	Alcoholic	Regular	Middle Class
Following	No Exercise	Occasionally	Occasionally	Poor
Following	Regular Exercise	Occasionally	Regular	Middle Class
Following	Regular Exercise	Occasionally	Non Smoker	Poor
Following	Occasionally	Non Alcoholic	Non Smoker	Poor
Following	Regular Exercise	Occasionally	Occasionally	High Class
Following	Regular Exercise	Non Alcoholic	Regular	Poor
Occasionally	Occasionally	Alcoholic	Regular	High Class
Not Following	No Exercise	Non Alcoholic	Regular	High Class
Occasionally	Occasionally	Non Alcoholic	Non Smoker	High Class
Not Following	Regular Exercise	Occasionally	Occasionally	Middle Class
Following	Regular Exercise	Occasionally	Regular	Middle Class
Occasionally	Occasionally	Occasionally	Regular	Middle Class
Not Following	No Exercise	Non Alcoholic	Non Smoker	Middle Class
Occasionally	Regular Exercise	Non Alcoholic	Occasionally	Middle Class
Following	Regular Exercise	Non Alcoholic	Non Smoker	Poor
Occasionally	Occasionally	Occasionally	Occasionally	Poor
Occasionally	Occasionally	Occasionally	Occasionally	High Class
Following	No Exercise	Occasionally	Regular	Poor
Not Following	No Exercise	Alcoholic	Regular	Middle Class
Not Following	Regular Exercise	Occasionally	Regular	Middle Class
Following	Regular Exercise	Non Alcoholic	Regular	Poor
Not Following	No Exercise	Occasionally	Occasionally	Poor
Following	Regular Exercise	Non Alcoholic	Non Smoker	High Class
Following	Regular Exercise	Alcoholic	Non Smoker	Poor
Not Following	Occasionally	Alcoholic	Regular	Poor
Not Following	Regular Exercise	Occasionally	Non Smoker	Poor
Following	Regular Exercise	Occasionally	Non Smoker	Middle Class
Following	Regular Exercise	Non Alcoholic	Regular	High Class
Following	Occasionally	Occasionally	Non Smoker	Poor
Not Following	No Exercise	Alcoholic	Occasionally	High Class
Following	Occasionally	Alcoholic	Regular	Middle Class
Not Following	Regular Exercise	Alcoholic	Regular	Poor
Following	Regular Exercise	Non Alcoholic	Regular	High Class
Following	No Exercise	Non Alcoholic	Occasionally	Poor
Occasionally	No Exercise	Non Alcoholic	Non Smoker	High Class
Occasionally	Regular Exercise	Alcoholic	Non Smoker	Poor
Not Following	Occasionally	Occasionally	Regular	High Class
Not Following	Occasionally	Non Alcoholic	Regular	Poor
Occasionally	Regular Exercise	Non Alcoholic	Regular	Middle Class
Not Following	No Exercise	Alcoholic	Regular	Middle Class
Occasionally	Regular Exercise	Occasionally	Occasionally	Middle Class
Occasionally	Regular Exercise	Non Alcoholic	Non Smoker	Middle Class
Following	Regular Exercise	Non Alcoholic	Non Smoker	Middle Class

Occasionally	No Exercise	Occasionally	Non Smoker	Poor
Occasionally	Regular Exercise	Alcoholic	Regular	High Class
Following	Regular Exercise	Non Alcoholic	Occasionally	Middle Class
Following	Occasionally	Occasionally	Occasionally	High Class
Following	Regular Exercise	Non Alcoholic	Occasionally	Poor
Following	No Exercise	Alcoholic	Occasionally	High Class
Occasionally	No Exercise	Alcoholic	Occasionally	High Class
Occasionally	No Exercise	Alcoholic	Regular	High Class
Not Following	Occasionally	Non Alcoholic	Occasionally	Poor
Occasionally	Occasionally	Alcoholic	Regular	High Class
Not Following	No Exercise	Alcoholic	Regular	Poor
Following	Occasionally	Non Alcoholic	Regular	Poor
Not Following	Occasionally	Alcoholic	Regular	High Class
Not Following	Occasionally	Non Alcoholic	Regular	Poor
Not Following	No Exercise	Occasionally	Regular	Poor
Following	Occasionally	Non Alcoholic	Regular	Middle Class
Occasionally	No Exercise	Alcoholic	Non Smoker	High Class
Occasionally	Occasionally	Alcoholic	Regular	Middle Class
Occasionally	Occasionally	Occasionally	Occasionally	Poor
Occasionally	No Exercise	Occasionally	Regular	Poor
Not Following	Occasionally	Non Alcoholic	Regular	High Class
Following	Regular Exercise	Alcoholic	Occasionally	High Class
Following	Regular Exercise	Occasionally	Occasionally	High Class
Following	No Exercise	Alcoholic	Occasionally	High Class
Following	Occasionally	Non Alcoholic	Occasionally	High Class
Occasionally	Occasionally	Occasionally	Occasionally	High Class
Following	Occasionally	Occasionally	Regular	High Class
Not Following	Occasionally	Alcoholic	Regular	Poor
Following	Regular Exercise	Occasionally	Occasionally	Poor
Occasionally	No Exercise	Non Alcoholic	Non Smoker	Poor
Occasionally	Regular Exercise	Non Alcoholic	Regular	High Class
Occasionally	No Exercise	Alcoholic	Regular	High Class
Occasionally	Occasionally	Occasionally	Occasionally	Poor
Following	Occasionally	Occasionally	Regular	Poor
Not Following	Occasionally	Non Alcoholic	Regular	High Class
Following	Occasionally	Occasionally	Regular	Poor
Not Following	Occasionally	Occasionally	Occasionally	High Class
Not Following	Occasionally	Alcoholic	Occasionally	Poor
Occasionally	Occasionally	Non Alcoholic	Non Smoker	Poor
Occasionally	Regular Exercise	Occasionally	Non Smoker	High Class
Not Following	Occasionally	Occasionally	Occasionally	Middle Class
Following	Regular Exercise	Non Alcoholic	Non Smoker	Poor
Not Following	Regular Exercise	Alcoholic	Regular	High Class
Not Following	Regular Exercise	Occasionally	Occasionally	High Class
Occasionally	Occasionally	Occasionally	Non Smoker	Poor
Following	Occasionally	Non Alcoholic	Non Smoker	High Class
Not Following	Occasionally	Non Alcoholic	Regular	Poor

Following	Occasionally	Non Alcoholic	Non Smoker	Poor
Following	Occasionally	Non Alcoholic	Regular	Poor
Following	Occasionally	Alcoholic	Regular	Poor
Following	Regular Exercise	Alcoholic	Regular	Middle Class
Following	Regular Exercise	Non Alcoholic	Non Smoker	Middle Class
Following	Regular Exercise	Non Alcoholic	Occasionally	Poor
Following	Occasionally	Alcoholic	Regular	High Class
Following	Regular Exercise	Non Alcoholic	Non Smoker	High Class
Occasionally	Occasionally	Alcoholic	Occasionally	Middle Class
Occasionally	Occasionally	Non Alcoholic	Non Smoker	High Class
Following	Regular Exercise	Occasionally	Regular	Poor
Occasionally	Regular Exercise	Occasionally	Occasionally	Poor
Following	Occasionally	Occasionally	Non Smoker	Poor
Occasionally	Occasionally	Occasionally	Non Smoker	Poor
Following	Regular Exercise	Non Alcoholic	Regular	Poor
Following	No Exercise	Occasionally	Occasionally	High Class
Not Following	Occasionally	Non Alcoholic	Occasionally	Poor
Not Following	Regular Exercise	Alcoholic	Regular	Poor
Following	Regular Exercise	Occasionally	Regular	Middle Class
Not Following	No Exercise	Alcoholic	Regular	Middle Class
Following	Regular Exercise	Non Alcoholic	Non Smoker	High Class
Following	Regular Exercise	Alcoholic	Occasionally	Poor
Following	Regular Exercise	Non Alcoholic	Regular	Middle Class
Following	Regular Exercise	Non Alcoholic	Non Smoker	High Class
Not Following	No Exercise	Non Alcoholic	Occasionally	Poor
Following	Regular Exercise	Non Alcoholic	Occasionally	High Class
Following	No Exercise	Non Alcoholic	Regular	Middle Class
Following	No Exercise	Alcoholic	Regular	Middle Class
Occasionally	Occasionally	Occasionally	Occasionally	Middle Class
Occasionally	Occasionally	Non Alcoholic	Occasionally	High Class
Occasionally	Regular Exercise	Non Alcoholic	Occasionally	Poor
Not Following	No Exercise	Occasionally	Occasionally	High Class
Occasionally	Regular Exercise	Non Alcoholic	Non Smoker	High Class
Following	Regular Exercise	Non Alcoholic	Non Smoker	Middle Class
Following	Occasionally	Occasionally	Regular	High Class
Not Following	No Exercise	Non Alcoholic	Occasionally	High Class
Occasionally	No Exercise	Alcoholic	Non Smoker	High Class
Following	Regular Exercise	Non Alcoholic	Occasionally	Middle Class
Following	No Exercise	Occasionally	Regular	High Class
Following	Regular Exercise	Non Alcoholic	Occasionally	Poor
Following	No Exercise	Occasionally	Regular	Poor
Following	Regular Exercise	Non Alcoholic	Regular	Poor
Following	No Exercise	Non Alcoholic	Regular	High Class
Following	Regular Exercise	Non Alcoholic	Regular	Poor
Occasionally	Regular Exercise	Non Alcoholic	Occasionally	Poor
Occasionally	No Exercise	Non Alcoholic	Regular	High Class
Following	Regular Exercise	Non Alcoholic	Regular	High Class

Following	No Exercise	Non Alcoholic	Occasionally	Middle Class
Occasionally	Regular Exercise	Non Alcoholic	Regular	High Class
Occasionally	No Exercise	Non Alcoholic	Occasionally	High Class
Occasionally	Regular Exercise	Non Alcoholic	Regular	Poor
Occasionally	Regular Exercise	Non Alcoholic	Regular	High Class
Occasionally	No Exercise	Non Alcoholic	Occasionally	Middle Class
Occasionally	Regular Exercise	Non Alcoholic	Regular	High Class
Following	No Exercise	Non Alcoholic	Regular	High Class
Following	Regular Exercise	Non Alcoholic	Regular	Poor
Following	No Exercise	Non Alcoholic	Occasionally	High Class
Following	Regular Exercise	Non Alcoholic	Regular	High Class
Following	Regular Exercise	Non Alcoholic	Regular	High Class
Following	Regular Exercise	Non Alcoholic	Occasionally	Middle Class
Following	No Exercise	Non Alcoholic	Regular	Middle Class
Following	Regular Exercise	Non Alcoholic	Regular	Middle Class
Following	Regular Exercise	Non Alcoholic	Occasionally	Middle Class
Following	Regular Exercise	Occasionally	Regular	Middle Class
Following	No Exercise	Non Alcoholic	Occasionally	High Class
Following	Regular Exercise	Non Alcoholic	Regular	High Class
Following	Regular Exercise	Non Alcoholic	Regular	High Class
Following	No Exercise	Non Alcoholic	Occasionally	High Class
Following	Regular Exercise	Non Alcoholic	Regular	Poor
Following	Regular Exercise	Non Alcoholic	Regular	High Class
Following	No Exercise	Non Alcoholic	Regular	Middle Class
Following	Regular Exercise	Non Alcoholic	Occasionally	Poor
Following	No Exercise	Non Alcoholic	Regular	Poor
Following	Regular Exercise	Non Alcoholic	Regular	Poor
Following	Regular Exercise	Non Alcoholic	Regular	Poor
Following	Regular Exercise	Occasionally	Regular	Middle Class
Following	No Exercise	Non Alcoholic	Occasionally	Poor
Occasionally	Regular Exercise	Non Alcoholic	Regular	High Class
Occasionally	No Exercise	Non Alcoholic	Regular	High Class
Occasionally	Regular Exercise	Occasionally	Occasionally	Middle Class
Following	No Exercise	Non Alcoholic	Non Smoker	Middle Class
Following	Regular Exercise	Non Alcoholic	Occasionally	Middle Class
Following	Occasionally	Non Alcoholic	Regular	Middle Class
Occasionally	Regular Exercise	Non Alcoholic	Regular	Middle Class
Following	No Exercise	Occasionally	Occasionally	High Class
Occasionally	Regular Exercise	Non Alcoholic	Regular	Middle Class
Following	Regular Exercise	Non Alcoholic	Regular	Poor
Occasionally	No Exercise	Non Alcoholic	Regular	High Class
Following	Occasionally	Non Alcoholic	Occasionally	Poor
Following	Regular Exercise	Non Alcoholic	Non Smoker	Middle Class
Following	No Exercise	Alcoholic	Regular	High Class
Following	Regular Exercise	Occasionally	Regular	Middle Class
Following	No Exercise	Non Alcoholic	Regular	Poor
Following	Regular Exercise	Non Alcoholic	Occasionally	Poor

Following	No Exercise	Non Alcoholic	Regular	Middle Class
Following	Regular Exercise	Occasionally	Regular	Middle Class
Following	No Exercise	Non Alcoholic	Occasionally	Middle Class
Following	Regular Exercise	Non Alcoholic	Regular	High Class
Following	No Exercise	Non Alcoholic	Occasionally	High Class
Following	Occasionally	Non Alcoholic	Regular	High Class
Following	Regular Exercise	Non Alcoholic	Regular	Middle Class
Following	No Exercise	Non Alcoholic	Occasionally	Middle Class
Following	No Exercise	Non Alcoholic	Regular	Middle Class
Following	Regular Exercise	Occasionally	Regular	Middle Class
Following	Regular Exercise	Non Alcoholic	Regular	Poor
Following	No Exercise	Non Alcoholic	Non Smoker	Poor
Following	No Exercise	Non Alcoholic	Regular	Poor
Following	No Exercise	Non Alcoholic	Regular	High Class
Following	No Exercise	Non Alcoholic	Regular	High Class
Following	Regular Exercise	Non Alcoholic	Regular	High Class
Following	No Exercise	Non Alcoholic	Occasionally	High Class
Following	Regular Exercise	Non Alcoholic	Regular	Middle Class
Following	Occasionally	Occasionally	Regular	High Class
Following	No Exercise	Non Alcoholic	Occasionally	Middle Class
Following	No Exercise	Non Alcoholic	Non Smoker	High Class
Following	No Exercise	Non Alcoholic	Occasionally	Middle Class
Occasionally	Regular Exercise	Non Alcoholic	Regular	High Class
Following	No Exercise	Non Alcoholic	Regular	Poor
Following	Regular Exercise	Non Alcoholic	Occasionally	Poor
Following	No Exercise	Non Alcoholic	Regular	Middle Class
Following	Regular Exercise	Non Alcoholic	Regular	Middle Class
Following	No Exercise	Alcoholic	Regular	Middle Class
Following	Regular Exercise	Non Alcoholic	Occasionally	Middle Class
Following	No Exercise	Non Alcoholic	Regular	High Class
Following	Occasionally	Non Alcoholic	Regular	High Class
Occasionally	Regular Exercise	Non Alcoholic	Regular	Poor
Occasionally	Regular Exercise	Non Alcoholic	Regular	Poor
Following	Regular Exercise	Non Alcoholic	Occasionally	Middle Class
Occasionally	Regular Exercise	Non Alcoholic	Regular	Middle Class
Following	Regular Exercise	Non Alcoholic	Regular	Middle Class
Following	No Exercise	Non Alcoholic	Non Smoker	Middle Class
Following	Regular Exercise	Non Alcoholic	Regular	Middle Class
Following	Regular Exercise	Occasionally	Occasionally	High Class
Following	Regular Exercise	Non Alcoholic	Regular	Middle Class
Occasionally	Regular Exercise	Non Alcoholic	Regular	High Class
Occasionally	Regular Exercise	Non Alcoholic	Occasionally	High Class
Occasionally	Regular Exercise	Non Alcoholic	Regular	High Class
Occasionally	Regular Exercise	Non Alcoholic	Regular	High Class
Occasionally	Regular Exercise	Non Alcoholic	Regular	High Class
Following	No Exercise	Alcoholic	Non Smoker	High Class
Following	No Exercise	Non Alcoholic	Regular	Poor

Following	No Exercise	Alcoholic	Regular	Poor
Following	Regular Exercise	Non Alcoholic	Regular	Poor
Following	No Exercise	Non Alcoholic	Regular	Middle Class
Following	Regular Exercise	Non Alcoholic	Occasionally	High Class
Following	Regular Exercise	Non Alcoholic	Regular	Poor
Following	Regular Exercise	Non Alcoholic	Regular	Poor
Following	No Exercise	Non Alcoholic	Occasionally	Poor
Following	No Exercise	Non Alcoholic	Regular	Poor
Following	Regular Exercise	Alcoholic	Occasionally	Poor
Following	No Exercise	Non Alcoholic	Regular	Poor
Following	No Exercise	Non Alcoholic	Regular	Poor
Following	Regular Exercise	Non Alcoholic	Occasionally	Poor
Following	Regular Exercise	Non Alcoholic	Regular	Middle Class
Following	No Exercise	Non Alcoholic	Regular	High Class
Occasionally	Regular Exercise	Non Alcoholic	Non Smoker	Poor
Following	No Exercise	Non Alcoholic	Occasionally	Middle Class
Following	No Exercise	Non Alcoholic	Regular	Poor
Following	Regular Exercise	Non Alcoholic	Regular	Poor
Following	No Exercise	Non Alcoholic	Regular	Poor
Following	Regular Exercise	Non Alcoholic	Regular	High Class
Following	Regular Exercise	Occasionally	Occasionally	High Class
Following	No Exercise	Occasionally	Regular	High Class
Following	No Exercise	Non Alcoholic	Regular	Poor
Following	No Exercise	Non Alcoholic	Occasionally	Poor
Not Following	Regular Exercise	Non Alcoholic	Regular	Middle Class
Following	Regular Exercise	Non Alcoholic	Occasionally	Middle Class
Not Following	Regular Exercise	Non Alcoholic	Regular	High Class
Following	No Exercise	Non Alcoholic	Regular	Poor
Following	Regular Exercise	Non Alcoholic	Occasionally	High Class
Following	No Exercise	Occasionally	Regular	Poor
Not Following	Regular Exercise	Non Alcoholic	Regular	Middle Class
Not Following	No Exercise	Non Alcoholic	Regular	Middle Class
Not Following	Regular Exercise	Non Alcoholic	Occasionally	High Class
Following	Regular Exercise	Non Alcoholic	Regular	Poor
Not Following	Regular Exercise	Non Alcoholic	Regular	Poor
Not Following	Regular Exercise	Occasionally	Regular	Poor
Following	Regular Exercise	Non Alcoholic	Regular	High Class
Not Following	Regular Exercise	Non Alcoholic	Occasionally	High Class
Not Following	No Exercise	Non Alcoholic	Regular	High Class
Not Following	Regular Exercise	Non Alcoholic	Non Smoker	High Class
Occasionally	Regular Exercise	Non Alcoholic	Occasionally	High Class
Not Following	Regular Exercise	Occasionally	Regular	Poor
Following	Regular Exercise	Non Alcoholic	Non Smoker	Middle Class
Occasionally	Occasionally	Non Alcoholic	Regular	High Class
Occasionally	Regular Exercise	Non Alcoholic	Regular	High Class
Occasionally	Regular Exercise	Non Alcoholic	Occasionally	High Class
Occasionally	Occasionally	Occasionally	Regular	High Class

Following	No Exercise	Non Alcoholic	Regular	High Class
Following	No Exercise	Non Alcoholic	Regular	High Class
Occasionally	Occasionally	Non Alcoholic	Occasionally	High Class
Occasionally	Regular Exercise	Non Alcoholic	Regular	High Class
Not Following	No Exercise	Non Alcoholic	Regular	Middle Class
Not Following	Regular Exercise	Non Alcoholic	Regular	Middle Class
Following	Regular Exercise	Non Alcoholic	Regular	Middle Class
Following	Regular Exercise	Non Alcoholic	Occasionally	Poor
Not Following	No Exercise	Non Alcoholic	Regular	Poor
Following	Regular Exercise	Occasionally	Regular	High Class
Following	No Exercise	Non Alcoholic	Occasionally	Middle Class
Following	Regular Exercise	Occasionally	Regular	High Class
Not Following	No Exercise	Non Alcoholic	Occasionally	Poor
Not Following	Regular Exercise	Non Alcoholic	Regular	Middle Class
Not Following	Regular Exercise	Non Alcoholic	Regular	High Class
Not Following	No Exercise	Non Alcoholic	Occasionally	Poor
Following	Occasionally	Non Alcoholic	Regular	Middle Class
Not Following	Regular Exercise	Non Alcoholic	Regular	High Class
Following	No Exercise	Occasionally	Regular	Middle Class
Not Following	Regular Exercise	Non Alcoholic	Occasionally	Poor
Following	No Exercise	Non Alcoholic	Non Smoker	High Class
Following	Regular Exercise	Non Alcoholic	Regular	Middle Class
Following	No Exercise	Occasionally	Regular	Poor
Following	Regular Exercise	Non Alcoholic	Regular	High Class
Following	No Exercise	Non Alcoholic	Occasionally	Middle Class
Following	Regular Exercise	Non Alcoholic	Regular	High Class
Not Following	No Exercise	Non Alcoholic	Regular	Middle Class
Following	No Exercise	Non Alcoholic	Occasionally	Poor
Following	Regular Exercise	Non Alcoholic	Regular	Poor
Not Following	Occasionally	Non Alcoholic	Occasionally	Poor
Not Following	No Exercise	Occasionally	Regular	Poor
Occasionally	Regular Exercise	Non Alcoholic	Regular	High Class
Occasionally	Regular Exercise	Non Alcoholic	Occasionally	High Class
Occasionally	No Exercise	Non Alcoholic	Regular	Poor
Not Following	No Exercise	Non Alcoholic	Regular	High Class
Occasionally	No Exercise	Non Alcoholic	Regular	High Class
Occasionally	No Exercise	Non Alcoholic	Occasionally	Middle Class
Occasionally	Regular Exercise	Non Alcoholic	Regular	High Class
Not Following	No Exercise	Non Alcoholic	Regular	Middle Class
Not Following	Regular Exercise	Occasionally	Regular	High Class
Not Following	Occasionally	Non Alcoholic	Regular	Poor
Following	No Exercise	Non Alcoholic	Occasionally	Poor
Following	No Exercise	Non Alcoholic	Regular	Poor
Following	No Exercise	Occasionally	Regular	Poor
Following	Regular Exercise	Non Alcoholic	Occasionally	Poor
Not Following	No Exercise	Non Alcoholic	Non Smoker	High Class
Not Following	Regular Exercise	Non Alcoholic	Occasionally	High Class

Following	No Exercise	Non Alcoholic	Regular	Poor
Following	Regular Exercise	Non Alcoholic	Regular	Poor
Not Following	No Exercise	Non Alcoholic	Occasionally	High Class
Occasionally	Regular Exercise	Non Alcoholic	Regular	Poor
Not Following	No Exercise	Non Alcoholic	Regular	Middle Class
Not Following	Occasionally	Occasionally	Regular	Middle Class
Not Following	Regular Exercise	Non Alcoholic	Occasionally	Middle Class
Occasionally	Occasionally	Non Alcoholic	Regular	Poor
Occasionally	Regular Exercise	Non Alcoholic	Regular	Poor
Not Following	Regular Exercise	Non Alcoholic	Regular	High Class
Not Following	Occasionally	Non Alcoholic	Regular	Middle Class
Not Following	No Exercise	Non Alcoholic	Occasionally	Poor
Not Following	Regular Exercise	Non Alcoholic	Regular	Poor
Not Following	Regular Exercise	Non Alcoholic	Regular	Middle Class
Occasionally	Regular Exercise	Occasionally	Occasionally	Middle Class
Not Following	No Exercise	Non Alcoholic	Regular	High Class
Occasionally	Regular Exercise	Non Alcoholic	Occasionally	High Class
Following	Regular Exercise	Non Alcoholic	Regular	High Class
Following	Regular Exercise	Non Alcoholic	Regular	Poor
Not Following	Regular Exercise	Non Alcoholic	Occasionally	Poor
Not Following	No Exercise	Occasionally	Non Smoker	High Class
Following	Occasionally	Non Alcoholic	Regular	Middle Class
Occasionally	No Exercise	Non Alcoholic	Regular	High Class
Following	Regular Exercise	Non Alcoholic	Non Smoker	Middle Class
Occasionally	No Exercise	Occasionally	Regular	High Class
Following	Regular Exercise	Non Alcoholic	Regular	High Class
Following	No Exercise	Non Alcoholic	Regular	High Class
Occasionally	Regular Exercise	Non Alcoholic	Regular	High Class
Occasionally	No Exercise	Non Alcoholic	Occasionally	High Class
Following	Occasionally	Non Alcoholic	Regular	Poor
Occasionally	Regular Exercise	Occasionally	Regular	High Class
Occasionally	No Exercise	Non Alcoholic	Occasionally	High Class
Following	No Exercise	Non Alcoholic	Regular	High Class
Occasionally	Occasionally	Non Alcoholic	Occasionally	High Class
Not Following	Regular Exercise	Non Alcoholic	Regular	High Class
Not Following	No Exercise	Occasionally	Regular	Middle Class
Following	No Exercise	Occasionally	Occasionally	High Class
Following	No Exercise	Non Alcoholic	Regular	Middle Class
Not Following	No Exercise	Non Alcoholic	Regular	Poor
Following	Regular Exercise	Non Alcoholic	Regular	High Class
Following	No Exercise	Occasionally	Occasionally	Middle Class
Not Following	Regular Exercise	Occasionally	Regular	Poor
Following	Regular Exercise	Non Alcoholic	Regular	Poor
Following	Regular Exercise	Non Alcoholic	Regular	Poor
Not Following	No Exercise	Alcoholic	Regular	Poor
Following	Occasionally	Non Alcoholic	Occasionally	Poor
Not Following	Regular Exercise	Non Alcoholic	Regular	High Class

Following	No Exercise	Non Alcoholic	Regular	Poor
Not Following	Regular Exercise	Occasionally	Occasionally	High Class
Following	Occasionally	Non Alcoholic	Regular	Poor
Following	Regular Exercise	Non Alcoholic	Occasionally	Poor
Not Following	No Exercise	Non Alcoholic	Regular	High Class
Following	Regular Exercise	Non Alcoholic	Regular	Middle Class
Not Following	No Exercise	Non Alcoholic	Occasionally	Middle Class
Not Following	No Exercise	Alcoholic	Regular	Middle Class
Following	Regular Exercise	Non Alcoholic	Regular	Poor
Not Following	Regular Exercise	Non Alcoholic	Regular	High Class
Following	Regular Exercise	Non Alcoholic	Occasionally	Middle Class
Not Following	Regular Exercise	Non Alcoholic	Regular	Poor
Not Following	Regular Exercise	Occasionally	Regular	Poor
Following	No Exercise	Non Alcoholic	Regular	High Class
Following	Regular Exercise	Non Alcoholic	Regular	Poor
Following	Regular Exercise	Non Alcoholic	Occasionally	Poor
Not Following	Regular Exercise	Non Alcoholic	Regular	Middle Class
Following	No Exercise	Occasionally	Regular	Poor
Following	Regular Exercise	Non Alcoholic	Occasionally	Poor
Not Following	Regular Exercise	Non Alcoholic	Regular	High Class
Not Following	Regular Exercise	Occasionally	Occasionally	High Class
Following	Regular Exercise	Non Alcoholic	Regular	Poor
Following	No Exercise	Non Alcoholic	Regular	Poor
Not Following	No Exercise	Alcoholic	Occasionally	Poor
Not Following	No Exercise	Non Alcoholic	Regular	Poor
Following	Regular Exercise	Non Alcoholic	Regular	Poor
Not Following	No Exercise	Non Alcoholic	Non Smoker	High Class
Not Following	No Exercise	Occasionally	Occasionally	High Class
Not Following	No Exercise	Non Alcoholic	Regular	High Class
Not Following	Regular Exercise	Non Alcoholic	Regular	High Class
Not Following	No Exercise	Non Alcoholic	Regular	High Class
Following	Regular Exercise	Non Alcoholic	Regular	High Class
Not Following	No Exercise	Occasionally	Occasionally	Middle Class
Following	Regular Exercise	Non Alcoholic	Regular	Middle Class
Not Following	Regular Exercise	Non Alcoholic	Regular	Middle Class
Not Following	Regular Exercise	Non Alcoholic	Occasionally	Middle Class
Not Following	Regular Exercise	Non Alcoholic	Regular	High Class
Following	No Exercise	Non Alcoholic	Occasionally	High Class
Not Following	Regular Exercise	Non Alcoholic	Regular	High Class
Following	No Exercise	Alcoholic	Regular	High Class
Not Following	No Exercise	Non Alcoholic	Occasionally	High Class
Following	Regular Exercise	Non Alcoholic	Regular	Middle Class
Following	Occasionally	Occasionally	Regular	Poor
Following	Occasionally	Non Alcoholic	Regular	Middle Class
Following	No Exercise	Non Alcoholic	Occasionally	Middle Class
Not Following	Occasionally	Non Alcoholic	Regular	High Class
Following	No Exercise	Non Alcoholic	Regular	Poor

Following	No Exercise	Non Alcoholic	Regular	Middle Class
Following	No Exercise	Non Alcoholic	Non Smoker	High Class
Following	Regular Exercise	Non Alcoholic	Occasionally	Middle Class
Occasionally	Regular Exercise	Non Alcoholic	Regular	High Class
Occasionally	No Exercise	Occasionally	Non Smoker	Middle Class
Occasionally	Regular Exercise	Occasionally	Occasionally	High Class
Occasionally	Regular Exercise	Non Alcoholic	Regular	Middle Class
Occasionally	Occasionally	Non Alcoholic	Occasionally	High Class
Following	Regular Exercise	Non Alcoholic	Regular	Poor
Occasionally	No Exercise	Non Alcoholic	Regular	Poor
Following	No Exercise	Non Alcoholic	Occasionally	Poor
Not Following	No Exercise	Non Alcoholic	Regular	Poor
Following	Regular Exercise	Non Alcoholic	Regular	Middle Class
Not Following	Regular Exercise	Non Alcoholic	Regular	Middle Class
Following	No Exercise	Occasionally	Occasionally	High Class
Not Following	Regular Exercise	Non Alcoholic	Regular	High Class
Following	Occasionally	Non Alcoholic	Regular	High Class
Following	Regular Exercise	Non Alcoholic	Regular	Middle Class
Occasionally	No Exercise	Non Alcoholic	Regular	Middle Class
Occasionally	Regular Exercise	Non Alcoholic	Occasionally	Middle Class
Not Following	Occasionally	Non Alcoholic	Regular	High Class
Occasionally	Regular Exercise	Non Alcoholic	Regular	High Class
Not Following	No Exercise	Non Alcoholic	Occasionally	Poor
Not Following	Occasionally	Non Alcoholic	Regular	Poor
Not Following	No Exercise	Non Alcoholic	Occasionally	Poor
Occasionally	Occasionally	Occasionally	Regular	High Class
Not Following	No Exercise	Occasionally	Regular	High Class
Not Following	Occasionally	Non Alcoholic	Occasionally	High Class
Not Following	No Exercise	Non Alcoholic	Regular	Middle Class
Occasionally	No Exercise	Non Alcoholic	Regular	Middle Class
Occasionally	Regular Exercise	Non Alcoholic	Regular	Middle Class
Occasionally	No Exercise	Occasionally	Occasionally	Middle Class
Not Following	No Exercise	Non Alcoholic	Regular	Poor
Not Following	Regular Exercise	Non Alcoholic	Regular	Poor
Following	No Exercise	Non Alcoholic	Regular	High Class
Following	No Exercise	Non Alcoholic	Regular	High Class
Not Following	No Exercise	Non Alcoholic	Occasionally	High Class
Following	No Exercise	Non Alcoholic	Regular	Middle Class
Not Following	No Exercise	Occasionally	Regular	Middle Class
Not Following	Regular Exercise	Non Alcoholic	Occasionally	Middle Class
Not Following	Regular Exercise	Non Alcoholic	Regular	Middle Class
Following	Regular Exercise	Occasionally	Occasionally	Middle Class
Occasionally	Occasionally	Non Alcoholic	Occasionally	Poor
Occasionally	No Exercise	Occasionally	Non Smoker	Poor
Following	Regular Exercise	Non Alcoholic	Occasionally	Poor
Occasionally	Regular Exercise	Occasionally	Occasionally	Middle Class
Occasionally	No Exercise	Occasionally	Regular	Middle Class

Following	No Exercise	Non Alcoholic	Regular	High Class
Following	Regular Exercise	Non Alcoholic	Regular	Poor
Occasionally	No Exercise	Non Alcoholic	Regular	Poor
Not Following	Regular Exercise	Occasionally	Occasionally	Poor
Not Following	No Exercise	Occasionally	Regular	High Class
Following	Regular Exercise	Alcoholic	Regular	Middle Class
Not Following	No Exercise	Occasionally	Occasionally	Middle Class
Following	No Exercise	Occasionally	Non Smoker	High Class
Not Following	Occasionally	Alcoholic	Regular	Poor
Not Following	No Exercise	Alcoholic	Non Smoker	High Class
Occasionally	Regular Exercise	Non Alcoholic	Non Smoker	Poor
Following	Regular Exercise	Non Alcoholic	Non Smoker	Poor
Occasionally	Regular Exercise	Occasionally	Regular	Poor
Following	No Exercise	Alcoholic	Occasionally	High Class
Not Following	Regular Exercise	Alcoholic	Regular	Poor
Following	Occasionally	Non Alcoholic	Regular	Poor
Occasionally	No Exercise	Occasionally	Occasionally	Middle Class
Not Following	No Exercise	Occasionally	Occasionally	High Class
Not Following	No Exercise	Non Alcoholic	Non Smoker	Poor
Not Following	No Exercise	Alcoholic	Regular	High Class
Occasionally	Occasionally	Occasionally	Occasionally	High Class
Not Following	No Exercise	Non Alcoholic	Non Smoker	High Class
Following	No Exercise	Non Alcoholic	Non Smoker	Poor
Not Following	No Exercise	Non Alcoholic	Regular	Poor
Not Following	Occasionally	Occasionally	Occasionally	Middle Class
Following	Regular Exercise	Non Alcoholic	Non Smoker	High Class
Not Following	Occasionally	Alcoholic	Regular	High Class
Not Following	No Exercise	Occasionally	Regular	Middle Class
Not Following	Regular Exercise	Occasionally	Regular	Middle Class
Following	No Exercise	Non Alcoholic	Occasionally	High Class
Following	No Exercise	Alcoholic	Regular	Poor
Following	No Exercise	Alcoholic	Occasionally	Poor
Following	Regular Exercise	Non Alcoholic	Occasionally	High Class
Occasionally	Occasionally	Non Alcoholic	Occasionally	High Class
Occasionally	No Exercise	Occasionally	Non Smoker	Poor
Not Following	Occasionally	Occasionally	Occasionally	High Class
Not Following	No Exercise	Alcoholic	Regular	High Class
Occasionally	No Exercise	Non Alcoholic	Regular	High Class
Not Following	No Exercise	Non Alcoholic	Non Smoker	Middle Class
Not Following	No Exercise	Occasionally	Occasionally	Poor
Occasionally	No Exercise	Non Alcoholic	Occasionally	Poor
Occasionally	Occasionally	Non Alcoholic	Non Smoker	Middle Class
Following	Regular Exercise	Non Alcoholic	Regular	Middle Class
Following	No Exercise	Non Alcoholic	Non Smoker	Middle Class
Occasionally	Occasionally	Non Alcoholic	Non Smoker	High Class
Not Following	No Exercise	Non Alcoholic	Non Smoker	Middle Class
Following	No Exercise	Alcoholic	Non Smoker	Middle Class

Not Following	No Exercise	Non Alcoholic	Non Smoker	High Class
Following	No Exercise	Non Alcoholic	Non Smoker	Poor
Not Following	No Exercise	Alcoholic	Regular	Poor
Following	No Exercise	Non Alcoholic	Occasionally	Poor
Not Following	No Exercise	Occasionally	Occasionally	Poor
Not Following	No Exercise	Non Alcoholic	Regular	Middle Class
Following	Regular Exercise	Alcoholic	Regular	Poor
Not Following	Regular Exercise	Non Alcoholic	Occasionally	High Class
Following	Occasionally	Non Alcoholic	Non Smoker	High Class
Occasionally	Occasionally	Occasionally	Occasionally	Middle Class
Following	No Exercise	Alcoholic	Regular	Poor
Occasionally	No Exercise	Occasionally	Regular	High Class
Not Following	No Exercise	Non Alcoholic	Regular	Poor
Not Following	Regular Exercise	Occasionally	Occasionally	Middle Class
Following	Regular Exercise	Non Alcoholic	Non Smoker	High Class
Not Following	Regular Exercise	Alcoholic	Non Smoker	Poor
Following	Regular Exercise	Alcoholic	Regular	High Class
Following	Occasionally	Non Alcoholic	Non Smoker	Poor
Following	Occasionally	Occasionally	Occasionally	Middle Class
Following	Regular Exercise	Non Alcoholic	Non Smoker	Poor
Occasionally	Regular Exercise	Non Alcoholic	Regular	High Class
Occasionally	Regular Exercise	Alcoholic	Regular	High Class
Occasionally	No Exercise	Occasionally	Occasionally	High Class
Occasionally	Regular Exercise	Alcoholic	Non Smoker	Poor
Occasionally	Occasionally	Non Alcoholic	Non Smoker	Poor
Occasionally	No Exercise	Non Alcoholic	Regular	Poor
Occasionally	Regular Exercise	Alcoholic	Occasionally	Poor
Occasionally	Occasionally	Non Alcoholic	Non Smoker	Middle Class
Occasionally	Regular Exercise	Non Alcoholic	Regular	High Class
Occasionally	Occasionally	Alcoholic	Occasionally	Poor
Occasionally	Regular Exercise	Occasionally	Occasionally	Poor
Occasionally	Regular Exercise	Alcoholic	Non Smoker	High Class
Occasionally	Regular Exercise	Alcoholic	Regular	Poor
Occasionally	Regular Exercise	Occasionally	Non Smoker	Poor
Occasionally	No Exercise	Alcoholic	Occasionally	Poor
Occasionally	No Exercise	Alcoholic	Occasionally	High Class
Occasionally	Regular Exercise	Occasionally	Non Smoker	Poor
Not Following	No Exercise	Non Alcoholic	Regular	Middle Class
Occasionally	Regular Exercise	Non Alcoholic	Regular	High Class
Occasionally	Occasionally	Alcoholic	Regular	Middle Class
Occasionally	Occasionally	Alcoholic	Non Smoker	Middle Class
Occasionally	No Exercise	Occasionally	Regular	Middle Class
Following	No Exercise	Alcoholic	Regular	Middle Class
Following	Occasionally	Non Alcoholic	Regular	Poor
Not Following	Occasionally	Alcoholic	Non Smoker	High Class
Following	Regular Exercise	Alcoholic	Non Smoker	High Class
Not Following	Regular Exercise	Non Alcoholic	Regular	High Class

Not Following	Occasionally	Alcoholic	Non Smoker	Middle Class
Occasionally	No Exercise	Alcoholic	Regular	Middle Class
Not Following	No Exercise	Alcoholic	Regular	Poor
Following	No Exercise	Non Alcoholic	Regular	High Class
Following	Regular Exercise	Occasionally	Regular	High Class
Not Following	Occasionally	Occasionally	Non Smoker	Middle Class
Occasionally	Occasionally	Non Alcoholic	Occasionally	Middle Class
Following	No Exercise	Occasionally	Occasionally	Middle Class
Occasionally	No Exercise	Alcoholic	Occasionally	High Class
Following	No Exercise	Non Alcoholic	Occasionally	High Class
Occasionally	Regular Exercise	Non Alcoholic	Regular	High Class
Occasionally	No Exercise	Alcoholic	Occasionally	Poor
Occasionally	Occasionally	Non Alcoholic	Regular	Poor
Not Following	Occasionally	Non Alcoholic	Regular	Middle Class
Following	Regular Exercise	Occasionally	Regular	Poor
Not Following	No Exercise	Non Alcoholic	Occasionally	High Class
Following	No Exercise	Non Alcoholic	Regular	Poor
Following	Regular Exercise	Non Alcoholic	Regular	High Class
Following	No Exercise	Non Alcoholic	Regular	Poor
Following	No Exercise	Occasionally	Regular	Middle Class
Not Following	No Exercise	Non Alcoholic	Occasionally	Middle Class
Following	Regular Exercise	Non Alcoholic	Regular	High Class
Not Following	Occasionally	Non Alcoholic	Occasionally	Middle Class
Following	Regular Exercise	Non Alcoholic	Regular	Poor
Following	No Exercise	Non Alcoholic	Occasionally	Poor
Following	Regular Exercise	Non Alcoholic	Regular	High Class
Occasionally	Regular Exercise	Alcoholic	Regular	Middle Class
Occasionally	Occasionally	Non Alcoholic	Regular	High Class
Occasionally	Regular Exercise	Non Alcoholic	Occasionally	Middle Class
Occasionally	Regular Exercise	Non Alcoholic	Regular	Middle Class
Not Following	No Exercise	Occasionally	Regular	High Class
Occasionally	No Exercise	Non Alcoholic	Regular	Poor
Not Following	No Exercise	Non Alcoholic	Regular	Middle Class
Occasionally	No Exercise	Non Alcoholic	Regular	Middle Class
Occasionally	No Exercise	Non Alcoholic	Regular	High Class
Occasionally	No Exercise	Occasionally	Occasionally	Poor
Following	Regular Exercise	Alcoholic	Regular	Middle Class
Following	Regular Exercise	Alcoholic	Regular	Middle Class
Following	Regular Exercise	Non Alcoholic	Non Smoker	Middle Class
Following	No Exercise	Non Alcoholic	Regular	High Class
Following	Regular Exercise	Occasionally	Occasionally	High Class
Following	Occasionally	Non Alcoholic	Regular	High Class
Following	No Exercise	Non Alcoholic	Regular	High Class
Following	No Exercise	Non Alcoholic	Occasionally	Poor
Following	Occasionally	Non Alcoholic	Regular	Poor
Following	No Exercise	Non Alcoholic	Regular	High Class
Following	No Exercise	Non Alcoholic	Regular	Poor

Following	No Exercise	Non Alcoholic	Regular	Poor
Following	No Exercise	Non Alcoholic	Regular	Poor
Following	Regular Exercise	Non Alcoholic	Regular	Poor
Occasionally	Regular Exercise	Alcoholic	Regular	Poor
Occasionally	Regular Exercise	Occasionally	Occasionally	Poor
Not Following	No Exercise	Non Alcoholic	Regular	Middle Class
Occasionally	No Exercise	Non Alcoholic	Non Smoker	High Class
Not Following	Regular Exercise	Non Alcoholic	Occasionally	Poor
Not Following	Regular Exercise	Alcoholic	Regular	Middle Class
Following	Regular Exercise	Non Alcoholic	Occasionally	Poor
Following	No Exercise	Non Alcoholic	Regular	Poor
Not Following	Regular Exercise	Occasionally	Regular	High Class
Following	Regular Exercise	Non Alcoholic	Regular	Poor
Following	Occasionally	Occasionally	Regular	Poor
Following	Regular Exercise	Non Alcoholic	Regular	Middle Class
Following	Regular Exercise	Non Alcoholic	Occasionally	Middle Class
Following	No Exercise	Non Alcoholic	Regular	High Class
Following	Regular Exercise	Non Alcoholic	Occasionally	High Class
Following	No Exercise	Occasionally	Regular	High Class
Following	No Exercise	Non Alcoholic	Regular	High Class
Occasionally	No Exercise	Occasionally	Regular	Middle Class
Occasionally	Regular Exercise	Non Alcoholic	Regular	Poor
Occasionally	No Exercise	Non Alcoholic	Regular	High Class
Occasionally	No Exercise	Non Alcoholic	Occasionally	High Class
Occasionally	Regular Exercise	Non Alcoholic	Regular	High Class
Occasionally	Regular Exercise	Non Alcoholic	Regular	High Class
Occasionally	No Exercise	Non Alcoholic	Regular	Poor
Occasionally	Occasionally	Non Alcoholic	Occasionally	High Class
Following	No Exercise	Non Alcoholic	Regular	Poor
Following	Regular Exercise	Non Alcoholic	Regular	High Class
Following	Regular Exercise	Non Alcoholic	Regular	High Class
Following	No Exercise	Non Alcoholic	Regular	Poor
Following	Occasionally	Non Alcoholic	Occasionally	Middle Class
Not Following	Regular Exercise	Alcoholic	Regular	High Class
Following	Regular Exercise	Non Alcoholic	Occasionally	High Class
Following	Regular Exercise	Non Alcoholic	Occasionally	Middle Class
Following	Regular Exercise	Non Alcoholic	Regular	High Class
Following	No Exercise	Non Alcoholic	Occasionally	Poor
Following	Regular Exercise	Non Alcoholic	Regular	Poor
Not Following	Regular Exercise	Occasionally	Regular	Poor
Not Following	Regular Exercise	Non Alcoholic	Occasionally	Poor
Not Following	Regular Exercise	Non Alcoholic	Regular	High Class
Following	No Exercise	Non Alcoholic	Regular	High Class
Not Following	No Exercise	Non Alcoholic	Occasionally	Middle Class
Not Following	Regular Exercise	Non Alcoholic	Regular	High Class
Not Following	No Exercise	Non Alcoholic	Occasionally	High Class
Following	No Exercise	Non Alcoholic	Regular	Poor

Not Following	Regular Exercise	Non Alcoholic	Regular	High Class
Following	No Exercise	Non Alcoholic	Occasionally	Middle Class
Following	No Exercise	Non Alcoholic	Regular	High Class
Following	Regular Exercise	Non Alcoholic	Regular	High Class
Not Following	Regular Exercise	Non Alcoholic	Regular	Poor
Following	Regular Exercise	Non Alcoholic	Occasionally	High Class
Not Following	Regular Exercise	Non Alcoholic	Regular	High Class
Following	Regular Exercise	Non Alcoholic	Regular	High Class
Following	No Exercise	Non Alcoholic	Non Smoker	High Class
Not Following	Occasionally	Non Alcoholic	Regular	High Class
Following	No Exercise	Non Alcoholic	Occasionally	Middle Class
Not Following	Occasionally	Non Alcoholic	Regular	Middle Class
Occasionally	No Exercise	Non Alcoholic	Non Smoker	Middle Class
Not Following	Regular Exercise	Non Alcoholic	Occasionally	Middle Class
Not Following	No Exercise	Non Alcoholic	Regular	Middle Class
Not Following	Regular Exercise	Non Alcoholic	Occasionally	Middle Class
Occasionally	No Exercise	Alcoholic	Regular	Poor
Following	Regular Exercise	Non Alcoholic	Regular	Poor
Not Following	No Exercise	Non Alcoholic	Occasionally	Poor
Not Following	Occasionally	Non Alcoholic	Regular	Poor
Not Following	No Exercise	Non Alcoholic	Regular	High Class
Not Following	No Exercise	Non Alcoholic	Regular	High Class
Not Following	Regular Exercise	Non Alcoholic	Occasionally	High Class
Following	Regular Exercise	Non Alcoholic	Regular	Poor
Following	Occasionally	Alcoholic	Regular	High Class
Following	No Exercise	Non Alcoholic	Regular	Poor
Following	No Exercise	Non Alcoholic	Regular	Middle Class
Not Following	Regular Exercise	Non Alcoholic	Occasionally	Middle Class
Following	No Exercise	Occasionally	Regular	Middle Class
Following	Regular Exercise	Non Alcoholic	Regular	Middle Class
Not Following	No Exercise	Non Alcoholic	Occasionally	Middle Class
Following	Regular Exercise	Non Alcoholic	Regular	Middle Class
Not Following	Regular Exercise	Non Alcoholic	Occasionally	High Class
Occasionally	Regular Exercise	Non Alcoholic	Regular	High Class
Occasionally	Regular Exercise	Non Alcoholic	Regular	High Class
Occasionally	Regular Exercise	Non Alcoholic	Occasionally	High Class
Occasionally	No Exercise	Non Alcoholic	Regular	Poor
Occasionally	No Exercise	Non Alcoholic	Regular	High Class
Occasionally	No Exercise	Non Alcoholic	Regular	Middle Class
Occasionally	Regular Exercise	Occasionally	Occasionally	Poor
Occasionally	No Exercise	Non Alcoholic	Regular	Poor
Occasionally	Regular Exercise	Non Alcoholic	Regular	Poor
Occasionally	No Exercise	Non Alcoholic	Regular	Poor
Occasionally	Regular Exercise	Non Alcoholic	Regular	Middle Class
Occasionally	No Exercise	Non Alcoholic	Occasionally	Poor
Occasionally	No Exercise	Non Alcoholic	Regular	High Class
Following	Regular Exercise	Non Alcoholic	Regular	High Class

Following	Regular Exercise	Non Alcoholic	Occasionally	Middle Class
Occasionally	Regular Exercise	Non Alcoholic	Regular	Middle Class
Following	No Exercise	Non Alcoholic	Occasionally	Middle Class
Occasionally	Regular Exercise	Non Alcoholic	Regular	Middle Class
Occasionally	No Exercise	Occasionally	Regular	Middle Class
Occasionally	Regular Exercise	Non Alcoholic	Occasionally	High Class
Not Following	Regular Exercise	Non Alcoholic	Regular	Middle Class
Not Following	No Exercise	Non Alcoholic	Regular	Poor
Following	Regular Exercise	Non Alcoholic	Regular	High Class
Not Following	Regular Exercise	Non Alcoholic	Occasionally	Poor
Following	No Exercise	Non Alcoholic	Regular	Middle Class
Following	Regular Exercise	Non Alcoholic	Non Smoker	High Class
Not Following	No Exercise	Non Alcoholic	Regular	Middle Class
Following	Regular Exercise	Non Alcoholic	Regular	Poor
Not Following	Regular Exercise	Non Alcoholic	Occasionally	Poor
Not Following	Regular Exercise	Non Alcoholic	Regular	Middle Class
Not Following	Regular Exercise	Alcoholic	Regular	Middle Class
Following	No Exercise	Non Alcoholic	Occasionally	Middle Class
Not Following	Regular Exercise	Non Alcoholic	Regular	High Class
Following	No Exercise	Non Alcoholic	Occasionally	High Class
Following	Regular Exercise	Non Alcoholic	Regular	High Class
Not Following	Regular Exercise	Non Alcoholic	Regular	Middle Class
Following	No Exercise	Non Alcoholic	Occasionally	Middle Class
Following	Regular Exercise	Non Alcoholic	Regular	Middle Class
Following	No Exercise	Non Alcoholic	Regular	Middle Class
Not Following	Regular Exercise	Non Alcoholic	Regular	Poor
Occasionally	Regular Exercise	Non Alcoholic	Occasionally	Poor
Occasionally	Occasionally	Non Alcoholic	Regular	Poor
Following	No Exercise	Non Alcoholic	Regular	High Class
Following	Occasionally	Non Alcoholic	Regular	High Class
Occasionally	No Exercise	Alcoholic	Regular	High Class
Occasionally	Regular Exercise	Non Alcoholic	Occasionally	High Class
Following	Regular Exercise	Non Alcoholic	Regular	Middle Class
Not Following	Regular Exercise	Non Alcoholic	Regular	High Class
Not Following	No Exercise	Non Alcoholic	Occasionally	Middle Class
Not Following	No Exercise	Non Alcoholic	Regular	High Class
Not Following	No Exercise	Non Alcoholic	Occasionally	Middle Class
Following	No Exercise	Non Alcoholic	Regular	High Class
Not Following	Regular Exercise	Non Alcoholic	Regular	Poor
Following	Regular Exercise	Non Alcoholic	Occasionally	Poor
Following	Regular Exercise	Non Alcoholic	Non Smoker	Middle Class
Following	No Exercise	Non Alcoholic	Regular	Middle Class
Following	No Exercise	Non Alcoholic	Regular	Middle Class
Occasionally	Regular Exercise	Occasionally	Occasionally	Middle Class
Following	Regular Exercise	Occasionally	Regular	High Class
Following	Occasionally	Alcoholic	Regular	High Class
Occasionally	Regular Exercise	Non Alcoholic	Regular	High Class

Occasionally	No Exercise	Non Alcoholic	Regular	High Class
Following	Regular Exercise	Non Alcoholic	Occasionally	High Class
Not Following	Regular Exercise	Non Alcoholic	Regular	Middle Class
Not Following	No Exercise	Non Alcoholic	Regular	Middle Class
Following	Regular Exercise	Non Alcoholic	Occasionally	Poor
Not Following	No Exercise	Non Alcoholic	Regular	Middle Class
Not Following	Regular Exercise	Non Alcoholic	Occasionally	Poor
Not Following	No Exercise	Occasionally	Regular	High Class
Not Following	Regular Exercise	Non Alcoholic	Regular	High Class
Following	Regular Exercise	Non Alcoholic	Occasionally	Poor
Not Following	No Exercise	Non Alcoholic	Regular	High Class
Following	Regular Exercise	Non Alcoholic	Regular	High Class
Not Following	Regular Exercise	Non Alcoholic	Regular	High Class
Following	Regular Exercise	Non Alcoholic	Occasionally	High Class
Following	Regular Exercise	Non Alcoholic	Non Smoker	High Class
Not Following	No Exercise	Non Alcoholic	Regular	Poor
Following	No Exercise	Non Alcoholic	Regular	Middle Class
Following	Regular Exercise	Non Alcoholic	Regular	Middle Class
Not Following	No Exercise	Non Alcoholic	Occasionally	Poor
Following	Regular Exercise	Non Alcoholic	Regular	Poor
Not Following	Regular Exercise	Non Alcoholic	Regular	Poor
Following	Regular Exercise	Alcoholic	Occasionally	Middle Class
Not Following	Regular Exercise	Non Alcoholic	Regular	Poor
Not Following	Regular Exercise	Non Alcoholic	Occasionally	Poor
Not Following	No Exercise	Non Alcoholic	Regular	Poor
Following	Regular Exercise	Non Alcoholic	Regular	Poor
Not Following	No Exercise	Non Alcoholic	Occasionally	Poor
Not Following	Regular Exercise	Non Alcoholic	Regular	High Class
Following	Regular Exercise	Non Alcoholic	Regular	Middle Class
Following	Regular Exercise	Non Alcoholic	Regular	Middle Class
Not Following	Regular Exercise	Non Alcoholic	Occasionally	Middle Class
Following	Regular Exercise	Non Alcoholic	Regular	High Class
Following	Regular Exercise	Non Alcoholic	Regular	High Class
Following	No Exercise	Alcoholic	Regular	High Class
Not Following	Regular Exercise	Non Alcoholic	Regular	Middle Class
Following	Regular Exercise	Non Alcoholic	Occasionally	Middle Class
Not Following	Regular Exercise	Non Alcoholic	Regular	Middle Class
Following	No Exercise	Non Alcoholic	Regular	Middle Class
Following	No Exercise	Non Alcoholic	Occasionally	Middle Class
Not Following	Regular Exercise	Non Alcoholic	Regular	Middle Class
Not Following	Regular Exercise	Non Alcoholic	Occasionally	Poor
Following	Regular Exercise	Non Alcoholic	Regular	Poor
Not Following	No Exercise	Non Alcoholic	Regular	High Class
Following	No Exercise	Non Alcoholic	Occasionally	High Class
Not Following	Regular Exercise	Non Alcoholic	Regular	High Class
Not Following	Regular Exercise	Alcoholic	Regular	Poor
Following	Regular Exercise	Non Alcoholic	Regular	Poor

Not Following	Regular Exercise	Non Alcoholic	Occasionally	Poor
Not Following	Regular Exercise	Non Alcoholic	Regular	Poor
Occasionally	Regular Exercise	Alcoholic	Regular	Poor
Not Following	Regular Exercise	Non Alcoholic	Regular	High Class
Not Following	No Exercise	Non Alcoholic	Regular	Middle Class
Not Following	Regular Exercise	Non Alcoholic	Occasionally	Middle Class
Occasionally	No Exercise	Non Alcoholic	Regular	Middle Class
Occasionally	Regular Exercise	Non Alcoholic	Regular	Middle Class
Occasionally	Regular Exercise	Non Alcoholic	Occasionally	Middle Class
Not Following	Regular Exercise	Non Alcoholic	Regular	Middle Class
Not Following	Regular Exercise	Non Alcoholic	Occasionally	Middle Class
Occasionally	Regular Exercise	Non Alcoholic	Regular	Middle Class
Not Following	No Exercise	Non Alcoholic	Regular	Middle Class
Not Following	Regular Exercise	Non Alcoholic	Occasionally	Middle Class
Occasionally	Regular Exercise	Occasionally	Regular	Middle Class
Occasionally	Regular Exercise	Non Alcoholic	Regular	Middle Class
Not Following	No Exercise	Non Alcoholic	Regular	Middle Class
Not Following	Regular Exercise	Non Alcoholic	Non Smoker	Poor
Following	Occasionally	Alcoholic	Regular	Poor
Following	Regular Exercise	Non Alcoholic	Regular	Poor
Not Following	Regular Exercise	Non Alcoholic	Regular	Poor
Not Following	Occasionally	Non Alcoholic	Regular	Poor
Not Following	Regular Exercise	Non Alcoholic	Occasionally	Poor
Not Following	Regular Exercise	Non Alcoholic	Regular	Poor
Not Following	Regular Exercise	Non Alcoholic	Regular	Poor
Following	Regular Exercise	Non Alcoholic	Occasionally	High Class
Not Following	No Exercise	Occasionally	Regular	High Class
Following	Regular Exercise	Non Alcoholic	Occasionally	High Class
Not Following	Regular Exercise	Non Alcoholic	Regular	High Class
Following	No Exercise	Non Alcoholic	Regular	High Class
Following	Occasionally	Non Alcoholic	Occasionally	High Class
Following	Occasionally	Non Alcoholic	Regular	Middle Class
Not Following	No Exercise	Non Alcoholic	Regular	Middle Class
Following	Occasionally	Non Alcoholic	Regular	Middle Class
Following	Regular Exercise	Occasionally	Occasionally	High Class
Following	No Exercise	Non Alcoholic	Regular	High Class
Occasionally	Regular Exercise	Non Alcoholic	Regular	High Class
Following	No Exercise	Non Alcoholic	Regular	Poor
Occasionally	Regular Exercise	Alcoholic	Regular	Poor
Following	Regular Exercise	Non Alcoholic	Occasionally	Middle Class
Occasionally	Regular Exercise	Non Alcoholic	Regular	High Class
Occasionally	No Exercise	Non Alcoholic	Regular	Middle Class
Not Following	Regular Exercise	Non Alcoholic	Occasionally	Poor
Following	No Exercise	Non Alcoholic	Regular	Poor
Not Following	Regular Exercise	Non Alcoholic	Occasionally	Poor
Following	No Exercise	Non Alcoholic	Regular	Poor
Following	Regular Exercise	Non Alcoholic	Non Smoker	Poor

Not Following	Occasionally	Non Alcoholic	Occasionally	Poor
Not Following	Regular Exercise	Non Alcoholic	Regular	Poor
Following	Regular Exercise	Non Alcoholic	Regular	High Class
Occasionally	Regular Exercise	Non Alcoholic	Regular	Middle Class
Following	Regular Exercise	Non Alcoholic	Occasionally	High Class
Occasionally	Occasionally	Non Alcoholic	Regular	Poor
Occasionally	Regular Exercise	Non Alcoholic	Regular	Poor
Occasionally	Regular Exercise	Occasionally	Regular	Poor
Following	Regular Exercise	Non Alcoholic	Regular	Poor
Not Following	Occasionally	Non Alcoholic	Occasionally	High Class
Following	Regular Exercise	Non Alcoholic	Regular	High Class
Not Following	Regular Exercise	Non Alcoholic	Regular	High Class
Not Following	Regular Exercise	Non Alcoholic	Occasionally	High Class
Not Following	No Exercise	Non Alcoholic	Regular	Middle Class
Not Following	Regular Exercise	Non Alcoholic	Occasionally	Middle Class
Not Following	Regular Exercise	Non Alcoholic	Regular	Middle Class
Not Following	Regular Exercise	Occasionally	Regular	Middle Class
Following	Regular Exercise	Occasionally	Occasionally	Middle Class
Not Following	Regular Exercise	Non Alcoholic	Regular	High Class
Not Following	Regular Exercise	Non Alcoholic	Regular	High Class
Not Following	No Exercise	Non Alcoholic	Non Smoker	High Class
Occasionally	Regular Exercise	Non Alcoholic	Occasionally	Poor
Following	Regular Exercise	Non Alcoholic	Regular	Poor
Following	Regular Exercise	Non Alcoholic	Regular	Middle Class
Following	Regular Exercise	Occasionally	Regular	Poor
Following	No Exercise	Occasionally	Regular	Middle Class
Not Following	Regular Exercise	Non Alcoholic	Occasionally	Poor
Following	No Exercise	Non Alcoholic	Regular	Poor
Not Following	Regular Exercise	Non Alcoholic	Regular	Middle Class
Not Following	No Exercise	Non Alcoholic	Occasionally	Poor
Not Following	Regular Exercise	Non Alcoholic	Regular	High Class
Following	Regular Exercise	Non Alcoholic	Occasionally	High Class
Not Following	Regular Exercise	Non Alcoholic	Regular	High Class
Following	Regular Exercise	Non Alcoholic	Regular	Poor
Not Following	Regular Exercise	Non Alcoholic	Occasionally	Middle Class
Following	Regular Exercise	Non Alcoholic	Regular	High Class
Not Following	No Exercise	Non Alcoholic	Regular	Poor
Not Following	Regular Exercise	Non Alcoholic	Regular	Poor
Following	Regular Exercise	Non Alcoholic	Occasionally	Poor
Not Following	Regular Exercise	Non Alcoholic	Regular	Poor
Following	No Exercise	Non Alcoholic	Regular	Poor
Following	No Exercise	Non Alcoholic	Regular	Poor
Not Following	Regular Exercise	Non Alcoholic	Regular	Middle Class
Not Following	Regular Exercise	Occasionally	Occasionally	Middle Class
Not Following	No Exercise	Non Alcoholic	Regular	Middle Class
Not Following	Regular Exercise	Occasionally	Non Smoker	High Class
Not Following	No Exercise	Non Alcoholic	Occasionally	High Class

Not Following	Regular Exercise	Non Alcoholic	Regular	High Class
Not Following	No Exercise	Non Alcoholic	Occasionally	High Class
Following	Regular Exercise	Non Alcoholic	Regular	High Class
Following	Regular Exercise	Non Alcoholic	Regular	High Class
Not Following	Regular Exercise	Non Alcoholic	Occasionally	Middle Class
Not Following	No Exercise	Non Alcoholic	Regular	Poor
Following	No Exercise	Non Alcoholic	Regular	Poor
Following	No Exercise	Non Alcoholic	Regular	Poor
Following	Regular Exercise	Alcoholic	Occasionally	Poor
Following	Regular Exercise	Non Alcoholic	Regular	Middle Class
Following	No Exercise	Non Alcoholic	Regular	Middle Class
Not Following	Regular Exercise	Non Alcoholic	Regular	Middle Class
Not Following	Regular Exercise	Non Alcoholic	Regular	Middle Class
Following	Regular Exercise	Non Alcoholic	Occasionally	Middle Class
Not Following	No Exercise	Non Alcoholic	Regular	Middle Class
Not Following	No Exercise	Alcoholic	Regular	High Class
Not Following	Regular Exercise	Non Alcoholic	Occasionally	High Class
Not Following	Regular Exercise	Non Alcoholic	Regular	High Class
Following	Regular Exercise	Non Alcoholic	Occasionally	High Class
Not Following	No Exercise	Non Alcoholic	Regular	High Class
Following	Occasionally	Non Alcoholic	Regular	High Class
Not Following	No Exercise	Non Alcoholic	Occasionally	High Class
Not Following	No Exercise	Non Alcoholic	Regular	High Class
Following	No Exercise	Non Alcoholic	Non Smoker	Poor
Occasionally	Regular Exercise	Non Alcoholic	Regular	Poor
Occasionally	Occasionally	Non Alcoholic	Occasionally	Poor
Not Following	Regular Exercise	Non Alcoholic	Regular	Middle Class
Not Following	Regular Exercise	Occasionally	Non Smoker	Poor
Not Following	No Exercise	Non Alcoholic	Regular	Poor
Occasionally	Regular Exercise	Non Alcoholic	Regular	High Class
Not Following	Regular Exercise	Non Alcoholic	Occasionally	Poor
Not Following	No Exercise	Non Alcoholic	Regular	Poor
Occasionally	Regular Exercise	Non Alcoholic	Regular	Middle Class
Following	No Exercise	Non Alcoholic	Occasionally	Poor
Following	Regular Exercise	Non Alcoholic	Regular	Poor
Occasionally	No Exercise	Non Alcoholic	Occasionally	High Class
Not Following	Regular Exercise	Non Alcoholic	Regular	Poor
Occasionally	Occasionally	Non Alcoholic	Regular	Poor
Occasionally	Regular Exercise	Non Alcoholic	Occasionally	Middle Class
Occasionally	Regular Exercise	Non Alcoholic	Regular	Poor
Occasionally	Regular Exercise	Non Alcoholic	Regular	Poor
Following	Regular Exercise	Non Alcoholic	Regular	Middle Class
Occasionally	Regular Exercise	Non Alcoholic	Occasionally	Poor
Occasionally	No Exercise	Non Alcoholic	Regular	Poor
Following	Occasionally	Non Alcoholic	Regular	Middle Class
Occasionally	No Exercise	Alcoholic	Regular	Middle Class
Not Following	No Exercise	Non Alcoholic	Regular	Middle Class

Not Following	Regular Exercise	Non Alcoholic	Occasionally	Middle Class
Not Following	Regular Exercise	Non Alcoholic	Non Smoker	Poor
Following	No Exercise	Non Alcoholic	Regular	Poor
Following	Regular Exercise	Alcoholic	Occasionally	Poor
Following	No Exercise	Non Alcoholic	Regular	High Class
Following	No Exercise	Non Alcoholic	Occasionally	High Class
Not Following	No Exercise	Non Alcoholic	Regular	High Class
Following	Occasionally	Non Alcoholic	Regular	High Class
Occasionally	Regular Exercise	Non Alcoholic	Occasionally	High Class
Occasionally	Regular Exercise	Non Alcoholic	Regular	Poor
Occasionally	No Exercise	Non Alcoholic	Regular	Poor
Following	Regular Exercise	Alcoholic	Regular	High Class
Not Following	No Exercise	Non Alcoholic	Occasionally	High Class
Not Following	Regular Exercise	Non Alcoholic	Regular	High Class
Following	No Exercise	Non Alcoholic	Regular	High Class
Following	Regular Exercise	Non Alcoholic	Regular	Middle Class
Following	No Exercise	Non Alcoholic	Regular	Middle Class
Not Following	Occasionally	Non Alcoholic	Occasionally	Middle Class
Following	No Exercise	Non Alcoholic	Regular	Middle Class
Following	No Exercise	Non Alcoholic	Regular	High Class
Not Following	Occasionally	Non Alcoholic	Occasionally	Middle Class
Not Following	No Exercise	Non Alcoholic	Regular	Middle Class
Not Following	Occasionally	Occasionally	Occasionally	High Class
Following	Regular Exercise	Non Alcoholic	Regular	Middle Class
Following	No Exercise	Non Alcoholic	Regular	Middle Class
Following	No Exercise	Non Alcoholic	Occasionally	Poor
Not Following	Occasionally	Non Alcoholic	Non Smoker	Poor
Following	No Exercise	Non Alcoholic	Regular	High Class
Not Following	No Exercise	Occasionally	Regular	High Class
Not Following	Occasionally	Non Alcoholic	Occasionally	High Class
Not Following	Regular Exercise	Non Alcoholic	Regular	High Class
Following	Regular Exercise	Non Alcoholic	Regular	Poor
Not Following	Regular Exercise	Non Alcoholic	Regular	Poor
Following	No Exercise	Non Alcoholic	Regular	Poor
Following	No Exercise	Non Alcoholic	Occasionally	High Class
Following	Regular Exercise	Non Alcoholic	Regular	High Class
Not Following	Regular Exercise	Non Alcoholic	Regular	High Class
Following	Regular Exercise	Non Alcoholic	Occasionally	High Class
Not Following	Regular Exercise	Non Alcoholic	Regular	High Class
Not Following	Occasionally	Non Alcoholic	Occasionally	Poor
Following	Regular Exercise	Alcoholic	Regular	High Class
Following	Regular Exercise	Non Alcoholic	Regular	Middle Class
Following	Regular Exercise	Non Alcoholic	Occasionally	Middle Class
Following	Regular Exercise	Non Alcoholic	Regular	Poor
Following	No Exercise	Non Alcoholic	Regular	Middle Class
Following	Regular Exercise	Non Alcoholic	Regular	Poor
Not Following	Regular Exercise	Non Alcoholic	Occasionally	Poor

Following	Occasionally	Non Alcoholic	Regular	High Class
Following	No Exercise	Non Alcoholic	Regular	Poor
Following	Regular Exercise	Non Alcoholic	Regular	High Class
Following	Regular Exercise	Non Alcoholic	Regular	Middle Class
Following	No Exercise	Non Alcoholic	Non Smoker	Poor
Following	Regular Exercise	Non Alcoholic	Regular	Poor
Following	No Exercise	Non Alcoholic	Regular	High Class
Not Following	Regular Exercise	Non Alcoholic	Occasionally	High Class
Not Following	No Exercise	Non Alcoholic	Non Smoker	Middle Class
Not Following	No Exercise	Occasionally	Occasionally	High Class
Not Following	Regular Exercise	Non Alcoholic	Regular	High Class
Following	Regular Exercise	Non Alcoholic	Regular	High Class
Following	No Exercise	Non Alcoholic	Occasionally	Poor
Occasionally	Occasionally	Non Alcoholic	Regular	Middle Class
Following	No Exercise	Non Alcoholic	Regular	Poor
Following	Regular Exercise	Non Alcoholic	Regular	Middle Class
Not Following	No Exercise	Non Alcoholic	Occasionally	Poor
Following	Regular Exercise	Non Alcoholic	Regular	Poor
Following	Occasionally	Non Alcoholic	Regular	Poor
Following	Regular Exercise	Occasionally	Regular	Middle Class
Not Following	Regular Exercise	Non Alcoholic	Regular	Middle Class
Not Following	No Exercise	Non Alcoholic	Occasionally	Middle Class
Following	No Exercise	Non Alcoholic	Regular	Middle Class
Not Following	Regular Exercise	Non Alcoholic	Regular	High Class
Following	No Exercise	Non Alcoholic	Occasionally	High Class
Not Following	Regular Exercise	Non Alcoholic	Regular	High Class
Following	Regular Exercise	Non Alcoholic	Occasionally	High Class
Not Following	Regular Exercise	Alcoholic	Regular	High Class
Following	Regular Exercise	Non Alcoholic	Regular	High Class
Not Following	Regular Exercise	Non Alcoholic	Non Smoker	High Class
Following	Regular Exercise	Non Alcoholic	Regular	Middle Class
Occasionally	Regular Exercise	Non Alcoholic	Regular	Middle Class
Not Following	Regular Exercise	Non Alcoholic	Regular	Middle Class
Occasionally	Regular Exercise	Non Alcoholic	Occasionally	High Class
Not Following	Regular Exercise	Non Alcoholic	Regular	High Class
Not Following	Regular Exercise	Non Alcoholic	Regular	High Class
Occasionally	Regular Exercise	Non Alcoholic	Regular	High Class
Not Following	Regular Exercise	Non Alcoholic	Regular	High Class
Occasionally	Regular Exercise	Non Alcoholic	Occasionally	High Class
Not Following	Regular Exercise	Non Alcoholic	Regular	High Class
Not Following	Regular Exercise	Alcoholic	Regular	Middle Class
Occasionally	Regular Exercise	Non Alcoholic	Occasionally	Middle Class
Not Following	Regular Exercise	Non Alcoholic	Regular	Middle Class
Occasionally	Regular Exercise	Non Alcoholic	Occasionally	High Class
Not Following	Regular Exercise	Non Alcoholic	Regular	High Class
Occasionally	No Exercise	Non Alcoholic	Regular	Middle Class
Occasionally	Occasionally	Non Alcoholic	Occasionally	High Class

Not Following	No Exercise	Alcoholic	Regular	Middle Class
Not Following	No Exercise	Non Alcoholic	Regular	High Class
Occasionally	Regular Exercise	Non Alcoholic	Regular	Poor
Occasionally	Regular Exercise	Non Alcoholic	Occasionally	Poor
Not Following	Regular Exercise	Non Alcoholic	Regular	Poor
Not Following	Regular Exercise	Alcoholic	Regular	Poor
Not Following	Regular Exercise	Non Alcoholic	Regular	Poor
Not Following	No Exercise	Non Alcoholic	Non Smoker	Poor
Not Following	Regular Exercise	Non Alcoholic	Occasionally	Middle Class
Not Following	No Exercise	Non Alcoholic	Regular	Middle Class
Following	Regular Exercise	Non Alcoholic	Regular	Middle Class
Following	Occasionally	Non Alcoholic	Occasionally	Middle Class
Following	Regular Exercise	Non Alcoholic	Regular	High Class
Not Following	Regular Exercise	Non Alcoholic	Occasionally	Poor
Not Following	No Exercise	Alcoholic	Regular	Poor
Following	No Exercise	Non Alcoholic	Regular	High Class
Following	No Exercise	Non Alcoholic	Occasionally	Middle Class
Not Following	No Exercise	Alcoholic	Regular	Middle Class
Occasionally	Regular Exercise	Alcoholic	Regular	Middle Class
Following	Regular Exercise	Non Alcoholic	Regular	High Class
Occasionally	Regular Exercise	Non Alcoholic	Occasionally	High Class
Following	No Exercise	Non Alcoholic	Regular	High Class
Following	Regular Exercise	Non Alcoholic	Regular	Poor
Following	Regular Exercise	Non Alcoholic	Regular	Poor
Occasionally	Regular Exercise	Non Alcoholic	Regular	High Class
Following	No Exercise	Non Alcoholic	Occasionally	Middle Class
Not Following	Regular Exercise	Non Alcoholic	Regular	High Class
Following	Regular Exercise	Non Alcoholic	Regular	Poor
Not Following	Regular Exercise	Non Alcoholic	Occasionally	High Class
Not Following	No Exercise	Non Alcoholic	Regular	High Class
Not Following	Regular Exercise	Alcoholic	Occasionally	High Class
Not Following	No Exercise	Non Alcoholic	Regular	High Class
Following	Regular Exercise	Non Alcoholic	Non Smoker	Poor
Not Following	No Exercise	Non Alcoholic	Occasionally	High Class
Not Following	Regular Exercise	Non Alcoholic	Regular	High Class
Following	No Exercise	Non Alcoholic	Regular	Poor
Not Following	Regular Exercise	Non Alcoholic	Non Smoker	High Class
Following	No Exercise	Non Alcoholic	Occasionally	Poor
Not Following	Regular Exercise	Non Alcoholic	Regular	Middle Class
Following	No Exercise	Alcoholic	Regular	Poor
Not Following	Regular Exercise	Alcoholic	Regular	Middle Class
Not Following	No Exercise	Non Alcoholic	Regular	Middle Class
Following	Regular Exercise	Non Alcoholic	Occasionally	Poor
Following	No Exercise	Non Alcoholic	Regular	Poor
Not Following	Regular Exercise	Non Alcoholic	Regular	Middle Class
Following	No Exercise	Non Alcoholic	Occasionally	Middle Class
Following	Regular Exercise	Non Alcoholic	Regular	Middle Class

Not Following	Regular Exercise	Non Alcoholic	Occasionally	Middle Class
Not Following	Regular Exercise	Non Alcoholic	Regular	Middle Class
Following	No Exercise	Non Alcoholic	Regular	Poor
Not Following	No Exercise	Alcoholic	Occasionally	Middle Class
Following	No Exercise	Non Alcoholic	Regular	Middle Class
Following	Regular Exercise	Non Alcoholic	Regular	High Class
Following	No Exercise	Non Alcoholic	Regular	High Class
Not Following	No Exercise	Non Alcoholic	Occasionally	High Class
Not Following	No Exercise	Non Alcoholic	Regular	High Class
Not Following	No Exercise	Non Alcoholic	Regular	High Class
Following	Regular Exercise	Non Alcoholic	Non Smoker	Poor
Not Following	Regular Exercise	Non Alcoholic	Regular	Poor
Following	Regular Exercise	Non Alcoholic	Occasionally	Poor
Following	No Exercise	Non Alcoholic	Regular	Poor
Not Following	No Exercise	Non Alcoholic	Regular	High Class
Following	Regular Exercise	Non Alcoholic	Occasionally	Poor
Not Following	No Exercise	Alcoholic	Regular	High Class
Not Following	Regular Exercise	Non Alcoholic	Occasionally	High Class
Following	Regular Exercise	Non Alcoholic	Regular	Poor
Following	Occasionally	Non Alcoholic	Regular	Poor
Following	Regular Exercise	Non Alcoholic	Occasionally	Poor
Following	Occasionally	Non Alcoholic	Regular	Middle Class
Occasionally	No Exercise	Non Alcoholic	Regular	Poor
Following	No Exercise	Non Alcoholic	Regular	High Class
Following	No Exercise	Non Alcoholic	Occasionally	High Class
Occasionally	No Exercise	Occasionally	Regular	High Class
Following	Regular Exercise	Non Alcoholic	Regular	Middle Class
Occasionally	No Exercise	Non Alcoholic	Regular	Middle Class
Following	No Exercise	Non Alcoholic	Regular	Poor
Following	No Exercise	Non Alcoholic	Occasionally	Poor
Not Following	Regular Exercise	Occasionally	Regular	Middle Class
Not Following	Regular Exercise	Occasionally	Regular	Middle Class
Following	Regular Exercise	Non Alcoholic	Occasionally	Poor
Not Following	Regular Exercise	Non Alcoholic	Regular	High Class
Not Following	Regular Exercise	Non Alcoholic	Occasionally	High Class
Following	Regular Exercise	Non Alcoholic	Non Smoker	Poor
Not Following	Regular Exercise	Non Alcoholic	Regular	High Class
Not Following	Regular Exercise	Non Alcoholic	Occasionally	High Class
Not Following	Occasionally	Non Alcoholic	Regular	High Class
Not Following	No Exercise	Non Alcoholic	Regular	Middle Class
Following	Occasionally	Alcoholic	Regular	Poor
Occasionally	No Exercise	Non Alcoholic	Occasionally	Middle Class
Occasionally	No Exercise	Non Alcoholic	Regular	High Class
Occasionally	Occasionally	Non Alcoholic	Regular	High Class
Not Following	Regular Exercise	Non Alcoholic	Regular	Middle Class
Not Following	Regular Exercise	Non Alcoholic	Regular	High Class
Not Following	No Exercise	Non Alcoholic	Occasionally	High Class

Occasionally	Regular Exercise	Non Alcoholic	Regular	High Class
Not Following	Regular Exercise	Non Alcoholic	Regular	Middle Class
Not Following	Regular Exercise	Non Alcoholic	Occasionally	High Class
Occasionally	Regular Exercise	Non Alcoholic	Regular	High Class
Not Following	Regular Exercise	Non Alcoholic	Occasionally	Middle Class
Occasionally	Regular Exercise	Non Alcoholic	Regular	High Class
Not Following	No Exercise	Non Alcoholic	Regular	High Class
Following	No Exercise	Occasionally	Occasionally	Poor
Following	Regular Exercise	Non Alcoholic	Regular	Middle Class
Following	No Exercise	Non Alcoholic	Regular	Middle Class
Following	Regular Exercise	Non Alcoholic	Non Smoker	Poor
Not Following	No Exercise	Non Alcoholic	Occasionally	Poor
Following	No Exercise	Non Alcoholic	Regular	Middle Class
Following	No Exercise	Non Alcoholic	Regular	Poor
Not Following	No Exercise	Non Alcoholic	Regular	Middle Class
Not Following	Regular Exercise	Non Alcoholic	Regular	High Class
Not Following	Regular Exercise	Non Alcoholic	Occasionally	High Class
Following	No Exercise	Non Alcoholic	Regular	High Class
Not Following	Regular Exercise	Non Alcoholic	Regular	High Class
Occasionally	No Exercise	Non Alcoholic	Occasionally	High Class
Not Following	Occasionally	Alcoholic	Regular	High Class
Not Following	No Exercise	Non Alcoholic	Occasionally	High Class
Not Following	Regular Exercise	Non Alcoholic	Regular	High Class
Not Following	No Exercise	Non Alcoholic	Regular	High Class
Occasionally	Regular Exercise	Non Alcoholic	Occasionally	High Class
Not Following	Regular Exercise	Non Alcoholic	Regular	High Class
Not Following	Regular Exercise	Occasionally	Regular	High Class
Following	No Exercise	Non Alcoholic	Regular	High Class
Not Following	No Exercise	Non Alcoholic	Occasionally	High Class
Following	Regular Exercise	Non Alcoholic	Regular	High Class
Following	No Exercise	Non Alcoholic	Occasionally	High Class
Not Following	Regular Exercise	Occasionally	Regular	Middle Class
Not Following	Regular Exercise	Occasionally	Regular	Middle Class
Not Following	Regular Exercise	Non Alcoholic	Occasionally	Middle Class
Not Following	Occasionally	Non Alcoholic	Regular	Poor
Occasionally	Regular Exercise	Non Alcoholic	Regular	Poor
Occasionally	Regular Exercise	Non Alcoholic	Regular	High Class
Occasionally	Occasionally	Occasionally	Occasionally	Middle Class
Occasionally	Regular Exercise	Non Alcoholic	Regular	High Class
Following	Regular Exercise	Non Alcoholic	Regular	Poor
Not Following	Regular Exercise	Non Alcoholic	Regular	Middle Class
Not Following	Regular Exercise	Non Alcoholic	Regular	High Class
Not Following	Regular Exercise	Non Alcoholic	Occasionally	Poor
Not Following	Regular Exercise	Occasionally	Non Smoker	Middle Class
Not Following	Occasionally	Non Alcoholic	Regular	High Class
Not Following	Regular Exercise	Non Alcoholic	Occasionally	Middle Class
Following	Regular Exercise	Occasionally	Regular	Poor

Not Following	Occasionally	Non Alcoholic	Occasionally	High Class
Not Following	Regular Exercise	Non Alcoholic	Regular	Middle Class
Not Following	Regular Exercise	Non Alcoholic	Regular	Poor
Not Following	Regular Exercise	Non Alcoholic	Occasionally	High Class
Following	No Exercise	Non Alcoholic	Regular	Middle Class
Following	No Exercise	Non Alcoholic	Regular	High Class
Not Following	No Exercise	Occasionally	Regular	Middle Class
Not Following	No Exercise	Non Alcoholic	Occasionally	Poor
Following	No Exercise	Non Alcoholic	Regular	Poor
Not Following	Regular Exercise	Non Alcoholic	Regular	Poor
Following	Regular Exercise	Non Alcoholic	Regular	Poor
Following	Occasionally	Occasionally	Regular	High Class
Occasionally	Regular Exercise	Non Alcoholic	Occasionally	High Class
Not Following	No Exercise	Non Alcoholic	Regular	Poor
Not Following	Regular Exercise	Non Alcoholic	Regular	High Class
Occasionally	No Exercise	Non Alcoholic	Occasionally	High Class
Occasionally	Regular Exercise	Non Alcoholic	Regular	Middle Class
Occasionally	No Exercise	Occasionally	Occasionally	High Class
Occasionally	Regular Exercise	Non Alcoholic	Regular	Middle Class
Occasionally	Regular Exercise	Non Alcoholic	Regular	High Class
Not Following	No Exercise	Non Alcoholic	Occasionally	Poor
Following	Occasionally	Non Alcoholic	Regular	Poor
Not Following	No Exercise	Non Alcoholic	Regular	Poor
Occasionally	No Exercise	Occasionally	Regular	Poor
Following	Regular Exercise	Non Alcoholic	Occasionally	Poor
Following	Regular Exercise	Non Alcoholic	Non Smoker	High Class
Not Following	Regular Exercise	Non Alcoholic	Regular	High Class
Not Following	Regular Exercise	Non Alcoholic	Regular	Poor
Following	Regular Exercise	Non Alcoholic	Regular	Poor
Not Following	Regular Exercise	Non Alcoholic	Occasionally	High Class
Not Following	Occasionally	Non Alcoholic	Regular	Poor
Following	No Exercise	Alcoholic	Regular	Middle Class
Occasionally	Regular Exercise	Non Alcoholic	Occasionally	Middle Class
Not Following	Regular Exercise	Non Alcoholic	Regular	Middle Class
Following	Regular Exercise	Non Alcoholic	Occasionally	Poor
Following	No Exercise	Non Alcoholic	Regular	Poor
Not Following	Regular Exercise	Non Alcoholic	Regular	High Class
Not Following	No Exercise	Non Alcoholic	Occasionally	Middle Class
Not Following	Regular Exercise	Non Alcoholic	Regular	Poor
Not Following	Occasionally	Non Alcoholic	Regular	Poor
Occasionally	Regular Exercise	Non Alcoholic	Regular	Middle Class
Following	No Exercise	Non Alcoholic	Occasionally	Middle Class
Occasionally	Regular Exercise	Non Alcoholic	Regular	High Class
Following	Occasionally	Non Alcoholic	Regular	High Class
Occasionally	Regular Exercise	Alcoholic	Regular	High Class
Following	No Exercise	Non Alcoholic	Regular	Poor
Occasionally	Regular Exercise	Non Alcoholic	Non Smoker	Poor

Occasionally	No Exercise	Non Alcoholic	Regular	High Class
Following	Occasionally	Non Alcoholic	Regular	Middle Class
Following	No Exercise	Non Alcoholic	Occasionally	High Class
Occasionally	Regular Exercise	Alcoholic	Regular	Middle Class
Occasionally	Occasionally	Non Alcoholic	Occasionally	High Class
Occasionally	Regular Exercise	Non Alcoholic	Regular	High Class
Not Following	Regular Exercise	Non Alcoholic	Regular	High Class
Not Following	Regular Exercise	Non Alcoholic	Occasionally	High Class
Not Following	No Exercise	Non Alcoholic	Regular	High Class
Not Following	Occasionally	Non Alcoholic	Regular	Poor
Not Following	No Exercise	Occasionally	Regular	High Class
Following	Occasionally	Non Alcoholic	Occasionally	High Class
Following	No Exercise	Non Alcoholic	Regular	High Class
Following	No Exercise	Non Alcoholic	Regular	High Class
Not Following	No Exercise	Occasionally	Regular	High Class
Following	Occasionally	Non Alcoholic	Regular	Middle Class
Not Following	Regular Exercise	Non Alcoholic	Occasionally	High Class
Following	Occasionally	Non Alcoholic	Regular	Middle Class
Not Following	Regular Exercise	Non Alcoholic	Regular	Poor
Not Following	No Exercise	Occasionally	Occasionally	High Class
Following	No Exercise	Non Alcoholic	Regular	Middle Class
Following	Regular Exercise	Non Alcoholic	Occasionally	Poor
Not Following	No Exercise	Occasionally	Regular	Poor
Following	Regular Exercise	Non Alcoholic	Regular	Poor
Following	Regular Exercise	Occasionally	Occasionally	Poor
Following	Regular Exercise	Occasionally	Regular	Poor
Following	Regular Exercise	Non Alcoholic	Regular	High Class
Not Following	Regular Exercise	Non Alcoholic	Regular	Poor
Not Following	Occasionally	Non Alcoholic	Occasionally	High Class
Not Following	No Exercise	Non Alcoholic	Regular	Poor
Following	No Exercise	Non Alcoholic	Regular	Poor
Not Following	No Exercise	Non Alcoholic	Regular	High Class
Following	Regular Exercise	Non Alcoholic	Regular	Middle Class
Following	No Exercise	Non Alcoholic	Occasionally	Middle Class
Not Following	No Exercise	Non Alcoholic	Regular	Middle Class
Not Following	No Exercise	Non Alcoholic	Regular	Poor
Following	Occasionally	Non Alcoholic	Occasionally	High Class
Not Following	Regular Exercise	Non Alcoholic	Regular	Middle Class
Following	Regular Exercise	Non Alcoholic	Occasionally	Poor
Following	Regular Exercise	Non Alcoholic	Regular	Poor
Not Following	Regular Exercise	Non Alcoholic	Regular	High Class
Not Following	Occasionally	Non Alcoholic	Occasionally	Poor
Not Following	Regular Exercise	Non Alcoholic	Regular	Poor
Not Following	Regular Exercise	Occasionally	Regular	Middle Class
Not Following	No Exercise	Occasionally	Regular	Poor
Not Following	Occasionally	Non Alcoholic	Occasionally	Poor
Following	No Exercise	Non Alcoholic	Regular	High Class

Not Following	Occasionally	Non Alcoholic	Regular	High Class
Not Following	No Exercise	Non Alcoholic	Regular	Poor
Following	No Exercise	Non Alcoholic	Regular	Poor
Not Following	Regular Exercise	Non Alcoholic	Occasionally	Poor
Not Following	Regular Exercise	Non Alcoholic	Regular	Poor
Following	No Exercise	Non Alcoholic	Regular	Poor
Not Following	Regular Exercise	Non Alcoholic	Non Smoker	High Class
Not Following	Regular Exercise	Occasionally	Regular	High Class
Not Following	Regular Exercise	Non Alcoholic	Occasionally	High Class
Not Following	Regular Exercise	Non Alcoholic	Regular	Middle Class
Following	Regular Exercise	Non Alcoholic	Regular	High Class
Following	Regular Exercise	Non Alcoholic	Occasionally	Poor
Following	No Exercise	Non Alcoholic	Regular	Poor
Following	No Exercise	Non Alcoholic	Regular	Poor
Following	Regular Exercise	Non Alcoholic	Regular	Poor
Not Following	No Exercise	Occasionally	Occasionally	High Class
Following	Regular Exercise	Non Alcoholic	Regular	High Class
Not Following	No Exercise	Non Alcoholic	Regular	High Class
Following	No Exercise	Non Alcoholic	Regular	High Class
Following	No Exercise	Non Alcoholic	Regular	Middle Class
Occasionally	Occasionally	Non Alcoholic	Occasionally	Middle Class
Not Following	Regular Exercise	Non Alcoholic	Regular	Middle Class
Not Following	Regular Exercise	Non Alcoholic	Regular	Middle Class
Not Following	No Exercise	Non Alcoholic	Occasionally	Middle Class
Occasionally	Regular Exercise	Non Alcoholic	Regular	High Class
Occasionally	No Exercise	Occasionally	Occasionally	High Class
Not Following	Regular Exercise	Non Alcoholic	Regular	High Class
Occasionally	No Exercise	Non Alcoholic	Non Smoker	Poor
Not Following	Regular Exercise	Non Alcoholic	Occasionally	Poor
Occasionally	No Exercise	Non Alcoholic	Regular	Middle Class
Occasionally	Regular Exercise	Non Alcoholic	Regular	Poor
Not Following	Regular Exercise	Non Alcoholic	Regular	Middle Class
Not Following	Regular Exercise	Occasionally	Occasionally	Poor
Occasionally	No Exercise	Non Alcoholic	Regular	Poor
Following	Regular Exercise	Non Alcoholic	Regular	Middle Class
Following	No Exercise	Non Alcoholic	Regular	Poor
Following	Regular Exercise	Non Alcoholic	Regular	High Class
Not Following	Regular Exercise	Non Alcoholic	Occasionally	High Class
Not Following	Regular Exercise	Non Alcoholic	Regular	High Class
Not Following	Regular Exercise	Non Alcoholic	Regular	Poor
Occasionally	Regular Exercise	Alcoholic	Occasionally	Middle Class
Occasionally	No Exercise	Non Alcoholic	Regular	High Class
Following	No Exercise	Non Alcoholic	Occasionally	Poor
Following	No Exercise	Non Alcoholic	Regular	Poor
Following	No Exercise	Non Alcoholic	Regular	Poor
Occasionally	Regular Exercise	Non Alcoholic	Occasionally	Poor
Occasionally	No Exercise	Non Alcoholic	Regular	Poor

Following	Regular Exercise	Non Alcoholic	Regular	Poor
Occasionally	No Exercise	Non Alcoholic	Regular	Middle Class
Occasionally	Regular Exercise	Non Alcoholic	Occasionally	Middle Class
Occasionally	No Exercise	Non Alcoholic	Regular	Middle Class
Occasionally	Regular Exercise	Non Alcoholic	Regular	High Class
Occasionally	No Exercise	Non Alcoholic	Regular	High Class
Occasionally	No Exercise	Non Alcoholic	Regular	High Class
Occasionally	Regular Exercise	Alcoholic	Occasionally	High Class
Occasionally	No Exercise	Non Alcoholic	Regular	High Class
Occasionally	Regular Exercise	Non Alcoholic	Regular	High Class
Occasionally	No Exercise	Non Alcoholic	Occasionally	Middle Class
Occasionally	Regular Exercise	Non Alcoholic	Regular	Poor
Following	Regular Exercise	Non Alcoholic	Occasionally	Poor
Not Following	No Exercise	Non Alcoholic	Regular	Poor
Not Following	Regular Exercise	Non Alcoholic	Regular	Poor
Not Following	No Exercise	Non Alcoholic	Occasionally	Middle Class
Following	Regular Exercise	Non Alcoholic	Regular	Middle Class
Not Following	No Exercise	Non Alcoholic	Regular	Middle Class
Following	Regular Exercise	Non Alcoholic	Regular	Middle Class
Not Following	No Exercise	Non Alcoholic	Occasionally	Middle Class
Following	Regular Exercise	Non Alcoholic	Regular	Middle Class
Following	Regular Exercise	Non Alcoholic	Regular	High Class
Not Following	No Exercise	Non Alcoholic	Regular	High Class
Not Following	Regular Exercise	Non Alcoholic	Regular	High Class
Following	Regular Exercise	Non Alcoholic	Occasionally	High Class
Following	No Exercise	Non Alcoholic	Regular	High Class
Not Following	Regular Exercise	Non Alcoholic	Regular	High Class
Following	No Exercise	Non Alcoholic	Occasionally	High Class
Not Following	Regular Exercise	Occasionally	Regular	High Class
Not Following	Regular Exercise	Non Alcoholic	Occasionally	Poor
Following	No Exercise	Non Alcoholic	Non Smoker	Poor
Not Following	No Exercise	Non Alcoholic	Regular	Poor
Following	Regular Exercise	Non Alcoholic	Occasionally	Middle Class
Following	No Exercise	Non Alcoholic	Regular	Poor
Not Following	Regular Exercise	Non Alcoholic	Regular	Poor
Not Following	No Exercise	Occasionally	Regular	High Class
Not Following	Regular Exercise	Non Alcoholic	Occasionally	Poor
Following	Regular Exercise	Non Alcoholic	Regular	Poor
Not Following	No Exercise	Occasionally	Regular	Middle Class
Following	Regular Exercise	Non Alcoholic	Regular	Poor
Occasionally	No Exercise	Non Alcoholic	Regular	Poor
Not Following	Regular Exercise	Non Alcoholic	Occasionally	High Class
Following	No Exercise	Non Alcoholic	Regular	Poor
Not Following	Regular Exercise	Occasionally	Regular	Poor
Not Following	No Exercise	Occasionally	Occasionally	Middle Class
Following	Occasionally	Occasionally	Regular	Poor
Not Following	Regular Exercise	Non Alcoholic	Occasionally	Poor

Following	No Exercise	Non Alcoholic	Regular	Middle Class
Not Following	Regular Exercise	Non Alcoholic	Regular	Poor
Following	Regular Exercise	Non Alcoholic	Occasionally	Poor
Not Following	No Exercise	Non Alcoholic	Regular	Middle Class
Not Following	Regular Exercise	Non Alcoholic	Regular	Middle Class
Not Following	No Exercise	Alcoholic	Regular	Middle Class
Not Following	Regular Exercise	Non Alcoholic	Occasionally	Middle Class
Following	Occasionally	Non Alcoholic	Regular	Poor
Not Following	Regular Exercise	Non Alcoholic	Regular	Poor
Not Following	No Exercise	Occasionally	Regular	Poor
Not Following	Regular Exercise	Occasionally	Regular	High Class
Not Following	No Exercise	Occasionally	Occasionally	High Class
Following	Regular Exercise	Non Alcoholic	Non Smoker	High Class
Following	Occasionally	Non Alcoholic	Regular	High Class
Following	Regular Exercise	Occasionally	Occasionally	High Class
Not Following	No Exercise	Non Alcoholic	Regular	Poor
Following	Regular Exercise	Non Alcoholic	Occasionally	Poor
Not Following	No Exercise	Occasionally	Regular	High Class
Following	Occasionally	Non Alcoholic	Regular	High Class
Not Following	Regular Exercise	Non Alcoholic	Occasionally	High Class
Not Following	No Exercise	Non Alcoholic	Regular	High Class
Following	No Exercise	Non Alcoholic	Regular	Middle Class
Not Following	Regular Exercise	Non Alcoholic	Regular	Middle Class
Following	Regular Exercise	Non Alcoholic	Occasionally	Middle Class
Not Following	Regular Exercise	Non Alcoholic	Regular	Middle Class
Not Following	Regular Exercise	Non Alcoholic	Regular	High Class
Following	Regular Exercise	Occasionally	Regular	Middle Class
Not Following	Occasionally	Non Alcoholic	Regular	Middle Class
Following	Occasionally	Non Alcoholic	Occasionally	High Class
Not Following	Occasionally	Non Alcoholic	Regular	Middle Class
Occasionally	Regular Exercise	Non Alcoholic	Regular	Middle Class
Occasionally	Occasionally	Non Alcoholic	Occasionally	Poor
Following	Occasionally	Non Alcoholic	Regular	Poor
Occasionally	Regular Exercise	Occasionally	Non Smoker	High Class
Following	No Exercise	Non Alcoholic	Regular	High Class
Occasionally	No Exercise	Non Alcoholic	Regular	High Class
Following	Regular Exercise	Non Alcoholic	Occasionally	High Class
Occasionally	Regular Exercise	Non Alcoholic	Regular	Poor
Following	Regular Exercise	Alcoholic	Regular	Poor
Not Following	Regular Exercise	Non Alcoholic	Regular	Poor
Not Following	Regular Exercise	Occasionally	Occasionally	High Class
Not Following	No Exercise	Non Alcoholic	Regular	High Class
Not Following	Regular Exercise	Non Alcoholic	Regular	High Class
Not Following	No Exercise	Non Alcoholic	Regular	High Class
Not Following	Regular Exercise	Non Alcoholic	Regular	High Class
Following	Regular Exercise	Non Alcoholic	Occasionally	Poor
Not Following	No Exercise	Non Alcoholic	Regular	High Class

Following	No Exercise	Non Alcoholic	Regular	Middle Class
Not Following	No Exercise	Non Alcoholic	Occasionally	Middle Class
Not Following	Regular Exercise	Non Alcoholic	Regular	Poor
Not Following	No Exercise	Occasionally	Occasionally	Middle Class
Following	Regular Exercise	Non Alcoholic	Regular	Poor
Following	No Exercise	Non Alcoholic	Regular	Poor
Occasionally	Regular Exercise	Non Alcoholic	Occasionally	High Class
Following	No Exercise	Non Alcoholic	Regular	Poor
Occasionally	Regular Exercise	Non Alcoholic	Regular	High Class
Not Following	No Exercise	Occasionally	Regular	Middle Class
Following	Regular Exercise	Non Alcoholic	Occasionally	Poor
Not Following	Regular Exercise	Non Alcoholic	Regular	Poor
Following	Regular Exercise	Non Alcoholic	Regular	High Class
Following	Regular Exercise	Non Alcoholic	Regular	High Class
Occasionally	Regular Exercise	Non Alcoholic	Regular	Middle Class
Occasionally	Regular Exercise	Alcoholic	Occasionally	High Class
Occasionally	No Exercise	Non Alcoholic	Regular	High Class
Occasionally	Regular Exercise	Non Alcoholic	Regular	High Class
Occasionally	No Exercise	Non Alcoholic	Occasionally	Poor
Occasionally	Regular Exercise	Non Alcoholic	Regular	Middle Class
Occasionally	No Exercise	Non Alcoholic	Occasionally	Poor
Occasionally	Regular Exercise	Occasionally	Regular	Middle Class
Occasionally	Regular Exercise	Non Alcoholic	Regular	Poor
Occasionally	Regular Exercise	Non Alcoholic	Occasionally	Poor
Occasionally	Regular Exercise	Non Alcoholic	Regular	Poor
Occasionally	No Exercise	Non Alcoholic	Regular	Middle Class
Not Following	No Exercise	Non Alcoholic	Regular	Middle Class
Following	No Exercise	Alcoholic	Occasionally	Middle Class
Not Following	Regular Exercise	Non Alcoholic	Regular	Middle Class
Not Following	No Exercise	Non Alcoholic	Regular	High Class
Following	Regular Exercise	Non Alcoholic	Regular	High Class
Occasionally	Regular Exercise	Non Alcoholic	Regular	High Class
Not Following	Regular Exercise	Non Alcoholic	Occasionally	High Class
Not Following	No Exercise	Non Alcoholic	Regular	High Class
Occasionally	No Exercise	Non Alcoholic	Regular	High Class
Occasionally	Regular Exercise	Occasionally	Occasionally	High Class
Not Following	No Exercise	Alcoholic	Non Smoker	Middle Class
Occasionally	No Exercise	Non Alcoholic	Occasionally	Middle Class
Following	Regular Exercise	Non Alcoholic	Regular	Middle Class
Following	Regular Exercise	Non Alcoholic	Regular	High Class
Not Following	No Exercise	Non Alcoholic	Occasionally	High Class
Following	No Exercise	Non Alcoholic	Regular	High Class
Following	Regular Exercise	Non Alcoholic	Regular	High Class
Following	No Exercise	Non Alcoholic	Regular	High Class
Following	Regular Exercise	Occasionally	Occasionally	High Class
Following	No Exercise	Non Alcoholic	Regular	High Class
Following	Regular Exercise	Non Alcoholic	Regular	Middle Class

Following	Regular Exercise	Non Alcoholic	Regular	Middle Class
Following	No Exercise	Occasionally	Regular	Middle Class
Following	No Exercise	Non Alcoholic	Occasionally	High Class
Following	No Exercise	Non Alcoholic	Regular	High Class
Following	Regular Exercise	Non Alcoholic	Regular	Middle Class
Not Following	No Exercise	Non Alcoholic	Occasionally	High Class
Not Following	Regular Exercise	Non Alcoholic	Regular	Middle Class
Following	No Exercise	Non Alcoholic	Occasionally	High Class
Not Following	Regular Exercise	Non Alcoholic	Regular	Poor
Following	Occasionally	Occasionally	Regular	Poor
Following	Regular Exercise	Non Alcoholic	Occasionally	Poor
Following	No Exercise	Non Alcoholic	Regular	Poor
Not Following	No Exercise	Non Alcoholic	Regular	Poor
Following	Regular Exercise	Non Alcoholic	Regular	Poor
Not Following	Regular Exercise	Non Alcoholic	Occasionally	Middle Class
Following	Regular Exercise	Non Alcoholic	Regular	Middle Class
Following	Regular Exercise	Occasionally	Regular	Middle Class
Not Following	Occasionally	Non Alcoholic	Regular	Middle Class
Not Following	No Exercise	Occasionally	Non Smoker	High Class
Not Following	Regular Exercise	Non Alcoholic	Occasionally	Poor
Following	Regular Exercise	Non Alcoholic	Regular	Poor
Following	Regular Exercise	Non Alcoholic	Regular	High Class
Following	No Exercise	Non Alcoholic	Occasionally	Middle Class
Not Following	Regular Exercise	Non Alcoholic	Regular	Middle Class
Not Following	Regular Exercise	Non Alcoholic	Occasionally	Middle Class
Following	Regular Exercise	Non Alcoholic	Regular	High Class
Following	Occasionally	Non Alcoholic	Regular	High Class
Not Following	No Exercise	Alcoholic	Occasionally	High Class
Not Following	No Exercise	Non Alcoholic	Regular	Poor
Not Following	No Exercise	Non Alcoholic	Regular	Poor
Not Following	Regular Exercise	Non Alcoholic	Regular	Poor
Occasionally	No Exercise	Non Alcoholic	Occasionally	High Class
Occasionally	Regular Exercise	Non Alcoholic	Regular	Poor
Not Following	No Exercise	Non Alcoholic	Regular	Poor
Not Following	Regular Exercise	Occasionally	Regular	Middle Class
Not Following	No Exercise	Non Alcoholic	Regular	High Class
Occasionally	Regular Exercise	Non Alcoholic	Occasionally	High Class
Not Following	Occasionally	Non Alcoholic	Regular	Poor
Not Following	Regular Exercise	Alcoholic	Regular	High Class
Following	No Exercise	Non Alcoholic	Non Smoker	High Class
Following	Regular Exercise	Non Alcoholic	Regular	High Class
Not Following	Regular Exercise	Non Alcoholic	Occasionally	Middle Class
Not Following	No Exercise	Non Alcoholic	Regular	High Class
Following	No Exercise	Non Alcoholic	Regular	Poor
Not Following	Regular Exercise	Non Alcoholic	Occasionally	High Class
Not Following	No Exercise	Non Alcoholic	Regular	Poor
Not Following	Regular Exercise	Non Alcoholic	Regular	Poor

Following	No Exercise	Non Alcoholic	Regular	High Class
Following	Regular Exercise	Non Alcoholic	Occasionally	Poor
Following	Regular Exercise	Non Alcoholic	Regular	Poor
Not Following	Regular Exercise	Occasionally	Regular	High Class
Not Following	No Exercise	Non Alcoholic	Regular	Middle Class
Occasionally	Regular Exercise	Non Alcoholic	Regular	High Class
Following	No Exercise	Non Alcoholic	Occasionally	Middle Class
Occasionally	No Exercise	Non Alcoholic	Regular	Middle Class
Occasionally	Regular Exercise	Non Alcoholic	Regular	High Class
Following	No Exercise	Non Alcoholic	Occasionally	High Class
Not Following	No Exercise	Non Alcoholic	Regular	Poor
Not Following	Regular Exercise	Non Alcoholic	Occasionally	Poor
Following	Regular Exercise	Non Alcoholic	Regular	Poor
Not Following	No Exercise	Non Alcoholic	Regular	Middle Class
Not Following	Regular Exercise	Non Alcoholic	Occasionally	High Class
Not Following	Regular Exercise	Non Alcoholic	Regular	High Class
Not Following	No Exercise	Non Alcoholic	Regular	High Class
Not Following	Regular Exercise	Non Alcoholic	Regular	Poor
Not Following	No Exercise	Non Alcoholic	Occasionally	Poor
Occasionally	Regular Exercise	Non Alcoholic	Regular	Poor
Occasionally	Regular Exercise	Non Alcoholic	Regular	High Class
Occasionally	No Exercise	Non Alcoholic	Regular	Poor
Occasionally	Regular Exercise	Alcoholic	Regular	Poor
Not Following	Regular Exercise	Non Alcoholic	Occasionally	High Class
Not Following	Regular Exercise	Non Alcoholic	Regular	Middle Class
Not Following	No Exercise	Occasionally	Regular	Middle Class
Not Following	Regular Exercise	Occasionally	Occasionally	High Class
Not Following	No Exercise	Non Alcoholic	Regular	High Class
Occasionally	Regular Exercise	Non Alcoholic	Occasionally	Poor
Not Following	No Exercise	Non Alcoholic	Regular	Poor
Following	Regular Exercise	Non Alcoholic	Regular	Poor
Following	No Exercise	Non Alcoholic	Occasionally	Middle Class
Following	Regular Exercise	Non Alcoholic	Regular	High Class
Following	Regular Exercise	Non Alcoholic	Regular	High Class
Not Following	Regular Exercise	Non Alcoholic	Regular	Middle Class
Occasionally	Regular Exercise	Non Alcoholic	Occasionally	Middle Class
Occasionally	Regular Exercise	Non Alcoholic	Regular	Middle Class
Occasionally	Regular Exercise	Non Alcoholic	Regular	High Class
Occasionally	No Exercise	Alcoholic	Regular	Poor
Following	Regular Exercise	Non Alcoholic	Regular	Middle Class
Not Following	Regular Exercise	Non Alcoholic	Occasionally	Poor
Following	Regular Exercise	Non Alcoholic	Regular	Poor
Following	No Exercise	Non Alcoholic	Non Smoker	Poor
Not Following	Regular Exercise	Non Alcoholic	Occasionally	Poor
Not Following	Regular Exercise	Non Alcoholic	Regular	Poor
Not Following	Regular Exercise	Non Alcoholic	Occasionally	High Class
Not Following	Regular Exercise	Occasionally	Regular	Middle Class

Not Following	No Exercise	Non Alcoholic	Regular	High Class
Not Following	No Exercise	Non Alcoholic	Occasionally	Middle Class
Not Following	No Exercise	Non Alcoholic	Regular	High Class
Not Following	Regular Exercise	Non Alcoholic	Regular	Poor
Following	No Exercise	Non Alcoholic	Regular	Middle Class
Following	Regular Exercise	Non Alcoholic	Occasionally	Poor
Following	No Exercise	Non Alcoholic	Regular	Middle Class
Following	Regular Exercise	Non Alcoholic	Regular	Poor
Not Following	No Exercise	Non Alcoholic	Regular	Poor
Following	Occasionally	Non Alcoholic	Regular	Poor
Following	Regular Exercise	Occasionally	Occasionally	High Class
Not Following	No Exercise	Non Alcoholic	Regular	High Class
Not Following	No Exercise	Occasionally	Regular	High Class
Not Following	Regular Exercise	Non Alcoholic	Occasionally	High Class
Not Following	Regular Exercise	Non Alcoholic	Regular	High Class
Not Following	No Exercise	Non Alcoholic	Occasionally	Middle Class
Following	No Exercise	Non Alcoholic	Regular	Poor
Following	Occasionally	Occasionally	Regular	High Class
Following	No Exercise	Non Alcoholic	Occasionally	High Class
Following	Regular Exercise	Non Alcoholic	Regular	Middle Class
Following	No Exercise	Non Alcoholic	Regular	High Class
Not Following	Regular Exercise	Non Alcoholic	Regular	High Class
Following	Regular Exercise	Non Alcoholic	Occasionally	Poor
Occasionally	No Exercise	Non Alcoholic	Regular	Poor
Occasionally	Occasionally	Non Alcoholic	Non Smoker	Middle Class
Occasionally	No Exercise	Non Alcoholic	Regular	Middle Class
Occasionally	Regular Exercise	Non Alcoholic	Regular	Middle Class
Not Following	No Exercise	Non Alcoholic	Occasionally	Middle Class
Following	Regular Exercise	Occasionally	Regular	Middle Class
Following	No Exercise	Non Alcoholic	Regular	High Class
Following	Regular Exercise	Non Alcoholic	Occasionally	High Class
Following	Occasionally	Non Alcoholic	Regular	Middle Class
Not Following	Regular Exercise	Non Alcoholic	Occasionally	Middle Class
Not Following	Occasionally	Non Alcoholic	Regular	High Class
Not Following	No Exercise	Non Alcoholic	Regular	Poor
Following	Regular Exercise	Non Alcoholic	Occasionally	Poor
Following	Regular Exercise	Non Alcoholic	Regular	High Class
Following	Regular Exercise	Non Alcoholic	Regular	High Class
Not Following	Regular Exercise	Occasionally	Regular	Poor
Following	Regular Exercise	Non Alcoholic	Occasionally	Poor
Not Following	Regular Exercise	Non Alcoholic	Regular	High Class
Following	Regular Exercise	Non Alcoholic	Regular	Poor
Following	No Exercise	Occasionally	Occasionally	Middle Class
Not Following	Regular Exercise	Non Alcoholic	Regular	Middle Class
Following	No Exercise	Non Alcoholic	Occasionally	Poor
Following	No Exercise	Non Alcoholic	Regular	Poor
Following	Regular Exercise	Non Alcoholic	Regular	High Class

Not Following	No Exercise	Non Alcoholic	Occasionally	Poor
Following	Regular Exercise	Non Alcoholic	Regular	Poor
Not Following	No Exercise	Non Alcoholic	Regular	Poor
Not Following	No Exercise	Non Alcoholic	Regular	Middle Class
Following	Regular Exercise	Non Alcoholic	Occasionally	High Class
Following	Regular Exercise	Non Alcoholic	Regular	Poor
Not Following	No Exercise	Occasionally	Regular	High Class
Following	Regular Exercise	Non Alcoholic	Regular	Middle Class
Not Following	Regular Exercise	Non Alcoholic	Regular	Poor
Not Following	No Exercise	Non Alcoholic	Occasionally	High Class
Following	Regular Exercise	Non Alcoholic	Regular	Poor
Following	Regular Exercise	Non Alcoholic	Regular	High Class
Not Following	No Exercise	Non Alcoholic	Occasionally	Poor
Following	Regular Exercise	Occasionally	Regular	High Class
Following	No Exercise	Non Alcoholic	Occasionally	Poor
Following	Regular Exercise	Non Alcoholic	Regular	Middle Class
Following	Regular Exercise	Non Alcoholic	Regular	Middle Class
Following	Regular Exercise	Non Alcoholic	Occasionally	Middle Class
Following	Regular Exercise	Non Alcoholic	Regular	Middle Class
Not Following	No Exercise	Occasionally	Regular	Middle Class
Following	Regular Exercise	Non Alcoholic	Regular	Poor
Not Following	No Exercise	Non Alcoholic	Occasionally	High Class
Not Following	Regular Exercise	Non Alcoholic	Regular	Middle Class
Following	No Exercise	Non Alcoholic	Regular	High Class
Following	Regular Exercise	Occasionally	Regular	Poor
Not Following	Regular Exercise	Non Alcoholic	Regular	High Class
Not Following	Regular Exercise	Non Alcoholic	Non Smoker	High Class
Following	Regular Exercise	Non Alcoholic	Regular	High Class
Not Following	No Exercise	Non Alcoholic	Regular	Poor
Not Following	Regular Exercise	Non Alcoholic	Occasionally	High Class
Not Following	No Exercise	Non Alcoholic	Non Smoker	Poor
Not Following	Regular Exercise	Non Alcoholic	Occasionally	Poor
Occasionally	No Exercise	Non Alcoholic	Regular	High Class
Occasionally	Regular Exercise	Non Alcoholic	Regular	Poor
Occasionally	Occasionally	Non Alcoholic	Regular	Middle Class
Not Following	Regular Exercise	Non Alcoholic	Regular	High Class
Occasionally	No Exercise	Non Alcoholic	Occasionally	High Class
Occasionally	Regular Exercise	Non Alcoholic	Regular	Middle Class
Occasionally	No Exercise	Non Alcoholic	Regular	High Class
Occasionally	Regular Exercise	Non Alcoholic	Non Smoker	High Class
Not Following	Regular Exercise	Alcoholic	Occasionally	Middle Class
Occasionally	Regular Exercise	Non Alcoholic	Regular	High Class
Occasionally	Regular Exercise	Non Alcoholic	Regular	Poor
Following	Occasionally	Occasionally	Regular	High Class
Not Following	Regular Exercise	Non Alcoholic	Regular	High Class
Not Following	Regular Exercise	Occasionally	Occasionally	High Class
Not Following	Regular Exercise	Non Alcoholic	Occasionally	Poor

Occasionally	Regular Exercise	Non Alcoholic	Regular	Poor
Occasionally	No Exercise	Non Alcoholic	Non Smoker	Middle Class
Occasionally	Regular Exercise	Non Alcoholic	Occasionally	High Class
Occasionally	No Exercise	Non Alcoholic	Regular	High Class
Occasionally	No Exercise	Occasionally	Regular	High Class
Occasionally	No Exercise	Non Alcoholic	Regular	Poor
Occasionally	Regular Exercise	Occasionally	Occasionally	Poor
Occasionally	Regular Exercise	Occasionally	Regular	Poor
Occasionally	No Exercise	Occasionally	Regular	High Class
Occasionally	Regular Exercise	Occasionally	Occasionally	High Class
Occasionally	Regular Exercise	Non Alcoholic	Regular	High Class
Occasionally	Regular Exercise	Non Alcoholic	Regular	Middle Class
Not Following	No Exercise	Non Alcoholic	Regular	Poor
Occasionally	Regular Exercise	Non Alcoholic	Regular	Middle Class
Not Following	No Exercise	Non Alcoholic	Occasionally	Poor
Not Following	No Exercise	Occasionally	Non Smoker	Poor
Not Following	No Exercise	Non Alcoholic	Regular	Poor
Not Following	Occasionally	Non Alcoholic	Occasionally	Poor
Following	No Exercise	Non Alcoholic	Regular	Poor
Occasionally	Regular Exercise	Non Alcoholic	Occasionally	High Class
Occasionally	Regular Exercise	Non Alcoholic	Regular	Middle Class
Occasionally	No Exercise	Non Alcoholic	Regular	High Class
Not Following	No Exercise	Non Alcoholic	Occasionally	Middle Class
Occasionally	Regular Exercise	Non Alcoholic	Regular	High Class
Following	No Exercise	Occasionally	Regular	Poor
Not Following	Regular Exercise	Occasionally	Regular	Middle Class
Not Following	No Exercise	Non Alcoholic	Occasionally	Poor
Not Following	Regular Exercise	Non Alcoholic	Regular	Middle Class
Not Following	Occasionally	Non Alcoholic	Regular	Poor
Occasionally	Regular Exercise	Occasionally	Non Smoker	High Class
Occasionally	No Exercise	Occasionally	Regular	High Class
Occasionally	Regular Exercise	Occasionally	Occasionally	High Class
Not Following	Regular Exercise	Non Alcoholic	Regular	High Class
Occasionally	Occasionally	Non Alcoholic	Occasionally	Middle Class
Occasionally	Regular Exercise	Non Alcoholic	Regular	High Class
Occasionally	No Exercise	Non Alcoholic	Regular	Middle Class
Occasionally	Regular Exercise	Non Alcoholic	Occasionally	Poor
Occasionally	Regular Exercise	Non Alcoholic	Regular	Middle Class
Occasionally	Occasionally	Non Alcoholic	Regular	Middle Class
Occasionally	Regular Exercise	Non Alcoholic	Non Smoker	Middle Class
Occasionally	No Exercise	Non Alcoholic	Regular	Middle Class
Following	No Exercise	Occasionally	Occasionally	High Class
Occasionally	Regular Exercise	Non Alcoholic	Regular	Poor
Occasionally	No Exercise	Non Alcoholic	Regular	High Class
Following	No Exercise	Non Alcoholic	Regular	High Class
Following	Occasionally	Non Alcoholic	Regular	Middle Class
Following	No Exercise	Occasionally	Occasionally	Middle Class

Following	No Exercise	Non Alcoholic	Regular	Middle Class
Occasionally	Occasionally	Non Alcoholic	Regular	Poor
Occasionally	Regular Exercise	Non Alcoholic	Occasionally	Poor
Occasionally	Occasionally	Non Alcoholic	Occasionally	High Class
Occasionally	Regular Exercise	Non Alcoholic	Regular	Middle Class
Occasionally	Regular Exercise	Non Alcoholic	Regular	Middle Class
Occasionally	No Exercise	Non Alcoholic	Regular	High Class
Following	Regular Exercise	Non Alcoholic	Occasionally	Middle Class
Following	Regular Exercise	Non Alcoholic	Non Smoker	Middle Class
Occasionally	No Exercise	Non Alcoholic	Regular	High Class
Following	No Exercise	Non Alcoholic	Regular	Poor
Following	No Exercise	Non Alcoholic	Regular	Poor
Following	Occasionally	Non Alcoholic	Regular	Middle Class
Occasionally	No Exercise	Non Alcoholic	Occasionally	High Class
Occasionally	Occasionally	Non Alcoholic	Regular	Poor
Following	No Exercise	Non Alcoholic	Regular	High Class
Not Following	Regular Exercise	Alcoholic	Regular	Middle Class
Following	Regular Exercise	Non Alcoholic	Non Smoker	Poor
Following	Regular Exercise	Non Alcoholic	Occasionally	High Class
Not Following	Regular Exercise	Occasionally	Regular	Poor
Following	No Exercise	Non Alcoholic	Regular	High Class
Not Following	Regular Exercise	Non Alcoholic	Occasionally	Poor
Following	No Exercise	Occasionally	Regular	High Class
Occasionally	Regular Exercise	Non Alcoholic	Occasionally	Poor
Following	No Exercise	Non Alcoholic	Regular	Middle Class
Occasionally	Regular Exercise	Non Alcoholic	Regular	Middle Class
Following	No Exercise	Non Alcoholic	Occasionally	Middle Class
Following	Regular Exercise	Non Alcoholic	Regular	Middle Class
Following	No Exercise	Non Alcoholic	Regular	Middle Class
Following	No Exercise	Non Alcoholic	Occasionally	High Class
Following	No Exercise	Non Alcoholic	Regular	Poor
Following	Regular Exercise	Non Alcoholic	Occasionally	High Class
Following	Regular Exercise	Occasionally	Regular	High Class
Following	Regular Exercise	Occasionally	Occasionally	High Class
Following	Regular Exercise	Alcoholic	Regular	High Class
Occasionally	No Exercise	Non Alcoholic	Regular	Middle Class
Occasionally	No Exercise	Non Alcoholic	Regular	High Class
Occasionally	No Exercise	Non Alcoholic	Occasionally	High Class
Occasionally	Regular Exercise	Alcoholic	Regular	Poor
Occasionally	Regular Exercise	Non Alcoholic	Regular	High Class
Occasionally	Regular Exercise	Non Alcoholic	Regular	Poor
Occasionally	No Exercise	Non Alcoholic	Regular	High Class
Occasionally	Regular Exercise	Non Alcoholic	Non Smoker	Poor
Following	Regular Exercise	Non Alcoholic	Non Smoker	Poor
Occasionally	Regular Exercise	Non Alcoholic	Regular	High Class
Occasionally	No Exercise	Non Alcoholic	Regular	High Class
Occasionally	Regular Exercise	Alcoholic	Occasionally	High Class

Occasionally	No Exercise	Alcoholic	Occasionally	High Class
Occasionally	Regular Exercise	Alcoholic	Regular	Poor
Following	Regular Exercise	Non Alcoholic	Regular	Middle Class
Occasionally	No Exercise	Alcoholic	Occasionally	Poor
Occasionally	No Exercise	Alcoholic	Regular	High Class
Occasionally	No Exercise	Non Alcoholic	Occasionally	Poor
Following	No Exercise	Non Alcoholic	Regular	Poor
Not Following	Regular Exercise	Non Alcoholic	Occasionally	Middle Class
Not Following	Regular Exercise	Occasionally	Regular	High Class
Not Following	No Exercise	Non Alcoholic	Occasionally	Poor
Not Following	Regular Exercise	Non Alcoholic	Regular	High Class
Not Following	Regular Exercise	Non Alcoholic	Occasionally	High Class
Occasionally	Regular Exercise	Non Alcoholic	Regular	High Class
Occasionally	No Exercise	Non Alcoholic	Occasionally	High Class
Occasionally	Regular Exercise	Non Alcoholic	Regular	High Class
Not Following	No Exercise	Non Alcoholic	Regular	High Class
Occasionally	No Exercise	Non Alcoholic	Regular	High Class
Not Following	No Exercise	Alcoholic	Non Smoker	High Class
Following	No Exercise	Alcoholic	Regular	High Class
Not Following	Regular Exercise	Occasionally	Occasionally	High Class
Not Following	Regular Exercise	Non Alcoholic	Regular	High Class
Not Following	Regular Exercise	Occasionally	Occasionally	Middle Class
Not Following	Regular Exercise	Non Alcoholic	Regular	High Class
Not Following	Regular Exercise	Non Alcoholic	Regular	High Class
Not Following	Regular Exercise	Non Alcoholic	Regular	High Class
Not Following	Regular Exercise	Non Alcoholic	Regular	High Class
Not Following	Occasionally	Non Alcoholic	Occasionally	Middle Class
Not Following	Regular Exercise	Alcoholic	Regular	Middle Class
Not Following	No Exercise	Non Alcoholic	Occasionally	Middle Class
Not Following	No Exercise	Occasionally	Regular	Poor
Following	Regular Exercise	Occasionally	Regular	Poor
Following	Regular Exercise	Alcoholic	Regular	High Class
Not Following	No Exercise	Non Alcoholic	Regular	High Class
Not Following	Occasionally	Occasionally	Non Smoker	High Class
Not Following	Regular Exercise	Non Alcoholic	Regular	High Class
Not Following	Regular Exercise	Occasionally	Regular	Middle Class
Following	No Exercise	Non Alcoholic	Occasionally	Middle Class
Following	Regular Exercise	Non Alcoholic	Regular	Middle Class
Not Following	No Exercise	Non Alcoholic	Occasionally	High Class
Not Following	Regular Exercise	Non Alcoholic	Regular	High Class
Not Following	Regular Exercise	Non Alcoholic	Regular	High Class
Not Following	Regular Exercise	Alcoholic	Occasionally	High Class
Not Following	Regular Exercise	Occasionally	Regular	High Class
Occasionally	No Exercise	Occasionally	Regular	Middle Class
Not Following	Regular Exercise	Non Alcoholic	Regular	Poor
Not Following	No Exercise	Non Alcoholic	Regular	Poor
Not Following	Regular Exercise	Non Alcoholic	Occasionally	Poor

Occasionally	Regular Exercise	Non Alcoholic	Regular	High Class
Occasionally	Occasionally	Non Alcoholic	Regular	High Class
Occasionally	Regular Exercise	Occasionally	Non Smoker	Middle Class
Occasionally	Regular Exercise	Non Alcoholic	Regular	Middle Class
Occasionally	Occasionally	Non Alcoholic	Occasionally	Middle Class
Occasionally	Regular Exercise	Non Alcoholic	Regular	Middle Class
Occasionally	No Exercise	Alcoholic	Regular	Middle Class
Occasionally	Regular Exercise	Non Alcoholic	Occasionally	Poor
Occasionally	Regular Exercise	Non Alcoholic	Regular	Middle Class
Occasionally	Regular Exercise	Occasionally	Regular	Poor
Occasionally	Regular Exercise	Non Alcoholic	Regular	Middle Class
Occasionally	Regular Exercise	Non Alcoholic	Regular	Middle Class
Occasionally	No Exercise	Alcoholic	Occasionally	Middle Class
Occasionally	Regular Exercise	Occasionally	Regular	Middle Class
Occasionally	Regular Exercise	Non Alcoholic	Non Smoker	Poor
Occasionally	No Exercise	Occasionally	Occasionally	Poor
Occasionally	Occasionally	Occasionally	Regular	Poor
Occasionally	Regular Exercise	Non Alcoholic	Regular	High Class
Not Following	Regular Exercise	Non Alcoholic	Occasionally	High Class
Not Following	Regular Exercise	Occasionally	Regular	High Class
Not Following	Regular Exercise	Non Alcoholic	Occasionally	Middle Class
Not Following	No Exercise	Non Alcoholic	Regular	High Class
Not Following	Occasionally	Non Alcoholic	Regular	High Class
Not Following	No Exercise	Non Alcoholic	Occasionally	Poor
Not Following	Regular Exercise	Non Alcoholic	Regular	Middle Class
Not Following	Regular Exercise	Non Alcoholic	Regular	Middle Class
Not Following	Occasionally	Occasionally	Regular	Middle Class
Not Following	Regular Exercise	Non Alcoholic	Non Smoker	Middle Class
Not Following	Occasionally	Non Alcoholic	Occasionally	High Class
Not Following	Regular Exercise	Non Alcoholic	Regular	Middle Class
Occasionally	Occasionally	Alcoholic	Occasionally	Poor
Not Following	Occasionally	Non Alcoholic	Occasionally	Middle Class
Following	Regular Exercise	Occasionally	Occasionally	High Class
Following	Regular Exercise	Non Alcoholic	Regular	Middle Class
Occasionally	Regular Exercise	Occasionally	Regular	Middle Class
Not Following	Occasionally	Non Alcoholic	Regular	High Class
Following	Regular Exercise	Non Alcoholic	Non Smoker	High Class
Following	Regular Exercise	Non Alcoholic	Occasionally	Poor
Following	Regular Exercise	Occasionally	Occasionally	Poor
Occasionally	No Exercise	Non Alcoholic	Regular	Middle Class
Following	No Exercise	Non Alcoholic	Regular	High Class
Occasionally	Regular Exercise	Occasionally	Regular	High Class
Occasionally	Occasionally	Occasionally	Regular	Middle Class
Occasionally	Regular Exercise	Non Alcoholic	Occasionally	Middle Class
Occasionally	Regular Exercise	Occasionally	Regular	High Class
Occasionally	Regular Exercise	Non Alcoholic	Occasionally	High Class
Occasionally	Regular Exercise	Alcoholic	Regular	Poor

Occasionally	Regular Exercise	Non Alcoholic	Occasionally	Poor
Occasionally	Occasionally	Occasionally	Occasionally	Poor
Occasionally	Regular Exercise	Non Alcoholic	Occasionally	High Class
Following	No Exercise	Non Alcoholic	Regular	Middle Class
Occasionally	No Exercise	Alcoholic	Regular	Middle Class
Occasionally	No Exercise	Alcoholic	Regular	Poor
Occasionally	Regular Exercise	Non Alcoholic	Occasionally	Middle Class
Following	Regular Exercise	Non Alcoholic	Non Smoker	Middle Class
Occasionally	No Exercise	Non Alcoholic	Non Smoker	Middle Class
Following	Occasionally	Non Alcoholic	Non Smoker	Poor
Occasionally	No Exercise	Occasionally	Non Smoker	Middle Class
Occasionally	No Exercise	Alcoholic	Regular	Middle Class
Occasionally	Regular Exercise	Occasionally	Regular	Middle Class
Not Following	No Exercise	Occasionally	Non Smoker	High Class
Not Following	No Exercise	Alcoholic	Occasionally	High Class
Following	Regular Exercise	Non Alcoholic	Occasionally	High Class
Occasionally	Regular Exercise	Alcoholic	Regular	Poor
Occasionally	Regular Exercise	Non Alcoholic	Non Smoker	Poor
Occasionally	Regular Exercise	Non Alcoholic	Occasionally	Middle Class
Occasionally	Regular Exercise	Occasionally	Regular	High Class
Not Following	Regular Exercise	Alcoholic	Regular	High Class
Not Following	Regular Exercise	Non Alcoholic	Non Smoker	Middle Class
Occasionally	Occasionally	Occasionally	Occasionally	High Class
Occasionally	Regular Exercise	Alcoholic	Regular	Poor
Occasionally	Regular Exercise	Alcoholic	Regular	Poor
Following	Regular Exercise	Non Alcoholic	Occasionally	High Class
Following	Regular Exercise	Non Alcoholic	Regular	Poor
Not Following	Regular Exercise	Non Alcoholic	Occasionally	High Class
Following	Regular Exercise	Occasionally	Regular	Middle Class
Not Following	Regular Exercise	Occasionally	Regular	High Class
Not Following	Regular Exercise	Non Alcoholic	Regular	Middle Class
Following	Regular Exercise	Non Alcoholic	Regular	Middle Class
Occasionally	Occasionally	Alcoholic	Occasionally	High Class
Occasionally	Regular Exercise	Non Alcoholic	Non Smoker	High Class
Following	Regular Exercise	Occasionally	Regular	Poor
Following	Occasionally	Occasionally	Occasionally	Poor
Occasionally	Regular Exercise	Occasionally	Regular	Middle Class
Following	Regular Exercise	Non Alcoholic	Regular	High Class
Not Following	Occasionally	Occasionally	Regular	Poor
Not Following	Regular Exercise	Alcoholic	Non Smoker	High Class
Not Following	Regular Exercise	Occasionally	Non Smoker	Poor
Occasionally	Occasionally	Occasionally	Occasionally	Poor
Following	Regular Exercise	Alcoholic	Regular	High Class
Following	Regular Exercise	Non Alcoholic	Occasionally	Middle Class
Occasionally	Occasionally	Non Alcoholic	Regular	Poor
Occasionally	Occasionally	Non Alcoholic	Occasionally	Poor
Following	Regular Exercise	Alcoholic	Regular	Poor

Following	Regular Exercise	Alcoholic	Occasionally	High Class
Occasionally	No Exercise	Non Alcoholic	Regular	Middle Class
Occasionally	Occasionally	Occasionally	Regular	Middle Class
Following	No Exercise	Alcoholic	Non Smoker	High Class
Following	Occasionally	Occasionally	Occasionally	Poor
Following	Occasionally	Alcoholic	Regular	Poor
Following	Regular Exercise	Non Alcoholic	Occasionally	Poor
Occasionally	Regular Exercise	Alcoholic	Occasionally	Poor
Occasionally	Regular Exercise	Alcoholic	Occasionally	High Class
Following	Regular Exercise	Occasionally	Regular	Middle Class
Following	Regular Exercise	Non Alcoholic	Non Smoker	High Class
Following	Occasionally	Alcoholic	Occasionally	Middle Class
Not Following	Regular Exercise	Occasionally	Occasionally	Middle Class
Following	Regular Exercise	Non Alcoholic	Non Smoker	Poor
Following	Regular Exercise	Non Alcoholic	Occasionally	Poor
Following	Regular Exercise	Occasionally	Occasionally	Poor
Not Following	Occasionally	Alcoholic	Regular	High Class
Following	Occasionally	Occasionally	Occasionally	High Class
Following	Occasionally	Non Alcoholic	Regular	Middle Class
Following	Regular Exercise	Alcoholic	Occasionally	Poor
Following	Occasionally	Occasionally	Regular	Middle Class
Following	Occasionally	Non Alcoholic	Occasionally	High Class
Following	Regular Exercise	Alcoholic	Regular	High Class
Occasionally	Occasionally	Occasionally	Occasionally	Poor
Following	Occasionally	Alcoholic	Regular	Middle Class
Occasionally	No Exercise	Non Alcoholic	Regular	Middle Class
Occasionally	Regular Exercise	Non Alcoholic	Regular	Middle Class
Occasionally	Occasionally	Alcoholic	Occasionally	Middle Class
Not Following	Occasionally	Non Alcoholic	Occasionally	Middle Class
Not Following	No Exercise	Occasionally	Regular	High Class
Following	Regular Exercise	Occasionally	Occasionally	Middle Class
Following	Regular Exercise	Occasionally	Occasionally	Middle Class
Following	Regular Exercise	Non Alcoholic	Regular	Poor
Not Following	Occasionally	Occasionally	Occasionally	Poor
Occasionally	Regular Exercise	Occasionally	Regular	High Class
Not Following	Occasionally	Non Alcoholic	Regular	High Class
Not Following	No Exercise	Non Alcoholic	Non Smoker	High Class
Following	Regular Exercise	Non Alcoholic	Non Smoker	High Class
Following	Regular Exercise	Non Alcoholic	Occasionally	Poor
Following	Regular Exercise	Non Alcoholic	Non Smoker	Poor
Following	Regular Exercise	Non Alcoholic	Occasionally	Poor
Following	Regular Exercise	Occasionally	Occasionally	High Class
Not Following	No Exercise	Non Alcoholic	Regular	High Class
Not Following	No Exercise	Non Alcoholic	Regular	High Class
Following	No Exercise	Non Alcoholic	Regular	High Class
Following	Occasionally	Alcoholic	Regular	High Class
Following	No Exercise	Non Alcoholic	Regular	Poor

Following	Regular Exercise	Occasionally	Regular	High Class
Not Following	Occasionally	Alcoholic	Regular	Middle Class
Not Following	Occasionally	Occasionally	Regular	Middle Class
Following	Regular Exercise	Non Alcoholic	Occasionally	Poor
Following	Occasionally	Alcoholic	Regular	Middle Class
Not Following	Regular Exercise	Occasionally	Regular	Poor
Following	Regular Exercise	Non Alcoholic	Occasionally	Poor
Following	Regular Exercise	Alcoholic	Regular	High Class
Following	Regular Exercise	Non Alcoholic	Occasionally	Poor
Occasionally	Occasionally	Occasionally	Occasionally	High Class
Not Following	No Exercise	Non Alcoholic	Non Smoker	Middle Class
Following	Regular Exercise	Non Alcoholic	Occasionally	Poor
Following	Regular Exercise	Occasionally	Regular	Poor
Following	No Exercise	Non Alcoholic	Regular	High Class
Not Following	No Exercise	Alcoholic	Regular	High Class
Following	Regular Exercise	Non Alcoholic	Occasionally	Middle Class
Following	Regular Exercise	Non Alcoholic	Non Smoker	High Class
Following	Regular Exercise	Non Alcoholic	Non Smoker	High Class
Occasionally	No Exercise	Alcoholic	Regular	High Class
Following	Regular Exercise	Non Alcoholic	Regular	Poor
Following	No Exercise	Non Alcoholic	Occasionally	Middle Class
Occasionally	No Exercise	Non Alcoholic	Regular	Poor
Following	Regular Exercise	Occasionally	Regular	Middle Class
Following	Regular Exercise	Non Alcoholic	Occasionally	Poor
Following	Regular Exercise	Non Alcoholic	Non Smoker	Poor
Not Following	No Exercise	Non Alcoholic	Non Smoker	Poor
Not Following	No Exercise	Non Alcoholic	Non Smoker	Middle Class
Not Following	Occasionally	Alcoholic	Regular	Middle Class
Following	Occasionally	Non Alcoholic	Non Smoker	Middle Class
Occasionally	No Exercise	Alcoholic	Regular	Middle Class
Following	Regular Exercise	Alcoholic	Regular	High Class
Following	Occasionally	Occasionally	Occasionally	High Class
Occasionally	No Exercise	Alcoholic	Regular	High Class
Not Following	Regular Exercise	Alcoholic	Occasionally	High Class
Following	Regular Exercise	Non Alcoholic	Non Smoker	High Class
Occasionally	No Exercise	Occasionally	Non Smoker	High Class
Occasionally	No Exercise	Alcoholic	Regular	High Class
Occasionally	Regular Exercise	Occasionally	Regular	Middle Class
Not Following	No Exercise	Occasionally	Non Smoker	Middle Class
Not Following	No Exercise	Non Alcoholic	Non Smoker	Middle Class
Not Following	No Exercise	Alcoholic	Occasionally	High Class
Occasionally	Occasionally	Occasionally	Occasionally	High Class
Following	Regular Exercise	Non Alcoholic	Occasionally	High Class
Following	Regular Exercise	Alcoholic	Regular	High Class
Following	Regular Exercise	Non Alcoholic	Non Smoker	High Class
Following	Regular Exercise	Non Alcoholic	Occasionally	High Class
Following	Regular Exercise	Occasionally	Regular	High Class

Following	Occasionally	Non Alcoholic	Occasionally	Middle Class
Not Following	Regular Exercise	Non Alcoholic	Regular	Middle Class
Following	Regular Exercise	Non Alcoholic	Non Smoker	Middle Class
Not Following	Regular Exercise	Alcoholic	Regular	High Class
Following	Regular Exercise	Non Alcoholic	Non Smoker	High Class
Not Following	Regular Exercise	Non Alcoholic	Non Smoker	Middle Class
Following	Occasionally	Occasionally	Occasionally	High Class
Following	Regular Exercise	Alcoholic	Regular	Middle Class
Not Following	Regular Exercise	Occasionally	Non Smoker	High Class
Following	Regular Exercise	Alcoholic	Regular	Poor
Following	Regular Exercise	Non Alcoholic	Regular	Poor
Following	Regular Exercise	Non Alcoholic	Occasionally	Poor
Following	Regular Exercise	Alcoholic	Regular	Poor
Following	Regular Exercise	Non Alcoholic	Regular	Poor
Not Following	Regular Exercise	Non Alcoholic	Occasionally	Poor
Following	Regular Exercise	Occasionally	Regular	Middle Class
Not Following	Regular Exercise	Occasionally	Regular	Middle Class
Not Following	Regular Exercise	Non Alcoholic	Regular	Middle Class
Following	Regular Exercise	Non Alcoholic	Regular	Middle Class
Occasionally	Occasionally	Alcoholic	Occasionally	High Class
Not Following	Regular Exercise	Non Alcoholic	Non Smoker	Poor
Following	Regular Exercise	Occasionally	Regular	Poor
Following	Regular Exercise	Non Alcoholic	Non Smoker	High Class
Following	Occasionally	Occasionally	Occasionally	Middle Class
Not Following	Regular Exercise	Occasionally	Regular	Middle Class
Occasionally	Occasionally	Alcoholic	Occasionally	Middle Class
Not Following	No Exercise	Non Alcoholic	Non Smoker	High Class
Following	Regular Exercise	Non Alcoholic	Regular	High Class
Not Following	No Exercise	Alcoholic	Occasionally	High Class
Not Following	Occasionally	Occasionally	Regular	Poor
Occasionally	Occasionally	Non Alcoholic	Non Smoker	Poor
Not Following	Regular Exercise	Alcoholic	Non Smoker	Poor
Not Following	Regular Exercise	Occasionally	Non Smoker	High Class
Occasionally	Occasionally	Occasionally	Occasionally	Poor
Following	Regular Exercise	Alcoholic	Regular	Poor
Following	Regular Exercise	Non Alcoholic	Occasionally	Middle Class
Occasionally	No Exercise	Alcoholic	Non Smoker	High Class
Following	No Exercise	Alcoholic	Regular	High Class
Not Following	Occasionally	Occasionally	Occasionally	Poor
Occasionally	Occasionally	Non Alcoholic	Regular	High Class
Occasionally	Occasionally	Non Alcoholic	Occasionally	High Class
Following	Regular Exercise	Alcoholic	Regular	High Class
Following	No Exercise	Occasionally	Non Smoker	Middle Class
Following	Regular Exercise	Alcoholic	Occasionally	High Class
Not Following	Occasionally	Occasionally	Regular	Poor
Occasionally	No Exercise	Non Alcoholic	Regular	High Class
Occasionally	Occasionally	Occasionally	Regular	Poor

Following	Regular Exercise	Alcoholic	Regular	Poor
Following	No Exercise	Alcoholic	Non Smoker	High Class
Following	Occasionally	Occasionally	Occasionally	Poor
Following	Occasionally	Occasionally	Non Smoker	Poor
Occasionally	Occasionally	Occasionally	Occasionally	High Class
Following	Occasionally	Alcoholic	Regular	Middle Class
Following	Regular Exercise	Non Alcoholic	Occasionally	High Class
Following	Regular Exercise	Alcoholic	Occasionally	Middle Class
Following	Regular Exercise	Non Alcoholic	Non Smoker	Middle Class
Following	Regular Exercise	Alcoholic	Occasionally	High Class
Following	Regular Exercise	Occasionally	Regular	High Class
Following	Regular Exercise	Non Alcoholic	Non Smoker	Poor
Following	Occasionally	Alcoholic	Occasionally	Poor
Occasionally	Regular Exercise	Occasionally	Non Smoker	Poor
Not Following	No Exercise	Occasionally	Regular	Middle Class
Following	Occasionally	Occasionally	Occasionally	High Class
Not Following	Regular Exercise	Occasionally	Non Smoker	High Class
Not Following	Regular Exercise	Occasionally	Occasionally	High Class
Following	Regular Exercise	Non Alcoholic	Non Smoker	Poor
Following	Regular Exercise	Non Alcoholic	Occasionally	Poor
Following	Regular Exercise	Occasionally	Occasionally	Poor
Occasionally	Occasionally	Occasionally	Non Smoker	High Class
Following	Regular Exercise	Alcoholic	Regular	Poor
Occasionally	Regular Exercise	Occasionally	Occasionally	Poor
Not Following	Occasionally	Alcoholic	Regular	High Class
Following	Occasionally	Occasionally	Occasionally	Middle Class
Following	Occasionally	Non Alcoholic	Regular	Middle Class
Following	Regular Exercise	Alcoholic	Occasionally	High Class
Following	Regular Exercise	Non Alcoholic	Non Smoker	High Class
Not Following	Regular Exercise	Occasionally	Regular	Poor
Following	Occasionally	Occasionally	Regular	Poor
Following	Occasionally	Non Alcoholic	Occasionally	Poor
Following	Regular Exercise	Alcoholic	Regular	Middle Class
Occasionally	Occasionally	Occasionally	Occasionally	High Class
Not Following	Regular Exercise	Alcoholic	Regular	High Class
Occasionally	No Exercise	Occasionally	Regular	Middle Class
Following	Occasionally	Alcoholic	Regular	Middle Class
Not Following	Regular Exercise	Alcoholic	Regular	Middle Class
Occasionally	No Exercise	Non Alcoholic	Regular	High Class
Occasionally	Regular Exercise	Non Alcoholic	Regular	Poor
Occasionally	Regular Exercise	Non Alcoholic	Regular	Middle Class
Following	No Exercise	Alcoholic	Occasionally	Poor
Not Following	Occasionally	Alcoholic	Regular	Poor
Following	Regular Exercise	Non Alcoholic	Occasionally	Poor
Following	Regular Exercise	Non Alcoholic	Non Smoker	Poor
Following	Regular Exercise	Non Alcoholic	Non Smoker	Poor
Occasionally	Regular Exercise	Occasionally	Regular	High Class

Following	Occasionally	Occasionally	Regular	Middle Class
Following	Regular Exercise	Non Alcoholic	Non Smoker	High Class
Occasionally	Occasionally	Occasionally	Occasionally	Middle Class
Following	Regular Exercise	Non Alcoholic	Non Smoker	High Class
Not Following	No Exercise	Alcoholic	Non Smoker	Poor
Following	Regular Exercise	Alcoholic	Regular	Middle Class
Not Following	Regular Exercise	Occasionally	Regular	Poor
Not Following	Regular Exercise	Occasionally	Regular	Middle Class
Not Following	Occasionally	Occasionally	Occasionally	Poor
Following	Regular Exercise	Alcoholic	Regular	Poor
Following	No Exercise	Occasionally	Occasionally	Poor
Following	Regular Exercise	Occasionally	Regular	High Class
Following	Regular Exercise	Occasionally	Non Smoker	High Class
Following	Occasionally	Non Alcoholic	Non Smoker	High Class
Following	Regular Exercise	Occasionally	Occasionally	High Class
Following	Regular Exercise	Non Alcoholic	Regular	High Class
Occasionally	Occasionally	Alcoholic	Regular	Middle Class
Not Following	No Exercise	Non Alcoholic	Regular	Poor
Occasionally	Occasionally	Non Alcoholic	Non Smoker	High Class
Not Following	Regular Exercise	Occasionally	Occasionally	High Class
Following	Regular Exercise	Occasionally	Regular	Middle Class
Occasionally	Occasionally	Occasionally	Regular	High Class
Not Following	No Exercise	Non Alcoholic	Non Smoker	High Class
Occasionally	Regular Exercise	Non Alcoholic	Occasionally	Poor
Following	Regular Exercise	Non Alcoholic	Non Smoker	Poor
Occasionally	Occasionally	Occasionally	Occasionally	Middle Class
Occasionally	Occasionally	Occasionally	Occasionally	Middle Class
Following	No Exercise	Occasionally	Regular	Middle Class
Not Following	No Exercise	Alcoholic	Regular	Middle Class
Not Following	Regular Exercise	Occasionally	Regular	Middle Class
Following	Regular Exercise	Non Alcoholic	Regular	High Class
Not Following	No Exercise	Occasionally	Occasionally	High Class
Following	Regular Exercise	Non Alcoholic	Non Smoker	Middle Class
Following	Regular Exercise	Alcoholic	Non Smoker	Middle Class
Not Following	Occasionally	Alcoholic	Regular	High Class
Not Following	Regular Exercise	Occasionally	Non Smoker	Poor
Following	Regular Exercise	Occasionally	Non Smoker	Poor
Following	Regular Exercise	Non Alcoholic	Regular	High Class
Following	Occasionally	Occasionally	Non Smoker	High Class
Not Following	No Exercise	Alcoholic	Occasionally	Poor
Following	Occasionally	Alcoholic	Regular	Poor
Not Following	Regular Exercise	Alcoholic	Regular	High Class
Following	Regular Exercise	Non Alcoholic	Regular	Poor
Following	No Exercise	Non Alcoholic	Occasionally	Middle Class
Following	No Exercise	Non Alcoholic	Non Smoker	Middle Class
Occasionally	Regular Exercise	Alcoholic	Non Smoker	Poor
Not Following	Occasionally	Occasionally	Regular	Poor

Not Following	Occasionally	Non Alcoholic	Regular	High Class
Following	Regular Exercise	Non Alcoholic	Regular	Poor
Not Following	No Exercise	Alcoholic	Regular	Poor
Following	Regular Exercise	Occasionally	Occasionally	Poor
Not Following	Regular Exercise	Non Alcoholic	Non Smoker	Middle Class
Following	Regular Exercise	Non Alcoholic	Non Smoker	High Class
Not Following	No Exercise	Occasionally	Non Smoker	Poor
Not Following	Regular Exercise	Alcoholic	Regular	High Class
Following	Regular Exercise	Non Alcoholic	Occasionally	Middle Class
Following	Occasionally	Occasionally	Occasionally	Poor
Following	Regular Exercise	Non Alcoholic	Occasionally	High Class
Following	No Exercise	Alcoholic	Occasionally	Poor
Not Following	No Exercise	Alcoholic	Occasionally	High Class
Occasionally	No Exercise	Alcoholic	Regular	Poor
Not Following	Occasionally	Non Alcoholic	Occasionally	High Class
Occasionally	Occasionally	Alcoholic	Regular	Poor
Not Following	No Exercise	Alcoholic	Regular	Middle Class
Following	Occasionally	Non Alcoholic	Regular	Middle Class
Not Following	Occasionally	Alcoholic	Regular	Middle Class
Not Following	Occasionally	Non Alcoholic	Regular	Middle Class
Not Following	No Exercise	Occasionally	Regular	Middle Class
Following	Occasionally	Non Alcoholic	Regular	Poor
Occasionally	No Exercise	Alcoholic	Non Smoker	High Class
Occasionally	Occasionally	Alcoholic	Regular	Middle Class
Occasionally	Occasionally	Occasionally	Occasionally	High Class
Not Following	No Exercise	Occasionally	Regular	Poor
Not Following	Occasionally	Non Alcoholic	Regular	High Class
Following	Regular Exercise	Alcoholic	Occasionally	High Class
Following	Regular Exercise	Occasionally	Occasionally	High Class
Following	No Exercise	Alcoholic	Occasionally	Poor
Following	Occasionally	Non Alcoholic	Occasionally	High Class
Not Following	Occasionally	Occasionally	Occasionally	Poor
Following	Occasionally	Occasionally	Regular	Poor
Not Following	Occasionally	Alcoholic	Regular	High Class
Following	Regular Exercise	Occasionally	Occasionally	Poor
Not Following	No Exercise	Non Alcoholic	Non Smoker	Middle Class
Not Following	Regular Exercise	Non Alcoholic	Regular	High Class
Occasionally	No Exercise	Alcoholic	Regular	High Class
Occasionally	Occasionally	Occasionally	Occasionally	Middle Class
Following	Occasionally	Occasionally	Regular	High Class
Not Following	Occasionally	Non Alcoholic	Regular	High Class
Following	Occasionally	Occasionally	Regular	Middle Class
Not Following	Occasionally	Occasionally	Occasionally	High Class
Not Following	Occasionally	Alcoholic	Occasionally	Poor

Diagnosed
Yes
No
Yes
Yes
No
No
No
Yes
No
No
No
No
No
No
No
No
No
No
Yes
Yes
Yes
Yes
Yes
No
No
Yes
Yes
Yes
Yes
Yes
Yes
Yes
Yes
No
Yes
No
No
No
No
No
No
No
No
No
No

No
No
No
No
No
No
No
Yes
Yes
No
No
Yes
No
No
No
Yes
No
Yes
No
Yes
Yes
No
Yes
No
No
Yes
No
No
Yes
No
Yes
Yes
Yes
No
Yes
No
No
Yes
Yes
Yes
Yes
No
Yes
No
No
Yes
Yes

No
No
Yes
Yes
No
Yes
Yes
Yes
Yes
No
Yes
No
Yes
Yes
No
Yes
No
No
Yes
Yes
No
Yes
No
No
No
No
No
No
No
No
No
No
No
No
No
No
No
No
No
No
No
Yes
No
Yes
Yes
No
Yes

No
No
Yes
No
Yes
Yes
Yes
Yes
Yes
Yes
No
No
Yes
Yes
Yes
No
Yes
No
Yes
Yes
Yes
Yes
Yes
No
No
Yes
Yes
No
No
No
Yes
Yes
No
No
Yes
Yes
No
No
Yes
No
Yes
Yes
Yes
No
No
Yes
No

No
Yes
Yes
Yes
No
No
Yes
Yes
Yes
Yes
Yes
Yes
Yes
Yes
Yes
Yes
No
No
Yes
Yes
Yes
Yes
Yes
Yes
No
No
No
Yes
No
No
No
Yes
No
Yes
Yes
Yes
Yes
Yes
Yes
No
No
Yes
No
No
Yes
No
No

Yes
Yes
Yes
Yes
No
No
No
No
No
No
No
No
No
No
Yes
No
No
Yes
Yes
No
No
Yes
No
No
No
No
No
No
No
Yes
Yes
No
Yes
Yes
No
Yes
Yes
No
No
Yes
No
Yes
Yes
Yes
Yes
Yes
No

No
No
Yes
No
No
No
No
Yes
Yes
No
Yes
No
No
No
Yes
No
Yes
No
No
No
No
No
Yes
Yes
Yes
Yes
No
No
No
No
Yes
No
Yes
No
Yes
No
No
Yes
No
Yes
Yes
No
No
Yes
Yes
No
Yes

No
No
Yes
No
No
Yes
No
No
No
No
No
Yes
No
Yes
No
No
Yes
Yes
Yes
No
Yes
Yes
Yes
Yes
No
No
No
Yes
Yes
No
Yes
Yes
Yes
Yes
Yes
Yes
No
No
No
No
Yes
No
Yes
No
No
No
No

No
Yes
Yes
Yes
No
Yes
No
No
No
Yes
No
Yes
No
Yes
No
No
No
No
No
No
No
No
No
Yes
No
No
Yes
Yes
No
Yes
No
Yes
No
No
No
No
No
Yes
Yes
Yes
No
No
No
Yes
No
Yes
No

No
Yes
No
No
Yes
Yes
No
No
No
Yes
No
No
No
No
Yes
Yes
Yes
Yes
No
No
No
No
Yes
Yes
Yes
No
No
No
No
Yes
Yes
No
No
No
No
Yes
Yes
No
Yes
No
No
Yes
Yes
No
Yes
Yes
No

No
No
No
No
No
Yes
No
Yes
No
Yes
Yes
No
No
No
Yes
No
Yes
No
Yes
Yes
No
No
No
No
No
No
No
No
No
No
No
No
No
No
No
No
No
No
No
No
No
No
No
No
No
No
No

Yes
No
Yes
No
No
Yes
Yes
yes
Yes
No
No
No
No
Yes
No
No
No
Yes
Yes
No
No
No
No
No
Yes
No
No
Yes
Yes
Yes
No
Yes
No
No
Yes
No
No
No
Yes
Yes
No
Yes
No
Yes
Yes
No
Yes

Yes
Yes
No
No
No
No
No
Yes
Yes
No
No
No
Yes
No
Yes
Yes
Yes
Yes
No
No
No
Yes
Yes
No
Yes
No
Yes
No
No
No
No
No
No
Yes
No
Yes
No
Yes
Yes
Yes
No
No
Yes
Yes
No
Yes
No

Yes
Yes
No
No
Yes
Yes
No
No
Yes
No
No
No
Yes
Yes
No
Yes
No
Yes
Yes
No
Yes
No
No
Yes
No
Yes
Yes
No
Yes
No
Yes
No
No
No
No
Yes
No
No
No
No
No
No
No
No
No
Yes
No

No
No
No
No
No
Yes
Yes
Yes
No
No
Yes
Yes
No
No
No
Yes
No
Yes
No
Yes
No
No
Yes
Yes
No
Yes
Yes
No
No
Yes
Yes
No
Yes
Yes
No
No
Yes
Yes
No
No
Yes
No
Yes
No
Yes
No
No

Yes
Yes
Yes
No
No
No
Yes
No
No
No
Yes
Yes
No
Yes
No
Yes
No
No
No
No
Yes
Yes
Yes
No
Yes
Yes
Yes
Yes
Yes
No
Yes
No
No
No
Yes
No
Yes
Yes
No
No
Yes
No
Yes
Yes
Yes
Yes
No

No
No
No
No
No
No
No
No
No
No
No
No
Yes
No
No
No
No
No
No
Yes
No
Yes
No
No
No
No
Yes
Yes
No
Yes
Yes
Yes
Yes
Yes
Yes
No
Yes
Yes
No
No
No
No
Yes
No
No
No
Yes

Yes
Yes
Yes
Yes
Yes
No
Yes
Yes
No
No
Yes
Yes
Yes
No
No
No
No
No
No
Yes
Yes
No
Yes
Yes
No
No
Yes
Yes
No
Yes
No
No
Yes
Yes
Yes
No
No
Yes
Yes
No
Yes
No
Yes
Yes
No
No
No

No
Yes
Yes
No
Yes
No
Yes
No
Yes
Yes
No
Yes
No
No
Yes
Yes
Yes
No
No
Yes
Yes
Yes
No
No
Yes
No
No
Yes
Yes
Yes
Yes
Yes
Yes
Yes
No
Yes
No
Yes
No
Yes
Yes
No
No
Yes
No
No
Yes

Yes
Yes
Yes
Yes
No
Yes
No
Yes
No
Yes
No
Yes
No
No
No
Yes
Yes
Yes
No
Yes
Yes
Yes
Yes
Yes
No
Yes
No
No
No
Yes
No
Yes
Yes
No
Yes
No
No
Yes
No
No
No
Yes
Yes
No
Yes
Yes
No

Yes
No
Yes
No
No
No
Yes
Yes
Yes
Yes
Yes
No
No
Yes
Yes
No
No
No
No
No
Yes
No
Yes
Yes
No
Yes
No
Yes
No
Yes
No
No
Yes
No
No
Yes
No
No
Yes
No
Yes
Yes
No
Yes
Yes
Yes
No

Yes
Yes
Yes
No
No
No
Yes
No
No
Yes
Yes
No
No
No
Yes
No
No
No
No
No
No
Yes
No
No
Yes
No
Yes
No
No
No
Yes
Yes
No
No
Yes
Yes
No
Yes
No
Yes
Yes
No
Yes
Yes
Yes
Yes
Yes

No
No
No
Yes
No
No
No
No
Yes
No
No
Yes
Yes
No
No
No
Yes
Yes
No
Yes
No
Yes
No
No
No
No
Yes
Yes
No
No
No
No
Yes
No
No
No
No
Yes
Yes
No
No
No
Yes
No
No
No
No

Yes
Yes
No
Yes
No
No
No
Yes
No
No
No
No
No
No
No
No
No
No
No
No
No
No
No
No
No
No
No
No
Yes
No
No
Yes
No
Yes
No
Yes
No
No
No
Yes
Yes
No
Yes
No
No
No
No

Yes
No
Yes
No
No
No
Yes
Yes
No
No
No
No
No
Yes
No
No
No
No
Yes
Yes
Yes
No
Yes
No
Yes
No
Yes
No
No
No
Yes
No
No
Yes
No
Yes
No
No
No
No
Yes
No
No
No
Yes
No
Yes

No
No
No
No
No
No
Yes
No
No
Yes
No
Yes
No
No
No
No
No
No
No
No
No
No
No
No
No
Yes
Yes
Yes
No
No
No
No
Yes
Yes
No
No
No
No
Yes
No
Yes
Yes
No
Yes
Yes
Yes
No

Yes
No
No
Yes
No
Yes
Yes
Yes
No
No
No
Yes
No
No
No
Yes
Yes
Yes
No
No
No
Yes
Yes
Yes
No
Yes
Yes
Yes
No
Yes
No
No
Yes
No
Yes
No
Yes
No
No
Yes
No
Yes
No
Yes
Yes
Yes
No

Yes
No
Yes
Yes
No
Yes
No
Yes
Yes
No
No
Yes
Yes
Yes
Yes
Yes
Yes
Yes
Yes
Yes
Yes
No
Yes
No
No
Yes
Yes
No
No
No
No
No
No
No
No
No
Yes
Yes
Yes
Yes
No
No
No
No
No
No
No

Yes
No
No
No
No
Yes
Yes
No
No
Yes
Yes
Yes
Yes
Yes
No
No
No
Yes
Yes
Yes
Yes
No
No
No
Yes
Yes
Yes
Yes
No
Yes
Yes
No
Yes
No
Yes
Yes
Yes
No
No
No
No
No
Yes
No
No
No
No

No
No
Yes
Yes
No
No
No
No
Yes
No
No
No
Yes
Yes
Yes
No
Yes
No
Yes
Yes
No
Yes
No
No
Yes
Yes
Yes
Yes
No
No
No
Yes
Yes
No
No
Yes
No
Yes
No
Yes
Yes
No
Yes
Yes
No
No
Yes

Yes
No
No
Yes
No
No
Yes
Yes
No
No
No
Yes
No
Yes
Yes
Yes
No
No
No
Yes
Yes
Yes
No
Yes
No
Yes
Yes
No
Yes
No
Yes
Yes
No
No
Yes
Yes
Yes
Yes
No
No
Yes
No
No
Yes
No
Yes
No

No
Yes
No
Yes
No
Yes
No
Yes
No
No
Yes
Yes
Yes
Yes
Yes
No
No
Yes
No
Yes
No
Yes
Yes
No
No
Yes
Yes
Yes
No
No
Yes
No
Yes
Yes
Yes
No
Yes
Yes
Yes
No
No
Yes
No
Yes
No
No
Yes

No
Yes
Yes
No
No
Yes
Yes
Yes
No
Yes
No
Yes
No
No
No
No
Yes
Yes
Yes
No
Yes
No
Yes
No
Yes
Yes
Yes
Yes
Yes
No
Yes
No
Yes
Yes
No
No
No
No
No
Yes
Yes
Yes
Yes
Yes
Yes
No
No

Yes
No
No
Yes
Yes
No
No
Yes
Yes
No
Yes
No
Yes
No
No
Yes
No
Yes
No
Yes
Yes
Yes
No
Yes
Yes
No
No
Yes
No
Yes
Yes
Yes
No
Yes
No
Yes
Yes
No
No
No
Yes
No
Yes
No
No
Yes
Yes

Yes
No
Yes
No
Yes
Yes
No
No
No
No
No
No
No
Yes
No
No
No
No
No
No
Yes
Yes
Yes
Yes
No
No
No
No
No
No
No
No
Yes
No
No
No
No
No
No
Yes
Yes
Yes
Yes
No
No
No
No

No
Yes
No
Yes
Yes
No
Yes
No
No
Yes
Yes
Yes
No
No
Yes
Yes
Yes
Yes
Yes
Yes
No
No
Yes
No
No
No
No
No
No
Yes
Yes
No
No
No
Yes
Yes
Yes
Yes
No
Yes
Yes
Yes
No
No
No
No
No

No
No
No
No
No
No
No
No
No
No
No
No
No
No
No
No
No
Yes
Yes
No
No
No
No
No
No
Yes
Yes
No
No
No
Yes
No
Yes
No
No
Yes
Yes
Yes
Yes
No
No
No
Yes
No
Yes
No
Yes

Yes
No
Yes
Yes
No
No
Yes
No
Yes
Yes
No
Yes
Yes
No
Yes
No
Yes
No
Yes
Yes
No
Yes
No
No
Yes
Yes
No
Yes
No
No
No
Yes
Yes
Yes
Yes
No
No
No
Yes
No
Yes
No
No
No
No
No
No

No
No
No
No
Yes
Yes
Yes
No
No
Yes
No
Yes
No
No
No
Yes
Yes
Yes
Yes
No
Yes
No
No
No
No
No
Yes
Yes
Yes
Yes
No
Yes
Yes
No
Yes
Yes
No
Yes
Yes
Yes
No
Yes
No
No
No
No
No

Yes
No
Yes
Yes
No
No
Yes
No
No
Yes
Yes
Yes
Yes
Yes
Yes
No
Yes
No
No
No
No
Yes
No
No
Yes
No
No
No
No
Yes
No
Yes
Yes
Yes
Yes
No
Yes
Yes
No
Yes
Yes
No
No
Yes
No
No
No

No
Yes
Yes
Yes
Yes
No
Yes
No
Yes
Yes
No
No
No
Yes
No
No
No
Yes
No
No
Yes
Yes
No
Yes
Yes
Yes
No
Yes
No
Yes
No
No
No
Yes
Yes
Yes
No
No
No
No
No
Yes
Yes
No
No
Yes
Yes

Yes

No

No

Yes

No

No

Yes

Yes

No

No

Yes

Yes

Yes

No

No

No

No

Yes

No

Yes

Yes

Yes

Yes

No

Yes

Yes

Yes

Yes

No

No

No

Yes

No

No

No

No

Yes

No

No

Yes

Yes

Yes

Yes

Yes

Yes

No

Yes

No
No
No
Yes
Yes
Yes
Yes
Yes
Yes
No
Yes
No
Yes
Yes
No
No
No
No
No
No
No
No
No
No
No
Yes
Yes
Yes
Yes
Yes
No
No
Yes
No
No
No
No
Yes
No
Yes
No
No
Yes
Yes
Yes
No
No

No
Yes
No
No
Yes
No
Yes
Yes
No
Yes
Yes
Yes
No
Yes
No
No
Yes
No
No
No
No
Yes
No
Yes
No
No
Yes
No
No
No
Yes
No
No
Yes
Yes
Yes
No
Yes
Yes
Yes
No
No
Yes
No
Yes
No
No

Yes
No
Yes
Yes
No
Yes
No
Yes
No
No
No
No
No
Yes
Yes
No
Yes
Yes
No
Yes
Yes
Yes
No
No
No
No
No
No
No
No
No
No
No
No
No
No
No
No
No
No
No
No
No
No
No
Yes
No

Yes
No
Yes
Yes
No
Yes
No
Yes
Yes
Yes
Yes
No
No
Yes
No
Yes
No
Yes
Yes
No
No
Yes
Yes
No
No
No
No
Yes
No
Yes
No
Yes
Yes
No
Yes
Yes
Yes
Yes
Yes
Yes
Yes
Yes
No
No
No
No
Yes

No
Yes
Yes
Yes
Yes
Yes
No
Yes
No
Yes
Yes
No
Yes
Yes
No
Yes
Yes
Yes
Yes
No
Yes
Yes
No
No
No
Yes
Yes
No
No
Yes
No
No
No
Yes
No
No
Yes
No
Yes
Yes
Yes
Yes
No
No
Yes
Yes
No

Yes
Yes
Yes
No
Yes
Yes
Yes
Yes
No
Yes
Yes
Yes
No
Yes
No
Yes
No
Yes
No
Yes
No
No
Yes
No
Yes
Yes
No
No
Yes
No
Yes
Yes
No
No
Yes
No
No
No
Yes
Yes
No
Yes
Yes
Yes
Yes
No
Yes

No
No
Yes
No
Yes
Yes
No
Yes
Yes
Yes
Yes
Yes
Yes
Yes
Yes
Yes
Yes
Yes
No
No
No
Yes
Yes
Yes
Yes
Yes
No
No
Yes
No
No
Yes
No
Yes
Yes
Yes
Yes
No
Yes
No
No
No
No
No
Yes
Yes
No

Yes
No
Yes
Yes
Yes
Yes
Yes
Yes
Yes
Yes
No
Yes
No
No
No
No
Yes
Yes
No
Yes
No
No
Yes
No
Yes
No
Yes
No
Yes
No
No
No
Yes
Yes
Yes
Yes
Yes
Yes
No
No
No
No
No
Yes
Yes
No
No

Yes
No
No
Yes
No
Yes
No
Yes
Yes
No
Yes
Yes
No
Yes
No
No
No
No
No
No
No
No
No
No
No
No
No
No
No
No
No
No
No
No
No
No
No
No
Yes
No
No
Yes
No
No
Yes
No
Yes
Yes

Yes
Yes
Yes
Yes
Yes
Yes
No
Yes
Yes
Yes
Yes
No
Yes
Yes
No
Yes
Yes
No
Yes
No
No
No
Yes
No
No
No
Yes
No
No
Yes
No
Yes
Yes
Yes
Yes
Yes
No
No
Yes
Yes
Yes
No
Yes
Yes
Yes
No
Yes

No
No
No
Yes
No
No
Yes
Yes
Yes
Yes
No
No
Yes
Yes
Yes
Yes
Yes
No
Yes
No
Yes
No
No
Yes
Yes
No
Yes
Yes
Yes
Yes
Yes
Yes
No
No
No
Yes
No
Yes
Yes
No
Yes
No
Yes
Yes
Yes
Yes
Yes

Yes
Yes
No
Yes
Yes
Yes
No
Yes
No
Yes
Yes
No
Yes
No
No
No
Yes
No
Yes
Yes
Yes
No
No
No
Yes
No
Yes
Yes
Yes
No
Yes
Yes
No
Yes
No
No
Yes
Yes
No
Yes
Yes
No
Yes
No
Yes
No
No

Yes
Yes
Yes
Yes
Yes
Yes
Yes
Yes
No
Yes
Yes
No
No
Yes
No
Yes
No
No
Yes
Yes
Yes
Yes
No
No
Yes
Yes
Yes
Yes
Yes
No
Yes
Yes
No
Yes
Yes
Yes
Yes
Yes
Yes
Yes
No
Yes
No
Yes
No
Yes
No

No
Yes
No
No
Yes
Yes
Yes
Yes
Yes
Yes
Yes
Yes
Yes
Yes
Yes
Yes
Yes
Yes
Yes
Yes
Yes
Yes
Yes
Yes
Yes
Yes
No
No
Yes
Yes
Yes
Yes
Yes
Yes
Yes
Yes
Yes
Yes
Yes
Yes
Yes
No
Yes
Yes
Yes
No
Yes

No
Yes
Yes
Yes
Yes
No
Yes
Yes
Yes
Yes
No
Yes
Yes
Yes
Yes
Yes
Yes
Yes
Yes
Yes
Yes
Yes
Yes
Yes
Yes
No
Yes
Yes
No
No
Yes
Yes
Yes
No
Yes
Yes
No
Yes
Yes
No
No
Yes
No
No
No
No
No

No
No
No
No
No
No
No
No
No
Yes
Yes
Yes
No
No
No
No
No
No
Yes
Yes
No
Yes
No
Yes
No
No
No
Yes
No
Yes
Yes
Yes
No
No
Yes
No
No
No
No
No
No
No
No
No
No
No
No

No
Yes
No
No
No
Yes
Yes
No
No
No
Yes
No
No
No
No
Yes
No
Yes
Yes
Yes
Yes
No
Yes
No
No
No
No
No
Yes
No
No
No
Yes
No
No
No
No
Yes
Yes
Yes
No
No
Yes
Yes
No
No
No

Yes
No
No
No
No
Yes
No
No
No
No
Yes
Yes
No
Yes
Yes
No
No
No
No
No
No
Yes
Yes
No
Yes
Yes
Yes
No
No
No
No
No
Yes
Yes
Yes
No
Yes
No
Yes
Yes
Yes
Yes
Yes
No
Yes
No
Yes

Yes
No
No
Yes
No
Yes
Yes
Yes
Yes
Yes
Yes
Yes
No
Yes
No
No
No
Yes
No
Yes
No
No
Yes
Yes
Yes
Yes
No
Yes
No
No
Yes
Yes
Yes
No
No
No
No
Yes
Yes
Yes
Yes
No
Yes
No
Yes
Yes
Yes

Yes
Yes
Yes
Yes
No
Yes
Yes
Yes
Yes
Yes
Yes
No
No
Yes
Yes
Yes
Yes
Yes
No
No
Yes
Yes
No
No
Yes
No
No
Yes
No
No
Yes
Yes
No
Yes
Yes
Yes
No
Yes
Yes
Yes
No
Yes
No
No
Yes
No
No

Yes
No
No
Yes
Yes
No
Yes
Yes
Yes
No
Yes
Yes
Yes
Yes
Yes
Yes
Yes
Yes
Yes
No
Yes
Yes
Yes
No
No
No
Yes
No
No
Yes
Yes
No
Yes
Yes
Yes
Yes
No
Yes
Yes
Yes
Yes
Yes
Yes
Yes
Yes
Yes
Yes

Yes
Yes
Yes
Yes
Yes
Yes
Yes
Yes
Yes
Yes
No
No
Yes
No
Yes
Yes
Yes
No
Yes
Yes
Yes
No
No
No
Yes
Yes
Yes
Yes
No
Yes
No
Yes
Yes
Yes
Yes
Yes
Yes
Yes
Yes
Yes
Yes
Yes
Yes
Yes
Yes
Yes
Yes

Yes
Yes
Yes
Yes
No
No
Yes
Yes
Yes
Yes
Yes
Yes
Yes
Yes
Yes
No
Yes
Yes
Yes
No
Yes
No
Yes
Yes
Yes
Yes
Yes
No
Yes
No
Yes
No
Yes
Yes
Yes
Yes
Yes
Yes
Yes
No
No
Yes
No
Yes
Yes
Yes
Yes

No
Yes
Yes
Yes
No
No
Yes
Yes
No
Yes
Yes
Yes
Yes
No
No
Yes
Yes
Yes
Yes
No
Yes
Yes
Yes
No
No
Yes
Yes
Yes
Yes
Yes
Yes
Yes
No
No
No
Yes
Yes
Yes
Yes
Yes
Yes
Yes
No
Yes
Yes
No
Yes

No
Yes
No
Yes
Yes
Yes
Yes
Yes
Yes
No
Yes
Yes
Yes
Yes
Yes
Yes
Yes
Yes
No
Yes
Yes
Yes
Yes
No
Yes
Yes
No
Yes
Yes
Yes
Yes
No
Yes
Yes
Yes
Yes
Yes
Yes
Yes
Yes
Yes
Yes
No
Yes
No
No
Yes

No
Yes
Yes
No
Yes
Yes
Yes
Yes
Yes
No
Yes
No
No
Yes
Yes
Yes
Yes
Yes
Yes
No
Yes
Yes
Yes
Yes
Yes
Yes
No
Yes
No
No
Yes
Yes
Yes
No
Yes
Yes
Yes
No
Yes
Yes
Yes
No
Yes
No
Yes
Yes
Yes

Yes
Yes
No
Yes
No
Yes
Yes
No
Yes
Yes
No
No
Yes
No
No
Yes
No
Yes
No
Yes
No
No
No
No
No
No
No
No
No
No
No
No
Yes
Yes
Yes
Yes
Yes
Yes
No
Yes
Yes
No
Yes
Yes
Yes
Yes
Yes

No
No
No
Yes
No
No
Yes
Yes
Yes
Yes
Yes
Yes
No
No
Yes
No
No
No
Yes
No
Yes
No
Yes
Yes
No
Yes
No
No
Yes
No
No
Yes
No
Yes
Yes
Yes
No
Yes
No
No
Yes
Yes
Yes
Yes
No
Yes
No

No
Yes
Yes
No
No
Yes
Yes
No
Yes
Yes
Yes
Yes
No
Yes
No
Yes
Yes
No
Yes
No
No
Yes
Yes
No
Yes
Yes
Yes
No
No
Yes
Yes
Yes
No
No
Yes
No
Yes
No
Yes
No
Yes
No
Yes
Yes
Yes
No
Yes

Yes
No
Yes
No
No
Yes
No
Yes
Yes
Yes
Yes
Yes
Yes
No
No
Yes
Yes
Yes
No
Yes
No
Yes
Yes
Yes
Yes
Yes
No
No
Yes
Yes
No
No
No
Yes
Yes
No
No
Yes
Yes
No
No
Yes
No
Yes
Yes
Yes
No

No
Yes
No
No
Yes
Yes
Yes
Yes
No
Yes
Yes
No
No
No
No
Yes
No
Yes
Yes
Yes
No
Yes
No
No
No
No
No
No
Yes
No
No
Yes
Yes
No
No
Yes
No
No
No
No
No
No
Yes
No
No
No
No

Yes
Yes
No
Yes
No
No
No
Yes
No
Yes
No
No
No
No
No
Yes
Yes
Yes
Yes
No
No
No
No
Yes
No
Yes
No
Yes
No
No
Yes
No
Yes
Yes
No
No
Yes
Yes
No
Yes
No
No
Yes
No
No
Yes
No

No
No
No
No
Yes
No
Yes
No
No
Yes
Yes
Yes
No
Yes
Yes
Yes
Yes
No
No
No
Yes
Yes
No
Yes
Yes
Yes
Yes
Yes
Yes
No
No
No
No
Yes
No
Yes
No
No
No
No
No
Yes
Yes
Yes
No
Yes
No

No
No
Yes
No
Yes
No
Yes
No
No
No
No
No
No
No
No
No
Yes
No
No
Yes
Yes
No
Yes
No
Yes
No
No
No
No
No
Yes
Yes
Yes
No
No
No
Yes
No
Yes
No
No
Yes
No
No
Yes
Yes
No

No
No
Yes
No
No
No
No
Yes
Yes
Yes
Yes
No
No
No
No
Yes
Yes
Yes
No
No
No
No
Yes
Yes
No
No
No
No
Yes
Yes
No
Yes
No
No
Yes
Yes
No
Yes
Yes
No
No
No
No
No
No
Yes
No

Yes
No
Yes
Yes
No
No
No
Yes
No
Yes
No
Yes
Yes
Yes
No
No
Yes
Yes
No
No
No
No
Yes
Yes
Yes
Yes
No
No
No
No
No
No
Yes
Yes
No
Yes
Yes
No
No
Yes
Yes
No
Yes
No
No
Yes
Yes

yes
Yes
No
No
No
No
Yes
No
No
No
Yes
Yes
No
No
No
No
No
Yes
No
No
Yes
Yes
Yes
No
Yes
No
No
Yes
No
No
No
Yes
Yes
No
Yes
No
Yes
Yes
No
Yes
Yes
Yes
No
No
No
No
No

Yes
Yes
No
No
No
Yes
No
Yes
Yes
Yes
Yes
No
No
No
Yes
Yes
No
Yes
No
Yes
No
No
No
No
No
No
Yes
No
Yes
No
Yes
Yes
Yes
No
No
Yes
Yes
No
Yes
No
Yes
Yes
No
No
Yes
Yes
No

No
Yes
No
No
No
Yes
Yes
No
Yes
No
Yes
Yes
No
No
No
No
No
No
No
Yes
No
No
No
No
No
No
No
No
No
No
No
No
No
No
No
No
No
No
No
No
No
No
No
No
No
No
Yes

Yes
No
No
Yes
Yes
No
Yes
Yes
No
No
Yes
Yes
No
No
Yes
No
Yes
No
Yes
No
No
Yes
Yes
Yes
No
No
No
Yes
No
No
No
Yes
Yes
No
Yes
No
Yes
No
No
No
No
Yes
Yes
Yes
No
Yes
Yes

Yes
Yes
Yes
No
Yes
No
No
No
Yes
No
Yes
Yes
No
No
Yes
No
Yes
Yes
Yes
Yes
No
No
No
No
No
No
No
No
No
No
No
No
No
Yes
No
No
No
No
No
No
Yes
No
Yes
No
No
No
No

Yes
Yes
No
Yes
Yes
Yes
Yes
Yes
Yes
No
Yes
Yes
No
No
No
No
Yes
No
No
No
Yes
Yes
Yes
Yes
Yes
Yes
No
Yes
Yes
No
No
Yes
Yes
Yes
No
No
No
No
No
No
Yes
Yes
No
Yes
Yes
No
No

Yes
Yes
No
Yes
No
No
Yes
Yes
Yes
No
No
Yes
Yes
No
Yes
No
Yes
Yes
No
No
No
Yes
No
Yes
No
No
No
Yes
No
Yes
No
Yes
Yes
No
No
Yes
No
Yes
No
Yes
Yes
No
Yes
Yes
No
Yes
No

Yes
No
Yes
Yes
No
Yes
No
No
Yes
Yes
Yes
No
No
Yes
Yes
Yes
No
No
Yes
No
No
Yes
Yes
Yes
Yes
Yes
Yes
Yes
No
Yes
No
Yes
No
Yes
Yes
No
No
Yes
No
No
Yes
Yes
Yes
Yes
Yes
No
Yes

No
Yes
No
Yes
No
No
No
Yes
Yes
Yes
Yes
No
Yes
Yes
Yes
Yes
Yes
Yes
Yes
Yes
Yes
Yes
Yes
No
Yes
Yes
No
No
No
Yes
Yes
Yes
No
Yes
Yes
Yes
Yes
Yes
No
Yes
No
No
Yes
Yes
Yes
Yes
Yes

Yes
Yes
Yes
Yes
Yes
Yes
Yes
Yes
Yes
Yes
Yes
Yes
Yes
Yes
Yes
Yes
Yes
Yes
Yes
Yes
Yes
Yes
Yes
Yes
Yes
Yes
Yes
Yes
Yes
Yes
Yes
Yes
Yes
Yes
Yes
Yes
Yes
Yes
Yes
Yes
Yes
Yes
Yes
Yes
Yes
Yes
Yes

Yes
Yes
Yes
Yes
Yes
Yes
Yes
Yes
Yes
Yes
Yes
Yes
Yes
Yes
Yes
Yes
Yes
Yes
Yes
Yes
Yes
Yes
Yes
Yes
Yes
Yes
Yes
Yes
Yes
Yes
Yes
Yes
Yes
Yes
Yes
Yes
Yes
Yes
Yes
Yes
Yes
Yes
Yes
Yes
Yes
Yes
Yes

Yes
Yes
Yes
Yes
Yes
Yes
Yes
Yes
Yes
Yes
Yes
Yes
Yes
Yes
Yes
Yes
Yes
Yes
Yes
Yes
Yes
Yes
Yes
Yes
Yes
Yes
Yes
Yes
Yes
Yes
Yes
Yes
Yes
Yes
Yes
Yes
Yes
Yes
Yes
Yes
Yes
Yes
Yes
Yes
Yes
Yes
Yes

Yes
Yes
Yes
Yes
Yes
Yes
Yes
Yes
Yes
Yes
Yes
Yes
Yes
Yes
Yes
Yes
Yes
Yes
Yes
Yes
Yes
Yes
Yes
Yes
Yes
Yes
Yes
Yes
Yes
Yes
Yes
Yes
Yes
Yes
Yes
Yes
Yes
Yes
Yes
No
No
Yes
Yes
Yes
No
No
Yes

No
Yes
Yes
Yes
No
Yes
No
Yes
Yes
No
Yes
No
No
No
No
No
No
Yes
No
Yes
No
No
No
Yes
Yes
Yes
Yes
No
Yes
No
No
No
No
No
Yes
Yes
Yes
Yes
No
Yes
Yes
No
Yes
Yes
No
Yes
Yes

Yes
No
Yes
No
No
No
No
No
Yes
No
Yes
Yes
No
No
Yes
No
No
Yes
Yes
Yes
Yes
Yes
Yes
No
Yes
No
No
No
No
Yes
No
No
Yes
No
No
No
No
Yes
No
Yes
Yes
Yes
Yes
No
Yes
Yes
No

Yes
Yes
No
No
Yes
No
No
No
No
Yes
Yes
Yes
Yes
No
Yes
No
Yes
Yes
No
No
No
Yes
No
No
No
Yes
No
No
Yes
No
No
No
No
No
No
No
No
No
No
No
No
No
No
No
No
No
No

No
No
Yes
Yes
No
No
Yes
Yes
Yes
No
No
Yes
No
No
Yes
Yes
No
No
Yes
Yes
Yes
No
No
No
No
Yes
No
Yes
Yes
Yes
Yes
No
Yes
Yes
Yes
Yes
No
No
No
Yes
No
No
No
No
No
Yes
No

No
Yes
Yes
No
Yes
No
Yes
No
Yes
No
No
No
Yes
No
No
No
No
Yes
No
No
Yes
Yes
Yes
Yes
Yes
Yes
No
Yes
No
No
No
Yes
Yes
Yes
Yes
Yes
Yes
No
Yes
No
Yes
Yes
No
No
No
No
No

No
No
No
No
No
No
Yes
Yes
Yes
Yes
Yes
No
No
Yes
No
No
No
No
Yes
No
Yes
No
No
Yes
Yes
Yes
No
No
No
Yes
No
No
Yes
No
Yes
Yes
No
Yes
Yes
Yes
No
Yes
No
No
Yes
No
No

No
No
Yes
No
Yes
No
No
Yes
No
No
No
Yes
No
No
Yes
Yes
Yes
No
Yes
Yes
Yes
No
No
Yes
No
Yes
No
No
Yes
No
Yes
Yes
No
Yes
No
Yes
No
No
No
No
No
Yes
Yes
No
Yes
Yes
No

Yes
Yes
Yes
Yes
Yes
Yes
No
No
Yes
No
No
Yes
Yes
No
Yes
No
No
Yes
No
Yes
No
No
Yes
No
Yes
No
Yes
No
Yes
No
Yes
Yes
No
Yes
No
Yes
Yes
Yes
Yes
No
No
Yes
No
Yes
No
Yes
Yes

No
No
Yes
Yes
No
No
No
No
Yes
No
Yes
No
Yes
Yes
No
Yes
Yes
Yes
Yes
Yes
Yes
Yes
Yes
No
No
No
No
Yes
No
Yes
No
No
Yes
Yes
Yes
Yes
No
Yes
No
Yes
No
No
Yes
Yes
No
Yes
Yes

Yes
Yes
Yes
Yes
No
Yes
Yes
Yes
Yes
No
Yes
No
Yes
Yes
No
Yes
Yes
No
Yes
Yes
Yes
Yes
No
Yes
Yes
No
No
No
Yes
Yes
No
No
Yes
No
No
No
Yes
No
No
Yes
No
Yes
Yes
Yes
Yes
No
No

Yes
Yes
No
Yes
Yes
Yes
No
Yes
Yes
Yes
Yes
No
Yes
Yes
Yes
No
Yes
No
Yes
No
Yes
No
Yes
No
Yes
Yes
Yes
Yes
No
No
Yes
No
No
No
No
Yes
Yes
Yes
Yes
No
No
No
No
No
No
No
Yes

No
No
No
No
Yes
Yes
No
No
Yes
Yes
Yes
Yes
Yes
No
No
No
Yes
Yes
Yes
Yes
No
No
No
Yes
Yes
Yes
No
No
No
No
No
No
No
No
No
No
No
No
No
No
No
No
No
No
No
No
No

No
No
Yes
No
No
No
No
Yes
No
No
No
Yes
Yes
Yes
No
Yes
No
Yes
Yes
No
Yes
No
No
Yes
Yes
Yes
Yes
No
No
No
Yes
Yes
No
No
Yes
No
Yes
No
Yes
Yes
No
Yes
Yes
No
No
Yes
Yes

No
No
Yes
No
No
Yes
Yes
No
No
No
Yes
No
Yes
Yes
Yes
No
No
No
Yes
Yes
Yes
No
Yes
No
Yes
Yes
No
Yes
No
Yes
Yes
No
No
Yes
Yes
Yes
Yes
No
No
Yes
No
No
Yes
No
Yes
No
No

Yes
No
Yes
No
Yes
No
Yes
No
No
Yes
Yes
Yes
Yes
Yes
No
No
Yes
No
Yes
No
Yes
Yes
No
No
Yes
Yes
No
Yes
Yes
No
Yes
No
Yes
Yes
No
No
No
Yes
Yes
Yes
Yes
No
Yes
No
No
Yes
No

Yes
No
Yes
No
No
No
No
Yes
No
No
Yes
No
Yes
Yes
Yes
No
Yes
Yes
Yes
No
No
Yes
No
Yes
No
No
Yes
No
Yes
Yes
No
No
Yes
Yes
Yes
No
Yes
No
Yes
No
No
No
No
Yes
Yes
Yes
No

Yes
No
Yes
No
Yes
Yes
Yes
Yes
Yes
No
Yes
No
Yes
Yes
No
No
No
No
No
Yes
Yes
Yes
Yes
Yes
Yes
No
No
Yes
No
No
Yes
Yes
No
No
Yes
Yes
No
Yes
No
Yes
No
No
Yes
No
Yes
No
Yes

Yes
Yes
No
Yes
Yes
No
No
Yes
No
Yes
Yes
Yes
No
Yes
No
Yes
Yes
No
No
No
Yes
No
Yes
No
No
Yes
Yes
Yes
No
Yes
No
Yes
Yes
No
No
No
No
No
No
No
Yes
No
No
No
No
No
No

Yes
Yes
Yes
Yes
No
No
No
No
No
No
No
No
Yes
No
No
No
No
No
No
Yes
Yes
Yes
Yes
No
No
No
No
No
Yes
No
Yes
Yes
No
Yes
No
No
No
No
No
Yes
No
Yes
Yes
No
Yes
No
Yes

No
No
Yes
No
No
Yes
Yes
Yes
No
Yes
Yes
Yes
No
No
Yes
Yes
Yes
Yes
Yes
Yes
No
No
Yes
No
No
No
No
No
No
Yes
Yes
No
No
No
Yes
Yes
Yes
Yes
No
No
No
No
No
No
No
No
No

No
No
No
No
No
No
No
No
No
No
No
No
No
No
No
No
No
No
No
No
No
No
No
No
No
No
No
No
No
No
No
No
No
No
No
No
No
No
No
No
No
No
No
No
No
No
No

No
No
No
No
No
No
No
No
No
No
No
No
No
No
No
No
No
No
No
No
No
No
No
No
No
No
No
No
No
No
No
No
No
No
No
No
No
No
No
No
No
No
No
No
No
No
No

No
No
No
No
No
No
No
No
No
No
No
No
No
No
No
No
No
No
No
No
No
No
No
No
No
No
No
No
No
No
No
No
No
No
No
No
No
No
No
No
No
No
No
No
No
No
No

No
No
No
No
No
No
No
No
No
No
No
No
No
No
No
No
No
No
No
No
No
No
No
No
No
No
No
No
No
No
No
No
No
No
No
No
No
No
No
No
No
No
No
No
No
No
No

No
No
No
No
No
No
No
No
No
No
No
No
No
No
No
No
No
No
No
No
No
No
No
No
No
No
No
No
No
No
No
No
No
No
No
No
No
No
No
No
No
No
No
No
No
No
No

No
No
No
No
No
No
No
No
No
No
No
No
No
No
No
No
No
No
No
No
No
No
No
No
No
No
No
No
No
No
No
No
No
No
No
No
No
No
No
No
No
No
No
No
No
No
No

No
No
No
No
No
No
No
No
No
No
No
No
No
No
No
No
No
No
No
No
No
No
No
No
No
No
No
No
No
Yes
No
Yes
No
Yes
No
No
No
Yes
Yes
Yes
Yes
No
Yes
Yes
No
Yes
Yes

No
Yes
No
No
Yes
No
No
No
No
No
Yes
No
Yes
No
No
Yes
Yes
Yes
No
Yes
Yes
Yes
Yes
No
No
No
Yes
Yes
No
Yes
Yes
Yes
Yes
Yes
Yes
No
No
No
No
Yes
No
Yes
No
No
No
No
No

Yes
Yes
Yes
No
Yes
No
No
No
Yes
No
Yes
No
Yes
No
No
No
No
No
No
No
No
No
Yes
No
No
Yes
Yes
No
Yes
No
Yes
No
No
No
No
No
Yes
Yes
Yes
No
No
No
Yes
No
Yes
No
No

Yes
No
No
Yes
Yes
No
No
No
Yes
No
No
No
No
Yes
Yes
Yes
Yes
No
No
No
No
Yes
Yes
Yes
No
No
No
No
Yes
Yes
No
No
No
No
Yes
Yes
No
Yes
No
No
Yes
Yes
No
Yes
Yes
No
No

No
No
No
No
Yes
No
Yes
No
Yes
Yes
No
No
No
Yes
No
Yes
No
Yes
Yes
Yes
No
No
Yes
Yes
No
No
No
No
Yes
Yes
Yes
Yes
No
No
No
No
No
No
Yes
No
No
Yes
Yes
No
No
Yes
Yes

No
Yes
No
No
Yes
Yes
yes
Yes
No
No
No
No
Yes
No
No
No
Yes
Yes
No
No
No
No
No
Yes
No
No
Yes
Yes
Yes
No
Yes
No
No
Yes
No
No
No
Yes
Yes
No
Yes
Yes
Yes
Yes

58	Male	153	57	121	91	Yes	Occasional	Regular Exe	Yes
62	Male	154	58	148	91	No	Occasional	No Exercise	No
41	Male	165	70	145	89	No	Occasional	Regular Exe	Yes
45	Female	180	75	101	78	Yes	Occasional	Regular Exe	Yes
46	Male	179	79	108	82	Yes	Not Follow	Regular Exe	Yes
66	Female	142	83	132	81	No	Not Follow	Regular Exe	Yes
54	Male	140	52	112	88	Yes	Following	No Exercise	Yes
62	Female	165	65	120	75	Yes	Not Follow	Regular Exe	No
63	Female	167	67	102	95	Yes	Following	No Exercise	Yes
60	Male	156	60	121	74	Yes	Occasional	No Exercise	Yes
61	Female	155	67	120	88	No	Occasional	Regular Exe	Yes
60	Female	150	80	119	83	Yes	Occasional	No Exercise	No
51	Female	178	81	123	88	Yes	Occasional	Regular Exe	Yes
49	Female	179	83	98	95	Yes	Occasional	No Exercise	Yes
61	Female	145	60	142	90	Yes	Occasional	Occasional	Yes
43	Female	146	80	130	80	No	Following	Occasional	Yes
57	Female	140	65	132	78	Yes	Occasional	No Exercise	Yes
66	Female	140	59	96	89	Yes	Occasional	No Exercise	No
39	Female	152	57	120	81	No	Not Follow	Regular Exe	Yes
36	Female	150	59	115	95	Yes	Not Follow	Regular Exe	During Pre
54	Male	165	69	114	66	Yes	Not Follow	Occasional	Yes
45	Male	166	66	129	89	No	Not Follow	Regular Exe	Yes
53	Male	175	90	130	78	Yes	Following	No Exercise	Yes
59	Male	171	73	101	82	No	Not Follow	No Exercise	Yes
57	Female	172	75	105	92	No	Not Follow	Regular Exe	Yes
57	Female	143	76	140	79	Yes	Following	Regular Exe	No
35	Male	147	56	152	80	No	Not Follow	No Exercise	Yes
58	Male	149	89	145	86	Yes	Following	No Exercise	Yes
57	Male	156	89	100	78	Yes	Following	Regular Exe	Yes
48	Male	155	65	122	80	No	Following	Regular Exe	No
45	Female	154	65	102	88	No	Following	Regular Exe	Yes
56	Male	152	62	101	90	No	Not Follow	No Exercise	Yes
62	Female	151	69	145	92	No	Not Follow	Occasional	Yes
71	Female	153	89	109	71	Yes	Not Follow	No Exercise	Yes
47	Male	158	71	120	95	No	Not Follow	Regular Exe	Yes
57	Male	175	81	125	90	Yes	Not Follow	Regular Exe	No
59	Male	180	83	142	85	Yes	Not Follow	Regular Exe	Yes
58	Male	170	83	97	88	Yes	Not Follow	Regular Exe	No
35	Male	174	84	128	89	No	Not Follow	Regular Exe	Yes
54	Male	176	84	120	75	No	Not Follow	Regular Exe	Yes
65	Female	179	81	148	84	Yes	Following	Occasional	Yes
67	Male	174	82	145	95	No	Occasional	No Exercise	No
63	Female	167	85	109	90	Yes	Following	Regular Exe	Yes
48	Male	168	66	152	90	Yes	Occasional	No Exercise	Yes
58	Female	156	79	108	84	No	Following	Regular Exe	Yes
45	Male	159	52	128	88	Yes	Following	No Exercise	Yes

59	Female	180	55	121	82	Yes	Occasional	Regular Ex	Yes
54	Male	174	56	111	66	Yes	Occasional	No Exercis	No
64	Female	173	60	105	89	No	Following	Occasional	Yes
53	Male	156	60	122	78	Yes	Occasional	Regular Ex	Yes
57	Female	154	61	149	82	Yes	Occasional	No Exercis	Yes
56	Female	154	57	110	92	No	Following	No Exercis	No
42	Male	157	57	135	79	Yes	Occasional	Occasional	Yes
71	Female	159	58	101	80	Yes	Not Follow	Regular Ex	Yes
48	Male	140	70	145	86	Yes	Not Follow	No Exercis	Yes
49	Female	152	75	147	78	No	Following	No Exercis	Yes
52	Female	155	79	145	80	Yes	Following	No Exercis	Yes
62	Female	145	83	145	88	Yes	Not Follow	No Exercis	No
65	Female	146	52	152	75	Yes	Following	Regular Ex	Yes
56	Female	165	65	121	92	Yes	Following	No Exercis	No
44	Male	146	67	151	78	No	Occasional	Regular Ex	No
66	Female	165	68	121	78	No	Occasional	Regular Ex	Yes
52	Male	148	66	99	78	No	Occasional	No Exercis	Yes
52	Female	153	67	95	89	Yes	Following	Regular Ex	Yes
56	Male	154	60	111	88	No	Not Follow	Regular Ex	No
64	Female	165	66	108	66	Yes	Following	Regular Ex	Yes
70	Female	166	67	121	80	Yes	Following	No Exercis	Yes
52	Male	177	64	102	80	Yes	Not Follow	Regular Ex	No
55	Male	179	72	152	98	No	Not Follow	Regular Ex	Yes
56	Male	145	73	121	80	No	Not Follow	Regular Ex	Yes
46	Female	165	59	142	90	Yes	Not Follow	Regular Ex	Yes
42	Female	167	81	131	88	No	Not Follow	No Exercis	No
37	Female	156	82	148	67	No	Not Follow	No Exercis	Yes
50	Female	155	85	143	88	Yes	Not Follow	No Exercis	Yes
58	Male	176	66	129	80	No	Not Follow	Regular Ex	No
57	Male	178	86	108	71	No	Following	No Exercis	Yes
52	Male	179	52	135	68	Yes	Following	Regular Ex	Yes
58	Female	145	55	120	95	No	Following	No Exercis	Yes
60	Female	146	56	140	85	Yes	Following	Regular Ex	No
65	Female	165	60	150	78	No	Not Follow	No Exercis	Yes
51	Male	155	50	93	74	Yes	Not Follow	Occasional	No
56	Female	176	53	92	89	No	Following	Regular Ex	Yes
59	Male	178	54	100	78	Yes	Not Follow	No Exercis	Yes
62	Male	179	59	110	68	Yes	Not Follow	Regular Ex	No
71	Female	145	76	102	99	Yes	Following	Regular Ex	Yes
59	Female	146	71	104	88	No	Following	No Exercis	Yes
66	Female	165	68	106	77	Yes	Not Follow	No Exercis	No
51	Male	155	79	120	80	No	Occasional	No Exercis	No
62	Female	152	85	121	94	Yes	Following	Regular Ex	Yes
61	Female	150	88	135	90	Yes	Not Follow	No Exercis	Yes
64	Male	151	87	142	78	Yes	Following	Regular Ex	No
51	Male	145	84	152	80	Yes	Not Follow	No Exercis	Yes
44	Male	149	82	150	90	Yes	Not Follow	Regular Ex	Yes

68	Female	145	65	142	67	Yes	Following	No Exercise	No
62	Female	148	68	124	80	Yes	Following	Regular Ex	Yes
41	Male	152	76	132	92	No	Not Follow	No Exercise	Yes
53	Male	157	87	134	95	Yes	Not Follow	No Exercise	No
67	Female	156	90	129	99	Yes	Following	Regular Ex	Yes
69	Female	178	79	120	92	Yes	Following	Regular Ex	Yes
37	Female	169	78	121	85	No	Not Follow	Regular Ex	No
68	Female	168	79	130	101	Yes	Not Follow	Regular Ex	No
58	Female	179	80	131	88	No	Not Follow	Regular Ex	Yes
46	Male	172	82	129	85	Yes	Following	No Exercise	Yes
78	Female	168	61	98	94	Yes	Not Follow	Occasional	Yes
78	Female	175	93	99	94	Not Known	Occasional	Occasional	No
78	Female	175	86	127	92	Not Known	Not Follow	No Exercise	No
78	Female	167	85	128	93	Yes	Following	Regular Ex	No
78	Female	169	80	130	91	Not Known	Occasional	No Exercise	Yes
78	Female	162	67	131	96	No	Not Follow	Occasional	No
78	Female	173	83	137	95	Not Known	Following	No Exercise	Yes
78	Female	152	60	139	94	Yes	Following	Regular Ex	No
78	Female	181	75	141	75	Not Known	Not Follow	No Exercise	Yes
78	Female	164	75	90	99	No	Following	Occasional	No
78	Female	177	90	99	85	Not Known	Not Follow	Regular Ex	No
78	Female	180	78	95	88	No	Occasional	No Exercise	Yes
78	Female	169	82	120	94	Not Known	Occasional	Occasional	Yes
79	Female	173	74	123	95	No	Not Follow	No Exercise	No
79	Female	170	87	127	80	No	Following	No Exercise	No
79	Female	170	90	128	80	Yes	Following	No Exercise	No
79	Female	179	77	129	80	No	Occasional	No Exercise	Yes
79	Female	162	55	131	92	Not Known	Not Follow	Regular Ex	Yes
79	Male	155	76	132	94	Not Known	Occasional	Regular Ex	Yes
79	Female	171	96	132	95	Yes	Occasional	Occasional	Yes
35	Female	145	73	125	91	No	Not Follow	Regular Ex	Yes
61	Female	146	69	129	88	No	Following	No Exercise	Yes
59	Male	165	66	132	84	No	Following	Regular Ex	No
63	Female	155	86	142	78	No	Following	No Exercise	Yes
57	Male	152	81	145	90	Yes	Not Follow	Regular Ex	Yes
61	Male	150	83	102	74	No	Occasional	No Exercise	No
60	Female	151	69	110	77	Yes	Occasional	Regular Ex	Yes
39	Male	145	77	108	75	No	Occasional	Regular Ex	Yes
67	Male	149	71	128	85	Yes	Following	No Exercise	Yes
44	Female	145	71	129	87	No	Occasional	No Exercise	During Pre
57	Female	148	67	135	91	Yes	Occasional	Regular Ex	Yes
55	Female	152	68	138	85	Yes	Following	Regular Ex	Yes
60	Male	157	67	140	94	Yes	Following	No Exercise	No
45	Female	156	64	142	95	Yes	Occasional	No Exercise	Yes
88	Female	178	72	148	83	No	Following	No Exercise	Yes
45	Male	173	73	120	92	No	Not Follow	No Exercise	Yes
71	Female	162	59	110	88	Yes	Following	Regular Ex	No

53	Male	181	81	120	90	No	Not Follow	No Exercise	Yes
50	Male	181	82	123	94	No	Not Follow	Regular Ex	Yes
43	Male	177	85	125	90	No	Following	Regular Ex	No
66	Female	170	88	144	78	No	Following	Regular Ex	Yes
63	Female	179	83	105	82	No	Occasional	No Exercise	Yes
54	Male	139	90	124	81	Yes	Following	No Exercise	Yes
61	Female	165	90	112	88	Yes	Following	Regular Ex	Yes
48	Male	167	90	140	75	Yes	Following	No Exercise	No
58	Male	156	90	152	95	Yes	Occasional	Regular Ex	Yes
61	Male	155	90	112	74	Yes	Not Follow	Regular Ex	No
63	Female	160	90	114	92	No	Occasional	Regular Ex	Yes
51	Male	178	67	118	83	No	Occasional	No Exercise	Yes
55	Female	179	87	132	88	Yes	Occasional	No Exercise	No
58	Male	145	120	108	95	Yes	Occasional	Occasional	Yes
57	Male	146	67	132	92	Yes	Not Follow	No Exercise	Yes
64	Female	165	120	145	80	Yes	Occasional	Regular Ex	Yes
77	Male	155	81	149	78	Yes	Not Follow	Regular Ex	Yes
44	Male	152	65	99	89	No	Not Follow	Regular Ex	Yes
44	Male	150	120	90	81	No	Occasional	No Exercise	No
53	Male	151	120	111	88	Yes	Occasional	Occasional	Yes
69	Male	145	60	134	95	Yes	Not Follow	Regular Ex	Yes
62	Female	149	120	104	92	Yes	Occasional	Regular Ex	Yes
58	Male	145	54	149	80	Yes	Occasional	No Exercise	No
76	Female	148	51	121	78	Yes	Occasional	Regular Ex	Yes
54	Male	152	55	107	89	No	Not Follow	No Exercise	Yes
57	Male	157	51	120	81	No	Not Follow	Regular Ex	Yes
66	Female	156	56	118	95	Yes	Not Follow	Regular Ex	Yes
68	Male	170	78	142	71	Yes	Occasional	No Exercise	Yes
67	Male	180	71	121	98	Yes	Occasional	Regular Ex	No
41	Male	179	76	140	80	Yes	Occasional	No Exercise	Yes
43	Male	180	77	132	78	Yes	Occasional	Regular Ex	No
58	Male	174	65	120	95	No	Occasional	No Exercise	Yes
51	Male	180	67	135	88	No	Not Follow	No Exercise	Yes
54	Male	145	71	123	94	Yes	Occasional	Occasional	No
66	Female	156	72	101	72	Yes	Not Follow	No Exercise	Yes
51	Female	176	77	141	90	Yes	Not Follow	Regular Ex	Yes
70	Male	170	79	147	87	Yes	Occasional	Regular Ex	Yes
67	Male	179	75	132	95	Yes	Occasional	Regular Ex	Yes
54	Female	170	90	147	87	No	Following	Regular Ex	Yes
50	Male	155	56	149	80	No	Occasional	No Exercise	No
47	Male	159	74	118	84	Yes	Not Follow	No Exercise	Yes
55	Female	165	90	147	71	Yes	Following	Regular Ex	Yes
43	Male	176	61	120	95	Yes	Following	No Exercise	Yes
55	Male	178	65	120	78	Yes	Not Follow	Regular Ex	No
65	Female	165	90	114	75	Yes	Following	No Exercise	Yes
67	Male	153	49	149	77	No	Not Follow	Regular Ex	Yes
35	Male	153	49	138	92	No	Not Follow	Regular Ex	Yes

76	Female	155	90	121	88	Yes	Following	Regular Ex	Yes
58	Male	156	51	131	85	Yes	Not Follow	No Exercis	Yes
52	Female	157	90	138	82	Yes	Following	Regular Ex	No
60	Female	156	90	120	90	Yes	Following	No Exercis	Yes
66	Male	157	56	121	90	Yes	Not Follow	Regular Ex	No
77	Female	158	71	110	84	No	Not Follow	Regular Ex	Yes
56	Male	159	77	126	88	No	Not Follow	Regular Ex	Yes
56	Male	161	76	121	82	No	Not Follow	Regular Ex	No
54	Male	165	69	114	66	Yes	Not Follow	Occasional	Yes
45	Male	166	66	129	89	No	Not Follow	Regular Ex	Yes
53	Male	175	90	130	78	Yes	Following	No Exercis	Yes
59	Male	171	73	101	82	No	Not Follow	No Exercis	Yes
56	Female	153	68	142	78	Yes	Following	Occasional	Yes
58	Female	155	79	122	86	Yes	Following	No Exercis	Yes
64	Female	156	85	104	88	Yes	Following	Regular Ex	Yes
67	Female	157	88	120	88	No	Following	Regular Ex	No
64	Male	156	87	108	88	No	Not Follow	Regular Ex	Yes
56	Male	157	84	144	70	No	Occasional	No Exercis	Yes
59	Male	158	82	105	74	No	Not Follow	Regular Ex	No
65	Male	159	65	124	92	No	Not Follow	Regular Ex	Yes
56	Male	161	68	112	87	Yes	Occasional	Occasional	Yes
56	Female	165	76	140	87	Yes	Occasional	Regular Ex	Yes
63	Male	166	87	152	66	No	Following	No Exercis	No
56	Male	176	90	112	66	Yes	Following	No Exercis	Yes
58	Female	171	79	114	80	Yes	Not Follow	No Exercis	Yes
63	Female	172	78	118	80	No	Occasional	Regular Ex	No
60	Male	143	79	132	89	No	Occasional	No Exercis	Yes
59	Male	147	77	108	82	Yes	Following	Occasional	Yes
49	Male	149	85	132	78	No	Occasional	Occasional	Yes
64	Female	156	87	132	80	Yes	Following	Regular Ex	No
61	Female	155	90	120	90	No	Occasional	No Exercis	Yes
47	Male	154	83	125	82	Yes	Following	Regular Ex	Yes
44	Female	152	76	110	80	Yes	Occasional	No Exercis	No
41	Male	151	80	134	80	Yes	Not Follow	Regular Ex	Yes
50	Female	166	67	120	68	Yes	Occasional	Regular Ex	Yes
41	Male	176	76	101	73	Yes	Occasional	Regular Ex	Yes
59	Male	171	68	142	85	Yes	Occasional	Occasional	No
41	Female	172	69	120	88	Yes	Following	Regular Ex	Yes
51	Female	143	64	140	88	No	Occasional	No Exercis	No
58	Female	147	92	120	78	Yes	Following	No Exercis	Yes
59	Male	149	90	104	85	Yes	Not Follow	No Exercis	Yes
59	Female	156	89	142	66	No	Following	Regular Ex	No
57	Female	155	88	121	87	Yes	Not Follow	Regular Ex	Yes
61	Female	154	79	99	75	Yes	Following	No Exercis	Yes
56	Male	152	94	118	78	No	Not Follow	Regular Ex	Yes
56	Male	151	79	120	88	Yes	Following	Occasional	Yes
41	Male	153	81	148	80	No	Following	Regular Ex	Yes

60	Male	158	82	119	87	Yes	Occasional	No Exercise	No
51	Male	169	76	101	84	Yes	Occasional	Regular Exe	Yes
61	Male	177	78	139	90	Yes	Not Follow	Occasional	Yes
57	Male	178	75	118	82	Yes	Occasional	Regular Exe	Yes
60	Female	156	73	105	74	Yes	Not Follow	No Exercise	No
59	Male	176	83	121	80	No	Not Follow	Occasional	Yes
55	Male	167	84	149	88	Yes	Not Follow	No Exercise	Yes
68	Female	166	81	108	81	No	Occasional	Occasional	Yes
69	Female	168	82	132	90	Not Known	Occasional	No Exercise	No
45	Male	157	56	112	91	Yes	Not Follow	No Exercise	No
51	Female	159	51	120	91	No	Following	No Exercise	Yes
48	Male	140	65	102	89	Yes	Not Follow	Regular Exe	Yes
59	Female	152	65	121	78	Yes	Occasional	Regular Exe	No
47	Male	155	62	120	82	No	Not Follow	No Exercise	Yes
46	Male	145	69	119	81	Yes	Not Follow	Regular Exe	Yes
58	Female	146	70	123	88	No	Occasional	No Exercise	No
58	Female	165	71	98	75	Yes	Occasional	Occasional	Yes
61	Male	148	81	142	95	Yes	Following	No Exercise	No
56	Female	153	83	130	74	Yes	Following	Occasional	Yes
66	Female	154	83	132	92	Yes	Following	Regular Exe	Yes
52	Female	165	84	96	83	Yes	Following	Regular Exe	No
62	Female	177	77	120	88	Yes	Following	No Exercise	Yes
58	Male	178	78	115	95	No	Not Follow	No Exercise	Yes
39	Male	179	77	135	92	Yes	Not Follow	No Exercise	No
59	Female	139	71	98	80	Yes	Following	Regular Exe	Yes
64	Male	165	77	121	78	No	Not Follow	Regular Exe	No
51	Female	167	75	131	89	Yes	Following	No Exercise	Yes
66	Male	156	73	101	81	No	Not Follow	Regular Exe	Yes
65	Male	155	69	135	95	Yes	Not Follow	Occasional	No
44	Male	155	66	114	73	Yes	Not Follow	Regular Exe	Yes
51	Male	145	54	114	81	Yes	Following	Occasional	No
64	Male	146	58	101	88	Yes	Following	Regular Exe	Yes
65	Female	165	56	145	78	Yes	Following	Regular Exe	Yes
52	Female	148	67	135	81	Yes	Following	No Exercise	No
71	Male	153	65	110	85	Yes	Not Follow	Occasional	Yes
65	Female	154	68	104	71	No	Not Follow	No Exercise	Yes
67	Male	165	69	119	92	Yes	Not Follow	No Exercise	No
54	Female	177	73	152	75	Yes	Following	Regular Exe	Yes
41	Male	178	77	117	89	No	Not Follow	Regular Exe	No
29	Female	179	79	139	85	Yes	Following	No Exercise	Yes
44	Male	138	74	138	66	No	Not Follow	Regular Exe	Yes
53	Female	165	55	101	77	Yes	Not Follow	Occasional	Yes
61	Male	167	54	117	90	Yes	Not Follow	Regular Exe	Yes
59	Male	156	50	123	90	Yes	Not Follow	No Exercise	Yes
65	Female	155	51	135	84	Yes	Not Follow	Occasional	Yes
54	Female	176	55	101	88	Yes	Following	No Exercise	Yes
45	Male	178	52	107	82	Yes	Following	Regular Exe	No

46	Female	179	75	123	66	No	Not Follow	Regular Exe	Yes
62	Female	145	65	148	68	Yes	Following	No Exercise	Yes
53	Male	146	67	118	78	Yes	Not Follow	No Exercise	No
59	Male	165	92	132	82	No	Following	Regular Exe	Yes
71	Female	155	88	109	92	Yes	Following	No Exercise	Yes
71	Female	166	84	145	80	No	Not Follow	Regular Exe	Yes
56	Male	178	67	152	78	Yes	Following	No Exercise	Yes
52	Female	179	66	132	95	Yes	Not Follow	Regular Exe	No
58	Female	142	63	120	88	No	Not Follow	Regular Exe	Yes
52	Female	165	58	122	94	Yes	Following	No Exercise	No
64	Female	167	56	118	72	No	Not Follow	No Exercise	Yes
60	Female	156	67	105	94	Yes	Following	Regular Exe	Yes
59	Female	155	58	120	87	Yes	Following	No Exercise	Yes
77	Female	176	61	123	95	No	Not Follow	Regular Exe	No
46	Male	178	63	126	87	Yes	Not Follow	No Exercise	Yes
58	Male	179	67	127	80	No	Not Follow	Regular Exe	Yes
60	Female	145	78	128	84	Yes	Following	Regular Exe	No
50	Female	146	79	120	71	No	Not Follow	No Exercise	Yes
53	Male	165	92	123	95	No	Following	Regular Exe	Yes
58	Male	155	54	124	78	Yes	Occasional	No Exercise	Yes
58	Male	152	55	125	75	Yes	Not Follow	Regular Exe	No
57	Male	150	59	124	77	Yes	Following	No Exercise	Yes
50	Male	151	61	120	82	No	Not Follow	Regular Exe	No
46	Male	145	61	90	88	No	Not Follow	No Exercise	Yes
54	Male	149	89	93	85	Yes	Following	Occasional	Yes
66	Male	145	89	92	95	No	Not Follow	Regular Exe	Yes
57	Male	148	90	94	90	Yes	Following	No Exercise	No
48	Male	152	60	99	90	No	Not Follow	Regular Exe	Yes
60	Male	157	66	100	84	Yes	Following	Regular Exe	Yes
55	Male	156	64	124	88	Yes	Not Follow	No Exercise	No
59	Male	178	67	125	82	No	Not Follow	Regular Exe	Yes
52	Male	166	61	95	66	No	Not Follow	No Exercise	Yes
51	Male	177	62	121	89	No	Not Follow	Regular Exe	Yes
55	Male	168	67	130	95	No	Following	No Exercise	Yes
41	Female	179	63	145	77	No	Not Follow	Regular Exe	Yes
46	Female	177	59	134	87	Yes	Following	No Exercise	No
57	Female	176	61	134	82	No	Following	No Exercise	Yes
57	Female	177	76	128	92	Yes	Following	Regular Exe	Yes
55	Female	178	72	132	88	No	Not Follow	No Exercise	Yes
60	Female	179	89	120	66	Yes	Following	Regular Exe	No
49	Female	174	90	120	89	No	Not Follow	No Exercise	Yes
51	Male	167	88	101	78	No	Not Follow	No Exercise	Yes
57	Male	168	68	142	88	Yes	Following	Regular Exe	No
61	Male	156	76	120	95	Yes	Following	Regular Exe	Yes
55	Female	159	59	140	80	No	Not Follow	No Exercise	Yes
66	Male	180	80	120	98	Yes	Following	Regular Exe	Yes
56	Male	174	81	104	91	Yes	Not Follow	Regular Exe	No

48	Male	173	67	142	68	Yes	Not Follow	No Exercis	Yes
56	Male	156	76	121	90	Yes	Following	Regular Ex	Yes
63	Male	154	68	99	70	Yes	Following	Regular Ex	No
55	Female	154	69	118	75	No	Not Follow	No Exercis	Yes
51	Female	157	64	120	85	No	Following	Regular Ex	Yes
59	Female	159	92	148	69	No	Following	No Exercis	Yes
52	Female	140	90	119	75	No	Following	Regular Ex	No
64	Male	152	89	101	81	No	Following	Regular Ex	Yes
57	Male	155	88	139	80	Yes	Following	Regular Ex	Yes
58	Female	145	79	118	77	No	Following	Regular Ex	No
42	Male	146	94	105	92	No	Not Follow	No Exercis	Yes
67	Male	165	79	121	82	Yes	Following	Regular Ex	Yes
58	Male	148	81	149	78	Yes	Not Follow	No Exercis	Yes
69	Male	153	82	114	82	No	Not Follow	Regular Ex	No
66	Male	154	76	114	86	Yes	Following	No Exercis	Yes
65	Female	173	55	102	95	No	Not Follow	No Exercis	Yes
51	Female	156	52	128	92	Yes	Not Follow	Regular Ex	Yes
46	Female	154	51	133	80	No	Not Follow	Regular Ex	Yes
51	Male	154	65	122	78	Yes	Not Follow	Regular Ex	Yes
52	Male	157	67	104	89	Yes	Not Follow	Regular Ex	Yes
58	Female	159	92	120	81	Yes	Following	No Exercis	Yes
41	Male	140	88	108	95	Yes	Not Follow	No Exercis	No
43	Female	152	82	144	71	Yes	Not Follow	Regular Ex	Yes
67	Female	155	81	105	99	No	Not Follow	No Exercis	No
58	Male	145	81	124	80	Yes	Following	No Exercis	Yes
62	Female	146	84	112	78	Yes	Not Follow	Regular Ex	Yes
44	Male	165	67	140	95	Yes	Following	No Exercis	No
60	Male	148	78	152	88	Yes	Following	No Exercis	No
60	Male	153	77	112	94	No	Following	Regular Ex	Yes
57	Female	154	75	114	72	Yes	Not Follow	Regular Ex	Yes
53	Male	165	75	118	94	No	Following	Regular Ex	Yes
56	Female	168	67	132	87	Yes	Not Follow	Regular Ex	Yes
62	Male	177	65	108	95	Yes	Following	Regular Ex	Yes
61	Male	179	80	132	87	Yes	Following	No Exercis	Yes
62	Female	142	81	132	80	Yes	Not Follow	Occasional	Yes
34	Female	165	82	120	84	Yes	Following	No Exercis	No
48	Female	167	69	135	71	No	Not Follow	Occasional	Yes
65	Female	156	65	123	95	Yes	Occasional	No Exercis	No
52	Female	155	62	101	78	Yes	Not Follow	Regular Ex	Yes
56	Female	176	61	141	75	Yes	Not Follow	No Exercis	Yes
51	Female	168	68	114	92	Yes	Not Follow	Regular Ex	Yes
50	Female	156	61	129	79	Yes	Following	No Exercis	Yes
63	Female	159	60	130	80	No	Not Follow	Regular Ex	No
57	Male	180	59	101	86	Yes	Following	No Exercis	Yes
44	Male	174	54	105	69	No	Following	Regular Ex	No
54	Female	173	51	98	80	Yes	Not Follow	Occasional	Yes
41	Male	156	55	152	88	Yes	Not Follow	Regular Ex	Yes

40	Male	154	51	145	90	Yes	Following	Regular Ex	No
61	Female	154	56	100	92	Yes	Occasional	Regular Ex	Yes
57	Female	157	46	122	98	Yes	Following	Regular Ex	No
51	Male	159	70	102	95	No	Occasional	Occasional	Yes
55	Female	140	76	101	90	Yes	Occasional	Regular Ex	Yes
47	Male	152	77	145	85	Yes	Occasional	Regular Ex	No
53	Female	155	65	109	88	Yes	Following	Regular Ex	Yes
63	Female	145	67	120	89	Yes	Not Follow	Occasional	No
59	Female	146	71	125	75	No	Following	Regular Ex	Yes
56	Male	165	72	142	77	Yes	Not Follow	Regular Ex	Yes
55	Female	148	77	97	90	No	Not Follow	Regular Ex	No
51	Female	153	79	128	88	Yes	Not Follow	No Exercis	Yes
57	Male	154	75	120	89	Yes	Not Follow	Regular Ex	No
55	Female	159	71	134	70	No	Not Follow	Regular Ex	No
63	Male	161	72	135	74	Yes	Not Follow	No Exercis	No
62	Male	165	77	135	92	Yes	Following	Regular Ex	Yes
60	Male	166	66	135	87	Yes	Not Follow	No Exercis	Yes
51	Male	176	54	136	87	Yes	Not Follow	Regular Ex	Yes
57	Male	171	58	136	66	No	Following	No Exercis	Yes
39	Female	172	56	137	66	Yes	Not Follow	Regular Ex	Yes
59	Male	143	67	137	80	Yes	Following	No Exercis	No
42	Female	147	65	137	80	Yes	Not Follow	Regular Ex	Yes
39	Female	149	68	137	92	Yes	Following	No Exercis	Yes
46	Female	156	69	137	82	Yes	Not Follow	Regular Ex	Yes
67	Female	155	73	137	78	Yes	Not Follow	No Exercis	Yes
60	Male	154	77	137	70	No	Following	Regular Ex	Yes
67	Male	152	79	138	90	Yes	Following	No Exercis	No
67	Male	151	61	138	77	No	Not Follow	Regular Ex	No
77	Female	153	55	138	80	Yes	Following	No Exercis	Yes
58	Female	158	54	138	80	Yes	Following	Regular Ex	Yes
71	Female	166	50	139	91	Yes	Not Follow	Regular Ex	Yes
48	Male	169	51	140	81	Yes	Not Follow	Regular Ex	Yes
52	Female	180	55	140	66	No	Following	No Exercis	Yes
35	Male	177	52	144	95	Yes	Not Follow	No Exercis	No
70	Female	176	51	140	77	Yes	Following	No Exercis	Yes
48	Male	175	67	138	69	Yes	Not Follow	No Exercis	No
47	Male	174	71	145	92	Yes	Not Follow	Regular Ex	Yes
49	Female	165	72	152	75	Yes	Not Follow	No Exercis	Yes
53	Female	176	77	106	95	No	Occasional	Regular Ex	Yes
70	Male	178	79	111	67	Yes	Not Follow	Regular Ex	No
59	Male	165	75	125	88	No	Occasional	Regular Ex	Yes
64	Male	153	56	128	68	Yes	Not Follow	No Exercis	No
45	Female	153	56	129	71	Yes	Not Follow	Regular Ex	No
51	Female	155	74	132	73	No	Not Follow	Occasional	Yes
56	Male	156	67	135	91	Yes	Occasional	Regular Ex	Yes
59	Male	157	61	138	75	No	Occasional	Regular Ex	Yes
60	Male	156	65	142	79	Yes	Not Follow	Regular Ex	Yes

63	Male	157	76	141	80	Yes	Following	Regular Ex	Yes
41	Male	158	49	121	88	Yes	Occasional	Occasional	No
66	Female	159	49	128	90	Yes	Occasional	No Exercis	Yes
54	Male	161	55	127	92	No	Occasional	No Exercis	Yes
43	Male	165	51	129	98	Yes	Not Follow	No Exercis	Yes
52	Male	166	58	132	95	Yes	Following	No Exercis	Yes
63	Female	176	58	142	90	Yes	Not Follow	Regular Ex	Yes
50	Male	171	56	144	85	Yes	Following	No Exercis	No
42	Male	172	71	145	88	Yes	Not Follow	Regular Ex	No
61	Male	143	77	139	89	Yes	Following	No Exercis	Yes
53	Male	147	76	137	75	No	Following	Regular Ex	Yes
55	Female	149	69	131	77	Yes	Not Follow	No Exercis	Yes
48	Male	156	66	151	90	No	Following	Regular Ex	Yes
67	Male	155	72	121	88	Yes	Following	No Exercis	Yes
60	Female	154	73	127	89	Yes	Following	No Exercis	No
64	Female	152	52	125	92	No	Following	Regular Ex	Yes
34	Male	166	77	106	99	Yes	Not Follow	No Exercis	No
59	Female	176	71	109	98	Yes	Following	Regular Ex	Yes
52	Male	171	77	111	95	Not Knowr	Not Follow	Occasional	Yes
57	Female	172	76	100	96	No	Occasional	No Exercis	No
66	Male	143	73	105	92	Yes	Not Follow	Regular Ex	Yes
56	Male	147	69	120	80	Yes	Not Follow	Regular Ex	Yes
54	Female	149	66	132	88	Yes	Occasional	Regular Ex	No
54	Female	156	86	125	87	No	Occasional	No Exercis	Yes
66	Female	155	81	124	85	Yes	Following	Regular Ex	Yes
50	Male	154	83	128	80	No	Not Follow	No Exercis	No
41	Male	152	69	129	80	Yes	Not Follow	Regular Ex	Yes
63	Female	151	79	124	78	Yes	Following	Regular Ex	Yes
57	Male	153	71	126	80	Yes	Following	Occasional	No
43	Female	158	71	121	78	Yes	Occasional	Regular Ex	During Pre
55	Male	166	67	122	69	Yes	Following	No Exercis	Yes
66	Male	169	68	132	75	Yes	Following	No Exercis	Yes
64	Female	179	66	130	81	Yes	Occasional	No Exercis	No
53	Female	177	67	131	80	No	Following	Regular Ex	Yes
68	Female	176	60	123	77	Yes	Following	No Exercis	Yes
45	Female	177	66	125	92	Yes	Occasional	Regular Ex	During Pre
59	Female	177	67	128	82	Yes	Following	Regular Ex	Yes
71	Female	172	92	95	90	No	Occasional	No Exercis	No
56	Female	143	88	105	78	No	Not Follow	Regular Ex	No
45	Female	147	82	119	80	No	Occasional	No Exercis	Yes
60	Female	149	81	98	90	Yes	Following	Regular Ex	Yes
49	Male	156	81	104	67	Yes	Following	No Exercis	No
65	Male	155	84	117	80	Yes	Following	Regular Ex	Yes
64	Male	154	67	120	92	No	Not Follow	No Exercis	Yes
51	Male	152	78	115	95	No	Occasional	Regular Ex	No
35	Male	151	77	121	99	Yes	Following	No Exercis	Yes
70	Female	153	75	120	92	No	Not Follow	No Exercis	Yes

71	Female	158	75	123	85	No	Occasional	No Exercis	No
46	Male	166	67	123	101	No	Occasional	Regular Ex	Yes
41	Male	173	65	123	88	No	Occasional	Regular Ex	Yes
59	Female	179	80	122	85	Yes	Following	Regular Ex	No
66	Female	177	81	124	97	Yes	Following	Regular Ex	No
52	Male	176	82	120	100	No	Not Follow	No Exercis	Yes
50	Female	179	69	120	92	Yes	Following	Occasional	Yes
45	Male	178	65	120	101	No	Occasional	No Exercis	No
41	Female	179	62	124	106	No	Not Follow	No Exercis	Yes
41	Female	174	61	123	98	No	Following	Regular Ex	Yes
56	Female	167	60	124	104	No	Not Follow	Occasional	No
70	Male	150	69	135	78	Yes	Occasional	No Exercis	Yes
41	Male	151	65	138	90	Yes	Not Follow	No Exercis	No
42	Male	145	62	142	74	Yes	Not Follow	Regular Ex	Yes
54	Male	149	61	141	77	Yes	Not Follow	Regular Ex	Yes
48	Male	145	60	121	75	No	Occasional	Regular Ex	No
64	Male	148	65	128	85	No	Occasional	Regular Ex	Yes
77	Male	152	61	127	87	Yes	Occasional	Regular Ex	Yes
48	Male	157	62	129	91	Yes	Not Follow	Regular Ex	Yes
64	Female	156	59	132	85	Yes	Occasional	No Exercis	No
50	Female	178	58	142	84	Yes	Following	Regular Ex	Yes
57	Female	173	81	144	79	Yes	Following	No Exercis	Yes
68	Female	168	71	145	87	Yes	Following	Regular Ex	No
44	Male	179	72	139	92	Yes	Not Follow	Regular Ex	Yes
54	Female	179	86	137	92	No	Following	Regular Ex	Yes
49	Male	177	85	131	90	Yes	Not Follow	Regular Ex	No
50	Female	145	87	151	71	Yes	Not Follow	Regular Ex	Yes
65	Male	156	88	121	90	Yes	Following	No Exercis	Yes
61	Male	176	83	127	94	Yes	Occasional	Regular Ex	Yes
53	Male	179	45	125	88	Yes	Following	Regular Ex	No
51	Male	166	56	129	75	Yes	Following	Regular Ex	Yes
35	Male	174	67	132	68	No	Occasional	No Exercis	Yes
54	Female	156	71	131	69	No	Following	Regular Ex	No
70	Female	159	71	130	78	Yes	Following	Regular Ex	Yes
54	Female	165	75	138	77	Yes	Occasional	Regular Ex	Yes
66	Female	176	67	135	71	Yes	Following	Regular Ex	No
69	Male	167	68	139	72	Not Known	Occasional	No Exercis	No
69	Male	152	68	139	74	No	Occasional	No Exercis	No
69	Male	155	94	120	76	No	Following	No Exercis	Yes
75	Male	164	70	132	95	Yes	Occasional	Occasional	Yes
75	Male	166	70	142	92	Not Known	Following	Regular Ex	Yes
75	Female	175	93	145	84	Not Known	Not Follow	No Exercis	No
75	Female	177	83	102	88	No	Occasional	Occasional	No
75	Female	170	81	110	84	Not Known	Not Follow	Regular Ex	No
75	Female	167	104	108	78	Not Known	Following	No Exercis	No
76	Female	169	54	128	90	No	Not Follow	Regular Ex	Yes
76	Male	150	55	129	74	Yes	Following	Regular Ex	Yes

76	Female	175	85	135	77	Not Knowr	Not Follow	No Exercis	Yes
76	Male	174	80	138	75	Not Knowr	Not Follow	No Exercis	Yes
76	Female	180	83	140	85	Yes	Following	Occasional	No
76	Male	175	62	142	87	Not Knowr	Occasional	Occasional	No
76	Female	174	81	148	77	Not Knowr	Occasional	Regular Ex	No
76	Male	160	89	120	85	No	Not Follow	No Exercis	No
76	Male	170	91	110	94	Not Knowr	Not Follow	Occasional	No
76	Female	180	80	120	95	Not Knowr	Not Follow	No Exercis	Yes
76	Female	150	47	123	92	Not Knowr	Not Follow	Regular Ex	Yes
76	Female	173	88	125	92	No	Occasional	Occasional	No
76	Female	172	75	127	88	Not Knowr	Occasional	No Exercis	No
77	Male	152	59	128	68	No	Occasional	No Exercis	No
77	Male	155	82	128	78	Yes	Occasional	No Exercis	No
77	Male	160	65	129	67	No	Not Follow	No Exercis	No
86	Female	173	86	137	87	No	Occasional	No Exercis	Yes
87	Female	152	68	137	100	Yes	Following	No Exercis	Yes
87	Female	176	81	138	90	Not Knowr	Following	No Exercis	No
97	Female	159	75	138	84	Not Knowr	Occasional	Occasional	Yes
88	Female	175	94	138	90	No	Not Follow	No Exercis	No
93	Male	160	112	138	96	Yes	Not Follow	Occasional	No
87	Female	178	75	139	82	No	Following	No Exercis	No
85	Female	167	67	140	87	Not Knowr	Not Follow	Occasional	No
91	Female	177	70	140	79	Yes	Following	Occasional	No
91	Female	180	97	144	76	No	Not Follow	Occasional	No
93	Male	168	69	140	102	Yes	Following	No Exercis	No
84	Female	181	91	142	90	Not Knowr	Following	No Exercis	No
88	Female	175	81	142	89	Not Knowr	Not Follow	No Exercis	Yes
85	Female	181	92	142	84	No	Not Follow	Occasional	Yes
89	Male	173	95	144	102	Not Knowr	Not Follow	No Exercis	Yes
85	Male	166	69	144	102	Not Knowr	Not Follow	Occasional	No
96	Female	170	64	144	78	Not Knowr	Following	No Exercis	No
99	Female	174	85	145	72	Yes	Following	No Exercis	No
92	Female	160	59	145	75	No	Following	No Exercis	Yes
92	Female	167	64	140	82	No	Not Follow	No Exercis	Yes
93	Female	167	82	152	87	Not Knowr	Following	No Exercis	No
88	Female	168	81	147	88	Not Knowr	Not Follow	Occasional	No
92	Female	181	84	147	76	Not Knowr	Not Follow	No Exercis	No
62	Male	179	83	120	90	Yes	Following	Regular Ex	Yes
61	Male	175	45	129	90	Yes	Following	No Exercis	No
64	Male	174	56	131	90	Yes	Not Follow	Regular Ex	Yes
68	Male	158	67	135	91	Yes	Following	Regular Ex	Yes
51	Male	158	71	134	95	No	Not Follow	No Exercis	No
53	Male	165	71	145	95	Yes	Not Follow	Regular Ex	Yes
50	Male	176	75	142	98	Yes	Following	No Exercis	Yes
58	Male	178	67	111	83	Yes	Following	Regular Ex	Yes
65	Male	165	87	110	81	No	Following	Occasional	No
69	Male	153	69	103	90	Yes	Following	Regular Ex	Yes

60	Male	153	67	102	90	Yes	Not Follow	No Exercis	Yes
51	Male	155	59	120	91	No	Not Follow	Regular Ex	No
64	Male	156	81	125	90	Yes	Following	No Exercis	Yes
61	Male	157	65	127	94	No	Following	Regular Ex	Yes
60	Male	156	68	129	95	Yes	Not Follow	No Exercis	Yes
51	Female	157	61	129	92	Yes	Occasional	Regular Ex	No
55	Male	158	60	130	93	Yes	Not Follow	No Exercis	Yes
41	Male	159	59	131	95	No	Following	Regular Ex	Yes
56	Male	161	54	134	95	Yes	Not Follow	No Exercis	No
57	Female	165	51	137	98	Yes	Not Follow	Regular Ex	Yes
61	Male	166	55	138	97	Yes	Following	Regular Ex	Yes
56	Female	176	51	139	86	Yes	Not Follow	Occasional	Yes
42	Female	171	56	142	98	Yes	Occasional	Occasional	During Pre
60	Female	165	76	130	92	Yes	Following	Regular Ex	Yes
51	Female	155	49	138	93	No	Occasional	Regular Ex	Yes
47	Female	152	49	134	91	Yes	Following	Regular Ex	During Pre
44	Female	150	55	135	96	Yes	Occasional	No Exercis	Yes
42	Male	151	51	134	95	No	Occasional	Regular Ex	Yes
53	Female	145	58	135	94	No	Occasional	Regular Ex	Yes
54	Male	149	58	142	98	Yes	Occasional	Regular Ex	No
66	Female	145	56	144	74	No	Following	No Exercis	Yes
40	Male	148	71	145	85	No	Occasional	Regular Ex	Yes
62	Female	152	77	143	88	No	Following	Regular Ex	No
64	Female	157	76	147	71	Not Known	Following	Regular Ex	Yes
49	Female	156	69	105	95	No	Occasional	Regular Ex	Yes
67	Female	178	66	101	80	Yes	Following	Regular Ex	Yes
61	Female	173	72	109	80	No	Following	No Exercis	No
44	Female	162	73	103	80	No	Occasional	Regular Ex	Yes
44	Female	181	70	121	92	No	Following	Regular Ex	Yes
46	Female	174	70	123	94	Yes	Occasional	No Exercis	During Pre
50	Female	177	56	128	95	Yes	Following	Regular Ex	Yes
64	Female	145	56	127	92	Yes	Following	No Exercis	Yes
64	Female	156	51	125	91	Yes	Following	Regular Ex	Yes
62	Female	176	65	129	88	Yes	Following	No Exercis	No
55	Female	181	65	132	84	Yes	Occasional	Regular Ex	Yes
50	Female	175	62	142	78	Yes	Following	Regular Ex	Yes
57	Male	174	69	145	90	Yes	Not Follow	No Exercis	No
48	Male	168	70	102	74	Yes	Not Follow	Regular Ex	Yes
60	Female	166	71	110	77	Yes	Following	Regular Ex	Yes
64	Female	165	81	108	75	Yes	Not Follow	Regular Ex	Yes
66	Female	176	83	128	85	Yes	Following	No Exercis	No
59	Female	178	83	129	87	No	Not Follow	Occasional	Yes
63	Female	165	84	135	91	No	Occasional	No Exercis	Yes
59	Male	153	70	138	85	No	Not Follow	Regular Ex	No
58	Female	153	78	140	94	Yes	Not Follow	Regular Ex	Yes
65	Male	155	77	142	95	Yes	Not Follow	No Exercis	Yes
58	Female	156	71	148	77	No	Occasional	Regular Ex	Yes

41	Female	157	77	120	92	No	Following	Regular Ex	During Pre
50	Female	156	70	110	88	No	Not Follow	Regular Ex	Yes
45	Male	157	73	99	68	No	Not Follow	Regular Ex	Yes
53	Female	158	69	95	64	Yes	Following	Regular Ex	No
58	Female	159	66	93	67	No	Not Follow	Regular Ex	Yes
41	Male	161	86	97	69	Yes	Not Follow	Regular Ex	Yes
65	Female	165	81	91	78	Yes	Following	Regular Ex	Yes
61	Male	166	83	96	88	Not Known	Not Follow	No Exercis	No
54	Female	176	69	94	80	Yes	Following	Regular Ex	Yes
44	Male	171	70	90	83	Yes	Not Follow	Regular Ex	Yes
41	Male	172	90	100	85	No	Not Follow	Regular Ex	No
56	Male	152	66	132	87	No	Occasional	Regular Ex	Yes
53	Male	155	67	111	90	No	Occasional	Regular Ex	No
52	Male	145	60	120	95	Yes	Occasional	Occasional	Yes
41	Male	146	66	151	92	No	Occasional	Regular Ex	Yes
63	Male	165	67	142	97	Yes	Not Follow	Regular Ex	Yes
59	Female	148	64	123	90	Yes	Not Follow	No Exercis	Yes
44	Male	153	72	132	97	Yes	Occasional	No Exercis	Yes
54	Female	154	73	128	93	No	Not Follow	Regular Ex	No
65	Female	165	59	99	91	Yes	Not Follow	Regular Ex	Yes
45	Female	180	81	135	95	Yes	Occasional	Regular Ex	No
64	Female	175	82	124	80	Yes	Not Follow	No Exercis	Yes
61	Female	160	85	120	85	Yes	Not Follow	No Exercis	Yes
66	Male	139	66	125	88	Yes	Occasional	Regular Ex	Yes
44	Female	165	80	132	86	Yes	Occasional	Regular Ex	Yes
60	Female	167	52	125	87	Yes	Occasional	No Exercis	Yes
38	Male	156	55	128	89	No	Occasional	Regular Ex	No
44	Female	155	56	99	71	Yes	Occasional	No Exercis	Yes
43	Male	176	60	98	99	Yes	Occasional	Regular Ex	No
48	Male	178	60	99	80	Yes	Not Follow	No Exercis	Yes
35	Female	179	61	99	78	No	Not Follow	Regular Ex	Yes
49	Male	145	57	102	95	Yes	Not Follow	No Exercis	Yes
56	Female	172	83	108	89	Yes	Following	Regular Ex	No
47	Female	143	84	107	92	No	Following	Regular Ex	Yes
41	Male	147	82	106	66	Yes	Following	No Exercis	Yes
51	Male	149	76	109	77	Yes	Following	Regular Ex	No
47	Female	156	79	111	90	Yes	Following	Regular Ex	Yes
47	Male	155	78	100	90	Yes	Following	No Exercis	Yes
54	Female	154	86	105	84	Yes	Following	Regular Ex	No
67	Female	152	89	120	88	No	Following	No Exercis	Yes
56	Male	151	67	132	82	No	Following	Regular Ex	No
57	Female	153	66	125	66	Yes	Following	Regular Ex	Yes
43	Male	158	63	124	89	Yes	Following	No Exercis	Yes
59	Male	179	58	128	78	Yes	Following	Regular Ex	Yes
55	Female	181	56	129	82	No	Following	Regular Ex	Yes
55	Male	181	67	124	92	Yes	Following	No Exercis	Yes
52	Male	174	58	126	79	Yes	Following	Regular Ex	Yes

53	Male	176	61	121	80	Yes	Following	No Exercise	Yes
58	Male	167	63	122	86	Yes	Following	Regular Exe	No
29	Male	178	67	132	85	Yes	Following	Regular Exe	Yes
71	Female	179	78	130	71	No	Following	No Exercise	Yes
62	Female	176	75	108	73	Yes	Following	Occasional	Yes
68	Female	178	72	121	81	Yes	Following	No Exercise	Yes
41	Male	165	70	102	88	Yes	Not Follow	Regular Exe	No
57	Female	153	67	152	78	Yes	Following	Regular Exe	Yes
64	Female	153	66	121	81	No	Following	Regular Exe	Yes
57	Female	155	79	142	85	Yes	Following	Regular Exe	Yes
49	Female	156	89	131	71	No	Occasional	Occasional	No
52	Female	157	90	148	84	Yes	Following	Regular Exe	Yes
54	Female	156	93	148	75	Yes	Following	Regular Exe	Yes
41	Female	157	94	129	89	Yes	Occasional	No Exercise	No
62	Female	158	87	108	92	Yes	Following	Regular Exe	Yes
59	Male	159	49	135	66	Yes	Following	No Exercise	Yes
58	Male	161	58	120	77	No	Occasional	Regular Exe	Yes
58	Female	165	84	140	80	Yes	Following	No Exercise	No
50	Female	166	88	150	74	Yes	Following	Regular Exe	Yes
62	Female	176	94	112	84	Yes	Occasional	Regular Exe	Yes
45	Male	171	91	142	88	Yes	Not Follow	No Exercise	No
74	Female	172	68	130	82	No	Following	Regular Exe	Yes
55	Male	143	88	114	66	Yes	Not Follow	No Exercise	Yes
48	Male	147	69	131	89	No	Not Follow	Regular Exe	Yes
53	Female	149	81	142	78	Yes	Following	No Exercise	No
60	Female	156	83	151	82	Yes	Occasional	No Exercise	Yes
40	Male	155	85	132	92	Yes	Not Follow	No Exercise	Yes
63	Male	154	76	138	79	Yes	Occasional	Regular Exe	No
52	Male	152	71	101	80	Yes	Not Follow	No Exercise	Yes
44	Male	151	72	110	86	No	Not Follow	Regular Exe	No
61	Male	153	77	108	66	Yes	Not Follow	No Exercise	Yes
48	Female	158	66	104	71	Yes	Occasional	Regular Exe	Yes

Non Alcoho	Low	Regular	Middle Cla	No
Non Alcoho	Low	Regular	High Class	No
Non Alcoho	High	Non Smoke	High Class	No
Non Alcoho	Low	Regular	High Class	Yes
Alcoholic	Low	Occasional	Middle Cla	No
Non Alcoho	High	Regular	High Class	Yes
Non Alcoho	High	Regular	Poor	Yes
Non Alcoho	Low	Occasional	High Class	Yes
Non Alcoho	High	Regular	Poor	Yes
Non Alcoho	High	Non Smoke	Poor	Yes
Non Alcoho	Low	Regular	High Class	No
Non Alcoho	High	Non Smoke	Poor	Yes
Non Alcoho	High	Regular	Poor	No
Alcoholic	High	Regular	High Class	Yes
Non Alcoho	High	Regular	Middle Cla	Yes
Occasional	High	Regular	High Class	No
Occasional	High	Occasional	Middle Cla	Yes
Non Alcoho	Low	Regular	Middle Cla	Yes
Non Alcoho	High	Regular	High Class	No
Occasional	High	Occasional	High Class	No
Non Alcoho	High	Occasional	Middle Cla	No
Alcoholic	Low	Regular	Middle Cla	No
Alcoholic	High	Regular	Middle Cla	Yes
Non Alcoho	High	Occasional	Middle Cla	No
Non Alcoho	Low	Regular	Middle Cla	Yes
Non Alcoho	High	Occasional	Middle Cla	Yes
Occasional	High	Regular	Poor	No
Alcoholic	High	Regular	Poor	Yes
Non Alcoho	High	Occasional	Poor	Yes
Occasional	High	Regular	Poor	No
Alcoholic	High	Regular	High Class	No
Non Alcoho	High	Regular	High Class	No
Occasional	High	Non Smoke	High Class	No
Alcoholic	Low	Regular	Poor	Yes
Non Alcoho	High	Regular	High Class	No
Occasional	High	Regular	Poor	Yes
Alcoholic	High	Regular	Middle Cla	Yes
Non Alcoho	High	Occasional	Middle Cla	Yes
Occasional	Low	Regular	Middle Cla	No
Alcoholic	High	Regular	Middle Cla	Yes
Non Alcoho	High	Regular	Middle Cla	Yes
Non Alcoho	High	Regular	High Class	No
Non Alcoho	High	Non Smoke	Middle Cla	Yes
Occasional	High	Regular	High Class	No
Non Alcoho	High	Regular	High Class	No
Non Alcoho	Low	Regular	High Class	No

Non Alcoh	High	Regular	High Class	Yes
Non Alcoh	Low	Occasional	High Class	No
Non Alcoh	High	Regular	Poor	Yes
Occasional	Low	Regular	High Class	No
Non Alcoh	High	Occasional	High Class	Yes
Non Alcoh	Low	Regular	High Class	Yes
Non Alcoh	Low	Occasional	High Class	No
Non Alcoh	High	Regular	High Class	No
Occasional	Low	Regular	Middle Cla	Yes
Occasional	Low	Occasional	High Class	No
Non Alcoh	High	Regular	Middle Cla	Yes
Non Alcoh	Low	Regular	Poor	No
Non Alcoh	High	Regular	High Class	Yes
Occasional	Low	Occasional	Middle Cla	Yes
Non Alcoh	High	Regular	Middle Cla	No
Non Alcoh	High	Regular	High Class	No
Alcoholic	High	Regular	Poor	No
Non Alcoh	High	Regular	Middle Cla	Yes
Non Alcoh	Moderate	Occasional	Poor	No
Non Alcoh	High	Regular	Poor	Yes
Non Alcoh	High	Non Smoke	Poor	Yes
Non Alcoh	High	Occasional	Poor	No
Non Alcoh	High	Regular	Poor	Yes
Non Alcoh	High	Occasional	High Class	No
Occasional	High	Regular	Middle Cla	Yes
Non Alcoh	Moderate	Regular	High Class	No
Non Alcoh	Moderate	Occasional	Middle Cla	No
Non Alcoh	High	Regular	High Class	Yes
Non Alcoh	High	Regular	Poor	No
Non Alcoh	High	Regular	Middle Cla	No
Non Alcoh	Low	Occasional	Poor	Yes
Non Alcoh	High	Regular	Middle Cla	Yes
Non Alcoh	High	Regular	Poor	Yes
Non Alcoh	Moderate	Regular	Poor	No
Non Alcoh	High	Regular	Middle Cla	No
Non Alcoh	High	Regular	High Class	No
Non Alcoh	Moderate	Regular	High Class	No
Non Alcoh	Low	Regular	Middle Cla	No
Non Alcoh	High	Regular	High Class	Yes
Non Alcoh	High	Occasional	Middle Cla	Yes
Non Alcoh	Moderate	Regular	High Class	No
Non Alcoh	Low	Regular	Middle Cla	Yes
Non Alcoh	High	Occasional	High Class	Yes
Non Alcoh	High	Regular	Middle Cla	No
Non Alcoh	High	Occasional	High Class	Yes
Non Alcoh	High	Regular	High Class	No
Alcoholic	Moderate	Regular	Poor	Yes

Non Alcoh	Low	Occasional	Middle Cla	Yes
Non Alcoh	High	Regular	Middle Cla	Yes
Non Alcoh	High	Regular	Poor	No
Non Alcoh	Moderate	Regular	High Class	Yes
Non Alcoh	Low	Occasional	High Class	Yes
Non Alcoh	High	Regular	High Class	Yes
Non Alcoh	High	Regular	High Class	Yes
Non Alcoh	Moderate	Regular	Middle Cla	Yes
Non Alcoh	Low	Regular	High Class	No
Non Alcoh	High	Regular	Middle Cla	No
Occasional	High	Non Smok	High Class	No
Non Alcoh	High	Non Smok	Poor	Yes
Occasional	High	Occasional	High Class	Yes
Non Alcoh	High	Occasional	High Class	No
Occasional	Low	Non Smok	Middle Cla	No
Alcoholic	Low	Regular	High Class	No
Alcoholic	Low	Regular	Poor	No
Alcoholic	Low	Regular	High Class	No
Alcoholic	Low	Non Smok	Poor	Yes
Alcoholic	Low	Occasional	High Class	Yes
Alcoholic	Low	Non Smok	High Class	Yes
Non Alcoh	Moderate	Regular	High Class	No
Non Alcoh	Moderate	Occasional	Poor	Yes
Non Alcoh	Low	Non Smok	High Class	Yes
Non Alcoh	Low	Non Smok	High Class	No
Non Alcoh	Low	Non Smok	High Class	Yes
Non Alcoh	Low	Regular	Middle Cla	Yes
Alcoholic	Moderate	Regular	Poor	No
Alcoholic	Moderate	Regular	Poor	No
Alcoholic	Moderate	Non Smok	High Class	Yes
Alcoholic	High	Regular	Poor	Yes
Non Alcoh	High	Occasional	Poor	Yes
Non Alcoh	High	Regular	Poor	No
Non Alcoh	High	Regular	Poor	Yes
Non Alcoh	Low	Occasional	Middle Cla	Yes
Non Alcoh	High	Regular	Poor	Yes
Non Alcoh	High	Occasional	Poor	Yes
Non Alcoh	High	Regular	Middle Cla	No
Non Alcoh	Low	Regular	Poor	Yes
Non Alcoh	High	Occasional	Poor	Yes
Non Alcoh	High	Regular	Poor	Yes
Alcoholic	High	Regular	Poor	Yes
Non Alcoh	High	Regular	Poor	Yes
Non Alcoh	Low	Occasional	Middle Cla	No
Non Alcoh	High	Regular	Poor	Yes
Non Alcoh	High	Regular	Poor	Yes
Non Alcoh	High	Regular	Middle Cla	Yes

Non Alcoho	Low	Regular	Poor	Yes
Non Alcoho	High	Regular	Middle Cla	No
Non Alcoho	High	Regular	Poor	Yes
Non Alcoho	High	Regular	High Class	Yes
Non Alcoho	High	Occasional	Poor	No
Non Alcoho	High	Regular	Poor	No
Non Alcoho	High	Regular	Poor	Yes
Alcoholic	High	Occasional	Poor	Yes
Occasional	High	Regular	Poor	Yes
Non Alcoho	High	Non Smoke	High Class	Yes
Non Alcoho	High	Regular	Poor	Yes
Alcoholic	High	Regular	Poor	Yes
Non Alcoho	High	Occasional	Middle Cla	Yes
Non Alcoho	High	Regular	Middle Cla	Yes
Alcoholic	Low	Regular	High Class	Yes
Non Alcoho	High	Regular	Poor	Yes
Non Alcoho	High	Occasional	Middle Cla	No
Occasional	High	Regular	High Class	No
Non Alcoho	High	Regular	Poor	Yes
Occasional	High	Regular	High Class	Yes
Occasional	High	Regular	Middle Cla	Yes
Non Alcoho	High	Occasional	Middle Cla	Yes
Non Alcoho	High	Regular	Middle Cla	Yes
Non Alcoho	High	Regular	High Class	Yes
Non Alcoho	High	Occasional	Poor	No
Non Alcoho	High	Regular	High Class	No
Non Alcoho	High	Occasional	High Class	No
Non Alcoho	High	Regular	Middle Cla	Yes
Non Alcoho	High	Regular	High Class	No
Non Alcoho	High	Occasional	High Class	No
Non Alcoho	High	Regular	High Class	No
Non Alcoho	High	Regular	Middle Cla	Yes
Non Alcoho	High	Regular	High Class	No
Non Alcoho	High	Occasional	Poor	Yes
Non Alcoho	High	Regular	Poor	Yes
Non Alcoho	Low	Non Smoke	Poor	Yes
Non Alcoho	High	Regular	High Class	Yes
Non Alcoho	High	Regular	Poor	Yes
Non Alcoho	High	Occasional	Poor	Yes
Non Alcoho	High	Regular	High Class	No
Alcoholic	High	Non Smoke	High Class	No
Alcoholic	High	Occasional	Middle Cla	Yes
Alcoholic	High	Regular	High Class	No
Occasional	High	Occasional	High Class	No
Non Alcoho	Low	Regular	Poor	Yes
Non Alcoho	High	Regular	High Class	No
Occasional	High	Occasional	Middle Cla	No

Non Alcoh	High	Regular	High Class	Yes
Non Alcoh	High	Regular	High Class	Yes
Occasional	High	Regular	Poor	Yes
Non Alcoh	High	Occasional	High Class	Yes
Non Alcoh	High	Regular	High Class	No
Non Alcoh	High	Regular	High Class	No
Non Alcoh	High	Regular	High Class	No
Non Alcoh	High	Regular	High Class	No
Non Alcoh	High	Occasional	Middle Cla	No
Alcoholic	Low	Regular	Middle Cla	No
Alcoholic	High	Regular	Middle Cla	Yes
Non Alcoh	High	Occasional	Middle Cla	No
Non Alcoh	High	Occasional	Middle Cla	Yes
Non Alcoh	High	Regular	Poor	Yes
Non Alcoh	High	Regular	Poor	Yes
Non Alcoh	Low	Regular	Middle Cla	Yes
Non Alcoh	Low	Regular	Poor	Yes
Non Alcoh	Low	Occasional	Middle Cla	Yes
Occasional	Low	Non Smoke	Middle Cla	Yes
Non Alcoh	Low	Regular	Poor	Yes
Non Alcoh	High	Occasional	Middle Cla	Yes
Non Alcoh	High	Regular	Poor	Yes
Non Alcoh	Moderate	Occasional	Poor	Yes
Non Alcoh	High	Regular	High Class	Yes
Non Alcoh	High	Regular	Poor	No
Non Alcoh	High	Occasional	High Class	No
Occasional	High	Regular	High Class	No
Non Alcoh	High	Regular	High Class	Yes
Non Alcoh	High	Regular	Middle Cla	No
Non Alcoh	High	Occasional	Middle Cla	No
Occasional	High	Regular	Middle Cla	No
Non Alcoh	High	Regular	Middle Cla	No
Non Alcoh	High	Regular	Middle Cla	No
Non Alcoh	High	Regular	Poor	No
Occasional	High	Occasional	High Class	No
Non Alcoh	High	Regular	Middle Cla	No
Non Alcoh	High	Occasional	High Class	Yes
Non Alcoh	Low	Regular	Poor	Yes
Non Alcoh	Low	Regular	Poor	No
Non Alcoh	Low	Occasional	Poor	No
Non Alcoh	Low	Regular	Poor	No
Non Alcoh	High	Regular	Middle Cla	No
Non Alcoh	High	Regular	Middle Cla	No
Occasional	High	Occasional	High Class	No
Non Alcoh	High	Regular	High Class	No
Non Alcoh	High	Regular	High Class	No
Non Alcoh	High	Regular	Middle Cla	No

Non Alcoh	High	Regular	Middle Cla	No
Non Alcoh	High	Occasional	Middle Cla	No
Non Alcoh	Low	Regular	High Class	Yes
Non Alcoh	High	Regular	High Class	Yes
Non Alcoh	High	Occasional	Poor	Yes
Non Alcoh	Low	Regular	Poor	No
Non Alcoh	High	Occasional	Poor	Yes
Non Alcoh	Low	Regular	High Class	No
Alcoholic	Moderate	Occasional	High Class	Yes
Occasional	High	Regular	High Class	No
Non Alcoh	Low	Occasional	High Class	Yes
Non Alcoh	Low	Regular	Poor	No
Non Alcoh	Low	Regular	High Class	Yes
Non Alcoh	Low	Occasional	Middle Cla	No
Non Alcoh	Low	Regular	Poor	No
Alcoholic	Low	Occasional	Poor	Yes
Non Alcoh	Low	Regular	Poor	Yes
Occasional	High	Regular	Poor	Yes
Non Alcoh	High	Occasional	Middle Cla	Yes
Non Alcoh	High	Regular	Poor	Yes
Non Alcoh	High	Regular	High Class	Yes
Non Alcoh	High	Regular	High Class	Yes
Non Alcoh	High	Non Smoke	Middle Cla	No
Occasional	High	Regular	Middle Cla	Yes
Non Alcoh	High	Regular	Middle Cla	Yes
Non Alcoh	High	Regular	Middle Cla	No
Non Alcoh	High	Regular	Middle Cla	Yes
Non Alcoh	Low	Occasional	High Class	No
Non Alcoh	Low	Regular	Middle Cla	Yes
Non Alcoh	High	Occasional	High Class	No
Occasional	High	Regular	Poor	Yes
Non Alcoh	High	Regular	Middle Cla	Yes
Non Alcoh	Low	Occasional	Middle Cla	Yes
Non Alcoh	Low	Regular	High Class	Yes
Non Alcoh	High	Occasional	Poor	No
Non Alcoh	High	Regular	Poor	No
Non Alcoh	Low	Regular	Poor	No
Non Alcoh	Low	Occasional	High Class	Yes
Occasional	Low	Regular	High Class	No
Occasional	High	Regular	High Class	Yes
Non Alcoh	High	Regular	High Class	No
Occasional	High	Occasional	High Class	No
Non Alcoh	High	Regular	Poor	No
Non Alcoh	High	Regular	Middle Cla	No
Non Alcoh	Low	Regular	High Class	No
Non Alcoh	High	Regular	High Class	Yes
Occasional	Low	Occasional	High Class	No

Occasional	Low	Regular	High Class	Yes
Non Alcoho	High	Regular	High Class	Yes
Non Alcoho	Low	Occasional	High Class	No
Non Alcoho	High	Regular	High Class	No
Non Alcoho	High	Occasional	High Class	Yes
Non Alcoho	Low	Regular	High Class	No
Non Alcoho	High	Occasional	High Class	Yes
Occasional	High	Regular	High Class	Yes
Non Alcoho	Low	Occasional	Poor	No
Non Alcoho	High	Non Smoke	Poor	Yes
Non Alcoho	High	Regular	Poor	No
Non Alcoho	High	Occasional	Middle Cla	Yes
Non Alcoho	High	Regular	Poor	Yes
Non Alcoho	Low	Regular	Poor	No
Occasional	High	Regular	High Class	No
Non Alcoho	Low	Occasional	Poor	No
Non Alcoho	High	Regular	Poor	Yes
Occasional	Low	Regular	Middle Cla	Yes
Non Alcoho	Low	Regular	Poor	Yes
Non Alcoho	High	Regular	Poor	Yes
Non Alcoho	High	Occasional	High Class	No
Non Alcoho	High	Regular	Poor	Yes
Occasional	Low	Regular	Poor	No
Occasional	Low	Occasional	Middle Cla	No
Occasional	High	Regular	Poor	Yes
Non Alcoho	Low	Occasional	Poor	No
Non Alcoho	High	Regular	Middle Cla	Yes
Non Alcoho	Low	Regular	Poor	No
Non Alcoho	High	Occasional	Poor	Yes
Non Alcoho	High	Regular	Middle Cla	No
Non Alcoho	High	Regular	Middle Cla	No
Alcoholic	High	Regular	Middle Cla	No
Non Alcoho	High	Occasional	Middle Cla	No
Occasional	Moderate	Occasional	Middle Cla	No
Non Alcoho	Moderate	Regular	Middle Cla	No
Non Alcoho	High	Occasional	Poor	Yes
Non Alcoho	Moderate	Regular	Poor	No
Non Alcoho	High	Regular	High Class	Yes
Non Alcoho	High	Occasional	Poor	No
Non Alcoho	High	Regular	Poor	Yes
Non Alcoho	High	Regular	Poor	No
Non Alcoho	High	Regular	Middle Cla	No
Non Alcoho	High	Occasional	High Class	Yes
Non Alcoho	High	Regular	Poor	Yes
Occasional	High	Regular	High Class	No
Non Alcoho	High	Regular	Middle Cla	Yes
Non Alcoho	High	Regular	Poor	No

Non Alcoh	High	Occasional	High Class	No
Non Alcoh	High	Regular	Poor	Yes
Non Alcoh	High	Regular	High Class	Yes
Non Alcoh	High	Occasional	Poor	Yes
Occasional	High	Regular	High Class	No
Non Alcoh	High	Occasional	Poor	Yes
Non Alcoh	High	Regular	Middle Cla	No
Non Alcoh	High	Regular	Middle Cla	No
Non Alcoh	High	Occasional	Middle Cla	Yes
Non Alcoh	Moderate	Regular	Middle Cla	No
Occasional	Moderate	Regular	Middle Cla	No
Non Alcoh	High	Regular	Poor	Yes
Non Alcoh	High	Occasional	High Class	No
Non Alcoh	Moderate	Regular	Middle Cla	No
Non Alcoh	High	Regular	High Class	Yes
Non Alcoh	High	Regular	Poor	No
Non Alcoh	High	Regular	High Class	No
Occasional	High	Regular	Poor	No
Non Alcoh	Low	Occasional	Poor	No
Non Alcoh	High	Regular	High Class	Yes
Non Alcoh	High	Regular	High Class	Yes
Non Alcoh	High	Occasional	Middle Cla	Yes
Non Alcoh	High	Regular	High Class	No
Non Alcoh	High	Occasional	High Class	No
Non Alcoh	High	Regular	Poor	Yes
Non Alcoh	High	Regular	High Class	No
Non Alcoh	High	Occasional	Middle Cla	Yes
Non Alcoh	Low	Regular	High Class	Yes
Non Alcoh	High	Regular	High Class	Yes
Non Alcoh	High	Regular	Poor	No
Non Alcoh	High	Occasional	High Class	Yes
Non Alcoh	High	Regular	High Class	No
Non Alcoh	High	Regular	High Class	Yes
Non Alcoh	High	Non Smoke	High Class	Yes
Non Alcoh	High	Regular	High Class	No
Non Alcoh	High	Occasional	Middle Cla	Yes
Non Alcoh	High	Regular	Middle Cla	No
Non Alcoh	Moderate	Non Smoke	Middle Cla	No
Non Alcoh	Low	Occasional	Middle Cla	No
Non Alcoh	Moderate	Regular	Middle Cla	No
Non Alcoh	High	Occasional	Poor	No
Non Alcoh	High	Regular	Poor	No
Non Alcoh	High	Occasional	Poor	No
Non Alcoh	High	Regular	Poor	Yes
Non Alcoh	High	Non Smoke	Poor	No
Non Alcoh	Moderate	Occasional	Poor	No
Non Alcoh	Moderate	Regular	Poor	Yes

Non Alcoh	High	Regular	High Class	Yes
Non Alcoh	Moderate	Regular	Middle Cla	No
Non Alcoh	High	Occasional	High Class	Yes
Non Alcoh	High	Regular	Poor	No
Non Alcoh	High	Regular	Poor	Yes
Occasional	High	Regular	Poor	No
Non Alcoh	High	Regular	Poor	Yes
Non Alcoh	Moderate	Occasional	High Class	No
Non Alcoh	High	Regular	High Class	No
Non Alcoh	Moderate	Regular	High Class	No
Non Alcoh	Moderate	Occasional	High Class	Yes
Non Alcoh	High	Regular	Middle Cla	No
Non Alcoh	High	Occasional	Middle Cla	No
Alcoholic	High	Occasional	High Class	No
Non Alcoh	Low	Regular	High Class	No
Non Alcoh	High	Non Smok	Poor	Yes
Non Alcoh	High	Occasional	High Class	Yes
Non Alcoh	High	Regular	High Class	Yes
Non Alcoh	High	Regular	Poor	Yes
Non Alcoh	Low	Non Smok	High Class	No
Non Alcoh	High	Occasional	Poor	Yes
Non Alcoh	Low	Regular	Middle Cla	No
Alcoholic	High	Regular	Poor	Yes
Alcoholic	Low	Regular	Middle Cla	No
Non Alcoh	Low	Regular	Middle Cla	No
Non Alcoh	High	Occasional	Poor	Yes
Non Alcoh	High	Regular	Poor	Yes
Non Alcoh	High	Regular	Middle Cla	No
Non Alcoh	High	Occasional	Middle Cla	Yes
Non Alcoh	High	Regular	Middle Cla	Yes
Non Alcoh	High	Occasional	Middle Cla	Yes
Non Alcoh	High	Regular	Middle Cla	Yes
Non Alcoh	High	Regular	Poor	Yes
Alcoholic	Low	Occasional	Middle Cla	Yes
Non Alcoh	Low	Regular	Middle Cla	No
Non Alcoh	High	Occasional	High Class	No
Non Alcoh	Low	Regular	High Class	Yes
Non Alcoh	Low	Regular	High Class	No
Non Alcoh	High	Occasional	High Class	Yes
Non Alcoh	High	Regular	High Class	Yes
Non Alcoh	Low	Regular	Middle Cla	Yes
Non Alcoh	Low	Regular	High Class	No
Occasional	High	Occasional	Middle Cla	Yes
Non Alcoh	High	Regular	Poor	No
Non Alcoh	Low	Regular	High Class	Yes
Non Alcoh	Low	Regular	Middle Cla	Yes
Non Alcoh	High	Regular	High Class	No

Non Alcoh	High	Occasional	Middle Cla	Yes
Non Alcoh	High	Regular	High Class	Yes
Non Alcoh	High	Non Smoke	Middle Cla	No
Non Alcoh	Low	Occasional	High Class	Yes
Non Alcoh	Low	Regular	Middle Cla	No
Occasional	High	Occasional	High Class	Yes
Non Alcoh	High	Regular	Poor	Yes
Non Alcoh	High	Regular	Poor	Yes
Non Alcoh	High	Occasional	High Class	Yes
Non Alcoh	High	Regular	High Class	Yes
Non Alcoh	High	Regular	Poor	Yes
Non Alcoh	High	Regular	Poor	No
Non Alcoh	High	Occasional	Poor	Yes
Non Alcoh	High	Regular	Poor	Yes
Occasional	High	Regular	Poor	Yes
Non Alcoh	High	Regular	Poor	No
Non Alcoh	High	Occasional	High Class	No
Non Alcoh	High	Regular	High Class	Yes
Non Alcoh	High	Regular	High Class	No
Non Alcoh	High	Occasional	High Class	Yes
Non Alcoh	Low	Regular	Middle Cla	No
Non Alcoh	High	Occasional	High Class	No
Non Alcoh	Low	Regular	High Class	Yes
Non Alcoh	Low	Regular	Poor	Yes
Non Alcoh	High	Occasional	High Class	Yes
Non Alcoh	High	Regular	High Class	No
Alcoholic	Low	Regular	Middle Cla	No
Non Alcoh	Low	Regular	High Class	Yes
Non Alcoh	High	Occasional	Middle Cla	Yes
Alcoholic	High	Regular	High Class	No
Non Alcoh	Low	Regular	High Class	Yes
Non Alcoh	Low	Regular	Poor	Yes
Non Alcoh	High	Regular	High Class	No
Non Alcoh	High	Regular	High Class	No
Non Alcoh	Low	Regular	Poor	Yes
Non Alcoh	Low	Regular	High Class	No
Non Alcoh	High	Regular	High Class	Yes
Non Alcoh	Low	Regular	Poor	No
Non Alcoh	High	Regular	High Class	Yes
Non Alcoh	High	Regular	High Class	No
Non Alcoh	High	Occasional	High Class	Yes
Occasional	Low	Regular	High Class	Yes
Non Alcoh	Low	Regular	Poor	Yes
Non Alcoh	High	Occasional	Poor	Yes
Non Alcoh	High	Regular	High Class	No
Non Alcoh	High	Occasional	High Class	Yes
Non Alcoh	Low	Regular	High Class	Yes

Non Alcoh	High	Regular	High Class	No
Non Alcoh	High	Occasional	High Class	Yes
Occasional	High	Regular	High Class	No
Non Alcoh	Low	Regular	Poor	Yes
Non Alcoh	High	Regular	Poor	Yes
Non Alcoh	High	Occasional	High Class	No
Non Alcoh	High	Regular	Poor	Yes
Non Alcoh	Low	Regular	High Class	No
Non Alcoh	High	Regular	High Class	No
Non Alcoh	High	Regular	High Class	Yes
Non Alcoh	Low	Regular	Middle Cla	No
Non Alcoh	High	Regular	Poor	Yes
Non Alcoh	High	Occasional	High Class	Yes
Occasional	High	Regular	High Class	No
Non Alcoh	High	Regular	High Class	No
Non Alcoh	High	Regular	Middle Cla	Yes
Non Alcoh	Low	Occasional	Poor	Yes
Non Alcoh	High	Regular	Poor	Yes
Non Alcoh	High	Regular	High Class	No
Non Alcoh	High	Regular	Poor	Yes
Non Alcoh	High	Regular	Middle Cla	Yes
Non Alcoh	High	Regular	Middle Cla	Yes
Non Alcoh	Low	Regular	Poor	Yes
Non Alcoh	High	Regular	Poor	Yes
Non Alcoh	High	Regular	High Class	Yes
Non Alcoh	High	Occasional	High Class	No
Non Alcoh	High	Regular	High Class	Yes
Non Alcoh	High	Regular	High Class	Yes
Non Alcoh	Low	Occasional	Middle Cla	Yes
Non Alcoh	High	Regular	High Class	No
Non Alcoh	High	Occasional	Middle Cla	Yes
Non Alcoh	High	Regular	High Class	No
Non Alcoh	High	Regular	Middle Cla	No
Non Alcoh	Low	Occasional	High Class	Yes
Non Alcoh	High	Regular	Middle Cla	No
Occasional	High	Regular	Poor	Yes
Alcoholic	Moderate	Occasional	Middle Cla	No
Alcoholic	Moderate	Occasional	Poor	No
Alcoholic	Low	Occasional	Poor	No
Alcoholic	Moderate	Occasional	Poor	No
Non Alcoh	Moderate	Occasional	Middle Cla	No
Occasional	Low	Non Smoke	Poor	Yes
Alcoholic	Low	Regular	Poor	Yes
Non Alcoh	Low	Regular	Middle Cla	Yes
Occasional	Low	Regular	Middle Cla	Yes
Alcoholic	Low	Non Smoke	Poor	Yes
Alcoholic	Low	Non Smoke	Poor	No

Alcoholic	Low	Non Smoke	High Class	Yes
Non Alcoho	Low	Regular	High Class	No
Non Alcoho	Moderate	Regular	High Class	No
Non Alcoho	Moderate	Regular	Poor	No
Non Alcoho	Moderate	Regular	Poor	Yes
Non Alcoho	Moderate	Regular	Poor	No
Occasional	Low	Regular	High Class	No
Occasional	Low	Regular	Middle Cla	Yes
Occasional	Low	Regular	Poor	Yes
Non Alcoho	Low	Regular	Middle Cla	Yes
Alcoholic	Low	Regular	Middle Cla	Yes
Occasional	Low	Occasional	Poor	No
Occasional	Low	Occasional	Poor	No
Occasional	Low	Regular	Poor	No
Alcoholic	High	Non Smoke	High Class	Yes
Alcoholic	Low	Regular	Middle Cla	Yes
Non Alcoho	Low	Regular	High Class	Yes
Non Alcoho	Low	Regular	Middle Cla	Yes
Non Alcoho	Low	Non Smoke	Poor	Yes
Non Alcoho	High	Non Smoke	High Class	Yes
Non Alcoho	Low	Regular	Middle Cla	Yes
Alcoholic	Low	Regular	High Class	Yes
Occasional	Moderate	Non Smoke	High Class	Yes
Occasional	Low	Occasional	High Class	Yes
Non Alcoho	High	Non Smoke	High Class	Yes
Non Alcoho	Low	Non Smoke	Poor	Yes
Non Alcoho	Low	Occasional	Poor	Yes
Alcoholic	Low	Regular	High Class	Yes
Non Alcoho	Low	Non Smoke	Poor	Yes
Alcoholic	Low	Regular	High Class	Yes
Non Alcoho	High	Non Smoke	Poor	Yes
Non Alcoho	Low	Non Smoke	High Class	Yes
Occasional	Low	Occasional	High Class	Yes
Occasional	Moderate	Occasional	Poor	Yes
Non Alcoho	High	Non Smoke	High Class	Yes
Non Alcoho	Low	Occasional	High Class	Yes
Occasional	Moderate	Non Smoke	High Class	Yes
Non Alcoho	High	Regular	Middle Cla	Yes
Non Alcoho	High	Occasional	High Class	Yes
Non Alcoho	High	Regular	Middle Cla	Yes
Non Alcoho	Low	Regular	High Class	Yes
Non Alcoho	High	Regular	Middle Cla	No
Non Alcoho	High	Occasional	Poor	Yes
Occasional	High	Regular	Middle Cla	Yes
Non Alcoho	High	Regular	Poor	Yes
Non Alcoho	Low	Regular	Poor	No
Non Alcoho	High	Regular	Poor	Yes

Non Alcoh	High	Regular	Poor	No
Alcoholic	High	Regular	Poor	No
Non Alcoh	Low	Regular	Poor	Yes
Non Alcoh	High	Regular	Middle Cla	Yes
Non Alcoh	High	Occasional	Middle Cla	Yes
Non Alcoh	High	Regular	Middle Cla	No
Non Alcoh	High	Regular	Middle Cla	Yes
Non Alcoh	Low	Occasional	Middle Cla	Yes
Non Alcoh	High	Regular	Middle Cla	Yes
Non Alcoh	High	Occasional	Middle Cla	Yes
Non Alcoh	High	Regular	Middle Cla	Yes
Non Alcoh	Low	Regular	Middle Cla	Yes
Non Alcoh	High	Occasional	Middle Cla	No
Non Alcoh	High	Regular	Poor	Yes
Non Alcoh	Moderate	Regular	Poor	No
Non Alcoh	Low	Regular	Poor	Yes
Non Alcoh	High	Regular	Middle Cla	No
Non Alcoh	Moderate	Regular	Middle Cla	No
Non Alcoh	High	Occasional	Middle Cla	No
Alcoholic	Low	Regular	Middle Cla	No
Non Alcoh	High	Regular	Poor	Yes
Non Alcoh	High	Occasional	Poor	No
Non Alcoh	High	Regular	Poor	Yes
Non Alcoh	Moderate	Occasional	Poor	Yes
Non Alcoh	Low	Regular	Poor	No
Non Alcoh	High	Regular	Middle Cla	Yes
Non Alcoh	Moderate	Occasional	Middle Cla	No
Non Alcoh	High	Regular	Poor	Yes
Non Alcoh	Low	Regular	Middle Cla	Yes
Non Alcoh	High	Regular	Poor	No
Alcoholic	High	Occasional	Poor	Yes
Non Alcoh	High	Regular	Poor	Yes
Non Alcoh	Low	Regular	Poor	Yes
Non Alcoh	Low	Non Smoke	Poor	Yes
Non Alcoh	High	Regular	Poor	No
Non Alcoh	Moderate	Regular	Poor	Yes
Non Alcoh	High	Regular	High Class	Yes
Non Alcoh	Low	Regular	High Class	No
Non Alcoh	High	Regular	Poor	Yes
Non Alcoh	High	Non Smoke	Poor	Yes
Non Alcoh	High	Regular	Poor	Yes
Non Alcoh	Moderate	Regular	Poor	Yes
Non Alcoh	Low	Occasional	High Class	Yes
Occasional	High	Regular	High Class	No
Non Alcoh	Moderate	Occasional	Poor	Yes
Non Alcoh	High	Regular	Middle Cla	Yes
Non Alcoh	Low	Regular	Poor	Yes

Non Alcoh	High	Occasional	Poor	Yes
Non Alcoh	High	Regular	Middle Cla	Yes
Non Alcoh	High	Regular	Middle Cla	No
Non Alcoh	Moderate	Regular	Poor	Yes
Non Alcoh	Moderate	Occasional	Poor	Yes
Occasional	High	Regular	Poor	No
Non Alcoh	High	Regular	Middle Cla	Yes
Non Alcoh	High	Regular	High Class	No
Non Alcoh	High	Regular	Poor	Yes
Non Alcoh	High	Regular	High Class	No
Non Alcoh	High	Regular	Middle Cla	No
Occasional	High	Non Smoke	Middle Cla	No
Non Alcoh	High	Regular	Middle Cla	No
Non Alcoh	High	Occasional	Middle Cla	No
Non Alcoh	High	Regular	Middle Cla	No
Non Alcoh	High	Regular	Middle Cla	Yes
Non Alcoh	High	Occasional	High Class	Yes
Alcoholic	High	Regular	Middle Cla	No
Non Alcoh	High	Regular	Poor	Yes
Non Alcoh	High	Regular	High Class	Yes
Non Alcoh	High	Occasional	Poor	No
Non Alcoh	High	Regular	Middle Cla	Yes
Non Alcoh	High	Regular	High Class	Yes
Non Alcoh	High	Regular	Middle Cla	No
Occasional	High	Regular	Poor	No
Non Alcoh	High	Occasional	Poor	Yes
Non Alcoh	High	Regular	Middle Cla	No
Non Alcoh	High	Regular	Middle Cla	Yes
Occasional	High	Occasional	Middle Cla	Yes
Alcoholic	High	Regular	High Class	Yes
Alcoholic	High	Occasional	High Class	Yes
Non Alcoh	High	Regular	High Class	Yes
Non Alcoh	High	Regular	High Class	No
Non Alcoh	High	Regular	High Class	Yes
Non Alcoh	High	Occasional	Middle Cla	No
Non Alcoh	High	Regular	Middle Cla	No
Non Alcoh	High	Regular	Middle Cla	Yes
Occasional	High	Occasional	Middle Cla	No
Occasional	High	Non Smoke	Middle Cla	Yes
Non Alcoh	High	Occasional	Middle Cla	Yes
Non Alcoh	High	Regular	Poor	No
Non Alcoh	High	Regular	Poor	Yes
Non Alcoh	High	Occasional	Poor	No
Non Alcoh	High	Regular	Poor	Yes
Non Alcoh	High	Regular	High Class	No
Non Alcoh	High	Regular	High Class	No
Occasional	Low	Occasional	High Class	No

Non Alcoho	High	Non Smoke	Poor	No
Non Alcoho	High	Regular	High Class	Yes
Non Alcoho	High	Regular	Poor	No
Occasional	High	Regular	Middle Cla	No
Non Alcoho	High	Occasional	High Class	Yes
Non Alcoho	Moderate	Regular	Poor	Yes
Non Alcoho	Moderate	Regular	Poor	No
Non Alcoho	Moderate	Occasional	Poor	Yes
Non Alcoho	High	Regular	Poor	No
Non Alcoho	High	Regular	Middle Cla	Yes
Non Alcoho	High	Regular	Middle Cla	No
Non Alcoho	High	Occasional	Poor	Yes
Non Alcoho	High	Regular	High Class	Yes
Occasional	High	Regular	High Class	No
Non Alcoho	Low	Regular	Middle Cla	Yes
Non Alcoho	High	Non Smoke	High Class	No
Alcoholic	High	Occasional	Poor	No
Non Alcoho	Low	Regular	Poor	Yes
Non Alcoho	High	Regular	Poor	Yes
Non Alcoho	High	Occasional	Poor	No
Non Alcoho	Moderate	Regular	Poor	Yes
Non Alcoho	High	Occasional	High Class	No
Non Alcoho	High	Regular	Poor	No
Non Alcoho	High	Regular	Poor	No
Non Alcoho	High	Occasional	Middle Cla	Yes
Non Alcoho	High	Regular	Middle Cla	Yes
Non Alcoho	Moderate	Regular	High Class	Yes
Non Alcoho	High	Regular	Poor	Yes
Non Alcoho	Moderate	Occasional	Middle Cla	No
Non Alcoho	High	Regular	High Class	No
Non Alcoho	Moderate	Regular	Poor	No
Occasional	High	Regular	High Class	Yes

73	Male	176	70	107	69	Yes	Occasional	Occasional	No
25	Female	174	70	91	78	No	Following	Regular Ex	No
35	Female	159	78	109	81	No	Following	No Exercis	During Pre
40	Female	154	50	142	68	Yes	Occasional	Regular Ex	During Pre
40	Male	155	56	104	88	No	Following	No Exercis	Yes
76	Female	162	78	98	82	No	Occasional	Occasional	Yes
40	Female	174	60	121	71	Yes	Following	Regular Ex	During Pre
40	Male	172	61	145	92	No	Occasional	Occasional	No
38	Female	152	45	152	88	Yes	Following	No Exercis	Yes
50	Male	180	80	152	73	No	Following	Regular Ex	Yes
35	Male	165	88	120	88	No	Following	Regular Ex	No
26	Male	145	49	121	81	Yes	Following	Regular Ex	No
72	Male	179	70	99	85	Not Known	Not Follow	Regular Ex	No
72	Female	152	77	95	71	No	Not Follow	No Exercis	Yes
35	Male	155	54	121	77	Yes	Occasional	Regular Ex	Yes
26	Female	159	68	142	90	No	Not Follow	Occasional	No
50	Female	157	51	149	88	No	Following	No Exercis	No
50	Male	146	41	129	82	Not Known	Occasional	Regular Ex	Yes
24	Female	143	55	96	61	No	Not Follow	Regular Ex	No
69	Female	162	50	135	89	Yes	Following	Regular Ex	Yes
35	Male	157	53	120	78	No	Following	Occasional	Yes
29	Male	156	59	150	92	No	Following	No Exercis	No
50	Female	142	60	135	86	No	Occasional	Occasional	No
50	Male	167	62	131	71	Yes	Occasional	Regular Ex	Yes
23	Female	168	70	92	66	No	Occasional	Regular Ex	No
74	Female	170	78	138	80	Yes	Not Follow	No Exercis	Yes
39	Female	155	75	101	85	No	Occasional	Occasional	No
39	Male	177	60	108	75	Not Known	Following	Regular Ex	Yes
23	Female	143	50	91	65	Not Known	Not Follow	Regular Ex	No
37	Female	180	66	145	77	No	Not Follow	Occasional	Yes
78	Female	148	54	110	68	Not Known	Occasional	No Exercis	Yes
26	Male	172	72	132	77	No	Not Follow	Regular Ex	Yes
37	Female	145	48	118	78	Yes	Occasional	No Exercis	No
79	Male	153	57	131	78	No	Not Follow	No Exercis	No
27	Male	154	49	102	66	No	Not Follow	Occasional	No
82	Female	152	55	128	80	No	Occasional	No Exercis	No
23	Male	176	85	93	72	Yes	Occasional	Regular Ex	No
31	Male	174	72	104	80	No	Not Follow	No Exercis	No
34	Female	152	48	120	90	No	Occasional	Regular Ex	No
80	Female	155	52	105	98	Not Known	Following	No Exercis	No
38	Female	168	71	124	80	Not Known	Occasional	No Exercis	Yes
37	Male	156	62	112	71	Yes	Following	Regular Ex	No
26	Female	162	83	152	95	Yes	Following	Occasional	Yes
26	Female	160	55	112	95	No	Following	Regular Ex	Yes
28	Male	164	87	114	78	Yes	Occasional	Occasional	No
34	Female	155	59	118	70	Not Known	Following	Occasional	No
28	Female	162	84	132	66	No	Following	No Exercis	Yes

34	Female	168	56	108	84	No	Following	No Exercis	No
34	Female	165	87	132	71	Yes	Not Follow	No Exercis	Yes
22	Female	143	52	93	64	No	Following	Occasional	No
80	Male	167	62	123	80	Not Knowr	Following	No Exercis	No
24	Female	153	74	91	77	Not Knowr	Following	Regular Ex	No
24	Male	178	88	99	76	No	Following	No Exercis	No
34	Female	153	56	132	86	Yes	Not Follow	Occasional	Yes
34	Female	154	58	118	90	No	Occasional	Occasional	Yes
24	Female	146	62	90	90	No	Occasional	Occasional	During Pre
82	Female	174	60	114	90	Not Knowr	Not Follow	Occasional	Yes
24	Male	168	60	92	80	Yes	Not Follow	Regular Ex	No
81	Female	150	55	138	98	Not Knowr	Following	Occasional	No
28	Male	166	70	114	83	Not Knowr	Following	No Exercis	No
29	Male	173	90	129	78	No	Not Follow	No Exercis	Yes
29	Male	175	67	130	86	Yes	Following	Occasional	No
30	Male	166	90	105	88	No	Not Follow	No Exercis	No
30	Male	168	68	154	88	No	Following	Occasional	Yes
29	Female	152	51	152	70	Yes	Following	Regular Ex	Yes
25	Male	174	86	94	74	Yes	Following	No Exercis	No
25	Male	158	62	92	82	Yes	Not Follow	Occasional	No
25	Female	172	71	93	84	No	Not Follow	No Exercis	During Pre
25	Male	176	90	92	80	No	Not Follow	No Exercis	Yes
25	Female	167	65	100	80	No	Not Follow	Regular Ex	During Pre
25	Male	165	64	93	83	No	Not Follow	Occasional	Yes
25	Female	157	61	94	87	No	Following	Regular Ex	During Pre
25	Female	155	62	97	82	No	Following	Regular Ex	No
27	Female	170	68	100	80	No	Following	Regular Ex	Yes
27	Female	176	88	127	98	Not Knowr	Following	No Exercis	No
27	Female	153	52	109	81	No	Occasional	No Exercis	No
25	Female	157	71	152	66	Yes	Following	Regular Ex	No
29	Female	152	75	152	87	No	Following	Occasional	Yes
29	Female	158	60	145	82	No	Following	Occasional	No
29	Female	152	58	128	92	Yes	Following	Regular Ex	No
30	Female	153	52	120	88	No	Occasional	No Exercis	No
30	Female	158	55	142	89	Yes	Following	Regular Ex	During Pre
25	Female	148	53	92	78	Yes	Following	Regular Ex	Yes
32	Male	170	75	118	88	Not Knowr	Occasional	No Exercis	No
32	Male	176	101	106	95	No	Following	Regular Ex	Yes
33	Female	165	72	114	91	Yes	Occasional	Occasional	During Pre
33	Male	180	85	118	68	No	Following	Regular Ex	Yes
33	Male	152	55	110	90	No	Following	No Exercis	Yes
36	Female	158	75	128	75	Yes	Occasional	Regular Ex	Yes
36	Male	180	105	128	85	Not Knowr	Following	No Exercis	No
33	Male	180	102	99	69	Yes	Following	Regular Ex	No
36	Female	174	98	130	75	No	Following	Regular Ex	Yes
75	Female	180	64	109	77	Not Knowr	Following	No Exercis	No
23	Male	179	100	94	67	Not Knowr	Occasional	Regular Ex	No

23	Female	148	62	95	71	No	Occasional	No Exercis	Yes
34	Female	171	94	122	82	Yes	Following	Regular Ex	During Pre
43	Female	175	90	120	94	No	Following	Occasional	During Pre
43	Male	156	74	118	92	Yes	Occasional	No Exercis	Yes
44	Male	170	73	152	72	Yes	Occasional	No Exercis	No
45	Female	178	98	134	79	No	Following	No Exercis	During Pre
47	Female	178	101	137	83	No	Following	No Exercis	During Pre
47	Female	181	91	141	88	No	Occasional	Regular Ex	During Pre
49	Male	157	94	122	66	No	Following	Regular Ex	No
49	Female	180	101	132	86	No	Occasional	No Exercis	Yes
50	Male	178	94	121	85	No	Occasional	No Exercis	No
50	Female	172	60	142	79	No	Occasional	Regular Ex	Yes
50	Female	170	94	138	72	No	Following	Occasional	No
52	Female	176	97	125	77	Yes	Occasional	No Exercis	No
52	Female	175	102	128	78	No	Not Follow	Regular Ex	During Pre
52	Male	173	93	121	80	Yes	Occasional	Occasional	No
52	Female	174	91	104	87	No	Following	Regular Ex	No
54	Female	181	93	132	89	No	Following	Regular Ex	No
55	Female	177	102	132	90	No	Following	Regular Ex	No
55	Female	166	84	128	76	Not Known	Occasional	Regular Ex	No
55	Female	160	67	108	79	Yes	Occasional	Occasional	Yes
55	Male	164	88	102	85	No	Occasional	No Exercis	No
56	Male	170	94	121	90	No	Occasional	Regular Ex	Yes
56	Male	155	81	137	88	No	Occasional	Regular Ex	No
57	Female	170	65	99	99	No	Occasional	Occasional	No
57	Male	175	58	129	78	No	Following	Regular Ex	Yes
57	Female	167	90	129	65	No	Following	Regular Ex	No
58	Female	178	95	132	85	No	Occasional	Occasional	No
58	Female	178	91	123	81	No	Occasional	Occasional	No
59	Male	158	49	108	88	No	Not Follow	Regular Ex	No
59	Female	180	105	99	66	No	Following	Regular Ex	No
59	Male	168	88	114	69	No	Following	Regular Ex	No
59	Male	161	70	117	92	No	Following	Regular Ex	No
60	Male	164	67	120	77	No	Not Follow	Regular Ex	No
60	Male	160	101	113	67	No	Not Follow	No Exercis	No
60	Male	178	104	120	68	No	Not Follow	Regular Ex	No
61	Male	145	85	120	71	No	Following	Occasional	No
61	Male	165	79	109	73	No	Not Follow	Regular Ex	No
61	Male	167	98	116	79	No	Occasional	Regular Ex	Yes
62	Male	174	83	120	80	No	Following	Regular Ex	No
62	Female	152	59	110	88	No	Following	Occasional	Yes
62	Male	156	73	101	90	No	Following	No Exercis	No
62	Male	178	89	123	92	No	Not Follow	Occasional	Yes
62	Male	161	81	130	95	No	Following	Regular Ex	No
62	Male	178	79	131	90	No	Not Follow	No Exercis	No
62	Male	162	70	142	85	No	Occasional	Regular Ex	Yes
62	Female	169	119	145	88	No	Not Follow	No Exercis	Yes

63	Male	160	86	121	90	No	Not Follow	Occasional	No
63	Female	172	76	128	89	Yes	Not Follow	Regular Ex	No
63	Male	165	126	129	82	No	Following	Occasional	No
63	Female	160	52	145	81	No	Following	No Exercis	No
63	Female	155	61	132	82	Yes	Occasional	Occasional	Yes
63	Female	180	86	133	95	No	Occasional	Regular Ex	Yes
63	Female	180	100	136	97	No	Not Follow	Occasional	No
63	Female	180	92	139	95	Yes	Following	Regular Ex	No
64	Female	161	97	102	91	Yes	Not Follow	Occasional	Yes
64	Female	164	60	121	78	No	Not Follow	Occasional	No
64	Male	157	64	120	87	Yes	Not Follow	No Exercis	No
64	Female	175	78	130	90	Not Knowr	Following	No Exercis	Yes
64	Male	157	97	131	95	No	Following	No Exercis	Yes
64	Male	163	81	137	97	No	Following	No Exercis	No
64	Female	177	102	129	90	Not Knowr	Occasional	Regular Ex	Yes
64	Female	160	73	132	97	Yes	Occasional	Regular Ex	Yes
64	Male	176	84	142	91	No	Following	Occasional	No
65	Male	153	83	101	80	Yes	Following	No Exercis	No
65	Male	178	80	110	85	No	Occasional	Regular Ex	Yes
65	Male	157	99	112	88	Yes	Occasional	Regular Ex	No
65	Male	167	77	124	86	No	Following	No Exercis	Yes
65	Female	172	98	120	87	No	Not Follow	No Exercis	No
65	Female	179	100	118	89	No	Occasional	Regular Ex	No
65	Male	164	79	138	95	No	Following	Occasional	No
65	Female	174	88	140	97	No	Occasional	Regular Ex	No
66	Female	175	64	99	68	No	Following	Occasional	No
66	Male	175	99	105	95	Not Knowr	Occasional	No Exercis	Yes
66	Female	180	94	130	76	Not Knowr	Occasional	No Exercis	No
66	Female	179	86	132	79	No	Following	Regular Ex	No
66	Male	170	79	134	73	No	Not Follow	No Exercis	Yes
66	Female	181	78	128	71	Yes	Not Follow	Regular Ex	No
66	Male	167	80	129	75	Not Knowr	Not Follow	Occasional	Yes
66	Female	171	86	130	82	No	Following	Occasional	No
66	Male	167	68	145	80	Not Knowr	Following	Occasional	No
67	Male	155	66	118	85	No	Following	No Exercis	Yes
67	Female	170	80	117	75	No	Following	Occasional	Yes
67	Male	158	59	116	74	Yes	Following	Regular Ex	No
67	Male	161	78	109	73	No	Not Follow	Regular Ex	No
67	Female	177	121	132	71	No	Not Follow	Occasional	Yes
68	Female	160	97	101	68	No	Following	Regular Ex	No
68	Male	157	77	102	75	No	Occasional	Regular Ex	No
68	Female	165	65	110	71	No	Occasional	Occasional	No
68	Female	176	97	108	72	Yes	Not Follow	No Exercis	No
68	Male	167	58	121	85	No	Not Follow	Regular Ex	No
68	Male	160	75	102	88	No	Occasional	No Exercis	No
68	Male	157	62	101	95	Not Knowr	Occasional	No Exercis	Yes
68	Male	158	87	101	92	Not Knowr	Following	Occasional	Yes

68	Female	177	78	121	97	No	Not Follow	Regular Exe	No
68	Female	180	105	101	97	Not Known	Following	Regular Exe	Yes
68	Female	166	81	108	81	No	Occasional	Occasional	Yes
45	Male	157	56	112	91	Yes	Not Follow	No Exercise	No
48	Male	140	65	102	89	Yes	Not Follow	Regular Exe	Yes
47	Male	155	62	120	82	No	Not Follow	No Exercise	Yes
46	Male	145	69	119	81	Yes	Not Follow	Regular Exe	Yes
61	Male	148	81	142	95	Yes	Following	No Exercise	No
56	Female	153	83	130	74	Yes	Following	Occasional	Yes
66	Female	154	83	132	92	Yes	Following	Regular Exe	Yes
52	Female	165	84	96	83	Yes	Following	Regular Exe	No
62	Female	177	77	120	88	Yes	Following	No Exercise	Yes
58	Male	178	78	115	95	No	Not Follow	No Exercise	Yes
39	Male	179	77	135	92	Yes	Not Follow	No Exercise	No
59	Female	139	71	98	80	Yes	Following	Regular Exe	Yes
64	Male	165	77	121	78	No	Not Follow	Regular Exe	No
51	Female	167	75	131	89	Yes	Following	No Exercise	Yes
66	Male	156	73	101	81	No	Not Follow	Regular Exe	Yes
43	Female	145	83	152	78	Yes	Occasional	Regular Exe	Yes
42	Female	165	80	128	88	No	Occasional	Occasional	Yes
59	Male	155	90	121	94	Yes	Not Follow	No Exercise	Yes
58	Male	150	88	105	94	No	Not Follow	No Exercise	Yes
37	Female	145	81	135	80	Yes	Not Follow	No Exercise	During Pre
54	Male	148	82	134	84	Yes	Following	Regular Exe	Yes
57	Female	152	76	145	71	Yes	Not Follow	Occasional	Yes
47	Male	156	65	145	78	Yes	Not Follow	Regular Exe	Yes
44	Male	178	73	145	75	No	Not Follow	Regular Exe	Yes
48	Male	177	83	152	77	Yes	Not Follow	No Exercise	No
65	Male	177	79	102	95	No	Occasional	No Exercise	Yes
35	Male	145	78	101	71	Yes	Occasional	Regular Exe	No
41	Female	156	69	109	88	Yes	Occasional	Occasional	Yes
58	Male	174	63	123	68	Yes	Occasional	Regular Exe	No
62	Male	176	58	120	69	No	Not Follow	Occasional	Yes
65	Female	165	63	111	68	No	Not Follow	Regular Exe	Yes
34	Female	153	67	108	73	Yes	Following	Occasional	Yes
49	Male	155	79	110	88	Yes	Not Follow	Regular Exe	Yes
60	Male	157	54	110	78	Yes	Not Follow	No Exercise	No
43	Female	157	59	142	66	No	Not Follow	No Exercise	Yes
55	Female	158	61	98	87	Yes	Not Follow	Regular Exe	No
58	Female	161	89	121	78	No	Not Follow	No Exercise	No
44	Male	166	90	107	80	No	Following	Occasional	Yes
41	Female	143	67	125	82	Yes	Not Follow	No Exercise	Yes
54	Male	149	62	134	88	Yes	Following	Occasional	No
57	Male	154	71	129	74	Yes	Not Follow	No Exercise	No
60	Female	151	72	145	83	Yes	Occasional	Regular Exe	Yes
59	Male	169	66	128	92	Yes	Occasional	Occasional	No
48	Male	177	79	132	80	Yes	Not Follow	No Exercise	Yes

34	Female	159	91	99	94	Yes	Not Follow	Regular Ex	Yes
44	Female	174	88	120	88	Yes	Not Follow	Occasional	During Pre
62	Female	173	69	148	82	Yes	Not Follow	Regular Ex	Yes
41	Female	154	85	139	84	Yes	Not Follow	No Exercis	Yes
56	Female	157	76	118	81	Yes	Not Follow	Regular Ex	Yes
41	Female	159	71	105	88	No	Not Follow	Regular Ex	Yes
50	Female	140	72	121	94	Yes	Not Follow	Occasional	Yes
38	Female	152	77	149	75	No	Not Follow	No Exercis	Yes
44	Male	155	66	114	73	Yes	Not Follow	Regular Ex	Yes
71	Male	153	65	110	85	Yes	Not Follow	Occasional	Yes
65	Female	154	68	104	71	No	Not Follow	No Exercis	Yes
67	Male	165	69	119	92	Yes	Not Follow	No Exercis	No
41	Male	178	77	117	89	No	Not Follow	Regular Ex	No
44	Male	138	74	138	66	No	Not Follow	Regular Ex	Yes
53	Female	165	55	101	77	Yes	Not Follow	Occasional	Yes
61	Male	167	54	117	90	Yes	Not Follow	Regular Ex	Yes
59	Male	156	50	123	90	Yes	Not Follow	No Exercis	Yes
65	Female	155	51	135	84	Yes	Not Follow	Occasional	Yes
45	Male	178	52	107	82	Yes	Following	Regular Ex	No
51	Female	178	83	101	72	No	Not Follow	No Exercis	Yes
43	Female	179	67	117	77	Yes	Not Follow	Regular Ex	Yes
49	Female	145	66	123	78	No	Occasional	No Exercis	No
56	Female	165	58	101	87	Yes	Occasional	Regular Ex	Yes
42	Male	152	67	123	90	No	Occasional	No Exercis	Yes
41	Male	150	58	148	76	Yes	Occasional	Regular Ex	Yes
58	Male	148	79	142	88	No	Occasional	No Exercis	No
42	Male	152	92	104	95	No	Occasional	No Exercis	Yes
59	Male	157	54	105	99	Yes	Occasional	Regular Ex	Yes
52	Male	178	59	121	78	No	Occasional	Regular Ex	No
35	Male	177	61	149	65	No	Not Follow	Regular Ex	Yes
57	Female	177	90	101	88	Yes	Occasional	No Exercis	Yes
41	Male	176	64	100	71	No	Not Follow	Regular Ex	Yes
46	Male	179	67	120	92	Yes	Not Follow	Regular Ex	Yes
45	Male	174	62	121	81	Yes	Not Follow	Regular Ex	Yes
58	Male	165	88	111	75	No	Following	No Exercis	No
66	Male	177	54	101	90	Yes	Occasional	Regular Ex	No
63	Female	179	56	110	65	No	Following	Regular Ex	Yes
64	Male	177	67	132	80	Yes	Not Follow	Regular Ex	Yes
64	Male	176	65	132	85	No	Not Follow	No Exercis	No
41	Male	177	68	123	85	Yes	Not Follow	Regular Ex	Yes
46	Female	178	69	129	75	Yes	Occasional	Regular Ex	Yes
57	Male	179	73	118	76	Yes	Not Follow	No Exercis	No
48	Male	174	77	142	88	Yes	Occasional	Regular Ex	Yes
55	Male	167	79	131	77	Yes	Not Follow	No Exercis	No
44	Male	168	61	95	68	No	Following	Regular Ex	Yes
55	Male	156	55	131	69	Yes	Not Follow	No Exercis	Yes
65	Female	173	55	102	95	No	Not Follow	No Exercis	Yes

51	Female	156	52	128	92	Yes	Not Follow	Regular Ex Yes

Occasional	Moderate	Occasional	Poor		No
Non Alcoh	Low	Occasional	Poor		No
Occasional	Moderate	Occasional	Middle Cla		No
Occasional	Low	Occasional	Middle Cla		No
Alcoholic	Moderate	Non Smoke	Middle Cla		No
Occasional	Moderate	Regular	Middle Cla		No
Alcoholic	Moderate	Occasional	High Class		No
Alcoholic	High	Occasional	High Class		No
Alcoholic	High	Occasional	High Class		No
Alcoholic	High	Regular	Middle Cla		No
Non Alcoh	Low	Non Smoke	High Class		No
Non Alcoh	High	Occasional	High Class		No
Alcoholic	Moderate	Occasional	High Class		No
Alcoholic	Moderate	Regular	High Class		No
Non Alcoh	Low	Non Smoke	Poor		No
Non Alcoh	High	Regular	High Class		No
Occasional	High	Regular	High Class		No
Occasional	Low	Occasional	Middle Cla		No
Non Alcoh	High	Regular	High Class		No
Occasional	Moderate	Occasional	Middle Cla		No
Occasional	Moderate	Occasional	Middle Cla		No
Non Alcoh	High	Regular	High Class		No
Non Alcoh	Moderate	Regular	Poor		No
Alcoholic	High	Regular	High Class		No
Non Alcoh	Moderate	Non Smoke	High Class		No
Alcoholic	Low	Occasional	Poor		No
Occasional	Moderate	Occasional	Poor		No
Alcoholic	High	Regular	Poor		No
Occasional	High	Regular	Poor		No
Occasional	Moderate	Regular	Middle Cla		No
Non Alcoh	Moderate	Non Smoke	Middle Cla		No
Alcoholic	Moderate	Regular	High Class		No
Occasional	High	Regular	Poor		No
Non Alcoh	High	Occasional	Middle Cla		No
Occasional	High	Occasional	Poor		No
Non Alcoh	Low	Non Smoke	Middle Cla		No
Occasional	Moderate	Occasional	High Class		No
Non Alcoh	Moderate	Regular	Poor		No
Non Alcoh	Low	Non Smoke	Poor		No
Non Alcoh	Moderate	Non Smoke	Middle Cla		No
Non Alcoh	High	Regular	High Class		No
Non Alcoh	Moderate	Occasional	Middle Cla		No
Non Alcoh	Moderate	Occasional	Poor		No
Non Alcoh	Moderate	Non Smoke	Middle Cla		No
Occasional	Low	Occasional	Poor		No
Occasional	High	Occasional	Middle Cla		No
Alcoholic	Moderate	Non Smoke	Poor		No

Non Alcoh	Moderate	Regular	Poor	No
Non Alcoh	High	Regular	High Class	No
Alcoholic	High	Regular	High Class	No
Non Alcoh	Moderate	Non Smoke	High Class	No
Occasional	Moderate	Occasional	Middle Cla	No
Occasional	High	Regular	Poor	No
Non Alcoh	Low	Non Smoke	High Class	No
Occasional	Low	Occasional	High Class	No
Alcoholic	Low	Non Smoke	High Class	No
Non Alcoh	Low	Non Smoke	Middle Cla	No
Alcoholic	High	Regular	Middle Cla	No
Non Alcoh	Moderate	Non Smoke	High Class	No
Alcoholic	High	Regular	Poor	No
Non Alcoh	Low	Non Smoke	High Class	No
Occasional	Moderate	Occasional	High Class	No
Non Alcoh	Low	Non Smoke	Middle Cla	No
Occasional	Moderate	Occasional	Poor	No
Non Alcoh	Low	Non Smoke	Poor	No
Alcoholic	High	Regular	Middle Cla	No
Occasional	Moderate	Regular	Middle Cla	No
Non Alcoh	High	Non Smoke	Middle Cla	No
Alcoholic	High	Regular	Poor	No
Occasional	High	Non Smoke	Poor	No
Occasional	Moderate	Non Smoke	High Class	No
Occasional	High	Regular	High Class	No
Alcoholic	High	Regular	Middle Cla	No
Non Alcoh	Low	Non Smoke	Middle Cla	No
Occasional	High	Regular	Middle Cla	No
Non Alcoh	High	Occasional	Middle Cla	No
Occasional	Moderate	Occasional	Poor	No
Non Alcoh	High	Occasional	Poor	No
Alcoholic	Moderate	Occasional	Middle Cla	No
Non Alcoh	Low	Non Smoke	Middle Cla	No
Non Alcoh	Low	Occasional	Poor	No
Occasional	Moderate	Regular	High Class	No
Non Alcoh	Moderate	Occasional	Poor	No
Non Alcoh	High	Non Smoke	Poor	No
Occasional	High	Regular	Poor	No
Occasional	Moderate	Occasional	Poor	No
Alcoholic	Moderate	Occasional	High Class	No
Non Alcoh	Moderate	Regular	Middle Cla	No
Non Alcoh	Moderate	Regular	High Class	No
Non Alcoh	Low	Non Smoke	Poor	No
Occasional	Low	Non Smoke	High Class	No
Alcoholic	High	Occasional	Poor	No
Alcoholic	Moderate	Occasional	Middle Cla	No
Non Alcoh	Low	Regular	Poor	No

Non Alcoho	Moderate	Occasional	Middle Cla	No
Non Alcoho	Low	Non Smoke	Middle Cla	No
Alcoholic	High	Regular	High Class	No
Alcoholic	Low	Non Smoke	Middle Cla	No
Non Alcoho	High	Regular	High Class	No
Non Alcoho	Low	Non Smoke	Poor	No
Occasional	Low	Non Smoke	High Class	No
Non Alcoho	Low	Occasional	High Class	No
Alcoholic	High	Regular	High Class	No
Alcoholic	Low	Regular	Poor	No
Non Alcoho	Low	Non Smoke	High Class	No
Occasional	Low	Non Smoke	Poor	No
Alcoholic	Low	Non Smoke	Poor	No
Occasional	High	Occasional	High Class	No
Non Alcoho	Moderate	Non Smoke	Poor	No
Alcoholic	Low	Non Smoke	Poor	No
Alcoholic	Low	Non Smoke	Middle Cla	No
Alcoholic	Low	Regular	High Class	No
Alcoholic	Low	Non Smoke	Middle Cla	No
Occasional	Low	Regular	Poor	No
Occasional	Low	Regular	Poor	No
Alcoholic	Low	Regular	High Class	No
Non Alcoho	Moderate	Occasional	High Class	No
Occasional	Low	Non Smoke	High Class	No
Alcoholic	Low	Occasional	High Class	No
Non Alcoho	Moderate	Non Smoke	Poor	No
Occasional	High	Non Smoke	Poor	No
Non Alcoho	Moderate	Regular	High Class	No
Non Alcoho	Low	Occasional	High Class	No
Alcoholic	Low	Non Smoke	High Class	No
Alcoholic	Low	Occasional	High Class	No
Alcoholic	Low	Occasional	Poor	No
Alcoholic	Low	Occasional	Poor	No
Non Alcoho	Low	Occasional	High Class	No
Non Alcoho	Low	Regular	Poor	No
Non Alcoho	Low	Occasional	High Class	No
Alcoholic	Low	Non Smoke	Poor	No
Non Alcoho	Low	Non Smoke	High Class	No
Occasional	Moderate	Regular	Poor	No
Alcoholic	Low	Occasional	Poor	No
Alcoholic	Moderate	Regular	Middle Cla	No
Non Alcoho	Low	Regular	Middle Cla	No
Occasional	Low	Occasional	Poor	No
Non Alcoho	Low	Occasional	High Class	No
Alcoholic	Low	Regular	Middle Cla	No
Occasional	Low	Regular	High Class	No
Occasional	Low	Occasional	Poor	No

Non Alcoho	Low	Occasional	Poor	No
Non Alcoho	Moderate	Non Smoke	Poor	No
Occasional	High	Non Smoke	Middle Cla	No
Non Alcoho	Moderate	Regular	High Class	No
Occasional	High	Regular	Poor	No
Non Alcoho	Low	Occasional	Middle Cla	No
Alcoholic	Moderate	Non Smoke	Poor	No
Alcoholic	Low	Occasional	High Class	No
Non Alcoho	Moderate	Non Smoke	Middle Cla	No
Alcoholic	Low	Non Smoke	High Class	No
Non Alcoho	Low	Regular	Poor	No
Alcoholic	Low	Regular	High Class	No
Occasional	Moderate	Occasional	High Class	No
Non Alcoho	High	Occasional	High Class	No
Non Alcoho	Low	Occasional	High Class	No
Alcoholic	Moderate	Occasional	High Class	No
Non Alcoho	Low	Regular	High Class	No
Non Alcoho	Low	Non Smoke	Poor	No
Non Alcoho	Low	Non Smoke	Poor	No
Occasional	Low	Non Smoke	High Class	No
Occasional	Low	Non Smoke	Poor	No
Alcoholic	Low	Non Smoke	Poor	No
Alcoholic	Low	Non Smoke	High Class	No
Occasional	Low	Non Smoke	Middle Cla	No
Occasional	Low	Non Smoke	High Class	No
Non Alcoho	Low	Non Smoke	Poor	No
Occasional	Low	Non Smoke	Middle Cla	No
Occasional	Low	Non Smoke	High Class	No
Alcoholic	Low	Non Smoke	High Class	No
Alcoholic	Moderate	Occasional	Poor	No
Alcoholic	Low	Non Smoke	High Class	No
Occasional	Low	Non Smoke	High Class	No
Alcoholic	High	Non Smoke	High Class	No
Non Alcoho	Low	Non Smoke	High Class	No
Non Alcoho	Low	Regular	High Class	No
Non Alcoho	Low	Non Smoke	Middle Cla	No
Occasional	Low	Non Smoke	Middle Cla	No
Occasional	Low	Non Smoke	Middle Cla	No
Occasional	Moderate	Non Smoke	Middle Cla	No
Alcoholic	Low	Occasional	Poor	No
Alcoholic	Low	Occasional	Poor	No
Non Alcoho	Low	Occasional	Poor	No
Non Alcoho	Low	Regular	High Class	No
Occasional	High	Occasional	Poor	No
Occasional	Low	Occasional	High Class	No
Alcoholic	Moderate	Non Smoke	Poor	No
Occasional	High	Non Smoke	Middle Cla	No

Non Alcoho	Low	Non Smoke	Middle Cla	No
Occasional	Moderate	Occasional	Middle Cla	No
Non Alcoho	Low	Regular	High Class	No
Occasional	High	Regular	High Class	No
Non Alcoho	Low	Regular	Poor	No
Non Alcoho	Low	Occasional	Middle Cla	No
Non Alcoho	Low	Regular	Poor	No
Occasional	High	Regular	Poor	No
Non Alcoho	High	Occasional	Middle Cla	No
Non Alcoho	High	Regular	Poor	No
Non Alcoho	High	Regular	High Class	No
Non Alcoho	High	Regular	High Class	No
Non Alcoho	High	Non Smoke	Middle Cla	No
Occasional	High	Regular	Middle Cla	No
Non Alcoho	High	Regular	Middle Cla	No
Non Alcoho	High	Regular	Middle Cla	No
Non Alcoho	High	Regular	Middle Cla	No
Non Alcoho	Low	Occasional	High Class	No
Non Alcoho	High	Occasional	Middle Cla	No
Non Alcoho	High	Regular	Middle Cla	No
Occasional	High	Occasional	Poor	No
Non Alcoho	Low	Regular	Middle Cla	No
Non Alcoho	High	Regular	High Class	No
Occasional	High	Regular	High Class	No
Non Alcoho	High	Regular	Middle Cla	No
Non Alcoho	Low	Regular	Middle Cla	No
Non Alcoho	Low	Regular	Middle Cla	No
Occasional	Low	Occasional	Poor	No
Occasional	Low	Regular	High Class	No
Non Alcoho	Low	Occasional	High Class	No
Alcoholic	High	Regular	High Class	No
Occasional	High	Regular	High Class	No
Non Alcoho	High	Occasional	Poor	No
Non Alcoho	High	Regular	Middle Cla	No
Non Alcoho	High	Occasional	Middle Cla	No
Non Alcoho	High	Occasional	High Class	No
Non Alcoho	Low	Regular	Poor	No
Non Alcoho	High	Regular	Middle Cla	No
Non Alcoho	High	Regular	Middle Cla	No
Non Alcoho	High	Occasional	Middle Cla	No
Alcoholic	High	Regular	High Class	No
Non Alcoho	High	Regular	High Class	No
Occasional	High	Occasional	High Class	No
Non Alcoho	Low	Regular	Poor	No
Non Alcoho	Low	Occasional	Middle Cla	No
Non Alcoho	High	Regular	Poor	No
Occasional	High	Occasional	Poor	No

Non Alcoh	High	Non Smoke	Poor	No
Non Alcoh	High	Occasional	High Class	No
Non Alcoh	Low	Regular	High Class	No
Non Alcoh	High	Occasional	Poor	No
Non Alcoh	Low	Regular	Middle Cla	No
Non Alcoh	High	Regular	Middle Cla	No
Occasional	High	Regular	High Class	No
Non Alcoh	High	Regular	Poor	No
Non Alcoh	High	Occasional	High Class	No
Non Alcoh	High	Occasional	Poor	No
Non Alcoh	High	Regular	Poor	No
Non Alcoh	Low	Regular	Poor	No
Occasional	Low	Regular	High Class	No
Non Alcoh	High	Regular	High Class	No
Occasional	High	Occasional	High Class	No
Non Alcoh	High	Regular	Poor	No
Non Alcoh	High	Regular	Middle Cla	No
Non Alcoh	Low	Regular	High Class	No
Occasional	Low	Occasional	High Class	No
Non Alcoh	Moderate	Occasional	Poor	No
Non Alcoh	High	Regular	Middle Cla	No
Occasional	Moderate	Regular	High Class	No
Non Alcoh	High	Occasional	Poor	No
Non Alcoh	High	Regular	High Class	No
Non Alcoh	High	Regular	High Class	No
Non Alcoh	High	Occasional	High Class	No
Non Alcoh	Moderate	Regular	Poor	No
Non Alcoh	High	Occasional	Poor	No
Alcoholic	Moderate	Regular	High Class	No
Non Alcoh	Moderate	Regular	Poor	No
Non Alcoh	High	Occasional	Poor	No
Non Alcoh	High	Regular	Poor	No
Non Alcoh	High	Regular	High Class	No
Non Alcoh	High	Regular	Poor	No
Non Alcoh	Moderate	Regular	Poor	No
Occasional	Moderate	Regular	Middle Cla	No
Non Alcoh	High	Regular	Middle Cla	No
Non Alcoh	High	Regular	High Class	No
Non Alcoh	High	Occasional	Poor	No
Non Alcoh	High	Regular	High Class	No
Non Alcoh	High	Occasional	High Class	No
Non Alcoh	High	Regular	Middle Cla	No
Non Alcoh	Moderate	Regular	High Class	No
Non Alcoh	High	Occasional	High Class	No
Non Alcoh	High	Regular	High Class	No
Non Alcoh	Moderate	Regular	Middle Cla	No
Non Alcoh	High	Regular	Poor	No

Non Alcoh	High	Regular	High Class	No
